# 29th Annual Computational Neuroscience Meeting: CNS*2020

**DOI:** 10.1186/s12868-020-00593-1

**Published:** 2020-12-21

## K1 Deep reinforcement learning and its neuroscientific implications

### Matthew Botvinick

#### Google DeepMind, Neuroscience Research, London, United Kingdom

##### **Correspondence:** Matthew Botvinick (botvinick@google.com)

*BMC Neuroscience* 2020, **21(Suppl 1)**:K1

The last few years have seen some dramatic developments in artificial intelligence research. What implications might these have for neuroscience? Investigations of this question have, to date, focused largely on deep neural networks trained using supervised learning, in tasks such as image classification. However, there is another area of recent AI work which has so far received less attention from neuroscientists, but which may have more profound neuroscientific implications: deep reinforcement learning. Deep RL offers a rich framework for studying the interplay among learning, representation and decision-making, offering to the brain sciences a new set of research tools and a wide range of novel hypotheses. I’ll provide a high-level introduction to deep RL, discuss some recent neuroscience-oriented investigations from my group at DeepMind, and survey some wider implications for research on brain and behavior.

## K2 A new computational framework for understanding vision in our brain

### Zhaoping Li

#### Max Planck Institute for Biological Cybernetics, Tübingen, Germany

##### **Correspondence:** Zhaoping Li (li.zhaoping@tuebingen.mpg.de)

*BMC Neuroscience* 2020, **21(Suppl 1)**:K2

Visual attention selects only a tiny fraction of visual input information for further processing. Selection starts in the primary visual cortex (V1), which creates a bottom-up saliency map to guide the fovea to selected visual locations via gaze shifts. This motivates a new framework that views vision as consisting of encoding, selection, and decoding stages, placing selection on center stage. It suggests a massive loss of non-selected information from V1 downstream along the visual pathway. Hence, feedback from downstream visual cortical areas to V1 for better decoding (recognition), through analysis-by-synthesis, should query for additional information and be mainly directed at the foveal region. Accordingly, non-foveal vision is not only poorer in spatial resolution, but also more susceptible to many illusions.

## K3 Information and decision-making

### Daniel Polani

#### University of Hertfordshire, School of Computer Science, Hatfield, United Kingdom

##### **Correspondence:** Daniel Polani (d.polani@herts.ac.uk)

*BMC Neuroscience* 2020, **21(Suppl 1)**:K3

In recent years it has become increasingly clear that (Shannon) information is a central resource for organisms, akin in importance to energy. Any decision that an organism or a subsystem of an organism takes involves the acquisition, selection, and processing of information and ultimately its concentration and enaction. It is the consequences of this balance that will occupy us in this talk.

This perception-action loop picture of an agent’s life cycle is well established and expounded especially in the context of Fuster’s sensorimotor hierarchies. Nevertheless, the information-theoretic perspective drastically expands the potential and predictive power of the perception-action loop perspective.

On the one hand information can be treated - to a significant extent - as a resource that is being sought and utilized by an organism. On the other hand, unlike energy, information is not additive. The intrinsic structure and dynamics of information can be exceedingly complex and subtle; in the last two decades one has discovered that Shannon information possesses a rich and nontrivial intrinsic structure that must be taken into account when informational contributions, information flow or causal interactions of processes are investigated, whether in the brain or in other complex processes.

In addition, strong parallels between information and control theory have emerged. This parallelism between the theories allows one to obtain unexpected insights into the nature and properties of the perception-action loop. Through the lens of information theory, one can not only come up with novel hypotheses about necessary conditions for the organization of information processing in a brain, but also with constructive conjectures and predictions about what behaviours, brain structure and dynamics and even evolutionary pressures one can expect to operate on biological organisms, induced purely by informational considerations.

## K4 Computational models of neural development

### Geoffrey J. Goodhill

#### University of Queensland, Queensland Brain Institute; School of Mathematics and Physics, St Lucia, Australia

##### **Correspondence:** Geoffrey J Goodhill (g.goodhill@uq.edu.au)

*BMC Neuroscience* 2020, **21(Suppl 1)**:K4

Unlike even the most sophisticated current forms of artificial intelligence, developing biological organisms must build their neural hardware from scratch. Furthermore they must start to evade predators and find food before this construction process is complete. I will discuss an interdisciplinary program of mathematical and experimental work which addresses some of the computational principles underlying neural development. This includes (i) how growing axons navigate to their targets by detecting and responding to molecular cues in their environment, (ii) the formation of maps in the visual cortex and how these are influenced by visual experience, and (iii) how patterns of neural activity in the zebrafish brain develop to facilitate precisely targeted hunting behaviour. Together this work contributes to our understanding of both normal neural development and the etiology of neurodevelopmental disorders.

## F1 Delineating reward/avoidance decision process in the impulsive-compulsive spectrum disorders through a probabilistic reversal learning task

### Xiaoliu Zhang^1^, Chao Suo^2^, Amir Dezfouli^3^, Ben J Harrison^4^, Leah Braganza^2^, Ben Fulcher^2^, Lenardo Fontenelle^2^, Carsten Murawski^5^, Murat Yucel^2^

#### ^1^Monash University, Monash Biomedical Imaging, Melbourne, Australia; ^2^Monash University, BrainPark, Turner Institute for Brian and Mental Health, School of Psychological Science, Melbourne, Australia; ^3^Machine Learning Research Group, Data61, CSIRO, Sydney, Australia; ^4^University of Melbourne and Melbourne Health, Melbourne Neuropsychiatry Centre, Department of Psychiatry, Melbourne, Australia; ^5^University of Melbourne, Department of Finance, Melbourne, Australia

##### **Correspondence:** Xiaoliu Zhang (smile.in.sjtu@gmail.com)

*BMC Neuroscience* 2020, **21(Suppl 1)**:F1

Impulsivity and compulsivity are behavioural traits that underlie many aspects of decision-making and form the characteristic symptoms of Obsessive Compulsive Disorder (OCD) and Gambling Disorder (GD). The neural underpinnings of aspects of reward and avoidance learning under the expression of these traits and symptoms are only partially understood.

The present study combined behavioural modelling and neuroimaging technique to examine brain activity associated with critical phases of reward and loss processing in OCD and GD.

Forty-two healthy controls (HC), forty OCD and twenty-three GD participants were recruited in our study to complete a two-session reinforcement learning (RL) task featuring a “probability switch (PS)” with imaging scanning. Finally, 39 HC (20F/19M, 34 yrs±9.47), 28 OCD (14F/14M, 32.11 yrs±9.53) and 16 GD (4F/12M, 35.53yrs±12.20) were included with both behavioural and imaging data available. The functional imaging wasconducted by using 3.0-T SIEMENS MAGNETOM Skyra syngo MR D13C at Monash Biomedical Imaging. Each volume compromised 34 coronal slices of 3 mm thickness with 2000ms TR and 30ms TE. A total of 479 volumes were acquired for each participant in each session in an interleaved-ascending manner.

The standard Q-learning model was fitted to the observed behavioural data and the Bayesian model was used for the parameter estimation. Imaging analysis was conducted using SPM12 (Welcome Department of Imaging Neuroscience, London, United Kingdom) in the Matlab (R2015b) environment. The pre-processing commenced with the slice timing, realignment, normalization to MNI space according to T1-weighted image and smoothing with a 8 mm Gaussian kernel.

The frontostriatal brain circuit including the *putamen and* medial orbitofrontal (mOFC) were significantly more active in response to receiving reward and avoiding punishment compared to receiving an aversive outcome and missing reward at *p < 0.001* with FEW correction at cluster level; While the *right insula* showed greater activation in response to missing rewards and receiving punishment. Compared to healthy participants, GD patients showed significantly lower activation in the *left superior frontal* and *posterior cingulum* at *p < 0.001* for the gain omission.

The reward prediction error (PE) signal was found positively correlated with the activation at several clusters expanding across cortical and subcortical region including *the striatum, cingulate, bilateral insula, thalamus* and *superior frontal* at *p < 0.001* with FWE correction at cluster level. The GD patients showed a trend of decreased reward PE response in the *right precentral* extending to *left posterior cingulate* compared to controls at *p < 0.05* with FWE correction. The aversive PE signal was negatively correlated with brain activity in regions including *bilateral thalamus, hippocampus, insula and striatum* at *p < 0.001* with FWE correction. Compared with the control group, GD group showed an increased aversive PE activation in the cluster encompassing *right thalamus* and *right hippocampus*, and also the *right middle frontal* extending to the *right anterior cingulum* at *p < 0.005* with FWE correction.

Through the reversal learning task, the study provided further support of the dissociable brain circuits for distinct phases of reward and avoidance learning. Also, the OCD and GD are characterised by aberrant patterns of reward and avoidance processing.

## F2 Using evolutionary algorithms to explore single-cell heterogeneity and microcircuit operation in the hippocampus

### Andrea Navas-Olive, Liset M de la Prida

#### Cajal Institute, Madrid, Spain

##### **Correspondence:** Andrea Navas-Olive (acnavasolive@gmail.com)

*BMC Neuroscience* 2020, **21(Suppl 1)**:F2

The hippocampus-entorhinal system is critical for learning and memory. Recent cutting-edge single-cell technologies from RNAseq to electrophysiology are disclosing a so far unrecognized heterogeneity within the major cell types [1]. Surprisingly, massive high-throughput recordings of these very same cells identify low dimensional microcircuit dynamics [2,3]. Reconciling both views is critical to understand how the brain operates.

The CA1 region is considered high in the hierarchy of the entorhinal-hippocampal system. Traditionally viewed as a single layered structure, recent evidence has disclosed an exquisite laminar organization across deep and superficial pyramidal sublayers at the transcriptional, morphological and functional levels [1,4,5]. Such a low-dimensional segregation may be driven by a combination of intrinsic, biophysical and microcircuit factors but mechanisms are unknown.

Here, we exploit evolutionary algorithms to address the effect of single-cell heterogeneity on CA1 pyramidal cell activity [6]. First, we developed a biophysically realistic model of CA1 pyramidal cells using the Hodgkin-Huxley multi-compartment formalism in the Neuron+Python platform and the morphological database Neuromorpho.org. We adopted genetic algorithms (GA) to identify passive, active and synaptic conductances resulting in realistic electrophysiological behavior. We then used the generated models to explore the functional effect of intrinsic, synaptic and morphological heterogeneity during oscillatory activities. By combining results from all simulations in a logistic regression model we evaluated the effect of up/down-regulation of different factors. We found that multidimensional excitatory and inhibitory inputs interact with morphological and intrinsic factors to determine a low dimensional subset of output features (e.g. phase-locking preference) that matches non-fitted experimental data (Fig. 1).

**Fig. 1:**
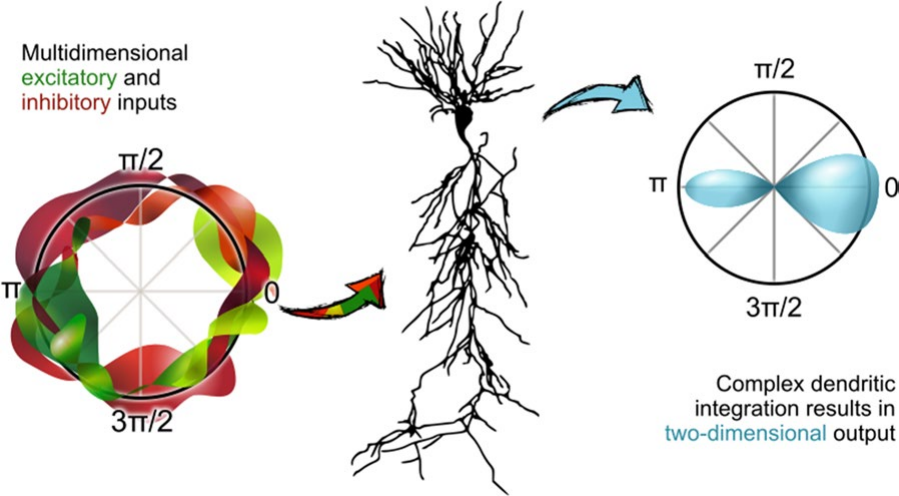
Conceptualization of mechanisms operating over intrinsic and synaptic factors to restrict neuronal firing of CA1 pyramidal cells during theta oscillations. Non-linear dendritic integration of multidimensional excitatory and inhibitory inputs results in segregated firing

**Acknowledgments:** Andrea Navas-Olive is supported by PhD Fellowship FPU17/03268.

**References**

1. Cembrowski MS, Spruston N. Heterogeneity within classical cell types is the rule: lessons from hippocampal pyramidal neurons. Nature Reviews Neuroscience. 2019; 20(4): 193-204.
2. Chaudhuri R, Gerçek B, Pandey B, Peyrache A, Fiete I. The intrinsic attractor manifold and population dynamics of a canonical cognitive circuit across waking and sleep. Nature Neuroscience. 2019; 22(9): 1512-1520.
3. Guo W, Zhang JJ, Newman JP, Wilson MA. Latent learning drives sleep-dependent plasticity in distinct CA1 subpopulations. bioRxiv. 2020; Preprint at: https://doi.org/10.1101/2020.02.27.967794
4. Bannister NJ, Larkman AU. Dendritic morphology of CA1 pyramidal neurones from the rat hippocampus: I. Branching patterns. Journal of Comparative Neurology. 1995; 360: 150–160.
5. Valero M, et al. Determinants of different deep and superficial CA1 pyramidal cell dynamics during sharp-wave ripples. Nature Neuroscience. 2015; 18(9): 1281-1290.
6. Navas-Olive A, et al. Multimodal determinants of phase-locked dynamics across deep-superficial hippocampal sublayers during theta oscillations. Nature Communications. 2020; 11(1): 1-4.

## F3 Neuronal morphology imposes a tradeoff between stability, accuracy and efficiency of synaptic scaling

### Adriano Bellotti, Saeed Aljaberi, Fulvio Forni, Timothy O’Leary

#### University of Cambridge, Department of Engineering, Cambridge, United Kingdom

##### **Correspondence:** Adriano Bellotti (adriano.bellotti@gmail.com)

*BMC Neuroscience* 2020, **21(Suppl 1)**:F3

Synaptic scaling is a homeostatic normalization mechanism that preserves relative synaptic strengths by adjusting them with a common factor. This multiplicative change is believed to be critical, since synaptic strengths are involved in learning and memory retention. Further, this homeostatic process is thought to be crucial for neuronal stability, playing a stabilizing role in otherwise runaway Hebbian plasticity [1-3]. Synaptic scaling requires a mechanism to sense total neuron activity and globally adjust synapses to achieve some activity set-point [4]. This process is relatively slow, which places limits on its ability to stabilize network activity [5]. Here we show that this slow response is inevitable in realistic neuronal morphologies. Furthermore, we reveal that global scaling can in fact be a source of instability unless responsiveness or scaling accuracy are sacrificed.

A neuron with tens of thousands of synapses must regulate its own excitability to compensate for changes in input. The time requirement for global feedback can introduce critical phase lags in a neuron’s response to perturbation. The severity of phase lag increases with neuron size. Further, a more expansive morphology worsens cell responsiveness and scaling accuracy, especially in distal regions of the neuron. Local pools of reserve receptors improve efficiency, potentiation, and scaling, but this comes at a cost. Trafficking large quantities of receptors requires time, exacerbating the phase lag and instability. Local homeostatic feedback mitigates instability, but this too comes at the cost of reducing scaling accuracy.

Realization of the phase lag instability requires a unified model of synaptic scaling, regulation, and transport. We present such a model with global and local feedback in realistic neuron morphologies (Fig. 1). This combined model shows that neurons face a tradeoff between stability, accuracy, and efficiency. Global feedback is required for synaptic scaling but favors either system stability or efficiency. Large receptor pools improve scaling accuracy in large morphologies but worsen both stability and efficiency. Local feedback improves the stability-efficiency tradeoff at the cost of scaling accuracy. This project introduces unexplored constraints on neuron size, morphology, and synaptic scaling that are weakened by an interplay between global and local feedback.

**Fig. 1:**
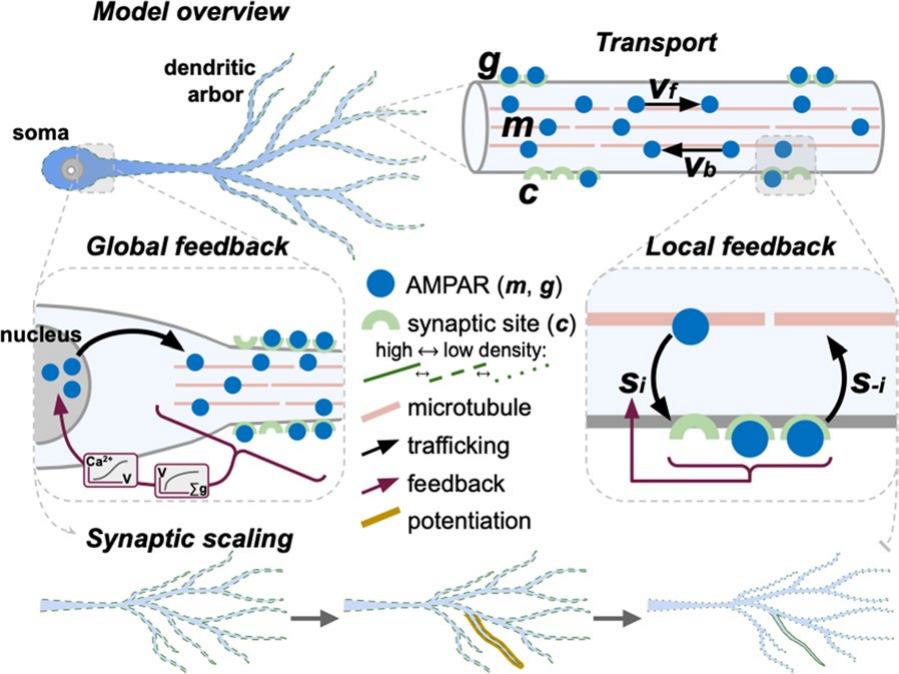
Schematic representation of unified model of AMPA receptor transport, potentiation, and scaling with global and local feedback

**Acknowledgements:** The authors are supported by European Research Council Grant FLEXNEURO (716643), Abu Dhabi National Oil Company, NIH OxCam Scholars program and Gates Cambridge Trust.

**References**

1. Royer S, Paré D. Conservation of total synaptic weight through balanced synaptic depression and potentiation. Nature. 2003; 422(6931): 518-522.
2. Chen JY, et al. Heterosynaptic plasticity prevents runaway synaptic dynamics. Journal of Neuroscience. 2013; 33(40): 15915-15929.
3. Chistiakova M, Bannon NM, Chen JY, Bazhenov M, Volgushev M. Homeostatic role of heterosynaptic plasticity: models and experiments. Frontiers in Computational Neuroscience. 2015; 9: 89.
4. Turrigiano GG. The self-tuning neuron: synaptic scaling of excitatory synapses. Cell. 2008; 135(3): 422-435.
5. Zenke F, Hennequin G, Gerstner W. Synaptic plasticity in neural networks needs homeostasis with a fast rate detector. PLoS Computational Biology. 2013; 9(11).

## F4 Who can turn faster? Comparison of the head direction circuit of two species

### Ioannis Pisokas^1^, Stanley Heinze^2^, Barbara Webb^1^

#### ^1^University of Edinburgh, School of Informatics, Edinburgh, United Kingdom; ^2^Lund University, Department of Biology, Lund, Sweden

##### **Correspondence:** Ioannis Pisokas (i.pisokas@sms.ed.ac.uk)

*BMC Neuroscience* 2020, **21(Suppl 1)**:F4

Ants, bees and other insects have the ability to return to their nest or hive using a navigation strategy known as path integration. Similarly, fruit flies employ path integration to return to a previously visited food source. An important component of path integration is the ability of the insect to keep track of its heading relative to salient visual cues. A highly conserved brain region known as the central complex has been identified as being of key importance for the computations required for an insect to keep track of its heading [1,2]. However, the similarities or differences of the underlying heading tracking circuit between species are not well understood. We sought to address this shortcoming by using reverse engineering techniques to derive the effective underlying neuronal circuits of two evolutionary distant species, the fruit fly and the locust. Our analysis revealed that regardless of the anatomical differences between the two species the essential circuit structure has not changed. Both effective neuronal circuits have the structural topology of a ring attractor with an eight-fold radial structure (Fig. 1). However, despite the strong similarities between the two ring attractors, there remain differences. Using computational modelling we found that two apparently small anatomical differences have significant functional effect on the ability of the two circuits to track fast rotational movements and to maintain a stable heading signal [6]. In particular, the fruit fly circuit responds faster to abrupt heading changes of the animal while the locust circuit maintains a heading signal that is more robust to inhomogeneities in cell membrane properties and synaptic weights. We suggest that the effects of these differences are consistent with the behavioural ecology of the two species. On the one hand, the faster response of the ring attractor circuit in the fruit fly accommodates the fast body saccades that fruit flies are known to perform. On the other hand, the locust is a migratory species, so its behaviour demands maintenance of a defined heading for a long period of time. Our results highlight that even seemingly small differences in the distribution of dendritic fibres can have a significant effect on the dynamics of the effective ring attractor circuit with consequences for the behavioural capabilities of each species. These differences, emerging from morphologically distinct single neurons highlight the importance of a comparative approach to neuroscience.

**Fig. 1:**
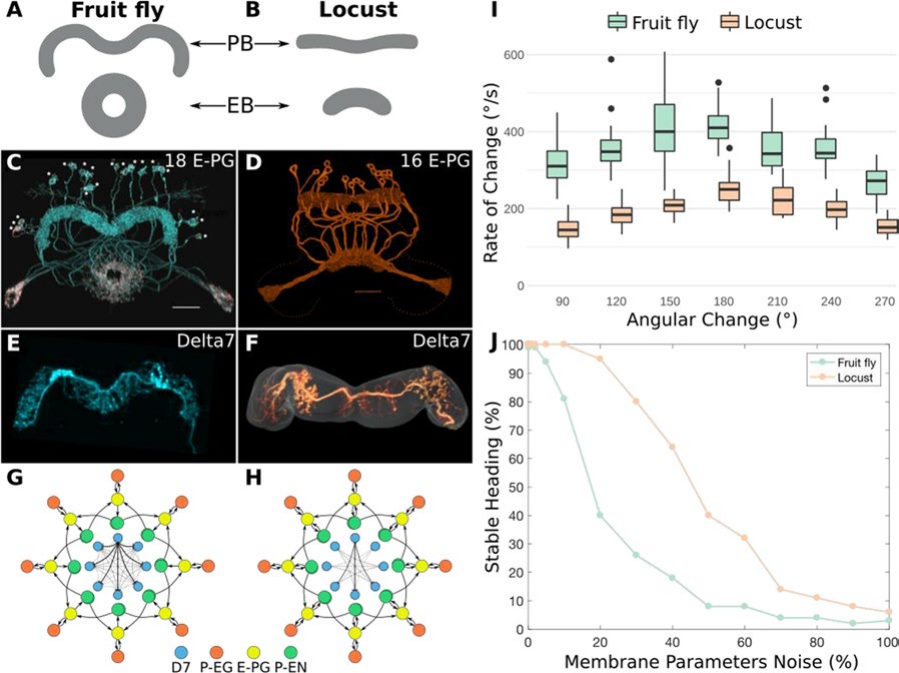
**A-F** Schematics and example neuron anatomy of the fruit fly and the desert locust (Reproduced with permission from [3,4,5]). **G, H** Effective circuit in the fruit fly and the locust, respectively. **I** Maximum rate of heading change sustained by each model for different magnitudes of heading change. **J** Ring attractor stability as function of noise in the cell membrane parameters

**References**

1. Neuser K, Triphan T, Mronz M, Poeck B, Strauss R. Analysis of a spatial orientation memory in Drosophila. Nature. 2008; 453(7199): 1244–1247.
2. Pfeiffer K, Homberg U. Organization and functional roles of the central complex in the insect brain. Annual Review of Entomology. 2013; 59(1):165–184.
3. Wolff T, Rubin G M. Neuroarchitecture of the Drosophila central complex: a catalog of nodulus and asymmetrical body neurons and a revision of the protocerebral bridge catalog. Journal of Comparative Neurology. 2018; 526(16): 2585–2611.
4. Vitzthum H, Homberg U. Immunocytochemical demonstration of locustatachykinin-related peptides in the central complex of the locust brain. Journal of Comparative Neurology. 1998; 390(4): 455–469.
5. Beetz MJ, el Jundi B, Heinze S, Homberg U. Topographic organization and possible function of the posterior optic tubercles in the brain of the desert locust Schistocerca gregaria. Journal of Comparative Neurology. 2015; 523(11): 1589–1607.
6. Pisokas I, Heinze S, Webb B. The head direction circuit of two insect species. bioRxiv 2019.

## O1 Dopamine role in learning and action inference

### Rafal Bogacz

#### University of Oxford, MRC Brain Network Dynamics Unit, Oxford, United Kingdom

##### **Correspondence:** Rafal Bogacz (rafal.bogacz@ndcn.ox.ac.uk)

*BMC Neuroscience* 2020, **21(Suppl 1)**: O1

Much evidence suggests that some dopaminergic neurons respond to unexpected rewards, and computational models have suggested that these neurons encode reward prediction error, which drives learning about rewards. However, these models do not explain recently observed diversity of dopaminergic responses, and dopamine function in action planning, evident from movement difficulties in Parkinson’s disease. The presented work aims at extending existing models to account for these data. It proposes that a more complete description of dopaminergic activity can be achieved by combining reinforcement learning with elements of other recently proposed theories including active inference.

The presented model describes how the basal ganglia network infers actions required to obtained reward using Bayesian inference. The model assumes that a likelihood of reward given action in encoded by the goal-directed system, while the prior probability of making a particular action in a given context is provided by the habit system. It is shown how the inference of the optimal action can be achieved through minimization of free-energy, and how this inference can be implemented in a network with an architecture bearing a striking resemblance to the known anatomy of the striato-dopaminergic circuit. In particular, this network includes nodes encoding prediction errors, which are connected with other nodes in the network in a way resembling the “ascending spiral” structure of dopaminergic connections.

In the proposed model, dopaminergic neurons projecting to different parts of the striatum encode errors in predictions made by the corresponding systems within the basal ganglia. These prediction errors are equal to differences between rewards and expectations in the goal-directed system, and to differences between the chosen and habitual actions in the habit system. The prediction errors enable learning about rewards resulting from actions and habit formation. During action planning, the expectation of reward in the goal-directed system arises from formulating a plan to obtain that reward. Thus dopaminergic neurons in this system provide feedback on whether the current motor plan is sufficient to obtain the available reward, and they facilitate action planning until a suitable plan is found. Presented models account for dopaminergic responses during movements, effects of dopamine depletion on behaviour, and make several experimental predictions.

## O2 Neural manifold models for characterising brain circuit dynamics in neurodegenerative disease

### Seigfred Prado^1^, Simon R. Schultz^2^, Mary A. Go^1^

#### ^1^Imperial College London, Department of Bioengineering, London, United Kingdom; ^2^Imperial College London, London, United Kingdom

##### **Correspondence:** Seigfred Prado (s.prado17@imperial.ac.uk)

*BMC Neuroscience* 2020, **21(Suppl 1)**: O2

Although much is known about neural circuits and molecular pathways required for normal hippocampal functions, the processes by which neurodegenerative diseases, such as Alzheimer’s Disease (AD), disable the functioning of the hippocampus and connected structures remain to be determined. In order to make substantial advances in the treatment of such diseases, we must improve our understanding of how neural circuits process information and how they are disrupted during the progression of these diseases. Recent advances in optical imaging technologies that allow simultaneous recording of large populations of neurons in deeper structures [1] have shown great promise for revealing circuit dynamics during memory tasks [2]. However, to date, no study has revealed how large numbers of neurons in hippocampal-cortical circuits act together to encode, store and retrieve memories in animal models of AD. In this work, we explored the use of neural manifold analysis techniques to characterising brain circuit dynamics in neurodegenerative disease. To understand more precisely the basis of memory and cognitive impairments in AD, we extracted the underlying neural manifolds in large-scale neural responses of hippocampal circuits involved in spatial cognition of behaving mice. For validation, we simulated a model that generates a set of data that mimics the neural activity of hippocampal cells of mouse models running on a linear circular track, while taking into account the effects of amyloid-beta plaques on circuit dynamics [3]. We compare our model with real data obtained by multiphoton imaging of hippocampal CA1 cells in mice engaged in a spatial memory task. We used recurrence analysis to show how neural manifolds evolve over time during memory encoding, storage and recall processes in a repetitive memory task. This work will help with understanding how amyloid-beta proteins affect the neural manifolds for spatial memory, which is particularly disturbed during AD.

**Fig. 1:**
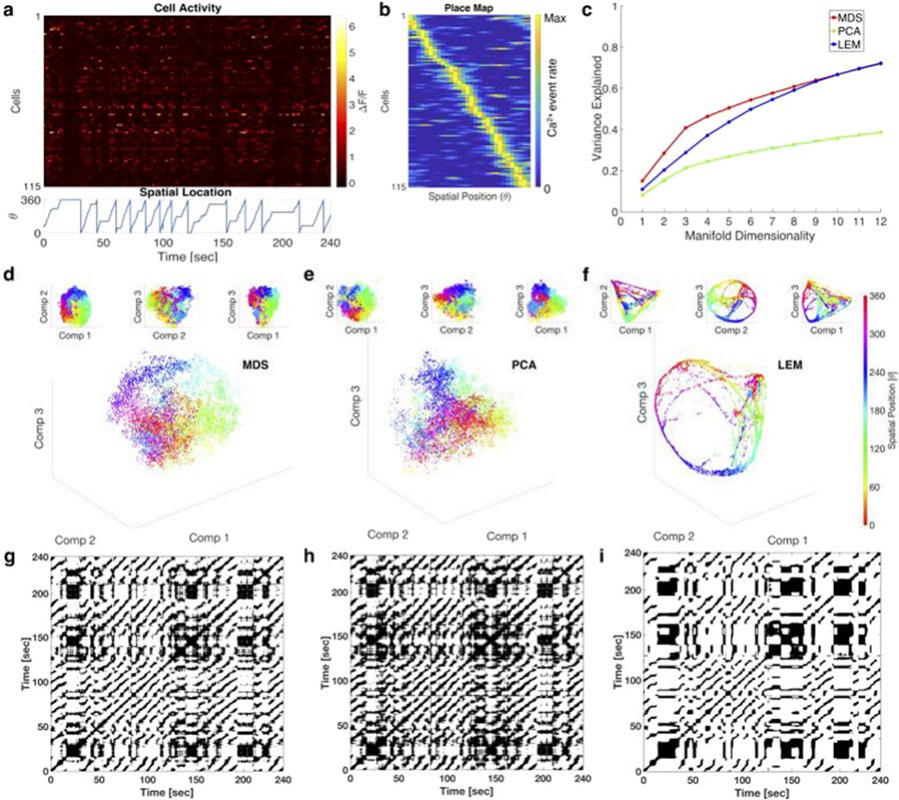
Analyses for a mouse during a 4-minute recording session. **a** Cell activity and mouse position along the linear circular track. **b** Place map showing neuronal tuning curves. **c** Cumulative fraction of variance explained by manifolds of increasing dimensionality. **d-f** The distribution of data points in the reduced dimensional space using MDS, PCA and LEM, respectively. **g-i** Recurrence plots of d-f

**References**

1. Schultz SR, Copeland CS, Foust AJ, Quicke P, Schuck R. Advances in two-photon scanning and scanless microscopy technologies for functional neural circuit imaging. Proceedings of the IEEE. 2016; 105(1): 139-157.
2. Low RJ, Lewallen S, Aronov D, Nevers R, Tank DW. Probing variability in a cognitive map using manifold inference from neural dynamics. bioRxiv. 2018; 418939.
3. Busche MA, et al. Clusters of hyperactive neurons near amyloid plaques in a mouse model of Alzheimer’s disease. Science. 2008; 321(5896): 1686-1689.

## O3 Coupled experimental and modeling representation of the mechanisms of epileptic discharges in rat brain slices

### Anton Chizhov^1^, Dmytry Amakhin^2^, Elena Smirnova^2^, Aleksey Zaitsev^2^

#### ^1^Ioffe Institute, Saint-Petersburg, Russia; ^2^Sechenov Institute of Evolutionary Physiology and Biochemistry of the Russian Academy of Sciences, Laboratory of Molecular Mechanisms of Neural Interactions, Saint Petersburg, Russia

##### **Correspondence:** Anton Chizhov (anton.chizhov@mail.ioffe.ru)

*BMC Neuroscience* 2020, **21(Suppl 1)**:O3

Epileptic seizures and interictal discharges (IIDs) are determined by neuronal interactions and ionic dynamics and thus help to reveal valuable knowledge about the mechanisms of brain functioning in not only pathological but also normal state. As synchronized pathological discharges are much simpler to study than normal functioning, we were able to accomplish their description with a set of electrophysiological evidences constrained by a biophysical mathematical model. In the combined hippocampal-entorhynal cortex slices of rat in high potassium, low magnesium and 4-AP containing solution we evaluated separate AMPA, NMDA and GABA-A conductances for different types of IIDs, using an original experimental technique [1]. The conductances have shown that the first type of the discharges (IID1) is determined by activity of only GABA-A channels due to their pathologically depolarized reversal potential. The second type (IID2) is determined by an early GABA-A followed by AMPA and NMDA components. The third type is pure glutamatergic discharges observed in case of disinhibition. Our mathematical model of interacting neuronal populations reproduces the recorded synaptic currents and conductances for IIDs of the three types [2,3], confirming the major role of interneuron synchronization for IID1 and IID2, and revealing that the duration of IIDs is determined mainly by synaptic depression. IIDs occur spontaneously and propagate as waves with a speed of about a few tens of mm/s [4]. IDs are clusters of IID-like discharges and are determined by the ionic dynamics [5]. To reveal only major processes, main ions and variables, we have formulated a reduced mathematical model “Epileptor-2”, which is a minimal model that reproduces both IDs and IIDs [6] (Fig. 1). It shows that IIDs are spontaneous bursts that are governed by the membrane depolarization and synaptic resource, whereas IDs represent bursts of bursts. Important is the role of the Na/K-ATPhase. Potassium accumulation governs the onset of each ID. The sodium accumulates during the ID and activates the sodium-potassium pump, which terminates the ID by restoring the potassium gradient and thus repolarizing the neurons. A spatially-distributed version of the Epileptor-2 model reveals that it is not extracellular potassium diffusion but synaptic connectivity determines the speed of the ictal wavefront [7], which is consistent with our optogenetic experiments. The revealed factors are to be potential targets for antiepileptic medical treatment.

**Fig. 1:**
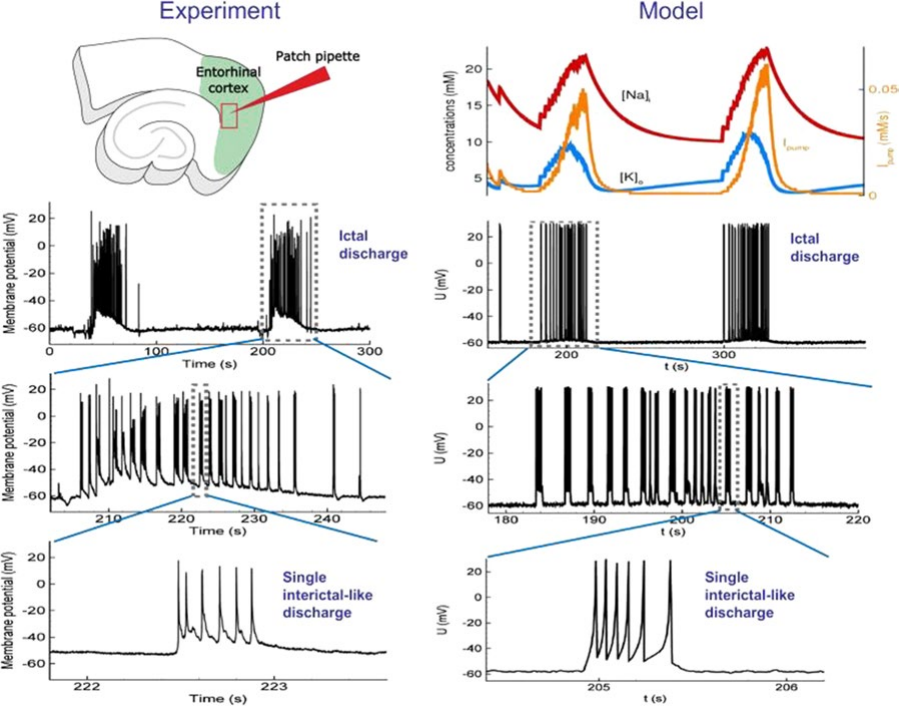
Minimal model Epileptor-2 reproduces ictal events observed in slices. Intracellular sodium and extracellular potassium concentrations and membrane potential of a representative neuron are shown

**Acknowledgments:** This work was supported by the Russian Science Foundation (project 16-15-10201).

**References**

1. Amakhin DV, Ergina JL, Chizhov AV, Zaitsev AV. Synaptic conductances during interictal discharges in pyramidal neurons of rat entorhinal cortex. Frontiers in Cellular Neuroscience. 2016; 10: 233.
2. Chizhov A, Amakhin D, Zaitsev A. Computational model of interictal discharges triggered by interneurons. PLoS ONE. 2017; 12(10): e0185752.
3. Chizhov AV, Amakhin DV, Zaizev AV, Magazanik LG. AMPAR-mediated Interictal Discharges in Neurons of Entorhinal Cortex: Experiment and Model. Doklady Biological Sciences. 2018; 479(1): 47-50.
4. Chizhov AV, Amakhin DV, Zaitsev AV. Spatial propagation of interictal discharges along the cortex. Biochemical and Biophysical Research Communications. 2019; 508(4): 1245-1251.
5. Chizhov AV, Amakhin DV, Zaitsev AV. Mathematical model of Na-K-Cl homeostasis in ictal and interictal discharges. PLoS ONE. 2019; 14(3): e0213904.
6. Chizhov AV, Zefirov AV, Amakhin DV, Smirnova EY, Zaitsev AV. Minimal model of interictal and ictal discharges “Epileptor-2”. PLoS Computational Biology. 2018; 14(5): e1006186.
7. Chizhov AV, Sanin AE. A simple model of epileptic seizure propagation: Potassium diffusion versus axo-dendritic spread. PLoS ONE. 2020; 15(4): e0230787.

## O4 Towards multipurpose bio-realistic models of cortical circuits

### Anton Arkhipov, Yazan N Billeh, Binghuang Cai, Sergey L Gratiy, Kael Dai, Ramakrishnan Iyer, Nathan W Gouwens, Reza Abbasi-Asl, Xiaoxuan Jia, Joshua H Siegle, Shawn R Olsen, Stefan Mihalas, Christof Koch

#### Allen Institute for Brain Science, Seattle, Washington State, United States of America

##### **Correspondence:** Anton Arkhipov (antona@alleninstitute.org)

*BMC Neuroscience* 2020, **21(Suppl 1)**: O4

One of the central questions in neuroscience is how structure of brain circuits determines their activity and function. To explore such structure-function relations systematically, we integrate information from large-scale experimental surveys into data-driven, bio-realistic models of brain circuits, with the current focus on the mouse cortex.

Our 230,000-neuron models of the mouse cortical area V1 [1] were constructed at two levels of granularity—using either biophysically-detailed or point-neurons. These models systematically integrated a broad array of experimental data [1-3]: the information about distribution and morpho-electric properties of different neuron types in V1; connection probabilities, synaptic weights, axonal delays, and dendritic targeting rules inferred from a thorough survey of the literature; and a sophisticated representation of visual inputs into V1 from the Lateral Geniculate Nucleus, fit to *in vivo* recordings. The model activity has been tested against large-scale *in vivo* recordings of neural activity [4]. We found a good agreement between these experimental data and the V1 models for a variety of metrics, such as direction selectivity, as well as less good agreement for other metrics, suggesting avenues for future improvements. In the process of building and testing models, we also made predictions about the logic of recurrent connectivity with respect to functional properties of the neurons, some of which have been verified experimentally [1].

In this presentation, we will focus on the model’s successes in quantitative matching of multiple experimental measures, as well as failures in matching other metrics. Both successes and failures shed light on the potential structure-function relations in cortical circuits, leading to experimentally testable hypotheses. Our models are shared freely with the community: https://portal.brain-map.org/explore/models/mv1-all-layers. We also freely share our software tools – the Brain Modeling ToolKit (BMTK; alleninstitute.github.io/bmtk/), which is a software suite for model building/simulation [5], and the SONATA [6] file format (github.com/allenInstitute/sonata).

**References**

1. Billeh YN, et al. Systematic integration of structural and functional data into multi-scale models of mouse primary visual cortex. Neuron. 2020.
2. Gouwens NW, et al. Classification of electrophysiological and morphological neuron types in the mouse visual cortex. Nature neuroscience. 2019; 22(7): 1182-1195.
3. Gouwens NW, et al. Systematic generation of biophysically detailed models for diverse cortical neuron types. Nature communications. 2018; 9(1): 1-13.
4. Siegle JH, et al. A survey of spiking activity reveals a functional hierarchy of mouse corticothalamic visual areas. bioRxiv. 2019; 805010 [preprint].
5. Gratiy SL, et al. BioNet: A Python interface to NEURON for modelling large-scale networks. PLoS One. 2019; 13(8): e0201630.
6. Dai K, et al. The SONATA data format for efficient description of large-scale network models. PLoS Computational Biology. 2020; 16(2): e1007696.

## O5 How stimulus statistics affect the receptive fields of cells in primary visual cortex

### Ali Almasi^1^, Hamish Meffin^2^, Shi Sun^3^, Michael R Ibbotson^4^

#### ^1^National Vision Research Institute, Melbourne, Australia; ^2^University of Melbourne, Biomedical Engineering, Melbourne, Australia; ^3^University of Melbourne, Optometry and Vision Sciences, Melbourne, Australia; ^4^Australian College of Optometry, The National Vision Research Institute, Carlton, Australia

##### **Correspondence:** Ali Almasi (aalmasi@aco.org.au)

*BMC Neuroscience* 2020, **21(Suppl 1)**: O5

Our understanding of sensory coding in the visual system is largely derived from parametrizing neuronal responses to basic stimuli. Recently, mathematical tools have developed that allow estimating the parameters of a receptive field (RF) model, which are typically a cascade of linear filters on the stimulus, followed by static nonlinearities that map the output of the filters to the neuronal spike rates. However, how much do these characterizations depend on the choice of the stimulus type?

We studied the changes that neuronal RF models undergo due to the change in the statistics of the visual stimulus. We applied the nonlinear input model (NIM) [1] to the recordings of single units in cat primary visual cortex (V1) in response to white Gaussian noise (WGN) and natural scenes (NS). These two stimulus types were matched in their global RMS contrast; however, they are fundamentally different in terms of second- and higher-order statistics, which are abundant in natural scenes but do not exist in white noise. We estimated for each cell the spatial filters constituting the neuronal RF and their corresponding nonlinear pooling mechanism, while making minimal assumptions about the underlying neuronal processing.

We found that cells respond differently to these two stimulus types, with mostly higher spike rates and shorter response latencies to NS than to WGN. The most striking finding was that NS stimuli resulted in around twice as many uncovered RF filters compared to using WGN stimuli. Via careful analysis of the data, we discovered that this difference between the number of identified RF filters is not related to the higher spike rates of cells to NS stimuli. Instead, we found it to be attributed to the difference in the contrast levels of specific features that exhibit different prevalence in NS versus WGN. These features correspond to the V1 RF filters recovered in the model. This specific feature-contrast attains much higher values in NS compared to WGN stimuli. When the feature-contrast is controlled for, it explains the differences in the number of RF filters obtained. Our findings imply that a greater extent of nonlinear processing in V1 neurons can be uncovered using natural scene stimulation.

We also compared the identified RF filters under the two stimulation regimes in terms of their spatial characteristics. Population analysis of the RF filters revealed a statistically significant bias towards higher spatial frequency filters with narrower spatial frequency bandwidth under the NS stimulation regime (p-value < 0.0025).

**Acknowledgements**: The authors acknowledge the support the Australian Research Council Centre of Excellence for Integrative Brain function (CE140100007), the National Health and Medical Research Council (GNT1106390), and Lions Club of Victoria.

**References**

1. McFarland JM, Cui Y, Butts DA. Inferring nonlinear neuronal computation based on physiologically plausible inputs. PLoS Computational Biology. 2013; 9(7).

## O6 Analysis and modelling of response features of accessory olfactory bulb neurons

### Yoram Ben-Shaul^1^, Rohini Bansal^1^, Romana Stopkova^2^, Maximilian Nagel^3^, Pavel Stopka^2^, Marc Spehr^3^

#### ^1^The Hebrew University, Medical Neurobiology, Jerusalem, Israel; ^2^Charles University Prague, Zoology, Prague, Czechia; ^3^RWTH Aachen University, Chemosensation, Aachen, Germany

##### **Correspondence:** Yoram Ben-Shaul (yoramb@ekmd.huji.ac.il)

*BMC Neuroscience* 2020, **21(Suppl 1)**:O6

The broad goal of this work is to understand how consistency on a macroscopic scale can be achieved despite random connectivity at the level of individual neurons.

A central aspect of any sensory system is the manner by which features of the external world are represented by neurons at various processing stages. Yet, it is not always clear what these features are, how they are represented, and how they emerge mechanistically. Here, we investigate this issue in the context of the vomeronasal system (VNS), a vertebrate chemosensory system specialized for processing of cues from other organisms. We focus on the accessory olfactory bulb AOB, which receives all vomeronasal sensory neuron inputs. Unlike the main olfactory system, where MTCs sample information from a single receptor type, AOB MTCs sample information from a variable number of glomeruli, in a manner that seems largely random. This apparently random connectivity is puzzling given the presumed role of this system in processing cues with innately relevant significance.

We use multisite extracellular recordings to measure the responses of mouse AOB MTCs to controlled presentation of natural urine stimuli from male and female mice from various strains, including from wild mice. Crucially, we also measured the levels of both volatile and peptide chemical components in the very same stimulus samples that were presented to the mice. As subjects, we used two genetically distinct mouse strains, allowing us to test if macroscopic similarity can emerge despite variability at the level of receptor expression.

First, we then explored neuronal receptive fields, and found that neurons selective for specific strains (regardless of sex), or a specific sex (regardless of strain), are less common than expected by chance. This is consistent with our previous findings indicating that high level stimulus features are represented in a distributed manner in the AOB. We then compared various aspects of neuronal responses across the two strains, and found a high degree of correlation among them, suggesting that despite apparent randomness and strain specific genetic aspects, consistent features emerge at the level of the AOB.

Next, we set out to model the responses of AOB neurons. Briefly, AOB responses to a given stimulus are modelled as dot products of random tuning profiles to specific chemicals and the actual level of those chemicals in the stimulus. In this manner we derive a population of AOB responses, which we can then compare to the measured responses. Our analysis thus far reveals several important insights. First, neuronal response properties are best accounted for by sampling of protein/peptide components, but not by volatile urinary components. This is consistent with the known physiology of the VNS. Second, several response features (population level neuronal distances, sparseness, distribution of receptive field types) are best reproduced in the model with random sampling of multiple, rather than single molecules per neuron. This suggests that the sampling mode of AOB neurons may mitigate some of the consequences of random sampling. Finally, we note that random sampling of molecules provides a reasonable fit for some, but not all metrics of the observed responses. Our ongoing work aims to identify which changes must be made to our initial simplistic model in order to account for these features.

**Acknowledgement:** This work is funded by GIF and DFG grants to Marc Spehr and Yoram Ben-Shaul.

## O7 ‘Awake delta’ and theta-rhythmic modes of hippocampal network activity track intermittent locomotor behaviors in rats

### Nathan Schultheiss^1^, Tomas Guilarte^2^, Tim Allen^1^

#### ^1^Florida International University, Psychology, Miami, United States of America; ^2^Florida International University, Robert Stempel College of Public Health and Social Work & Environmental Health Sciences, Miami, United States of America

##### **Correspondence:** Nathan Schultheiss (nschulth@fiu.edu)

*BMC Neuroscience* 2020, **21(Suppl 1)**:O7

Delta-frequency activity in the local field potential (LFP) is widely believed to correspond to so-called ‘cortical silence’ during phases of non-REM sleep, but delta in awake behaving animals is not well understood and is rarely studied in detail. By integrating novel analyses of the hippocampal (HC) LFP with simultaneous behavioral tracking, we show for the first time that HC synchronization in the delta frequency band (1-4 Hz) is related to animals’ locomotor behaviors during free exploration and foraging in an open field environment. In contrast to well-established relationships between animals’ running speeds and the theta rhythm (6-10 Hz), we found that delta was most prominent when animals were stationary or moving slowly (i.e. when theta and fast gamma (65-120 Hz) were weak). Furthermore, delta synchronization often developed rapidly when animals paused briefly between intermittent running bouts.

Next, we developed an innovative strategy for identifying putative *modes* of network function based on the spectral content of the LFP. By applying hierarchical clustering algorithms to time-windowed power spectra throughout behavioral sessions (i.e. the spectrogram), we categorized moment-by-moment estimations of the power spectral density (PSD) into spectral modes of HC activity. That is, we operationalized putative *functional modes* of network computation as *spectral modes* of LFP activity. Delta and theta power were strikingly orthogonal across the resultant spectral modes, suggesting the possibility that delta- and theta-dominated hippocampal activity patterns represent distinct modes of HC function during navigation. Delta and theta were also remarkably orthogonal across precisely-defined bouts of running and stationary behavior, indicating that the stops-and-starts that compose rats’ locomotor trajectories are accompanied by alternating delta- and theta-dominated HC states.

We then asked whether the incidence of delta and theta modes was related to the coherence between recording sites in hippocampus or between hippocampus and medial prefrontal cortex (mPFC). We found that intrahippocampal coherences in both the delta-band and the theta-band were monotonically related to theta-delta ratios across modes. Furthermore, in two rats implanted with dual-site recording arrays, we found that theta coherence between HC and mPFC increased during running, and delta-band coherence between mPFC and HC increased during stationary bouts. Taken together, our findings suggest that delta-dominated network modes (and corresponding mPFC-HC couplings) represent functionally-distinct circuit dynamics that are temporally and behaviorally interspersed among theta-dominated modes during spatial navigation. As such, delta modes could play a fundamental role in coordinating mnemonic functions including encoding and retrieval mechanisms, or decision-making processes incorporating prospective or retrospective representations of experience, at a timescale that segments event sequences within behavioral episodes.

## O8 Finite element simulation of ionic electrodiffusion in cellular geometries

### Ada J Ellingsrud

#### Simula Research Laboratory, Scientific Computing and Numerical Analysis, Oslo, Norway

##### **Correspondence:** Ada J Ellingsrud (ada@simula.no)

*BMC Neuroscience* 2020, **21(Suppl 1)**:O8

Electrical conduction in brain tissue is commonly modeled using classical bidomain models. These models fundamentally assume that the discrete nature of brain tissue can be represented by homogenized equations where the extracellular space, the cell membrane, and the intracellular spare are continuous and exist everywhere. Consequently, they do not allow simulations highlighting the effect of a nonuniform distribution of ion channels along the cell membrane or the complex morphology of the cells. In this talk, we present a more accurate framework for cerebral electrodiffusion with an explicit representation of the geometry of the cell, the cell membrane and the extracellular space. To take full advantage of this framework, a numerical solution scheme capable of efficiently handling three-dimensional, complicated geometries is required. We propose a novel numerical solution scheme using a mortar finite element method, allowing for the coupling of variational problems posed over the non-overlapping intra and extracellular domains by weakly enforcing interface conditions on the cell membrane. This solution algorithm flexibly allows for arbitrary geometries and efficient solution of the separate subproblems. Finally, we study ephaptic coupling induced in an unmyelinated axon bundle and demonstrate how the presented framework can give new insights in this setting. Simulations of 9 idealized, tightly packed axons show that inducing action potentials in one or more axons yields ephaptic currents that have a pronounced excitatory effect on neighboring axons, but fail to induce action potentials there [1].

**Acknowledgements:** This project has received funding from the European Research Council (ERC) under the European Union’s Horizon 2020 research and innovation programme under grant agreement 714892 (Waterscales), and from the Research Council of Norway (BIOTEK2021 Digital Life project ‘DigiBrain’, project 248828).

**References**

1. Ellingsrud AJ, Solbrå A, Einevoll GT, Halnes G, Rognes ME. Finite element simulation of ionic electrodiffusion in cellular geometries. Frontiers in Neuroinformatics. 2020; 14: 11.

## O9 Discovering synaptic mechanisms underlying the propagation of cortical activity: a model-driven experimental and data analysis approach

### Heidi Teppola^1^, Jugoslava Acimovic^1^, Marja-Leena Linne^2^

#### ^1^Tampere University, Faculty of Medicine and Health Technology, Tampere, Finland; ^2^Tampere University, Medicine and Health Sciences, Tampere, Finland

##### **Correspondence:** Ada J Ellingsrud (heidi.teppola@tuni.fi)

*BMC Neuroscience* 2020, **21(Suppl 1)**:O9

Spontaneous, synchronized activity is a well-established feature of cortical networks *in vitro* and *in vivo*. The landmark of this activity is the repetitive emergence of bursts propagating across networks as spatio-temporal patterns. Cortical bursts are governed by excitatory and inhibitory synapses via AMPA, NMDA and GABA_A_ receptors. Although spontaneous activity is a well-known phenomenon in developing networks, its specific underlying mechanisms in health and disease are not fully understood. In order to study the synaptic mechanisms regulating the propagation of cortical activity it is important to combine the experimental wet-lab studies with *in silico* modeling and build detailed, realistic, computational models of cortical network activity. Moreover, experimental studies and analysis of microelectrode array (MEA) data are not typically designed to support computational modeling. We show here how the synaptic AMPA, NMDA and GABA_A_ receptors shape the initiation, propagation and termination of the cortical burst activity in rodent networks *in vitro* and *in silico* and develop model-driven data analysis workflow to support the development of spiking and biophysical network models *in silico* [1].

We created a model-driven data analysis workflow with multiple steps to examine the contributions of synaptic receptors to burst dynamics both *in vitro* and *in silico* neuronal networks (Fig. 1). First, the cortical networks were prepared from the forebrains of the postnatal rats and maintained on MEA plates. Second, network-wide activity was recorded by MEA technique under several pharmacological conditions of receptor antagonists. Third, multivariate data analysis was conducted in a way that supports both neurobiological questions as well as the fitting and validation of computational models to quantitatively produce the experimental results. Fourth, the computational models were simulated with different parameters to test putative mechanisms responsible for network activity.

**Fig. 1:**
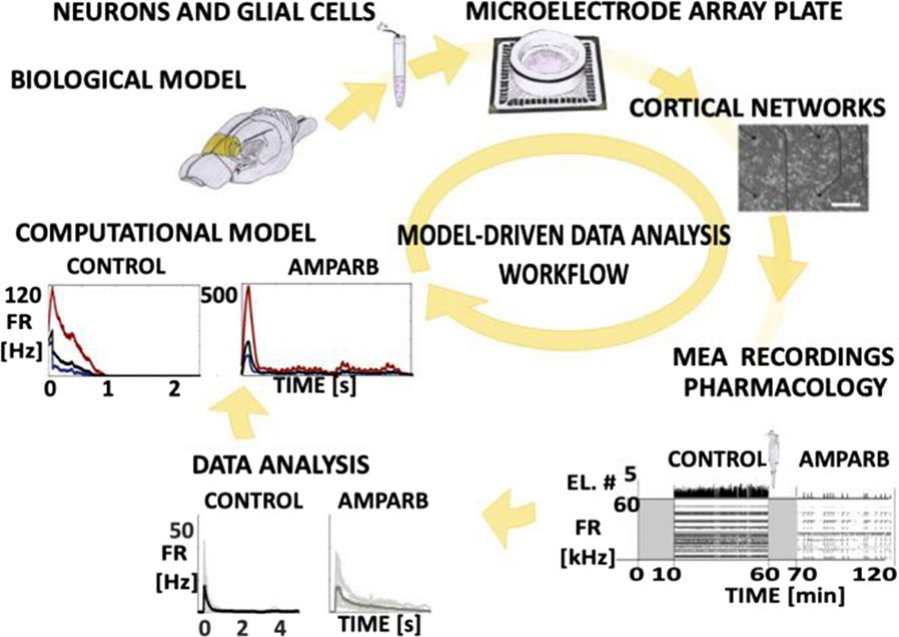
Model-driven data analysis workflow for discovering synaptic mechanisms underlying the propagation of cortical activity in vitro and in silico

The experimental results obtained in this study show that AMPA receptors initiate bursts by rapidly recruiting cells whereas NMDA receptors maintain them. GABA_A_ receptors inhibit the spiking frequency of AMPA receptor-mediated spikes at the onset of bursts and attenuate the NMDA receptor-mediated late phase. These findings highlight the importance of both excitatory and inhibitory synapses in activity propagation and demonstrate a specific interaction between AMPA and GABA_A_ receptors for fast excitation and inhibition. In the presence of this interaction, the spatio-temporal propagation patterns of activity are richer and more diverse than in its absence. Moreover, we emphasize the systematic data analysis approach with model-driven workflow throughout the study for comparison of results obtained from multiple *in vitro* networks and for validation of data-driven model development *in silico*. A well-defined workflow can reduce the amount of biological experiments, promote more reliable and efficient use of the MEA technique, and improve the reproducibility of research. It helps reveal in detail how excitatory and inhibitory synapses shape cortical activity propagation and dynamics in rodent networks *in vitro* and *in silico*.

**References**

1. Teppola H, Aćimović J, Linne M-L. Unique features of network bursts emerge from the complex interplay of excitatory and inhibitory receptors in rat neocortical networks. Frontiers in Cellular Neuroscience. 2019; 13(377): 1-22.

## O10 Neural flows: estimation of wave velocities and identification of singularities in 3D+t brain data

### Paula Sanz-Leon^1^, Leonardo L Gollo^2^, James A Roberts^3^

#### ^1^University of Sydney, QIMR Berghofer, Brisbane, Australia; ^2^QIMR Berghofer/ Monash University, Melbourne, Australia; ^3^QIMR Berghofer, Computational Biology, Brisbane, Australia

##### **Correspondence:** Matthew Botvinick (pmsl.academic@gmail.com)

*BMC Neuroscience* 2020, **21(Suppl 1)**:O10

**Background:** Neural activity organizes in constantly evolving spatiotemporal patterns of activity, also known as brain waves [1]. Indeed, wave-like patterns have been observed across multiple neuroimaging modalities and across multiple spatiotemporal scales [2-4]. However, due to experimental constraints most attention has thus far been given to localised wave dynamics in the range of micrometers to a few centimeters, rather than at the global or large-scale that would encompass the whole brain. Existing toolboxes [2,4] are geared particularly for 2D spatial domains (e.g., LFPs or VSDs on structured rectangular grids). No tool exists to study spatiotemporal waves naturally unfolding in 3D+t as recorded with different non-invasive neuroimaging techniques (e.g, EEG, MEG, and fMRI). In this work, we present results of using our toolbox neural flows (Fig. 1).

**Fig. 1:**
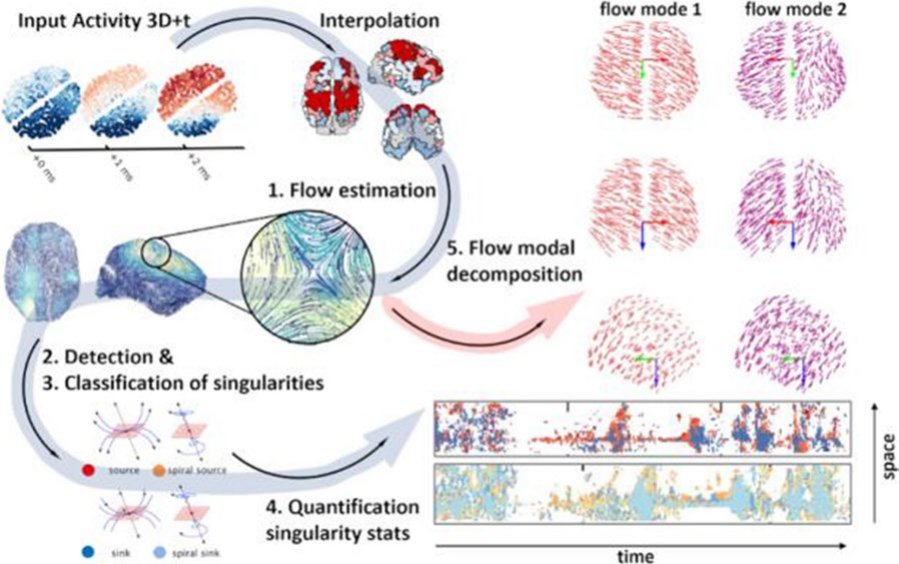
The toolbox has five main capabilities. In the core module: interpolation; **1** estimation of flows; **2** detection of singularities; and, in the analysis module: **3** classification, **4** quantification and tracking of singularities; and, **5** classification of large-scale patterns with modal decomposition

**Methods and Results:** Our toolbox handles irregularly sampled data such as those produced via brain network modelling [5,6] or source-reconstructed M/EEG, and regularly sampled data such as voxel-based fMRI. The toolbox performs the following steps: 1) Estimation of neural flows [5,7]. 2) Detection of 3D singularities (i.e., points of vanishing flow). 3) Classification of 3D singularities. In that regard, the key flow singularities detected so far had been sources and sinks (from where activity emerges and vanishes, respectively), but no methods or tools existed to detect 3D saddles (around which activity is redirected to other parts of the brain). 4) Quantification of singularity statistics. 5) Finally, modal decomposition of neural flow dynamics. This decomposition allows for the detection and prediction of the most common spatiotemporal patterns of activity found in empirical data.

**Conclusions:** Representation of neural activity based on singularities (commonly known as critical points) is essentially a dimensionality reduction framework to understand large-scale brain dynamics. The distribution of singularities in physical space allows us to simplify the complex structure of flows into areas with similar dynamical behavior (e.g., fast versus slow, stagnant, laminar, or rotating). For modelling work, this compact representation allows for an intuitive and systematic understanding of the effects of various parameters in brain network dynamics such as spatial heterogeneity, lesions and noise. For experimental work, neural flows enable a rational understanding of large-scale brain dynamics directly in anatomical space which facilitates the interpretation and comparison of results across multiple modalities. Toolbox capabilities are presented in the accompanying figure. Watch this space for the open-source code: https://github.com/brain-modelling-group.

**References**

1. Roberts JA, et al. Metastable brain waves. Nature Communications. 2019; 10(1): 1-17.
2. Muller L, et al. Rotating waves during human sleep spindles organize global patterns of activity that repeat precisely through the night. eLife. 2016; 5: e17267.
3. Contreras D, Destexhe A, Sejnowski TJ, Steriade M. Spatiotemporal patterns of spindle oscillations in cortex and thalamus. Journal of Neuroscience. 1997; 17: 1179-1196.
4. Destexhe A, Contreras D, Steriade M. Spatiotemporal analysis of local field potentials and unit discharges in cat cerebral cortex during natural wake and sleep states. Journal of Neuroscience. 1999; 19(11), 4595-4608.
5. Sanz-Leon, et al. Neuroimage toolbox 2020 *[in prep].*
6. Breakspear M. Dynamic models of large-scale brain activity. Nature Neuroscience. 2018; 20(3): 340-352.
7. Townsend RG, Gong P. Detection and analysis of spatiotemporal patterns in brain activity. PLoS computational biology. 2018; 14(12): e1006643.

## O11 Experimental and computational characterization of interval variability in the sequential activity of the Lymnaea feeding CPG

### Alicia Garrido-Peña^1^, Irene Elices^1^, Rafael Levi^1^, Francisco B Rodriguez^2^, Pablo Varona^2^

#### ^1^Universidad Autonoma Madrid, Grupo de Neurocomputacion Biologica, Madrid, Spain; ^2^Universidad Autónoma Madrid, Ingeniería Informática, Madrid, Spain

##### **Correspondence:** Alicia Garrido-Peña (alicia.garrido@uam.es)

*BMC Neuroscience* 2020, **21(Suppl 1)**:O11

Central Pattern Generators (CPG) generate and coordinate motor movements by producing rhythms composed of patterned sequences of activations in their constituent neurons. These robust rhythms are yet flexible and the time intervals that build the neural sequences can adapt as a function of the behavioral context. We have recently revealed the presence of robust dynamical invariants in the form of cycle-by-cycle linear relationships between two specific intervals of the crustacean pyloric CPG sequence and the period [1]. Following the same strategy, the present work characterizes the intervals that build the rhythm and the associated sequence of the feeding CPG of the mollusk *Lymnaea Stagnalis*. The study entails both the activity obtained in electrophysiological recordings of living neurons and the rhythm produced by a realistic conductance-based model. The analysis reported here first assesses the quantification of the variability of the intervals and the characterization of relationships between the intervals that build the sequence and the period, which allows the identification of dynamical invariants. To induce variability in the CPG model, we use current injection ramps in individual CPG neurons following the stimulation used in experimental recordings in [2]. Our work extends previous analyses characterizing the *Lymnaea* feeding CPG rhythm from experimental recordings and from modeling studies by considering all intervals that build the sequence [3]. We report the presence of distinct variability in the sequence time intervals and the existence of dynamical invariants, which depend on the neuron being stimulated. The presence of dynamical invariants in CPG sequences, not only in the model but also in two animal species, points out the universality of this phenomena.

**Acknowledgements**: We acknowledge support from AEI/FEDER PGC2018-095895-B-I00 and TIN2017-84452-R.

**References**

1. Elices I, Levi R, Arroyo D, Rodriguez FB, Varona P. Robust dynamical invariants in sequential neural activity. Scientific Reports. 2019; 9(1): 1-13.
2. Elliott CJ, Andrew T. Temporal analysis of snail feeding rhythms: a three-phase relaxation oscillator. Journal of Experimental Biology. 1991; 157(1): 391– 408.
3. Vavoulis DV, et al. Dynamic control of a central pattern generator circuit: a computational model of the snail feeding network. European Journal of Neuroscience. 2007; 25(9): 2805–2818.

## O12 A spatial developmental generative model of human brain structural connectivity

### Stuart Oldham^1^, Ben Fulcher^2^, Kevin Aquino^3^, Aurina Arnatkevičiūtė^3^, Rosita Shishegar^3^, Alex Fornito^3^

#### ^1^Monash University, Clayton, Australia; ^2^The University of Sydney, School of Physics, Sydney, Australia; ^3^Monash University, The Turner Institute for Brain and Mental Health, School of Psychological Sciences and Monash Biomed, Clayton, Australia

##### **Correspondence:** Stuart Oldham (sjold1@student.monash.edu)

*BMC Neuroscience* 2020, **21(Suppl 1)**:O12

The human connectome has a complex topology that is thought to enable adaptive function and behaviour. Yet the mechanisms leading to the emergence of this topology are unknown. Generative models can shed light on this question, by growing networks in silico according to specific wiring rules and comparing properties of model-generated networks to those observed in empirical data [1]. Models involving trade-offs between the metabolic cost and functional value of a connection can reproduce topological features of human brain networks at a statistical level, but are less successful in replicating how certain properties, most notably hubs, are spatially embedded [2,3]. A potential reason for this limited predictive ability is that current models assume a fixed geometry based on the adult brain, ignoring the major changes in shape and size that occur early in development, when connections form.

To address this limitation, we developed a generative model that accounts for developmental changes in brain geometry, informed by structural MRIs obtained from a public database of foetal scans acquired from 21–38 weeks gestational age [4]. We manually segmented the cortical surface of each brain and registered each surface to an adult template surface using Multimodal Surface Matching [5,6]. This procedure allowed us to map nodes to consistent spatial locations through development and measure how distances between nodes (a proxy for connectome wiring cost) change through development. We evaluated the performance of classic trade-off models [2] that either assume a fixed, adult brain geometry (static), or those where cost-value trade-offs dynamically change in accordance with developmental variations in brain shape and size (growth). We used connectomes generated from 100 healthy adults with diffusion MRI to benchmark model performance. Model fit was calculated by comparing model and empirical distributions of topological properties. An optimisation procedure was used to find the optimal parameters and best-fitting models for each individual adult brain network [2]. For fair comparison of model fit across models of varying parametric complexity, we used a leave-one out cross-validation procedure.

Spatial models (*sptl*; which include only distance information) produced poorer fits than those involving distance–topology trade-offs. Homophily models (*matching*, *neighbours*; where connections form between nodes with common neighbours) were among the best fitting. Growth models produced slightly better fits than static models overall. These results still generally held when the cross-validation procedure was employed (Fig. 1a). Neither growth nor static models reproduced the spatial topography of network hubs, but growth models are associated with a less centralized anatomical distribution of hubs across the brain, which is more consistent with the empirical data (Fig. 1b).

**Fig. 1:**
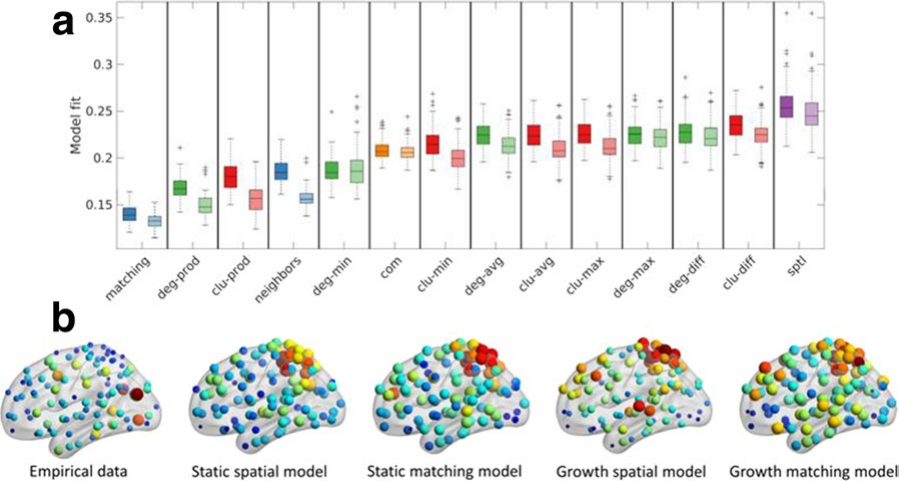
**a** Cross-validation results for static and growth models. Different models are shown on the x-axis and the shading of the boxplot indicates the model type: static models are darker while growth models are lighter. **b** Hub distribution under different models. The size and colour show the degree of each node (blue/small indicates a low degree node, red/large indicates a high degree node)

In summary, we introduce a new framework for examining how developmental changes in brain geometry influence brain connectivity. Our results suggest that while such changes influence network topology, they are insufficient to explain how complex connectivity patterns emerge in brain networks.

**References**

1. Betzel RF, Bassett DS. Generative models for network neuroscience: prospects and promise. Journal of The Royal Society Interface. 2017; 14(136): 20170623.
2. Betzel RF, et al. Generative models of the human connectome. Neuroimage. 2016; 124: 1054-64.
3. Zhang X, et al. Generative network models identify biological mechanisms of altered structural brain connectivity in schizophrenia. bioRxiv. 2019; 604322.
4. Gholipour A, et al. A normative spatiotemporal MRI atlas of the fetal brain for automatic segmentation and analysis of early brain growth. Scientific Reports. 2017; 7: 476.
5. Robinson EC, et al. MSM: a new flexible framework for multimodal surface matching. Neuroimage. 2014; 100: 414-426.
6. Robinson EC, et al. Multimodal surface matching with higher-order smoothness constraints. Neuroimage. 2018; 167: 453-465.

## O13 Cortical integration and segregation explained by harmonic modes of functional connectivity

### Katharina Glomb^1^, Gustavo Deco^2^, Morten L Kringelbach^3^, Patric Hagmann^4^, Joel Pearson^5^, Selen Atasoy^3^

#### ^1^Centre Hospitalier Universitaire Vaudois, Department of Radiology, Lausanne, Switzerland; ^2^Universitat Pompeu Fabra, Barcelona, Spain; ^3^University of Oxford, Department of Psychiatry, Oxford, United Kingdom; ^4^University Hospital of Lausanne and University of Lausanne, Department of Radiology, Lausanne, Switzerland; ^5^University of New South Wales, School of Psychology, Sydney, Australia

##### **Correspondence:** Katharina Glomb (katharina.glomb@upf.edu)

*BMC Neuroscience* 2020, **21(Suppl 1)**:O13

The idea that harmonic modes - basis functions of the Laplace operator - are meaningful building blocks of brain function are gaining attention [1–3]. We extracted harmonic modes from the Human Connectome Project’s (HCP) dense functional connectivity (dFC), an average over 812 participants’ resting state fMRI dFC matrices. In this case, harmonic modes give rise to functional harmonics. Each functional harmonic is a connectivity gradient [4] that is associated with a different spatial frequency, and thus, functional harmonics provide a frequency-ordered, multi-scale, multi-dimensional description of cortical functional organization.

We propose functional harmonics as an underlying principle of integration and segregation. Figure 1a shows 2 functional harmonics on the cortical surface. In harmonic 11 (ψ11), the two functional regions that correspond to the two hands are on opposite ends of the gradient (different colors on the surface) and are thus functionally segregated. In contrast, in harmonic 7 (ψ7), the two areas are on the same end of the gradient, and are thus integrated. This way, functional harmonics explain how two brain regions can be both functionally integrated and segregated, depending on the context.

**Fig. 1:**
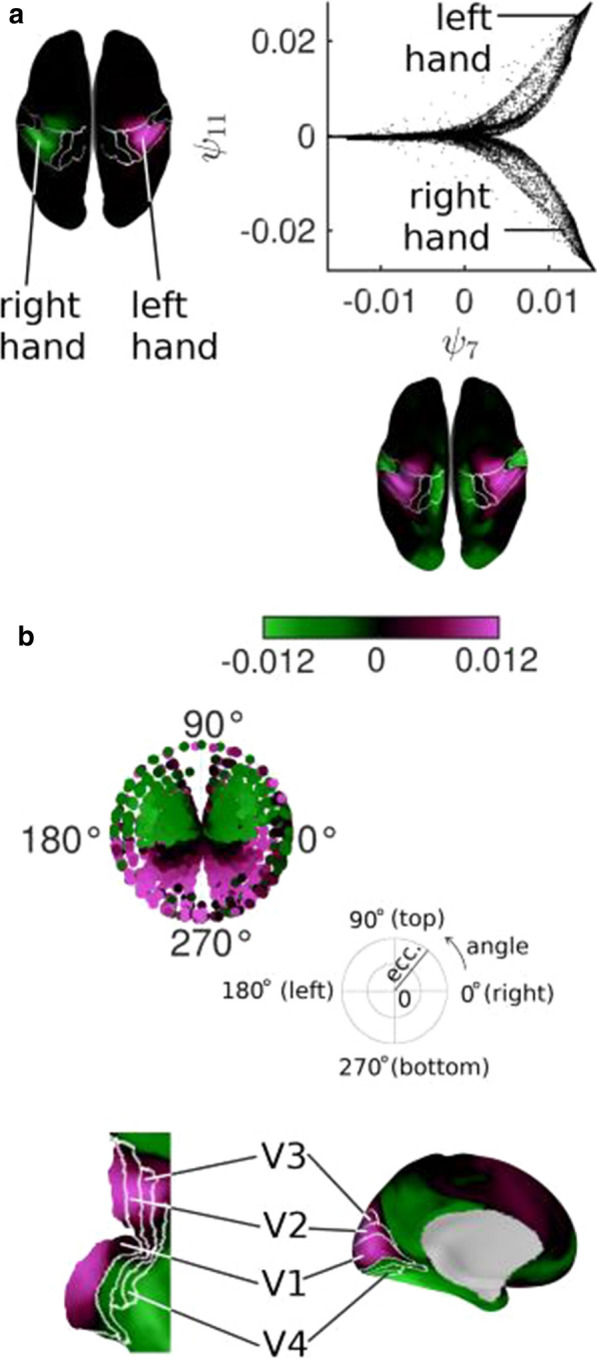
**a** Specialized brain regions emerge from continuous gradients in multiple dimensions. In this example, somatotopic areas arise from functional harmonics 3 and 11. **b** Certain functional harmonics (here, functional harmonic 8) capture retinotopy. Vertices in V1-V4 are plotted in retinotopic space [6] in the same colors as on the flattened surface of the early visual cortex

Figure 1a illustrates how specialized areas emerge from the smooth gradients of functional harmonics: the two hand areas occupy well-separated regions of the space spanned by ψ7 and ψ11. Thus, functional harmonics unify two perspectives, a view where the brain is organized in discrete modules, and one in which function varies gradually [4].

The borders drawn on the cortex correspond to functional areas in the HCP’s multimodal parcellation [5]. In this example, the isolines of the gradients of the functional harmonics follow the borders. We quantified how well, in general, the first 11 functional harmonics follow the borders of cortical areas by comparing the variability of the functional harmonics within and between the areas given by the HCP parcellation; i.e. we computed the silhouette value (SH), averaged over all 360 cortical areas. The SH lies between 0 and 1, where 1 means perfect correspondence between isolines and parcels. We found average SHs between 0.65 (ψ10) and 0.85 (ψ1), indicating a very good correspondence. Thus, functional harmonics capture the “modular perspective” of brain function.

On the other hand, several functional harmonics are found to capture topographic maps and thus, gradually varying function. One important example is retinotopic organization of the visual cortex. Figure 1b shows functional harmonic 8 (ψ8) as an example in which both angular and eccentricity gradients are present [6]. Topographic organization is also found in the somatosensory/motor cortex, known as somatotopy. This is shown in Figure 1a, where several somatotopic body areas are reproduced.

Taken together, our results show that functional specialization, topographic maps, and the multi-scale, multi-dimensional nature of functional networks are captured by functional harmonics, thereby connecting these empirical observations to the general mathematical framework of harmonic eigenmodes.

**References**

1. Atasoy S, Donnelly I, Pearson J. Human brain networks function in connectome-specific harmonic waves. Nature Communications. 2016; 7: 10340.
2. Robinson PA, et al. Eigenmodes of brain activity: Neural field theory predictions and comparison with experiment. Neuroimage. 2016; 142: 79-98.
3. Tewarie P, et al. How do spatially distinct frequency specific MEG networks emerge from one underlying structural connectome? The role of the structural eigenmodes. NeuroImage. 2019; 186: 211-220.
4. Margulies DS, et al. Situating the default-mode network along a principal gradient of macroscale cortical organization. Proceedings of the National Academy of Sciences. 2016; 113(44): 12574-12579.
5. Glasser MF, et al. A multi-modal parcellation of human cerebral cortex. Nature. 2016; 536(7615): 171–178.
6. Benson NC, et al. The Human Connectome Project 7 Tesla retinotopy dataset: Description and population receptive field analysis. Journal of Vision. 2018; 18(13): 23-23.

## O14 Reconciling emergences: an information-theoretic approach to identify causal emergence in multivariate data

### Pedro Mediano^1^, Fernando Rosas^2^, Henrik Jensen^3^, Anil Seth^4^, Adam Barrett^4^, Robin Carhart-Harris^2^, Daniel Bor^1^

#### ^1^University of Cambridge, Department of Psychology, Cambridge, United Kingdom; ^2^Imperial College London, Department of Medicine, London, United Kingdom; ^3^Imperial College London, Department of Mathematics, London, United Kingdom; ^4^University of Sussex, Department of Informatics, Brighton, United Kingdom

##### **Correspondence:** Pedro Mediano (pam83@cam.ac.uk)

*BMC Neuroscience* 2020, **21(Suppl 1)**:O14

The broad concept of emergence is instrumental in various key open scientific questions – yet, few quantitative theories of what constitutes emergent phenomena have been proposed. We introduce a formal theory of causal emergence in multivariate systems, which studies the relationship between the dynamics of parts of a system and macroscopic features of interest. Our theory provides a quantitative definition of downward causation, and introduces a complementary modality of emergent behaviour, which we refer to as causal decoupling. Moreover, we provide criteria that can be efficiently calculated in large systems, making the theory applicable in a range of practical scenarios. We illustrate our framework in a number of case studies, including Conway’s Game of Life and ECoG data from macaques during a reaching task, which suggest that the neural representation of motor behaviour may be causally decoupled from cortical activity.

## P1 Homeostatic recognition circuits emulating network-wide bursting and surprise

### Tsvi Achler

#### Optimizing Mind, Palo Alto, CA, United States of America

##### **Correspondence:** Tsvi Achler (achler@gmail.com)

*BMC Neuroscience* 2020, **21(Suppl 1)**:P1

Understanding the circuits of recognition is essential to build a deeper understanding of virtually all of the brains behaviors and circuits.

The goal of this work is to capture simultaneous findings on both the neural and behavioral levels, namely Network Wide Bursting (NWB) dynamics with surprise (unexpected inputs), using a hypothesized recognition circuit based on the idea of homeostasis flow.

If real neural brains at a resting state are presented with an unexpected or new stimulus, the brain network shows a fast network-wide increase in activation (NWB of many neurons) followed by a slower inhibition, until the network settles again to a resting state. Bursting phenomena during recognition is found ubiquitously in virtually every type of organism, within isolated brain dissections and even neural tissue grown in a dish (Fig. 1). Its source and function remain poorly understood. Behavioral manifestation of surprise can be observed if the input is much unexpected and may involve multiple brain regions.

**Fig. 1:**
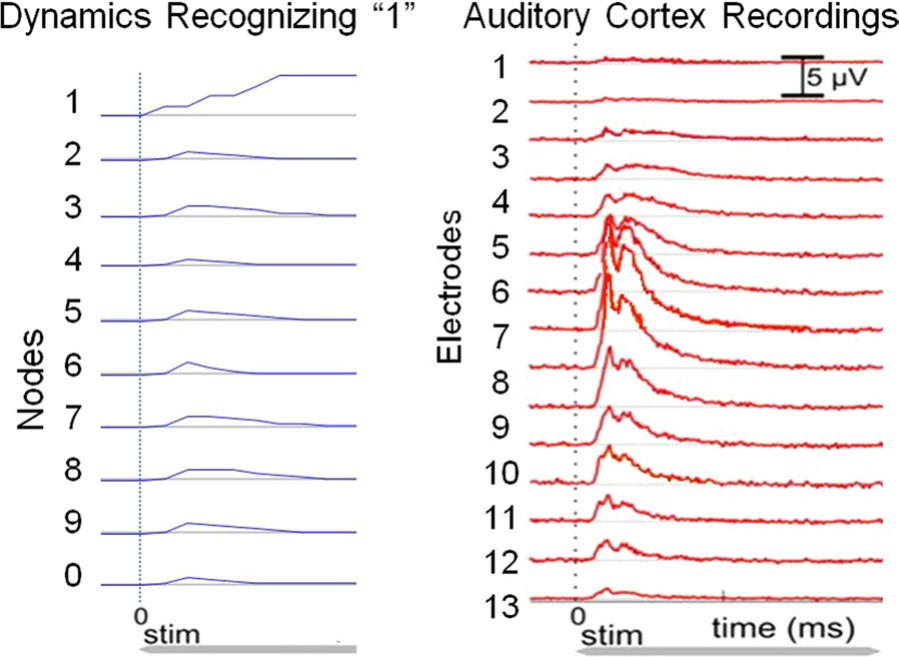
Network-Wide Bursting. Left: A 26 node Homeostatic network trained on MNIST digit recognition data & its response to a numeral digit 1 presented at time 0. The nodes for all digits 0-9 briefly burst but then settle down, leaving only node 1 active. Right: example neuron recordings from audio cortex & surrounding regions in response to sound stimuli (modified from Lakatos et al. 2005)

The homeostatic flow model posits that activation from inputs is balanced with top down pre-synaptic regulatory feedback from output neurons. Information is projected from inputs to outputs with forward connections then back to inputs with backwards homeostatic connections which inhibits the inputs. This effectively acts to balance the inputs & outputs (homeostasis) and generates an internal error-dependent input. This homeostatic input is then projected again to outputs and back again until output values relate recognition. This occurs during recognition and no weights are learned.

When a surprise or unexpected input stimulus is presented, NWB occurs because the homeostatic balance is disturbed with the new stimulus. The system subsequently calms down as it settles back to a new homeostasis.

In comparing to existing models, this circuit is different from Adaptive Resonance Theory because: 1) no lateral connections are required (inhibitory or otherwise) 2) all neurons feed backwards pre-synaptically at the same time 3) there is no vigilance parameter. It is different from Hopfield networks because instead of top-down feedback being positive, it is negative (inhibitory & homeostatic). This changes the functions and dynamics of the model making it stable: its dynamics eventually converge to steady state as long as inputs do not change.

The homeostatic feedback should not be confused with error of learning algorithms since: 1) it is implemented during recognition 2) does not adjust any weights at any time 3) not generated using training data. It is different from generative and predictive coding models because 1) it is primarily used during recognition not learning 2) the generative and recognition components are inseparable and contained within a single integrated homeostatic circuit.

The network is connectionist but approximates a Bayesian network by: 1) homeostatic weights are roughly equivalent to Bayesian likelihood values 2) output values can behave as Bayesian priors if they are maintained externally or if inputs suddenly change. Maintaining priors changes circuit recognition and dynamics without changing weights.

Learning can be achieved with simple Hebbian learning, obtaining weights that are similar to Bayesian likelihood. Both directions of the homeostatic process learn the same weights. Single layer learning is demonstrated with standard MNIST digits while capturing the neural findings of NWB.

## P2 The monosynaptic inference problem: linking statistics and dynamics in ground truth models

### Zach Saccomano^1^, Rodrigo Pena^2^, Sam Mckenzie^3^, Horacio Rotstein^4^, Asohan Amarasingham^5^

#### ^1^City University of New York, Biology, New York, United States of America; ^2^New Jersey Institute of Technology, Federated Department of Biological Sciences, Newark, United States of America; ^3^New York University, Neuroscience Institute, New York, United States of America; ^4^New Jersey Institute of Technology, Federated Department of Biological Sciences, NJIT / Rutgers University, Newark, United States of America; ^5^New Jersey Institute of Technology, Biological Sciences, New York, United States of America

##### **Correspondence:** Zach Saccomano (zsaccomano@ccny.cuny.edu)

*BMC Neuroscience* 2020, **21(Suppl 1)**:P2

Given the ability to record spike trains from populations of neurons, a natural aim in neuroscience is to infer properties of synapses, including connectivity maps, from such recordings. These inferences can derive from observations of strong millisecond-timescale correlations among spike train pairs, as typically reflected by a sharp, short-latency peak in the causal direction of cross-correlograms (CCG) between the reference and target neurons. However, such sharp peaks may also occur when two disconnected neurons systematically fire close together in time in the absence of a direct monosynaptic connection. A further confound is that a monosynapse likely influences the postsynaptic cell on broader timescales as well. These observations motivate a systematic analysis of how a monosynapse exerts influence on the intrinsic dynamics of its postsynaptic target and how this affects the properties of the CCG and the ability to infer the monosynaptic properties. In previous work [1], we adapted a statistical framework for monosynaptic inference based on a (statistical) separation-of-timescale principle, in which monosynaptic interactions are systematically assumed to drive spike-spike correlations at finer timescales than non-monosynaptic interactions. We examined this principle in a simplified ground truth neuron model with minimal intrinsic dynamics, such as the leaky integrate-and-fire (LIF) model with an adaptive threshold. In this work, we extend these ideas to more realistic models and multiple time scales. We use a generalized LIF model with two-dimensional subthreshold dynamics and multiple (dynamic) time scales. The model describes the nonlinear dynamics of the voltage and a slower adaptation gating variable. These subthreshold dynamics also describe the onset of spikes, but not the spiking dynamics. Spikes are added manually. Our previous work exploited our ability to study counterfactual causal inferences in simulations. For example, in simulations with two neurons and comodulated noise, how much would the peak of the CCG change if the monosynapse were deleted? Here we extend this approach to the more complex models where the properties of the CCG are affected by the model nonlinearities and time scales. In this scenario, the model’s slow time scale (captured by the adaptation time constant) affects the CCG time scales (an emergent property of the monosynaptic interaction). Finally, we assess how bias induced by the separation-of-timescale principle (in the statistical sense) depends on the intrinsic dynamics of postsynaptic cells, in particular on the separation of time scales in the dynamic modeling sense.

**References**

1. Platkiewicz J, Saccomano Z, McKenzie S, English D, Amarasingham A. Monosynaptic inference via finely-timed spikes. arXiv. 2019; 1909.08553 *[preprint].*

## P3 Using higher-order networks to analyze non-linear dynamics in neural network models

### Xerxes Arsiwalla

#### Pompeu Fabra University, Barcelona, Spain

##### **Correspondence:** Xerxes Arsiwalla (x.d.arsiwalla@gmail.com)

*BMC Neuroscience* 2020, **21(Suppl 1)**:P3

In linear dynamical systems, one has an elegant way to analyze the system’s dynamics using a network representation of the state transition matrix, obtained from a state space formulation of the system of ODEs. However, in non-linear systems, there is no state space formulation to begin with. In recent work, we have established a correspondence between non-linear dynamical systems and higher-order networks [1] (see also [2]). The latter refer to graphs that include links between nodes and edges, as well as links between two edges. It turns out that such networks have a rich structure capable of representing non-linearities in the vector field of dynamical systems. To do this, one has to first dimensionally unfold a system of non-linear ODEs such that non-linear terms in the vector field can be re-expressed using auxiliary dynamical variables. This results in an unfolded dynamical system with only polynomial non-linearities. This operation works for a large class of non-linear systems. It turns out that once we have a polynomial vector field, the system can then be expressed in generalized state space form. This is what ultimately admits a graphical representation of the system. However, the resulting graph consists of higher-order edges. This generalizes the more common usage of networks with dyadic edges to networks with compounded edges. Here, we show an application of these graphs to analyze neural networks built from sigmoidal rate models as well as mean-field models. Higher-order graphs enable one to systematically decompose contributions of various non-linear terms to the dynamics as well as analyze stability and control of the system using network properties.

**References**

1. Arsiwalla XD. A functorial correspondence between non-linear dynamical systems and higher-order networks. 2020 *[submitted].*
2. Baez JC, Pollard BS. A compositional framework for reaction networks. Reviews in Mathematical Physics. 2017; 29(09): 1750028.

## P4 Self-organization of connectivity in spiking neural networks with balanced excitation and inhibition

### Jihoon Park^1^, Yuji Kawai^2^, Minoru Asada^2^

#### ^1^Osaka University, Suita, Japan; ^2^Osaka University, Institute for Open and Transdisciplinary Research Initiatives, Suita, Japan

##### **Correspondence:** Jihoon Park (jihoon.park@otri.osaka-u.ac.jp)

*BMC Neuroscience* 2020, **21(Suppl 1)**:P4

Atypical neural activity and structural network changes have been detected in the brains of autism spectrum disorder (ASD) [1]. It has been hypothesized that an imbalance in the activity of excitatory and inhibitory neurons causes the pathological changes in autistic brains, denoted by the E/I balance hypothesis [2]. In this study, we investigate the effect of E/I balance on the self-organization of network connectivity and neural activity using a model approach. Our model follows the Izhikevich spiking neuron model [3], and consists of three neuron groups, each composed of 800 excitatory neurons and *N*_I_ inhibitory neurons (Fig. 1A). Each excitatory neuron had 100 intraconnections with randomly selected neurons in the same neuron group, and 42 inter-connections with randomly selected neurons in its neighboring neuron group. These synaptic weights were modified using the Spike-timing-dependent plasticity rule [3]. Each inhibitory neuron had 100 intraconnections with randomly selected excitatory neurons in the same neuron group, but they did not have any interconnections nor plasticity. We simulated the model with different and inhibitory synaptic weights (*W*_I_) in one neuron group (neuron group 1 in Fig. 1A) to change the degree of inhibition in the neuron group. *N*_I_ and *W*_I_ in the other groups (2 and 3 in Fig. 1A) were set to 200 and -5, respectively. The simulation results show greater intraconnections in all neuron groups when *N*_I_ and *W*_I_ were lower values, i.e., the E/I ratio increased compared to those in the typical E/I ratio (Fig. 1B). Moreover, asymmetric interconnections between neuron groups emerged where the synaptic weights from neuron groups 2 to 1 were higher than when the connectivity was in the opposite direction (Fig. 1C), where the E/I ratio was found to increase. Furthermore, the phase coherence between the average potentials of neuron groups was found to be weak with an increased E/I ratio (Fig. 1D). These results indicate that the disruption of the E/I balance, especially the weak inhibitory, induces excessive local connections and asymmetric intergroup connections. Therefore, the synchronization between neuron groups decreases, i.e., there is a weak long-range functional connectivity. These results suggest that the E/I imbalance might cause strong local anatomical connectivity and weak long-range functional connectivity in the brains of ASD [1].

**Fig. 1:**
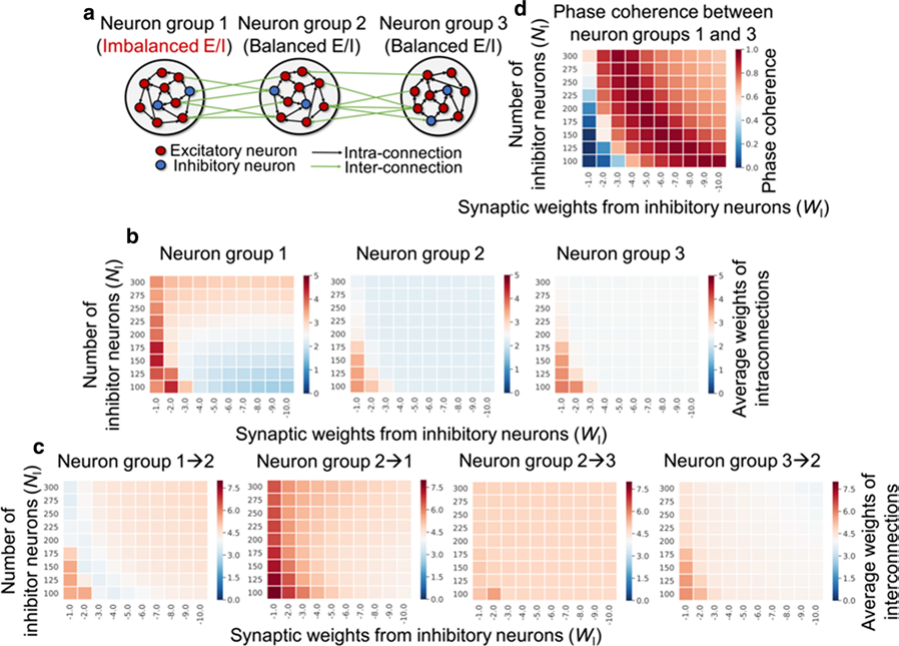
Model overview and results. **A** A model consisting of neuron groups. Neuron group 1 has controlled inhibitory neurons and does not directly connect with neuron group 3. **B** Average weights of intraconnections in each neuron group after self-organization. **C** Average weights of interconnections among neuron groups after self-organization. **D** Phase coherence between neuron groups 1 and 3

**Acknowledgements:** This work was supported by JST CREST Grant Number JPMJCR17A4 including the AIP challenge (conceptualization and resources), and a project commissioned by the New Energy and Industrial Technology Development Organization (NEDO; implementation and data curation).

**References**

1. Belmonte MK, et al. Autism and abnormal development of brain connectivity. Journal of Neuroscience. 2004; 24: 9228–9231.
2. Nelson SB, Valakh V. Excitatory/inhibitory balance and circuit homeostasis in autism spectrum disorders. Neuron. 2015; 87: 684–698.
3. Izhikevich EM. Polychronization: computation with spikes. Neural Computation. 2006; 18: 245–282.

## P5 Mathematical modeling of the circadian clock in spiders

### Daniel Robb^1^, Lu Yang^2^, Nadia Ayoub^2^, Darrell Moore^3^, Thomas Jones^3^, Natalia Toporikova^2^

#### ^1^Roanoke College, Department of Mathematics, Computer Science and Physics, Salem, Virginia, United States of America; ^2^Washington and Lee University, Department of Biology, Lexington, Virginia, United States of America; ^3^East Tennessee State University, Department of Biological Sciences, Johnson City, Tennessee, United States of America

##### **Correspondence:** Daniel Robb (robb@roanoke.edu)

*BMC Neuroscience* 2020, **21(Suppl 1)**:P5

The circadian clock in organisms produces oscillations in neural and physiological functions with an intrinsic period on the order of the 24-hour circadian day. The circadian clock, controlled by the suprachiasmatic nucleus (SCN) in the brain, is entrained by light-dark cycles, so that organisms synchronize these essential oscillations with external conditions. The clock has been studied extensively in flies and yeast, among other species, with intrinsic periods ranging from 22-26 hours [1], and a fairly limited range of entrainment to external light-dark cycles. Interestingly, our previous work has shown that spiders have a significantly wider range of intrinsic periods, from 19-30 hours, and an ability to entrain to a much wider range of applied external light-dark cycles. To identify a potential mechanism for the unusual circadian clock in spiders, we have developed a mathematical model for the spider circadian clock which incorporates negative feedback. We have used the model to investigate two possible mechanisms for the wide range of entrainment in spiders. First, light could be a ‘strong stimulus’ which acts powerfully on a circadian clock of typical strength. Second, the circadian clock in spiders could be ‘weak’, i.e., not as robust to perturbations, relative to that of other species. To distinguish between these two mechanisms, a bifurcation analysis of the model has been performed, as shown in the figure (Fig. 1). Our model makes several testable predictions. In the ‘strong stimulus’ scenario, we predict a faster onset of locomotor activity for spiders with shorter intrinsic circadian periods with a change in the applied light cycle, and a slower onset of locomotor activity for spiders with longer intrinsic periods. In addition, the ‘strong stimulus’ scenario could often lead to a lack of locomotor activity in constant light. In contrast, the ‘weak clock’ scenario predicts a strong dependence of entrainment on the light intensity, and that, once achieved, entrainment would lead to an increase in the amplitude of model oscillations. The balance of our computational modeling and experimental results currently favors the weak clock scenario.

**Fig. 1:**
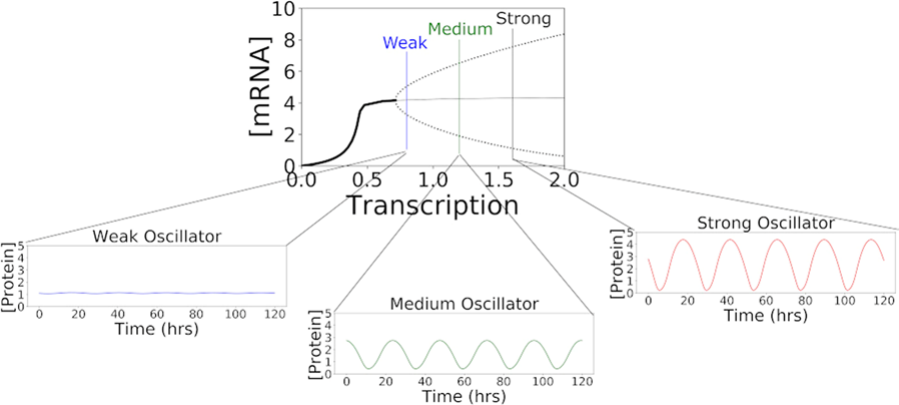
Bifurcation diagram of circadian clock model identifying weak, medium, and strong regimes

**References**

1. Johnson CH, Elliott J, Foster R, Honma K-I, Kronauer R. Fundamental properties of circadian rhythms. In: Chronobiology: Biological Timekeeping. Sinauer. 2004; 67-105.

## P6 Inference of functional connectivity in living neural networks

### Sarah Marzen^1^, Martina Lamberti^2^, Michael Hess^3^, Jacob Mehlman^4^, Denise Hernandez^4^, Joost le Feber^2^

#### ^1^Pitzer, Scripps, and Claremont McKenna College, Physics, Claremont, California, United States of America; ^2^University of Twente, Clinical Neurophysiology, Twente, Netherlands; ^3^Claremont McKenna College, Computational Neuroscience, Claremont, California, United States of America; ^4^Claremont McKenna College, Physics, Claremont, California, United States of America

##### **Correspondence:** Sarah Marzen (smarzen@cmc.edu)

*BMC Neuroscience* 2020, **21(Suppl 1)**:P6

In experiments with stimuli, we often wish to assess changes in connectivity between neurons as the experiment progresses. There are a number of methods for assessing connectivity with a variety of drawbacks, but it is not clear that these methods are connected to one another. Furthermore, it is not clear that these functional connectivities are connected to real synaptic connectivities. We present some evidence that functional connectivities from two disparate methods (Conditional Firing Probability analysis and Maximum Entropy analysis) and synaptic connectivities in one dynamical model (that of leaky integrate-and-fire neurons) are all related.

**Fig. 1:**
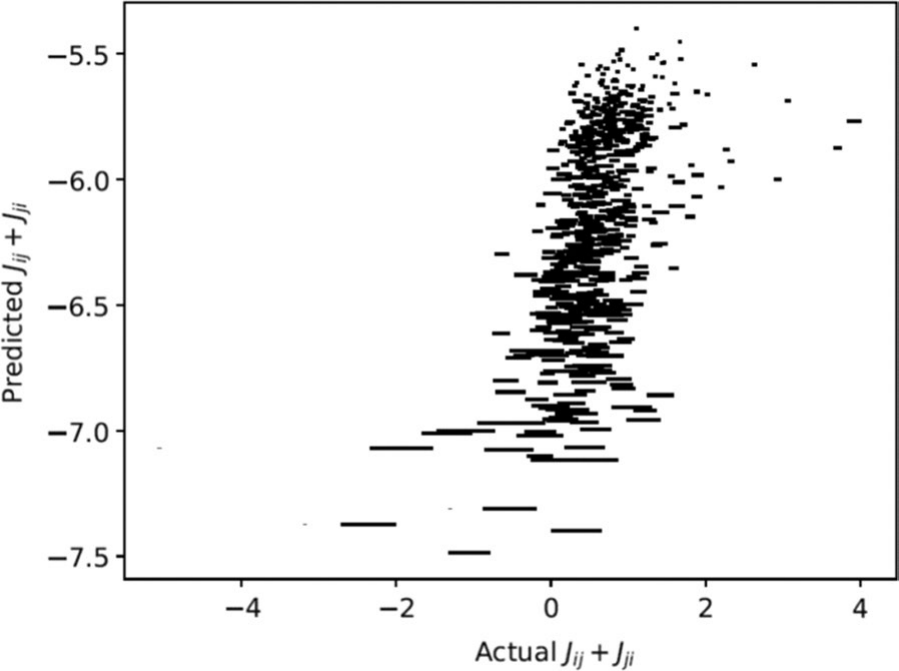
The predicted Ji,j + Jj,I is compared to the true Ji,j + Jj,I for a recording of spontaneous activity from a neuronal culture. Only excitatory connections are included. The analytic expression requires that Δt be sufficiently small, and here Δt is 3 ms—not small enough. Even so, we see a strong relationship between predictions and measurements with R = 0.5

## P7 Functional dissection of prefrontal networks using optogenetics and large-scale multi-electrode recordings

### Eloise V Giraud^1^, Jean-Philippe Thivierge^1^, Michael Lynn^2^, Jean-Claude Beique^2^

#### ^1^University of Ottawa, School of Psychology, Ottawa, Canada; ^2^University of Ottawa, Cellular and Molecular Medicine, Ottawa, Canada

##### **Correspondence:** Eloise V Giraud (egira024@uottawa.ca)

*BMC Neuroscience* 2020, **21(Suppl 1)**:P7

The prefrontal cortex (PFC) plays an important role in executive functions that guide reward-seeking, goal-directed and memory-guided behaviours. However, the contribution of specific cell types to the activity of broader cortical circuits remains largely unknown. This is important to inform accurate computational models of prefrontal cortex function. Here, we used high-density multielectrode arrays containing 4,096 closely spaced electrodes to monitor the spiking activity of PFC neurons in acute slice preparations. We developed spike sorting techniques that combined spline interpolation and principal component analysis to distinguish regular-spiking excitatory neurons from fast-spiking inhibitory interneurons. Our sorting algorithm was validated using a targeted combination of viral and optogenetic strategies. By cell-type-specific optogenetic stimulation, we described how parvalbumin interneurons regulate the interplay between excitation and inhibition. Specifically, we characterized the influence of parvalbumin interneurons on network-wide firing rates and distance-dependent pairwise correlations within the PFC. These results form a key target for computational models that aim to capture the interactions between excitation and inhibition in cortical areas.

## P8 Average beta burst duration profiles provide a signature of dynamical changes between the ON and OFF medication states in Parkinson’s disease

### Benoit Duchet^1,2^, Filippo Ghezzi^3^, Gihan Weerasinghe^1,2^, Gerd Tinkhauser^4^, Andrea A Kuhn^5^, Peter Brown^1,2^, Christian Bick^6,7,8^, Rafal Bogacz^1,2^

#### ^1^University of Oxford, Nuffield Department of Clinical Neuroscience, Oxford, United Kingdom; ^2^University of Oxford, MRC Brain Network Dynamics Unit, Oxford, United Kingdom; ^3^University of Oxford, Department of Physiology, Anatomy, and Genetics, Oxford, United Kingdom; ^4^Bern University Hospital and University of Bern, Department of Neurology, Bern, Switzerland; ^5^Charité – Universitätsmedizin Berlin, Department of Neurology, Movement Disorder and Neuromodulation Unit, Berlin, Germany; ^6^University of Oxford, Oxford Centre for Industrial and Applied Mathematics, Mathematical Institute, Oxford, United Kingdom; ^7^University of Exeter, Centre for Systems, Dynamics, and Control and Department of Mathematics, Exeter, United Kingdom; ^8^University of Exeter, EPSRC Centre for Predictive Modelling in Healthcare, Exeter, United Kingdom

##### **Correspondence:** Benoit Duchet (benoit.duchet@ndcn.ox.ac.uk)

*BMC Neuroscience* 2020, **21(Suppl 1)**:P8

Parkinson’s disease motor symptoms are associated with an increase in subthalamic nucleus beta band oscillatory power. However, these oscillations are phasic, and a growing body of evidence suggests that beta burst duration may be of critical importance to motor symptoms, making insights into the dynamics of beta bursting generation valuable. In this study, we ask the question “Can average burst duration reveal how dynamics change between the ON and OFF medication states?”. Our analysis of local field potentials from the subthalamic nucleus demonstrates using linear surrogates that the system generating beta oscillations acts in a more non-linear regime OFF medications and that the change in the degree of non-linearity is correlated with motor impairment. We further narrow-down dynamical changes responsible for changes in temporal patterning of beta oscillations between medication states by fitting to data biologically inspired models, and simpler models of the beta envelope (Fig 1). Finally, we show that the non-linearity can be directly extracted from average burst duration profiles under the assumption of constant noise in envelope models, revealing that average burst duration profiles provide a window into burst dynamics, which may underlie the success of burst duration as a biomarker. In summary, we have demonstrated a relationship between average burst duration profiles, dynamics of the system generating beta oscillations, and motor impairment, which puts us in a better position to understand the pathology and improve therapies.

**Fig. 1:**
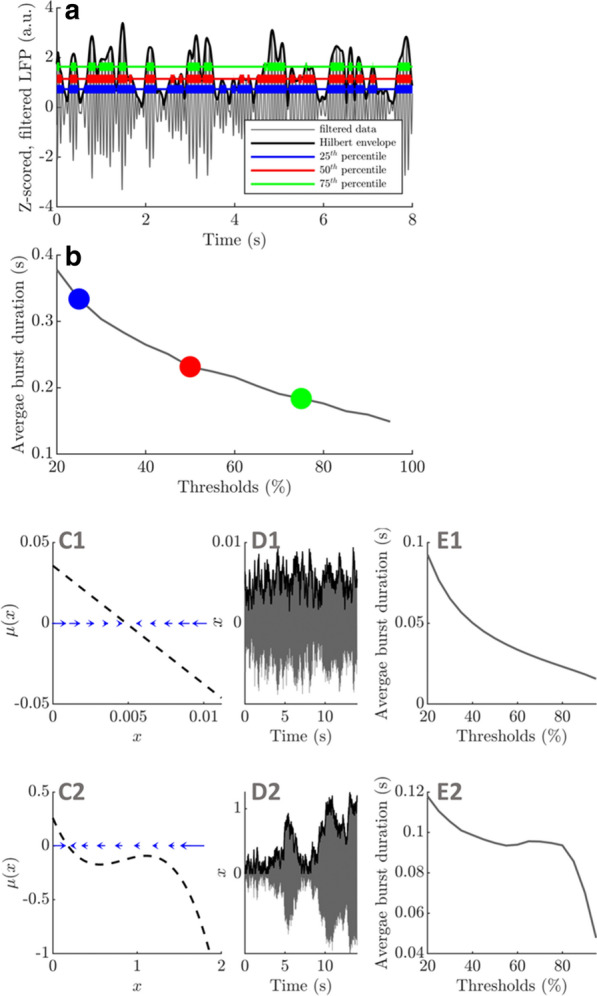
**A, B** Average burst duration profiles are obtained by computing beta envelope average burst duration for a range of thresholds. Considering envelope models of the form dXt=- µ(Xt)dt + ζdWt, where µ is the drift function, W a Wiener process, and ζ a constant noise parameter, we illustrate with two examples the link between envelope dynamics (**C1, C2**) and average burst duration profiles (**E1, E2**)

## P9 Introducing EBRAINS: European infrastructure for brain research

### Wouter Klijn^1^, Sandra Diaz^1^, Sebastian Spreizer^2^, Thomas Lippert^3^, Spiros Athanasiou^4^, Yannis Ioannidis^5^, Abigail Morrison^6^, Evdokia Mailli^4^, Katrin Amunts^7^, Jan Bjaalie^8^

#### ^1^Forschungszentrum Jülich, Jülich Supercomputing Centre, Simulation Lab Neuroscience,, Jülich, Germany; ^2^University of Freiburg, Bernstein Center Freiburg, Freiburg, Germany; ^3^Forschungszentrum Jülich, Institute for Advanced Simulation, Jülich Supercomputing Centre, Jülich, Germany; ^4^Athena Research & Innovation Center, Marousi, Greece; ^5^National & Kapodistrian University of Athens, Informatics & Telecom, Athens, Greece; ^6^Forschungszentrum Jülich, Institute of Neuroscience and Medicine (INM-6), Jülich, Germany; ^7^Forschungszentrum Jülich, Institute of Neuroscience and Medicine (INM 1), Jülich, Germany; ^8^University of Oslo, Institute of Basic Medical Sciences, Oslo, Norway

##### **Correspondence:** Wouter Klijn (w.klijn@fz-juelich.de)

*BMC Neuroscience* 2020, **21(Suppl 1)**:P9

The Human Brain Project (HBP), the ICT-based Flagship project of the EU, is developing EBRAINS - a research infrastructure providing tools and services which can be used to address challenges in brain research and brain-inspired technology development. EBRAINS will allow the creation of the necessary synergy between different national efforts to address one of the most challenging targets of research. This presentation will illustrate the services of the EBRAINS infrastructure with three use cases spanning the immensely diverse neuroscience field.

The first case is about Viktoria, a researcher who received a grant to investigate the distribution of interneuron types in the cortex and their activity under specific conditions. She needs a place to store, publish and share the data collected to add to the body of knowledge on the human brain. She contacts the HBP service desk and her case is forwarded to the data curation team, a part of the EBRAINS High Level Support Team. The data curators provide data management support and help make her data FAIR by registering it in the Knowledge Graph [1] and the Brain Atlas [2]. Her data is stored for 10 years, given a DOI to allow citations, and can be used by tools integrated in EBRAINS.

The second case is about Johanna, who has developed a software package for the analysis of iEEG data and now wants this tool to be used by as many researchers as possible. She contacts the HBP service desk and is put in contact with the EBRAINS technical coordination team. A co-design process is started together with the co-simulation framework developers, and her software is integrated into the simulation and analysis framework. After integration, Johanna’s tool can now be used with experimental data as well as to simulated iEEG data. Her tool is integrated into the operations framework of EBRAINS and is easily deployed on the HPC resources available through EBRAINS.

The third use case is about Jim, a neuroscience professor with a strong focus on teaching. After learning about the HBP he explores the EBRAINS website and discovers the wide range of educational tools available. NEST Desktop (https://github.com/babsey/nest-desktop), for instance, is a web accessible interface for spiking neuron networks. It allows the creation of a complete simulation with less than 10 mouse clicks, without the need to install any software. The output of the simulation can be then ported to Jupyter notebooks hosted on the EBRAINS’ systems to perform additional analysis. The functionality is accompanied with online MOOCs and detailed documentation to provide him with enough material to fill multiple courses on neuroscience.

With the EBRAINS infrastructure the HBP is delivering a set of tools and services in support of all aspects of neuroscience research. Get more information at: www.ebrains.eu or email the service desk at: support@ebrains.eu

**Acknowledgments:** This research has received funding from the European Union’s Horizon 2020 Framework Programme for Research and Innovation under the Specific Grant Agreement No. 785907 (Human Brain Project SGA2).

**References**

1. Amunts K, et al. The Human Brain Project—Synergy between neuroscience, computing, informatics, and brain-inspired technologies. PLoS biology. 2019; 17(7): e3000344.
2. Bjerke IE, et al. Data integration through brain atlasing: Human Brain Project tools and strategies. European Psychiatry. 2018; 50: 70–76.

## P10 Using adaptive exponential integrate-and-fire neurons to study general principles of patho-topology of cerebellar networks

### Maurizio De Pitta^1^, Jose A O Rodrigues^2^, Juan Sustacha^3^, Giulio Bonifazi^1^, Alicia Nieto-Reyes^2^, Sivan Kanner^4^, Miri Goldin^3^, Ari Barzilai^5^, Paolo Bonifazi^3^

#### ^1^Basque Center for Applied Mathematics, Bilbao, Spain; ^2^University of Cantabria, Department of Mathematics, Statistics and Computer Science, Santander, Spain; ^3^BioCruces Health Research Institute, Barakaldo, Spain; ^4^Friedrich Miescher Institute for Biomedical Research, Basel, Switzerland; ^5^Tel Aviv University, Department of Life Sciences, Ramat Aviv, Israel

##### **Correspondence:** Maurizio De Pitta (maurizio.depitta@gmail.com)

*BMC Neuroscience* 2020, **21(Suppl 1)**:P10

(A-T) is an example of a systemic genetic disease impacting the cerebellar circuit’s structure and function. Kanner et al. [1] have shown how the A-T phenotype in mice correlates with severe glial atrophy and increased synaptic markers, resulting in altered cerebellar networks’ dynamics. In particular, experiments in cerebellar cultures showed a disruption of networks’ synchronizations, which were recovered by replacements of A-T glial cells with healthy ones. Notably, the only presence of healthy astrocyte was sufficient to restore the physiological synaptic puncta level between mutated neurons. In the intact cerebellar circuits, glial morphological alterations and an increase in inhibitory synaptic connectivity markers were first reported and correlated (preliminary unpublished results) with an increase in the complex spiking of the Purkinje cells (PCs). In order to understand and model these structural-functional circuits’ alterations, we developed a simplified model of the cerebellar circuit. To this aim, we adopt the adaptive Exponential Integrate-and-Fire (aEIF) neuron model in different parameter configurations, to capture essential functional features of four different cell types: granule cells and excitatory neurons of the inferior olive (IONs); Purkinje cells and inhibitory neurons of the Deep Cerebellar Nuclei (DPNNs). Next, we explore different degrees of connectivity and synaptic weights, the dynamics of the simplified cerebellar circuitry. Our simulations suggest that the concomitant increased number of inhibitory connections from PC to DPNNs, and from DPNNs to IONs, ultimately results in a disinhibited IONs dynamics. As a consequence, IONs provide a higher rate of excitation to PCs within the cerebellar loop, which finally leads to higher complex spiking frequency in PCs. These results provide new insights into the dysfunctional A-T cerebellar dynamics and open a new perspective for targeted pharmacological treatments.

**Acknowledgements:** We thank the ‘Junior Leader’ Fellowship Program by ‘la Caixa’ Banking Foundation (Grant LCF/BQ/LI18/11630006).

**Reference**

1. Kanner S, et al. Astrocytes restore connectivity and synchronization in dysfunctional cerebellar networks. Proceedings of the National Academy of Sciences. 2018; 115(31): 8025-8030.

## P11 Effect of interglomerular inhibitory networks on olfactory bulb odor representations

### Daniel Zavitz^1^, Isaac Youngstrom^2^, Matt Wachowiak^2^, Alla Borisyuk^1^

#### ^1^University of Utah, Department of Mathematics, Salt Lake City, United States of America; ^2^University of Utah, Department of Neurobiology and Anatomy, Salt Lake City, United States of 

##### **Correspondence:** Daniel Zavitz (zavitz@math.utah.edu)

*BMC Neuroscience* 2020, **21(Suppl 1)**:P11

Lateral inhibition is a fundamental feature of circuits that process sensory information. In the mouse olfactory system, inhibitory interneurons called short axon cells initially mediate lateral inhibition between glomeruli, the functional units of early olfactory coding and processing. However, their interglomerular connectivity and its impact on odor representations is not well understood. To explore this question, we constructed a computational model of the interglomerular inhibitory network using detailed characterizations of short axon cell morphologies and simplified intraglomerular circuitry. We then examined how this network transformed glomerular patterns of odorant-evoked sensory input (taken from previously-published datasets) at different values of interglomerular inhibition selectivity. We examined three connectivity schemes: selective (each glomerulusconnects to few others with heterogeneous strength), nonselective (glomeruli connect to most others with heterogeneous strength) and or global (glomeruli connect to all others with equal strength). We found that both selective and nonselective interglomerular networks could mediate heterogeneous patterns of inhibition across glomeruli when driven by realistic sensory input patterns, but that global inhibitory networks were unable to produce input-output transformations that matched experimental data.

We further studied networks whose interglomerular connectivity was tuned by sensory input profile. We found that this network construction improved contrast enhancement as measured by decorrelation of odor representations. These results suggest that, despite their multiglomerular innervation patterns, short axon cells are capable of mediating odorant-specific patterns of inhibition between glomeruli that could, theoretically, be tuned by experience or evolution to optimize discrimination of particular odorants.

## P12 A population level model of movement disorder oscillations and deep brain stimulation

### Nada Yousif^1^, Peter Bain^2^, Dipankar Nandi^3^, Roman Borisyuk^4^

#### ^1^University of Hertfordshire, Hatfield, United Kingdom; ^2^Imperial College London, Division of Brain Sciences, London, United Kingdom; ^3^Imperial College Healthcare NHS Trust, Neurosurgery, London, United Kingdom; ^4^University of Exeter, College of Engineering, Mathematics and Physical Sciences, Exeter, United Kingdom

##### **Correspondence:** Nada Yousif (n.yousif@herts.ac.uk)

*BMC Neuroscience* 2020, **21(Suppl 1)**:P12

The presence of neural oscillations is thought to be a hallmark of two of the most common movement disorders, Parkinson’s disease (PD) and Essential tremor (ET). The symptoms of PD are tremor, slowness of movement and stiffness and is caused by the loss of dopaminergic neurons in the substantia nigra. Although the pathological changes in the basal ganglia network are not yet fully understood, it is widely accepted that beta-band (15-30 Hz) oscillations play a role. Essential tremor (ET) affects up to one percent of adults over 40 years of age and is characterized by an uncontrollable shaking of the affected body part [1-3]. The neurophysiological basis of ET remains unknown, but pathological neural oscillations in the thalamocortical-cerebellar network are also implicated in generating symptoms.

In our previous network study of PD, we studied how a multi-channel model of Wilson-Cowan oscillators representing the STn-GPe behaved in healthy and Parkinsonian conditions. We found that oscillations exist for a much wider range of parameters in the Parkinsonian case and demonstrated how an input representing DBS caused the oscillations to become chaotic and flattened the power spectrum. Looking at ET, we again used a mean-field approach combined with intraoperative local field potential recordings from the Vim via DBS electrodes, and simultaneous electromyographic activity from the contralateral affected limb(s). We used the Wilson-Cowan approach to model the thalamocortical-cerebellar network implicated in ET. We found that the network exhibited oscillatory behaviour within the tremor frequency range of 4-5 Hz, as did our electrophysiological data. Applying a DBS-like input to the modelled network had the effect of suppressing these oscillations. Our two previous studies therefore show that the dynamics of the cerebellar-basal ganglia thalamocortical network support oscillations at frequency ranges relevant to movement disorders. The application of a DBS-like input into the modelled networks disrupts such pathological activity. We believe that this is an important way to study the impact of DBS on the human brain and should be used in conjunction with experimental recordings of neural activity as well as with single neuron biophysical modelling work.

In this work we present new results from a combined model which exhibits Parkinsonian oscillations in the beta band, oscillations in the tremor frequency range, as well as oscillations in the gamma band which we term healthy [4,5]. We find critical boundaries in the parameter space of the model separating regions with different dynamics. We go on to examine the transition from one oscillatory regime to another behavior and the impact of DBS on these two types of pathological activity. This approach will not only allow us to better understand the mechanisms of DBS, but allow us to optimize the lengthy and difficult clinical process of parameter setting via trial and error, upon which the cited improvement in symptoms is reliant [6,7]. Furthermore, with the advent of electrodes with more contacts this process is becoming increasingly difficult. Hence, the need for a theoretical understanding of DBS is particularly important at present.

**References**

1. Louis ED, Ottman R, Allen Hauser W. How common is the most common adult movement disorder? Estimates of the prevalence of essential tremor throughout the world. Movement Disorders. 1998; 13(1): 5-10.
2. Brin MF, Koller W. Epidemiology and genetics of essential tremor. Movement Disorders. 1998; 13(S3): 55-63.
3. Deuschl G, Bain P, Brin M, Ad Hoc Scientific Committee. Consensus statement of the movement disorder society on tremor. Movement Disorders. 1998; 13(S3): 2-23.
4. Beudel M, Brown P. Adaptive deep brain stimulation in Parkinson’s disease. Parkinsonism & related disorders. 2016; *22*: S123-S126.
5. Fischer P, et al. Subthalamic nucleus gamma activity increases not only during movement but also during movement inhibition. Elife. 2017; 6: e23947.
6. Rizzone M, et al. Deep brain stimulation of the subthalamic nucleus in Parkinson’s disease: effects of variation in stimulation parameters. Journal of Neurology, Neurosurgery & Psychiatry. 2001; 71(2): 215-219.
7. Moro E, et al. The impact on Parkinson’s disease of electrical parameter settings in STN stimulation. Neurology. 2002; 59(5): 706-713.

## P13 Organization of connectivity between areas in the monkey frontoparietal network

### Bryan Conklin^1^, Steve Bressler^2^

#### ^1^Florida Atlantic University, Center for Complex Systems & Brain Science, West Palm Beach, Florida, United States of America; ^2^Florida Atlantic University, West Palm Beach, Florida, United States of America

##### **Correspondence:** Bryan Conklin (bconkli4@fau.edu)

*BMC Neuroscience* 2020, **21(Suppl 1)**:P13

Anatomical projections between cortical areas are known to condition the set of observable functional activity in a neural network. The large-scale cortical monkey frontoparietal network (FPN) has been shown to support complex cognitive functions. However, the organization of anatomical connectivity between areas in the FPN supporting such behavior is unknown. To identify the connections in this network, over 40 tract-tracing studies were collated according to the Petrides & Pandya [1] parcellation scheme, which provides a higher resolution map for the areas making up the FPN than other schemes. To understand how this structural profile can give rise to cognitive functions, a graph theoretic investigation was conducted in which the FPN’s degree distribution, structural motifs and small-worldness were analyzed. We present a new connectivity matrix detailing the anatomical connections between all frontal and parietal areas of the parcellation scheme. First, this matrix was found to have in and out-degree distributions that did not follow a power-law. Instead they were each best approximated by a Gaussian distribution, signifying that the connectivity of each area in the FPN is relatively similar and that it does not rely on hubs. Second, the dynamical relay motif, M9, was found to be overrepresented in the FPN. This 3-node motif is the optimal arrangement for near-zero and non-zero phase synchrony to propagate through the network. Finally, the FPN was found to utilize a small-world architecture. This allows for simultaneous integration and specialization of function. Important aspects of cognition such as attention and working memory have been shown to require both integration and specialization in order to function properly using near-zero and non-zero phase synchrony. Further, they benefit from the reliability afforded by the FPN’s homogenous connectivity profile which acts as a substrate resilient to targeted structural insult but vulnerable to a random attack. This suggests the diseases that impair cognitive function supported by the FPN may owe their effectiveness to a random attack strategy. These findings provide a candidate topological mechanism for the synchrony observed during complex cognitive functions in the M9 dynamical relay motif. The results also serve as a benchmark to be used in the network-level treatment of neurological disorders such as Alzheimer’s or Parkinson’s disease where the types of cognition the FPN supports are impaired. Finally, they can inform future neuromorphic circuit designs which aim to perform certain aspects of cognition.

**References**

1. Petrides M, Pandya DN. Efferent Association Pathways from the Rostral Prefrontal Cortex in the Macaque Monkey. Journal of Neuroscience. 2007; 27: 11573–11586.

## P14 Avalanches, criticality and correlations in self-organised nanoscale networks

### Josh Mallinson^1^, Shota Shirai^1^, Susant Acharya^1^, Saurabh Bose^1^, Matthew Pike^1^, Edoardo Galli^1^, Matthew Arnold^2^, Simon Brown^1^

#### ^1^University of Canterbury, School of Physical and Chemical Sciences, Christchurch, New Zealand; ^2^University of Technology Sydney, School of Mathematical and Physical Sciences, Sydney, Australia

##### **Correspondence:** Josh Mallinson (josh.mallinson@pg.canterbury.ac.nz)

*BMC Neuroscience* 2020, **21(Suppl 1)**:P14

Neuronal avalanches are one of the key characteristic features of signal propagation in the brain [1]. These avalanches originate from the complexity of the network of neurons and synapses, which are widely believed to form a self-organised critical system. Criticality is hypothesised to be intimately linked to the brain’s computational power [2,3] but efforts to achieve neuromorphic computation have so far focused on highly organised architectures, such as integrated circuits [4] and regular arrays of memristors [5]. To date, little attention has been given to developing complex network architectures that exhibit criticality and thereby maximise [6] computational performance. We show here, using methods developed by the neuroscience community [7], that electrical signals from self-organised percolating networks of nanoparticles [8] exhibit brain-like correlations and criticality [9]. Specifically, the sizes and durations of avalanches of switching events are power-law distributed, and the power-law exponents satisfy rigorous criteria for criticality. Additionally we show that both the networks and their dynamics are scale-free. These networks provide a low-cost platform for computational approaches that rely on spatiotemporal correlations, such as reservoir computing, and are a significant step towards creating neuromorphic device architectures.

**References**

1. Beggs JM, Plenz D. Neuronal avalanches in neocortical circuits. Journal of Neuroscience Research. 2003; 23: 11167-11177.
2. Munoz MA. Colloquium: criticality and dynamical scaling in living systems. Reviews of Modern Physics. 2018; 90(3): 031001.
3. Cocchi L, Gollo LL, Zalesky A, Breakspear M. Criticality in the brain: a synthesis of neurobiology, models and cognition. Progress in Neurobiology. 2017; 158: 132-152.
4. Merolla PA, et al. A million spiking-neuron integrated circuit with a scalable communication network and interface. Science. 2014; 345(6197): 668-673.
5. Burr GW, et al. Neuromorphic computing using non-volatile memory. Advances in Physics: X. 2017; 2(1), 89-124.
6. Srinivasa N, Stepp ND, Cruz-Albrecht J. Criticality as a set-point for adaptive behavior in neuromorphic hardware. Frontiers in Neuroscience. 2015; 9: 449.
7. Friedman N, et al. Universal critical dynamics in high resolution neuronal avalanche data. Physical Review Letters. 2012; 108(20): 208102.
8. Sattar A, Fostner S, Brown SA. Quantized conductance and switching in percolating nanoparticle films. Physical Review Letters. 2013; 111(13): 136808.
9. Mallinson JB, et al. Avalanches and criticality in self-organised nanoscale networks. Science Advances. 2019; 5(11): eaaw8438.

## P15 Learning sequences of correlated patterns in recurrent networks

### Subhadra Mokashe^1^, Nicolas Brunel^2^

#### ^1^Duke University, Neurobiology, Durham, North Carolina, United States of America; ^2^Duke University, Neurobiology and Physics, Durham, North Carolina, United States of America

##### **Correspondence:** Subhadra Mokashe (subhadramokashe@gmail.com)

*BMC Neuroscience* 2020, **21(Suppl 1)**:P15

What determines the format of memory representations in cortical networks is a subject of active research. During memory tasks, the retrieval of stored memories is characterized either by the persistent elevation in the firing rate of a set of neurons (‘persistent activity’) [1] or by ordered transient activation of different sets of neurons (‘sequential activity’) [2]. Multiple theoretical studies have shown that temporally symmetric Hebbian learning rules give rise to fixed point attractor representation of memory (e.g., [3] and references therein), while temporally asymmetric learning rules lead to a dynamic sequential representation of memories (e.g., [4] and references therein). These studies assume that inputs to the network during learning have no temporal correlations.

The sensory information received by brain networks is likely to be temporally correlated. We study temporally asymmetric Hebbian learning rules in a recurrent network of rate-based neurons in the presence of temporal correlations in the inputs and characterize how the inputs shape the network dynamics and memory representation using both numerical simulations and mean-field analysis. We show that the network dynamics depend on the temporal correlations in the input stream the network receives. For inputs with short correlation timescale, the network exhibits sequential activity (Fig. 1 A left), while for longer correlations within the stream of input, the network settles into a fixed point attractor during retrieval (Fig. 1A right). At intermediate value of correlations, the network partially traverses the input sequence before settling into an attractor state (Fig. 1 A middle). We find that correlations increase the sequential memory capacity of the network. Non-linear learning rules increase the range of timescale of correlation for which the networks represent the memories as sequential activity in the network (Fig. 1 B). We also show that the network maintains a sequential representation, both in the case of sequences of discrete patterns and in the continuum limit (Fig. 1 C). Our work thus suggests that the correlation time scales of inputs at the time of learning have a strong influence on the nature of network dynamics during retrieval.

**Fig. 1:**
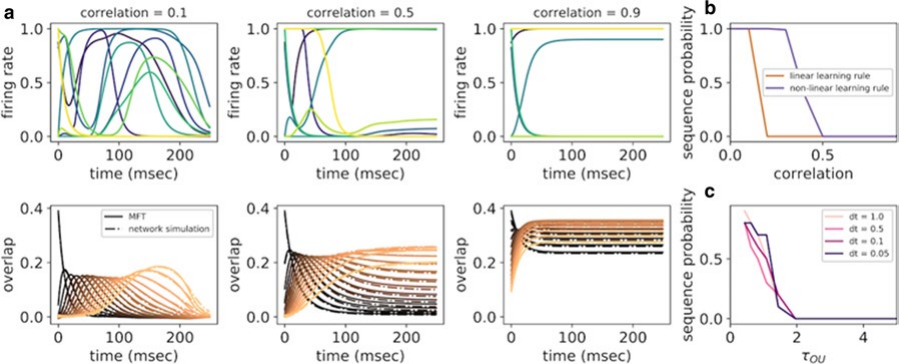
**A** The activity of neurons (top) and overlaps with stored patterns from the simulations and mean-field theory (bottom), for short, intermediate, and long correlation timescale (L to R). Probability of sequence retrieval with a linear learning and non-linear learning rule as a function of correlation **B** and as a function of tau_OU, for different discretizations of a continuous OU process **C**

**References**

1. Funahashi S, Bruce CJ, Goldman-Rakic PS. Mnemonic coding of visual space in the monkey’s dorsolateral prefrontal cortex. Journal of Neurophysiology. 1989; 61(2): 331–349.
2. Harvey CD, Coen P, Tank DW. Choice-specific sequences in parietal cortex during a virtual-navigation decision task. Nature. 2012; 484(7392): 62-68.
3. Pereira U, Brunel N. Unsupervised learning of persistent and sequential activity. Frontiers in Computational Neuroscience. 2020;13: 97.
4. Gillett M, Pereira U, Brunel N. Characteristics of sequential activity in networks with temporally asymmetric Hebbian learning. BioRxiv. 2019; 818773.

## P16 A computational model for time cell-based learning of interval timing

### Sorinel Oprisan^1^, Tristan Aft^1^, Michael Cox^2^, Mona Buhusi^3^, Catalin Buhusi^3^

#### ^1^College of Charleston, Department of Physics and Astronomy, Charleston, South Carolina, United States of America; ^2^Clemson University, Mechanical Engineering, Clemson, South Carolina, United States of America; ^3^Utah State University, Psychology, Logan, Utah, United States of America

##### **Correspondence:** Sorinel Oprisan (oprisans@cofc.edu)

*BMC Neuroscience* 2020, **21(Suppl 1)**:P16

Lesion and pharmacological studies found that interval timing is the emergent property of an extensive neural network that includes the prefrontal cortex (PFC), the basal ganglia (BG), and the hippocampus (HIP). We used our Striatal Beat Frequency (SBF) model with a large number of PFC oscillators to produce beats from the coincidence detection performed by BG [1,2]. The response of the PFC-BG neural network provides an output that (1) accurately identifies the criterion time, i.e., the time at which the reinforcement was presented during reinforced trails, and (2) is scalar, i.e., the prediction error is proportional to the criterion time. We found that, although the PFC-BG can create beats, the accuracy of the timing depends on the number of PRC oscillators and the frequency range they cover [3,4].

The ability to discriminate between multiple durations requires a metric space in which durations can be compared. We hypothesized that time cells, which were recently discovered in the hippocampus and ramp-up their firing when the subject is at a specific temporal marker in a behavioral test, can offer a time base for interval timing. We expanded the SBF model by incorporating the HIP time cells that (1) provide a natural time base, and (2) could be the cellular root of the scalar property of interval timing observed in all behavioral experiments (see [5]). Our model of interval timing learning assumes that there are two stages of this process. First, during the reinforced trials, the subject learns the boundaries of the temporal duration. This process is similar to the HIP space cell activity that first forms an accurate spatial map of the edges of the environment. Subsequently, the time cells are recruited to cover the entire to-be-timed duration uniformly. Without any learning rule, i.e., without any feedback from the PFC-BG network, the population of time cells simply produces a uniform average time field. In our computational model, the learning rule requires the HIP time cell to adjust their activity to mirror the output of the PFC-BG network. A plausible mechanism for the modulation of HIP time cell activity could involve dopamine released during the reinforced trials. We tested numerically different learning rules and found that one of the most efficient in terms of the number of trails required until convergence is the diffusion-like, or nearest-neighbor, algorithm.

**References**

1. Oprisan SA, Aft T, Buhusi M, Buhusi C. Scalar timing in memory: A temporal map in the hippocampus. Journal of Theoretical Biology. 2018; 438: 133 – 142.
2. Oprisan SA, Buhusi M, Buhusi CV. A population-based model of the temporal memory in the hippocampus. Frontiers in Neuroscience. 2018; 12: 521.
3. Buhusi CV, Oprisan SA, Buhusi M. Clocks within Clocks: timing by Coincidence Detection. Current Opinion in Behavioral Sciences. 2016; 8: 207-213.
4. Buhusi CV, Reyes MB, Gathers CA, Oprisan SA, Buhusi M. Inactivation of the Medial-Prefrontal Cortex Impairs Interval Timing Precision, but Not Timing Accuracy or Scalar Timing in a Peak-Interval Procedure in Rats. Frontiers in Integrative Neuroscience. 2018; 12: 20.
5. Oprisan SA, Buhusi CV. What is all the noise about in interval timing? Philosophical Transactions of the Royal Society B: Biological Sciences. 2014; 369(1637): 20120459.

## P17 The impacts of the connectome on coupled networks of wilson-cowan models with homeostatic plasticity

### Wilten Nicola^1^, Sue A Campbell^2^

#### ^1^University of Calgary, Calgary, Canada; ^2^University of Waterloo, Applied Mathematics, Waterloo, Canada

##### **Correspondence:** Wilten Nicola (wilten.nicola@ucalgary.ca)

*BMC Neuroscience* 2020, **21(Suppl 1)**:P17

We study large networks of Wilson-Cowan neural field systems with homeostatic plasticity. These networks have been known to display rich dynamical states such consisting of a single recurrently coupled, or two cross-coupled nodes [1]. These dynamics include chaos, mixed mode oscillations and chaos, and synchronized chaos, even under these simple connectivity profiles in small networks. Here, we consider these networks with connectomes that display so-called L1 normalization but are otherwise arbitrary under large network limits. We find that for the majority of classical connectomes considered (Random, Small World), the network displays a large-scale chaotic synchronization to the attractor states and bifurcation sequence of a single recurrently coupled node as in [1]. However, connectomes that display sufficiently large pairs of eigenvalues can trigger multiple Hopf bifurcations which can potentially collide in Torus bifurcations that can destabilize the synchronized, single node attractor solutions. Our analysis demonstrates that for Wilson-Cowan systems with homeostatic plasticity, the dominant determinant of network activity is not the connectome directly, but rather the connectome’s ability to generate large eigenvalues that can induce multiple nearby Hopf bifurcations. If the connectome cannot generate these large pairs of eigenvalues, the dynamics of the network considered become limited to the dynamics of a single recurrently coupled node.

**Reference**

1. Nicola W, Hellyer PJ, Campbell SA, Clopath C. Chaos in homeostatically regulated neural systems. Chaos: An Interdisciplinary Journal of Nonlinear Science. 2018; *28*(8): 083104.

## P18 Inhibitory gating in the dentate gyrus

### Claudio Mirasso^1^, Cristian Estarellas^1^, Santiago Canals^2^

#### ^1^Universitat de les Illes Balears, Instituto de Física Interdisciplinar y Sistemas Complejos, Palma de Mallorca, Spain; ^2^Instituto de Neurociencias, San Juan de Alicante, Spain

##### **Correspondence:** Claudio Mirasso (claudio@ifisc.uib-csic.es)

*BMC Neuroscience* 2020, **21(Suppl 1)**:P18

Electrophysiological recordings have demonstrated a tight inhibitory control of hilar interneurons over Dentate Gyrus granule cells (DGgc) [1,2]. This excitation/inhibition balance is crucial for information transmission [3] and likely relies on inhibitory synaptic plasticity [4]. Our experiments show that LTP induction in the Perforant Pathway (PP) not only potentiates glutamatergic synapses, but unexpectedly decreases feed-forward inhibition in the DG, facilitating activity propagation in the circuit and modifying the long-range connectivity in the brain. To investigate this phenomenon, we propose to study a circuit of populations of point neurons described by the Izhikevich model. The model contains entorhinal cortex (EC) neurons, DGgc, mossy cells, basket cells and hilar interneurons. The proportion of neurons per population and the connectivity of the neural network is based on anatomical published data and is fitted to achieve experimental electrophysiological in vivo recordings [2]. The study of the effect of LTP in the local circuit of the DG is performed in the model adapting synaptic weights in the EC projections. The results obtained from the model, before and after LTP induction, support the counterintuitive experimental observation of synaptic depression in the feed-forward inhibitory connection induced by LTP. We show that LTP increases the efficiency of the glutamatergic input to recruit the inhibitory network, resulting in a reciprocal cancellation of the basket cell population activity. We validate the result of the model by electrophysiological experiments inducing LTP in the PP of anaesthetized mice *in vivo* and recording excitatory and inhibitory currents *in vitro* in the same animals. Overall, our findings suggest that LTP of the EC input increases the excitation/inhibition balance, and facilitates activity propagation to the next station in the circuit by recruiting an interneuron-interneuron network that inhibits the tight control of basket cells over DGgc firing.

**References**

1. Bragin A, et al. Gamma (40-100 Hz) oscillation in the hippocampus of the behaving rat. Journal of Neuroscience. 1995; 15(1): 47-60.
2. Pernía-Andrade AJ, Jonas P. Theta-gamma-modulated synaptic currents in hippocampal granule cells in vivo define a mechanism for network oscillations. Neuron. 2014; 81(1): 140–152.
3. Bartos M, Vida I, Frotscher M, Jörg G, Jonas P. Rapid Signaling at Inhibitory Synapses in a Dentate Gyrus Interneuron Network. Journal of Neuroscience. 2001; 21(8): 2687-2698.
4. Vogels TP, Sprekeler H, Zenke F, Clopath C, Gerstner W. Inhibitory plasticity balances excitation and inhibition in sensory pathways and memory networks. Science. 2011; 334(6062): 1569-73.

## P19 Neuroscience gateway enabling modeling and data processing using high performance and high throughput computing resources

### Amitava Majumdar^1^, Subhashini Sivagnanam^1^, Kenneth Yoshimoto^1^, Dave Nadeau^1^, Martin Kandes^1^, Trever Petersen^1^, Ramon Martinez^2^, Dung Troung^2^, Arnaud Delorme^2^, Scott Makeig^2^, Ted Carnevale^3^

#### ^1^University of California, San Diego, San Diego Supercomputer Center, La Jolla, California, United States of America; ^2^University of California, San Diego, Institute for Neural Computation, La Jolla, California, United States of America; ^3^Yale University, Neurobiology, New Haven, Connecticut, United States of America

##### **Correspondence:** Amitava Majumdar (majumdar@sdsc.edu)

*BMC Neuroscience* 2020, **21(Suppl 1)**:P19

The Neuroscience Gateway (NSG) has been serving the computational neuroscience community since early 2013. Its initial goal was to reduce technical and administrative barriers that neuroscientists face in accessing and using high performance computing (HPC) resources needed for large scale neuronal modeling projects. For this purpose, NSG provided tools and software that require and run efficiently on HPC resources available as a part of the US XSEDE (Extreme Science and Engineering Discovery Environment) program that coordinates usage of academic supercomputers. Since around 2017 experimentalists such as cognitive neuroscientists, psychologists and biomedical researchers started to use NSG for their neuroscience data processing, analysis and machine learning work. Data processing workloads are more suitable on high throughput computing (HTC) resources that are suitable for single core jobs typically run to process individual data sets of subjects. Machine learning (ML) workloads require use of GPUs for well-known ML frameworks such as TensorFlow. NSG is adapting to respond to the needs of experimental neuroscientists by providing HTC resources, in addition to already enabling successfully the computational neuroscience community for many years by providing HPC resources. Data processing focused work of experimentalists also require NSG to add various data functionalities, such as ability to transfer/store large data to/on NSG, validate the data, process same data by multiple users, publish final data products, visualize the data, search the data etc. These features are being add to NSG currently. Separately there is a demand from the neuroscience community to make NSG an environment where neuroscience tool developers can test, benchmark, and scale their newly developed tools and eventually disseminate their tools via the NSG for neuroscience users.

The poster will describe NSG from its beginning and how it is evolving for the future needs of the neuroscience community such as: (i) NSG has been successfully serving primarily the computational neuroscience community, as well as some data processing focused neuroscience researchers, until now; (ii) new features are added to make it a suitable and efficient dissemination environment for lab-developed neuroscience tools. These will allow tool developers to disseminate their lab-developed tools on NSG taking advantage of the current functionalities that are being well served on NSG for the last seven years such as a growing user base, an easy user interface, an open environment, the ability to access and run jobs on powerful compute resources, availability of free supercomputer time, a well-established training and outreach program, and a functioning user support system. All of these well-functioning features of NSG will make it an ideal environment for dissemination and use of lab-developed computational and data processing neuroscience tools; (iii) NSG is being enhanced such that it can have more seamless access to HTC resources provided by the Open Science Grid (OSG) and commercial cloud. This will allow data processing and machine learning oriented workloads to be able to take advantage of HTC and cloud resources including GPUs; (iv) New data management features are being added to NSG and these include the ability to transfer/upload large data, validate uploaded data, share and publish data etc.

## P20 Effects of dopamine on networks of barrel cortex

### Fleur Zeldenrust^1^, Chao Huang^2^, Prescilla Uijtewaal^3^, Bernhard Englitz^1^, Tansu Celikel^1^

#### ^1^Donders Institute for Brain, Cognition and Behaviour, Radboud University, Department of Neurophysiology, Nijmegen, Netherlands; ^2^University of Leipzig, Department of Biology, Leipzig, Germany; ^3^UMC Utrecht, Imaging Division, Department of Radiotherapy, Utrecht, Netherlands

##### **Correspondence:** Fleur Zeldenrust (fleurzeldenrust@gmail.com)

*BMC Neuroscience* 2020, **21(Suppl 1)**:P20

The responses of excitatory pyramidal cells and inhibitory interneurons in cortical networks are shaped by each neuron’s place in the network (connectivity of the network) and its biophysical properties (ion channel expression [1]), which are modulated by top-down neuromodulatory input, including dopamine. Using a recently developed ex vivo method [2], we showed that the activation of the D1 receptor (D1R) increases the information transfer of fast spiking, but not regular spiking, cells, by decreasing their threshold [3]. Moreover, we showed that these differences in neural responses are accompanied by faster decision-making on a behavioural level. However, how the single-cell changes in spike responses result in these behavioural changes is still unclear. Here, we aim to bridge the gap between behavioural and single cell effects by considering the effects of D1R activation on a network level.

We took a 3-step approach and simulated the effects of dopamine by lowering the thresholds of inhibitory but not excitatory neurons:

1. Network construction. We created a balanced network of L2/3 and L4 of the barrel cortex, consisting of locally connected integrate-and-fire neurons. We reconstructed the somatosensory cortex in soma resolution ([4], Fig. 1A), and adapted the number and ratio of excitatory and inhibitory neurons and the number of thalamic inputs accordingly.
2. Activity of the balanced state. The adaptations in the neural populations and connectivity resulted in a heterogeneous asynchronous regime [5] in L2/3, with highly variable single-neuron firing rates and suggesting a functional role of stimulus separation, and a ‘classical’ asynchronous regime in L 4, with more constant firing rates and suggestive of an information transmission role (Fig. 1B).
3. Functional effects. We used a spike-based FORCE learning [6,7] application, trained on either a gap-crossing task (data from [8]) or on a pole detection task (publicly available data from [9], Fig. 1C). We compared the results against a benchmark test consisting of a 3-layer deep neural net with a recurrent layer.

**Fig. 1:**
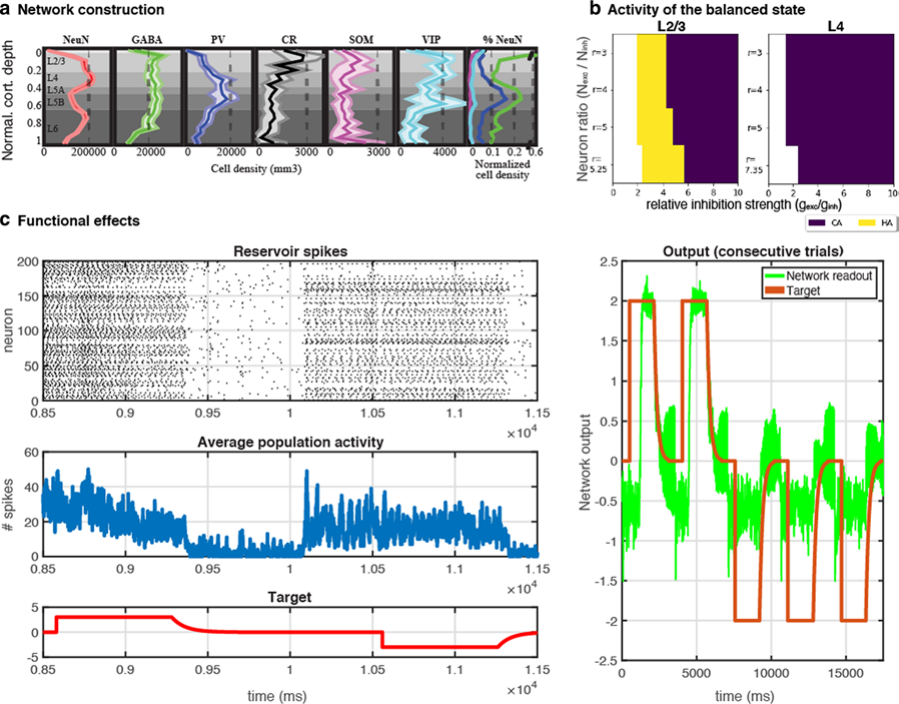
**A** Density of identified cellular populations across the six cortical layers. **B** Effects of the relative number of inhibitory neurons on the asynchronous state. Yellow indicates classical asynchronous (CA) dynamics, purple indicates a heterogeneous asynchronous (HA) regime, white indicates no asynchronous irregular state. **C** Spiking FORCE learning during a pole localization task

**References**

1. Azarfar A, Calcini N, Huang C, Zeldenrust F, Celikel T. Neural coding: A single neuron’s perspective. Neuroscience & Biobehavioral Reviews. 2018; 94: 238-247.
2. Zeldenrust F, de Knecht S, Wadman WJ, Denève S, Gutkin B. Estimating the information extracted by a single spiking neuron from a continuous input time series. Frontiers in Computational Neuroscience. 2017; 11: 49.
3. Calcini N, et al. Cell-type specific modulation of information transfer by dopamine. Cosyne Abstract 2019, Lisbon
4. Huang C, Zeldenrust F, Celikel T. DepartmentofNeurophysiology/Cortical-representation-of-touch-in-silico. GitHub. 2019; https://github.com/DepartmentofNeurophysiology/Cortical-representation-of-touch-in-silico (accessed March 2, 2020).
5. Ostojic S. Two types of asynchronous activity in networks of excitatory and inhibitory spiking neurons. Nature Neuroscience. 2014; 17: 594–600.
6. Sussillo D, Abbott LF. Generating Coherent Patterns of Activity from Chaotic Neural Networks. Neuron. 2009; 63: 544–57.
7. Nicola W, Clopath C. Supervised learning in spiking neural networks with FORCE training. Nature Communications. 2017; 8: 1–15.
8. Azarfar A, et al. An open-source high-speed infrared videography database to study the principles of active sensing in freely navigating rodents. GigaScience. 2018; 7(12); giy134.
9. Peron SP, Freeman J, Iyer V, Guo C, Svoboda K. A cellular resolution map of barrel cortex activity during tactile behavior. Neuron. 2015; 86(3); 783-799.

## P21 Synergy in the encoding of a moving bar by a retina

### Kuan H Chen^1^, Qi-Rong Lin^2^, Chi K Chen^2^

#### ^1^Academia Sinica, Taiwan; ^2^Academia Sinica, Institute for Physics, Taiwan

##### **Correspondence:** Kuan H Chen (nghdavid123@gmail.com)

*BMC Neuroscience* 2020, **21(Suppl 1)**:P21

To produce timely responses, animals must conquer delays from visual processing pathway by predicting motion. Previous studies [1] revealed that predictive information of motion is encoded in spiking activities of retinal ganglion cells (RGCs) early in the visual path. In order to study the predictive properties of a retina in a more systematic manner, stimuli in the form of a stochastic moving bar are used in experiments with retinas from bull frogs in a multi-electrode system. Trajectories of the bar are produced by Ornstein-Uhlenbeck (OU) processes with different time correlations (memories) induced by a butter-worth low-pass filter with various cut-off frequencies.

We then investigated the predictive properties of single RGC by calculating the time shifted mutual information (MI(x,r;δt)) between spiking output from RGCs and the bar trajectories. Intuitively, the peak position of MI(δt) is typically negative when considering the processing delay of the retina. Our measured peak positions of MI(δt) for some RGCs were characterized by both positive and negative peak position under low-pass OU (LPOU) stimulus. This finding indicates that some RGCs (P-RGCs) are predictive while the others are non-predictive (NP-RGCs). For LPOU with various correlation times, the MI peaks from the P-RGCs are positively correlated with the correlation times of the stimuli while those from the NP-RGCs are always around a fixed negative number (-50ms).

Furthermore, we apply principle component analysis [2] on the waveforms of stimuli preceding each of the neuron’s spike (spike triggered stimuli) to separate spikes into two clusters according to whether their projections to principle component are negative or positive. We find that predictive information can be extracted from the apparent non-predictive NP-RGCs when MI(δt) is obtained with spikes from each cluster. This last finding suggests that spikes from a single RGC might have different origins. Since the responses (r) from RGCs can carry information for both position (x) and velocity (v) of the moving bar, we have also performed partial information decomposition [3] for the mutual information between r and the combined state {x,v} which can be written as I[(x,v):r] = S + Ur + Ux + R where Ur and Ux are the unique contribution from x and v respectively while similarly R and S are the redundant and synergy contribution. We find that synergy from x and v is needed to produce anticipation. A simple spikes generation model with synergy from x and v is constructed to understand our experimental data.

**Fig. 1:**
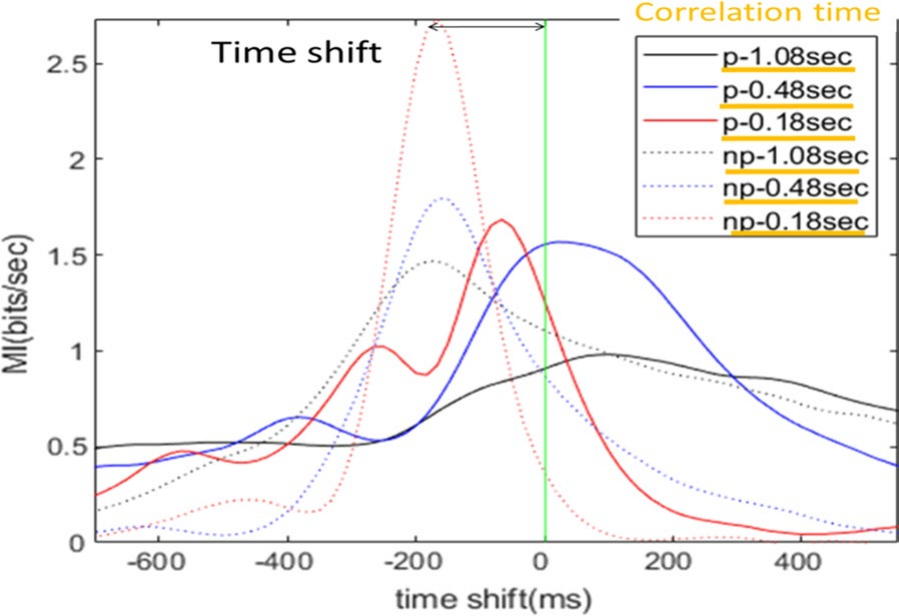
Two kinds of responses under low-passed-OU stimulation with different correlation time: Predicting cells (P, solid line) and Non-predicting cells (N, dot line)

**References**

1. Palmer SE, Marre O, Berry MJ, Bialek W. Predictive information in a sensory population. Proceedings of the National Academy of Sciences. 2015; 112(22): 6908-6913.
2. Geffen MN, de Vries SEJ, Meister M. Retinal Ganglion Cells Can Rapidly Change Polarity from Off to On. PloS Biology. 2007; 5(3): e65.
3. Williams PL, Beer RD. Nonnegative Decomposition of Multivariate Information. Arxiv. 2010; 1004.2515 *[preprint]*.

## P22 Retina as a negative group delay filter

### PoYu Chou^1^, Chi K Chen^2^

#### ^1^Academia Sinica, Taiwan; ^2^Academia Sinica, Institute for Physics, Taiwan

##### **Correspondence:** PoYu Chou (az6520555@gmail.com)

*BMC Neuroscience* 2020, **21(Suppl 1)**:P22

Anticipation is important for living organisms to survive. The anticipative dynamics is found in retina [1], which can compensate for the delay of visual signals during transmission and processing. Responses of retinas (r(t)) from bull frogs have been investigated for anticipative properties in a multi-electrode array system by using whole field stochastic stimulations (S(t)) generated by an Ornstein–Uhlenbeck (OU) process. Time correlated S(t) can be then created by passing the OU signal through a low pass filter with various cutoff frequencies. Anticipative properties of the elicited spikes from the retinas are then characterized by the method of time lag mutual information (TLMI) between r(t) and S(t) [1]. We find that only stimulations with long enough correlations can elicit anticipative responses from the retinas; similar to the finding of [1] in which information in the stimulation was time coded. However, information is being rate coded in the present experiment. Recently, Voss [2] proposed that a negative group delay filter can produce anticipative response to low-pass filtered random signals. To test this idea, it is shown that an NGD filter with appropriate parameters can indeed be used to produce TLMI from S(t) similar to those observed in experiments. Furthermore, experiments with dark and bright Gaussian light pulses further confirmed that retina can be considered as an NGD filter; but only for the dark pulses. This last finding and the NGD capability of the retina suggest that there is a delayed negative feedback in the off-pathway of the retina. In fact, a two neuron-model with delayed negative feedback can be shown to produce properties of an NGD filter. Presumably, such mechanism might also exist in a retina.

**References**

1. Chen KS, Chen CC, Chan CK. Characterization of predictive behavior of a retina by mutual information. Frontiers in Computational Neuroscience. 2017; 11: 66.
2. Voss HU. Signal prediction by anticipatory relaxation dynamics. Physical Review E. 2016; 93(3): 030201.

## P23 Closed-form approximations of single-channel calcium nanodomains in the presence of cooperative calcium buffers

### Victor Matveev, Yinbo Chen

#### New Jersey Institute of Technology, Department of Mathematical Sciences, Newark, United States of America

##### **Correspondence:** Victor Matveev (matveev@njit.edu)

*BMC Neuroscience* 2020, **21(Suppl 1)**:P23

Calcium ion (Ca2+) elevations produced in the vicinity of single open Ca2+ channels are termed Ca2+ nanodomains, and play an important role in triggering secretory vesicle exocytosis, myocyte contraction, and other fundamental physiological processes. Ca2+ nanodomains are shaped by the interplay between Ca2+ influx, Ca2+ diffusion and its binding to Ca2+ buffers, which absorb most of the Ca2+ entering the cell during a depolarization event. In qualitative studies of local Ca2+ signaling, the dependence of Ca2+ concentration on the distance from the Ca2+ channel source can be approximated with a reasonable accuracy by analytic approximations of quasi-stationary solutions of the corresponding reaction-diffusion equations. Such closed-form approximations help to reveal the qualitative dependence of nanodomain characteristics on Ca2+ buffering and diffusion parameters, without resorting to computationally expensive numerical simulations. Although a range of nanodomain approximations had been developed for the case of Ca2+ buffers with a single Ca2+ binding site, for example the Rapid Buffer Approximation, the Excess Buffer Approximation, and the Linear approximation [1,2], most biological buffers have more complex Ca2+-binding stoichiometry. Further, several important Ca2+ buffers and sensors such as calretinin and calmodulin consist of distinct EF-hand domains, each possessing two Ca2+ binding sites exhibiting significant cooperativity in binding, whereby the affinity of the second Ca2+ binding reaction is much higher compared to the first binding reaction. To date, only the Rapid Buffer Approximation (RBA) has been generalized to Ca2+ buffers with two binding sties [3]. However, the performance of RBA in the presence of cooperative Ca2+ buffers is limited by the complex interplay between the condition of slow diffusion implied by the RBA, and the slow rate of the first Ca2+ binding reaction characterizing cooperative Ca2+ binding. To resolve this problem, we present modified versions of several Ansatze recently introduced for the case of simple buffers [4], extending them to the case of Ca2+ buffers with 2-to-1 stoichiometry. These new approximants interpolate between the short-range and long-range distance-dependence of Ca2+ nanodomain concentration using a combination of rational and exponential functions. We examine in detail the parameter-dependence of the approximation accuracy, and show that this method is superior to RBA for a wide ranges of buffering parameter values. In particular, the new approximants accurately estimate the distance-dependence of Ca2+ concentration in the case of calretinin or calmodulin.

**Acknowledgements:** Supported in part by NSF DMS-1517085 (V.M).

**References**

1. Naraghi M, Neher E. Linearized buffered Ca2+ diffusion in microdomains and its implications for calculation of [Ca2+] at the mouth of a calcium channel. Journal of Neuroscience. 1997; 17: 6961- 6973.
2. Smith GD, Dai L, Miura RM, Sherman A. Asymptotic Analysis of Buffered Calcium Diffusion Near a Point Source. SIAM Journal on Applied Mathematics. 2001; 6: 1816-1838.
3. Matveev V. Extension of Rapid Buffering Approximation to Ca2+ Buffers with Two Binding Sites. Biophysical Journal. 2018; 114: 1204-1215.
4. Chen Y, Muratov C, Matveev V. Efficient approximations for stationary single-channel Ca2+ nanodomains across length scales. bioRxiv 2020.

## P24 A bump-attractor spiking neural network for motor adaptation and washout based on norepinephrine release in primary motor cortex

### Mariia Popova^1^, Melanie Tschiersch^1^, Nicolas Berberich^2^, Stefan K Ehrlich^2^, David Franklin^3^, Gordon Cheng^2^

#### ^1^Technical University of Munich, Department of Electrical and Computer Engineering, Munich, Germany; ^2^Technical University of Munich, Institute for Cognitive Systems, Munich, Germany; ^3^Technical University of Munich, Neuromuscular Diagnostics, Munich, Germany

##### **Correspondence:** Mariia Popova (maria.popova@tum.de)

*BMC Neuroscience* 2020, **21(Suppl 1)**:P24

In order to examine the formation of predictive motor memories, typical behavioural motor learning experiments perturb participants reaching movements using an external force field, to which they rapidly adapt, and exhibit after effects when the force field is removed. During the force field adaptation trials (Fig. 1), subjects move their hand from the start position (red circle) to the target position (black x) while a force field perturbs the movement (strength and direction indicated by yellow arrows) causing the hand to move to the final position (green circle). After the force field is removed, a washout effect is observed. While previous computational models can recreate the behavioral results, they do not account for the neural mechanisms involved. A computational model including a synaptic mechanism can help to explain the processes involved in motor learning. For this reason, we developed a bump-attractor, spiking neuron model of primary motor cortex (M1) proposing a synaptic mechanism using reward-based neurotransmitter release to explain motor adaptation and washout.

**Fig. 1:**
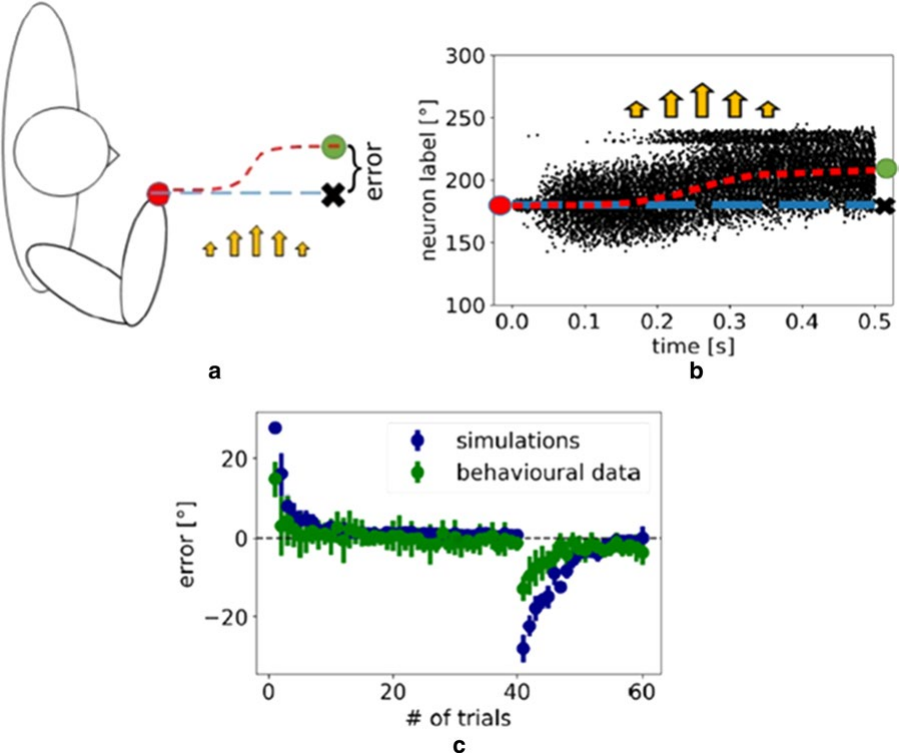
**a** Schematics of behavioural experiment. **b** Single trial simulation: Bump of neuronal activity in the first perturbed trial**. c** Adaptation results: Results of the simulations (blue) of error development over consecutive trials during adaptation and washout in comparison to behavioural data (green) from [1]

The developed model consists of directionally-tuned neurons, shown to exist in M1 in biology, that encode the hand position through average neural firing. The force field is modeled through a simulated, external current perturbing the neural activity in the direction of the force field. In biology, Norepinephrine is released from locus coreuleus to M1 when errors are detected in the visual pathway. Norepinephrine affects M1 in a goal-directed manner, increasing the excitatory synaptic responses in the so-called hotspot, which is determined by arousal. For the model to remain close to biology, adaptation is modeled through an error-dependent increase in excitatory to excitatory conductance in the target position within the M1 model, leading to a decrease of the perturbation on the stable bump of neural activity across trials. Washout is implemented through a shift of the hotspot and the accumulated Norepinephrine through a motor-coordinate system shift during force field removal. After the initial washout trial, the wrongful coordinate system shift is detected and Norepinephrine in the shifted hotspot decays.

The simulations from the proposed computational model qualitatively account for both: adaptation and washout as seen in comparison to the behavioural data from [1] (Fig. 1). Thus, the model suggests for the first time a biologically plausible synaptic mechanism in M1 that can explain the main features of motor learning of external dynamics.

**Reference**

1. Nozaki D, Kurtzer I, Scott S. Limited transfer of learning between unimanual and bimanual skills within the same limb. Nature Neuroscience. 2006; 9: 1364–1366.

## P25 Increased glutamate metabolism in ACC after brief mindfulness training: a pilot MR spectroscopy study

### Yiyuan Tang^1^, Pegah Askari^2^, Changho Choi^2^

#### ^1^Texas Tech University, Lubbock, Texas, United States of America; ^2^University of Texas Southwestern Medical Center, Advanced Imaging Research Center, Dallas, United States of America

##### **Correspondence:** Yiyuan Tang (yiyuan.tang@ttu.edu)

*BMC Neuroscience* 2020, **21(Suppl 1)**:P25

Mindfulness training (MT) involves paying attention to the present and increasing awareness of one’s thoughts and emotions without judgment and has become a promising intervention for promoting health and well-being. Neuroimaging studies have shown its beneficial effects on brain functional activity, connectivity, and structures [1-3]. A series of RCTs indicated that one form of MT, integrative body-mind training (IBMT) induces brain functional and structural changes in region related to self-control networks such as the anterior cingulate cortex (ACC) after 2-10 h of practice [1-3]. However, whether MT could change brain metabolism in the ACC remains unexplored. Utilizing a non-invasive proton magnetic resonance spectroscopy (MRS), we conducted the first pilot study investigating whether brief IBMT could change the excitatory and inhibitory responses of neurotransmitters within the ACC [1-3]. Nine healthy college students completed ten 1-hour IBMT sessions within 2-week and brain metabolism were assessed before and after using a 3T Siemens Prisma scanner. Following survey imaging and T1-weighted structural imaging, single-voxel point-resolved spectroscopy (PRESS) was conducted for estimating the metabolite concentrations in 2 regions - rostral and dorsal ACC based on prior literature [1-4]. PRESS scan parameters included TR 2s, TE 90 ms, sweep width 2.5 kHz, 1024 sampling points, and 256 signal averages. Water suppression and B0 shimming up to second order were performed with the vendor-supplied tools. Reference water signal was acquired for eddy current compensation, multi-channel combination, and metabolite quantification. Spectral fitting was performed with LCModel software [5], using in-house basis spectra of metabolites which were calculated incorporating the PRESS slice selective RF and gradient pulses. The spectral fitting was performed between 0.5-4.0 ppm. After correcting the LCModel estimates of metabolite signals for the T2 relaxation effects using published T2 values [6], the millimolar concentrations of metabolites were calculated with reference to water at 42 M. Paired t-tests were performed to examine changes. Results indicated a significant increase in glutamate metabolism (t = 3.24, p = 0.012), as well as Glx (glutamate + glutamine) (t = 2.44, p = 0.041) in the rostral ACC. Results indicate that MT may not only increase ACC activity, but also may induce neurochemical changes in regions of self-control networks, suggesting a potential mechanism of MT’s effects on disorders such as addiction and schizophrenia, which often involve the dysfunction of glutamatergic system (i.e. lower glutamate metabolism).

**References**

1. Tang YY, Holzel BK, Posner MI. The neuroscience of mindfulness meditation. Nature Reviews Neuroscience. 2015; 16: 213-22
2. Tang YY. The Neuroscience of Mindfulness Meditation: How the Body and Mind Work Together to Change Our Behavior? Springer Nature. 2017.
3. Tang YY, Tang R, Gross JJ. Promoting emotional well-being through an evidence-based mindfulness training program. Frontiers in Human Neuroscience. 2019;13: 237.
4. Yang S, et al. Lower glutamate levels in rostral anterior cingulate of chronic cocaine users - A (1)H-MRS study using TE-averaged PRESS at 3T. Psychiatry Research. 2009; 174: 171-6.
5. Provencher SW. Estimation of metabolite concentrations from localized in vivo proton NMR spectra. Magnetic Resonance in Medicine. 1993; 30: 672–679.
6. Ganji SK, et al. T2 measurement of J‐coupled metabolites in the human brain at 3T. NMR in Biomedicine. 2012; 25(4): 523-529.

## P26 Learning recurrent dynamic patterns in spiking neural networks

### Christopher Kim, Carson Chow

#### National Institutes of Health, Laboratory of Biological Modeling, NIDDK, Maryland, United States of America

##### **Correspondence:** Christopher Kim (chrismkkim@gmail.com)

*BMC Neuroscience* 2020, **21(Suppl 1)**:P26

Understanding the recurrent dynamics of cortical circuits engaged in complex tasks is one of the central questions in computational neuroscience. Most of recent studies train the output of recurrent models to per- form cognitive or motor tasks and investigate if the recurrent dynamics emerging from task-driven learning can explain neuronal data. However, the possible range of recurrent dynamics that can be realized within a recurrent model after learning, particularly in a spiking neural network, is not well understood. In this study, we focus on investigating spiking network’s capability to learn recurrent dynamics and characterize the learning capacity in terms of network size, intrinsic synaptic decay time and target decay time. We find that, by modifying recurrent synaptic weights, spiking networks can generate arbitrarily complex recurrent patterns if 1) the target patterns can be produced self-consistently, 2) the synaptic dynamics are fast enough to track the targets, and 3) the number of neurons in the network is large enough for noisy postsynaptic currents to approximate the targets. We examine spiking network’s learning capacity analytically and corroborate the predictions by training spiking networks to learn arbitrary patterns and in-vivo cortical activity. Furthermore, we show that a trained network can operate in balanced state if the total excitatory and inhibitory synaptic weights to each neuron are constrained to preserve the balanced network structure. Under such synaptic constraints, the trained network generates spikes at the desired rate with large trial-to-trial variability and exhibits paradoxical features of inhibition-stabilized network.

These results show that spiking neural networks with fast synapses and a large number of neurons can generate arbitrarily complex dynamics. When learning is not optimal, our findings can suggest potential sources of learning errors. Moreover, networks can be trained in dynamic regime relevant to cortical circuits.

## P27 Lessons from Artificial Neural Network for studying coding principles of Biological Neural Network

### Hyojin Bae^1^, Chang-eop Kim^2^, Gehoon Chung^3^

#### ^1^Gachon university, Gyeonggi-do, South Korea; ^2^Gachon university, College of Korean Medicine, Gyeonggi-do, South Korea; ^3^Seoul National University, Oral Physiology, Seoul, South Korea

##### **Correspondence:** Hyojin Bae (qogywls1573@gmail.com)

*BMC Neuroscience* 2020, **21(Suppl 1)**:P27

An individual neuron or neuronal population is conventionally said to be “selective” to a feature of stimulus if they differentially respond to the feature. Also, they are considered to encode certain information if decoding algorithms successfully predict a given stimulus or behavior from the neuronal activity. However, an erroneous assumption about the feature space could mislead the researcher about a neural coding principle. In this study, by simulating several likely scenarios through artificial neural networks (ANNs) and showing corresponding cases of biological neural networks (BNNs), we point out potential biases evoked by unrecognized features i.e., confounding variable.

We modeled an ANN classifier with the open-source neural network library Keras, running Tensorflow as backend. The model is composed of five hidden layers, dense connections and rectified linear activation. We added a dropout layer and l2-regularizer on each layer to apply penalties on layer activity during optimization. The model was trained with CIFAR-10 dataset and showed a saturated test set accuracy at about 53% (the chance level accuracy = 10%). For a stochastic sampling of individual neuron’s activity from each deterministic unit, we generated the Gaussian distribution through modeling within-population variability according to each assumption.

Using this model, we showed 4 possible misinterpretation cases induced by a missing feature. (1) The researcher can choose the second-best feature which has similarity to ground truth feature. (2) An irrelative feature which correlated with ground truth feature can be chosen. (3) Evaluating decoder in incomplete feature space could result in the overestimation of the performance of the decoder. (4) Misconception about the receptive field of the unit could make a signal to be incorporated in noise.

In conclusion, we suggest that the comparative study of ANN and BNN from the perspective of machine learning can be a great strategy for deciphering the neural coding principle.

## P28 Inhibitory gain allows transitions between integrated and segregated states: a neuromodulatory analysis from whole-brain models

### Carlos Coronel^1^, Patricio Orio^2^, Rodrigo Cofré^3^

#### ^1^Universidad de Valparaíso, Centro Interdisciplinario de Neurociencia de Valparaíso, Valparaíso, Chile; ^2^Universidad de Valparaíso, Instituto de Neurociencia, Valparaiso, Chile; ^3^Universidad de Valparaíso, Centro de Investigación y Modelamiento de Fenómenos Aleatorios, Valparaíso, Chile

##### **Correspondence:** Carlos Coronel (carlos.coronel@postgrado.uv.cl)

*BMC Neuroscience* 2020, **21(Suppl 1)**:P28

In the brain, at the macroscale level, two organizational principles participate in the processing of information: segregation and integration. While segregation allows the processing of information in specific brain regions, integration coordinates the activity of these regions to generate a behavioral response [1]. Recent studies suggest that the cholinergic system promotes segregated states and the noradrenergic system promotes integrated states, both measured using graph theoretical tools over the functional connectivity (FC) matrices [2,3]. We extended this neuromodulatory framework by including the noradrenergic system (filter gain), and the effect of the cholinergic system in the excitatory and inhibitory circuits separately (excitatory and inhibitory gain). The neuromodulatory framework was tested using the Jansen Rit neural mass model [4], built from real human structural connectivity matrices and heterogeneous transmission delays for long-range connections (Fig. 1). The fMRI-BOLD signals were simulated using a generalized hemodynamic function model, and features such as the global phase synchronization, oscillatory frequency and SNR were measured. On the other hand, FC matrices were built using pairwise Pearson’s correlation from the simulated BOLD signals. Thresholded FC matrices were analyzed with graph theoretical tools for computing segregation and integration. Our results suggest that functional integration is possible only with the suppression of the feedback excitation, mediated by the inhibitory gain, and follows a sigmoid or inverted U-shaped function, depending of the noise intensity levels. Also, the integration is accompanied by an increase in signal to noise ratio and regularity of EEG signals. The results suggest a mechanistic interpretation. We propose that the cholinergic system neuromodulation on the excitatory connections increases SNR locally, and the effect of that system on the inhibitory interneurons suppresses the local cortico-cortical transmission, increasing the responsivity of pyramidal neurons to stimulus from distant regions. Finally, the noradrenergic system coordinates long-range neural activity promoting integration. This framework constitutes a new set of tools and ideas to test how neural gain mechanisms mediate the balance between integration and segregation in the brain.

**Fig. 1:**
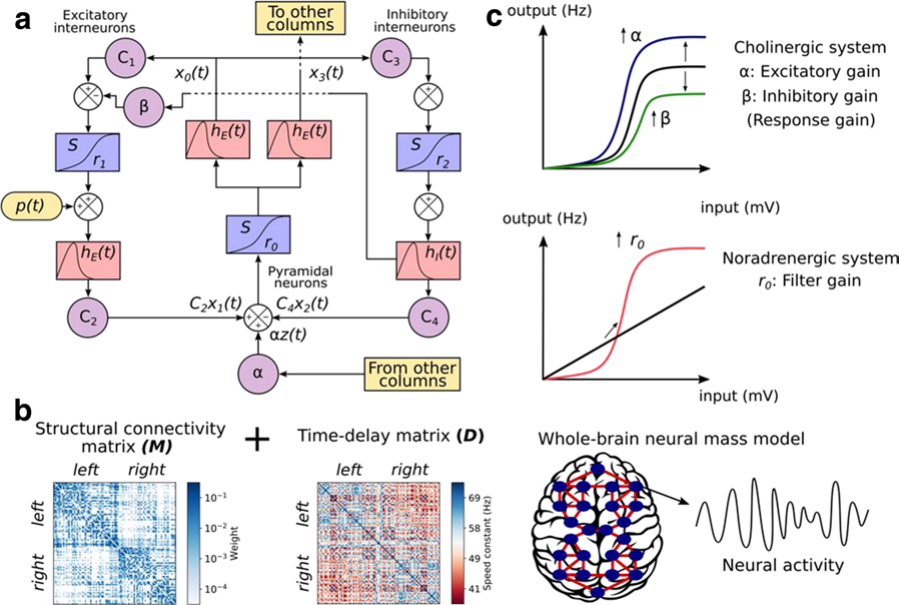
Whole-brain neural mass model. **A** The model consists in a population of pyramidal neurons, and two populations of excitatory and inhibitory interneurons. **B** The N = 90 cortical columns are connected by real human structural connectivity matrices, with heterogeneous time-delays. **C** The cholinergic system operates with the parameters α and β, and the noradrenergic system with the parameter r0

**References**

1. Rubinov M, Sporns O. Complex network measures of brain connectivity: uses and interpretations. Neuroimage. 2010*; 52(3)*: 1059-1069.
2. Shine JM, Aburn MJ, Breakspear M, Poldrack RA. The modulation of neural gain facilitates a transition between functional segregation and integration in the brain. Elife. 2018*; 7*: e31130.
3. Shine JM. Neuromodulatory influences on integration and segregation in the brain. Trends in Cognitive Sciences. 2019.
4. Jansen BH, Rit VG. Electroencephalogram and visual evoked potential generation in a mathematical model of coupled cortical columns. Biological Cybernetics. 1995*; 73(4)*: 357-366.

## P29 High-order interdependencies and the functional balance of segregation-integration in the aging brain

### Marilyn Gatica^1^, Rodrigo Cofre^2^, Fernando Rosas^3^, Pedro Mediano^4^, Patricio Orio^5^, Ibai Diez^6^, Stephan Swinnen^7^, Jesus Cortes^8^

#### ^1^Universidad de Valparaíso and Biomedical Research Doctorate Program UPV Spain, CINV, Faculty of Sciences, Valparaíso, Chile; ^2^Universidad de Valparaiso, Institute of Mathematical Engineering, Valparaiso, Chile; ^3^Imperial College London, Department of Medicine, London, United Kingdom; ^4^Cambridge University, Department of Psychology, Cambridge, United Kingdom; ^5^Universidad de Valparaíso, Instituto de Neurociencia, Valparaiso, Chile; ^6^Gordon Center for Medical Imaging, Harvard Medical School, Department of Radiology, Boston, United States of America; ^7^KU Leuven, Research Center for Movement Control and Neuroplasticity, Department of Movement Sciences, Leuven, Belgium; ^8^Computational Neuroimaging Lab, IKERBASQUE: The Basque Foundation for Science, Biocruces Health Research Institute, Bilbao, Spain

##### **Correspondence:** Rodrigo Cofre (rodrigocofre@gmail.com)

*BMC Neuroscience* 2020, **21(Suppl 1)**:P29

The interdependencies in the brain can be studied either from a structural/anatomical perspective (“structural connectivity”, SC) or by considering statistical interdependencies (“functional connectivity”, FC). While the SC is essentially pairwise (white-matter fibers start in a certain region and arrive at another), the FC is not, i.e., there is no reason to consider statistical interdependencies pairwise. A promising tool to study high-order interdependencies is the recently proposed O-Information [1]. This quantity captures the balance between redundancies and synergies in arbitrary sets of variables, thus extending the properties of the interaction information of three variables to larger sets. Redundancy is here understood as an extension of the conventional notion of correlation to more than two variables In contrast, synergy corresponds to an emergent statistical relationships that control the whole but not the parts.

In this study, we follow the seminal ideas introduced by Tononi, Sporns, and Edelman [2], which state that high brain functions might depend on the co-existence of integration and segregation. While the latter enables brain areas to perform specialized tasks independently of each other, the former serves to bind together brain areas towards an integrated whole for the purpose of goal-directed task performance. A key insight put forward in [2] is that segregation and integration can coexist and that this coexistence is measurable by assessing the high-order interactions of neural elements. We used the O-Information to investigate how high-order statistical interdependencies are affected by aging. For this, we analyzed fMRI data at rest from 164 healthy participants, ranging from 10 to 80 years old. Our results show an important increase in redundant interdependencies in the older population (age ranging from 60 to 80 years). Moreover, this effect seems to be pervasive, taking place at all interaction orders, suggesting a change in the balance of differentiation and integration towards more synchronized arrangements. Additionally, a redundant core of brain modules was observed, which decreased in size with age. The framework presented here and in detail in [3], provide novel insights into the aging brain revealing the role of redundancy in prefrontal and motor cortices in older participants, thus affecting basic functions such as working memory, executive and motor functions. This methodology may help to provide a better understanding of some brain disorders from an informational perspective, providing “info-markers”, that may lead to fundamental insights into the human brain in health and disease. The code to compute the metrics is available at [4].

**Fig. 1:**
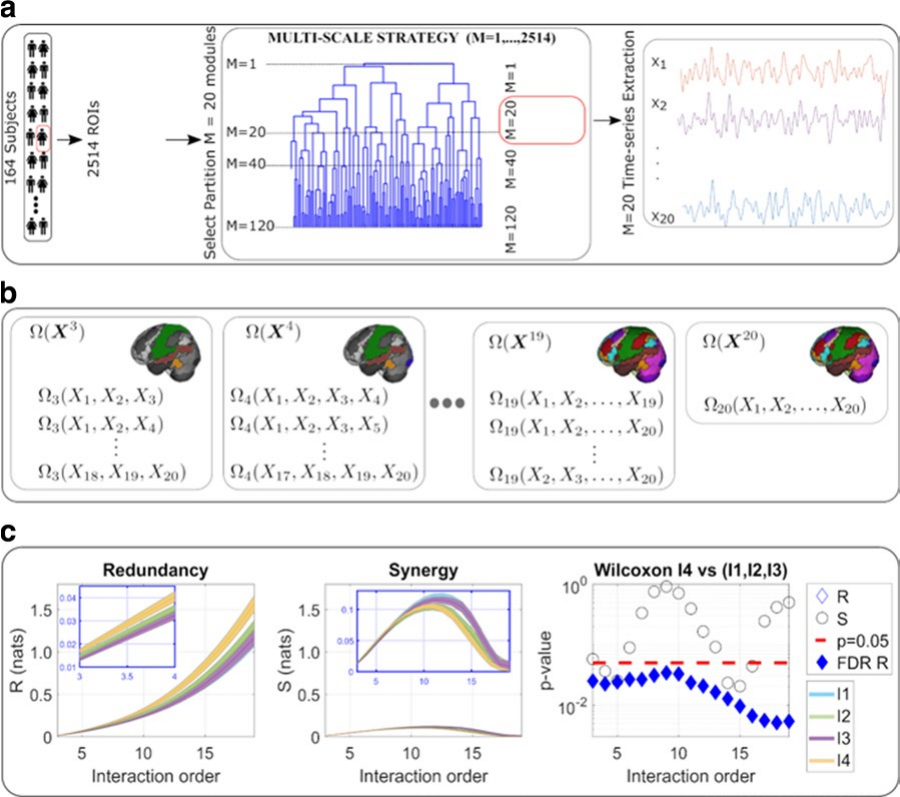
Method overview and main result. **A** 164 subjects, 2514 fMRI signals parcellated into 20 regions. **B** For each subject and interaction order we computed for each n-plet their O-Information. **C** Average over all 20 modules of redundancy and synergy for each of the age groups I1 (younger), I2, I3, I4 (oldest). The right panel shows that group differences in redundancy (represented by diamonds) using FDR

**References**

1. Rosas F, Mediano Pedro AM, Gastpar M, and Jensen HJ. Quantifying High-order Interdependencies via Multivariate Extensions of the Mutual Information. Physical Review E. 2019; 100: 032305.
2. Tononi G, Sporns O, Edelman GM. A measure for brain complexity: relating functional segregation and integration in the nervous system. Proceedings of the National Academy of Sciences. 1994; 91(11):5033-5037.
3. Gatica M, et al. High-order interdependencies in the aging brain. bioRxiv. 2020.
4. https://github.com/brincolab/High-Order-interactions.

## P30 Biophysically realistic model of mouse olfactory bulb gamma fingerprint

### Justas Birgiolas^1^, Richard Gerkin^2^, Sharon Crook^3^

#### ^1^Arizona State University, Arizona, United States of America; ^2^Arizona State University, School of Life Sciences, Tempe, Arizona, United States of America; ^3^Arizona State University, School of Mathematical and Statistical Sciences, Tempe, Arizona, United States of America

##### **Correspondence:** Justas Birgiolas (jbirgio@gmail.com)

*BMC Neuroscience* 2020, **21(Suppl 1)**:P30

The mammalian olfactory bulb is an intensively investigated system that is important in understanding neurodegenerative diseases. Insights gained from understanding the system also have important agricultural and national security applications. In this work, we developed a large-scale, biophysically, and geometrically realistic model of the mouse olfactory bulb and the gamma frequency oscillations [1] it exhibits. Model code, documentation, and tutorials are available atolfactorybulb.org.

The model consists of realistic mitral, tufted (excitatory), and granule (inhibitory) cell models whose electrophysiology and reconstructed morphology have been validated against experimental data using a suite of NeuronUnit [2] validation tests. The cell models were realistically placed, oriented, and confined within anatomically correct mouse olfactory bulb layers obtained from the Allen Brain Atlas [3] using features of BlenderNEURON software [4]. Dendritic proximity was used to form chemical and electrical synapses between principal and inhibitory cell dendrites. Glomeruli were stimulated using simulated odors obtained from optical imaging experiments. The local field potentials generated by the network were monitored and processed using wavelet analysis to replicate a gamma frequency pattern (fingerprint) consisting of an early-high, and later-low frequency temporal components. Simulations were performed using parallel-NEURON [5].

The network was subjected to computational manipulations, which revealed the critical importance of gap junctions, granule cell inhibition, and input strength differences between mitral and tufted cells in generating the gamma fingerprint. Specifically, at glomerular level, gap junctions synchronize the firing of mitral cell and tufted cell populations. Synchronized tufted cells activate granule cells, which inhibit mitral cells. Meanwhile, reduced afferent excitatory input results in mitral cell activation delay, which is amplified by tufted cell activated granule cell inhibition. The interaction between these three mechanisms results two clusters of activity seen in the gamma fingerprint (Fig. 1).

**Fig. 1:**
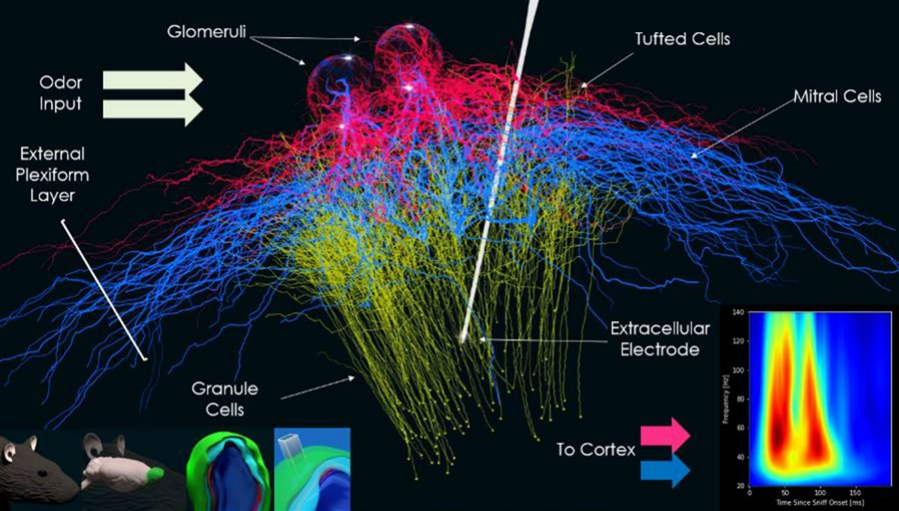
The network model consists of biophysically realistic mitral, tufted, and granule cell models, which are positioned within reconstructed mouse olfactory bulb layers. The network is activated by simulated glomerular inputs. Local field potentials generated by the network are measured using an extracellular electrode. The network produces a two-cluster gamma fingerprint (lower right)

The results of the computational experiments support mechanistic hypotheses proposed in earlier experimental work and provide novel insights into the mechanisms responsible for olfactory bulb gamma fingerprint generation, which can be directly tested using common experimental preparations.

**Acknowledgments**: This work was funded in part by the National Institutes of Health through 1F31DC016811 to JB, R01MH1006674 to SMC, and R01EB021711 to RCG.

**References**

1. Manabe H, Mori K. Sniff rhythm-paced fast and slow gamma-oscillations in the olfactory bulb: Relation to tufted and mitral cells and behavioral states. Journal of Neurophysiology. 2013; 110(7): 1593–1599.
2. Gerkin RC, Birgiolas J, Jarvis RJ, Omar C, Crook SM. NeuronUnit: A package for data-driven validation of neuron models using SciUnit. bioRxiv. 2019; 665331.
3. Oh S, et al. A mesoscale connectome of the mouse brain. Nature. 2014; 508(7495): 207–214.
4. Birgiolas J. Towards Brains in the Cloud: A Biophysically Realistic Computational Model of Olfactory Bulb *[Doctoral dissertation, Arizona State University].*
5. Hines M, Carnevale N. The NEURON Simulation Environment. Neural Computation. 1997; 9(6): 1179–1209.

## P31 Exploring fast and slow neural correlates of auditory perceptual bistability with diffusion-mapped delay coordinates

### Pake Melland, Rodica Curtu

#### University of Iowa, Department of Mathematics, Iowa City, Iowa, United States of America

##### **Correspondence:** Rodica Curtu (rodica-curtu@uiowa.edu)

*BMC Neuroscience* 2020, **21(Suppl 1)**:P31

Perceptual bistability is a phenomenon in which an observer is capable of perceiving identical stimuli with two or more interpretations. The auditory streaming task has been shown to produce spontaneous switching between two perceptual states [1]. In this task a listener is presented a stream of tones, called triplets, with the pattern ABA– where A and B are tones with different frequencies and ‘–’ is a brief period of silence. The listener can alternate between two perceptual states: 1-stream in which the stimulus is integrated into a single stream, and 2-stream in which the stimulus is perceived as two segregated streams. In order to study the localization and dynamic properties of neural correlates of auditory streaming we collected electrocorticography (ECoG) data from neurosurgical patients while they listened to sequences of repeated triplets and self-reported switching between the two perceptual states.

It is necessary to find meaningful ways to analyze ECoG recordings, which are noisy and inherently high dimensional. Diffusion Maps is a non-linear dimensionality reduction technique which embeds high dimensional data into low dimensional Euclidean space [2]. The Diffusion Map method leverages the creation of a Markov matrix from a similarity measure on the original data. Under reasonable assumptions, the eigenvalues of the Markov matrix are positive and bounded above by 1. The largest eigenvalues along with their respective eigenvectors provide coordinates for an embedding of the data into d-dimensional Euclidean space. In [3] Diffusion Maps were used for a group level analysis of neural signatures during auditory streaming based on subject reported perception. We extend this approach by taking into account the time ordered property of the ECoG signals. For data that has a natural time ordering, it is beneficial to structure the data to emphasize its temporal dynamics; in [4] the authors develop the Diffusion- Mapped Delayed Coordinates (DMDC) algorithm. In this algorithm, time-delayed data is first created from general time series data; this initial step projects the data onto its most stable sub-system. The stable sub-system may remain in a high dimensional space, so they next apply Diffusion Maps to the time-delayed data which projects the (potentially high dimensional) stable sub-system onto a low dimensional representation adapted to the dynamics of the system.

We apply the DMDC algorithm to ECoG recordings from Heschl’s Gyrus in order to explore and reconstruct the underlying dynamics present during the auditory steaming task. We find that the eigenvalues obtained through the DMDC algorithm provide a way to uncover multiple time scales present in the underlying system. The corresponding eigenvectors form a Fourier-like basis that is adapted both to the fast properties of ECoG signal encoding the physical properties of the stimulus as well as a slow mechanism that corresponds to perceptual switching reported by subjects.

**Acknowledgments:** National Science Foundation, CRCNS grant 1515678, and The Human Brain Research Lab, University of Iowa, Iowa (Matthew A. Howard & Kirill Nourski).

**References**

1. Noorden LP. Temporal coherence in the perception of tone sequences *[Doctoral dissertation, Eindhoven]*.
2. Coifman RR, Lafon S. Diffusion maps. Applied and Computational Harmonic Analysis. 2006; 21(1): 5-30.
3. Curtu R, Wang X, Brunton BW, Nourski KV. Neural signatures of auditory perceptual bistability revealed by large-scale human intracranial recordings. Journal of Neuroscience. 2019; 39(33): 6482-97.
4. Berry T, Cressman JR, Greguric-Ferencek Z, Sauer T. Time-scale separation from diffusion-mapped delay coordinates. SIAM Journal on Applied Dynamical Systems. 2013; 12(2): 618-49.

## P32 Contribution of the Na/K pump to rhythmic bursting.

### Ronald Calabrese^1^, Ricardo J E Toscano^2^, Parker J Ellingson^2^, Gennady Cymbalyuk^2^

#### ^1^Emory University, Department of Biology, Atlanta, Georgia, United States of America; ^2^Georgia State University, Neuroscience Institute, Atlanta, Georgia, United States of America

##### **Correspondence:** Ronald Calabrese (biolrc@emory.edu)

*BMC Neuroscience* 2020, **21(Suppl 1)**:P32

The Na/K pump, often thought of as a background function in neuronal activity, contributes an outward current (IPump) that responds to the internal concentration of Na+([Na+]i). In bursting neurons, such as those found in central pattern generators (CPGs) that produce rhythmic movements, one can expect the [Na+]i and thus IPump to vary throughout the burst cycle [1-3]. This variation with electrical activity and the independence from membrane potential endow IPump with dynamical properties not available in channel-based currents (e.g. voltage- or transmitter- gated, or leak channels). Moreover, in many neurons the pump’s activity is modulated by a variety of modulators further expanding the potential role of IPump in rhythmic bursting activity [4]. Using a combination of experiment, modeling, and hybrid systems analyses, we have sought to determine how IPump and its modulation influence rhythmic activity in a CPG.

**References**

1. Rybak IA, Molkov YI, Jasinski PE, Shevtsova NA, Smith JC. Rhythmic bursting in the pre-Bötzinger complex: mechanisms and models. Progress in Brain Research. 2014; 209: 1-23.
2. Kueh D, Barnett WH, Cymbalyuk GS, Calabrese RL. Na(+)/K(+) pump interacts with the h-current to control bursting activity in central pattern generator neurons of leeches. Elife. 2016; 5: e19322.
3. Picton LD, Nascimento F, Broadhead MJ, Sillar KT, Miles GB. Sodium Pumps Mediate Activity-Dependent Changes in Mammalian Motor Networks. Journal of Neuroscience. 2017; 37(4): 906-921.
4. Tobin AE, Calabrese RL. Myomodulin increases Ih and inhibits the NA/K pump to modulate bursting in leech heart interneurons. Journal of Neurophysiology. 2005; 94(6): 3938-50.

## P33 Temperature coding mechanisms in the cold-sensitive Drosophila neurons

### Natalia Maksymchuk^1^, Akira Sakurai^1^, Jamin M Letcher^1^, Daniel N Cox^1^, Gennady Cymbalyuk^1^

#### ^1^Georgia State University, Neuroscience Institute, Atlanta, Georgia, United States of America

##### **Correspondence:** Gennady Cymbalyuk (gcymbalyuk@gmail.com)

*BMC Neuroscience* 2020, **21(Suppl 1)**:P33

Sensation of noxious cold stimuli plays an essential role in the survival of organisms. Adequate perception of certain characteristics of cold stimuli is necessary for appropriate behavioral responses for body protection. There exist primary cold-sensing neurons that encode distinct information about the cold thermal stimulus: the rate of temperature decrease and absolute temperature coding. Here, we focus on the roles of different ion channels in the cold temperature coding of the *Drosophila* larva. We investigate dynamics of the Class III (CIII) somatosensory neurons. They trigger a stereotypic cold-evoked behavior, a full-body contraction (CT). We combined computational neuroscience, genetic, and electrophysiological methods to develop a biophysical model of CIII neurons. Our computational model includes ionic currents implicated by transcriptomic data of ion channels expression in CIII neurons. We implement these currents using the gating characteristics of *Drosophila* Na+ and K+ channels obtained from the experimental literature using patch-clamp data [1,2]. We consider three subsystems (1) fast spike-generating subsystem, (2) moderately slow pattern-generating subsystem, and (3) slow thermotransduction subsystem. We investigated the role of these subsystems in temperature coding. Using the slow-fast decomposition approach, we isolated the fast spike-generating subsystem (a CIII reduced model). We systematically varied two parameters, the temperature (T) and Gleak, classified observed regimes of activity and mapped them on the plane (T,Gleak). The model exhibits a wide spectrum of regimes: spiking, bursting, and silenced. Analysis of a model including a moderately slow pattern-generating subsystem unveiled a slow bursting activity pattern of the CIII model. This regime requires the participation of Ca2+-activated K+ currents at noxious cold temperatures and an increased level of intracellular Ca2+. Similar burst and pause activity pattern were implicated in encoding noxious heat stimuli by the heat-sensitive Drosophila CIV neurons. We investigated a full model including the TRP channels representing the slow thermotransduction subsystem. We show that they play the key roles in coding of noxious cold temperature and encoding rate of temperature decrease. Our model qualitatively reproduces the temporal properties of the recordings from Trpm, Pkd2, and TRPA1 knock-down animals. In agreement with these experimental results, the corresponding models of TRP mutant CIII neurons [1] show decreased averaged cold-evoked firing rate and [2] diminished peak of firing rate in response to fast temperature change relative to controls. Studying the role of ion channels subsystems helped us classify possible regimes of activity and transitions between them, understand the complex dynamics of the cold-sensing neuron and putative mechanisms of encoding sensory information.

**Acknowledgments:** This research was supported by NIH grants 1R01NS115209 (DNC and GC) and 2R01NS086082 (DNC) and GSU B&B Grant (DNC).

**References**

1. Wang L, Nomura Y, Du Y, Dong K. Differential effects of TipE and a TipE-homologous protein on modulation of gating properties of sodium channels from Drosophila melanogaster. PLoS One. 2013; 8(7): e67551.
2. Hardie RC. Voltage-sensitive potassium channels in Drosophila photoreceptors. Journal of Neuroscience. 1991; 11(10): 3079-3095.

## P34 Atlas-mapped reconstruction of the cerebellar Lingula

### Alice Geminiani^1^, Dimitri Rodarie^2^, Robin De Schepper^3^, Claudia Casellato^4^, Egidio D’Angelo^3^

#### ^1^University of Pavia, Pavia, Italy; ^2^École polytechnique fédérale de Lausanne, Blue Brain Project, Geneva, Switzerland; ^3^University of Pavia, Department of Brain and Behavioral Sciences, Pavia, Italy; ^4^University of Pavia, Department of Brain and Behavioral Sciences, Unit of Neurophysiology, Pavia, Italy

##### **Correspondence:** Alice Geminiani (alice.geminiani@unipv.it)

*BMC Neuroscience* 2020, **21(Suppl 1)**:P34

Large-scale neuronal networks are a powerful tool to investigate brain area functionality, embedding realistic temporal dynamics of neurons, synapses and microcircuits. However, spatial features differentiating regions within the same brain area are crucial for proper functioning of interconnected networks. In case of cerebellum, input signals are mapped and integrated in different regions with specific structural properties and local network dynamics [1].

Here we describe the reconstruction of the mouse Lingula (region of cerebellar Vermis) mapped on data from the Allen Brain Atlas as in [2], including neuron densities and orientation vectors in cubic voxels (25um side).

Network reconstruction was based on strategies of the cerebellar scaffold [3], with new features for folded volumes. The main cerebellar neurons were placed in the 3 layers of the cerebellar cortex, i.e. Granule and Golgi cells (GrC and GoC) in the Granular layer, Purkinje cells (PC) in the Purkinje layer, and Basket and Stellate cells in the Molecular layer. A particle placement strategy was used for Granular and Molecular neurons, in subvolumes made up of 50 voxels each. The algorithm included: placing the total number of neurons expected in each subvolume at random positions as repellent particles; iteratively re-positioning detected colliding particles within the subvolume; pruning particles placed outside. An adhoc algorithm was developed for PCs: in each parasagittal section, they were placed in parallel arrays at a minimum distance of 130um to avoid dendritic tree overlap, using A* search algorithm to find adjacent PC.

Orientation data were used for connectivity: each cell morphology was rotated based on orientation of the voxel containing the soma; connections were identified through an efficient algorithm searching for intersections of discretized rotated morphologies volumes. For connections from GrCs, parallel fibers were also bended to follow the orientation field. The resulting network included about 105 neurons and 107 connections (Fig. 1).

**Fig. 1:**
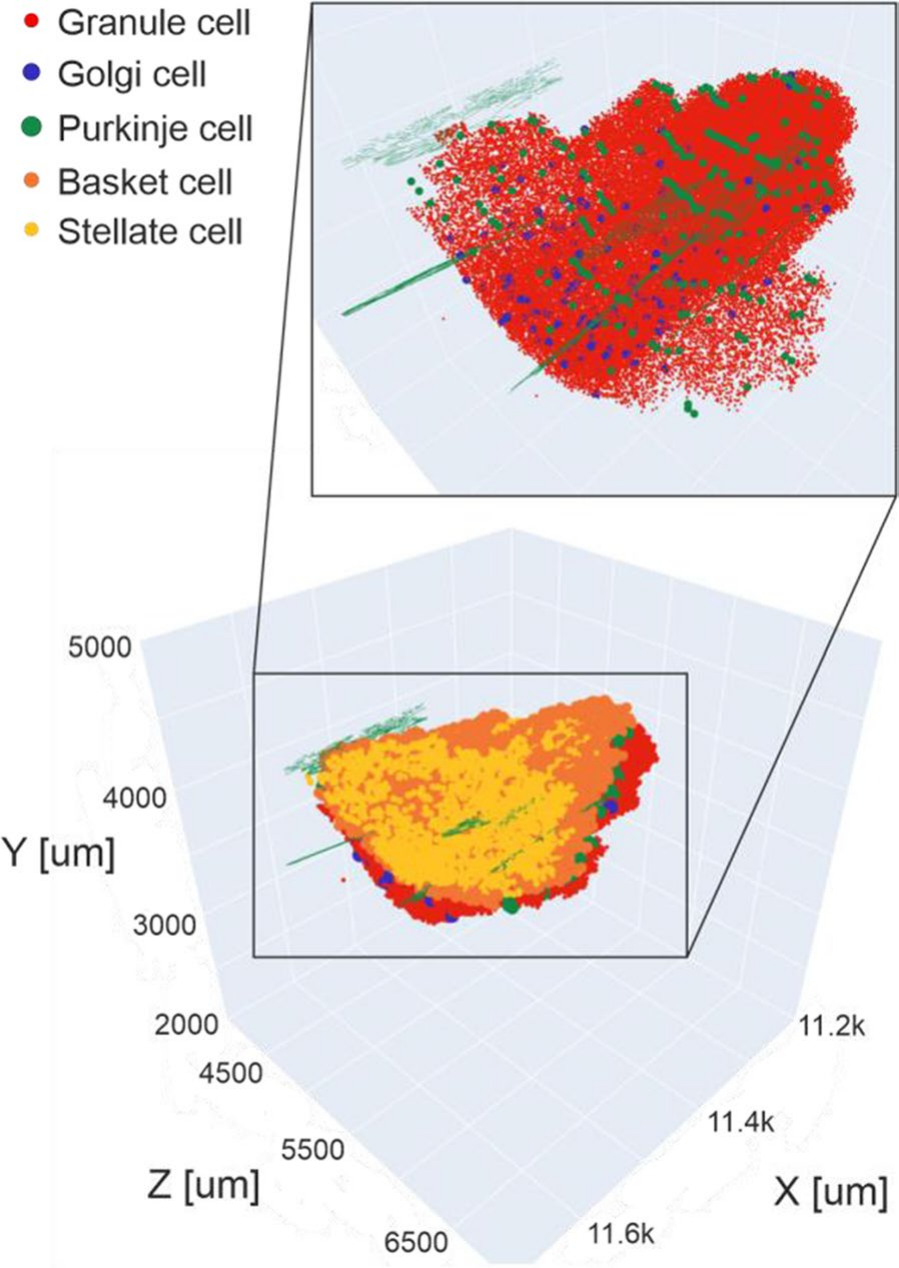
Mapped reconstruction of the Lingula with the main cerebellar neurons. Rotated morphologies are reported for some PCs. The inset panel shows more in detail the parallel arrays of PCs with rotated sample morphologies

This proposed pipeline will be generalized to other cerebellar regions up to reconstruction of a full mouse cerebellum. The network, filled with point or detailed neuron/synapse models, will be used to investigate spatial features of signal propagation in the cerebellum, and the specialization and integration of sensory signals across different regions [4]. This will allow reproducing spatially-mapped experimental data, e.g. Local Field Potentials, and embedding the cerebellum in whole-brain frameworks built on atlases, in which multiple interconnected brain areas can be simulated even using different descriptions for each region (e.g. hybrid models with spiking circuits and mean-field components).

The workflow, here developed for the cerebellum, could be complementary to other algorithms, and applied to other brain areas.

**Acknowledgements:** This work has received funding from European Union’s Horizon 2020 Framework Programme for Research and Innovation under Grant Agreement N. 785907 (Human Brain Project SGA2) and SGA3.

**References**

1. Casali S, Marenzi E, Medini C, Casellato C, D’Angelo E. Reconstruction and simulation of a scaffold model of the cerebellar network. Frontiers in Neuroinformatics. 2019; 13: 37.
2. Erö C, Gewaltig MO, Keller D, Markram H. A cell atlas for the mouse brain. Frontiers in Neuroinformatics. 2018; 12: 84.
3. De Schepper, et al. A scaffold model for the cerebellum. *[in prep -https://github.com/dbbs-lab/scaffold]*
4. Apps R, et al. Cerebellar modules and their role as operational cerebellar processing units. The Cerebellum. 2018; 17(5): 654-82.

## P35 Through synapses to spatial memory maps: a topological model

### Yuri Dabaghian

#### The University of Texas McGovern Medical School, Neurology, Houston, Texas, United States of America

##### **Correspondence:** Yuri Dabaghian (yuri.a.dabaghian@uth.tmc.edu)

*BMC Neuroscience* 2020, **21(Suppl 1)**:P35

Learning and memory are fundamentally collective phenomena, brought into existence by highly organized spiking activity of large ensembles of cells. Yet, linking the characteristics of the individual neurons and synapses to the properties of large-scale cognitive representations remains a challenge: we lack conceptual approaches for connecting the neuronal inputs and outputs to the integrated results at the ensemble level. For example, numerous experiments point out that weakening of the synapses correlates with weakening of memory and learning abilities—but how exactly does it happen? If, for example, the synaptic strengths decrease on average by 5%, then will the time required to learn a particular navigation task increase by 1%, by 5% or by 50%? How would the changes in learning capacity depend on the original cognitive state? Can an increase in learning time, caused by a synaptic depletion, be compensated by increasing the population of active neurons or by elevating their spiking rates? Answering these questions requires a theoretical framework that connects the individual cell outputs and the large-scale cognitive phenomena that emerge at the ensemble level.

We propose a modeling approach that allows bridging the “semantic gap” between electrophysiological parameters of neuronal activity and the characteristics of spatial learning, using techniques from algebraic topology. Specifically, we study influence of synaptic transmission probability and the effects of synaptic plasticity on the hippocampal network’s ability to produce a topological cognitive map of the ambient space. We simulate deterioration of spatial learning capacity as a function of synaptic depletion in the hippocampal network to get a better insight into the spatial learning deficits (as observed, e.g., in Alzheimer’s disease) and understanding why development of these deficits may correlate with changes in the number of spiking neurons and/or of their firing rates, variations in the “brain wave” frequency spectra, etc. The results shed light on the principles of spatial learning in plastic networks and may help our understanding of neurodegenerative conditions.

## P36 Robust spatial memories encoded by transient neuronal networks

### Yuri Dabaghian

#### The University of Texas McGovern Medical School at Houston, Neurology, Houston, Texas, United States of America

##### **Correspondence:** Yuri Dabaghian (yuri.a.dabaghian@uth.tmc.edu)

*BMC Neuroscience* 2020, **21(Suppl 1)**:P36

The principal cells in mammalian hippocampus encode an internalized representation of the environment - the hippocampal cognitive map, that underlies spatial memory and spatial awareness. However, the synaptic architecture of the hippocampal network is dynamic: it contains a transient population of “cell assemblies”- functional units of the hippocampal computations - that emerge among the groups of coactive neurons and may disband due to reduction or cessation of spiking activity, then reappear, then disband again, etc. Electrophysiological studies in rats and mice suggest that the characteristic lifetimes of typical hippocampal cell assemblies range between minutes to tens of milliseconds. In contrast, cognitive representations sustained by the hippocampal network can last in rodents for months, which raises a principal question: how can a stable large-scale representation of space emerge from a rapidly rewiring neuronal stratum? We propose a computational approach to answering this question based on Algebraic Topology techniques and ideas. By simulating the place cell spiking activity during the rat’s exploratory movements through different environments and testing the stability of the resulting large-scale neuronal maps, we find that the networks with “flickering” architectures can reliably capture the topology of the ambient spaces. Moreover, the model suggests that the information is processed at three principal timescales, which roughly correspond to the short term, intermediate term and the long-term memories. The rapid rewiring of the local network connections occurs at the fastest timescale. The timescale at which the large-scale structures defining the shape of the cognitive map may fluctuate is by about an order of magnitude slower than the timescale of the information processing at the synaptic level. Lastly, an emerging stable topological base provides lasting, qualitative information about the environment, which remains robust despite the ongoing transience of the local connections.

## P37 A realistic spatial model of the complete synaptic vesicle cycle

### Andrew Gallimore, Iain Hepburn, Erik De Schutter

#### Okinawa Institute of Science and Technology, Computational Neuroscience Unit, Onna-son, Japan

##### **Correspondence:** Andrew Gallimore (gallimore@cantab.net)

*BMC Neuroscience* 2020, **21(Suppl 1)**:P37

The release of neurotransmitters from synaptic vesicles is the fundamental mechanism of information transmission between neurons in the brain. The entire synaptic vesicle cycle involves a highly complex interplay of proteins that direct vesicle docking at the active zone, the detection of intracellular calcium levels, fusion with the presynaptic membrane, and the subsequent retrieval of the vesicle protein material for recycling [1]. Despite its central importance in many aspects of neuronal function, and even though computational models of subcellular neuronal processes are becoming increasingly important in neuroscience research, realistic models of the synaptic vesicular cycle are almost non-existent. This is largely because the modeling tools for detailed spatial modeling of vesicles are not available.

Extending the STEPS simulator [2], we have pioneered spherical ‘vesicle’ objects that occupy a unique excluded volume and sweep a path through the tetrahedral mesh as they diffuse through the cytosol. Our vesicles incorporate endo- and exocytosis, fusion with and budding from intracellular membranes, neurotransmitter packing, as well as interactions between vesicular proteins and cytosolic and plasma membrane proteins. This allows us to model all key aspects of the synaptic vesicle cycle, including docking, priming, calcium detection and vesicle fusion, as well as dynamin-mediated vesicle retrieval and recycling.

Using quantitative measurements of protein copy numbers [3], membrane and cytosolic diffusion rates, protein-protein interactions, and an EM-derived spatial model of a hippocampal pyramidal neuron, we used this technology to construct the complete synaptic vesicle cycle at the Schaffer Collateral–CA1 synapse at an unprecedented level of spatially-realistic and biochemical detail (Fig. 1). We envisage that this new modeling technology will open up pioneering research into all aspects of neural function in which synaptic transmission plays a role.

**Fig. 1:**
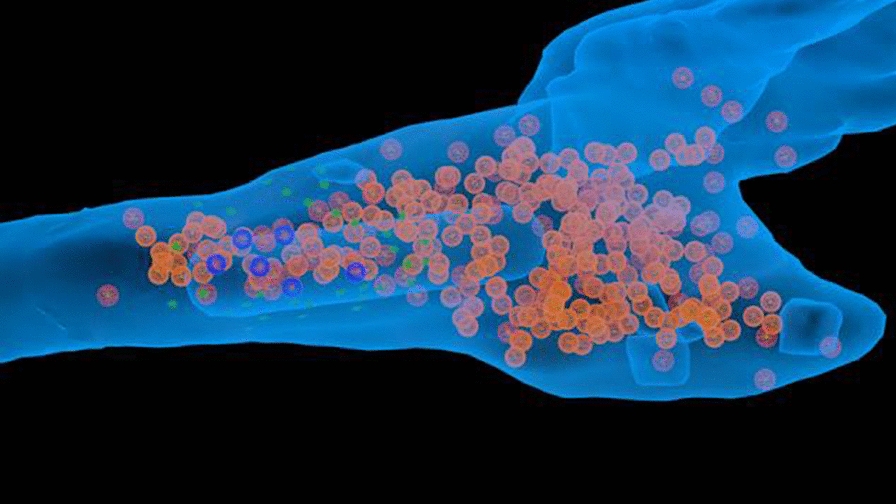
Model of a CA3-CA1 synaptic bouton with docked vesicles (blue), free vesicles (red), clustered vesicles (orange), and readily-retrievable pool (green)

**References**

1. Südhof TC. The molecular machinery of neurotransmitter release (Nobel lecture). Angewandte Chemie International Edition. *2014*; 53(47): 12696-717.
2. Hepburn I, Chen W, Wils S, De Schutter E. STEPS: efficient simulation of stochastic reaction-diffusion models in realistic morphologies. BMC Systems Biology. 2012; 6: 36.
3. Wilhelm BG, et al. Composition of isolated synaptic boutons reveals the amounts of vesicle trafficking proteins. Science. 2014; 344(6187): 1023-8.

## P38 Priors based on abstract rules modulate the encoding of pure tones in the subcortical auditory pathway

### Alejandro Tabas^1^, Glad Mihai^1^, Stefan Kiebel^2^, Robert Trampel^3^, Katharina von Kriegstein^4^

#### ^1^Max Planck Institute for Human Cognitive and Brain Sciences, Research Group in Neural Mechanisms of Human Communication, Leipzig, Germany; ^2^Technische Univerität Dresden, Chair of Neuroimage, Dresden, Germany; ^3^Max Planck Institute for Human Cognitive and Brain Sciences, Department of Neurophysics, Leipzig, Germany; ^4^Technische Univerität Dresden, Chair of Cognitive and Clinical Neuroscience, Dresden, Germany

##### **Correspondence:** Alejandro Tabas (alextabas@gmail.com)

*BMC Neuroscience* 2020, **21(Suppl 1)**:P38

Sensory pathways efficiently transmit information by adapting the neural responses to the local statistics of the sensory input. The predictive coding framework suggests that sensory neurons constantly match the incoming stimuli against an internal prediction derived from a generative model of the sensory input. Although predictive coding is generally accepted to underlay cortical sensory processing, the role of predictability in subcortical sensory coding is still unclear. Several studies have shown that single neurons and neuronal ensembles of the subcortical sensory pathway nuclei exhibit stimulus specific adaptation (SSA), a phenomenon where neurons adapt to frequently occurring stimuli (standards) yet show restored responses to a stimulus with deviating characteristics from the standard (deviant). Although neurons showing SSA are often interpreted as encoding prediction error, computational models to date have successfully explained SSA in terms of local network effects based on synaptic fatigue.

Here, we first introduce a novel experimental paradigm where abstract rules are used to manipulate predictability. 19 human participants listened to sequences of pure tones consisting on seven standards and one deviant while we recorded mesoscopic responses in auditory thalamus and auditory midbrain using 7-Tesla functional MRI. In each sequence, the deviant was constrained to occur once and only once, and always in locations 4, 5 or 6. Although the three locations were equiprobable at the beginning of the trial, the conditional probability of hearing a deviant in location n after hearing n-1 standards is 1/3, 1/2, and 1, for deviant locations 4, 5, and 6, respectively.

This paradigm yields different outcomes for habituation and predictive coding: if adaptation is driven by local habituation only, the three deviants should elicit similar neuronal responses; however, if it is predictive coding that entails adaptation, the neuronal responses to each deviant should depend on their abstract predictability. Our data showed that the responses to the deviants were strongly driven by abstract expectations, indicating that predictive coding is the main mechanism underlying mesoscopic SSA in the subcortical pathway. These results are robust even at the single-subject level.

Next, we developed a new model of pitch encoding for pure tones following the main directives of predictive coding. The model comprises two layers whose dynamics reflect two different levels of abstraction. The lower layer receives its inputs from the auditory nerve and makes use of the finite bandwidth of the peripheral filters to decode pitch fast and robustly. The second layer holds a sparse representation that integrates the activity in the first layer only once the pitch decision has been made. Top-down afferents from the upper layer reinforce the pitch decision and accept the inclusion of priors that facilitate the decoding of predictable tones.

Without the inclusion of priors, the model explains the key elements of SSA in animal recordings at the single-neuron level, as well as the main phenomenology of its mesoscopic representation. The inclusion of priors reflecting the abstract rules described in our paradigm facilitates the decoding of tones according to their predictability, effectively modulating the responses at the mesoscopic level. This modulation affects the mesoscopic fields generated during pitch encoding, fully explaining our experimental data.

**Fig. 1:**
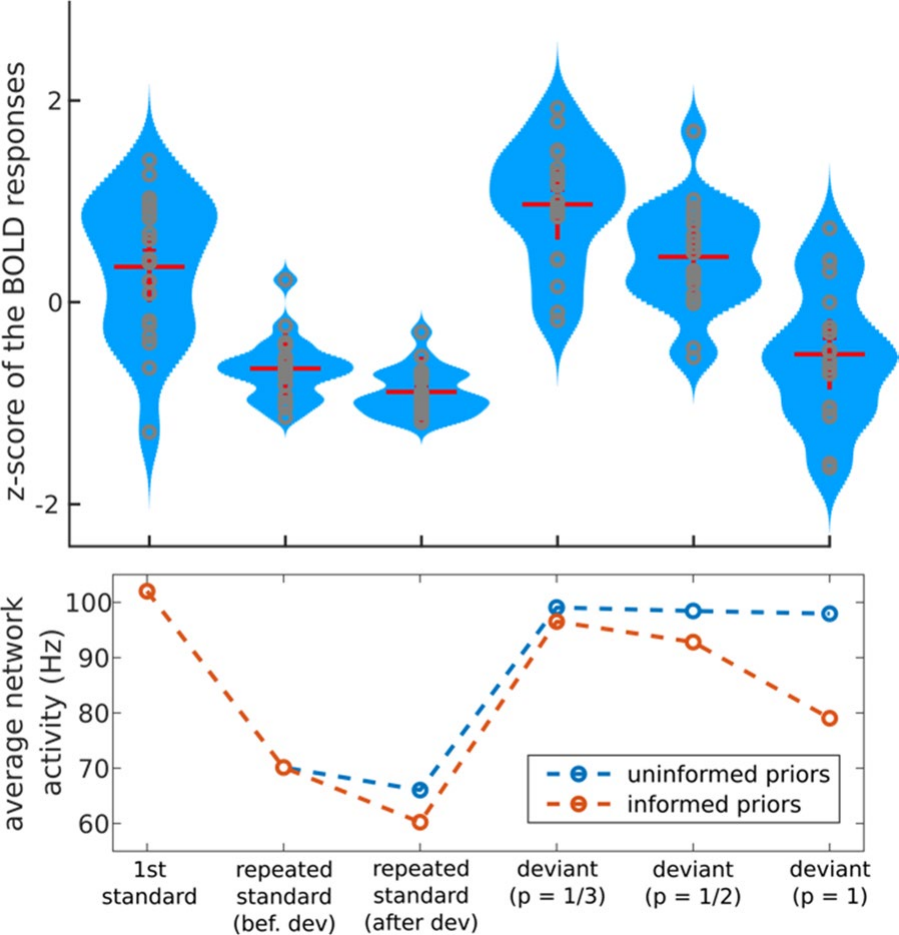
Top: z-scores of the BOLD responses to standards and deviants in the left auditory thalamus. Similar responses were recorded in the right auditory thalamus and bilateral auditory midbrain. Bottom: average model predictions under the habituation only (without informed priors) and predictive coding (with informed priors) scenarios

## P39 Simulating cell to cell interactions during the cerebellar development

### Mizuki Kato, Erik De Schutter

#### Okinawa Institute of Science and Technology, Computational Neuroscience Unit, Onna-son, Japan

##### **Correspondence:** Mizuki Kato (mizuki.kato@oist.jp)

*BMC Neuroscience* 2020, **21(Suppl 1)**:P39

The cerebellum is involved in both motor and non-motor functions in the brain. Any deficit during its development has been suggested to trigger ataxia as well as various psychiatric disorders.

During the development of both human and mouse cerebella, precursors of one of the main excitatory neurons, granule cells, first accumulate in the external granule layer on the surface and subsequently migrate down to the bottom of cerebellar cortex. In addition to the massive soma migration, these granule cell precursors also descend their axons through the migratory paths which further branch into parallel fibers, making the environment even more crowded. Although palisade-like Bergmann glia physically guide granule cells during the migration, mechanisms about how these two cell types interact to manage the migration through such a shambolic environment are still unclear.

Rodent cerebella have been widely used as subjects in experimental studies, and have provided great pictures of granule cells and Bergmann glia. However, technical limitations still hinder the observation of cerebellar development both as populations and in a continuous manner. Building a computational model by a reverse-engineering process which integrates available biological observations will be essential to point out differences in the developmental dynamics between normal and abnormal cerebellum.

Most computational models for simulating neuronal development have focused on intracellular factors of single cell types. Although models simulating limited environmental factors exist, models for cell-cell interactions during neuronal development are rare. Alternatively, we used new computational framework, NeuroDevSim, to simulate populations of granule cells and Bergmann glia during cerebellar development.

NeuroDevSim evolved from NeuroMaC [1] and so far is the only active software that can simultaneously simulate developmental dynamics of different types of neurons at population-scale.

The goal structure of simulation with NeuroDevSim comprises 3,000 granule cells and 200 Bergmann glia in a 1x106µm^3^ regular cube, calculated by assuming a cube of mice cerebellar cortex. 26 Purkinje cell somas are also introduced as interfering spherical objects. Their dendritic development will be included in the future. At current stage of the simulation, reduced systems are used, aiming to direct the traffic of granule cell somas and to navigate their axonal growth.

The resulted model will enable visualization of massive migration dynamics of cerebellar granule cells with growing parallel fibers and of their phenomenological interactions with Bergmann glia. This model will provide new insight to understand developmental dynamics of cerebellar cortex.

**Reference**

1. Torben-Nielsen B, De Schutter E. Context-aware modeling of neuronal morphologies. Frontiers in Neuroanatomy. 2014; 8: 92.

## P40 Modeling multi-state molecules with a pythonic STEPS interface

### Jules Lallouette, Erik De Schutter

#### Okinawa Institute of Science and Technology, Computational Neuroscience Unit, Onna-son, Japan

##### **Correspondence:** Jules Lallouette (jules.lallouette@oist.jp)

*BMC Neuroscience* 2020, **21(Suppl 1)**:P40

Molecules involved in biological signaling pathways can, in some cases, exist in a very high number of different functional states. Well-studied examples include the Ca2+/calmodulin dependent protein kinase II (CaMKII), or receptors from the ErbB family. Through a combination of binding sites, phosphorylation sites, and polymerization, these molecules can form complexes that reach exponentially increasing numbers of distinct states. This phenomenon of combinatorial explosion is a common obstacle when trying to establish detailed models of signaling pathways.

Classical approaches to the stochastic simulation of chemical reactions require the explicit characterization of all reacting species and all associated reactions. This approach is well suited to population based methods in which there are a relatively low number of different molecules that can be present at relatively high concentrations. Since each state of multi-state complexes would however have to be modeled as a distinct specie, the combinatorial explosion that we mentioned earlier makes these approaches inapplicable.

Two separate problems need to be tackled: the “specification problem” which requires a higher level of abstraction in the definition of complexes and reactions; and the “computation problem” that requires efficient methods to simulate the time evolution of reactions involving multi-state complexes. Rule based modeling (RBM) [1] tackles the former problem by allowing modelers to write “template” reactions that only contain the parts of the complexes actually involved in the reaction. Network-free methods together with particle-based methods [2] usually tackle the latter problem by only considering the states and reactions that are accessible from the current complex states and thus avoiding the computation of the full reaction network.

STEPS is a spatial stochastic reaction-diffusion simulation software that implements population based methods to simulate reaction-diffusion processes on realistic tetrahedral meshes [3]. In an effort to tackle the “specification problem” in STEPS, we present in this poster a novel, more pythonic, interface to STEPS that allows intuitive declaration of both classical and multi-state complexes reactions. Significant emphasis was put on simplifying model declaration, data access, and data saving during simulations.

To specifically tackle the “computation problem” in STEPS, we present a hybrid population/particle based method to simulate reactions involving multi-state complexes. By preventing the formation of arbitrarily structured macromolecules, we lower the computational cost of the pattern matching step necessary to identify potential reactants [4]. This diminished computational cost allows us to simulate larger spatial systems. We discuss these performance improvements and present examples of stochastic spatial simulations involving 12 subunits CamKII complexes which would have previously been intractable in STEPS.

**References**

1. Chylek LA, Stites EC, Posner RG, Hlavacek WS. Innovations of the rule-based modeling approach. Systems Biology. 2013; 273-300.
2. Tapia JJ, et al. Mcell-r: A particle-resolution network-free spatial modeling framework. Modeling Biomolecular Site Dynamics. 2019; 203-229.
3. Hepburn I, Chen W, Wils S, De Schutter E. STEPS: efficient simulation of stochastic reaction–diffusion models in realistic morphologies. BMC Systems Biology. 2012; 6(1): 1-9.
4. Blinov ML, Yang J, Faeder JR, Hlavacek WS. Graph theory for rule-based modeling of biochemical networks. Transactions on Computational Systems Biology VII. 2006; 89-106.

## P41 Reaction-diffusion simulations of astrocytic Ca2+ signals in realistic geometries

### Audrey Denizot^1^, Corrado Calì^2^, Weiliang Chen^1^, Iain Hepburn^1^, Hugues Berry^3^, Erik De Schutter^1^

#### ^1^Okinawa Institute of Science and Technology, Computational Neuroscience Unit, Onna-son, Japan; ^2^Neuroscience Institute Cavalieri Ottolenghi, University of Turin, Department of Neuroscience, Turin, Italy; ^3^INRIA, University of Lyon, LIRIS, UMR5205 CNRS, Villeurbanne, France

##### **Correspondence:** Audrey Denizot (audrey.denizot2@oist.jp)

*BMC Neuroscience* 2020, **21(Suppl 1)**:P41

Astrocytes, glial cells of the central nervous system, display a striking diversity of Ca2+ signals in response to neuronal activity. 80% of those signals take place in cellular ramifications that are too fine to be resolved by conventional light microscopy [1], often in apposition to synapses (perisynaptic astrocytic processes, PAPs). Understanding Ca2+ signaling in PAPs, where astrocytes potentially regulate neuronal information processing [2], is crucial. At this spatial scale, Ca2+ signals are not distributed uniformly, being preferentially located in so-called Ca2+ hotspots [3], suggesting the existence of subcellular spatial domains. However, because of the spatial scale at stake, little is currently known about the mechanisms that regulate Ca2+ signaling in fine processes. Here, we investigate the geometry of the endosplamic reticulum (ER), the predominant astrocytic Ca2+ store, using electron microscopy. Contrary to previous reports [4], we detect ER in PAPs, which can be as close as ~60nm to the closest postsynaptic density. We use computational modeling to investigate the impact of the observed cellular and ER geometries on Ca2+ signaling. Simulations using the stochastic voxel-based model from Denizot et al [5], both in simplified and in realistic 3D geometries, reproduce spontaneous astrocytic microdomain Ca2+ transients measured experimentally. In our simulations, the effect of the clustering of IP3R channels observed in 2 spatial dimensions [5] is still valid in a simple cylinder geometry but no longer holds in complex realistic geometries. We propose that those discrepancies might result from the geometry of the ER and that, in 3 spatial dimensions, the effects of molecular distributions (such as IP3R clustering) are particularly enhanced at ER-plasma membrane contact sites. Our results suggest that the predictions from simulations in 1D, 2D or simplified 3D geometries should be cautiously interpreted. Overall, this work provides a better understanding of IP3R-dependent Ca2+ signals in fine astrocytic processes and more generally in subcellular compartments, a prerequisite for understanding the dynamics of Ca2+ hotspots, which are deemed essential for local intercellular communication.

**References**

1. Bindocci E, et al. Three-dimensional Ca2+ imaging advances understanding of astrocyte biology. Science. 2017; 356(6339).
2. Savtchouk I, Volterra A. Gliotransmission: Beyond Black-and-White. Journal of Neuroscience. 2018; 38(1): 14–25.
3. Thillaiappan NB, Chavda AP, Tovey SC, Prole DL, Taylor CW. Ca 2+ signals initiate at immobile IP 3 receptors adjacent to ER-plasma membrane junctions. Nature Communications. 2017; 8(1): 1-6.
4. Patrushev I, Gavrilov N, Turlapov V, Semyanov A. Subcellular location of astrocytic calcium stores favors extrasynaptic neuron–astrocyte communication. Cell calcium. 2013; 54(5): 343-9.
5. Denizot A, Arizono M, Nägerl UV, Soula H, Berry H. Simulation of calcium signaling in fine astrocytic processes: Effect of spatial properties on spontaneous activity. PLoS Computational Biology. 2019; 15(8): e1006795.

## P42 A nanometer range reconstruction of the Purkinje cell dendritic tree for computational models

### Mykola Medvidov, Weiliang Chen, Christin Puthur, Erik De Schutter

#### Okinawa Institute of Science and Technology, Computational Neuroscience Unit, Onna-son, Japan

##### **Correspondence:** Mykola Medvidov (mykola.medvidov@gmail.com)

*BMC Neuroscience* 2020, **21(Suppl 1)**:P42

Purkinje neurons are used extensively in computational neuroscience [1]. However, despite extended knowledge about Purkinje cell morphology and ultrastructure, the complete dendritic tree of Purkinje cell as well as the complete dendritic tree of other types of neurons was never reconstructed at nanometer range resolution due to the cells size and complexity. At the same time, the use of real Purkinje cell dendritic tree morphology may be very important for computational models. Considering the development of new instruments and imaging techniques that nowadays allow reconstruction of large volumes of the neuronal tissue, the main goal of our project is to reconstruct a dendritic tree of a Purkinje cell with all its dendritic spines and synapses.

Serial Block Face Microscope (SBF) is widely used to examine large volume of neuronal tissue with nanometer range resolution [2]. To obtain volume data, perfused mouse brains were processed for SBF imaging using OTO staining techniques and the best quality cerebellum slice was imaged on FEI Teneo VS Electron Microscope with pixel resolution 8x8x60 nm. An imaged volume of approximately 2.2 Terapixel was processed and aligned with Image J and Adobe Photoshop. To reconstruct the Purkinje cell dendritic tree the imaged volume was first analyzed to locate the most appropriate full cell inside the imaged volume. Second, the volume containing the cell was segmented with Ilastik [https://www.ilastik.org] and Tensor Flow deep learning network [https://github.com/tensorflow]. The super-pixels were fused with custom made software to generate a dendritic tree represented by 3d voxels. Next, a 3d surface mesh was generated based on 3d voxels array using the marching cubes algorithm [https://github.com/ilastik/marching_cubes] and the resulting mesh was processed with MeshLab to generate a final surface mesh. Finally, a tetrahedral volume mesh was generated with the TetWild software [https://github.com/Yixin-Hu/TetWild]. The resulting tetrahedral mesh of Purkinje cell full dendritic tree including cell body and initial axonal segment will be used to run large scale stochastic models using the parallel STochastic Engine for Pathway Simulation [3] (STEPS) [http://steps.sourceforge.net].

**References**

1. Zang Y, Dieudonne S, De Schutter E. Voltage- and Branch-Specific Climbing Fiber Responses in Purkinje Cells. Cell Reports. 2018; 24, 1536–1549.
2. Titze B, Genoud C. Volume scanning electron microscopy for imaging biological ultrastructure. Biology of the *Cell* 2016;108, 307-323.
3. Chen W, De Schutter E. Parallel STEPS: Large Scale Stochastic Spatial Reaction-Diffusion Simulation with High Performance Computers. Frontiers in Neuroinformatics 2017; 11, 1-15.

## P43 Responses of a Purkinje cell model to inhibition and excitation

### Gabriela C Rangel, Erik De Schutter

#### Okinawa Institute of Science and Technology, Computational Neuroscience Unit, Onna-son, Japan

##### **Correspondence:** Gabriela C Rangel (gabriela-capo@oist.jp)

*BMC Neuroscience* 2020, **21(Suppl 1)**:P43

Although the effects of inhibition on Purkinje cells have first been observed over five decades ago and have since then been intensively studied, the manner in which the cerebellar output is regulated by both inhibitory and excitatory cells has yet to be fully understood. Purkinje cells represent the sole output of the cerebellar cortex and are known to fire simple spikes as a result of the integrated excitatory and inhibitory synaptic input originating from parallel fibers and the interneurons in the molecular layer. When studied *in vivo*, both Purkinje cells and interneurons exhibit a highly irregular pattern in the firing of action potentials. The mechanisms underlying the complex interaction between the intrinsic properties of the membrane and the pattern of synaptic inputs that generate the cerebellar output have not yet been completely understood. Recent literature has underlined the importance of the inhibitory interneurons (stellate and basket cells) in shaping the simple spikes of Purkinje cells. Moreover, when inhibitory interneurons are eliminated and only asynchronous excitation is taken into account, numerous computational [1] and experimental work have reported unrealistic behavior such as very little variability between the spiking intervals, as well as very small minimum firing frequencies. The modeling approach we propose here focuses on analyzing the effects that combined inhibition and excitation have on the shape of action potential, on the firing frequency and on the time intervals in between the simple spikes. The starting point of our work was a very detailed Purkinje cell model proposed by Zang et al in [2]. Instead of varying somatic holding currents as in previous work, in here, the dendritic voltage states are determined by the balance between the frequency of inhibitory cells and the frequency of parallel fibers. Our preliminary results indicate that inhibition presents both subtractive and divisive behavior, depending on stellate cells frequency. We discuss in detail the different shapes of firing we obtained. In particular, our results capture not only simple spikes but also a trimodal firing pattern, previously observed experimentally in [3]. This trimodal firing pattern is a characteristic of mature Purkinje cells and is given by a mixture of three different phases: tonic firing, bursting and silent mode. We mapped the regions in which simple spiking occur and the regions in which simple spikes appear and we further investigate the role of the SK2 channels in eliminating or prolonging the trimodal pattern.

**References**

1. De Schutter ER, Bower JM. An active membrane model of the cerebellar Purkinje cell II. Simulation of synaptic responses. Journal of neurophysiology. 1994; 71(1): 401-19.
2. Zang Y, Dieudonné S, De Schutter E. Voltage-and branch-specific climbing fiber responses in Purkinje cells. Cell reports. 2018; 24(6): 1536-49.
3. Womack MD, Khodakhah K. Somatic and dendritic small-conductance calcium-activated potassium channels regulate the output of cerebellar Purkinje neurons. Journal of Neuroscience. 2003; 23(7): 2600-7.

## P44 3D modeling of Purkinje cell activity

### Alexey Martyushev, Erik De Schutter

#### Okinawa Institute of Science and Technology, Computational Neuroscience Unit, Onna-son, Japan

##### **Correspondence:** Alexey Martyushev (martyushev.alexey@gmail.com)

*BMC Neuroscience* 2020, **21(Suppl 1)**:P32

The NEURON software remains the main neural physiology modeling tool for scientists. Its computational methods benefit from deterministic approximations of the cable equation solutions and 1-dimensional radial calcium diffusion in cylindrical neuron morphologies [1]. However, in real neurons ions diffuse in 3-dimensional volumes [2] and membrane channels get activated in a stochastic manner. Furthermore, NEURON does not suit to model nano-sized spine morphology. In contrast, the Stochastic Engine for Pathway Simulation (STEPS) uses fully stochastic 3-dimensional methods in tetrahedral morphologies that can provide realistic modeling of neurons at the nanoscale [3, 4].

In this work, we compare the modeling results between those two environments for the Purkinje cell model developed by Zang et al. [5]. This model considers a variety of calcium, potassium and sodium channels, and the resulting calcium concentrations affecting the membrane potential of a Purkinje cell. The results demonstrate that: (i) the used cylinder light microscopy morphology can not be identically transformed into a 3D mesh; (ii) the effect of stochastic channel activation determines the timing of membrane potential spikes; (iii) the kinetics of calcium activated potassium channels strongly depends on the specified sub-membrane volumes in both environments.

A further step in developing the model will be integration of a digital microscopy reconstruction of spines to the existing 3D tetrahedral mesh.

**References**

1. Carnevale NT, Hines ML. The NEURON book. Cambridge University Press; 2006 Jan 12.
2. Anwar H, Roome CJ, Nedelescu H, Chen W, Kuhn B, De Schutter E. Dendritic diameters affect the spatial variability of intracellular calcium dynamics in computer models. Frontiers in cellular neuroscience. 2014; 8: 168.
3. Hepburn I, Chen W, Wils S, De Schutter E. STEPS: efficient simulation of stochastic reaction–diffusion models in realistic morphologies. BMC systems biology. 2012; 6(1): 1-9.
4. Chen W, De Schutter E. Time to bring single neuron modeling into 3D. 2017.
5. Zang Y, Dieudonné S, De Schutter E. Voltage-and branch-specific climbing fiber responses in Purkinje cells. Cell reports. 2018; 24(6): 1536-49.

## P45 Exploring relevant spatiotemporal scales for analyses of brain dynamics

### Xenia Kobeleva^1^, Ane López-González^2^, Morten L Kringelbach^3^, Gustavo Deco^4^

#### ^1^University of Bonn, Department of Neurology, Bonn, Germany; ^2^Universitat Pompeu Fabra, Computational Neuroscience Group, Barcelona, Spain; ^3^University of Oxford, Department of Psychiatry, Oxford, United Kingdom; ^4^Universitat Pompeu Fabra, Barcelona, Spain

##### **Correspondence:** Xenia Kobeleva (xkobeleva@gmail.com)

*BMC Neuroscience* 2020, **21(Suppl 1)**:P45

**Introduction:** The brain switches between cognitive states at a high speed by rearranging interactions between distant brain regions. Using analyses of brain dynamics neuroimaging researchers were able to further describe this dynamical brain-behavior relationship. However, the diversity of methodological choices for the brain dynamics analyses impedes comparisons between studies of brain dynamics, reducing their reproducibility and generalizability. A key choice constitutes deciding on the spatiotemporal scale of the analysis, which includes both the number of regions (spatial scale) as well as the sampling rate (temporal scale). Choosing a suboptimal scale might either lead to loss of information or inefficient analyses with increase of noise. Therefore, the aim of this study was to assess the effect of different spatiotemporal scales on analyses of brain dynamics and to determine which spatiotemporal scale would retrieve the most relevant information on dynamic spatiotemporal patterns of brain regions.

**Methods:** We compared the effect of different spatiotemporal scales on the information content of the evolution of spatiotemporal patterns using empirical as well as simulated timeseries. Empirical timeseries were extracted from the Human Connectome Project [1]. We then created a whole-brain mean-field model of neural activity [2] resembling the key properties of the empirical data by fitting the global synchronization level and measures of dynamical functional connectivity. This resulted in different spatiotemporal with spatial scales from 100 to 900 regions and varying temporal scales from milliseconds to seconds. With a variation of an eigenvalue analysis [3], we estimated the number of spatiotemporal patterns over time and then extracted these patterns with an independent component analysis. The evolution of these patterns was then compared between scales in regard to the richness of switching activity (corrected for the number of patterns in total) using the measure of entropy. Given the probability of the occurrence of a pattern over time, we defined the entropy as a function of the probability of patterns.

**Results:** Using the entropy measure, we were able to specify both optimal and temporal scales for the evolution of spatiotemporal patterns. The entropy followed an inverted U-shaped function with the highest value at an intermediate parcellation of n = 300. The entropy was highest at a temporal scale of around 200ms.

**Conclusions and discussion:** We have investigated which spatiotemporal scale contained the highest information content for brain dynamics analyses. By combining whole-brain computational modelling with an estimation of the number of resulting patterns, we were able to analyze whole-brain dynamics in different spatial and temporal scales. From a probabilistic perspective, we explored the entropy of the probability of resulting brain patterns, which was highest at a parcellation of n = 300. Our results indicate that although more spatiotemporal patterns with increased heterogeneity are found with higher parcellations, the most relevant information on brain dynamics is captured when using a spatial scale of n = 200 and a temporal scale of 200ms. Our results therefore provide guidance for researchers on choosing the optimal spatiotemporal scale in studies of brain dynamics.

**References**

1.Van Essen DC, Smith SM, Barch DM, Behrens TE, Yacoub E, Ugurbil K, Wu-Minn HCP Consortium. The WU-Minn human connectome project: an overview. Neuroimage. 2013; 80: 62-79.
Deco G, et al. Resting-state functional connectivity emerges from structurally and dynamically shaped slow linear fluctuations. Journal of Neuroscience. 2013; 33(27): 11239-52.
Deco G, Cruzat J, Kringelbach ML. Brain songs framework used for discovering the relevant timescale of the human brain. Nature Communications. 2019; 10(1): 1-3.

## P46 Faster gradient descent learning

### Ho Ling Li^1^, Mark van Rossum^2^

#### ^1^University of Nottingham, School of Psychology, Nottingham, United Kingdom; ^2^University of Nottingham, School of Psychology and School of Mathematical Sciences, Nottingham, United Kingdom

##### **Correspondence:** Ho Ling Li (holing.li@nottingham.ac.uk)

*BMC Neuroscience* 2020, **21(Suppl 1)**:P46

Back-propagation is a popular machine learning algorithm that uses gradient descent in training neural networks for supervised learning. In stochastic gradient descent a cost function C is minimized by adjusting the weights wij as Δwij= -η(∂C/∂wij) at every training sample. However, learning with back-propagation can be very slow. A number of algorithms have been developed to speed up convergence and improve robustness of the learning. One way is to start with a high learning rate and anneal it to lower values at the end of learning. Other approaches combine past updates with the current weight update, such as momentum [1] and Adam [2]. These algorithms are now standard in most machine learning studies, but are complicated to implement biologically.

Inspired by synaptic competition in biology, we have come up with a simple and local gradient descent optimization algorithm that can reduce training time, with no demand on past information. Our algorithm works similarly to the traditional gradient descent used in back-propagation, except that instead of having a uniform learning rate across all synapses, the learning rate depends on the current connection weights of individual synapses and the L2norm of the weights of each neuron.

Our algorithm encourages neurons to form strong connections to a handful of neurons of their neighbouring layers by assigning higher learning rateηijto synapses with bigger weights wij: Δwij= -η0(|wij|+α)/(||**w**j||+α)(∂C/∂wij), where i represents the indices of the post-synaptic neurons and j represents the indices of the pre-synaptic neurons. The parameter α is set at the range of values such that at the beginning of training α > ||wj|| ≫ wij so that all synapses have learning rate close to η0. As learning progresses, the learning rate of large synapses stays close to η0, while the learning rate of small synapses decreases. Here, ||wj|| is summing over all the post-synaptic weights of a pre-synaptic neuron, leading to each pre-synaptic neuron having strong connections to a limited amount of post-synaptic neurons only. However, our algorithm also works by replacing this term with ||wi||, which promotes every post-synaptic neuron to form strong connections to small number of pre-synaptic neurons instead. We note that the proposed modulation of learning can easily be imagined to occur in biology, as it only requires post-synaptic factors and requires no memory.

We have tested our algorithm with back-propagation networks with one hidden layer consisting of 100 units to classify the MNIST handwritten digit dataset with 96% accuracy. Compared to networks equipped with the best constant learning rate, networks train 24% faster with our algorithm. The improvement is even greater with smaller networks: with 50 units in the hidden layer, our algorithm shortens the training time by 40% with respect to the best constant learning rate. Preliminary results also show that our algorithm is comparable to Adam for the small networks that we have tested. Thus, our algorithm has shown the possibility of a local and biological gradient descent optimization algorithm that only requires online information.

**References**

1. Plaut D, Nowlan S, Hinton G. Experiments on Learning by Back Propagation. Technical Report CMU-CS-86-126. 1986.
2. Kingma DP, Ba J. Adam: A method for stochastic optimization. arXiv preprint: 1412.6980. 2014.

## P47 Brain dynamics and structure-function relationships via spectral factorization and the transfer function

### James Henderson^1^, Peter Robinson^1^, Mukesh Dhamala^2^

#### ^1^The University of Sydney, School of Physics, Sydney, Australia; ^2^Georgia State University, Department of Physics and Astronomy, Atlanta, Georgia, United States of America

##### **Correspondence:** James Henderson (james.henderson@sydney.edu.au)

*BMC Neuroscience* 2020, **21(Suppl 1)**:P47

The relationships between brain activity and structure are of central importance to understanding how the brain carries out its functions and to interrelating and predicting different kinds of experimental measurements. The aim of this work is to first describe the transfer function and its relationships to many existing forms of brain analysis, and then to describe methods for obtaining the transfer function, with emphasis on spectral factorization using the Wilson algorithm [1,2] applied to correlations of time series measurements.

The transfer function of a system contains complete information about its linear properties, responses, and dynamics. This includes relationships to impulse responses, spectra, and correlations. In the case of brain dynamics, it has been shown that the transfer function is closely related to brain connectivity, including time delays, and we note that linear coupling is widely used to model the spatial interactions of locally nonlinear dynamics.

It is shown how the brain’s linear transfer function provides a means of systematically analyzing brain connectivity and dynamics, providing a robust way of inferring connectivity, and activity measures such as spectra, evoked responses, coherence and causality, all of which are widely used in brain monitoring. Additionally, the eigenfunctions of the transfer function are natural modes of the system dynamics and thus underlie spatial patterns of excitation in the cortex. Thus, the transfer function is a suitable object for describing and analyzing the structure-function relationship in brains.

The Wilson spectral factorization algorithm is outlined and used to efficiently obtain linear transfer functions from experimental two-point correlation functions. Criteria for time series measurements are described for the algorithm to accurately reconstruct the transfer function, including comparing the algorithm’s theoretical computational complexity with empirical runtimes for systems of similar size to current experiments. The algorithm is applied to a series of examples of increasing complexity and similarity to real brain structure in order to test and verify that it is free of numerical errors and instabilities (and modifying the method where required to ensure this). The results of applying the algorithm to a 1D test case with asymmetry and time delays is shown in (Fig. 1). The method is tested on increasingly realistic structures using neural field theory, introducing time delays, asymmetry, dimensionality, and complex network connectivity to verify the algorithm’s suitability for use on experimental data.

**Fig. 1:**
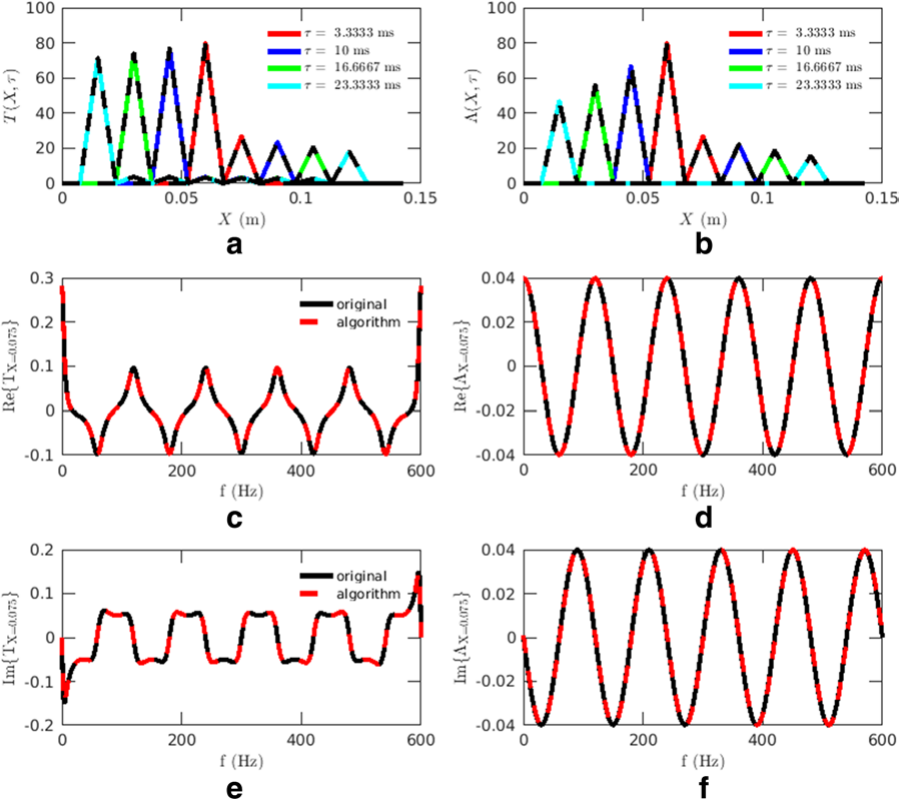
Reconstruction of the transfer function and propagator using the Wilson algorithm from a correlation matrix for a 1D test case with asymmetry and time delays. **a** The original (black lines) and reconstructed (colored lines) transfer functions as a function of space, for four time delays. **b** As for **a**, but for the system propagator. **c** The real part of the original (black line) and re constructed (red line) transfer function as a function of frequency. **d** As for c, but for the system propagator. **e** The imaginary part of the original (black line) and reconstructed (red line) transfer function as a function of frequency. **f** As for (e), but for the system propagator

**Acknowledgements:** This work was supported by the Australian Research Council under Center of Excellence grant CE140100007 and Laureate Fellowship grant FL140100025.

**References**

1. Dhamala M, Rangarajan G, Ding M. Estimating Granger causality from Fourier and wavelet transforms of time series data. Physical Review Letters. 2008; 100(1): 018701.
2. Wilson, GT. The Factorization of Matrical Spectral Densities. SIAM Journal on Applied Mathematics. 1972; 23: 420.

## P48 Large-scale spiking network models of primate cortex as research platforms

### Sacha J van Albada^1,2^, Aitor Morales-Gregorio^1,3^, Alexander van Meegen^1,3^, Jari Pronold^1,3^, Agnes Korcsak-Gorzo^1,3^, Hannah Vollenbröker^1,4^, Rembrandt Bakker^1,5^, Stine B Vennemo^6^, Håkon B Mørk^6^, Jasper Albers^1,3^, Hans E Plesser^1,6^, Markus Diesmann^1,7,8^

#### ^1^Jülich Research Centre, Institute of Neuroscience and Medicine (INM-6), Institute for Advanced Simulation (IAS-6), and JARA Institute Brain Structure-Function Relationships (INM-10), Jülich, Germany; ^2^Institute of Zoology, University of Cologne, Cologne, Germany; ^3^RWTH Aachen University, Aachen, Germany; ^4^Heinrich Heine University Düsseldorf, Düsseldorf, Germany; ^5^Radboud University, Donders Institute for Brain, Cognition and Behavior, Nijmegen, Netherlands; ^6^Norwegian University of Life Sciences, Faculty of Science and Technology, Ås, Norway; ^7^RWTH Aachen University, Department of Psychiatry, Psychotherapy and Psychosomatics, Medical Faculty, Aachen, Germany; ^8^RWTH Aachen University, Department of Physics, Faculty 1, Aachen, Germany

##### **Correspondence:** Sacha J van Albada (s.van.albada@fz-juelich.de)

*BMC Neuroscience* 2020, **21(Suppl 1)**:P48

Despite the wide variety of available models of the cerebral cortex, a unified understanding of cortical structure, dynamics, and function at different scales is still missing. Key to progress in this endeavor will be to bring together the different accounts into unified models. We aim to provide a stepping stone in this direction by developing large-scale spiking neuronal network models of primate cortex that reproduce a combination of microscopic and macroscopic findings on cortical structure and dynamics. A first model describes resting-state activity in all vision-related areas in one hemisphere of macaque cortex [1,2], representing each of the 32 areas with a 1 mm^2^ microcircuit [3] with the full density of neurons and synapses. Comprising about 4 million leaky integrate-and-fire neurons and 24 billion synapses, it is simulated on the Jülich supercomputers. The model has recently been ported to NEST 3, greatly reducing the construction time. The inter-area connectivity is based on axonal tracing [4] and predictive connectomics [5]. Findings reproduced include the spectrum and rate distribution of V1 spiking activity [6], feedback propagation of activity across the visual hierarchy [7], and a pattern of functional connectivity between areas as measured with fMRI [8]. The model is available open-source [https://inm-6.github.io/multi-area-model/] and uses the tool Snakemake [9] for formalizing the workflow from the experimental data to simulation, analysis, and visualization. It serves as a platform for further developments, including an extension with motor areas [10] for studying visuo-motor interactions, incorporating function using a learning-to-learn framework [11], and creating an analogous model of human cortex [12]. It is our hope that this work will contribute to an increasingly unified understanding of cortical structure, dynamics, and function.

**Acknowledgments:** EU Grants 269921 (BrainScaleS), 604102 (HBP SGA1), 785907 (HBP SGA2), HBP SGA ICEI 800858; VSR computation time grant JINB33; DFG SPP 2041.

**References**

1. Schmidt M, Bakker R, Hilgetag CC, Diesmann M, van Albada SJ. Multi-scale account of the network structure of macaque visual cortex. Brain Structure and Function. 2018; 223(3): 1409-35.
2. Schmidt M, Bakker R, Shen K, Bezgin G, Diesmann M, van Albada SJ. A multi-scale layer-resolved spiking network model of resting-state dynamics in macaque visual cortical areas. PLoS Computational Biology. 2018; 14(10): e1006359.
3. Potjans TC, Diesmann M. The cell-type specific cortical microcircuit: relating structure and activity in a full-scale spiking network model. Cerebral cortex. 2014; 24(3): 785-806.
4. Bakker R, Wachtler T, Diesmann M. CoCoMac 2.0 and the future of tract-tracing databases. Frontiers in Neuroinformatics. 2012; 6: 30.
5. Hilgetag CC, Beul SF, van Albada SJ, Goulas A. An architectonic type principle integrates macroscopic cortico-cortical connections with intrinsic cortical circuits of the primate brain. Network Neuroscience. 2019; 3(4): 905-23.
6. Chu CC, Chien PF, Hung CP. Tuning dissimilarity explains short distance decline of spontaneous spike correlation in macaque V1. Vision Research. 2014; 96: 113-32.
7. Nir Y, et al. Regional slow waves and spindles in human sleep. Neuron. 2011; 70(1): 153-69.
8. Babapoor-Farrokhran S, Hutchison RM, Gati JS, Menon RS, Everling S. Functional connectivity patterns of medial and lateral macaque frontal eye fields reveal distinct visuomotor networks. Journal of Neurophysiology. 2013; 109(10): 2560-70.
9. Köster J, Rahmann S. Snakemake—a scalable bioinformatics workflow engine. Bioinformatics. 2012; 28(19): 2520-2.
10. Morales-Gregorio A, et al. Bernstein Conference. 2019; https://abstracts.g-node.org/conference/BC19/abstracts#/uuid/0e114351-af44-4cc8-b457-acb41f9064e6
11. Korcsak-Gorzo A, et al. Bernstein Conference. 2019; https://abstracts.g-node.org/conference/BC19/abstracts#/uuid/e28680aa-eb4c-448e-b2a8-2e0b32c2ff49
12. Pronold J, Bakker R, Morales-Gregorio A, van Albada S, van Meegen A. Multi-area spiking network models of macaque and human cortices. In NEST Conference 2019 (No. FZJ-2019-04472). Computational and Systems Neuroscience.

## P49 Electro-diffusion of ions in dendritic signal transduction

### Yinyun Li^1^, Alexander Dimitrov^2^

#### ^1^Beijing Normal University, Department of Management, Beijing, China; ^2^Washington State University Vancouver, Vancouver, Washington, United States of America

##### **Correspondence:** Yinyun Li (leeyinyun@gmail.com)

*BMC Neuroscience* 2020, **21(Suppl 1)**:P49

Electrical dynamics of cellular membranes is central to our understanding of information processing in neurons. Until recently, the physics of ionic dynamics was largely ignored [1, 2]. Indeed, ionic concentrations are usually not significantly altered by the membrane conductance. However, their effects may be sizeable when the intracellular volume is relatively small [3, 4]. More importantly, a sudden change in concentration at one location may lead to gradients of ionic concentrations within a neural process. In our work, we demonstrate some realistic neural processes in which this effect is significant. The Nernst-Planck equation of electro-diffusion was applied to a dendrite, and voltage-gated potassium and sodium channels are added into the system. The difference of dynamics of ions and membrane voltage between the condition with electro-diffusion and without electro-diffusion were collected and compared. We found the voltage is the main driving force for the membranous ion fluxes, and the feed back loop from ion concentrations to the membrane voltage may dramatically change the dynamics of the membrane voltage. When voltage-gated calcium influx and electro-diffusion was added into the system, the dynamics of potassium becomes dramatically different from the case without electro-diffusion. We conclude that the electro-diffusion of ions in a small volume may significantly change neural information processing in a non-linear effect.

**Acknowledgements:** This work is supported by the China Scholarship Council.

**References**

1. Savtchenko LP, Poo MM, Rusakov DA. Electrodiffusion phenomena in neuroscience: a neglected companion. Nature Reviews Neuroscience. 2017; 18(10): 598.
2. Solbrå A, et al. A Kirchhoff-Nernst-Planck framework for modeling large scale extracellular electrodiffusion surrounding morphologically detailed neurons. PLoS Computational Biology. 2018; 14(10): e1006510.
3. Qian N, Sejnowski TJ. An electro-diffusion model for computing membrane potentials and ionic concentrations in branching dendrites, spines and axons. Biological Cybernetics. 1989; 62(1): 1-5.
4. Lopreore CL, et al. Computational modeling of three-dimensional electrodiffusion in biological systems: application to the node of Ranvier. Biophysical Journal. 2008; 95(6): 2624-35.

## P50 Spatial denoising through topographic modularity in networks of spiking neurons

### Barna Zajzon^1^, Abigail Morrison^2^, Renato Duarte^3^

#### ^1^Jülich Research Center, Institute of Neuroscience and Medicine (INM-6), Jülich, Germany; ^2^Forschungszentrum Jülich GmbH, Jülich, Germany; ^3^Jülich Research Center, Institute of Neuroscience and Medicine (INM-6) and Institute for Advanced Simulation (IAS-6), Jülich, Germany

##### **Correspondence:** Barna Zajzon (b.zajzon@fz-juelich.de)

*BMC Neuroscience* 2020, **21(Suppl 1)**:P50

Topographic maps are a pervasive structural feature of the mammalian brain, present throughout the cortical hierarchy and particularly prevalent in the early sensory systems. These ordered projections arrange and preserve the relative organization of cells between distinct populations and have been the object of many empirical studies. From providing a structural scaffold for spatial information segregation to the organization of spatiotemporal feature maps, these ubiquitous anatomical features are known to have significant, albeit not entirely understood, functional consequences.

In this work, we systematically investigate the functional and dynamical impact of the characteristics of modular propagation pathways in large networks of spiking neurons. Specifically, we manipulate key structural parameters such as modularity, map size and degree of overlap, and evaluate their impact on the network dynamics and computational performance during a continuous signal reconstruction task from noisy inputs. We show that transmission accuracy increases as topographic projections become more structured, with even moderate degrees of modularity improving overall discrimination capability.

Moreover, we identify a condition where the global population statistics converges towards a stable asynchronous irregular regime, allowing for a linear firing rate propagation along the topographic maps. In such conditions, the networks exhibit spatial denoising properties and the task performance improves vastly with hierarchical depth. Importantly, the relative performance gain throughout the hierarchy increases with the amount of noise in the input. This suggests that topographic modularity is not only essential for accurate neural communication, but it can also provide the structural underpinnings to handle noisy and corrupt information streams.

Using field-theoretic approximations, we demonstrate that this phenomenon can be attributed to a disruption in the E-I balance throughout the network. By changing the effective connectivity within the system, strongly modular projections facilitate the emergence of inhibition-dominated regimes where population responses along active maps are amplified, whereas others are weakened or silenced.

In addition, we analytically derive constraints on the possible extent of such maps, given that in cortical networks topographic specificity is assumed to decrease with hierarchical depth. Our findings suggest that, while task performance is relatively robust to variation in the map sizes, there is a fine balance between the spatial extent of the topographic projections and their modularity. Maps may not become arbitrarily small and must compensate for the size through denser topographic connections, whereas this can actually have a detrimental effect in the case of larger maps if they overlap.

Taken together, these results highlight the functional benefits of structured connectivity in hierarchical neural networks, and shed light on a potential new role for modular topographic maps as a denoising mechanism.

**Acknowledgements**: The authors gratefully acknowledge the computing time granted by the JARA Vergabegremium and provided on the JARA Partition part of the supercomputer JURECA at Forschungszentrum Jülich.

## P51 Data-driven multiscale modeling with NetPyNE: new features and example models

### Joe Graham^1^, Matteo Cantarelli^2^, Filippo Ledda^2^, Dario Del Piano^2^, Facundo Rodriguez^2^, Padraig Gleeson^3^, Samuel A Neymotin^4^, Michael Hines^5^, William W Lytton^6^, Salvador Dura-Bernal^6^

#### ^1^SUNY Downstate Medical Center, Neurosim Lab, Brooklyn, New York, United States of America; ^2^MetaCell, LLC, Cambridge, Massachusetts, United States of America; ^3^University College London, Department of Neuroscience, Physiology & Pharmacology, London, United Kingdom; ^4^Nathan Kline Institute for Psychiatric Research, Orangeburg, New York, United States of America; ^5^Yale University, School of Medicine, New Haven, Connecticut, United States of America; ^6^SUNY Downstate Medical Center, Department of Physiology and Pharmacology, Brooklyn, New York, United States of America

##### **Correspondence:** Joe Graham (joe.w.graham@gmail.com)

*BMC Neuroscience* 2020, **21(Suppl 1)**:P51

Neuroscience experiments generate vast amounts of data that span multiple scales: from interactions between individual molecules, to behavior of cells, to circuit activity, to waves of activity across the brain. Biophysically-realistic computational modeling provides a tool to integrate and organize experimental data at multiple scales. NEURON is a leading simulator for detailed neurons and neuronal networks. However, building and simulating networks in NEURON is technically challenging, requiring users to implement custom code for many tasks. Also, lack of format standardization makes it difficult to understand, reproduce, and reuse many existing models.

NetPyNE is a Python interface to NEURON which addresses these issues. It features a user-friendly, high-level declarative programming language. At the network level for example, NetPyNE automatically generates connectivity using a concise set of user-defined specifications rather than forcing the user to explicitly define millions of cell-to-cell connections. NetPyNE enables users to generate NEURON models, run them efficiently in automatically parallelized simulations, optimize and explore network parameters through automated batch runs, and use built-in functions for a wide variety of visualizations and analyses. NetPyNE facilitates sharing by exporting and importing standardized formats (NeuroML and SONATA), and is being widely used to investigate different brain phenomena. It is also being used to teach basic neurobiology and neural modeling. NetPyNE has recently added support for CoreNEURON, the compute engine of NEURON optimized for the latest supercomputer hardware architectures.

In order to make NetPyNE accessible to a wider range of researchers and students, including those with limited programming experience, and to encourage further collaboration between experimentalists and modelers, all its functionality is accessible via a state-of-the-art graphical user interface (GUI). From a browser window, users can intuitively define their network models, visualize and manipulate their cells and networks in 3D, run simulations, and visualize data and analyses. The GUI includes an interactive Python console which synchronizes with the underlying Python-based model.

The NetPyNE GUI is currently being improved in several ways. *Flex Layout* is being introduced to ensure a responsive, customizable GUI layout regardless of screen size or orientation. *Redux* is being added to the stack to ensure the complete state of the app is known at all times, minimizing bugs and improving performance. *Bokeh* is being used to create interactive plots. Furthermore, by integrating NetPyNE with *Open Source Brain*, users will be able to create online accounts to manage different workspaces and models (create, save, share, etc.). This will allow interaction with online repositories to pull data and models into NetPyNE projects, from resources such as *ModelDB*, *NeuroMorpho*, *GitHub*, etc.

In this poster, we present the latest improvements in NetPyNE and discuss recent data-driven multiscale models utilizing NetPyNE for different brain regions, including: primary motor cortex, primary auditory cortex, and a canonical neocortex model underlying the Human Neocortical Neurosolver, a software tool for interpreting the origin of MEG/EEG data.

**Acknowledgments**: Supported by NIH U24EB028998, U01EB017695, DOH01-C32250GG-3450000, R01EB022903, R01MH086638, R01DC012947, and ARO W911NF1910402.

## P52 Connectivity modulation by dual-site transcranial alternating current stimulation based on spike-timing dependent plasticity

### Bettina Schwab^1^, Peter König^2^, Andreas K Engel^1^

#### ^1^University Medical Center Hamburg-Eppendorf, Department of Neurophysiology and Pathophysiology, Hamburg, Germany; ^2^University of Osnabrück, Institute of Cognitive Science, Osnabrück, Germany

##### **Correspondence:** Bettina Schwab (b.schwab@uke.de)

*BMC Neuroscience* 2020, **21(Suppl 1)**:P52

Transcranial alternating current stimulation (tACS) noninvasively applies electric fields to the brain with the aim of entraining neural activity. As recordings of extracellular potentials are highly affected by the stimulation artifact, M/EEG recordings before and after tACS have become the standard to investigate neurophysiological effects of tACS in humans. In particular, we recently showed that dual-site tACS can modulate functional connectivity between the targeted regions outlasting the stimulation period, with in-phase stimulation (phase lag zero) increasing connectivity compared to anti-phase stimulation (phase lag π) [1]. Although the mechanism for such after-effects is not known, spike-timing dependent plasticity (STDP) has been proposed as a candidate [2,3]. We aim (1) to find a possible mechanism for our experimentally observed connectivity changes, and (2) to estimate if our dual-site tACS setting can successfully be extended to any stimulation frequency and target area.

We simulated two populations of each 1000 regularly spiking Izhikevich neurons [4] with realistic firing rate distributions. Excitatory connections from each neuron to 100 random neurons of the other population with synaptic delay were subject to an experimentally observed STDP rule [5]. tACS was applied as sinusoidal input currents to both populations with varying phase lags. To validate our model, we correlated experimentally found connectivity changes [1] between targeted sub-regions with the fiber length connecting those sub-regions.

Synaptic weight changes depended on tACS frequency, phase lag, and synaptic delay. For 10 Hz tACS, synaptic weight changes between in- and anti-phase tACS monotonically decreased within the range of physiological cortico-cortical conduction delays. Confirming this finding, our experimental data showed a negative correlation between functional connectivity modulation (in-phase vs anti-phase tACS) and fiber length. Extending the simulations to other tACS frequencies, we find that the expected direction of connectivity modulation can only be expected for low tACS frequencies and small delays or delays that are near multiples of the tACS cycle length.

In conclusion, our experimental findings [1] are in accordance with STDP of synapses between the stimulated regions. Nevertheless, the approach cannot be generalized to all tACS frequencies and delays. Most robust effects are expected for low tACS frequencies and small delays.

**Acknowledgements:** This work has been supported by DFG, SFB 936/project A3.

**References**

1. Schwab BC, Misselhorn J, Engel AK. Modulation of large-scale cortical coupling by transcranial alternating current stimulation. Brain stimulation. 2019; 12(5): 1187-96.
2. Wischnewski M, et al. NMDA receptor-mediated motor cortex plasticity after 20 Hz transcranial alternating current stimulation. Cerebral Cortex. 2019; 29(7): 2924-31.
3. Vossen A, Gross J, Thut G. Alpha power increase after transcranial alternating current stimulation at alpha frequency (α-tACS) reflects plastic changes rather than entrainment. Brain stimulation. 2015; 8(3): 499-508.
4. Izhikevich EM. Simple model of spiking neurons. IEEE Transactions on neural networks. 2003; 14(6): 1569-72.
5. Froemke RC, Dan Y. Spike-timing-dependent synaptic modification induced by natural spike trains. Nature. 2002; 416(6879): 433-8.

## P53 A simple, non-stationary normalization model to explain and successfully predict change detection in monkey area MT

### Detlef Wegener^1^, Xiao Chen^2^, Lisa Bohnenkamp^2^, Fingal O Galashan^1^, Udo Ernst^2^

#### ^1^University of Bremen, Brain Research Institute, Bremen, Germany; ^2^University of Bremen, Computational Neurophysics Lab, Institute for Theoretical Physics, Bremen, Germany

##### **Correspondence:** Detlef Wegener (wegener@brain.uni-bremen.de)

*BMC Neuroscience* 2020, **21(Suppl 1)**:P53

Successful visually-guided behavior in natural environments critically depends on rapid detection of changes in visual input. A wildcat chasing a gazelle needs to quickly adapt its motions to sudden direction changes of the prey, and a human driving a fast car on a highway must instantaneously react to the onset of the red brake light of the car in front. Visually responsive neurons represent such rapid feature changes in comparably rapid, transient changes of their firing rate. In the motion domain, for example, neurons in monkey area MT were shown to represent the sign and magnitude of a rapid speed change in the sign and amplitude of the evoked firing rate modulation following that change [1]. For positive speed changes, it was also shown that the transient’s latency closely correlates with reaction time, and is modulated by both spatial and non-spatial visual attention [2,3].

We here introduce a computational model based on a simple, canonical circuit in a cortical hypercolumn. We use the model to investigate the computational mechanisms underlying transient neuronal firing rate changes and their modulation by attention under a wide range of stimulus conditions. It is built of an excitatory and an inhibitory unit, both of which are in response to an external input *I*(t). The excitatory unit receives additional divisive input from the inhibitory unit. The model’s dynamics is described by two differential equations quantifying how mean activity *A*e of the excitatory unit and divisive input current change with time *t*. By fitting the model parameters to experimental data, we show that it is capable to reproduce the time courses of transient responses under passive viewing conditions. Mathematical analysis of the circuit explains hallmark effects of transient activations and identifies the relevant parameters determining response latency, peak response, and sustained activation. Visual attention is implemented by a simple multiplicative gain to the input of both units.

A key result of the analysis of the model’s dynamics is that steeper rise or decay times of the transient provide a consistent mechanisms of attentional modulation, independent of both the overall activation of the neuron prior to the speed change, and the sign of the change. This prediction is tested by new experiments requiring attention to both positive and negative speed changes. The results of the experiment are in full accordance with the prediction of the model, providing evidence that even decreases in firing rate in response to the reduction of the speed of an attended stimulus occur with shorter latency. Thus, the model provides a unique framework for a mechanistic understanding of MT response dynamics under very different sensory and behavioral conditions.

**Acknowledgments:** Supported by BMBF grant 01GQ1106 and DFG grant WE 5469/3-1.

**References**

1. Traschütz A, Kreiter AK, Wegener D. Transient activity in monkey area MT represents speed changes and is correlated with human behavioral performance. Journal of Neurophysiology. 2015; 113: 890-903.
2. Galashan FO, Saßen HC, Kreiter AK, Wegener D. Monkey area MT latencies to speed changes depend on attention and correlate with behavioral reaction times. Neuron. 2013; 78: 740-750.
3. Schledde B, Galashan FO, Przybyla M, Kreiter AK, Wegener D. Task-specific, dimension-based attentional shaping of motion processing in monkey area MT. Journal of Neurophysiology. 2017; 118: 1542-1555.

## P54 Non-invasive chronometric modeling of oculomotor processing

### Devin Kehoe, Mazyar Fallah

#### York University, Department of Psychology, Toronto, Canada

##### **Correspondence:** Devin Kehoe (dhkehoe@gmail.com)

*BMC Neuroscience* 2020, **21(Suppl 1)**:P54

There is a striking correlation between overt oculomotor behavior and neurophysiological processing in critical oculomotor substrates like the superior colliculus (SC). During oculomotor target selection, unresolved activation [1] or suppression [2] at a competing distractor locus in the epoch ~30 ms prior to saccade initiation elicits saccades curved towards or away from the distractor (respectively). We therefore developed a non-invasive technique to measure the time course of excitatory and inhibitory activity encoding a distractor in which human saccade curvature is modeled as a function of *saccade-distractor onset asynchrony* (SDOA): the time between the transient onset of a task irrelevant distractor and the initiation of a saccade to a target [3]. The distractor processing time course observed using this technique was closely aligned to the time course of visuomotor neural activity in SC during target selection [4] and we observed time course differences between luminance- and color-modulated distractors that were also in alignment with SC visuomotor cell activity [5].

We expanded the SDOA technique to examine oculomotor processing of complex objects during a perceptual discrimination saccade-task with varied visual similarity between distractors and targets. We saw that the latency of the initial excitatory response was ~60 ms longer for these complicated, task-relevant objects than for the task irrelevant distractors with simple visual features [3]. We also saw differences in the excitatory processing time course for the complex objects, which has critical implications for theories of oculomotor target selection processing. We developed additional analytic techniques that estimate the latency of excitatory processing independent of saccade curvature modeling, which provided consistent temporal estimates. The first analysis examined target selection accuracy as a function of distractor processing time and showed that prior to the estimated time of distractor information being projected into the oculomotor substrates, target selection was guided exclusively by the target representation. The second analysis examined the frequency of saccades as a function of SDOA and demonstrated that immediately after the estimated time of excitatory activity encoding the distractor, there is a transient drop in the likelihood of making a saccade, mirroring the effects of flash suppression. This work confirms the validity of our non-invasive chronometric technique and our original interpretation of it, while also illustrating that SDOA saccade curvature modeling is applicable to more complicated oculomotor target selection contexts.

**References**

1. McPeek RM, Han JH, Keller EL. Competition between saccade goals in the superior colliculus produces saccade curvature. Journal of Neurophysiology. 2003; 89: 2577–2590.
2. White BJ, Theeuwes J, Munoz DP. Interaction between visual- and goal-related neuronal signals on the trajectories of saccadic eye movements. Journal of Cognitive Neuroscience. 2012; 24: 707–717.
3. Kehoe DH, Fallah M. Rapid accumulation of inhibition accounts for saccades curved away from distractors. Journal of Neurophysiology. 2017; 118: 832–844.
4. McPeek RM, Keller EL. Saccade target selection in the superior colliculus during visual search task. Journal of Neurophysiology. 2002; 88: 2019–2034.
5. White BJ, Boehnke SE, Marino RA, Itti L, Munoz DP. Color-related signals in the primate superior colliculus. Journal of Neuroscience. 2009; 29: 12159–12166.

## P55 Identifying changes in whole-brain functional connectivity in complex longitudinal clinical trials

### Sidhant Chopra^1^, Kristina Sabaroedin^2^, Shona Francey^3^, Brian O’Donoghue^3^, Vanessa Cropley^4^, Barnaby Nelson^3^, Jessica Graham^3^, Lara Baldwin^3^, Steven Tahtalian^4^, Hok Pan Yuen^3^, Kelly Allott^3^, Mario Alvarez^3^, Susy Harrigan^3^, Christos Pantelis^4^, Stephen Wood^3^, Patrick McGorry^3^, Alex Fornito^5^

#### ^1^Monash University, Turner Institute for Brain and Mental Health, Melbourne, Australia; ^2^Monash University, Melbourne, Australia; ^3^Orygen Youth Health, Melbourne, Australia; ^4^Melbourne Neuropsychiatry Centre, Melbourne, Australia; ^5^Monash University, The Turner Institute for Brain and Mental Health, School of Psychological Sciences and Monash Biomed, Melbourne, Australia

##### **Correspondence:** Sidhant Chopra (sid.chopra@monash.edu)

*BMC Neuroscience* 2020, **21(Suppl 1)**:P55

Resting-state Functional Magnetic Resonance Imaging (rs-fMRI) is increasingly being used as a secondary measure in complex clinical trials [1]. The inclusion of rs-fMRI allows researchers to investigate the impact interventions, such as medication, can have on regional and network-level brain hemodynamics. Such trials are expensive, difficult to conduct, often have small samples in rare clinical populations and high attrition rates. Standard neuroimaging analysis software are not usually suited to these sub-optimal design parameters. Accessible statistical tools that are robust to these conditions are much needed.

We propose an analysis workflow, which combines 1) ordinary least squares marginal model with a robust covariance estimator to account for within-subject correlation, 2) nonparametric p-value inference using a novel bootstrapping method [2] and, 3) edge- and component-level family-wise error (FWE) control using the Network Based Statistic [3]. This workflow has several advantages, including being robust to unbalanced longitudinal samples, small-sample correction using heteroskedasticity-consistent standard errors and simplified nonparametric inference. Additionally, this method is computationally less demanding than traditional mixed-linear models and does not bias the analysis by pre-selecting regions of interest.

We apply this novel workflow to a world-first triple-blind longitudinal placebo-controlled trial where 62 antipsychotic-naïve people aged between 15 to 24 with first-episode psychosis received either an atypical antipsychotic or a placebo pill over a treatment period of 6 months. Both patient groups received intensive psychosocial therapy. A third healthy control group with no psychiatric diagnosis (n=27) was also recruited. rs-fMRI scans were acquired at baseline, 3-months and 12-months. We show that our analysis method is sufficiently sensitive to detect FWE-corrected significant components in this complex three groups [healthy control, placebo, medication] by three time points [baseline, 12-weeks, 52-weeks] design.

Here, we introduce an analysis workflow which is capable of detecting changes in resting-state functional networks in complex clinical trials with multiple timepoints and unbalanced groups. This analysis workflow is freely available as an R function::netSandwich.

**References**

1. O’Donoghue B, et al. Staged treatment and acceptability guidelines in early psychosis study (STAGES): A randomized placebo controlled trial of intensive psychosocial treatment plus or minus antipsychotic medication for first‐episode psychosis with low‐risk of self‐harm or aggression. Study protocol and baseline characteristics of participants. Early Intervention in Psychiatry. 2019; 13(4): 953-960.
2. Guillaume B, et al. Improving mass-univariate analysis of neuroimaging data by modelling important unknown covariates: Application to Epigenome-Wide Association Studies. NeuroImage. 2018; 173: 57-71.
3. Zalesky A, Fornito A, Bullmore ET. Network-based statistic: identifying differences in brain networks. Neuroimage. 2010; 53(4): 1197-1207.

## P56 Impact of simulated asymmetric interregional cortical connectivity on the local field potential

### David Boothe, Alfred Yu, Kelvin Oie, Piotr Franaszczuk

#### U.S. Army Research Laboratory, Human Research Engineering Directorate, Aberdeen Proving Ground, Maryland, United States of America

##### **Correspondence:** David Boothe (david.l.boothe7.civ@mail.mil)

*BMC Neuroscience* 2020, **21(Suppl 1)**:P56

Spontaneous neuronal activity as observed using electroencephalogram is characterized by a non-stationary 1/f power spectrum interspersed with periods of rhythmic activity [1]. Underlying cortical neuronal activity is, by contrast, hypothesized to be sparse and arrhythmic [2]. Properties of cortical neuronal connectivity such as sparsity, small world organization, and conduction delays have all been proposed to play a critical role in the generation of spontaneous brain activity. However, the relationship between the structure reflected in measures of global brain activity, the underlying neuronal activity, and neuronal connectivity is, at present, poorly characterized. In order to explore the role of cortical connectivity in the generation of spontaneous brain activity, we present a simulation of cerebral cortex based on the Traub model [3] implemented in the GENESIS neuronal simulation environment.

We made extensive changes to the original Traub model in order to more faithfully reproduce the spontaneous cortical activity described here. We re-tuned the original Traub parameters to eliminate both intrinsic neuronal activity and removed the gap junctions. Tuning out intrinsic neuronal activity in the model allowed changes to the underlying connectivity to be the central factor in modifying overall model activity. The model we present consists of 16 simulated cortical regions each containing 976 neurons (15,616 neurons total). Previously we connected simulated regions in a nearest neighbor fashion via short range association fibers. These association fibers originated from pyramidal cells in cortical layer 2/3 (P23s). We found that the introduction of symmetric bidirectional inter-regional connectivity was sufficient to induce both a 1/f power spectrum as well as oscillatory behavior in the local field potential of the underlying cortical regions in the 2 to 40 Hz range. However we also found that sub-region activity was fairly uniform, even if these sub-region oscillations were not strongly correlated with one another. We hypothesize that introducing asymmetric inter-regional connectivity in this model may produce underlying simulated neuronal activity that is more variable in its output and more similar to the output observed in the biological system..

Connectivity between cortical regions in the biological brain are often asymmetric with outputs of layer 2/3 pyramidal cells terminating in different layers and in different proportions on receiving regions of cortex [4].

Here we explore how these asymmetrical connectivity schema alter microscopic (spikes) and macroscopic (local field potential) features of our cortical simulations. We re-organized our 16 simulated cortical regions in a hierarchical fashion using feedforward and feedback connectivity patterns observed between regions of the visual system [4]. We then compare the behavior of this network to our previous simulations using nearest neighbor and small world like inter-regional connectivity. We hypothesize that networks with asymmetric connectivity between regions will give richer and more heterogenous model outputs.

**References**

1. Le Van Quyen M. Disentangling the dynamic core: a research program for a neurodynamics at the large-scale. Biological Research. 2003; 36(1): 67-88.
2. Buzsáki G. Rhythms of the Brain: Oxford University Press; 2006.
3. Traub RD, et al. Single-column thalamocortical network model exhibiting gamma oscillations, sleep spindles, and epileptogenic bursts. Journal of Neurophysiology. 2005; 93(4): 2194-232.
4. Salin PA, Bullier J. Corticocortical connections in the visual system: structure and function. Physiological Reviews. 1995; 75(1): 107-54.

## P57 Motor cortex encodes a value function consistent with reinforcement learning

### Venkata Tarigoppula^1^, John Choi^2^, John Hessburg^2^, David McNiel^2^, Brandi Marsh^2^, Joseph Francis^2^

#### ^1^University of Melbourne, Biomedical Engineering, Melbourne, Australia; ^2^State University of New York Downstate Medical Center, Physiology and Pharmacology, New York, United States of America

##### **Correspondence:** Venkata Tarigoppula (adi.tarigoppula@unimelb.edu.au)

*BMC Neuroscience* 2020, **21(Suppl 1)**:P57

Reinforcement learning (RL) theory provides a simple model that can help explain many animal behaviors. RL models have been very successful in describing the neural activity in multiple brain regions and at several spatiotemporal scales ranging from single units up to hemodynamics during the learning process in animals including humans. A key component of RL is the value function, which captures the expected, temporally discounted reward, from a given state. A reward prediction error occurs when there is a discrepancy between the value function and actual reward, and this error is used to drive learning. The value function can also be modified by the animal’s knowledge and certainty of its environment. Here we show that the bilateral primary motor cortical (M1) neural activity in non-human primates (Rhesus and Bonnet macaques either sex) encodes a value function in line with temporal difference RL. M1 responds to the delivery of unpredictable reward (unconditional stimulus (US)), and shifts its value related response earlier in a trial, becoming predictive of expected reward, when reward is predictable due to the presence of an explicit cue (conditional stimulus (CS)). This is observed in tasks performed manually or observed passively and in tasks without an explicit CS, but with a predictable temporal reward environment. M1 also encodes the expected reward value in a multiple reward level CS-US task. Here we extend the Microstimulus temporal difference RL model (MSTD), reported to accurately capture RL related dopaminergic activity, to account for both phasic and tonic M1 reward-related neural activity in a multitude of tasks, during manual trials, as well as observational trials. This information has implications towards autonomously updating brain-machine interfaces.

## P58 Systematic testing and validation of models of hippocampal neurons against electrophysiological data

### Sára Sáray^1^, Andrew Davison^2^, Szabolcs Kali^3^, Christian Rössert^4^, Shailesh Appukuttan^5^, Eilif Muller^4^, Tamás Freund^3^

#### ^1^Pázmány Péter Catholic University, Faculty of Information Technology and Bioinics, Budapest, Hungary; ^2^Centre National de la Recherche Scientifique, Unité de Neuroscience, Information et Complexité, Gif sur Yvette, France; ^3^Institute of Experimental Medicine, Hungarian Academy of Sciences, Budapest, Hungary; ^4^École Polytechnique Fédérale de Lausanne, Blue Brain Project, Geneva, Switzerland; ^5^Centre National de la Recherche Scientifique/ Université Paris-Saclay, Paris-Saclay Institute of Neuroscience, Gif-sur-Yvette, France

##### **Correspondence:** Sára Sáray (saraysari@gmail.com)

*BMC Neuroscience* 2020, **21(Suppl 1)**:P58

Anatomically and biophysically detailed neuronal models, that are built in a data-driven manner, are useful tools in understanding and predicting the behavior and function of the different cell types of the brain. Due to the growing number of computational and software tools and the increasing body of experimental data from electrophysiological measurements, that enable more accurate neuronal modeling, there is a constantly increasing number of different models of many cell types available in the literature. These are usually developed using different methods and for different purposes, most often to reproduce the results of a few selected experiments, and it is often unknown how they would behave in other situation or whether they are able to generalize outside their original scope. This might be the reason why it is uncommon in the modelling community to re-use and further develop already existing models, which prevents the construction of consensus “community models” that could capture an increasing proportion of the electrophysiological properties of the given cell type. In addition, even when models are re-used they may lose their ability to capture their originally adjusted behavior while their parameters are retuned to make them fit another subset of experimental data.

The collaborative approach of model development requires extensive validation test suites which enables modelers to evaluate their models against experimental observations according to standardized criteria and to explore the changes in model behavior at the different stages of its development. Applying automated tests also facilitates optimal model re-use and co-operative model development by making it possible to learn more about models published by other groups (beyond the results included in the papers) with relatively little effort.

Initially we addressed this issue by developing an open-source Python test suite, called HippoUnit (https://github.com/KaliLab/hippounit) for the automated and systematic validation and quantitative comparison of the behavior of models of the hippocampal CA1 pyramidal cells (which is one of the most studied cell type) against electrophysiological data. We applied HippoUnit to test and compare the behavior of several different hippocampal CA1 pyramidal cell models available on ModelDB (results are available at: https://github.com/KaliLab/HippoUnit_demo). We also employed the test suite to aid the development of models within the Human Brain Project (HBP) and integrated the tests into the validation framework developed in the HBP.

Currently we are extending this test suite by adding new tests for the validation of other important hippocampal cell types. New validation tests cover somatic behavior and signal propagation in dendrites of basket cells and CA3 pyramidal cells, and the propagation of action potential in the axon of basket cells.

By presenting these results we hope to encourage the modeling community to use more systematic testing during model development, in order to create neural models that generalize better, and make the process of model building more reproducible and transparent.

**Acknowledgments:** Supported by the ÚNKP-19-3-III New National Excellence Program of the Ministry for Innovation and Technology; European Social Fund (EFOP-3.6.3-VEKOP-16-2017-00002); the European Union’s Horizon 2020 Framework Programme for Research and Innovation (Specific Grant Agreements No. 720270, 785907 - Human Brain Project SGA1, SGA2).

## P59 Reverse engineering neural networks to identify their cost functions and implicit generative models

### Takuya Isomura^1^, Karl Friston^2^

#### ^1^RIKEN Center for Brain Science, Saitama, Japan; ^2^University College London, London, United Kingdom

##### **Correspondence:** Takuya Isomura (takuya.isomura@riken.jp)

*BMC Neuroscience* 2020, **21(Suppl 1)**:P59

It is widely recognised that maximising a variational bound on model evidence – or equivalently, minimising variational free energy – provides a unified, normative formulation of inference and learning [1]. According to the complete class theorem [2], any dynamics that minimises a cost function can be viewed as performing Bayesian inference; implying that any neural network whose activity and plasticity follow the same cost function is implicitly performing Bayesian inference. However, the implicit Bayesian model that corresponds to any given cost function is a more delicate problem. Here, we identify a class of biologically plausible cost functions for canonical neural networks of rate coding neurons, where the same cost function is minimised by both neural activity and plasticity [3]. We then demonstrate that such cost functions can be cast as variational free energy under an implicit generative model in the well-known form of partially observed Markov decision processes. This equivalence means that the activity and plasticity in a canonical neural network can be understood as approximate Bayesian inference and learning, respectively. Mathematical analysis shows that the firing thresholds – that characterise the neural network cost function – correspond to prior beliefs about hidden states in the generative model. This means that the Bayes optimal encoding of hidden states is attained when the network’s implicit priors match the process generating its sensory inputs. The theoretical formulation was validated using *in vitro* neural networks comprising rat cortical cells cultured on a microelectrode array dish [4,5]. We observed that *in vitro* neural networks – that receive input stimuli generated from hidden sources – perform causal inference or source separation through activity-dependent plasticity. The learning process was consistent with Bayesian belief updating and the minimisation of variational free energy. Furthermore, constraints that characterise the firing thresholds were estimated from the empirical data to quantify the *in vitro* network’s prior beliefs about hidden states. These results highlight the potential utility of reverse engineering generative models to characterise the neuronal mechanisms underlying Bayesian inference and learning.

**References**

1. Friston K. The free-energy principle: a unified brain theory?. Nature Reviews Neuroscience. 2010; 11(2): 127-38.
2. Wald A. An essentially complete class of admissible decision functions. The Annals of Mathematical Statistics. 1947; 549-55.
3. Isomura T, Friston K. Reverse engineering neural networks to characterise their cost functions. bioRxiv. 2019; 654467.
4. Isomura T, Kotani K, Jimbo Y. Cultured cortical neurons can perform blind source separation according to the free-energy principle. PLoS Computational Biology. 2015; 11(12): e1004643.
5. Isomura T, Friston K. In vitro neural networks minimise variational free energy. Scientific Reports. 2018; 8(1): 1-4.

## P60 Cholinergic modulation can produce rapid task-related plasticity in the auditory cortex

### Jordan Chambers^1^, Shihab Shamma^2^, Anthony Burkitt^1^, David Grayden^1^, Diego Elgueda^3^, Jonathan Fritz^4^

#### ^1^University of Melbourne, Department of Biomedical Engineering, Melbourne, Australia; ^2^University of Maryland, Institute for Systems Research, College Park, Maryland, United States of America; ^3^Universidad de Chile, Facultad de Ciencias Veterinarias y Pecuarias, Santiago, Chile; ^4^New York University, Center for Neural Science, New York, United States of America

##### **Correspondence:** Jordan Chambers (jordanc@unimelb.edu.au)

*BMC Neuroscience* 2020, **21(Suppl 1)**:P60

Neurons in the primary auditory cortex (A1) display rapid task-related plasticity, which is believed to enhance the ability to selectively attend to one stream of sound in complex acoustic scenes. Previous studies have suggested that cholinergic projections from Nucleus Basalis to A1 modulate auditory cortical responses and may be a key component of rapid task related plasticity. However, the underlying molecular, cellular and network mechanisms of cholinergic modulation of cortical processing remain unclear.

A previously published model of A1 receptive fields [1] that can reproduce task-related plasticity was used to investigate mechanisms of cholinergic modulation in A1. The previous model comprised a cochlea model and integrate-and-fire model neurons to represent networks in A1. Action potentials from individual model neurons were used to calculate the receptive field using reverse correlation, which allowed direct comparison to experimental data. To allow an investigation into different mechanisms of cholinergic modulation at A1, this previous model was extended by: (1) adding integrate-and-fire neurons to represent neurons projecting from Nucleus Basalis to A1; (2) adding inhibitory interneurons in A1; (3) including internal calcium dynamics in the integrate-and-fire models; and (4) including calcium-dependent potassium conductance in the integrate-and-fire models. Since cholinergic modulation has several potential sites of action in A1, the current model was used to investigate acetylcholine acting through both muscarinic and nicotinic acetylcholine receptors (mAChR and nAChR, respectively) located presynaptically or postsynaptically.

Four possible mechanisms of cholinergic modulation on A1 receptive fields were investigated. Previous research indicates cholinergic modulation should be able to suppress an inhibitory region and enhance an excitatory region in the receptive fields [2]. Our model indicates it is unlikely that any one of these four mechanisms could produce these opposite changes to both excitatory and inhibitory regions. However, multiple mechanisms occurring simultaneously could produce the expected changes to the receptive fields in this model. We demonstrate that combining either presynaptic nAChR with presynaptic mAChR or presynaptic nAChR with postsynaptic nAChR is capable of producing changes to A1 receptive fields observed during rapid task-related plasticity.

This model tested four mechanisms by which cholinergic modulation may induce rapid task-related plasticity in A1. Cholinergic modulation could reproduce experimentally observed changes to A1 receptive fields when it was implemented using a combination of mechanisms. Two different combinations of cholinergic modulation were found to produce the expected changes in A1 receptive fields. Since the model predicts that these two different combinations of cholinergic modulation would have differential effects on the rate of neuronal firing, it will be possible to run experimental tests to distinguish between the two theoretic possibilities.

**References**

1. Chambers JD, et al. Computational Neural Modeling of Auditory Cortical Receptive Fields. Frontiers in Computational Neuroscience. 2019; 13: 28.
2. Fritz J, Shamma S, Elhilali M, Klein D. Rapid task-related plasticity of spectrotemporal receptive fields in primary auditory cortex. Nature neuroscience. 2003; 6(11): 1216-23.

## P61 Self-supervision mechanism of multiple dendritic compartments for temporal feature learning

### Milena M Carvalho^1^, Tomoki Fukai^2^

#### ^1^University of Tokyo, Department of Complexity Science and Engineering, Tokyo, Japan; ^2^Okinawa Institute of Science and Technology Graduate University, Neural Coding and Brain Computing Unit, Okinawa, Japan

##### **Correspondence:** Milena M Carvalho (glowingsea@gmail.com)

*BMC Neuroscience* 2020, **21(Suppl 1)**:P61

In recent years, there has been a surge of interest in how dendrites and their complex geometry allow for integration of spatiotemporal patterns of input and transformation into neuronal response [1,2]. Although sizeable amounts of synaptic input arrive at distinct branches, the dendritic tree must convert this composite signal into meaningful information, which will be then transferred to the soma and potentially evoke spiking activity [3]. While there has been considerable development in dendritic integration modeling in the last two decades, single-compartment neurons are still a hallmark of computational neuroscience and machine learning [2]; this level of abstraction, however, ignores components of dendritic integration that may be essential to properly represent neuronal dynamics, hence limiting the performance of the studied systems.

Some of these shortcomings were recently overcome by a two-compartment model that introduced a self-supervision rule within a single neuron to minimize information loss between dendritic synaptic input and somatic output spiking activity [4]. Networks composed of this neuron model could perform a variety of unsupervised temporal feature learning tasks such as chunking and blind source separation, usually performed by specialized networks with different learning rules. We wish to generalize this learning principle and develop a new framework in which dendritic trees have two or more compartments with hierarchical, linear-nonlinear integrations [3]. Here we investigate how can self-supervision be defined in this system and examine its accuracy when presented to the previously introduced temporal feature learning tasks. We expect that, by distributing the synaptic input into different compartments of the same neuron, our model can use delayed integration to differentiate similar temporal patterns that were previously indistinguishable.

**Acknowledgments**: This work was partly supported by KAKENHI (nos. 18H05213 and 19H04994). We would like to thank the Ministry of Education, Culture, Sports, Science and Technology (MEXT) of Japan and Okinawa Institute of Science and Technology Graduate University (OIST) for supporting M. M. C.

**References**

1. Stuart GJ, Spruston N. Dendritic integration: 60 years of progress. Nature neuroscience. 2015; 18(12): 1713-21.
2. Häusser M, Mel B. Dendrites: bug or feature? Current Opinion in Neurobiology. 2003; 13(3): 372-383.
3. Ujfalussy BB, Makara JK, Lengyel M, Branco T. Global and Multiplexed Dendritic Computations under In Vivo-like Conditions. Neuron. 2018; 100(3): 579 - 592.e5
4. Asabuki T, Fukai T. Somatodendritic consistency check for temporal feature segmentation. Nature Communications. 2020; 11: 1554.

## P62 Large scale discrimination between neural models and experimental data

### Russell Jarvis^1^, Sharon Crook^2^, Richard Gerkin^3^

#### ^1^Arizona State University, Neuroscience, Tempe, Arizona, United States of America; ^2^Arizona State University, School of Mathematical and Statistical Sciences, Tempe, Arizona, United States of America; ^3^Arizona State University, School of Life Sciences, Tempe, Arizona, United States of America

##### **Correspondence:** Russell Jarvis (rjjarvis@asu.edu)

*BMC Neuroscience* 2020, **21(Suppl 1)**:P62

Scientific insight is well-served by the discovery and optimization of abstract models that can reproduce experimental findings. NeuroML (NeuroML.org), a model description language for neuroscience, facilitates reproducibility and exchange of such models by providing an implementation-agnostic model description in a modular format. NeuronUnit (neuronunit.scidash.org) evaluates model accuracy by subjecting models to experimental data-driven validation tests, a formalization of the scientific method.

A neuron model that perfectly imitated real neuronal electrical behavior in response to any stimulus would not be distinguishable from experiments by any conventional physiological measurement. In order to assess whether existing neuron models approached this standard, we took 972 existing neuron models from NeuroML-DB.org and subjected them to a standard series of electrophysiological stimuli (somatic current injection waveforms). We then extracted analogous 448 stimulus-evoked recordings of real cortical neurons from the Allen Cell Types database. We applied multiple feature extraction algorithms on the physiological responses of both model simulations and experimental recordings in order to characterize physiological behavior with a very high degree of detail spanning hundreds of features.

After applying dimensionality reduction to this very high dimensional feature space, we show that the real (biological neurons) and simulated (model neurons) recordings are easily and fully discriminated by eye or any reasonable classifier. Consequently, not a single model neuron produced physiological responses that could be confused with a biological neuron. Was this a defect of the model design (e.g. key mechanisms unaccounted for) or of model parameterization? We found that if we introduced models that were revised via optimization the revised models overlapped with the distribution of biological neurons, and were mostly classified as such. The remaining post-optimization disagreement between models and biological neurons may reflect limitations of model design and can be investigated by probing the key features used by classifiers to distinguish these two populations.

## P63 Morphological determinants of neuronal dynamics and function

### Christoph Kirch^1^, Leonardo Gollo^2^

#### ^1^QIMR Berghofer, Brisbane, Australia; ^2^Monash University, Melbourne, Australia

##### **Correspondence:** Christoph Kirch (ckirc4@gmail.com)

*BMC Neuroscience* 2020, **21(Suppl 1)**:P63

Neurons use their complex dendritic tree to integrate complex spatio-temporal patterns of incoming signals. The nonlinear interactions of spikes along the bifurcating branches allows the neuron to perform dendritic computations [1]. While models often aim to realistically simulate the complicated chemical properties of these signals, they often do this at the cost of simplifying the spatial structure to one (e.g., Hodgkin Huxley, integrate-and-fire) [2] or few compartments [3]. These simplified structures do not accurately represent the morphology, and thus it is not possible to infer how the dendritic structure can shape a neuron’s output.

Here, we used detailed neuron reconstructions from the online database NeuroMorpho.Org [4]. Each neuron is made up of one somatic compartment and up to 10,000 dendritic compartments. By treating these compartments as an excitable network [5, 6], we could apply a simple discrete model of dendritic spike propagation to investigate how the morphology affects the firing behavior of a neuron.

Our approach allows for a detailed analysis of the neuron’s dendritic activity pattern. For example, we can generate spatial heatmaps of firing rate, revealing a significant spatial dependence of dynamics. By comparing the compartmental activity for different strengths of external stimulus, we can investigate the dynamic range – over what range of input strength a compartment’s firing rate is varied the most. We find that dendritic bifurcations boost the local dynamic range. Thus, a soma located in densely bifurcated regions tends to have large dynamic ranges.

Identifying how effectively a neuron utilizes its dendritic tree to amplify stimuli can be achieved by comparing the average dendritic compartment activity against how often the soma fires. Since it takes energy to control the ion channels responsible for dendritic spikes, we call this ratio the relative energy consumption of the neuron. If it is <1, the neuron is energy efficient. Conversely, if it is >1, the neuron is inefficient. We identified two morphological features – the number of somatic branches, and the centrality of the soma – that can be used to categorize the energy behavior of neurons (Fig. 1).

**Fig. 1:**
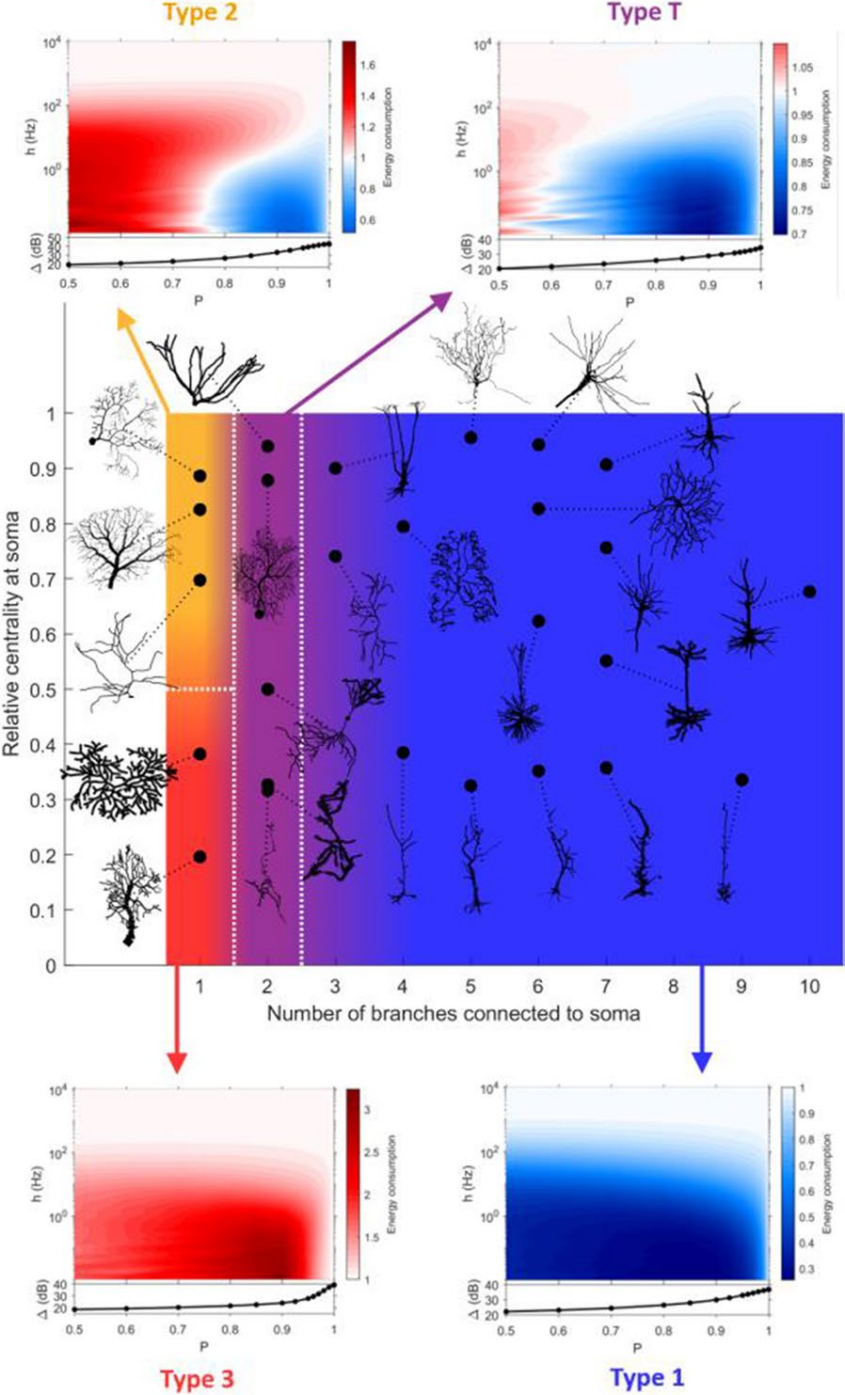
Neurons can be classified into functional groups based on two morphological features; the number of branches connected to the soma, and its centrality. Type 1 corresponds to energy efficient behaviour (energy < 1) across the parameter space of h and P. Type 2 exhibits mixed behaviour. Type 3 neurons are inefficient (energy > 1). Type T represents a transitional category with mixed behaviour

The classification scheme we have proposed provides an important testable basis in explaining structural differences across neurons. For example, depending on the required computational function, certain features of the dendritic tree would be more favorable. Our model can be applied to any of the 100,000+ reconstructions available at NeuroMorpho.Org, and can be extended to investigate the effect of changes in dendritic structure.

**References**

1. London M, Häusser M. Dendritic computation. Annual Review of Neuroscience. 2005; 28: 503-32.
2. Izhikevich EM. Simple model of spiking neurons. IEEE Transactions on Neural Networks. 2003; 14(6): 1569-72.
3. Izhikevich EM. Dynamical systems in neuroscience. MIT press; 2007.
4. Kirch C, Gollo LL. Spatially resolved dendritic integration: Towards a functional classification of neurons. bioRxiv. 2020; 657403.
5. Gollo LL, Kinouchi O, Copelli M. Active dendrites enhance neuronal dynamic range. PLoS Computational Biology. 2009; 5(6): e1000402.
6. Gollo LL, Kinouchi O, Copelli M. Statistical physics approach to dendritic computation: The excitable-wave mean-field approximation. Physical Review E. 2012; 85(1): 011911.

## P64 Gamma oscillations organized as localized burst patterns with anomalous propagation dynamics in primate cerebral cortex

### Xian Long^1^, Yuxi Liu^1^, Paul R Martin^2^, Samuel G Solomon^3^, Pulin Gong^1^

#### ^1^University of Sydney, School of physics, Sydney, Australia; ^2^University of Sydney, Save Sight Institute, Sydney, Australia; ^3^University College London, Department of Experimental Psychology, London, United Kingdom

##### **Correspondence:** Xian Long (longxian319@hotmail.com)

*BMC Neuroscience* 2020, **21(Suppl 1)**:P64

Gamma oscillations (30-80 Hz) occur in transient bursts with varying frequencies and durations. These non-stationary gamma bursts have been widely observed in many brain areas but have rarely been quantitatively characterized, and the mechanisms that produce them are not understood. In this study we investigate the spatiotemporal properties of gamma bursts through combined empirical and modeling investigation. Our array recordings of local field potentials in visual cortical area MT of the marmoset monkey reveal that gamma bursts form localized patterns with complex propagation dynamics. We also show that the propagations of these patterns are characterized by anomalous dynamics that are fundamentally different from regular or Brownian motions conventionally assumed. We show that all aspects of these anomalous dynamics can be quantitatively captured by a spatially extended, biophysically realistic circuit model. Circuit dissection of the model shows further that the anomalous dynamics rely on the intrinsic meta-stability near the critical transition between different circuit states (i.e. between synchronous and the regular propagating wave states). Our results thus reveal novel spatiotemporal organization properties of gamma bursts, and explain them in terms of underlying circuit mechanisms, providing new computational functions for gamma oscillations.

## P65 Dynamical circuit mechanisms of attentional sampling

### Guozhang Chen, Pulin Gong

#### The University of Sydney, School of Physics, Sydney, Australia

##### **Correspondence:** Guozhang Chen (gche4213@uni.sydney.edu.au)

*BMC Neuroscience* 2020, **21(Suppl 1)**:P65

Selective attention can sift out particular objects or features from the plethora of stimuli. Such preferential processing of attention is often compared to a spotlight pausing to illuminate relevant targets in visual fields in a stimulus-driven way (bottom-up attention) and/or task-driven way (top-down attention). Recent studies have revealed that bottom-up distributed attention involving multiple objects is not a sustained spotlight, but samples the visual environment in a fundamentally dynamical manner with theta-rhythmic cycles, with each sampling cycle being implemented through gamma oscillations. However, the fundamental questions regarding the dynamical nature and the circuit mechanism underlying such dynamical attentional sampling remain largely unknown. To address these questions, in this study we investigate a biophysically plausible cortical circuit model of spiking neurons and find that in the working regime of the model (i.e. the regime near the critical transition between the asynchronous and propagating wave states), the localized activity pattern emerging from the circuit exhibits rich spatiotemporal dynamics. We illustrate that the nonequilibrium nature of the localized pattern enables the circuit to dynamically shift to different salient external inputs, without introducing additional neural mechanisms such as inhibition of return as in the conventional winner-take-all models of attention. We elucidate that the dynamical shifting process of the activity pattern provides a mechanistic account of key neurophysiological and behavioral findings on attention, including theta oscillations, theta-gamma phase-amplitude coupling, and vigorous-faint spiking fluctuations. Furthermore, by using the saliency maps of natural stimuli, we demonstrate that the nonequilibrium activity pattern dynamics can better explain the psychophysical findings regarding attention maps and attention sampling paths than the conventional models, providing a profound computational advantage for efficiently sampling external environments. Our work thus establishes a novel circuit mechanism by which non-equilibrium, fluctuating pattern dynamics near the critical transition of circuit states can be exploited for implementing efficient attentional sampling.

## P66 Brain-computer interfaces using stereotactic electroencephalography: identification of discriminative recording sites for decoding imagined speech

### Kevin Meng^1^, David Grayden^1^, Mark Cook^2^, Farhad Goodarzy^3^

#### ^1^University of Melbourne, Department of Biomedical Engineering, Melbourne, Australia; ^2^University of Melbourne, Department of Medicine, Melbourne, Australia; ^3^University of Melbourne, Department of Medicine, Dental and Health Sciences, Melbourne, Australia

##### **Correspondence:** Kevin Meng (ksmeng@student.unimelb.edu.au)

*BMC Neuroscience* 2020, **21(Suppl 1)**:P66

As part of the monitoring of medication-resistant epilepsy before resective surgeries, patients are implanted with electrocorticography (ECoG) electrode arrays placed on the surface of the cortex or stereotactic electroencephalography (SEEG) depth electrodes penetrating the cortex. Both recording modalities measure local field potentials (LFPs) from their respective target locations. The patients are occasionally recruited to voluntarily participate in brain-computer interface (BCI) research. In recent years, ECoG-based BCIs have demonstrated long-term reliable decoding of various cortical processes involved in mental imagery tasks. Despite similarities in terms of clinical application and decoding strategies, SEEG-based BCIs have been the focus of only a limited number of studies. While the sparsity of their cortical coverage represents a disadvantage, SEEG depth electrodes have the potential to target bilateral combinations of deeper brain structures that are inaccessible with ECoG [1].

Here, we propose a framework for SEEG-based BCIs to identify discriminative recording sites for decoding imagined speech. Four patients with epilepsy were implanted with 7 to 11 SEEG depth electrodes, each consisting of 8 to 15 recording sites. Electrode placement and duration of monitoring were solely based on the requirements of clinical evaluation. Signals were amplified and recorded at a sampling rate of 5 kHz. The task consisted of listening to utterances and producing overt and covert (imagined) utterances of a selection of 20 monosyllabic English words made up of all combinations of five consonant patterns (/b_t/, /m_n/, /r_d/, /s_t/, /t_n/) and four vowels (/æ/, /ε/, /i:/, /u:/).

We determined the relative importance of recording sites based on classification accuracies obtained from features extracted at the corresponding electrode locations. Each trial was associated with a label (consonant pattern or vowel) and a set of features consisting of normalized log-transformed power spectral densities at different time points and selected frequency bands: delta (1-4 Hz), theta (4-8 Hz), alpha (8-12 Hz), beta (12-30 Hz), gamma 1 (30-45 Hz), gamma 2 (55-95 Hz), gamma 3 (105-145 Hz), and gamma 4 (155-195 Hz). A pair-wise classification model using logistic regression was used to predict the labels. Parameters were trained for different combinations of recording sites, as well as each condition (listening, overt, covert), patient, and pair of labels separately. The mean classification rate across all pairs of labels was calculated to quantify the discriminative power of individual and combined recording sites.

Our results consistently show across all patients that relevant depth electrodes for decoding imagined speech are found in both left and right superior temporal gyri. Anatomical analyses of these electrode locations revealed that recording sites in the grey matter were the most discriminative. This is in line with previous studies of speech BCIs [2]. In addition to providing a better understanding of the neural processes underlying imagined speech, our practical framework may be applied to reduce feature dimensionality and computational cost while improving accuracy in real-time SEEG-based BCI applications.

**References**

1. Herff C, Krusienski DJ, Kubben P. The Potential of Stereotactic-EEG for Brain-Computer Interfaces: Current Progress and Future Directions. Frontiers in Neuroscience. 2020; 14: 123.
2. Cooney C, Folli R, Coyle D. Neurolinguistics research advancing development of a direct-speech brain-computer interface. IScience. 2018; 8: 103-25.

## P67 A computational model to inform presurgical evaluation of epilepsy from scalp EEG

### Marinho Lopes^1^, Leandro Junges^2^, Luke Tait^3^, John Terry^2^, Eugenio Abela^4^, Mark Richardson^4^, Marc Goodfellow^5^

#### ^1^Cardiff University, Cardiff, United Kingdom; ^2^University of Birmingham, Centre for Systems Modelling and Quantitative Biomedicine, Birmingham, United Kingdom; ^3^Cardiff University, Cardiff University Brain Research Imaging Centre, Cardiff, United Kingdom; ^4^King’s College London, Institute of Psychiatry, Psychology and Neuroscience, London, United Kingdom; ^5^University of Exeter, Living Systems Institute, Exeter, United Kingdom

##### **Correspondence:** Marinho Lopes (m.lopes@exeter.ac.uk)

*BMC Neuroscience* 2020, **21(Suppl 1)**:P67

Epilepsy affects an estimated fifty million people worldwide. Approximately one third do not respond to anti-epileptic medication and are therefore potential candidates for alternative treatments such as epilepsy surgery. Surgery aims to remove the epileptogenic zone (EZ), the brain area responsible for the generation of seizures. Epilepsy surgery is thus preceded by an evaluation to determine the location of the EZ. A number of brain imaging modalities may be used in this evaluation, namely scalp electroencephalography (EEG) and magnetic resonance imaging (MRI), possibly followed by invasive intracranial EEG. The effectiveness of intracranial EEG to inform epilepsy surgery depends on where electrodes are implanted. This decision is informed by noninvasive recording modalities such as scalp EEG. The decision is frequently not trivial because scalp EEG may provide inconclusive or even contradictory predictions of the EZ location. A poor hypothesis based on noninvasive data may lead to an incorrect placement of intracranial electrodes, which in turn may make surgery ill-advised and potentially unsuccessful if performed [1].

Here we propose a framework to interrogate scalp EEG and determine epilepsy lateralization to aid in electrode implantation [2]. We used eLORETA to map source activities from seizure epochs recorded from scalp EEG and obtained functional networks using the phase-locking value (PLV). The networks were then studied using a mathematical model of epilepsy (a modified theta model to represent a network of interacting neural masses [2,3]). By removing different regions of interest from the network and simulating their impact on the network’s ability to generate seizures in silico, the framework provides predictions of epilepsy lateralization. We considered 15 individuals from the EPILEPSIAE database and studied a total of 62 seizures. Results were assessed by taking into account actual intracranial implantations and postsurgical outcome. The framework proved useful in assessing epilepsy lateralization in 12 out of 15 individuals considered. These results show promise for the use of this framework to better interrogate scalp EEG and aid clinicians in presurgical assessment of people with epilepsy.

**References**

1. Jayakar P, et al. Diagnostic utility of invasive EEG for epilepsy surgery: indications, modalities, and techniques. Epilepsia. 2016; 57(11): 1735-47.
2. Lopes MA, et al. Computational modelling in source space from scalp EEG to inform presurgical evaluation of epilepsy. Clinical Neurophysiology. 2020; 131(1): 225-34.
3. Lopes MA, et al. An optimal strategy for epilepsy surgery: Disruption of the rich-club?. PLoS Computational Biology. 2017; 13(8) :e1005637.

## P68 Large-scale calcium imaging of spontaneous activity in larval zebrafish reveals signatures of criticality

### Michael McCullough^1^, Robert Wong^1^, Zac Pujic^1^, Biao Sun^1^, Geoffrey J Goodhill^1,2^

#### ^1^University of Queensland, Queensland Brain Institute, St Lucia, Queensland, Australia; ^2^University of Queensland, School of Mathematics and Physics, St Lucia, Queensland, Australia

##### **Correspondence:** Geoffrey J Goodhill (g.goodhill@uq.edu.au)

*BMC Neuroscience* 2020, **21(Suppl 1)**:P68

Neural networks in the brain may self-organise such that they operate near criticality, that is, poised on the boundary between phases of order and disorder [1]. Models of neural networks tuned close to criticality are optimal in terms of dynamic range, information transmission, information storage and computational adaptability [2]. Most experimental evidence for criticality in the brain has come from studies of high resolution neural spiking data recorded from tissue cultures or anaesthetised animals using microelectrode arrays, or from studies of mesoscopic-scale neural activity using magnetic resonance imaging or electroencephalograms. These approaches are inherently limited either by under-sampling of the neural population or by coarse spatial resolution. This can be problematic for empirical studies of criticality because the characteristic dynamics of interest are theoretically scale-free.

Recently, Ponce-Alvarez et al. [3] investigated the larval zebrafish as a new model for neural criticality by utilising the unique properties of the organism that enable whole-brain imaging of neural activity in vivo and without anaesthetic. They identified hallmarks of neural criticality in larval zebrafish using 1-photon calcium imaging and voxel-based analysis of neuronal avalanches. Here we addressed two key limitations of their study by instead using 2-photon calcium imaging to observe truly spontaneous activity, and by extracting neural activity time series at single-cell resolution via state-of-the-art image segmentation [4]. Our data comprise fluorescence time series for large populations of neurons from 3-dimensional volumetric recordings of spontaneous activity in the optic tectum and cerebellum of larval zebrafish with pan-neuronal expression of GCaMP6s (n=5; approx. 10000 neurons per fish) (Fig. 1A). Neuronal avalanche statistics revealed power-law relationships and scale-invariant avalanche shape collapse which are consistent with crackling noise dynamics from a 3-dimensional random field Ising model [5] (Fig. 1B-C). Observed power laws were validated using shuffled surrogate data and log-likelihood ratio tests. This result provides the first evidence of criticality in the brain from large-scale in vivo neural activity at single cell resolution. Our findings demonstrate the potential of larval zebrafish as a model for the investigation of critical phenomena in the context of neurodevelopmental disorders that may perturb the brain away from criticality.

**Fig. 1:**
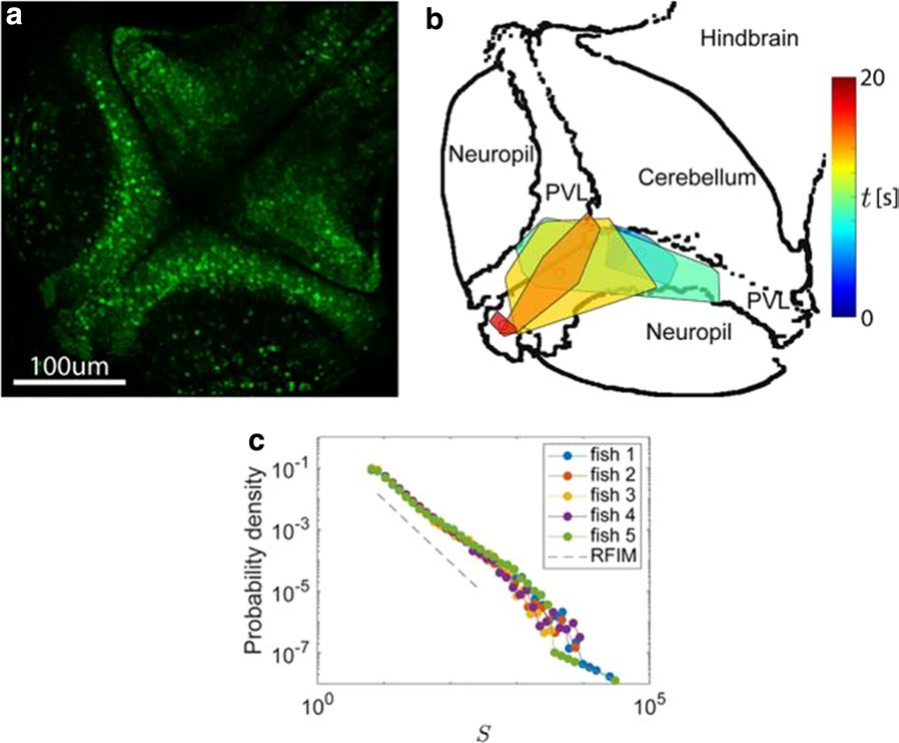
**A** z-projection of 2-photon data from a larval zebrafish. **B** The spatio-temporal profile of a neuronal avalanche (clusters of neural activations that propagate through the brain, as shown by shaded regions). **C** Avalanche statistics reveal characteristic power law relationships including the distribution of avalanche size (shown) consistent with a random field Ising model (dashed line)

**References**

1. Cocchi L, Gollo LL, Zalesky A, Breakspear M. Criticality in the brain: A synthesis of neurobiology, models and cognition. Progress in Neurobiology. 2017; 158: 132–152.
2. Shew WL, Plenz D. The functional benefits of criticality in the cortex. Neuroscientist. 2013; 19(1): 88–100.
3. Ponce-Alvarez A, et al. Whole-brain neuronal activity displays crackling noise dynamics. Neuron. 2018; 100(6): 1446–1459.
4. Giovannucci A, et al. CaImAn an open source tool for scalable calcium imaging data analysis. Elife. 2019; 8: e38173.
5. Sethna JP, Dahmen KA, Myers CR. Crackling noise. Nature. 2001; 410(6825): 242-250.

## P69 Population model of oscillatory dynamics in hippocampal CA1 and CA3 regions

### Ashraya S Shiva, Bruce Graham

#### University of Stirling, Department of Computing Science and Mathematics, Stirling, United Kingdom

##### **Correspondence:** Ashraya S Shiva (asv@cs.stir.ac.uk)

*BMC Neuroscience* 2020, **21(Suppl 1)**:P69

Theta oscillations may act as carrier waves for synchronizing activities across neuronal regions. Several gamma cycles, each containing a specific pattern of activity correlated with an event e.g. animal location forming place fields, are encompassed in a single theta cycle. We have extended the septo-hippocampal population firing rate model proposed by Denham and Borisyuk [1] to study the influence of inhibitory interneurons, specifically PV-containing basket cells (BCs) and bistratified cells (BSCs) on theta and theta-coupled gamma oscillations in both CA1 and CA3 hippocampal networks. Our CA1 microcircuit model is a combination of that of Denham and Borisyuk and Cutsuridis et. al [2]. The CA3 model is adapted from the CA1 model on the basis of CA3-specific experimental data.

The theta-phase relationships of the neurons in these models are largely determined by the afferent and efferent connections of BCs in both CA1 and CA3. In CA1, BCs and BSCs are active on opposite phases of theta in a pure-theta tuning (no gamma), as per data from Klausberger et. al [3,4] due to extra external drive to basket cells from CA3, enabling them to be active on the opposite cycle to CA1 pyramidal cells (PCs). As excitatory drive to BCs from PCs is increased there is a bifurcation in which BC activity switches to being in-phase with PCs, and BSC activity remains in-phase with PCs. A further increase in drive to BCs from PCs leads to gamma oscillations in both PC and BC activity, and forces BSC activity to shift to the opposite theta phase due to strong inhibition from BCs.

Varying strengths of external inputs also affects the strength of oscillations and phase relationships. Both in CA1 and CA3, PCs, BCs and BSCs reach steady state activity for very low or very high external inputs. BCs in CA1 rely on CA3 and EC input for their activity in theta-only tuning, so their activity reduces if these inputs are reduced. BSCs activity in CA1 actually reduces for increased CA3 and EC input, due to increased inhibition from BCs. In CA3, recurrent connections between PCs are far more likely (and hence stronger on a population scale) than in CA1. BSCs in CA3 are driven only by CA3 PCs and have no external sources of excitation. BCs in CA3 get dentate gyrus input in addition to PC input. Other interneurons (modeled as a single population) get excitation from CA1 and CA3 PCs, plus EC. In CA1, only CA3 and EC are external sources of input, apart from septum. The resultant main difference in activity in CA3, compared with CA1, is that BSCs are always in sync with BCs and PCs during theta. A minimum strength of septum input is required to generate theta, then increasing septum input raises the frequency of theta oscillations. Whereas, increasing the value of dentate gyrus input transforms the oscillatory activity into stable, non-oscillatory activity, and also decreases the septum activity. Strong EC and CA1 input to CA3 may silence CA3 PCs, BCs and BSCs through exciting other inhibitory interneurons.

**References**

1. Denham MJ, Borisyuk RM. A model of theta rhythm production in the septal‐hippocampal system and its modulation by ascending brain stem pathways. Hippocampus. 2000; 10(6): 698-716.
2. Cutsuridis V, Cobb S, Graham BP. Encoding and retrieval in a model of the hippocampal CA1 microcircuit. Hippocampus. 2010; 20(3): 423-46.
3. Klausberger T, et al. Brain-state-and cell-type-specific firing of hippocampal interneurons in vivo. Nature. 2003; 421(6925): 844-8.
4. Klausberger T, et al. Spike timing of dendrite-targeting bistratified cells during hippocampal network oscillations in vivo. Nature Neuroscience. 2004; 7(1): 41-7.

## P70 Influence of anatomical connectivity and intrinsic dynamics in a connectome based neural mass model of TMS-evoked potentials

### Neda Kaboodvand^1^, John Griffiths^2^

#### ^1^Karolinska Institutet, Department of Clinical Neuroscience, Stockholm, Sweden; ^2^Centre for Addiction and Mental Health, Krembil Centre for Neuroinformatics, Toronto, Canada

##### **Correspondence:** John Griffiths (j.davidgriffiths@gmail.com)

*BMC Neuroscience* 2020, **21(Suppl 1)**:P70

Perturbation via electromagnetic stimulation is a powerful way of probing neural systems to better understand their functional organization. One of the most widely used neurostimulation techniques in human neuroscience is transcranial magnetic stimulation (TMS) with concurrently recorded electroencephalography (EEG). The immediate EEG responses to single-pulse TMS stimulation, termed TMS-evoked potentials (TEPs), are spatiotemporal waveforms in EEG sensor- or source-space [1]. TEPs display several characteristic features, including i) rapid wave-like propagation away from the primary stimulation site, and ii) multiple volleys of recurrent activity, that continue for several hundred milliseconds following the stimulation pulse. These TEP patterns reflect reverberant activity in large-scale cortico-cortical and cortico-subcortical brain networks, and have been used to study neural excitability in a wide variety of research contexts, including sleep, anaesthesia, and coma [2]. There has been relatively little work done, however, on computational modelling of TEP waveform morphologies, and how these spatiotemporal patterns emerge from a combination of global brain network structure and local physiological characteristics. Here we present a novel connectome-based neural mass model of TEPs that accurately reproduces recordings across multiple subjects and stimulation sites. We employ a biophysical electric field model (using the simnibs [3] library) to identify the electrical field (‘E-field’) distribution over the cortical surface resulting from stimulation at a given TMS coil location and orientation, that is based on T1-weighted MRI-derived cortical geometry, and personalized to individual subjects. These TMS-induced E-field maps are then summed to yield a current injection pattern over regions in a canonical freesurfer-based brain parcellation. Whole-brain neural activity is modelled with a network of oscillatory (Fitzhugh-Nagumo) units [4,5], coupled by anatomical connectivity weights derived from diffusion-weighted MRI tractography [6], and perturbed by a brief square-wave current injection weighted regionally by the cortical E-field map magnitudes. Using this model we are able to accurately reproduce the typical radially propagating TEP patterns under a wide range of parameter values. For the later (150ms+) TEP components however, we find that it is necessary to modify the weight of cortico-thalamic and thalamo-cortical projections in the tractography-defined anatomical connectivity (see also [7]), which has the effect of promoting recurrent activity patterns. These results contribute important insights to our long-term objective of developing an accurate model of TEPs that can be used to guide the design and administration of TMS-EEG for excitability mapping in clinical contexts.

**References**

1. Ilmoniemi RJ, Kičić D. Methodology for combined TMS and EEG. Brain topography. 2010; 22(4): 233.
2. Massimini M, et al. Breakdown of cortical effective connectivity during sleep. Science. 2005; 309(5744): 2228-32.
3. Saturnino GB, et al. Simnibs 2.1: A comprehensive pipeline for individualized electric field modelling for transcranial brain stimulation. In Brain and Human Body Modeling 2019; 3-25. Springer, Cham.
4. Izhikevich EM, FitzHugh R. Fitzhugh-nagumo model. Scholarpedia. 2006; 1(9): 1349.
5. Spiegler A, Hansen EC, Bernard C, McIntosh AR, Jirsa VK. Selective activation of resting-state networks following focal stimulation in a connectome-based network model of the human brain. Eneuro. 2016; 3(5).
6. Schirner M, Rothmeier S, Jirsa VK, McIntosh AR, Ritter P. An automated pipeline for constructing personalized virtual brains from multimodal neuroimaging data. NeuroImage. 2015; 117: 343-57.
7. Bensaid S, Modolo J, Merlet I, Wendling F, Benquet P. COALIA: a computational model of human EEG for consciousness research. Frontiers in systems neuroscience. 2019; 13: 59.

## P71 Local homeostatic regulation of the spectral radius of echo-state networks

### Fabian Schubert^1^, Claudius Gros^2^

#### ^1^Goethe University Frankfurt, Institute for Theoretical Physics, Frankfurt am Main, Germany; ^2^Goethe University Frankfurt, Frankfurt am Main, Germany

##### **Correspondence:** Fabian Schubert (fschubert@itp.uni-frankfurt.de)

*BMC Neuroscience* 2020, **21(Suppl 1)**:P71

Recurrent cortical network dynamics plays a crucial role for sequential information processing in the brain. While the theoretical framework of reservoir computing provides a conceptual basis for the understanding of recurrent neural computation, it often requires manual adjustments of global network parameters, in particular of the spectral radius of the recurrent synaptic weight matrix. Being a mathematical and relatively complex quantity, the spectral radius is not readily accessible to biological neural networks, which are based on the principle that information about the network state should either be encoded in local intrinsic dynamical quantities (e.g. membrane potentials), or transmitted via synaptic connectivity. We present an intrinsic adaptation rule, termed *flow control*, for echo state networks that solely relies on locally accessible variables, while still being capable of tuning a global quantity, the spectral radius of the network, towards a desired value. The adaptation rule works online, in the presence of a continuous stream of input signals. It is based on a local comparison between the mean squared recurrent membrane potential and the mean squared activity of the neuron itself. It is derived from a global scaling condition on the dynamic flow of neural activities, and requires the separability between external and recurrent input currents. The effectiveness of the presented mechanism is tested numerically using different external input protocols. Furthermore, the network performance after applying the adaptation is evaluated by training the network to perform a time delayed XOR operation on binary sequences.

## P72 Comparison of surrogate techniques for evaluation of spatio-temporal patterns in massively parallel spike trains

### Alessandra Stella^1^, Peter Bouss^2^, Günther Palm^3^, Sonja Gruen^4^

#### ^1^Forschungszentrum Jülich, Jülich, Germany; ^2^Forschungszentrum Jülich, Institute of Neuroscience and Medicine (INM-6, INM-10, IAS-6), Jülich, Germany; ^3^University of Ulm, Institute of Neural Information Processing, Ulm, Germany; ^4^Jülich Research Centre, Institute of Neuroscience and Medicine (INM-6 and INM-10), Jülich, Germany

##### **Correspondence:** Alessandra Stella (a.stella@fz-juelich.de)

*BMC Neuroscience* 2020, **21(Suppl 1)**:P72

To identify active cell assemblies we developed a method to detect significant spatio-temporal spike patterns (STPs). The method, called SPADE [1-3], identifies repeating ms-precise spike patterns across neurons. SPADE first discretizes the spike trains in exclusive bins (defining the pattern precision, e.g. 5ms) and clips the bin content to 1 if more than 1 spike is therein. Second, STPs are mined by Frequent Itemset Mining [4], and their counts are evaluated for significance through comparison to surrogate data. The distribution of the pattern counts in the surrogate data provides p-values for determining the significance of grouped patterns. The surrogate data implement the null-hypothesis of independence, and a classical choice is to apply uniform dithering (UD) [5], i.e. independent, uniformly distributed displacement of each spike (e.g. in a range of +/- 5 times the bin width [1]). This approach does not maintain the absolute refractory period and a potentially existing ISI regularity. The binarization leads in the surrogates to a higher probability of more than 1 spike per bin, and thus by the consecutive clipping to a reduction of the spike count (up to 12%, in particular for high firing rates) as compared to the original data.

This may cause false positives (cmp. [6]). Therefore, we explored further methods for surrogate generation. To not have different spike counts in the original and the surrogate data, bin-shuffling shuffles the bins after binning the original data. To keep the refractory period (RP) uniform dithering with refractory period (UD-RP) does not allow dithered spikes within a short time interval after each spike. Dithering according to the ISI distribution (ISI-D) [e.g. 5] or the Joint-ISI distribution (J-ISI-D) [7] conserves the ISI and ISI/J-ISI distributions, respectively. Spike-train shifting (ST-Shift) [8,5] moves the whole spike train, trial by trial, by a random amount, thereby only affecting the relation of spike trains to each other. Thus all of these implement different null-hypotheses, as summarized in the table below. It shows the non-/preservation (no/yes) of features in the various surrogates compared to the original data.

We applied all surrogate methods (within SPADE) and compared their results using artificial, and experimental spike data simultaneously recorded in pre-/motor cortex of a macaque monkey performing a reach-to-grasp task [9]. We find that all methods besides UD lead to very similar results in terms of number of patterns and their composition. UD results in a much larger number of patterns, in particular if neurons have very high firing rates and exhibit regular spike trains. We conclude that the reduction in the spike count using UD increases the false positive rate for spike trains with CV<1 and/or high firing rates, the other methods are much less affected, the least spike train shifting.

**Table:** Table summarizing statistical properties of the spike trains being non-/preserved (no/yes) by the various surrogate techniques taken into consideration

Method/Feature	UD	ISI-D	J-ISI-D	UD-RP	Bin-Shuff	ST-shift
Spike count	no	yes	approx.	approx.	yes	yes
ISI	no	approx.	approx.	no	no	yes
J-ISI	no	no	approx.	no	no	yes

**References**

1. Torre E, et al. Synchronous spike patterns in macaque motor cortex during an instructed-delay reach-to-grasp task. Journal of Neuroscience. 2016; 36(32): 8329-40.
2. Quaglio P, Yegenoglu A, Torre E, Endres DM, Grün S. Detection and evaluation of spatio-temporal spike patterns in massively parallel spike train data with spade. Frontiers in Computational Neuroscience. 2017; 11: 41.
3. Stella A, Quaglio P, Torre E, Grün S. 3d-SPADE: Significance evaluation of spatio-temporal patterns of various temporal extents. Biosystems. 2019; 185: 104022.
4. Picado-Muiño D, Borgelt C, Berger D, Gerstein G, Grün S. Finding neural assemblies with frequent item set mining. Frontiers in Neuroinformatics. 2013; 7(9).
5. Louis S, Borgelt C, Grün S. Complexity distribution as a measure for assembly size and temporal precision. Neural Networks. 2010; 23(6): 705-12.
6. Pipa G, Grün S, Van Vreeswijk C. Impact of spike train autostructure on probability distribution of joint spike events. Neural Computation. 2013; 25(5): 1123-63.
7. Gerstein GL. Searching for significance in spatio-temporal firing patterns. Acta Neurobiologiae Experimentalis. 2004; 64(2): 203-8.
8. Pipa G, Wheeler DW, Singer W, Nikolić D. NeuroXidence: reliable and efficient analysis of an excess or deficiency of joint-spike events. Journal of computational neuroscience. 2008; 25(1): 64-88.
9. Brochier T, et al. Massively parallel recordings in macaque motor cortex during an instructed delayed reach-to-grasp task. Scientific data. 2018; 5(1): 1-23.

## P73 Effects of transneuronal spreading in the logistic diffusion model of Aβ protein with longitudinal PET scans

### Byeong Chang Jeong^1^, Myungwon Choi^2^, Daegyeom Kim^2^, Xue Chen^3^, Marcus Kaiser^4^, Cheol E Han^2^

#### ^1^Korea University, Sejong, South Korea; ^2^Korea University, Department of Electronics and Information Engineering, Sejong, South Korea; ^3^China University of Petroleum, College of Control Science and Engineering, Qingdao, China; ^4^Newcastle University, School of Computing, Newcastle, United Kingdom

##### **Correspondence:** Byeong Chang Jeong (ni3_jbc@naver.com)

*BMC Neuroscience* 2020, **21(Suppl 1)**:P73

Alzheimer’s disease (AD) is a neuro-degenerative disease which causes severe loss of cognitive functions and deteriorates the quality of daily life of elders. One hypothesis of AD development and progression is deposition of amyloid-beta protein which is known to cause neuronal cell death in the gray matter and to degrade the cognitive function of the affected regions. Recently, it is reported that the toxic protein can move through connectivity between neurons, which is called transneuronal projection, besides the local diffusion through the non-neuronal tissues. In this study, we investigated the effects of the transneuronal projection on the amyloid deposition pattern over the brain through simulation of a mathematical model based on the actual neuroimage data. The model consists of two components: transneuronal spreading, and local spreading. The transneuronal spreading captures propagation of the toxic protein in the white matter while the local spreading captures its local diffusion through the gray matter. Each component has its own parameter to balance between components. We estimated all parameters in the model through the Bayesian inference method that best describe the longitudinal data from Alzheimer’s disease neuroimaging initiative (ADNI) dataset, by comparing the results of simulation data with the actual dataset. We modelled our brain as a high-resolution graph whose nodes are a small volume of the cerebral cortices, and whose edges are the structure of adjacency between them, delineating spreading pathways. We transformed the cerebral cortices into a triangular lattice of prism-like volumes from structural magnetic resonance (MR) images. From the topology of the lattice we extracted local adjacency between the nodes. On the contrary, for the long-range connection through the neuronal fibers, we obtained the connectivity between the nodes from the diffusion-weighted MR imaging (DWI). Each node has the level of the amyloid deposition, obtained through the18F-Florbetapir positron emission tomography (PET) images with partial volume effect correction. This high-resolution graph model can enable to simulate more granularly and accurately. To investigate the effect of transneuronal spreading, we compared results of two conditions: simulation 1) with only the local spreading, and 2) with both the local and transneuronal spreading. Both models showed the spread of the amyloid from the regions where the initial protein accumulation is high, also known as epicenters. The result of the former condition may explain gradual spread from the epicenters to their nearby regions while it could not explain remote spread from the epicenters to their distant regions. On the other hand, the latter results illustrated not only local spread, but also remote spread. Thus, the model with both local and transneuronal spreading components is more feasible to explain the deposition of amyloid-beta in AD. The purpose of this study is to investigate the effect of transneuronal spread of the amyloid beta over AD’s progression using the actual neuroimage data. Our results support the previous research of transmission of amyloid through the neuronal pathways.

**Acknowledgements:** This work was supported by the Korea Health Technology R&D Project through the Korea Health Industry Development Institute (KHIDI) that was funded by the Ministry of Health & Welfare, Republic of Korea (HI19C0645), and Medical Research Council, UK (MR/T004347/1).

## P74 Implications of reduced somatostatin interneuron inhibition in depression on human cortical microcircuit activity

### Heng Kang Yao^1^, Alexandre Guet-McCreight^2^, Etay Hay^3^

#### ^1^University of Toronto, Physiology, Toronto, Canada; ^2^Centre for Addiction and Mental Health, Krembil Centre for Neuroinformatics, Toronto, Canada; ^3^Centre for Addiction and Mental Health/ University of Toronto, Krembil Centre for Neuroinformatics/ Psychiatry, Toronto, Canada

##### **Correspondence:** Heng Kang Yao (kant.yao@mail.utoronto.ca)

*BMC Neuroscience* 2020, **21(Suppl 1)**:P74

There is increasing evidence of reduced cortical inhibition in a variety of psychiatric disorders such as major depressive disorder (MDD) and schizophrenia. Cortical inhibition is mediated by GABAergic interneurons and plays a key role in modulating information processing in cortical pyramidal neurons. In particular, interneurons expressing somatostatin (SST) inhibit the distal dendrites of pyramidal neurons and mediate lateral inhibition. Recent postmortem studies showed reduced SST expression in these interneurons in MDD patients, suggesting weaker levels of inhibition. The reduced inhibition is thought to result in a lower signal-to-noise ratio of cortical microcircuit activity, due to abnormally increased intrinsic activity compared to stimulus-evoked activity. We test this hypothesis and characterize the implications of reduced SST inhibition in depression using novel computational models of human cortical microcircuits. We generated detailed models of the major neuronal types in human cortical layer 2/3, by integrating unique human electrophysiology data and applying machine-learning optimization algorithms. We connected the model neurons in a microcircuit according to connectivity statistics and synaptic parameters derived from the literature. We show that intrinsic activity significantly increases in models of MDD microcircuits, in which SST interneuron inhibition was reduced, compared to healthy microcircuits. We then then compared the signal-to-noise ratio of intrinsic and evoked activity in healthy and MDD microcircuits. Our results thus elucidate the role that inhibition plays in normal and pathological information processing by human cortical microcircuits.

## P75 The covariance perceptron: theory and application

### Matthieu Gilson^1^, David Dahmen^2^, Ruben Moreno-Bote^3^, Andrea Insabato^4^, Moritz Helias^5^

#### ^1^Universitat Pompeu Fabra, Center for Brain and Cognition, Barcelona, Spain; ^2^Jülich Research Centre, Institute of Neuroscience and Medicine (INM-6), Jülich, Germany; ^3^Universitat Pompeu Fabra, Center for Brain and Cognition & DTIC, Barcelona, Spain; ^4^Universty Pompeu Fabra, Barcelona, Spain; ^5^Jülich Research Centre, Institute of Neuroscience and Medicine (INM-6, INM-10) and Institute for Advanced Simulation (IAS-6), Jülich, Germany

##### **Correspondence:** Matthieu Gilson (matthieu.gilson@upf.edu)

*BMC Neuroscience* 2020, **21(Suppl 1)**:P75

**Introduction:** Many efforts in the study of the brain have focused on representations of stimuli by neurons and learning thereof. Our work [1] demonstrates the potential of a novel learning paradigm for neuronal activity with high variability, where distributed information is embedded in the correlation patterns.

**Learning theory:** We derive a learning rule to train a network to perform an arbitrary operation on spatio-temporal covariances for time series. To illustrate our scheme, we use the example of classification where the network is trained to perform an input-output mapping from given sets of input patterns to representative output patterns, one output per input group. This setup is the same as learning activity patterns for the classical perceptron [2], a central concept that has brought many fruitful theories in the fields of neural coding and learning in networks. For that reason, we refer to our classifier as “covariance perceptron”. Compared to the classical perceptron, a conceptual difference is that we base information on the the co-fluctuations of the input time series that result in second-order statistics. In this way, robust information can be conveyed despite a high apparent variability in the activity. This approach is a radical change of perspective compared to classical approaches that typically transform time series into a succession of static patterns where fluctuations are noise. On the technical ground, our theory relies on the multivariate autoregressive (MAR) dynamics, for which we derive the weight update (a gradient descent) such that input covariance patterns are mapped to given objective output covariance patterns.

**Application to MNIST database:** To further explore its robustness, we apply the covariance perceptron to the recognition of objects that move in the visual field by a network of sensory (input) and downstream (output) neurons. We use the MNIST database of handwritten digits 0 to 4. As illustrated in Figure 1, the traces “viewed” by an input neuron exhibit large variability across presentations. Because we want to identify both the digit identity and its moving direction, covariances of the input time series are necessary. We show that the proposed learning rule can successfully train the network to perform the classification task and robustly generalize to unseen data. In our work [1], we also show that the covariance perceptron favorably compares to the classical nonlinear perceptron in extracting second-order statistics.

**Fig. 1:**
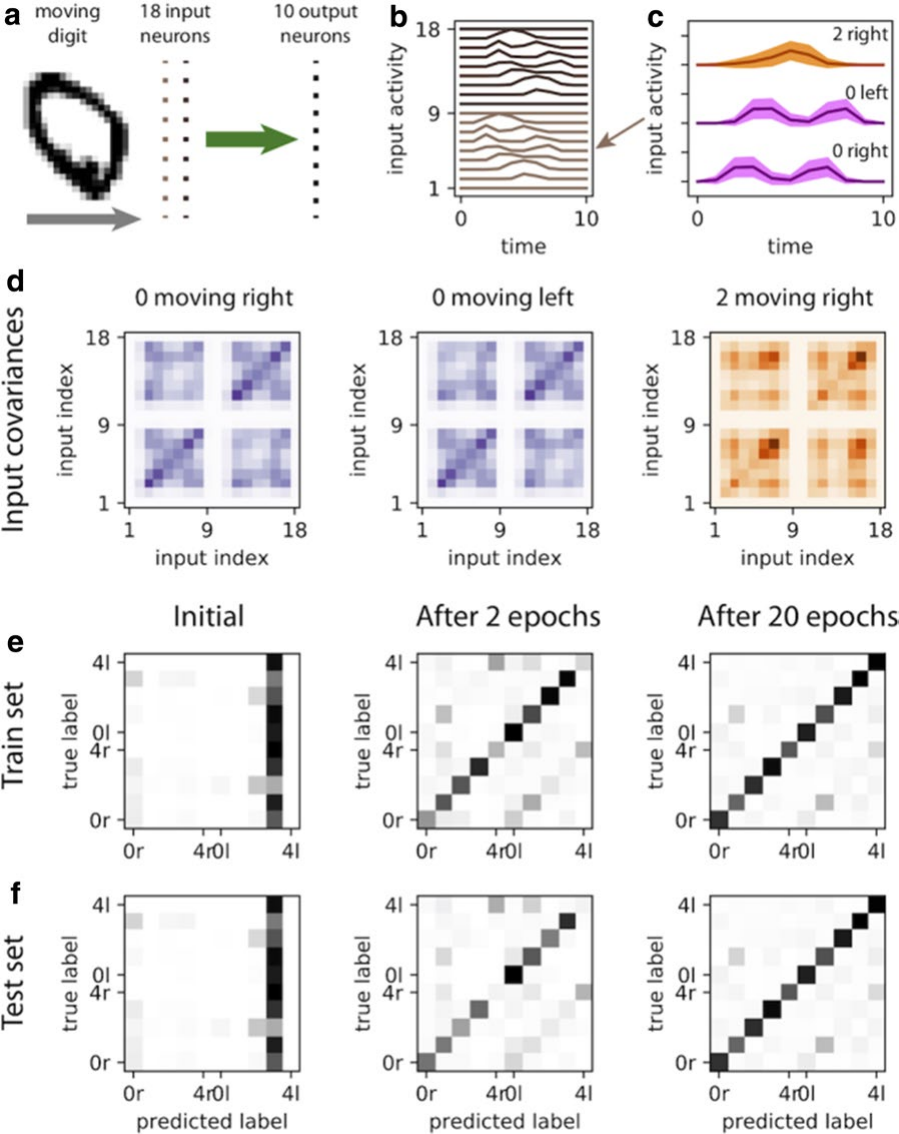
**A** Moving digit in the visual field with two columns of 9 input neurons each feeding 10 output neurons (one per category, the largest output variance indicates the predicted category). **B** Responses of the input to the digit 0 in panel A moving to the right. **C** Mean activity traces for an input neuron for digits 0 and 2, moving left or right as indicated above. The colored areas correspond to the standard deviation of the time-varying activity over all patterns. **D** The information relative to both digit and motion is reflected in the input covariances. **E** Confusion matrices of the covariance perceptron for the train and test sets for digits 0 to 4 moving left and right

**Towards distributed spike-based information processing:** We envisage future steps that transpose this work to information conveyed by high-orders in the spike trains, to obtain the supervised equivalent of spike-timing-dependent plasticity (STDP).

**References**

1. Gilson M, Dahmen D, Moreno-Bote R, Insabato A, Helias M. The covariance perceptron: A new framework for classification and processing of time series in recurrent neural networks. bioRxiv. 2019; 562546.
2. Bishop CM. Pattern recognition and machine learning. springer; 2006.

## P76 Inferring a simple mechanism for alpha-blocking by fitting a neural population model to EEG spectra

### Agus Hartoyo^1^, Peter Cadusch^2^, David Liley^3^, Damien Hicks^4^

#### ^1^Swinburne University of Technology, Optical Sciences Centre, Melbourne, Australia; ^2^Swinburne University of Technology, Department of Physics and Astronomy, Melbourne, Australia; ^3^Swinburne University of Technology, Centre for Human Psychopharmacology, Melbourne, Australia; ^4^Centre for Human Psychopharmacology, Optical Sciences Centre, Melbourne, Australia

##### **Correspondence:** Agus Hartoyo (ahartoyo@swin.edu.au)

*BMC Neuroscience* 2020, **21(Suppl 1)**:P76

Alpha blocking, a phenomenon where the alpha rhythm is reduced by attention to a visual, auditory, tactile or cognitive stimulus, is one of the most prominent features of human electroencephalography (EEG) signals. Here we identify a simple physiological mechanism by which opening of the eyes causes attenuation of the alpha rhythm. We fit a neural population model to EEG spectra from 82 subjects, each showing different degrees of alpha blocking upon opening of their eyes. Although it is notoriously difficult to estimate parameters from fitting such models, we show that, by regularizing the differences in parameter estimates between eyes-closed and eyes-open states, we can reduce the uncertainties in these differences without significantly compromising fit quality. From this emerges a parsimonious explanation for the spectral changes between states: just a single parameter, pei, corresponding to the strength of a tonic, excitatory input to the inhibitory population, is sufficient to explain the reduction in alpha rhythm upon opening of the eyes. When comparing parameter estimates across different subjects we find that the inferred differential change in pei for each subject increases monotonically with the degree of alpha blocking observed. In contrast, other parameters show weak or negligible differential changes that do not scale with the degree of alpha attenuation in each subject. Thus, most of the variation in alpha blocking across subjects can be attributed to the strength of a tonic afferent signal to the inhibitory cortical population.

## P77 Tonic GABAergic inhibition enhances activity-dependent dendritic calcium signaling

### Thomas M Morse^1^, Chiayu Q Chiu^2^, Francesca Nani^3^, Frederic Knoflach^3^, Maria-Clemencia Hernandez^3^, Monika Jadi^4^, Michael J Higley^1^

#### ^1^Yale University, Department of Neuroscience, Kavli Institute of Neuroscience, New Haven, Connecticut, United States of America; ^2^Universidad de Valparaiso, Centro Interdisciplinario de Neurociencia de Valparaiso, Valparaiso, Chile; ^3^Roche Innovation Center, Roche Pharmaceutical Research and Early Development, Neuroscience and Rare Diseases, Basel, Switzerland; ^4^Yale University, Department of Psychiatry, New Haven, Connecticut, United States of America

##### **Correspondence:** Thomas M Morse (tom.morse@yale.edu)

*BMC Neuroscience* 2020, **21(Suppl 1)**:P77

Brain activity is highly regulated by GABAergic activity, which acts via GABA type A receptors (GABAARs) to suppress somatic spike generation as well as dendritic synaptic integration and calcium signaling. Tonic GABAergic conductances mediated by distinct receptor subtypes can also inhibit neuronal excitability and spike output, though the consequences for dendritic calcium signaling are unclear. Here, we use 2-photon calcium imaging in cortical pyramidal neurons and computational modeling to show that low affinity GABAARs containing an alpha5 subunit mediate a tonic hyperpolarization of the dendritic membrane potential, resulting in deinactivation of voltage-gated calcium channels and a paradoxical boosting of action potential-evoked calcium influx. We also find that GABAergic enhancement of calcium signaling modulates short-term synaptic plasticity, augmenting depolarization-induced suppression of inhibition. These results demonstrate a novel role for GABA in the control of dendritic activity and suggest a mechanism for differential modulation of electrical and biochemical signaling.

**Acknowledgements:** The authors wish to thank members of the Higley laboratory and Dr. Jessica A. Cardin for helpful comments, the Yale Center for Research Computing for support with the Yale Farnam high performance cluster, Henner Knust for compound synthesis, Chiristian Miscenic and Marcello Foggetta for cell transfections and membrane preparations, Judith Lengyel, Gregoire Friz, and Maria Karg for cell line generation and radioligand binding assays, and Marie Claire Pflimlin for support with electrophysiological characterization of Compound A selectivity. This work was supported by funding from the NIH/NIMH (R01 MH099045 and MD113852 to MJH, K01 MH097961 to CQC), funding agencies in Chile (FONDECYT No. 1171840 and MILENIO PROYECTO P09-022-F, CINV to CQC), and Roche Pharmaceutical.

## P78 Phasic response of epileptic models

### Alberto P Cervera, Jaroslav Hlinka

#### Institute of Computer Science - Czech Academy of Sciences, Prague, Czechia

##### **Correspondence:** Alberto P Cervera (perez@cs.cas.cz)

*BMC Neuroscience* 2020, **21(Suppl 1)**:P78

Although epilepsy is the most chronic neurological disorder, the mechanisms underlying the initiation of epileptic seizure remain unknown. Epileptic seizures are generated by intense activity emerging from a highly synchronized neuronal population. These phenomena are usually preceded and followed by intervals of reduced activity, known as interictal periods. Importantly, the transient neuronal activity during these interictal periods -known as interictal epileptiform discharges (IEDs) - is considered a key mechanism governing the transition to seizure. However, whether IEDs prevent or facilitate that transition is still a matter of debate.

In this work, based on previous findings in [1], we show how these dual effects for IEDs can be interpreted in terms of the phasic response of a slow-fast system. Indeed, since the phase response of a given system follows from its isochrons distribution, we perform a theoretical and computational study of the isochrons and phase response curves of different planar slow-fast epileptic models. Our results unfold the strong influence of the slow vector field in the phasic response of the system to IEDs and suggest theoretical strategies whose effects range from the short delay to the full suppression of seizures.

**Reference**

1. Chang WC, et al. Loss of neuronal network resilience precedes seizures and determines the ictogenic nature of interictal synaptic perturbations. Nature Neuroscience. 2018; 21(12): 1742-52.

## P79 Frequency-dependent synaptic gain in a computational model of mouse thoracic sympathetic postganglionic neurons

### Astrid Prinz^1^, Michael McKinnon^1^, Kun Tian^1^, Shawn Hochman^2^

#### ^1^Emory University, Department of Biology, Atlanta, Georgia, United States of America; ^2^Emory University, Department of Physiology, Atlanta, Georgia, United States of America

##### **Correspondence:** Astrid Prinz (astrid.prinz@emory.edu)

*BMC Neuroscience* 2020, **21(Suppl 1)**:P79

Postganglionic neurons in the thoracic sympathetic chain represent the final common output of the sympathetic nervous system. These neurons receive synaptic inputs exclusively from preganglionic neurons located in the spinal cord. Synaptic inputs come in two varieties: primary inputs, which are invariably suprathreshold, and secondary inputs, which exhibit a range of typically subthreshold amplitudes. Postganglionic neurons typically receive a single primary input and a variable number of secondary inputs in what has been described as an “n+1” connectivity pattern. Secondary inputs have often been viewed as inconsequential to cell recruitment due to the short duration of measured synaptic inputs and the relatively low tonic firing rate of preganglionic neurons *in vivo*. However, recent whole-cell patch clamp recordings reveal that thoracic postganglionic neurons have a greater capacity for synaptic integration than previous microelectrode recordings would suggest. This supports a greater role for secondary synapses in cell recruitment.

We previously created a conductance-based computational model of mouse thoracic postganglionic neurons. In the present study, we have expanded the single-cell model into a network model with synaptic inputs based on whole-cell recordings. We systematically varied the average firing rate of a network of stochastically firing preganglionic neurons and measured the resultant firing rate in simulated postganglionic neurons. Synaptic gain was defined as the ratio of postganglionic to preganglionic firing rate.

We found that for a network configuration that mimics the typical arrangement in mouse, low presynaptic firing rates (<0.1Hz) resulted a synaptic gain close to 1, while firing rates closer to 1Hz resulted in a synaptic gain of 2.5.Synaptic gain diminished for firing rates higher than ~3Hz (Fig. 1). We also determined that synaptic gain linearly increases with the number of secondary synaptic inputs (n) within the range of physiologically realistic presynaptic firing rate. Amplitude of secondary inputs also determines frequency-dependent synaptic gain, with a bifurcation where secondary synaptic amplitude equals recruitment threshold. We further demonstrate that the synaptic gain phenomenon depends on the preservation of passive membrane properties as determined by whole-cell recordings.

**Fig. 1:**
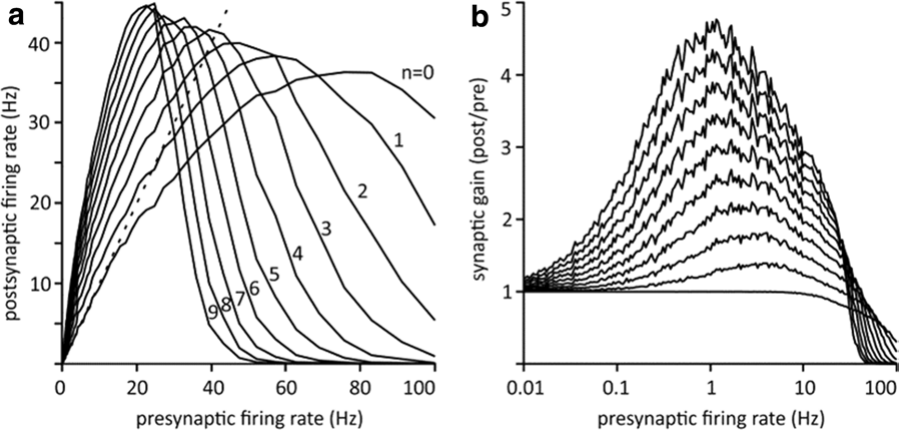
Effect of secondary inputs on synaptic gain. **A** Firing rate of postganglionic neurons as a function of presynaptic firing rate. Each simulation includes a single primary input, and n secondary synaptic inputs. Dashed line is the line of unity. **B** Synaptic gain as a function of presynaptic firing rate for postganglionic neurons with different numbers of secondary inputs

One major biological role of the sympathetic nervous system is the regulation of vascular tone in both skeletal muscle and cutaneous structures. The firing rate of muscle vasoconstrictor preganglionic neurons is modulated by the cardiac cycle, while cutaneous vasoconstrictor neurons fire independently of the cardiac cycle. We modulated preganglionic firing rate according to the typical mouse heart rate to determine if cardiac rhythmicity changes the overall firing rate of postganglionic neurons. Cardiac rhythmicity does not appear to have a significant impact on synaptic gain within the physiological range of preganglionic input.

Under normal physiological conditions, the unity gain of sympathetic neurons would lead to faithful transmission of central signals to peripheral targets. However, during episodes of high sympathetic activation, the postganglionic network can amplify central signals in a frequency-dependent manner. These results suggest that postganglionic neurons play a more active role in shaping sympathetic activity than previously thought.

## P80 Comp-NeuroFedora, a Free/Open Source operating system for computational neuroscience: download, install, research

### Ankur Sinha^1^, Aniket Pradhan^2^, Qianqian Fang^2^, Danny Lee^2^, Danishka Navin^2^, Alberto R Sanchez^2^, Luis Bazan^2^, Luis M Segundo^2^, Alessio Ciregia^2^, Zbigniew J˛edrzejewski-Szmek^2^, Sergio Pascual^2^, Antonio Trande^2^, Victor M T Yau^2^, Morgan Hough^2^

#### ^1^University of Hertfordshire, Biocomputation Research Group, Hatfield, United Kingdom; ^2^Fedora Project

##### **Correspondence:** Ankur Sinha (a.sinha2@herts.ac.uk)

*BMC Neuroscience* 2020, **21(Suppl 1)**:P80

The promotion and establishment of Open Neuroscience [1] is heavily dependent on the availability of Free/Open Source Software (FOSS) tools that support the modern scientific process. While more and more tools are now being developed using FOSS driven methods to ensure free (as in freedom, and thus also free of cost) access to all, the complexity of these domain specific tools tends to hamper their uptake by the target audience – scientists hailing from multiple, sometimes non-computing, disciplines. The NeuroFedora initiative aims to shrink the chasm between the development of neuroscience tools and their usage [2].

Using the resources of the FOSS Fedora community [3] to implement current best practices in software development, NeuroFedora volunteers identify, package, test, document, and disseminate neuroscience software for easy usage on the general purpose Fedora Linux Operating System (OS). The result is the reduction of the installation/deployment process for this software to a simple two-step process: install any flavour of the Fedora OS; install the required tools using the in-built package manager.

To make common computational neuroscience tools even more accessible, NeuroFedora now provides an OS image that is ready to download and use. In addition to a plethora of computational neuroscience software -Auryn [4], NEST [5], Brian [6], NEURON [7], GENESIS [8], Moose [9], Neurord [10], and others - the image also includes various utilities that are commonly used along with modelling tools, such as the complete Python science stack. Further, since this image is derived from the popular Fedora Workstation OS, it includes the modern GNOME integrated application suite and retains access to thousands of scientific, development, utility, and other daily use tools from the Fedora repositories.

A complete list of available software can be found at the NeuroFedora documentation at neuro.fedoraproject.org. We invite students, trainees, teachers, researchers, and hobbyists to use Comp-NeuroFedora in their work and provide feedback. As a purely volunteer driven initiative, in the spirit of the Open Science and FOSS, we welcome everyone to participate, engage, learn, and contribute in whatever capacity they wish.

**References**

1. Gleeson P, Davison AP, Silver RA, Ascoli GA. A commitment to open source in neuroscience. Neuron. 2017; 96(5): 964-5.
2. Sinha A, et al. NeuroFedora: a ready to use Free/Open Source platform for Neuroscientists. *BMC Neuroscience* 20; 1471-2202 at: https://neuro.fedoraproject.org.
3. Hat R. Fedora Project.
4. Zenke F, Gerstner W. Limits to high-speed simulations of spiking neural networks using general-purpose computers. Frontiers in Neuroinformatics. 2014; 8.
5. Linssen C, et al. NEST 2.16. 0. Jülich Supercomputing Center; 2018.
6. Goodman DF, Brette R. The brian simulator. Frontiers in Neuroscience. 2009; 3: 26.
7. Hines ML, Carnevale NT. The NEURON simulation environment. Neural computation. 1997; 9(6): 1179-209.
8. Bower JM, Beeman D, Hucka M. The GENESIS simulation system. 2003.
9. Dudani N, Ray S, George S, Bhalla US. Multiscale modeling and interoperability in MOOSE. BMC Neuroscience. 2009; 10(1): 1-2.
10. Jȩdrzejewski-Szmek Z, Blackwell KT. Asynchronous τ-leaping. The Journal of Chemical Physics. 2016; 144(12): 125104.

## P81 A computational neural model of pattern motion selectivity of MT neurons

### Parvin Zarei Eskikand^1^, David Grayden^2^, Tatiana Kameneva^3^, Anthony Burkitt^2^, Michael Ibbotson^1^

#### ^1^University of Melbourne, Melbourne, Australia; ^2^University of Melbourne, Department of Biomedical Engineering, Melbourne, Australia; ^3^Swinburne University of Technology, Telecommunication Electrical Robotics and Biomedical Engineering, Melbourne, Australia

##### **Correspondence:** Parvin Zarei Eskikand (pzarei@unimelb.edu.au)

*BMC Neuroscience* 2020, **21(Suppl 1)**:P81

The middle temporal area (MT) within the extrastriate primate visual cortex contains a high proportion of direction-selective neurons. When the visual system is stimulated with plaid patterns, a range of cell-specific MT responses are observed. MT neurons that are selective to the direction of the pattern motion are called “pattern cells”, while those that respond optimally to the motion of the individual component gratings of the plaid pattern are called “component cells”. The current theory on the generation of pattern selectivity of MT neurons is based on a hierarchical relationship between component and pattern MT neurons, where the responses of pattern MT neurons result from the summation of the responses of component MT neurons [1]. Where the gratings cross in plaids, the crossing junctions of the gratings move in the pattern direction. However, revealing the ends of the moving gratings (terminators) in human perceptual experiments breaks the illusion of the direction of pattern motion: the true directions of motion of the gratings are perceived.

Here, we propose a biologically plausible model of MT neurons that uses as inputs the known properties of three types of cells in the primary visual cortex (V1): complex V1 neurons, end-stopped V1 neurons (which only respond to the end-points of the stimulus), and V1 neurons with suppressive extra-classical receptive fields. The receptive fields of the neurons are modelled as spatiotemporal filters. There are two types of MT neurons: integration MT neurons with facilitatory surrounds and segmentation MT neurons with antagonistic surrounds [2]. A neuron’s pattern or component selectivity is controlled by the relative proportions of the inputs from the three types of V1 neurons. The model provides a simple mechanism by which component and pattern selective cells can be described; the model does not require a hierarchical relationship between component and pattern MT cells.

The results show that the responses of the model MT neurons are highly dependent on two parameters: the excitatory input that the model neurons receive from the complex V1 neurons with extra-classical RFs and the inhibitory effect of the end-stopped neurons. The results also show experimentally observed contrast dependency of the pattern motion preference of MT neurons: the level of the pattern selectivity of MT neurons drops significantly when the contrast of the bars is reduced.

The presented model solves several problems associated with MT motion detection, such as overcoming the aperture problem and extracting the correct motion directions from crossing bars. Apart from the mechanism of the computation of the pattern motion by MT neurons, the model inherently explains several important properties of pattern MT neurons, including their temporal dynamics, the contrast dependency of pattern selectivity, and the spatial and temporal limits of pattern motion detection.

**References**

1. Kumbhani RD, El-Shamayleh Y, Movshon JA. Temporal and spatial limits of pattern motion sensitivity in macaque MT neurons. Journal of Neurophysiology. 2015; 113(7): 1977-88.
2. Zarei Eskikand P, Kameneva T, Burkitt AN, Grayden DB, Ibbotson MR. Pattern motion processing by MT neurons. Frontiers in neural circuits. 2019; 13: 43.

## P82 Contrast invariant tuning in primary visual cortex

### Hamish Meffin^1^, Ali Almasi^1^, Michael R Ibbotson^2^

#### ^1^National Vision Research Institute, Carlton, Australia; ^2^Australian College of Optometry, The National Vision Research Institute, Carlton, Australia

##### **Correspondence:** Hamish Meffin (hmeffin@yahoo.com)

*BMC Neuroscience* 2020, **21(Suppl 1)**:P82

Previous studies show that neurons in primary visual cortex (V1) exhibit contrast invariant tuning to the orientation of spatial grating stimuli [1]. Mathematically this is equivalent to saying that their response is a multiplicatively separable function of contrast and orientation.

Here we investigated the contrast dependence of V1 tuning to visual features in a more general framework. We used a data-driven modelling approach [2] to identify the spectrum of spatial features to which individual V1 neurons were sensitive, from our recordings of single unit responses in V1 to white (Gaussian) noise and natural scenes. For each cell we identified between 1 and 5 spatial feature dimensions to which the cell was sensitive (e.g. Fig. 1A, with 2 feature dimensions; feature 1 & 2 as labelled, with red showing bright and blue showing dark regions of the feature). The response of a neuron to its set of features was estimated from the data as the spike rate equal to a function of the individual feature-contrasts: *r = F(c*_1_,…,c_K_) (Eq. 1) where *c*_1_,…,c_K_ are the contrast levels of a cell’s spatial features, *1,..K,* embedded in any stimulus (e.g. Fig. 1B).These features spanned a subspace, giving a spectrum of interpolated features to which the cell was sensitive (Fig.1A, examples labelled). The identity of these features varied along the angular polar coordinate in this subspace, which we term the feature-phase,*φ* (Fig. 1A, labelled). In this angular dimension, characteristics of the features, such as their spatial phase, orientation or spatial frequency, were found to vary continuously. In the radial coordinate, the contrast of these features varied, c = (*c*_1_,…,c_K_) (Fig. 1A, labelled).

We found that the neural response above the spontaneous rate, *r*_0_, was well approximated by a multiplicatively separable function of the feature-contrast and feature-phase (Fig. 1C): *r = f*_*c*_(*c*) f_φ_(*φ*) + r_0_ (Eq.2).

To quantify the accuracy of this approximation, we calculated a relative error between the original and separable forms of the feature-contrast response function (i.e. Eq. (1) & (2)). This relative error varied between 2% and 18% across the cell population, with a mean of 6%. This indicates that for most cells, the separable form of the feature-contrast response function was a good approximation.

This result may be interpreted as demonstrating a form of contrast invariant tuning to feature-phase in V1. This tuning to feature-phase is given by the function *f*_*φ*_(*φ*) (Fig. 1E), and the contrast response function is given by *f*_*c*_(*c*) (Fig. 1D). As several feature characteristics such as spatial phase, orientation or spatial frequency covary with feature-phase, this also leads to contrast invariant tuning under covariation in these characteristics as feature-phase varies.

**Acknowledgements**: The authors acknowledge the support the Australian Research Council Centre of Excellence for Integrative Brain function (CE140100007), the National Health and Medical Research Council (GNT1106390), and Lions Club of Victoria.

**Fig. 1:**
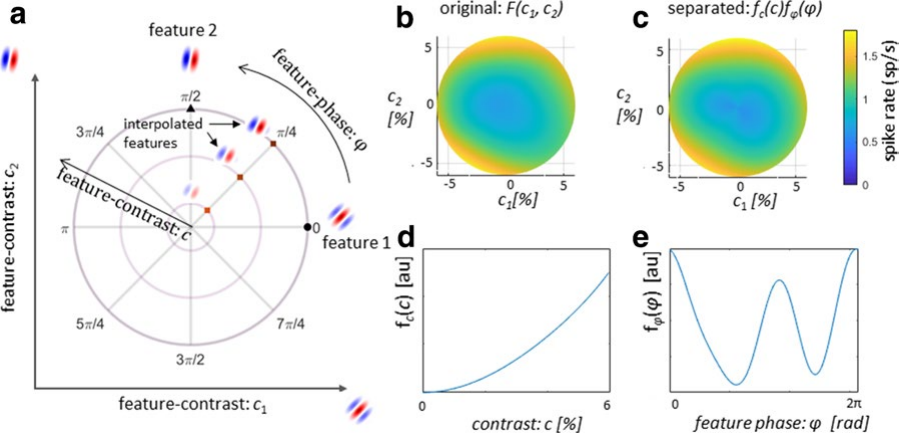
**A** Feature subspace of a cell. **B** Original feature-contrast response function. **C** Separable approximation of the feature-contrast response function. **D** Separable contrast response function. **E** Separable feature-phase tuning function

**References**

1. Alitto HJ, Usrey WM. Influence of contrast on orientation and temporal frequency tuning in ferret primary visual cortex. Journal of neurophysiology. 2004; 91(6): 2797-808.
2. Almasi A, et al. Mechanisms of feature selectivity and invariance in primary visual cortex. bioRxiv 2020.

## P83 One-shot model to learn 7 quantal parameters simultaneously, including desensitization

### Emina Ibrahimovic

#### University of Zurich and Elvesys, Zurich, Switzerland

##### **Correspondence:** Emina Ibrahimovic (emina.ibrahimovic@imls.uzh.ch)

*BMC Neuroscience* 2020, **21(Suppl 1)**:P83

Synaptic transmission is the hallmark behind informative and adaptive cognitive processes. Neurons store and enable retrieval of information by connecting via synapses. They can modulate the signal either to maintain stability or decrease or increase their strength. To improve quantification of synaptic dynamics with focus on computational efficiency and number of parameters, a fast variant of the binomial model is applied to estimate θ={N,p,q,σ,τ_d_,τ_f_,τ_des_}.

There is up to date no efficient method to simultaneously quantify many synaptic parameters from physiological recordings. Generative models have heavy computational loads as they depend on the number of release sites N [1], and need to compute multiple iterations. Empirical methods usually require specific conditions, such as changing the release probability p or requiring regular inter spike intervals, which makes them unsuitable for fitting physiological data. The fast algorithm proposed here is based on non-linear least squares to retrieve quantal parameters. We apply this model to spontaneous excitatory postsynaptic potentials (EPPs) patch-clamped at an in vitro mouse neuromuscular junction (NMJ) taken in [2] (Fig. 1A). Results in control are relatively in line with estimations found in vivo [3] (Fig. 1C). Estimated quantal parameters at control are subsequently compared with data undergoing curare poison (Fig. 1B). The addition of curare shows a drop in quantal size q as expected. Other parameters remain stable suggesting no homeostatic compensatory mechanisms in this case. The model is validated on synthetic data and compared with an iterative expectation-maximization technique. The supremacy of this one-shot method allows to retrieve many parameters without multiple batches. In contrast to [1], its temporal complexity is independent of the number of release sites. This method is adaptable to general synaptic recording, which makes it less constrained. It is also the first phenomenological model that incorporates desensitization τ_des_. The framework gives insights on synaptic weights and their long-short dynamics. It permits to correlate between subjective and objective variabilities in healthy and pathological cases to molecular functions.

**Fig. 1:**
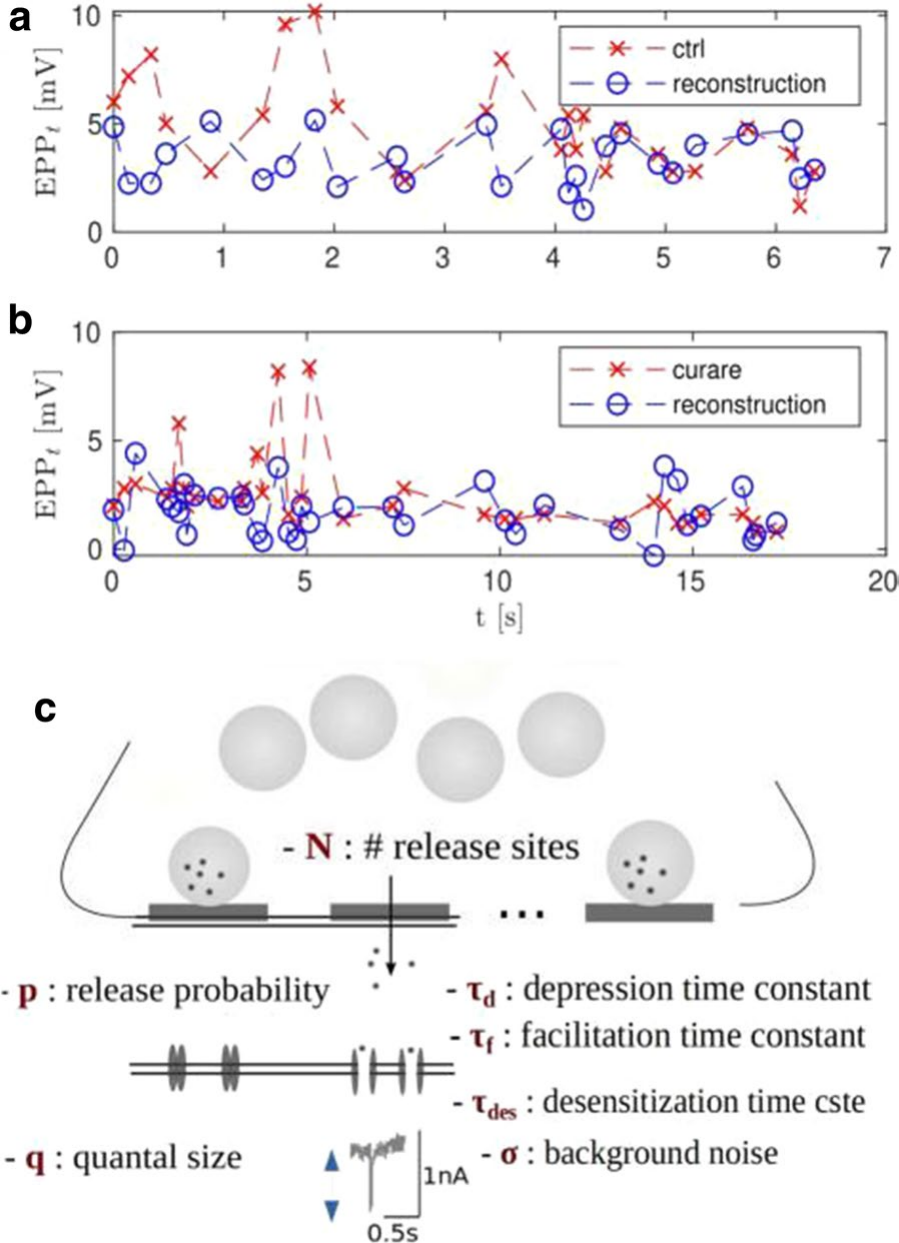
In vitro mouse NMJ EPPs with model reconstruction (blue). **A** Control: number of release sites N=100, release probability p=0.4, quantal size q=0.13mV, background noise σ=0.2mV, depression time constant τ_d_=95ms, refilling time constant τ_f_=50ms, desensitization τ_des_= 30ms. **B** Curare administration: N=100, p=0.5, q=0.05mV, σ=0.1mV, τ_d_=45ms, τ_f_=50ms, τ_des_=30ms. **C** Cartoon of a synaptic junction. Comparison of estimation of parameters with rat NMJ can be found in [3]

**References**

1. Barri A, Wang Y, Hansel D, Mongillo G. Quantifying repetitive transmission at chemical synapses: a generative-model approach. eNeuro. 2016; 3(2).
2. Vilmont V, Cadot B, Ouanounou G, Gomes ER. A system for studying mechanisms of neuromuscular junction development and maintenance. Development. 2016; 143(13): 2464-77.
3. Wilson DF. Estimates of quantal-release and binomial statistical-release parameters at rat neuromuscular junction. American Journal of Physiology-Cell Physiology. 1977; 233(5): C157-63.

## P84 Brainpower – investigating the role of power constraints in neural network plasticity

### Silviu Ungureanu^1^, Mark van Rossum^2^

#### ^1^University of Nottingham, School of Psychology, Nottingham, United Kingdom; ^2^University of Nottingham, School of Psychology and School of Mathematical Sciences, Nottingham, United Kingdom

##### **Correspondence:** Silviu Ungureanu (silviu.ungureanu@nottingham.ac.uk)

*BMC Neuroscience* 2020, **21(Suppl 1)**:P84

The central nervous system consumes approximately 20W of metabolic power in humans [1]. This is used for neural communication, and also for neural plasticity and the formation of new memories. Persistent forms of plasticity in particular consume so much energy that under sudden food scarcity, associative learning significantly reduces lifespan of fruit flies [2].

It is reasonable therefore that neural plasticity has evolved to learn at minimal power. However, how this changes plasticity and learning is not known. While previous work has considered an energy constraint [3], a power constraint might be more biological as, unlike many other tissues, the brain cannot store energy. A power constraint might be able to explain why plasticity induction requires a refractory time before it can be induced again [4], as well as spatial competition in plasticity between synapses and neurons [5,6].

Here, we developed a computational model of plasticity to examine the effect of a power constraint on plasticity dynamics. We first use a standard perceptron augmented with two types of synaptic weights: an inexpensive transient, decaying component and a costly long-term component, formed by the simultaneous consolidation of all the transient weights. We further assume that the brain attempts to consolidate new memories as soon as it is able to. Hence, the interval between consolidation events is limited and synaptic consolidation events occur at a fixed frequency, representing the refractory period caused by a dearth of energy. Higher consolidation frequencies correspond to more available power, and vice-versa. The perceptron is trained on a random-generated set of binary patterns until it correctly learns the output value for each pattern.

Results show that the power in the system has a significant impact on the training time. Unexpectedly, increasing the period between consolidations - thus reducing power - can reduce the required number of epochs by as much as 30%, depending on the strength of the weight decay, the number of patterns P in the training set, and the number of synapses N. Further increasing the period between consolidations increases the training time. This increase occurs not gradually, but in a staircase pattern, peaking whenever the period is 0 modulo P (Fig. 1).

**Fig. 1:**
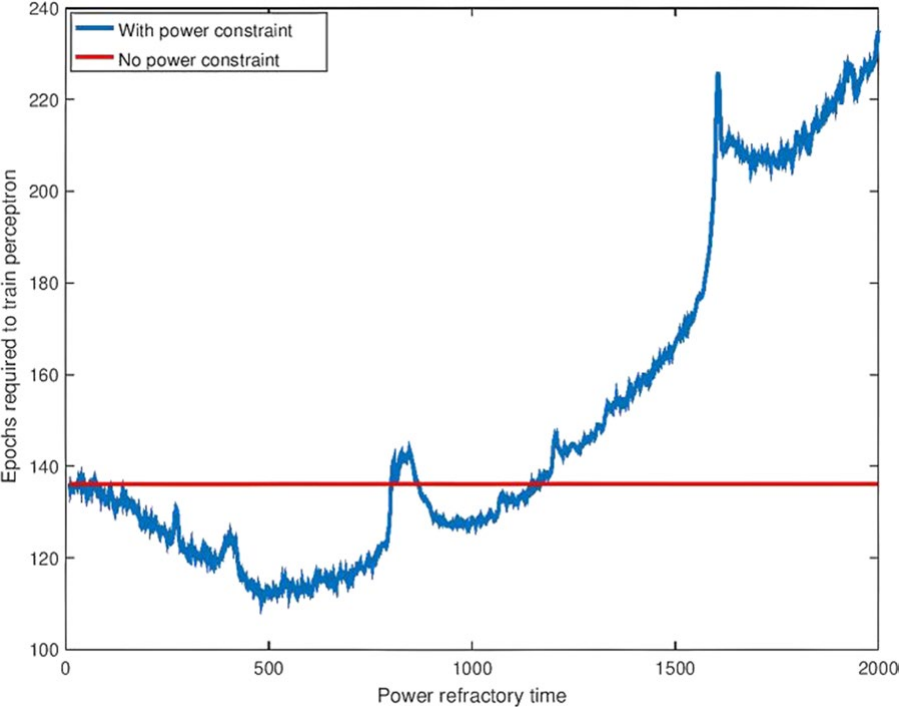
A perceptron with 500 synapses trained on 800 patterns. In blue, number of epochs necessary for the perceptron to learn the entire training set, as a function of the interval between consolidation events. In red, the number of epochs needed by a standard perceptron

The consequences of a power constraint are further explored in a multi-layer neural network and extended to a probabilistic model where the probability of consolidation increases proportional to the time since the previous consolidation event.

In summary, our results show that incorporation of a metabolic power constraint in synaptic plasticity can lead to important changes in the learning dynamics.

**References**

1. Attwell D, Laughlin SB. An energy budget for signaling in the grey matter of the brain. Journal of Cerebral Blood Flow & Metabolism. 2001; 21(10): 1133-45.
2. Mery F, Kawecki TJ. A cost of long-term memory in Drosophila. Science. 2005; 308(5725): 1148-.
3. Li HL, Van Rossum MC. Energy efficient synaptic plasticity. Elife. 2020; e50804.
4. Kramár EA, et al. Synaptic evidence for the efficacy of spaced learning. Proceedings of the National Academy of Sciences. 2012; 109(13): 5121-6.
5. Sajikumar S, Morris RG, Korte M. Competition between recently potentiated synaptic inputs reveals a winner-take-all phase of synaptic tagging and capture. PNAS 2014; 111: 12217.
6. Josselyn SA, Tonegawa S. Memory engrams: Recalling the past and imagining the future. Science. 2020; 367: 6473.

## P85 The Neuron growth and death model and simulator

### Yuko Ishiwaka, Tomohiro Yoshida, Tadateru Itoh

#### SoftBank Corp., Technology Unit, Hakodate, Japan

##### **Correspondence:** Yuko Ishiwaka (yuko.ishiwaka@g.softbank.co.jp)

*BMC Neuroscience* 2020, **21(Suppl 1)**:P85

Some kinds of characteristics of neurons depend on morphology. There are many neuron types in a brain and the functions of each cell type are varied. For example, auditory cells that receive sound stimulus from the external world and pyramidal cells that relate to thinking and memory have different functions. A bushy cell which is one of the sensory neurons of auditory treats tempo of sounds, therefore the immediate responses are required for producing action potential and short time refractory period. On the other hand, pyramidal cells that mainly exist in the hippocampus and amygdala treat memory and emotion, therefore the producing action potential is slower than sensor neurons and the refractory period is longer. Hodgkin and Huxley (H-H) equations can calculate action potentials based on ion channels. H-H does not consider morphology, however, on actual cell membranes, the number of exiting ion channels and locations are based on shapes of neurons. How quick or slow soma can produce action potentials are depend on how narrow and how many ion channels on the producing area. Therefore, we assume that there are strong relationships between cell shapes and characteristics of action potentials. Expanded H-H equations can adapt to the quickness of producing action potential by adding axon hillock parameters.

Connectivity between neurons is also important. Geometry varies according to cell types. Purkinje cells which are one of the inhibitory neurons have complex branches of the dendritic arbor. On the other hand, Pyramidal cells which are one of the excitatory neurons and multipolar type neurons have one axon and many dendrites, but the complexity of geometry is simpler than Purkinje cells. These differentials of geometry cause differences in connectivity.

In this paper, we propose a new neuron growth and deal model and simulator considered neuron morphology and connectivity between multi cell types. In our model, a characteristic of a growth cone is applied to neuron growth and treated as a navigation system, an L-system is adapted for creating the geometry of each neuron, and Life game is embedded for a cell division rule.

We also adopt glial cells for neuron growth, not only stimulus from other neurons. In our model, each neuron receives the energy for growing from contacted astrocytes which are one of the glial cells. The direction of growth of the growth cones has determined by set goal areas for far, and during growing, growth cones try to contract near oligodendrocytes to obtain myelin around their axons. A cell division rule for Oligodendrocytes follows life game rules. The glial cells are treated as obstacles.

In our simulation system, a user can create various types of neurons, set the goals for both dendrites and axons, create connections between various functions and geometries of neurons with growth rules and add injections such as inhibitory postsynaptic potential (IPSP) or excitatory postsynaptic potential (EPSP) on purpose to calculate action potentials.

In conclusion, Fig. 1 shows simulation results of our proposed model. In our simulator, variety of geometry can be produced automatically based on expanded L-system, variety and flexible connectivity can be also produced based on our proposed new neuron growth and death model. Furthermore, we added two types of glial cells for growth and goal rules and also treat as obstacles. Our proposed model and simulator is quite flexible to simulate cell geometry, action potentials, cell connections in each brain region.

**Fig. 1:**
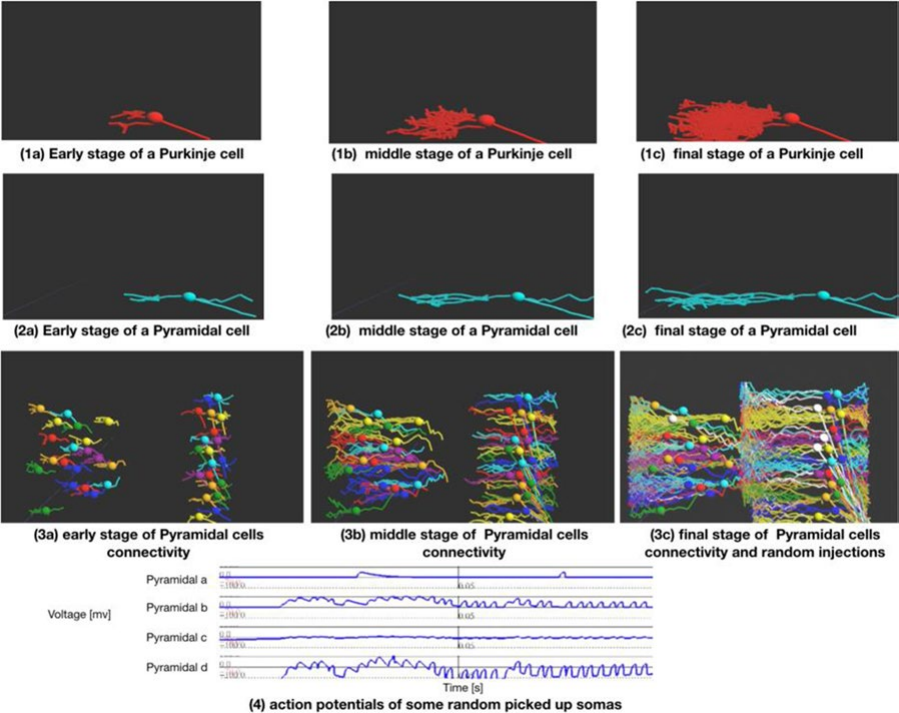
Simulation results of different geometries and neuron connections. The upper red neurons are simulated as a Purkinje, middle blue neurons are simulated as a Pyramidal cell, and the bottom neurons show connections and created action potentials by expanded H-H model

## P86 Studying neural mechanisms in recurrent neural network trained for multitasking depending on a context signal.

### Cecilia Jarne

#### National University of Quilmes and CONICET, Departement of Science and Technology, Quilmes, Argentina

##### **Correspondence:** Cecilia Jarne (katejarne@gmail.com)

*BMC Neuroscience* 2020, **21(Suppl 1)**:P86

Most biological brains, as well as artificial neural networks, are capable of performing multiple tasks [1]. The mechanisms through which simultaneous tasks are performed by the same set of units are not yet entirely clear. Such systems can be modular or mixed selective through some variable such as sensory stimulus [2,3]. Based on simple tasks studied in our previous work [4], where tasks consist of the processing of temporal stimuli, we build and analyze a simple model that can perform multiple tasks using a contextual signal. We study various properties of our trained recurrent networks, as well as the response of the network to the damage done in connectivity. In this way we are trying to illuminate those mechanisms similar to those that could occur in biological brains associated with multiple tasks.

**References**

1. Yang GR, Joglekar MR, Song HF, Newsome WT, Wang XJ. Task representations in neural networks trained to perform many cognitive tasks. Nature Neuroscience. 2019; 22(2): 297-306.
2. Yang GR, Cole MW, Rajan K. How to study the neural mechanisms of multiple tasks. Current Opinion in Behavioral Sciences. 2019; 29: 134-43.
3. Rigotti M, et al. The importance of mixed selectivity in complex cognitive tasks. Nature. 2013; 497(7451): 585-90.
4. Jarne C, Laje R. A detailed study of recurrent neural networks used to model tasks in the cerebral cortex. arXiv preprint arXiv:1906.01094. 2019.

## P87 Centrality of left inferior frontal gyrus reveals important aspect of motor learning generalization

### Youngjo Song, Sang Jin Jang, Pyeong-soo Kim, Byung Hyug Choi, Jaeseung Jeong

#### Korea Advanced Institute of Science and Technology, Bio and Brain Engineering, Daejeon, South Korea

##### **Correspondence:** Youngjo Song (syj1455@kaist.ac.kr)

*BMC Neuroscience* 2020, **21(Suppl 1)**:P87

Generalization of learning refers to the phenomenon in which knowledge learned in one context enhances performance in another context. Although the learning environment can never be precisely the same in the real world, animals including humans demonstrate excellent flexibility to adapt their learned skills in a new environment. Despite the universal occurrence of generalization phenomena in daily life, there is much lack of understanding about how the brain generalizes the skills and knowledge into different environments. In particular, most of the previous studies have only focused on identifying the cerebral networks used during the generalization stage, but failed to determine the elements during the preceding learning stage that could have enabled generalization. Thus, the aim of this study was to enhance understanding of the neural mechanisms that enable generalization, particularly the generalization of motor learning. In this study, we designed a new experimental paradigm called ‘mirror-erasing generalization task.’ The subjects erased (1) a simple shape (square) for the training session, and (2) a complex shape (cursive alphabet letter y) for the generalization session, which took place both before and after the training session, in an MRI scanner. We found that the subjects successfully generalized their motor skills (p < 0.0001) acquired during square-erasing to the letter-erasing context. However, counterintuitively, skill improvement during training did not correlate with the generalization of motor skills (p ~ 0.1). This result implicates that the dynamics underlying generalization is possibly nonlinear, and that performance enhancement in one specific context is not a reliable measure to estimate the generalization performance in another. Then, we computationally modeled the neuronal circuitry responsible for motor learning generalization and used the fMRI machine to construct a functional network model (using frequency between 0.049Hz and 0.09Hz). More interestingly, we found that the betweenness centrality of pars opercularis of left inferior frontal gyrus (IFG) had a significant correlation with the generalization performance (R ~ 0.8, p < 0.05, FWE corrected), which was the only measure before generalization session that correlated with generalization performance. We should note that the human pars opercularis of the left IFG has been considered as part of a mirror neuron system, which is currently hypothesized to be facilitating motor abstraction. This finding suggests that the IFG-mediated abstraction of new motor skill acquired during training may be the key to generalization of the learned skills in a different context. This study potentially provides evidence for the contemporary view that abstraction plays an essential role in generalization. Furthermore, we suggest that the centrality of par opercularis of the left IFG is possibly used to make predictions about future generalization performance, which opens new possibilities in motor rehabilitation. This study suggests that measuring brain functional networks of the patients undergoing rehabilitation programs potentially predict how much their motor function would be improved in real life.

**Acknowledgements:** This study was conducted as part of Global Singularity Research Program for 2020 financially supported by KAIST.

## P88 Phosphorylation-induced variation of NF kinetics and morphology of axon

### Zelin Jia, Yinyun Li

#### Beijing Normal University, School of System Science, Beijing, China

##### **Correspondence:** Zelin Jia (zljia@mail.bnu.edu.cn)

*BMC Neuroscience* 2020, **21(Suppl 1)**:P88

Neurofilaments (NFs) are transported along microtubule tracks in the axons and are gradually phosphorylated in this process [1]. At the node of Ranvier, the axon is not encased by myelin sheath. It is indicated that phosphorylation of NFs can reduce the transport rate of NFs, and therefore influence the formation of axon morphology [2]. In the theory of slow axonal transport, the “stop-and-go” model [3] can well describe the random kinetic behavior of NFs in axons. On the basis of “stop-and-go” model, we introduce the conversion between phosphorylation and dephosphorylation of neurofilaments and build the “eight-state” model. We assume that the phosphorylation and dephosphorylation of NFs has different “on-track” rate (γon), so as to achieve the effect of phosphorylation on the transport [4,5]. Through our theoretical derivation and simulation, we draw the conclusion that the modification on the “on-track” rate and the conversion between phosphorylation and dephosphorylation can both slow down the NFs transport along the axon. Our conclusion is also consistent with the Continuity equation that flux by the multiplication of the number of NFs and average velocity is constant at equilibrium state.

**References**

1. Nixon RA, Paskevich PA, Sihag RK, Thayer CY. Phosphorylation on carboxyl terminus domains of neurofilament proteins in retinal ganglion cell neurons in vivo: influences on regional neurofilament accumulation, interneurofilament spacing, and axon caliber. The Journal of cell biology. 1994; 126(4): 1031-46.
2. Jung C, Yabe JT, Shea TB. C-terminal phosphorylation of the high molecular weight neurofilament subunit correlates with decreased neurofilament axonal transport velocity. Brain research. 2000; 856(1-2): 12-9.
3. Wang L, Brown A. Rapid intermittent movement of axonal neurofilaments observed by fluorescence photobleaching. Molecular biology of the cell. 2001; 12(10): 3257-67.
4. Yabe JT, Pimenta A, Shea TB. Kinesin-mediated transport of neurofilament protein oligomers in growing axons. Journal of cell science. 1999; 112(21): 3799-814.
5. Yabe JT, Jung C, Chan WK, Shea TB. Phospho‐dependent association of neurofilament proteins with kinesin in situ. Cell motility and the cytoskeleton. 2000; 45(4): 249-62.

## P89 Quantification of changes in motor imagery skill during brain-computer interface use

### James Bennett, David Grayden, Anthony Burkitt, Sam John

#### University of Melbourne, Department of Biomedical Engineering, Melbourne, Australia

##### **Correspondence:** James Bennett (bennettj1@student.unimelb.edu.au)

*BMC Neuroscience* 2020, **21(Suppl 1)**:P89

Oscillatory activity over the sensorimotor cortex, known as sensorimotor rhythms, can be modulated by the kinaesthetic imagination of limb movement [1]. These event-related spectral perturbations can be observed in electroencephalography (EEG), offering a potential way to restore communication and control to people with severe neuromuscular conditions via a brain-computer interface (BCI). However, the ability of individuals to produce these modulations varies greatly across the population. Between 10-30% of people are unable to influence their SMRs sufficiently to be distinguishable by a BCI decoder [2]. Despite this, it has been shown that users can be trained to improve the extent of their SMR modulations. This research utilised a data-driven approach to characterise the skill development of participants undertaking a left- and right-hand motor imagery experiment.

Two publicly available motor imagery EEG datasets were analysed. Dataset 1 consisted of EEG data from 47 participants performing 200 trials of left- and right-hand motor imagery within a single session [3]. No real-time visual feedback was provided to the participants. Dataset 2 contained EEG from two sessions of 200 trials each from 54 participants [4]. Visual feedback was provided to users in the second session but not in the first. Various metrics characterising mental imagery skill were calculated across time for each participant.

The discriminability of EEG in the 8-30Hz range from left- and right-hand trials was found to increase across time for both datasets. Despite the overall improvement, there was great variability in the change of motor imagery skill across participants. For Dataset 1, the average change across time of the metric representing the discriminability of classes was 6.0±21.9%. For Sessions 1 and 2 of Dataset 2, the discriminability increased by 11.8±44.0% and 17.4±30.7%, respectively. Session 2 of Dataset 2 contained visual feedback and produced a larger overall improvement in motor imagery skill with a lower variability compared with Session 1.

In this work, we investigated the level of motor imagery skill acquisition during BCI use. The results indicate a baseline level of skill improvement that can be expected, and also emphasise the large variability across participants commonly seen in BCI studies. Overall, we provide a useful reference of BCI skill acquisition for future research that seeks to increase the rate of skill improvement and decrease the amount of variability.

**References**

1. Pfurtscheller G, Da Silva FL. Event-related EEG/MEG synchronization and desynchronization: basic principles. Clinical Neurophysiology. 1999; 110(11): 1842-57.
2. Allison BZ, Neuper C. Could anyone use a BCI? In Brain-computer interfaces 2010 (pp. 35-54). Springer, London.
3. Cho H, Ahn M, Ahn S, Kwon M, Jun SC. EEG datasets for motor imagery brain–computer interface. GigaScience. 2017; 6(7): gix034.
4. Lee MH, et al. EEG dataset and OpenBMI toolbox for three BCI paradigms: an investigation into BCI illiteracy. GigaScience. 2019; 8(5): giz002.

## P90 Texture-like neural representations

### Emil Dmitruk^1^, Ritesh Kumar^1^, Volker Steuber^2^, Michael Schmuker^2^, Carina Curto^3^, Vladimir Itskov^3^, Shabnam Kadir^4^

#### ^1^University of Hertfordshire, School of Engineering and Computer Science, Hatfield, United Kingdom; ^2^University of Hertfordshire, Biocomputation Research Group, Hatfield, United Kingdom; ^3^Pennsylvania State University, Department of Mathematics, State College, Pennsylvania, United States of America; ^4^University of Hertfordshire, Centre for Computer Science and Informatics Research, Hatfield, United Kingdom

##### **Correspondence:** Emil Dmitruk (e.dmitruk@herts.ac.uk)

*BMC Neuroscience* 2020, **21(Suppl 1)**:P90

An almost ubiquitous approach taken in systems neuroscience is that of devising stimulus response functions (SRFs), which specify how a stimulus is encoded into a neural response, e.g. place fields, tuning curves [1]. However, it is clear that the brain itself is able to infer properties of the environment in real time via neural activity alone, without having to resort to performing experiments on its own response to stimuli. A central aim in this work is to explore the relationship between the geometric/topological structure of the stimulus space (also animal behaviour) and neural activity and to investigate to what extent the former can be derived from the latter without having to perform the standard operation of constructing dictionaries between the two as provided by an SRF. In the last decade, successful attempts have been made to eliminate this albeit very useful middleman using topological data analysis (TDA) [2,3].

We build on an approach initiated in [3], which uses clique topology, a form of TDA. Common statistics related to neural activity and connectivity derived from experimental data are often presented in the form of a matrix of correlations or connectivity strengths between pairs of neurons, voxels, etc. Analogous statistics can be obtained from stimuli presented to the animal, e.g. textures both visual, auditory, images of natural scenes, olfaction. Clique topology enables us to test whether signatures of structures of stimulus spaces and environments are detectable in the correlation structures of the raw data obtained from neuronal recordings. The advantage of using clique topology over traditional eigenvalue-based methods is that the latter is badly distorted by monotone nonlinearities, whereas the information encoded in the `order complex’ is invariant under such transformations.

The statistical topological approach in [3] could determine whether correlations resulting from both the stimulus and response side were random or induced by a geometric process (e.g. pairwise distances obtained via sampling points from a unit cube in Rd). Here we introduce a new regime of complexes that are derived instead from textures. In [3] experimental scenarios were considered where neurons were tuned to features lying in a continuous coding space where correlations decrease with distance, e.g. hippocampal place cells. Textures are in many ways the antithesis of this and also exhibit both repetitive and random features. Clique topology techniques on textures have led us to a menagerie of order complexes which have very small values for Betti numbers throughout the filtration as compared to same-sized ‘random’ and ‘geometric’ order complexes (Fig. 1). Analogous Betti curves have been shown to have been induced by order complexes derived from low-rank matrices by Curto (unpublished). The matrices that emerge from textures, however, do not generally have low-rank and it is an open question as to whether the two can be related.

**Fig. 1:**
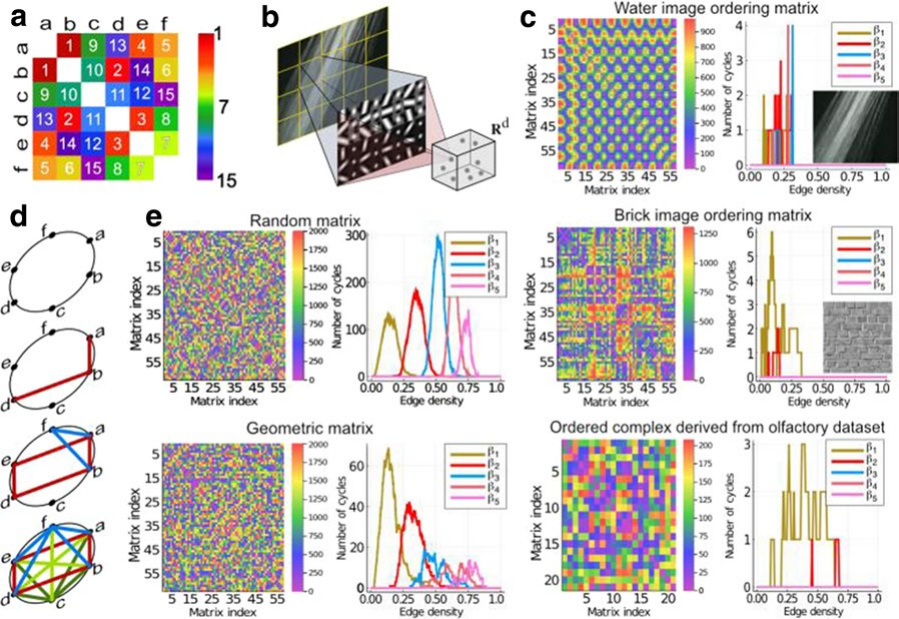
**A** An example of matrix ordering (values ordered according to the scale). **B** Derivation of order complex from a visual texture using Gabor filters. **C** Order matrices and Betti curves for textures (bricks, water) and olfactory dataset; images of textures are shown as insets on plots. **D** Order complexes (from A) as a simplicial complexes. **E** Betti curves for random and geometry matrix

We were surprised to find that datasets from a wider range of modalities appear to exhibit texture-like rather than ‘geometric’ structure. We have extracted texture-like order complexes from olfactory datasets [4] as well as a simulated dataset from a spiking neural network modelling speech recognition.

**References**

1. Meyer AF, Williamson RS, Linden JF, Sahani M. Models of neuronal stimulus-response functions: elaboration, estimation, and evaluation. Frontiers in Systems Neuroscience. 2017; 10: 109.
2. Curto C, Itskov V. Cell groups reveal structure of stimulus space. PLoS Computational Biology. 2008; 4(10): e1000205.
3. Giusti C, Pastalkova E, Curto C, Itskov V. Clique topology reveals intrinsic geometric structure in neural correlations. Proceedings of the National Academy of Sciences. 2015; 112(44): 13455-60.
4. Si G, et al. Structured odorant response patterns across a complete olfactory receptor neuron population. Neuron. 2019; 101(5): 950-62.

## P91 A unified framework for the application and evaluation of different methods for neural parameter optimization

### Máté Mohácsi^1^, Sára Sáray^1^, Márk P Török^2^, Szabolcs Kali^2^

#### ^1^Pázmány Péter Catholic University, Faculty of Information Technology and Bionics, Budapest, Hungary; ^2^Institute of Experimental Medicine, Hungarian Academy of Sciences, Budapest, Hungary

##### **Correspondence:** Szabolcs Kali (kali@koki.hu)

*BMC Neuroscience* 2020, **21(Suppl 1)**:P91

Currently available experimental data make it possible to create complex multicompartmental conductance-based models of neurons. In principle, such models can approximate the behavior of real neurons very well. However, these models have many parameters and some of these parameters often cannot be directly determined in experiments. Therefore, a common approach is to tune parameter values to bring the physiological behavior of the model as close as possible to the experimental data. Rather than tuning the parameters by hand, a more principled way of determining good model parameters is to carry out a systematic parameter search using an appropriate global optimization algorithm. Although many such algorithms have been developed and applied successfully in various domains, and high-quality general implementations of many popular algorithms are available, the majority of these solutions have not been tested in a neural context. Our goal in this study was to create a software tool that provides uniform access to a large variety of different optimization algorithms; to develop a set of benchmark problems for neural parameter tuning; and to systematically evaluate and compare the various algorithms and implementations using our software and benchmarking suite.

We have created an updated and enhanced version of our previously developed software tool. In Optimizer, model evaluations can be performed either by the NEURON simulator (handled internally) or any external (black-box) simulator. All functionalities can be accessed from the graphical user interface; there is also a command line interface for batch processing. The new version was developed in Python 3 to support recent open-source Python modules. The repertoire of algorithms was extended by several new methods that proved effective in other studies. For many of these search algorithms, parallel optimization is also supported. A wide variety of features (including those in the eFEL package) can be used to evaluate the error of the optimization; multiple, weighted features are also supported. Our optimization tool currently supports about fifteen different optimization algorithms implemented by four separate Python packages: Inspyred, Pygmo, BluePyOpt, and Scipy.

Our neural optimization benchmark suite includes six separate problems that differ in complexity, model type, simulation protocol, fitness functions, and the number of unknown parameters. Our examples range from the classical Hodgkin-Huxley model (3 conductance parameters) to an extended integrate-and-fire model (10 parameters) and a morphologically and biophysically detailed hippocampal pyramidal cell (16 parameters). Some of our benchmarks use target data generated by a neuronal model with known parameters. However, in most of our benchmarks, the target data were recorded in physiological experiments, or were generated by more complex models than the one we were fitting.

We then tested the various algorithms on the different model optimization tasks, and compared the final error (after 10,000 model evaluations) and also the convergence speed (Fig. 1). We found that several evolutionary and related search algorithms delivered consistently good results across our entire test suite, even for higher-dimensional, multi-objective problems. Therefore, we would recommend trying these algorithms first for novel optimization problems. We also hope to extend our test suite with new problems and algorithms.

**Fig. 1:**
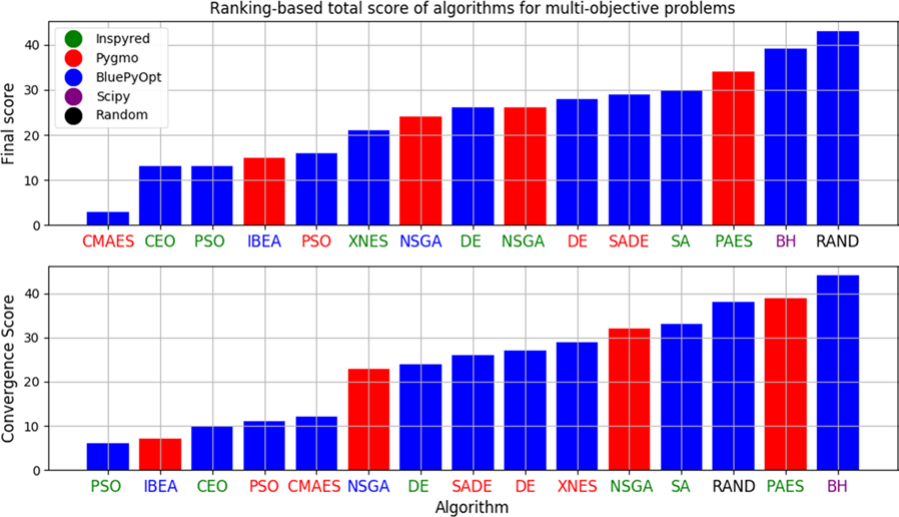
Rankings of the algorithms based on their combined performance on our multi-objective test case. Top: Ranking based on the final error after 10,000 model evaluations. Bottom: Ranking based on the area under the error curve (convergence speed). Lower scores indicate better performance. Red bars are for multi-objective algorithms, blue bars for single-objective algorithms

## P92 Modelling the responses of ON and OFF retinal ganglion cells to infrared neural stimulation

### James Begeng^1^, Wei Tong^2^, Michael R Ibbotson^2^, Paul Stoddart^3^, Tatiana Kameneva^4^

#### ^1^Swinburne University of Technology, Faculty of Science, Engineering and Technology, Melbourne, Australia; ^2^Australian College of Optometry, The National Vision Research Institute, Melbourne, Australia; ^3^Swinburne University of Technology, ARC Training Centre in Biodevices, Melbourne, Australia; ^4^Swinburne University of Technology, Telecommunication Electrical Robotics and Biomedical Engineering, Melbourne, Australia

##### **Correspondence:** James Begeng (jbegeng@swin.edu.au)

*BMC Neuroscience* 2020, **21(Suppl 1)**:P92

Retinal degenerative diseases such as retinitis pigmentosa and age-related macular degeneration cause progressive photoreceptor loss leading to partial or total patient blindness. Retinal prostheses attempt to obviate this loss of photoreceptors by direct stimulation of the underlying retinal ganglion cell (RGC) circuitry, and are capable of restoring limited visual sensation to blind patients. Because these devices typically inject current through implanted electrode arrays, their spatial resolution is significantly limited, and their capacity for selective stimulation of distinct RGC types has not yet been established. In particular, selective stimulation of ON and OFF RGCs (which exhibit opposite light responses *in vivo*) constitutes a long-standing open problem in retinal prosthesis design.

Infrared neural modulation (INM) uses pulsed infrared light to deliver sharp thermal transients to neural tissue, and is capable of both neural stimulation and inhibition with a high spatial precision. This technique relies on at least two distinct mechanisms: a temperature gradient dependent capacitive current, and thermosensitive activation of the TRPV ion channels. For retinal prostheses, this high stimulus resolution offers an attractive alternative to the low resolution of current electrical prostheses; however, it is unclear how infrared-evoked currents may vary between the wide variety of RGC types in mammalian retina, or whether these differences may be harnessed for selective stimulation.

In this study, a single-compartment Hodgkin-Huxley-type model was simulated in a NEURON environment. The model included leak, sodium, potassium, calcium and low voltage activated calcium currents based on published data [1,2]. Thermally-evoked currents were simulated by a dT/dt dependent capacitive current based on GCS theory of bilayer capacitance [3].

Our results show that INM responses differ between ON and OFF RGCs. In particular, OFF cells have a prolonged depolarisation in response to millisecond timescale heat pulses, whilst ON cells exhibit a short depolarisation with a larger post-pulse hyperpolarisation. This difference is mainly due to the low voltage activated calcium current that is present in OFF and absent in ON RGCs. This prediction is yet to be confirmed experimentally, but may have important implications for the development of infrared retinal prostheses.

**References**

1. Fohlmeister JF, Miller RF. Impulse encoding mechanisms of ganglion cells in the tiger salamander retina. Journal of Neurophysiology. 1997; 78(4): 1935-47.
2. Wang XJ, Rinzel J, Rogawski MA. A model of the T-type calcium current and the low-threshold spike in thalamic neurons. Journal of Neurophysiology. 1991; 66(3): 839-50.
3. Eom K, Byun KM, Jun SB, Kim SJ, Lee J. Theoretical study on gold-nanorod-enhanced near-infrared neural stimulation. Biophysical Journal. 2018; 115(8): 1481-97.

## P93 Investigation of Stimulation Protocols in Transcutaneous Vagus Nerve Stimulation (tVNS)

### Charlotte Keatch^1^, Paul Stoddart^2^, Elisabeth Lambert^3^, Will Woods^4^, Tatiana Kameneva^5^

#### ^1^Swinburne University of Technology, Biomedical Engineering, Melbourne, Australia; ^2^Swinburne University of Technology, ARC Training Centre in Biodevices, Melbourne, Australia; ^3^Swinburne University of Technology, Department of Health and Medical Sciences, Melbourne, Australia; ^4^Swinburne University of Technology, Faculty of Health, Arts and Design, Melbourne, Australia; ^5^Swinburne University of Technology, Telecommunication Electrical Robotics and Biomedical Engineering, Melbourne, Australia

##### **Correspondence:** Charlotte Keatch (ckeatch@swin.edu.au)

*BMC Neuroscience* 2020, **21(Suppl 1)**:P93

Transcutaneous vagus nerve stimulation (tVNS) is a type of non-invasive brain stimulation that is used increasingly in the treatment of a number of different health conditions such as epilepsy and depression. Although there is a great deal of research into different medical conditions that can be improved by tVNS there is little conclusive evidence into the optimal stimulation parameters, such as stimulation frequency, pulse type or amplitude. Understanding whether variation of these stimulation parameters can directly influence the brain response could improve treatment delivery.

The aim of this project is to determine whether varying the stimulation parameters of tVNS can influence the induced brain response, and if there is an optimal set of stimulation parameters that can be determined for targeted treatment of different medical conditions.

Twenty healthy participants were selected based on their suitability for both magnetoencephalography (MEG) and magnetic resonance imaging (MRI) based on predetermined exclusion criteria. The experimental sessions were carried out at the Swinburne Imaging Facility, Swinburne University of Technology. Four different stimulation protocols were delivered via electrical stimulation to the left ear; active stimulation to the cymba concha at stimulation frequency of 24 Hz regular pulses, sham stimulation to the ear lobe at stimulation frequency of 24 Hz regular pulses, stimulation to the cymba concha at stimulation frequency of 1 Hz regular pulses, and stimulation to the cymba concha at stimulation frequency of 24 Hz pulse frequency modulated (PFM) pulses (modulated at 6 Hz).

Participant brain dynamics were analysed in response to stimulation through different signal processing techniques. First the raw data was passed through the software MaxFilter which uses Signal Space Separation (SSS) of Maxwell’s equations to remove major sources of noise and artifacts. The stimulation artifact was then removed from the data by spline interpolation, which removed part of the data from the onset of the stimulation pulse and then interpolated to reconstruct the signal. The data was then downsampled and filtered before applying Fast Fourier Transforms (FFT) to obtain power spectrums at sensor level. The response to different protocols could be contrasted by taking ratios for all participants and was then averaged to see group response at sensor level.

Preliminary results show that results vary between individuals, with different brain areas activated due to the stimulation. Comparison between the active stimulation of the vagus nerve at 24Hz to sham stimulation of the ear lobe shows a dipole response to the active stimulation in the parietal lobe. Comparison of the PFM stimulation with the regular 24 Hz stimulation of the vagus nerve shows an inhibited response in the modulation frequency of 6 Hz in comparison with other frequency bands. Finally, comparing the 1 Hz with the 24 Hz active stimulation of the vagus nerve shows that the 1 Hz stimulation drives the brain more strongly than the 24 Hz across all frequency bands. These preliminary results may be used as a stepping-stone to investigate the effect of tVNS on brain dynamics and setting up stimulation protocols that may have therapeutic effects.

## P94 Brain rhythms enhance top-down influence on V1 responses in perceptual learning

### Ryo Tani^1^, Yoshiki Kashimori^2^

#### ^1^The University of Electro-Communications, Tokyo, Japan; ^2^The University of Electro-Communications, Department of Engineering Science, Tokyo, Japan

##### **Correspondence:** Ryo Tani (r.tani@uec.ac.jp)

*BMC Neuroscience* 2020, **21(Suppl 1)**:P94

Visual information is conveyed in a feedforward manner to progressively higher levels in the hierarchy, beginning with the analysis of simple attributes, such as orientation and contrast, and leading to more complex object features from one stage to the next. In contrast, visual systems have abundant feedback connections, whose number is even larger than the feedforward ones. Top-down influences, conveyed by the feedback pathways across entire brain areas, modulate the responses of neurons in early visual areas, depending on cognition and behavioral context. Li et al. [1] showed that top-down signals allowed neurons of the primary visual cortex (V1) to engage stimulus components that were relevant to a perceptional task and to discard influences from components that were irrelevant to the task. They showed that V1 neurons exhibited characteristic tuning patterns depending on the array of stimulus components. Ramalingam et al. [2] further examined dynamic aspects of V1 neurons in the tasks used by Li et al., and revealed the difference in the dynamic correlations between V1 responses evoked by the two tasks. Using a V1 model, we also proposed the neural mechanism of the tuning modulations by top-down signal [3].Top-down and bottom-up information are processed with different brain rhythms. Fast oscillations such as gamma rhythms are involved in sensory coding and feature binding in local circuits, while slower oscillations such as alpha and beta rhythms are evoked in higher brain areas and may contribute to the coupling of distinct brain areas. In this study, we investigate how information of top-down influence is conveyed by feedback pathway, and how information relevant to task context is coordinated by different brain oscillations. We present a model of visual system which consists of networks of V1 and V2. We consider the two types of perceptual tasks used by Li et al., bisection task and vernier one. We show that visual information relevant to each task context is coordinated by a push-pull effect of top-down signal. We also show that top-down signal reflecting a beta oscillation in V2 neurons, coupled with a gamma oscillation of V1 neurons, enable the efficient gating of task-relevant information in V1. This study provides a useful insight to understanding how rhythmic oscillations in distinct brain areas are coupled to gate task-relevant information encoded in early sensory areas.

**References**

1. Li W, Piech V, and Gilbert C D. Perceptual learning and top-down influences in primary visual cortex. Nature Neuroscience 2004; 7(6): 651-657.
2. Ramalingam N, McManus JNJ, Li W, and Gilbert CD. Top-Down Modulation of Lateral Interactions in Visual Cortex. Journal of Neuroscience. 2013; 33(5): 1773-1789.
3. Kamiyama A, Fujita K, and Kashimori Y. A neural mechanism of dynamic gating of task-relevant information by top-down influence in primary visual cortex. BioSystems. 2016; 150: 138-148.

## P95 A functional role of short-term synapses in maintenance of gustatory working memory in orbitofrontal cortex

### Layla Antaket^1^, Yoshiki Kashimori^2^

#### ^1^University of Electro-Communications, Tokyo, Japan; ^2^The University of Electro-Communications, Department of Engineering Science, Tokyo, Japan

##### **Correspondence:** Layla Antaket (a1943002@edu.cc.uec.ac.jp)

*BMC Neuroscience* 2020, **21(Suppl 1)**:P95

Taste perception is an important function for life activities, such as ingestion of nutrition and escape of toxic foods. Gustatory information is first processed by taste receptors in the taste buds present in the tongue. After that, it is transmitted to the orbitofrontal cortex (OFC), the hypothalamus, and the amygdala. In the course of a series of information processing processes, the gustatory cortex (GC) processes information on the quality and strength (concentration) of taste itself. Currently, taste research is proceeding with electrophysiological and molecular biological research on receptors. However, the processing mechanism of taste information encoded in each part of the taste transmission pathway is not well understood.

Furthermore, in addition to the higher-order processing of taste information, the OFC, located above the GC, integrates taste information and other sensory information such as tactile sensation, smell, and color to determine the flavor (flavor) of food and guide behavior. We proposed a binding mechanism of taste and odor information in the OFC [1]. A recent study has shown an alternative function of OFC, or working memory function of taste information [2]. The study showed that OFC neurons of the rhesus monkeys encoded a gustatory working memory in a delayed match-to-sample task. OFC neurons exhibited a persistent activity even when a gustatory stimulus presented in the sample period was turned off, whereas neurons of the primary gustatory cortex (GC) did not show a significant persistency of the activity. It is unclear how the gustatory working memory in the OFC is shaped by the interaction between the GC and the OFC.

To address this issue, we focus on a delayed match-to-sample task, in which monkeys have to decide whether the first juice stimulus is the same as the second stimulus separated by a delay period. We develop a model of gustatory system that consists of network models of GC and OFC. Each model of GC and OFC has two-dimensional array of neurons, which encode information of three kinds of foods, orange, guava, and tomato. These network models were based on the Izhikevich neuron model [3] and biophysical synapses mediated by neurotransmitters such as AMPA, NMDA, and GABA. The neural unit consists of a main neuron and an inhibitory interneuron, mutually connected with AMPA and GABA synapses. Main neurons are reciprocally connected with AMPA and NMDA synapses. The NMDA-synaptic connections between these networks are formed by Hebbian learning in a task-relevant way. The gustatory information of three foods is represented by dynamical attractors in the GC and OFC networks. Simulating our model for match/nonmatch trails, we explored the neural mechanism by which the working memory of gustatory information is generated in the OFC. We show that the working memory of gustatory information is shaped by the recurrent activation mediated by short-term synapses of OFC neurons. In addition, we examined how working memory formed by the OFC is used for match/nonmatch decision-making by adding a decision layer to the model.

**References**

1. Shimemura T, Fujita K, Kashimori Y. A neural mechanism of taste perception modulated by odor information. Chemical Senses. 2016; 41(7): 579-589.
2. Lara AH, Kennerley SW, Wallis JD. Encoding of Gustatory Working Memory by Orbitofrontal Neurons. Journal of Neuroscience. 2009; 29(3): 765-774.
3. Izhikevich EM. Simple Model of Spiking Neurons. IEEE Trans Neural Net. 2003; 14(6): 1569-1572.

## P96 Bayesian network change point detection for dynamic functional connectivity

### Lingbin Bian, Tiangang Cui, Adeel Razi, Jonathan Keith

#### Monash University, Melbourne, Australia

##### **Correspondence:** Lingbin Bian (lingbin.bian1@monash.edu)

*BMC Neuroscience* 2020, **21(Suppl 1)**:P96

We present a novel Bayesian method for identifying the change of dynamic network structure in working memory task fMRI data via model fitness assessment. Specifically, we detect dynamic community structure change-point(s) based on overlapped sliding window applied to multivariate time series. We use the weighted stochastic block model to quantify the likelihood of a network configuration, and develop a novel scoring criterion that we call posterior predictive discrepancy by evaluating the goodness of fit between model and observations within the sliding window. The parameters for this model include latent label vector assigning network nodes to interacting communities, and the block model parameter determining the weighted connectivity within and between communities. The GLM analyses were conducted in both subject level and group level and the contrast between 2-back, 0-back and baseline were used to localise the regions of interest in task fMRI data.

The working memory task fMRI data in the HCP were pre-processed and GLM analyses were applied. With the extracted time series of regions of interest, we propose to use the Gaussian latent block model [1], also known as the weighted stochastic block model (WSBM), to quantify the likelihood of a network and Gibbs sampling to sample a posterior distribution derived from this model. The Gibbs sampling approach we adopt is based on the work of [1,2] for finite mixture models. The proposed model fitness procedure draws parameters from the posterior distribution and uses them to generate a replicated adjacency matrix; then calculates a disagreement matrix to quantify the difference between the replicated adjacency matrix and realised adjacency matrix. For the evaluation of the model fitness, we define a parameter-dependent statistic called the posterior predictive discrepancy (PPD) by averaging the disagreement matrix. Then we compute the cumulative discrepancy energy (CDE) from PPD by applying another sliding window for smoothing and use CDE as a score criterion for change point detection. The CDE increases when change points are contained within the window, and can thus be used to assess whether a statistically significant change point exists within a period of time.

We first applied the algorithm to the synthetic data simulated from the Multivariate Gaussian distribution for validation. We visualise the Gibbs iteration of sampled latent labels and the histogram of the block parameters reflecting the characterisation of the connectivity within and between communities. We then demonstrated the performance of the change point detection with different window sizes. In real working memory task fMRI data analyses, the fixed effects analyses are conducted to estimate the average effect size across runs within subjects at the subject level. At group level, the mixed effects analyses are conducted, where the subject effect size is considered to be random. In this work, we mainly focus on the memory load contrast (2-back vs 0-back, 2-back vs baseline, or 0-back vs baseline).

**References**

1. Wyse J, Friel N. Block clustering with collapsed latent block models. Statistics and Computing. 2012; 22: 415-428.
2. Nobile A, Fearnside AT. Bayesian finite mixtures with an unknown number of components: the allocation sampler. Statistics and Computing. 2007; 17: 147-162.

## P97 Multiscale simulations of ischemia and spreading depolarization with NEURON

### Adam Newton^1^, Michael Hines^2^, William W Lytton^3^, Robert McDougal^4^, Craig Kelley^3^

#### ^1^Yale University, Yale School of Public Health, Connecticut, United States of America; ^2^Yale University, School of Medicine, Connecticut, United States of America; ^3^SUNY Downstate Medical Center, Department of Physiology and Pharmacology, New York, United States of America; ^4^Yale University, Department of Neuroscience, Connecticut, United States of America

##### **Correspondence:** Adam Newton (adam.newton@yale.edu)

*BMC Neuroscience* 2020, **21(Suppl 1)**:P97

Recent improvements and performance enhancements in the NEURON (neuron.yale.edu) reaction-diffusion module (rxd) allow us to model multiple relevant concentrations in the intracellular and extracellular space. The extracellular space is a coarse-grained macroscopic model based on a volume averaging approach, allowing the user to specify both the free volume fraction (the proportion of space in which species are able to diffuse) and the tortuosity (the average multiplicative increase in path length due to obstacles). These tissue characteristics can be spatially dependent to account for regional or pathological differences.

Using a multiscale modeling approach we have developed a pair of models for spreading depolarization at spatial scales from microns to mm, and time scales from ms to minutes. The cellular/subcellular-scale model adapted existing mechanisms for a morphologically detailed CA1 pyramidal neuron together with a simple astrocyte model. This model included reaction-diffusion of K+, Na+, Cl− and glutamate, with detailed cytosolic and endoplasmic reticulum Ca2+ regulation. Homeostatic mechanisms were added to the model, including; Na-K- ATPase pumps, Ca2+ pumps, SERCA, NKCC1, KCC2 and glutamate transporters. We use BluePyOpt to perform a parameter search, constrained by the requirements of realistic electrophysiological responses while maintaining ionic homeostasis. This detailed model was used to explore the hypothesis that individual dendrites have distinct vulnerability to damage due to area-volume ratios leading to different intracellular Ca2+ levels.

At the tissue-scale we adapted a simpler point neurons model, and densely packed them in a coarse-grained macroscopic 3D volume. The models include a simple model for oxygen and dynamic changes in volume fraction. This allows us to model the effect of changes in tissue diffusion characteristics on the wave propagation during spreading depolarization.

**Acknowledgments:** Research supported by NIH grant R01MH086638.

## P98 Bayesian mechanics in the brain under the free-energy principle

### Chang S Kim

#### Chonnam National University, Department of Physics, Gwangju, South Korea

##### **Correspondence:** Chang S Kim (cskim@jnu.ac.kr)

*BMC Neuroscience* 2020, **21(Suppl 1)**:P98

In the field of neurosciences, the free-energy principle (FEP) stipulates that all viable organisms cognize and behave using probabilistic models embodied in their brain in a manner that ensures their adaptive fitness in the environment [1].

Here, we report on our recent theoretical study that supports the use of the FEP as a more physically plausible theory, based on the principle of least action [2]. We recapitulate the FEP carefully [3] and evaluate that some technical facets in its conventional formalism require reformulation with finesse [4]. Accordingly, we articulate the FEP as living organisms minimize the sensory uncertainty, which is the average surprisal over a temporal horizon, and reformulate the recognition dynamics of the brain’s ability for actively inferring the external causes of sensory inputs. We effectively cast the Bayesian inversion problem in the organism’s brain to find the optimal neural trajectories by minimizing the time integral of the informational free energy (IFE), which is the upper bound of the long-term average surprisal. Specifically, we abstain from i) the non-Newtonian extension of continuous states, which yields the generalized motion, by recursively taking higher-order derivatives of the sensory observation and state equations, and ii) the heuristic gradient-descent minimization of the IFE in a moving frame of reference in a generalized-state space by viewing the nonequilibrium dynamics of brain states as drift-diffusion flows that locally conserve the probability density. The advantage of our formulation is that only bare variables (positions) and their first-order derivatives (velocities) are used in the Bayesian neural computation, thereby dismissing the need for the extra-physical assumptions.

Bare variables are an organism’s representations of the causal environment, and their conjugate momenta resemble the precision-weighted prediction errors in a predictive coding language [5].

Furthermore, we consider the sensory-data-generating dynamics to be nonstationary on an equal footing with intra- and inter-hierarchical-level dynamics in a neuronally based biophysical model.

Consequently, our theory delivers a natural account of the descending predictions and ascending prediction errors in the brain’s hierarchical message-passing structure (Fig. 1).

**Fig. 1:**
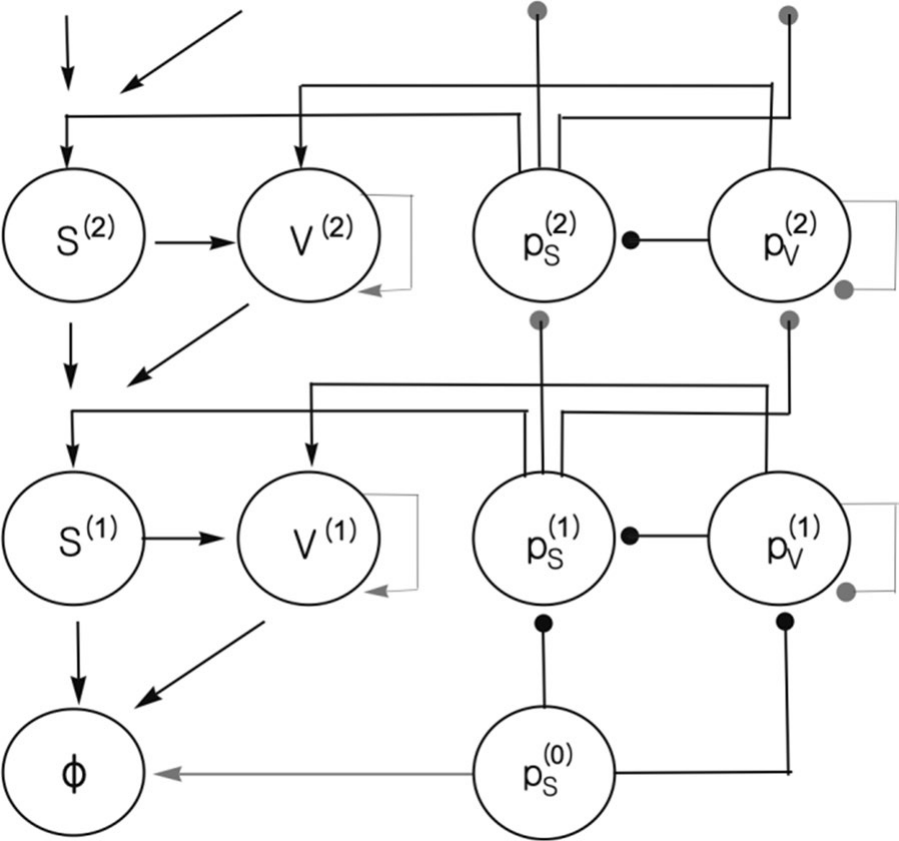
Schematic of the neural circuitry [4], where each cortical level is specified by the perceptual states (S(i),V(i)) and their conjugate momenta (Ps(i),Pv(i)). The prediction error Ps(0) of incoming sensory data at the lowest level induces an inhibitory change in the perceptual momenta (Ps(1),Pv(1)). Subsequently, the prediction error propagates up the hierarchy

The ensuing neural circuitry may be related to the alpha-beta and gamma rhythms that characterize the feedback and feed-forward influences, respectively, in the primate visual cortex [6].

**References**

1. Friston K. The free-energy principle: a unified brain theory? Nature reviews neuroscience. 2010; 11(2): 127-38.
2. Landau LD, Lifshitz EM. Mechanics: Volume 1 (Course of Theoretical Physics S). 3rd edition. Amsterdam: Elsevier Ltd.; 1976.
3. Buckley CL, Kim CS, McGregor S, Seth AK. The free energy principle for action and perception: A mathematical review. Journal of Mathematical Psychology. 2017; 81: 55-79.
4. Kim CS. Recognition dynamics in the brain under the free energy principle. Neural computation. 2018; 30(10): 2616-59.
5. Rao RP, Ballard DH. Predictive coding in the visual cortex: a functional interpretation of some extra-classical receptive-field effects. Nature neuroscience. 1999; 2(1): 79-87.
6. Michalareas G, et al. Alpha-beta and gamma rhythms subserve feedback and feedforward influences among human visual cortical areas. Neuron. 2016; 89(2): 384-97.

## P99 Integrated model of reservoir computing and autoencoder for explainable artificial intelligence

### Hoon-Hee Kim

#### Korea Advanced Institute of Science and Technology, Daejeon, South Korea

##### **Correspondence:** Hoon-Hee Kim (hkma19gm@kaist.ac.kr)

*BMC Neuroscience* 2020, **21(Suppl 1)**:P99

Due to the development of machine learning such as a deep neural network, Artificial Intelligence (AI) has been used in many areas. Modern AI technology accurately solves problems such as classification, regression, and prediction, but there is a lack of skill to explain the process of AI decision in terms of human understanding; it is called a black-box AI. The black-box AI, in which humans cannot understand the decision process, is difficult to use in high-risk areas such as important social and legal decisions, medical diagnosis, and financial predictions [1]. Although there are highly explainable machine learning methods such as a decision-tree, these machine learning methods tend to has a low performance and are not suitable for solving a complex problem [2]. In this study, I suggest a novel explainable AI method which has a high performance based on an integrated model of Reservoir Computing and Autoencoder. Reservoir Computing, a recurrent neural network consists of three layers: inputs, reservoir, and readouts can train nonlinear dynamics using linear learning methods [3]. Recently, a study was published in which neural networks induced actual physical laws using Variational Autoencoder which can extract interpretable features of the learning data [4]. In the integrated model, the features of the training data were learned by the autoencoder structure and linear learning rule of reservoir computing. Therefore, these features could be represented as a linear formula form that a human can simply understand. To validate the integrated model, I tested the model to predict trends of the S & P500 index. The model showed more than 80% accuracy and reported that which features were most important to the prediction in terms of weighted linear formula.

**Acknowledgments**: This study was supported by the National Research Foundation of Korea [NRF-2019R1A6A3A01096892].

**References**

1. Rudin C. Stop explaining black box machine learning models for high stakes decisions and use interpretable models instead. Nature Machine Intelligence. 2019; 1(5): 206-215.
2. Defense Advanced Research Projects Agency. Broad Agency Announcement, Explainable Artificial Intelligence. 2016. At: https://www.darpa.mil/attachments/DARPA-BAA-16-53.pdf
3. Jaeger H, Haas H. Harnessing nonlinearity: Predicting chaotic systems and saving energy in wireless communication. Science. 2004; 304(5667): 78-80.
4. Iten R, Metger T, Wilming H, Del Rio L, Renner R. Discovering physical concepts with neural networks. Physical Review Letters. 2020; 124(1): 010508.

## P100 Role of breathing signals and intra-vIRt connectivity in the generation of vibrissa movements

### David Golomb^1^, Jeffrey Moore^2^, Arash Fassihi^3^, Fan Wang^4^, David Kleinfeld^3^

#### ^1^Ben Gurion University, Physiology, Be’er-Sheva, Israel; ^2^Harvard University, Molecular and Cellular Biology, Cambridge, Massachusetts, United States of America; ^3^University of California San Diego, Physics, San Diego, California, United States of America; ^4^Duke University, Neurobiology, Durham, North Carolina, United States of America

##### **Correspondence:** David Golomb (golomb@bgu.ac.il)

*BMC Neuroscience* 2020, **21(Suppl 1)**:P100

The vIRt nucleus in the medulla, apparently composed of mainly inhibitory neurons, is necessary for whisking rhythm generation. It innervates motoneurons in the facial nucleus (FN) that project to intrinsic vibrissa muscles. The nearby pre-Bötzinger complex (pBötC), which generates inhalation, sends inhibitory inputs to the vIRt nucleus. We explore potential mechanisms of this vIRt synchronization, needed for whisking, using analysis of experimental data and computational modeling. Using time courses of breathing and whisking in rats, we compute the relative amplitude An of n-th whisking cycles within a breathing cycle. For head-restrained rats, the average values of An/A1 are 0.56, 0.46 and 0.43 of the amplitudes for n = 2, 3, 4. For freely behaving rats, the value for A2/A1is 0.79. The observations that A1 is larger than subsequent amplitudes suggests that the recovery of vIRt neurons from inhibition by the pBötC contributes to the synchronization of vIRt neurons. Lower-amplitude periodic whisking, however, can occur after decay of the pBötC signal. To explain how vIRt network generates these “intervening” whisks, and why the amplitude A1is larger than the following amplitudes, we construct and analyze a conductance-based model of the vIRt circuit composed of hypothetical two groups, vIRtr and vIRtp, of bursting inhibitory neurons with spike-frequency adaptive currents and constant external excitation. Only neurons in vIRtr are inhibited by the pBötC and inhibit FN motoneurons. We denote the strengths of the inhibitory conductances between neurons across and within each group by gas and gs, respectively. If gas is larger than gs, the two groups burst alternately, as observed experimentally. If gas is too large, however, one group is active and the second is silent. The oscillation amplitude depends linearly on the constant external excitation, and the period increases as a function of gas-gs. Thus, the external input to the circuit and the level of inhibition within the circuit control the amplitude and frequency of the intervening whisking, respectively. Our model thus provides a means to control the wide range of whisking frequencies observed in experiments.

**Acknowledgements:** supported by NIH grant 5U19NS107466-02.

## P101 Representing predictability of sequence patterns in a random network with short-term plasticity

### Vincent S C Chien, Richard Gast, Burkhard Maess, Thomas Knösche

#### Max Planck Institute for Human Cognitive and Brain Sciences, Leipzig, Germany

##### **Correspondence:** Thomas Knösche (knoesche@cbs.mpg.de)

*BMC Neuroscience* 2020, **21(Suppl 1)**:P101

The brain is capable of recognizing repetitive acoustic patterns within a few repetitions, which is essential for the timely identification of sound objects and the prediction of upcoming sounds. Several studies found neural correlates regarding the predictability of sequence patterns, but the underlying neural mechanism is not yet clear. To investigate the mechanism supporting the fast emergence of the predictive state, we use neural mass modeling to replicate the experimental observations during the sequential repetition [1]. First, we investigated the effect of short-term plasticity (STP) to the response of a Wilson-Cowan node to a prolonged stimulus, where the node consists of an excitatory (E) and an inhibitory (I) population. In total, 27 combinations of plasticity settings were examined, where the plasticity types include short-term depression (STD), short-term facilitation (STF), and no STP, and the connection types include E-to-E, E-to-I, I-to-E, and I-to-I connections. The simulated signals that best explain the observed MEG temporal profiles (i.e., an onset peak followed by a rising curve) rely on the setting where STD is applied on E-to-E connection and STF applied on E-to-I connection. Second, with the preferred plasticity settings (i.e., STD on E-to-E and STP on E-to-I), we simulated the dynamics of a random network in response to regular (REG) and random (RAND) sequences in PyRates [2]. The simulated signals can reproduce several experimental observations, including the above-mentioned MEG temporal profiles, the predictability-dependent MEG amplitude (i.e., dependency in terms of regularity and alphabet size of the input sequence), as well as the MEG responses in the switch conditions (i.e., from REG to RAND, and from RAND to REG). Third, we used a simplified two-level network to illustrate the main mechanisms supporting such representation of predictability during the sequential repetition. The simplified network consists of nodes that are selective to sound tone (level 1) and nodes that are selective to tone direction (level 2). The simulation reveals higher firing rates of I populations level-2 nodes during REG than RAND condition, which contributes to stronger simulated MEG amplitude via I-to-E connections (Fig 1). In conclusion, we provide a possible mechanism to account for the experimental observations. First, the increased MEG amplitude is mainly due to increased inhibitory activities. Second, the effect of alphabet size is due to two forms of STP (i.e., STD on E-to-E and STF on E-to-I). Third, the effect of regularity relies on the inclusion of the 2nd-level nodes that sparsely encodes the repetitive patterns. In short, the more predictable sequence patterns cause a stronger accumulation of inhibitory activities in direction-selective areas via STP, which in turn leads to a higher MEG amplitude. This mechanism emphasizes the need for STP at each stage of the bottom-up process, whereas the involvement of top-down processes is not necessary.

**References**

1. Barascud N, Pearce MT, Griffiths TD, Friston KJ, Chait M. Brain responses in humans reveal ideal observer-like sensitivity to complex acoustic patterns. Proceedings of the National Academy of Sciences. 2016; 113(5): E616-25.
2. Gast R, et al. PyRates—A Python framework for rate-based neural simulations. PloS one. 2019; 14(12): e0225900.

## P102 Ephaptic coupling in white matter fibre bundles modulates axonal transmission delays

### Helmut Schmidt^1^, Gerald Hahn^2^, Gustavo Deco^3^, Thomas Knösche^1^

#### ^1^Max Planck Institute for Human Cognitive and Brain Sciences, Brain Networks, Leipzig, Germany; ^2^Universitat Pompeu Fabra, Computational Neuroscience Group, Barcelona, Spain; ^3^Universitat Pompeu Fabra, Barcelona, Spain

##### **Correspondence:** Thomas Knösche (knoesche@cbs.mpg.de)

*BMC Neuroscience* 2020, **21(Suppl 1)**:P102

Axonal connections are widely regarded as faithful transmitters of neuronal signals with fixed delays. The reasoning behind this is that local field potentials (LFPs) caused by spikes travelling along axons are too small to have an effect on other axons. We demonstrate that, although the local field potentials generated by single spikes are of the order of microvolts, the collective local field potential generated by spike volleys can reach several millivolts. As a consequence, the resulting depolarisation of the axonal membranes (i.e. ephaptic coupling) increases the velocity of spikes, and therefore reduces axonal transmission delays between brain areas.

We first compute the local field potential using the line approximation [1,2] for a spike in a single axon. We find that it generates an LFP with about 20 microvolts amplitude, which is too weak to have a significant effect on neighbouring axons (Fig. 1A). Next, we extend this formalism to fibre bundles to compute the LFP generated by spike volleys, with different levels of synchrony. Such spike volleys can generate LFPs with amplitudes of several millivolts (Fig. 1B), and the amplitude of the LFP depends strongly on the level of synchrony of the spike volley. Finally, we devise a spike propagation model in which the LFPs generated by spikes modulate their propagation velocity. This model reveals that with increasing number of spikes in a spike volley, the axonal transmission delays decrease (Fig. 1C). To the best of our knowledge, this study is the first that investigates the effect of LFPs on axonal signal transmission in macroscopic fibre bundles. The main result is that axonal transmission delays decrease if spike volleys are sufficiently large and synchronous. This is in contrast to studies investigating ephaptic coupling between spikes at the microscopic level (e.g. [3]), which have used a different model setup that resulted in increasing axonal transmission delays. Our results are a possible explanation for the decreasing stimulus latency with increasing stimulus intensity observed in many psychological experiments (e.g. [4]). We speculate that the modulation of axonal transmission delays contributes to the flexible synchronisation of high frequency oscillations (e.g. gamma oscillations).

**Fig. 1:**
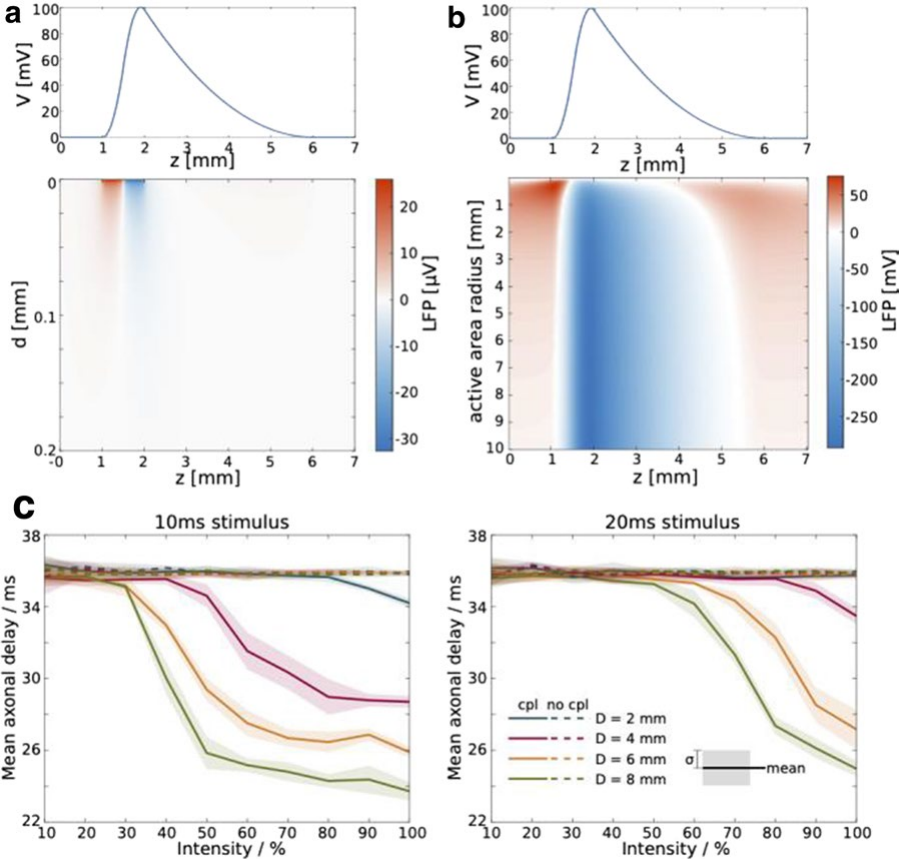
**A** Spike profile (top) and resulting LFP in a single axon (bottom). **B** Spike profile (top) and LFP generated by fully synchronised spike volley (bottom). **C** Axonal delays as function of number of spikes and bundle radius in the presence (solid) and absence (dashed) of ephaptic coupling

**Acknowledgements:** This work has been supported by the German Research Foundation (DFG), SPP2041.

**References**

1. Holt GR, Koch C. Electrical interactions via the extracellular potential near cell bodies. Journal of Computational Neuroscience. 1999; 6 :169-184.
2. McColgan T, et al. Dipolar extracellular potentials generated by axonal projections. eLife. 2017, 6: e25106.
3. Binczak S, Eilbeck JC, Scott AC. Ephaptic coupling of myelinated nerve fibres. Physica D. 2001; 148: 159-174
4. Ulrich R, Rinkenauer G, Miller J. Effects of stimulus duration and intensity on simple reaction time and response force. Journal of Experimental Psychology. 1998; 24: 915-928.

## P103 Constructing model surrogates of populations of dopamine neuron and medium spiny neuron models for understanding phenotypic differences

### Tim Rumbell^1^, Sushmita Allam^2^, Tuan Hoang-Trong^2^, Jaimit Parikh^2^, Viatcheslav Gurev^2^, James Kozloski^2^

#### ^1^IBM Research, Healthcare and Life Sciences, Raleigh, North Carolina, United States of America; ^2^IBM Research, Yorktown Heights, New York, United States of America

##### **Correspondence:** Tim Rumbell (timrumbell@gmail.com)

*BMC Neuroscience* 2020, **21(Suppl 1)**:P103

Neurons of a specific type have intrinsic variety in their electrophysiological properties. Intracellular parameters, such as ion channel conductances and kinetics, also have high variability within a neuron type, yet reliable functions emerge from a wide variety of parameter combinations. Recordings of electrophysiological properties from populations of neurons under different experimental conditions or perturbations produce sub-groups that form “electrophysiological phenotypes”. For example, different properties may derive from wild-type vs. disease model animals or may change across multiple age groups [1].

Populations of neuron models can represent a neuron type by varying parameter sets, each able to produce the outputs of a recording, and all spanning the ranges of recorded features. We previously generated model populations using evolutionary search with an error function that combines soft-thresholding with a crowdedness penalty in feature space, allowing coverage of the empirical range of features with models. The technique was used to generate a population of dopamine neuron (DA) models, which captured the majority of empirical features, generalized to perturbations, and revealed sets of coefficients predicted to reliably modulate activity [2]. We also used this technique to construct striatal medium spiny neuron (MSN) model populations, which recapitulated the effects of extracellular potassium changes [3] and captured differences in electrophysiological phenotype between MSNs from wild-type mice and from the Q175 model of Huntington’s disease. Our approach becomes prohibitively computationally expensive, however, when we seek to produce multiple populations that represent many phenotypes from across a spectrum. For example, to recreate the non-linear developmental trajectory observed across postnatal development of DAs [1] we would need to perform multiple optimizations.

Here we demonstrate the construction of model surrogates that map model parameters to features spanning the range of multiple electrophysiological phenotypes. We sampled from parameter space and simulated models to create a surrogate training set. Using our evolutionary search as prior knowledge of our parameter space enabled a dense sampling in regions of the high-dimensional model parameter space that were likely to produce valid features. We trained a deep neural network with our datasets, producing a surrogate for our model that maps parameter set distributions to output feature distributions. This can be used in place of the neuron model for model sampling, allowing rapid construction of populations of models that match different distributions of features from across multiple phenotypes. We demonstrate this approach using DA developmental age groups and MSN disease progression states as targets, facilitating a mechanistic understanding of parameter modulations that generate differences in phenotypes.

**References**

1. Dufour MA, Woodhouse A, Amendola J, Goaillard JM. Non-linear developmental trajectory of electrical phenotype in rat substantia nigra pars compacta dopaminergic neurons. Elife. 2014; 3: e04059.
2. Rumbell T, Kozloski J. Dimensions of control for subthreshold oscillations and spontaneous firing in dopamine neurons. PLoS Computational Biology. 2019; 15(9): e1007375.
3. Octeau JC, et al. Transient, consequential increases in extracellular potassium ions accompany channelrhodopsin2 excitation. Cell reports. 2019; 27(8): 2249-61.

## P104 Integrated cortical, thalamic and basal ganglia model of brain function: validation against functional requirements

### Sébastien Naze^1,2^, James Kozloski^2^

#### ^1^IBM Research, Melbourne, Australia; ^2^IBM Research, Yorktown Heights, New York, United States of America

##### **Correspondence:** Sébastien Naze (sebastien.naze@gmail.com)

*BMC Neuroscience* 2020, **21(Suppl 1)**:P104

Large scale brain models encompassing cortico-cortical, thalamo-cortical and basal ganglia processing are fundamental to understand the brain as an integrated system in healthy and disease conditions but are complex to analyze and interpret. Neuronal processes are typically segmented by region and modality in order to explain an experimental observation at a given scale and then integrated to a global framework [1]. Here, we present a set of functional requirements applied to validate the recently developed IBEx model [2] against a learning task involving coordinated activity across cortical and sub-cortical regions in a brain-computer interface (BCI) context involving volitional control of a sensory stimulus [3]. The original IBEx model comprises interacting modules for supra-granular, infra-granular cortical layers, thalamic integration, basal ganglia parallel processing and dopamine-mediated reinforcement learning. We decompose and analyze each subsystem in the context of the BCI learning task whereby parameters are tuned to comply to its functional requirements. Intermediate conclusions are presented for each subsystem according to the constraints imposed to satisfy the requirements, before re-incorporating the subsystem in the global framework. Consequences of model modifications and parameter tuning are assessed at the scales of the subsystem and the whole brain system. The relation between infra-granular spiking activity in different cortical regions, thalamo-cortical delta rhythms and higher-level description of cognitive or motor trajectories (according to the brain region) is displayed. The relation to phenotypes associated to Huntington’s disease is exposed and the framework is discussed in perspective to other state-of-art integrative efforts to understand complex high-order brain functions [4,5].

**References**

1. Eliasmith C, Trujillo O. The use and abuse of large-scale brain models. Current Opinion in Neurobiology. 2014; 25: 1-6.
2. Kozloski J. Closed-loop brain model of neocortical information-based exchange. Frontiers in Neuroanatomy. 2016; 10: 3.
3. Koralek AC, Jin X, Long II JD, Costa RM, Carmena JM. Corticostriatal plasticity is necessary for learning intentional neuroprosthetic skills. Nature. 2012; 483(7389): 331-5.
4. Oizumi M, Albantakis L, Tononi G. From the phenomenology to the mechanisms of consciousness: integrated information theory 3.0. PLoS Computational Biology. 2014; 10(5): e1003588.
5. Mashour GA, Roelfsema P, Changeux JP, Dehaene S. Conscious processing and the global neuronal workspace hypothesis. Neuron. 2020; 105(5): 776-98.

## P105 Generating executable mushroom body and lateral horn circuits from the hemibrain dataset with FlyBrainLab

### Aurel A Lazar, Mehmet K Turkcan, Yiyin Zhou

#### Columbia University, Electrical Engineering, New York, United States of America

##### **Correspondence:** Yiyin Zhou (yiyin@ee.columbia.edu)

*BMC Neuroscience* 2020, **21(Suppl 1)**:P105

We introduce executable circuits for two higher order olfactory processing centers in the brain of Drosophila melanogaster implicated in learning and memory, the lateral horn (LH) and mushroom body (MB). Despite the large amounts of data available on these two neuropils, there is a dearth of executable neural circuit models governing learning and memory in the MB and the LH. We use the recently-released Hemibrain dataset [1] as a biological basis of our implementations to analyze cell types, numbers and connectivity for realizing our computational models for both neuropils. Our implementations utilize the FlyBrainLab environment [2] to deliver the capability to visualize neural circuits morphologically or with diagrams, can run on GPUs, and are designed to facilitate customization of neuron and synapse models at a per-cell and per-cell type level.

We first study the neural types and connectivity of mushroom body neurons, a neuropil that implements the capability for associative learning. We model individual lobes that comprise the mushroom body and each of their so-called compartments, along with connectivities between the so-called Kenyon cells (KCs), mushroom body output neurons (MBONs) and dopaminergic neurons (DANs), thereby providing an interactive circuit diagram that allows for customizable ablation or activation experiments (Fig. 1). Implementing an interface between the input to the MB from antennal lobe projection neurons (PNs), we utilize the observed PN-to-KC connectivity and provide comparisons against randomly generated instantiations of PN-to-KC connectivity, a long standing hypothesis about the nature of PN-to-KC connectivity.

**Fig. 1:**
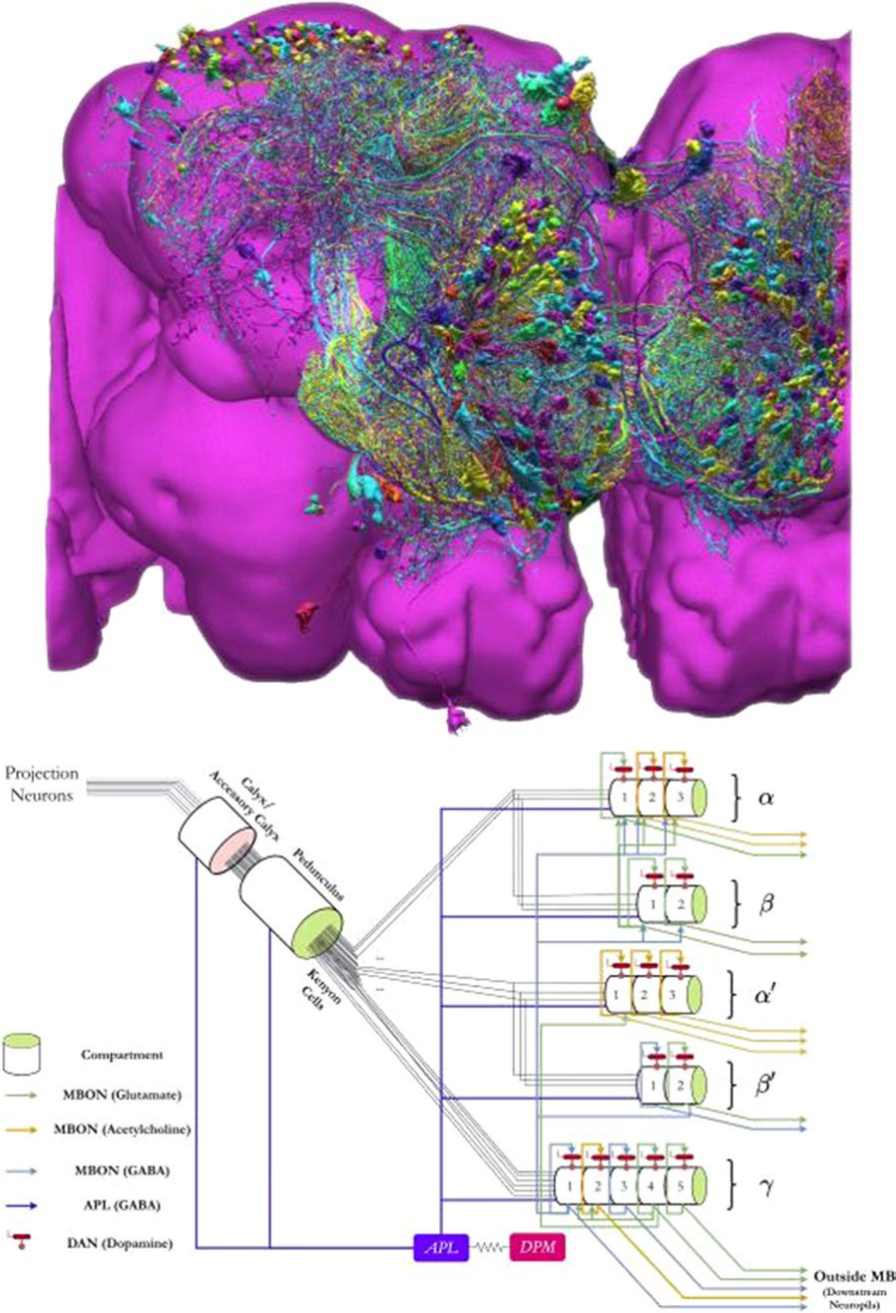
Visualization and exploration of the entire MB circuit in the Hemibrain dataset using FlyBrainLab (top). Customizable circuit diagram of the mushroom body (bottom)

Second we investigate the connectivity within the LH, a neuropil associated with innate memories, and the connectivity between the MB and the LH. We analyze in detail the connectivity between PNs, lateral horn local neurons and output neurons. We construct an executable circuit of the LH based on our analysis.

The implementations we provide are a step towards building integrated models of sensory systems derived from biological data. Our approach showcases the impact an integrative ecosystem can have on building executable circuit models for understanding the functional logic of neurocomputation in the brain of model organisms.

**Acknowledgments:** The research reported here was supported by DARPA under contract #HR0011-19-9-0035.

References

1. Xu CS, et al. A connectome of the adult drosophila central brain. BioRxiv. 2020.
2. Turkcan MK, et al. FlyBrainLab: an interactive computing environment for the fruit fly brain. Society for Neuroscience Meeting. 2019.

## P106 Efficient communication in distributed simulations of spiking neuronal networks with gap junctions

### Jakob Jordan^1^, Moritz Helias^2^, Markus Diesmann^2^, Susanne Kunkel^3^

#### ^1^University of Bern, Department of Physiology, Bern, Switzerland; ^2^Jülich Research Centre, Institute of Neuroscience and Medicine (INM-6, INM-10) and Institute for Advanced Simulation (IAS-6), Jülich, Germany; ^3^Norwegian University of Life Sciences, Faculty of Science and Technology, Ås, Norway

##### **Correspondence:** Markus Diesmann (diesmann@fz-juelich.de)

*BMC Neuroscience* 2020, **21(Suppl 1)**:P106

Investigating the dynamics and function of large-scale spiking neuronal networks with realistic numbers ofsynapses is made possible today by state-of-the-art simulation code that scales to the largest contemporary supercomputers. These implementations exploit the delayed and point-event like nature of the spike interaction between neurons. In a network with only chemical synapses the dynamics of all neurons is decoupled for the duration of the minimal synaptic transmission delay such that the dynamics of each neuron can be propagated independently for the duration of the minimal delay without requiring information from other neurons. Hence, in distributed simulations of such networks, compute nodes need to communicate spike data only after this period [1].

Electrical interactions, also called gap junctions at first seem to be incompatible with such a communication scheme as they couple membrane potentials of pairs of neurons instantaneously. Hahne et al. [2] however demonstrate that communication of spikes and gap-junction data can be unified using waveform-relaxation methods [3]. Despite these advances, simulations involving gap junctions scale only poorly due to a communication scheme that collects global data on each compute node. In comparison to chemical synapses, gap junctions are far less abundant. To improve scalability we exploit this sparsity by integrating the existing framework for continuous interactions with a recently proposed directed communication scheme for spikes [4]. Using a reference implementation in the NEST simulator (www.nest-simulator.org, [5]) we demonstrate excellent scalability of the integrated framework, accelerating large-scale simulations with gap junctions by more than an order of magnitude. This allows, for the first time, the efficient exploration of the interactions of chemical and electrical coupling in large-scale neuronal networks models with natural synapse density distributed across thousands of compute nodes.

**Acknowledgements:** Partly supported by Helmholtz young investigator group VH-NG-1028, European Union’s Horizon 2020 funding framework under grant agreement no. 785907 (Human Brain Project HBP SGA2) and no. 754304 (DEEP-EST), and Helmholtz IVF no. SO-092 (Advanced Computing Architectures, ACA). We would also like to acknowledge use of the JURECA supercomputer through VSR grant JINB33.

**References**

1. Morrison A, Mehring C, Geisel T, Aertsen AD, Diesmann M. Advancing the boundaries of high-connectivity network simulation with distributed computing. Neural Computation. 2005; 17(8): 1776-801.
2. Hahne J, et al. A unified framework for spiking and gap-junction interactions in distributed neuronal network simulations. Frontiers in Neuroinformatics. 2015; 9: 22.
3. Lelarasmee E, Ruehli AE, Sangiovanni-Vincentelli AL. The waveform relaxation method for time-domain analysis of large scale integrated circuits. IEEE transactions on computer-aided design of integrated circuits and systems. 1982; 1(3): 131-45.
4. Jordan J, et al. Extremely scalable spiking neuronal network simulation code: from laptops to exascale computers. Frontiers in Neuroinformatics. 2018; 12: 2.
5. Gewaltig MO, Diesmann M. Nest (neural simulation tool). Scholarpedia. 2007; 2(4): 1430.

## P107 Inferring parameters of DBS-induced short-term synaptic plasticity from in-vivo recordings of human brain

### Alireza Ghadimi^1^, Luka Milosevic^2^, Suneil Kalia^3^, Mojgan Hodaie^3^, Andres Lozano^3^, William Hutchison^4^, Milos Popovic^1^, Milad Lankarany^1^

#### ^1^University of Toronto, Institute of Biomaterials and Biomedical Engineering, Toronto, Canada; ^2^University of Tübingen, Department of Neurosurgery, Tübingen, Germany; ^3^University of Toronto, Department of Surgery, Toronto, Canada; ^4^University of Toronto, Department of Physiology, Toronto, Canada

##### **Correspondence:** Alireza Ghadimi (alireza.ghadimi@mail.utoronto.ca)

*BMC Neuroscience* 2020, **21(Suppl 1)**:P107

**Background**: Short-term synaptic plasticity (STP) is the dynamic of change in the synaptic weight with respect to the presynaptic spiking activity. Recent studies showed that STP is involved in several brain processes. Several computational works have been done on inferring STP parameters [1-4]; however, these studies utilized in-vitro signals of intracellular recordings (except Ghanbari et al.) from rodents to create their models, which is not necessarily representative of in-vivo human brain dynamics. To this end, we developed a parameter inference method which estimates parameters of STP induced by DBS, verified by experimental data obtained from intracranial recordings of single-neuron activity in surgical patients.

**Method**: To acquire spiking activity, two closely spaced microelectrodes were placed in the thalamic ventral intermediate nucleus (Vim) during awake DBS surgeries. One electrode was used to record single-unit spiking activity, while the other was used to deliver stimulation pulses at various frequencies (5Hz, 20Hz, 30Hz, 50Hz, 100Hz, 200Hz). The included data were collected from 15 patients [5]. The narrow stimulus pulses were removed, after which data were high-pass filtered to better isolate single unit activity. Using stimulus artifacts as triggers, we extracted the instantaneous firing rate of neurons in response to each DBS pulse of the 5Hz stimulation trains, and averaged over all inter-pulse intervals. Due to the low stimulation rate (i.e. 5Hz), no STP is induced in this data. The resultant waveform is equivalent to the impulse response.

In order to mimic STP behavior, we used the Tsodyks-Markram phenomenological model, which generates the postsynaptic current according to the spiking history of the presynaptic neuron [6]. To reconstruct the firing rate induced by DBS, we give the pulse train of DBS as the input of the Tsodyks-Markram model. The model generates a postsynaptic current in response to DBS pulses. We use this response to make modulated pulse trains that represent the effect of STP by changing the amplitude of each pulse. The modulated pulse train is convolved with the impulse response of the neuron in order to make an estimation of the DBS-induced firing rate. The estimated firing rate is compared with the experimental instantaneous firing rates throughout the stimulation trains at each of the other stimulation frequencies.

To achieve the true parameters of the Tsodyks-Markram model we should minimize the error between experimental and estimated firing rate. True parameters should be valid for all frequencies, therefore we define the error function as the average error of 30Hz, 50Hz, 100Hz, and 200Hz frequencies. To minimize the error, we employ Bayesian Adaptive Direct Search [7], which is a fast non-derivative optimization algorithm. The optimization algorithm should select parameters such that the output of the model most accurately represents the dynamic changes which occur to the synaptic weights induced by each individual successive stimulus pulse throughout individual stimulation trains.

**Results:** The figure below (Fig. 1) shows the experimental data versus the model output generated by the parameter estimation algorithm. The estimated parameters show a good match for all frequencies, verifying the validity of this approach. Overall, the results suggest that this method can be used for the assessment of STP dynamics of in-vivo human neuronal recordings.

**Fig. 1:**
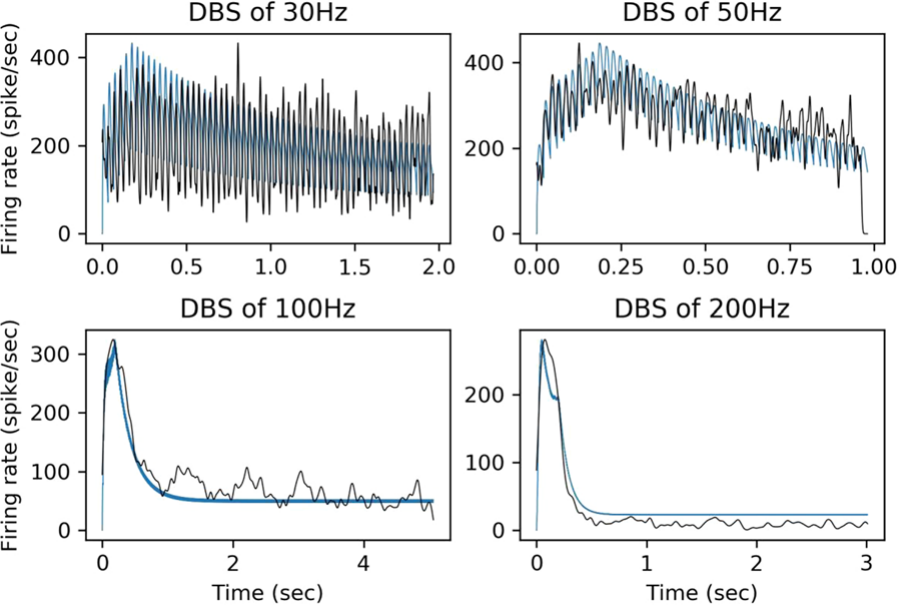
The output of the model with estimated parameters that shows matching between experimental firing rate extraxted during DBS surgery (black), and the model output (blue)

**References**

1. Ghanbari A, Malyshev A, Volgushev M, Stevenson IH. Estimating short-term synaptic plasticity from pre-and postsynaptic spiking. PLoS Computational Biology. 2017; 13(9): e1005738.
2. Costa RP, Sjostrom PJ, Van Rossum MC. Probabilistic inference of short-term synaptic plasticity in neocortical microcircuits. Frontiers in Computational Neuroscience. 2013; 7: 75.
3. Bird AD, Wall MJ, Richardson MJ. Bayesian inference of synaptic quantal parameters from correlated vesicle release. Frontiers in Computational Neuroscience. 2016; 10: 116.
4. Bykowska OS, Gontier C, Sax AL, Jia DW, Llera-Montero M, Bird AD, Houghton CJ, Pfister JP, Costa RP. Model-based inference of synaptic transmission. Frontiers in Synaptic Neuroscience. 2019; 11: 21.
5. Milosevic L, et al. Deciphering Mechanism of DBS-induced Synaptic Plasticity in Different Sub-structures of Basal Ganglia Network BGN. *[in prep]*
6. Tsodyks MV, Markram H. The neural code between neocortical pyramidal neurons depends on neurotransmitter release probability. Proceedings of the National Academy of Sciences. 1997; 94(2): 719-23.
7. Acerbi L, Ji W. Practical Bayesian optimization for model fitting with Bayesian adaptive direct search. In: Advances in neural information processing systems. 2017; 1836-1846.

## P108 Ensemble empirical mode decomposition of noisy local filed potentials from optogenetic data

### Sorinel Oprisan^1^, Xandre Clementsmith^2^, Tamas Tompa^3^, Antonieta Lavin^4^

#### ^1^College of Charleston, Department of Physics and Astronomy, Charleston, South Carolina, United States of America; ^2^College of Charleston, Psychology, Charleston, South Carolina, United States of America; ^3^University of Miskolc, Department of Preventive Medicine, Miskolc, Hungary; ^4^Medical University of South Carolina, Neuroscience, Charleston, South Carolina, United States of America

##### **Correspondence:** Sorinel Oprisan (oprisans@cofc.edu)

*BMC Neuroscience* 2020, **21(Suppl 1)**:P108

Gamma rhythms with frequencies over 30 Hz are thought to reflect cortical information processing. However, signals associated with gamma rhythms are notoriously difficult to record due to their low energy and relatively short duration of the order of a few seconds. In our experiments, of particular interest was the 40 Hz synchronization of neurons, which is believed to be indicative of temporal binding. Temporal binding glues together spatially distributed representations of different features of sensory input, e.g., during the analysis of a visual stimulus, to produce a coherent description of constituent elements.

Our goal was to investigate the effect of systemic cocaine injection on the local field potentials (LFPs) recorded from the medial prefrontal cortex (mPFC) of mice. We used male PV-Cre mice infected with a viral vector [1] that makes some proteins sensitive to light, such as the members of the opsin family, including retinal pigments in visual systems. By genetic engineering, channelrhodopsins was coupled to sodium channels express in neurons and increase their excitability when exposed to blue light [1].

We previously used nonlinear dynamics tools, such as delay embedding and false nearest neighbors [2,3], to estimate the embedding dimension and the delay time for attractor reconstruction from LFPs [4]. While nonlinear dynamics is a powerful tool for data analysis, recent developments suggested that ensemble empirical mode decomposition (EEMD) could be better suited for short and noisy time series. The traditional EMD method is a data-driven decomposition of the original data into orthogonal Intrinsic Mode Functions (IMFs) [5]. In the presence of noise, the time scale separation is not perfect, and IMF mixing produces significant energy leaks between modes. The advantage of EEMD is that by adding a controlled amount of noise to the data leads to a between demixing of the IMFs. We also performed a Hilbert-Huang [5] transform to the demixed IMFs and computed the instantaneous frequency spectrum. Our results indicate that cocaine significantly shifts the energy distribution towards earlier durations during the trial compared to control. Our findings allow us to estimate the contribution of different spectral components quantitatively and develop a dynamical model of the data.

**References**

1. Dilgen JE, Tompa T, Saggu S, Naselaris T, Lavin A. Optogenetically evoked gamma oscillations are disturbed by cocaine administration. Frontiers in Cellular Neuroscience. 2013; 7: 213.
2. Oprisan SA, Lynn PE, Tompa T, Lavin A. Low-dimensional attractor for neural activity from local field potentials in optogenetic mice. Frontiers in Computational Neuroscience. 2015; 9: 125.
3. Oprisan SA, Imperatore J, Helms J, Tompa T, Lavin A. Cocaine-induced changes in low-dimensional attractors of local field potentials in optogenetic mice. Frontiers in Computational Neuroscience. 2018; 12: 1.
4. Oprisan SA, Clementsmith X, Tompa T, Lavin A. Dopamine receptor antagonists effects on low-dimensional attractors of local field potentials in optogenetic mice. PLoS ONE. 2019; 14(10): e0223469.
5. Huang NE, et al. The Empirical Mode Decomposition and the Hilbert Spectrum for Nonlinear and Nonstationary Time Series Analysis. Proceedings of the Royal Society of London A. 1998; 454: 903-995.

## P109 Investigating water transport mechanisms in astrocytes with high dimensional parameter estimation

### Pierre-Louis Gagnon^1^, Kaspar Rothenfusser^2^, Nicolas Doyon^3^, Pierre Marquet^4^, François Laviolette^5^

#### ^1^Université Laval, Département d’Informatique et Génie Logiciel, Quebec, Canada; ^2^Centre hospitalier universitaire vaudois, Département de psychiatrie, Lausanne, Switzerland; ^3^Centre hospitalier universitaire vaudois, Department of Mathematics and Statistics, Quebec, Canada; ^4^Université Laval, Department of Psychiatry and Neuroscience, Quebec, Canada; ^5^Université Laval, Department of Software Engineering and Computer Science, Quebec, Canada

##### **Correspondence:** Pierre-Louis Gagnon (pierre-louis.gagnon.1@ulaval.ca)

*BMC Neuroscience* 2020, **21(Suppl 1)**:P109

Holographic microscopy allows one to measure subtle volume changes of cells submitted to challenges such as an osmotic shock or sudden increase in extracellular potassium. Interpreting volumetric data however remains a challenge. Specifically, relating the amplitude of volume changes to the biophysical properties of cells such as passive permeability to water or the rate of water transport by cation chloride cotransporter is a difficult but important task. Indeed, mechanisms of volume regulation are key for cell resilience and survival. Experimentally, the second author measured the volume response as well as the change in sodium concentration of astrocytes submitted to bath applied: hypo-osmotic solutions, solutions with high potassium concentration or solutions containing glutamate. Overall, he measured the time course of the response of over 2000 astrocytes. In order to interpret this rich data, we developed a mathematical model based on our biophysical knowledge of astrocytes. This model relates on the one hand the experimental perturbations of the extracellular medium and on the other the properties of the cell such as its various conductances or strengths of transporters to its responses in terms of volume change, changes in ionic concentrations and in membrane potential. Determining the biophysical properties of cells thus boils down to a problem of model calibration. This presentation is mainly focused on the work of the first author who designed and implemented a gradient-based optimization algorithm, to estimate model parameters and find the values of the parameters which best explain the data coming from distinct modalities and astrocytes.

A first computational challenge is to combine data from different modalities. In some experiments, the sodium response is measured while in others, the volume response is inferred from phase measurements. We also take advantage of the fact that expert knowledge provides information on variables which are not measured. For example, even if membrane potential is not measured, we impose that it is between -100 mV and -50 mV at equilibrium. Combining these different information sources translate into a complex loss function. Furthermore, using a priori knowledge on the value of parameters, we developed a Bayesian approach. Another challenge comes from the fact that different measurements come from different cells. Our goal is thus not to infer a single set of parameters but rather to infer how biophysical parameters are distributed within the population of cells. This was achieved by using a Tikhonov approach which penalizes parameter values laying far from the average of the distribution.

With our algorithm, we were able to infer the strength of the sodium potassium ATPase pump in each cell with a good precision. This could be useful in identifying cells which are more vulnerable. Parameters related to water transport such the passive membrane permeability to water or the rate of water transport through cation chloride cotransporters are elusive and cannot be determined by conventional methods. Our inference algorithms provided information on these values. Finally, our algorithm is flexible enough to adapt rapidly to take advantage of new experiment type or new data modality.

## P110 Synchronization and resilience in the Kuramoto white matter network model with adaptive state-dependent delays

### Daniel Park^1^, Jeremie Lefebvre^2^

#### ^1^University of Toronto, Department of Mathematics, Toronto, Canada; ^2^University of Ottawa, Department of Biology, Ottawa, Canada

##### **Correspondence:** Daniel Park (seonghyun.park@mail.utoronto.ca)

*BMC Neuroscience* 2020, **21(Suppl 1)**:P110

Myelin sheaths around axonal lengths are formed by mature oligodendrocytes, and play a critical part in regulating signal transmission in the nervous system. Contrary to traditional assumptions, recent experiments have revealed that myelin remodels itself in an activity-dependent way, during both developmental stages and well into adulthood in mammalian subjects. Indeed, it has shown that myelin structure is affected by extrinsic factors such as one’s social environment and intensified learning activity. As a result, axonal conduction delays continuously adjust in order to regulate the timing of neural signals propagating between different brain regions. While there is strong empirical support for such phenomena, the plasticity mechanism has yet to be extensively modeled in neurocomputational fields. As a preliminary step, we incorporate adaptive myelination in the form of state-dependent delays into neural network models, and analyze how it consequently alters its dynamics. In particular, we ask what role myelin plasticity plays in brain synchrony, which is a fundamental element of neurological function. Brain synchrony is simplistically represented in coupled phase-oscillator models such as the Kuramoto network model. As a prototype, we equip the Kuramoto model with a distribution of variable delays governed by a plasticity rule with phase difference gain that allows the delays and oscillatory phases to evolve over time with mutually dependent dynamics. We analyzed the equilibria and stability of this system, and applied our results to large dimensional networks. Our joint mathematical and numerical analysis demonstrates that plastic delays act as a stabilizing mechanism promoting the network’s ability to maintain synchronous activity. At a high-dimensional network level, our work also shows that global synchronization is more resilient to perturbations and injury towards network architecture. Specifically, our conducted numerical experiments imply that plastic delays play a positive role in improving a large-dimensional system’s resilience in achieving synchrony from a sustained injury. Our results provide key insights about the analysis and potential significance of activity-dependent myelination in large-scale brain synchrony.

## P111 The impact of noise on the temporal patterning of neural synchronization

### Leonid Rubchinsky^1^, Joel Zirkle^2^

#### ^1^Indiana University Purdue University Indianapolis and Indiana University School of Medicine, Department of Mathematical Sciences and Stark Neurosciences Research Institute, Indianapolis, United States of America; ^2^Indiana University Purdue University Indianapolis, Department of Mathematical Sciences, Indianapolis, United States of America

##### **Correspondence:** Leonid Rubchinsky (lrubchin@iupui.edu)

*BMC Neuroscience* 2020, **21(Suppl 1)**:P111

Neural synchrony in the brain is often present in an intermittent fashion, i.e. there are intervals of synchronized activity interspersed with intervals of desynchronized activity. A series of experimental studies showed that the temporal patterning of neural synchronization may be very specific, exhibiting predominantly short (although potentially numerous) desynchronized episodes [1], and may be correlated with behavior (even if the average synchrony strength is not changed) [2-4]. Prior computational neuroscience research showed that a network with many short desynchronized intervals may be functionally different than a network with few long desynchronized intervals [5]. In this study, we investigated the effect of noise on the temporal patterns of synchronization. We employed a simple network of two conductance-based neurons that were mutually connected via excitatory synapses. The resulting dynamics of the network was studied using the same time-series analysis methods used in prior experimental and computational studies. It has been well known that synchrony strength degrades with noise. We found that noise also affects the temporal patterning of synchrony. Increase in the noise level promotes dynamics with predominantly short desynchronizations. Thus, noise may be one of the mechanisms contributing to the short desynchronization dynamics observed in multiple experimental studies.

**Acknowledgements:** This work was supported by NSF grant DMS 1813819.

**References**

1. Ahn S, Rubchinsky LL. Short desynchronization episodes prevail in synchronous dynamics of human brain rhythms. Chaos. 2013; 23; 013138.
2. Ahn S, Rubchinsky LL, Lapish CC. Dynamical reorganization of synchronous activity patterns in prefrontal cortex - hippocampus networks during behavioral sensitization. Cerebral Cortex. 2014; 24: 2553-2561.
3. Ahn S, Zauber SE, Worth RM, Witt T, Rubchinsky LL. Neural synchronization: average strength vs. temporal patterning. Clinical Neurophysiology. 2018; 129: 842-844.
4. Malaia E, Ahn S, Rubchinsky LL. Dysregulation of temporal dynamics of synchronous neural activity in adolescents on autism spectrum. Autism Research. 2020; 13: 24-31.
5. Ahn S, Rubchinsky LL. Potential mechanisms and functions of intermittent neural synchronization. Frontiers in Computational Neuroscience. 2017; 11: 44.

## P112 Comparing Drosophila neural circuit models with FlyBrainLab

### Aurel A Lazar, Tingkai Liu, Mehmet K Turkcan, Yiyin Zhou

#### Columbia University, Electrical Engineering, New York, United States of America

##### **Correspondence:** Mehmet K Turkcan (mkt2126@columbia.edu du)

*BMC Neuroscience* 2020, **21(Suppl 1)**:P112

In recent years, a wealth of Drosophila neuroscience data have become available. These include cell type, connectome and synaptome datasets for both the larva and adult fly [1-4]. To facilitate integration across data modalities and to accelerate understanding the functional logic of the fly brain, we developed an interactive computing environment called FlyBrainLab [5].

FlyBrainLab brings together tools enabling the morphological visualization and exploration of large connectomics datasets, interactive circuit construction and visualization, multi-GPU execution of neural circuit models for in silico experimentation, and libraries to aid data analysis. FlyBrainLab provides the flexibility to readily navigate between the in vivo circuits and executable in silico circuits. Moreover, FlyBrainLab methodologically supports the efficient comparison of fly brain circuit models, either across model instances developed by different researchers, or across different developmental stages of the fruit fly. We provide two example comparisons below.

First, we constructed a wild-type biological central complex (CX) circuit followed by a corresponding interactive baseline CX circuit diagram (Fig. 1, left). This enabled us to automatically map three CX circuit models described in the literature into executable circuits (Fig. 1, middle-right). With these circuits on the same platform, we devised the same set of inputs to the CX models and an evaluation criterion. The evaluation of the function of the executable circuits revealed the differences in modeling assumptions and the less visible details underlying the mapping between neuroanatomy, neurocircuitry and computation in the executable circuits. Based on these and other comparisons, new executable CX circuit models can be developed, evaluated and scrutinized by the research community.

**Fig. 1:**
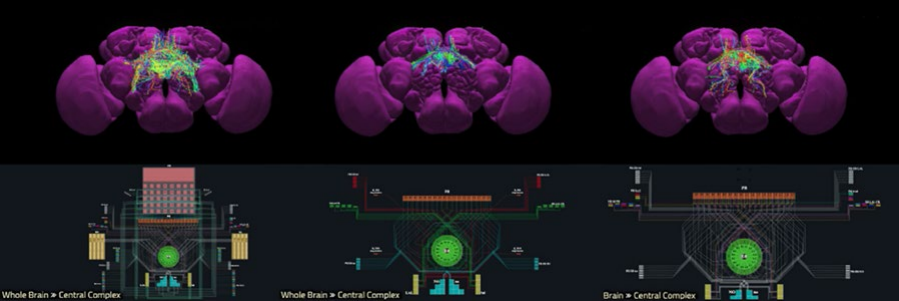
From the wild-type biological CX circuit and its circuit diagram (left column), two additional models of the CX are instantiated and compared (middle and right columns)

Second, by integrating biological data across adult and larval flies we developed executable models for early olfactory systems in both developmental stages. Circuit models are implemented for the Antenna, the Antennal Lobe and the Mushroom Body, and are interactively configurable. We evaluated the I/O characteristics of adult and larva, and discovered significant differences in odorant encoding capabilities. Noticeably, the adult excels in disparately representing differing odorant identities while requiring 10x higher neural hardware and as such much higher energy consumption. Such tradeoff between computation and energy requirement suggests a general principle of adaptation to environmental niches which we are currently exploring.

**Acknowledgments:** The research reported here was supported by AFOSR under grant #FA9550-16-1-0410 and DARPA under contract #HR0011-19-9-0035.

**References**

1. Chiang AS, et al. Three-dimensional reconstruction of brain-wide wiring networks in Drosophila at single-cell resolution. Current Biology. 2011; 21(1): 1-1.
2. Takemura SY, et al. Synaptic circuits and their variations within different columns in the visual system of Drosophila. Proceedings of the National Academy of Sciences. 2015; 112(44): 13711-6.
3. Berck ME, et al. The wiring diagram of a glomerular olfactory system. Elife. 2016; 5: e14859.
4. Xu CS, et al. A connectome of the adult drosophila central brain. BioRxiv. 2020.
5. Turkcan MK, et al. FlyBrainLab: an interactive computing environment for the fruit fly brain. Society of Neuroscience. 2019.

## P113 Dynamically damped stochastic alpha-band relaxation activity in 1/f noise and alpha blocking in resting M/EEG

### Rick Evertz^1^, Damien Hicks^2^, David Liley^3^

#### ^1^Swinburne University of Technology, Melbourne, Australia; ^2^Centre for Human Psychopharmacology, Optical Sciences Centre, Melbourne, Australia; ^3^The University of Melbourne, Department of Medicine, Melbourne, Australia

##### **Correspondence:** Rick Evertz (revertz@swin.edu.au)

*BMC Neuroscience* 2020, **21(Suppl 1)**:P113

Dynamical and physiological basis of alpha band activity and 1/f noise is a subject of continued speculation. Here we conjecture, on the basis of empirical data analysis, that both of these features can be dynamically unified if resting EEG is conceived of being the sum of multiple stochastically perturbed alpha band oscillatory relaxation processes. The modulation of alpha-band and 1/f noise activity by dynamic damping is explored in eyes closed (EC) and eyes open (EO) resting state Magneto/Electroencephalography (M/EEG). We assume that the resting M/EEG being recorded is composed of a superposition of stochastically perturbed alpha-band relaxation processes with a distribution of dampings, the functional form of which is unknown. We perform the inverse problem and take measured M/EEG power spectra and compute the distribution of dampings using Tikhonov regularization methods. The characteristics of the damping distribution are examined across subjects, sensors and recording condition (EC/EO).

We find that there are robust changes in the estimated damping distribution between EC/EO recording conditions across participants. Our findings suggest that alpha-blocking and the 1/f noise structure are both explicable through a singular process of dynamically damped alpha-band activity. The estimated damping distributions are typically found to be bimodal or trimodal (Fig. 1). The number and position of the modes is related to the sharpness of the alpha resonance (amplitude, FWHM) and the slope of the power spectrum. The results suggest that there exists an intimate relationship between resting state alpha activity and 1/f noise with changes in both governed by changes to the damping of the underlying alpha relaxation processes. In particular, alpha-blocking is observed to be the result of the most weakly damped distribution mode (peak at 0.4 - 0.6s^-1) becoming more heavily damped (peak at 1.0 - 1.5s^-1). Reductions in the slope of the 1/f noise are the result of the alpha relaxation processes becoming more broadly distributed in their respective dampings with more weighting towards heavily damped alpha activity. The results suggest a novel way of characterizing resting M/EEG power spectra and provides new insight into the central role that damped alpha-band activity may play in the interesting spatio-temporal features of resting state M/EEG.

**Fig. 1:**
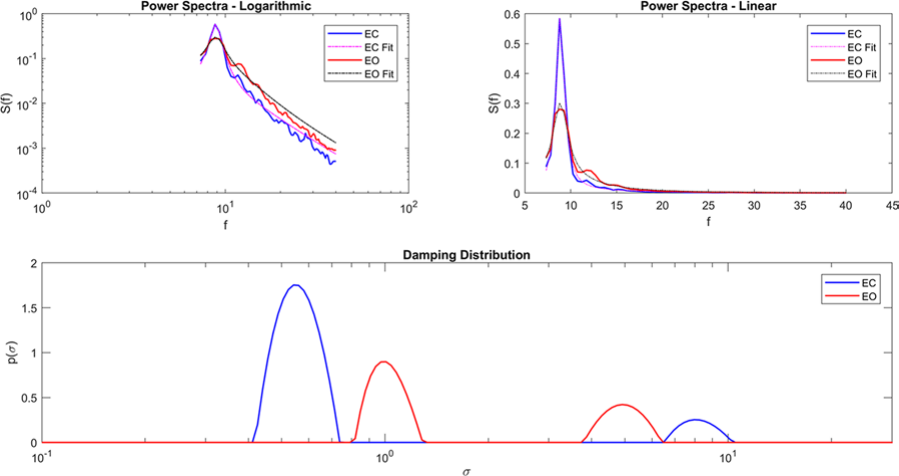
Top panel: Power spectrum for EC and EO (dashed) conditions plotted in logarithmic and linear coordinates. Power spectrum generated using the estimated damping distributions in the forward problem are shown for EC and EO (solid) alongside the measured power spectra. Bottom panel: Typical damping distribution results found in EC and EO for subjects who demonstrate alpha blocking

Future work will explore the more complex case where we expect a distribution over both frequency and damping for the stochastic relaxation processes, elucidating any frequency dependent damping effects between conditions. The inverse problem can be solved via gradient descent methods where we estimate the 2-dimensional probability density function over frequency and damping from a given power spectrum.

## P114 Effect of diverse recoding of granule cells on delay eyeblink conditioning in a cerebellar network

### Sang-Yoon Kim, Woochang Lim

#### Daegu National University of Education, Institute for Computational Neuroscience and Department of Science Education, Daegu, South Korea

##### **Correspondence:** Woochang Lim (wclim@icn.re.kr)

*BMC Neuroscience* 2020, **21(Suppl 1)**:P114

We consider a ring network for the delay eyeblink conditioning, and investigate the effect of diverse firing activities of granule (GR) cells on the eyeblink conditioning under conditioned stimulus (tone) by varying the connection probability *p*c from Golgi to GR cells. For an optimal value of p*c, individual GR cells exhibit diverse spiking patterns which are well- or poor-matched with the unconditioned stimulus (airpuff). Then, these diversely-recoded signals via parallel-fibers (PFs) from GR cells are effectively depressed by the error teaching signals via climbing fibers (CFs) from the inferior olive. Synaptic weights at well-matched PF–Purkinje cell (PC) synapses of active GR cells are strongly depressed via strong long-term depression (LTD), while no LTD occurs at poor-matched PF–PC synapses. This kind of “effective” depression at PF-PC synapses coordinates firings of PCs effectively, which then exert effective inhibitory coordination on cerebellar nucleus (CN) (which evokes conditioned response (CR; eyeblink)). When the learning trial passes a threshold, CR occurs. In this case, the timing degree *T*d becomes good due to presence of poor-matched spiking group which plays a role of protection barrier for the timing. With further increase in trials, strength of CR *S*CR increases due to strong LTD in the well-matched spiking group, while its timing degree decreases. Thus, the overall efficiency degree *L*e (taking into consideration both timing and strength of CR) for the eyeblink increases with trials, and eventually saturates. By changing *p*c, we also investigate the delay eyeblink conditioning and find that a plot of *L*e versus *p*c forms a bell-shaped curve with a peak at p*c (where the diversity degree *D* in firing of GR cells is also maximum). The more diverse in spiking patterns of GR cells, the more effective in CR for the eyeblink.

**Fig. 1:**
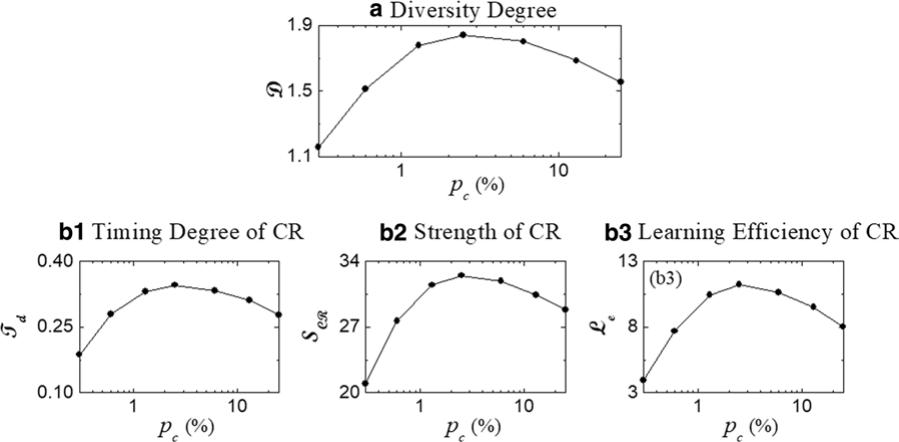
**a** Diversity degree D versus the connection probability p_c_ from Golgi cells to granule cells. **b1** Timing degree T_d_ versus p_c_, **b2** strength of conditional response S_CR_ versus p_c_, and **b3** overall efficiency degree L_e_ versus p_c_

## P115 Approximating information filtering of a two-stage neural system

### Gregory Knoll, Žiga Bostner, Benjamin Lindner

#### Humboldt-Universitaet zu Berlin, Physics, Berlin, Germany

##### **Correspondence:** Gregory Knoll (gregory.knoll@bccn-berlin.de)

*BMC Neuroscience* 2020, **21(Suppl 1)**:P115

Information streams are processed in the brain by populations of neurons tuned to perform specific computations, the results of which are forwarded to subsequent processing stages. Building on theoretical results for the behavior of single neurons and populations, we investigate the extent to which a postsynaptic cell (PSC) can detect the information present in the output stream of a population which has encoded a signal. In this two-stage system, illustrated in Figure 1A, the population is a simple feedforward network of integrate-and-fire neurons which integrate and relay the signal, reminiscent of auditory or electroreceptor afferents in the sensory periphery. Depending on the application, the information relevant for the PSC may be contained in a specific frequency band of the stimulus, requiring the PSC to properly tune its information encoding to that band (information filtering). In the specific setup studied here, information filtering is associated with detecting synchronous activity. It was found that synchronous activity of a neural population selectively encodes information about high-frequency bands of a broadband stimulus, and it was hypothesized that this information can be read out by coincidence detector cells that are activated only by synchronous input. Firstly, we test this hypothesis and match the key characteristics of information filtering, the spectral coherence function, of the PSC and the stimulus and of the time-dependent synchrony in the population output and the stimulus (Fig. 1B, left); we show that the relations between the synchrony and PSC thresholds and between the synchrony window and PSC time constant are roughly linear (Fig. 1B, right), which implies that the synchronous output of the population can be taken as a proxy for the postsynaptic coincidence detector and, conversely, that the PSC can be made to detect synchrony (or coincidence) by adjusting its time constant and threshold. Secondly, we develop an analytical approximation for the coherence function of the PSC and the stimulus and demonstrate its accuracy by comparison against numerical simulations (Fig. 1C), both in the fluctuation-dominated and mean-driven regimes of the PSC.

**Fig. 1:**
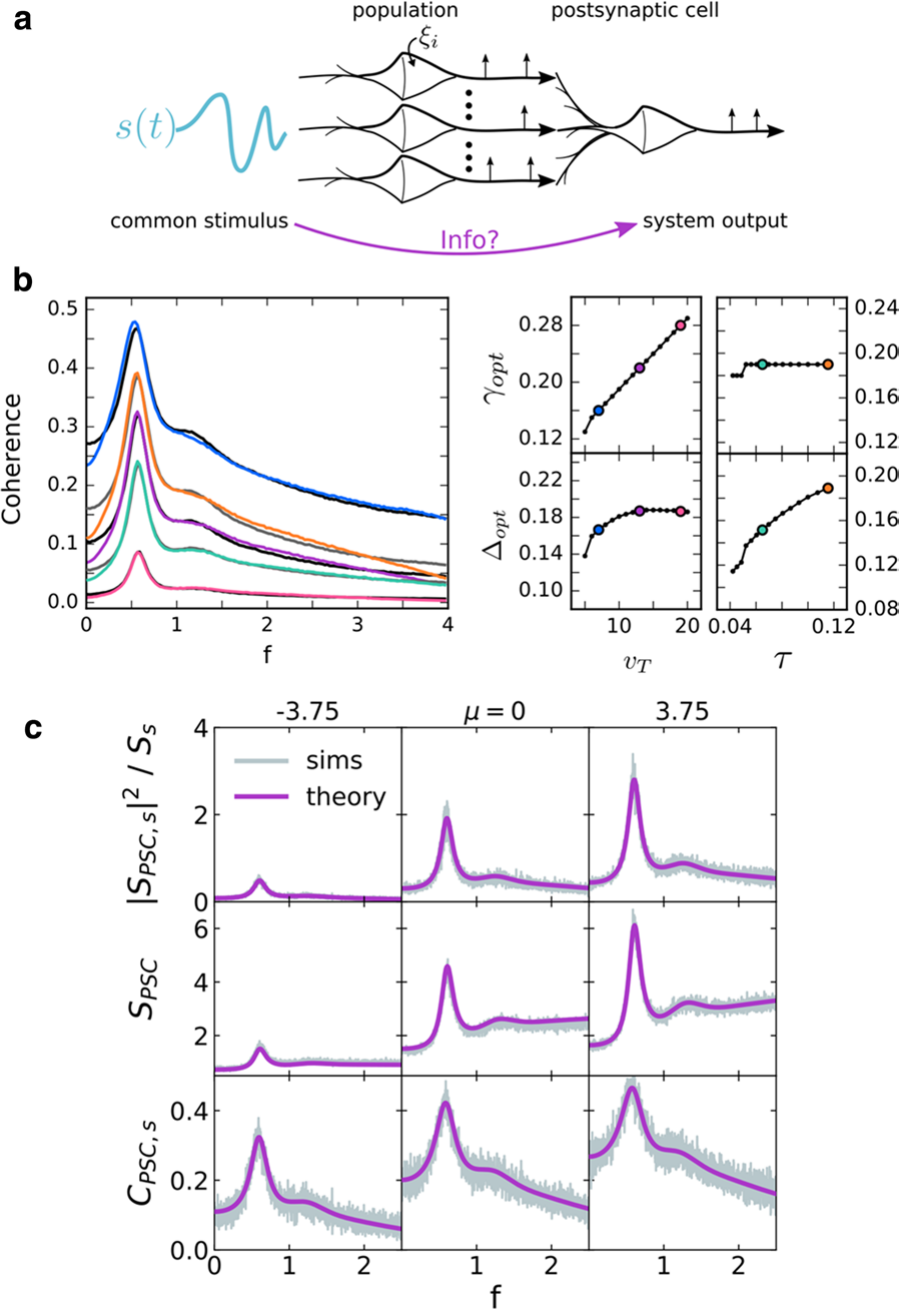
**A** System diagram of the two-stage neural system. **B** Matching coherence functions to relate synchronous output criteria to PSC parameters. **C** Band-pass filtering of information from a broadband stimulus by the two-stage neural system

## P116 Astrocyte simulations with realistic morphologies reveal diverse calcium transients in soma and processes

### Ippa Seppala^1^, Laura Keto^2^, Iiro Ahokainen^1^, Nanna Förster^1^, Tiina Manninen^2^, Marja-Leena Linne^1^

#### ^1^Tampere University, Medicine and Health Sciences, Tampere, Finland; ^2^Tampere University, Faculty of Medicine and Health Technology, Tampere, Finland

##### **Correspondence:** Ippa Seppala (ippa.seppala@tuni.fi)

*BMC Neuroscience* 2020, **21(Suppl 1)**:P116

The role of astroglia has long been overlooked in the field of computational neuroscience. Lately their involvement in multiple higher-level brain functions, including neurotransmission, plasticity, memory, and neurological disorders, has been found to be more significant than previously thought. It has been hypothesised that astrocytes fundamentally affect the information processing power of the mammalian brain. As the glia to neuron ratio increases when moving from simpler organisms to those more complex, it is clear that more attention should be directed to glial involvement. Despite the recent advances in neuroglial research there still exists a lack of glia-specific computational tools. Astroglia differ considerably from neurons in their morphology as well as their biophysical functions [1], making it difficult to acquire reliable simulation results with simulators made for studying neuronal behaviour. As the differences in cellular dynamics of astrocytes compared to those of neurons are significant, there clearly exists a need for tailored methods for simulating the behaviour of glial cells.

One such astrocyte specific simulator has been developed [2]. In simulations ASTRO uses MATLAB and NEURON environments [3] and is capable of representing various biologically relevant astroglial mechanisms such as calcium waves and diffusion. In this work we used ASTRO to simulate several astrocytic functions with the help of existing in vivo morphologies from various brain areas. We concentrated on calcium transients, as calcium-mediated signaling is thought to be the main mechanism of intra- and intercellular messaging between astroglia and other neural cell types. The time-scales of these calcium-mediated events have recently been shown to differ considerably in different spatial locations of astrocytes. We were able to reproduce these results in silico by simulating a morphologically detailed computational model that we developed based on previous work [4,5]. This was partly due to ASTRO’s capability to analyse the microscopic calcium dynamics in fine processes, branches and leaves.

With our model ASTRO proved to be a promising tool in simulating astrocytic functions and could potentially offer novel insights to glia-neuron interactions also in future work.

**Acknowledgements:** The work was supported by Academy of Finland through grants (297893, 326494, 326495) and the European Union’s Horizon 2020 Framework Programme for Research and Innovation under the Specific Grant Agreement No. 785907 (Human Brain Project SGA2).

**References**

1. Calì C, et al. 3D cellular reconstruction of cortical glia and parenchymal morphometric analysis from Serial Block-Face Electron Microscopy of juvenile rat. Progress in Neurobiology. 2019;183: 101696.
2. Savtchenko LP, et al. Disentangling astroglial physiology with a realistic cell model in silico. Nature Communications. 2018; 9(1): 1-15.
3. Carnevale T. Neuron simulation environment. Scholarpedia. 2007; 2(6): 1378.
4. Manninen T, Havela R, Linne M-L. Reproducibility and comparability of computational models for astrocyte calcium excitability. Frontiers in Neuroinformatics. 2017; 11: 11.
5. Manninen T, Havela R, Linne M-L. Computational models for calcium-mediated astrocyte functions. Frontiers in Computational Neuroscience. 2018; 12: 14.

## P117 The interplay of neural mechanisms regulates spontaneous cortical network activity: inferring the role of key mechanisms using data-driven modeling

### Jugoslava Acimovic, Tiina Manninen, Heidi Teppola, Marja-Leena Linne

#### Tampere University, Faculty of Medicine and Health Technology, Tampere, Finland

##### **Correspondence:** Jugoslava Acimovic (jugoslava.acimovic@tuni.fi)

*BMC Neuroscience* 2020, **21(Suppl 1)**:P117

In isolated neural systems devoid of external stimuli, the exchange between neuronal, synaptic and putatively also glial mechanisms gives rise to spontaneous self-sustained synchronous activity. This phenomenon has been extensively documented in dissociated cortical cultures in vitro that are routinely used to study neural mechanisms in health and disease. We examine these mechanisms using a new data-driven computational modeling approach. The approach integrates standard spiking network models, non-standard glial mechanisms and network-level experimental data.

The experimental data represents spontaneous activity in dissociated rat cortical cultures recorded using microelectrode arrays. The recordings were performed under several experimental protocols that involved pharmacological manipulation of network activity. Under each protocol the activity exhibited characteristic network bursts, the short intervals (100ms to 1s) of intensive network-wide spiking interleaved by longer (~10s) periods of sparse uncorrelated spikes. The data was analysed to extract, among other properties, duration, intensity and frequency of burst events [1].

The computational model incorporates fast burst propagation and decay mechanisms, as well as the slower burst initiation mechanisms. We first constructed the fast part of the model as a generic spiking neuronal network and optimized it to the experimental data describing intra-burst properties. We developed a model fitting routine relying on multi-objective optimization [2]. The optimized ‘fast’ model was then extended with a selected astrocytic mechanism operating on a similar time-scale as the network burst initiation [3]. Typically, the burst initiation is attributed to a combination of noisy inputs and the dynamics of neuronal (and synaptic) adaptation currents. While noise provides necessary depolarization of cell membrane the adaptation currents prohibit fast initiation of the next burst event. The noise might account for the randomness in ion channel opening and closing, the spontaneous synaptic release and other sources of randomness. The adaptation accounts for the kinetics of various ion channels. We explore the role of a non-standard deterministic mechanism introduced through slow inward current from astrocytes to neurons.

We demonstrate that the fast neuronal part of the model successfully reproduces intra-burst dynamics, including the duration and intensity of network bursts. The model is flexible enough to account for several experimental conditions. Coupled to the slower astrocyte-neuron interaction mechanism the system becomes capable of generating bursts with the frequency proportional to the one seen in vitro.

**Acknowledgements:** This research has received funding from the European Union’s Horizon 2020 Framework Programme for Research and Innovation under the Specific Grant Agreement No. 785907 (Human Brain Project SGA2). The funding has also been received from the Academy of Finland through grants No. 297893, 326494, 326495.

**References**

1. Teppola H, Aćimović J, Linne ML. Unique features of network bursts emerge from the complex interplay of excitatory and inhibitory receptors in rat neocortical networks. Frontiers in Cellular Neuroscience. 2019; 13: 377.
2. Aćimović J, Teppola H, Mäki-Marttunen T, Linne M-L. Data-driven study of synchronous population activity in generic spiking neuronal networks: How much do we capture using the minimal model for the considered phenomena? *BMC Neuroscience* 2018;19(2): 136.
3. Aćimović J, et al. Modeling the influence of neuron-astrocyte interactions on signal transmission in neuronal networks. *BMC Neuroscience* 2019; 20(1): 178.

## P118 Compartmental models for mammalian cortical pyramidal neurons: a survey of published models, model complexity and parameter sensitivity

### Emma Huhtala^1^, Jugoslava Acimovic^2^, Mikko Lehtimäki^2^, Marja-Leena Linne^3^

#### ^1^Tampere University, Tampere, Finland; ^2^Tampere University, Faculty of Medicine and Health Technology, Tampere, Finland; ^3^Tampere University, Medicine and Health Sciences, Tampere, Finland

##### **Correspondence:** Emma Huhtala (emma.huhtala@tuni.fi)

*BMC Neuroscience* 2020, **21(Suppl 1)**:P118

Pyramidal neurons are abundant in the neocortex and known to contribute to diverse and complex cognitive functions. To better understand the role of individual pyramidal cell types we need neuron models that can be efficiently and reliably simulated and analyzed when embedded into circuit-level models yet that maintain the necessary structural and functional complexity. In this study we contribute to this goal the following ways: 1) We review and compare over 50 published mammalian cortical pyramidal neuron models available in the literature and public repositories (with the focus on models implemented in the NEURON simulation environment). 2) We test two recently published tools that tackle critical issues for detailed neuron modeling, the role of model complexity and size and the sensitivity to numerous and occasionally unreliable model parameters.

In goal 1, we compared the models based on the following criteria: the brain area and layer, number of compartments, biophysical properties, software used to simulate the model, and the amount of experimental data used to construct and fine-tune the model. Based on this work, we chose a layer 2/3 pyramidal cell model from the rat somatosensory cortex, acquired from the Blue Brain Project data portal [1], as our test case. We used two recently published toolboxes, Neuron_Reduce and Uncertainpy, to analyze the model. Neuron_Reduce [2] is designed for simplifying the morphology of the model while replicating the model dynamics and accelerating the simulations considerably. Uncertainpy [3] allows the user to examine the impact of uncertainty and sensitivity of the model parameters.

The analyses carried out in this work show that Neuron_Reduce toolbox is an effective tool for simplifying the morphological structure of neuron models. With moderate reduction of the model (preserving 10-25% of the original model compartments) the Neuron_Reduce toolbox simplifies the dendritic structure of the cell while replicating the behavior of the original model. However, a dramatic reduction (preserving 3% of the original model compartments) led to changes in the shape of action potentials. The results obtained with Uncertainpy suggest that the reduced model shows sensitivity to only a subset of model parameters among those that affect the original model. For example, while the original model depends on several somatic conductances, the reduced model is sensitive mainly to sodium and potassium channel conductances. Based on our testing, both toolboxes will be useful tools for analyzing models in neuroscience. In addition, they can help the re-use of compartmental models in new modeling initiatives, particularly when modeling multiple spatiotemporal scales of the brain phenomena.

**Acknowledgments:** This work was supported by the Academy of Finland (decision Nos 297893 and 318879).

**References**

1. Blue Brain Portal – Digitally reconstructing and simulating the brain [Online]. Available: https://portal.bluebrain.epfl.ch/. [Accessed: 12-May-2020]
2. Amsalem O, et al. An efficient analytical reduction of detailed nonlinear neuron models. Nature Communications. 2020; 11(1): 1-3.
3. Tennøe S, Halnes G, Einevoll GT. Uncertainpy: A Python toolbox for uncertainty quantification and sensitivity analysis in computational neuroscience. Frontiers in Neuroinformatics. 2018; 12: 49.

## P119 Relating transfer entropy to network structure and motifs, and implications for brain network inference

### Leonardo Novelli, Joseph Lizier

#### The University of Sydney, Centre for Complex Systems, Sydney, Australia

##### **Correspondence:** Leonardo Novelli (lnov6504@uni.sydney.edu.au)

*BMC Neuroscience* 2020, **21(Suppl 1)**:P119

Transfer entropy is an established method for the analysis of directed relationships in neuroimaging data. In its original formulation, transfer entropy is a bivariate measure, i.e., a measure between a pair of elements or nodes [1]. However, when two nodes are embedded in a network, the strength of their direct coupling is not sufficient to fully characterize the transfer entropy between them. This is because transfer entropy results from network effects due to interactions between all the nodes.

In this theoretical work, we study the bivariate transfer entropy as a function of network structure, when the link weights are known. In particular, we use a discrete-time linear Gaussian model to investigate the contribution of small motifs, i.e., small subnetwork configurations comprising two to four nodes. Although the linear model is simplistic, it is widely used and has the advantage of being analytically tractable. Moreover, using this model means that our results extend to Granger causality, which is equivalent to transfer entropy for Gaussian variables.

We show analytically that the dependence of transfer entropy on the direct link weight is only a first approximation, valid for weak coupling. More generally, the transfer entropy increases with the in-degree of the source and decreases with the in-degree of the target, which suggests an asymmetry of information transfer between hubs and peripheral nodes.

Importantly, these results also have implications for directed functional network inference from time series, which is one of the main applications of transfer entropy in neuroscience. The asymmetry of information transfer suggests that links from hubs to peripheral nodes would generally be easier to infer than links between hubs, as well as links from peripheral nodes to hubs. This could bias the estimation of network properties such as the degree distribution and the rich-club coefficient.

In addition to the dependence on the in-degree, the transfer entropy is directly proportional to the weighted motifs involving common parents or multiple walks from the source to the target (Fig. 1). These motifs are more abundant in clustered or modular networks than in random networks, suggesting a higher transfer in the former case. Further, if the network has only positive edge weights, we have a positive correlation to the number of such motifs. This applies in the mammalian cortex (on average, since the majority of connections are thought to be excitatory) – implying that directed functional network inference with transfer entropy is better able to infer links within brain modules (where such motifs enhance transfer entropy values) in comparison to links across modules.

**Fig. 1:**
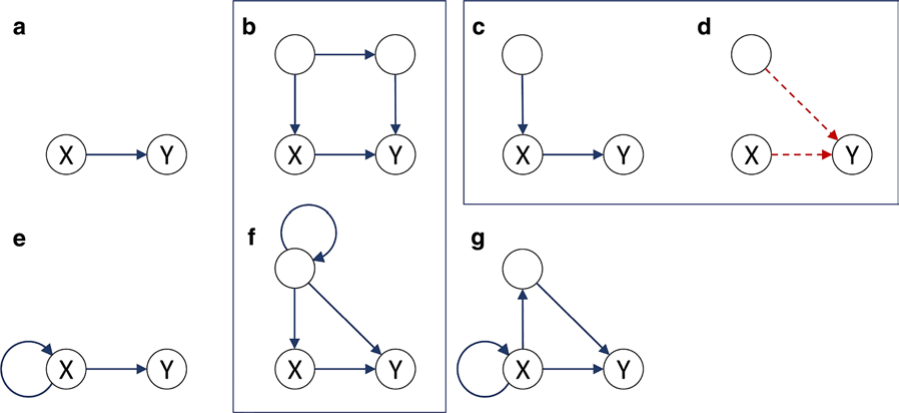
Network motifs involved in the bivariate transfer entropy from node X to node Y

**Reference**

1. Schreiber T. Measuring information transfer. Physical review letters. 2000; 85(2): 461.

## P120 Ageing-related changes in prosocial reinforcement learning and links to psychopathic traits

### Jo Cutler^1^, Marco Wittmann^1^, Ayat Abdurahman^2^, Luca Hargitai^1^, Daniel Drew^1^, Masud Husain^1^, Patricia Lockwood^1^

#### ^1^University of Oxford, Psychology, Oxford, United Kingdom; ^2^University of Cambridge, Psychology Department, Cambridge, United Kingdom

##### **Correspondence:** Jo Cutler (jo.cutler@psy.ox.ac.uk)

*BMC Neuroscience* 2020, **21(Suppl 1)**:P120

Prosocial behaviours, actions that help others, are vital for maintaining social bonds and are linked with improved health. However, our ability to learn which of our actions help others could change as we get older, as existing studies suggest declines in reinforcement learning across the lifespan [1]. This decline in associative learning could be explained by the significant age-related decrease in dopamine transmission [2] which has been suggested to code prediction errors [3]. Alternatively, prosocial learning might not only rely on learning abilities but also on the motivation to help others. This motivation, which is reduced in disorders such as psychopathy, might also shift with age, with a trend for lower levels of antisocial behaviour in older adults [4]. Interestingly, the decrease in dopamine levels in older adults could also support this hypothesis of increased prosociality, as higher dopamine has been linked to lower altruism [5].

Here, using computational modelling of a probabilistic reinforcement learning task (Fig. 1), we tested whether younger (age 18-36) and older (age 60-80, total n=152) adults can learn to gain rewards for themselves, another person (prosocial), or neither individual (control). We replicated existing work showing younger adults were faster to learn when their actions benefitted themselves, compared to when they helped others [6]. Strikingly however, older adults showed a reduced self-bias, compared to younger adults, with learning rates that did not significantly differ between self and other. In other words, older adults showed a relative increase in the willingness to learn about actions that helped others. Moreover, we find that these differences in prosocial learning could emerge from more basic changes in personality characteristics over the lifespan. In older adults, psychopathic traits were significantly reduced and correlated with the difference between prosocial and self learning rates. Importantly, the difference between self and other learning rate was most reduced in older people with the lowest psychopathic traits. Overall, we show that older adults are less self-biased than younger adults, and this change is associated with a decline in psychopathic traits. These findings highlight the importance of examining individual differences across development and have important implications for theoretical and neurobiological accounts of healthy ageing.

**Fig. 1:**
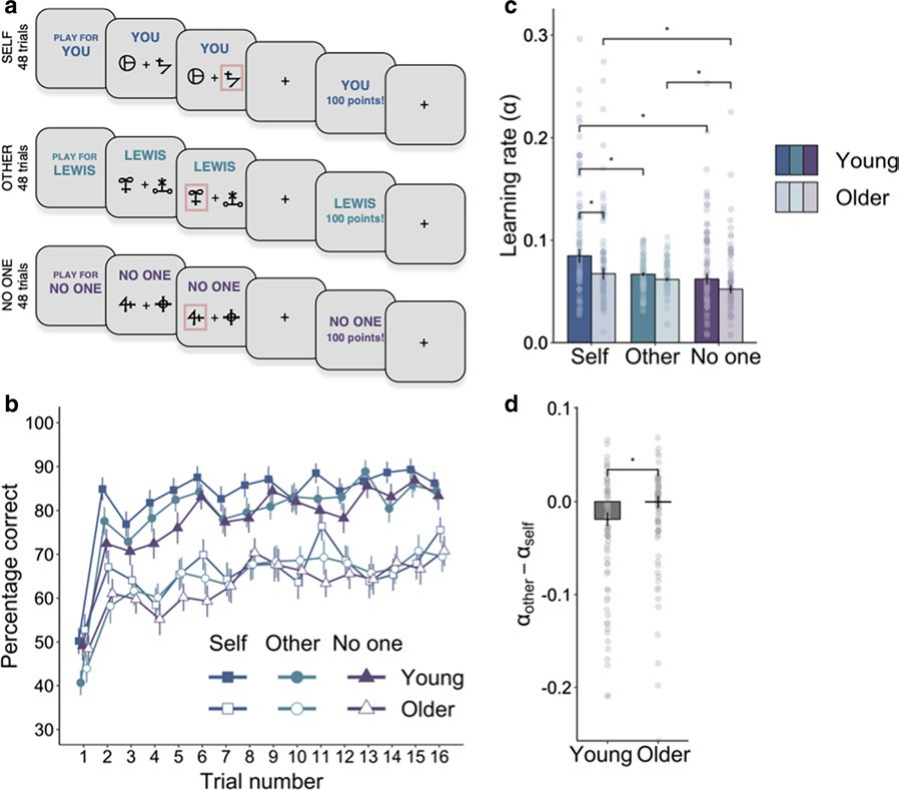
Behavioural task and data. **A** Reinforcement learning task: participants played for either themselves, the other participant, or no one. **B** Group-level learning curves showing choice behaviour in the three learning conditions for each age group. **C** Comparison of learning rates from the computational model. **D** Median difference between learning rates in the other and self conditions. Asterisks represent significant differences (p<.05)

**References**

1. Mell T, Heekeren HR, Marschner A, Wartenburger I, Villringer A, Reischies FM. Effect of aging on stimulus-reward association learning. Neuropsychologia. 2005; 43(4): 554-563.
2. Li S-C, Lindenberger U, Bäckman L. Dopaminergic modulation of cognition across the life span. Neuroscience and Biobehavioral Reviews. 2010; 34(5): 625-630.
3. Schultz W. Dopamine reward prediction error coding. Dialogues in Clinical Neuroscience. 2016; 18(1): 23-32.
4. Gill DJ, Crino RD. The Relationship between Psychopathy and Age in a Non-Clinical Community Convenience Sample. Psychiatry, Psychology and Law. 2012; 19(4): 547-557.
5. Crockett MJ, et al. Dissociable effects of serotonin and dopamine on the valuation of harm in moral decision making. Current Biology. 2015; 25(14): 1852-9.
6. Lockwood PL, Apps MAJ, Valton V, Viding E, Roiser JP. Neurocomputational mechanisms of prosocial learning and links to empathy. Proceedings of the National Academy of Sciences. 2016; 113(35): 9763-9768.

## P121 Avalanches and emergent activity patterns in simulated mouse primary motor cortex

### Donald Doherty, Salvador Dura-Burnal, William W Lytton

#### SUNY Downstate Medical Center, Department of Physiology and Pharmacology, Brooklyn, New York, United States of America

##### **Correspondence:** Donald Doherty (donald.doherty@actionpotential.com)

*BMC Neuroscience* 2020, **21(Suppl 1)**:P121

Avalanches display non-Poisson distributions of correlated neuronal activity that may play a significant role in signal processing. The phenomenon appears robust but mechanisms remain unknown due to an inability to gather large sample sizes, and difficulties in identifying neuron morphology, biophysics, and connections underlying avalanche dynamics. We set out to understand the relationship between power-law activity patterns, their values, and the neural responses observed from every neuron across different layers, cell populations, and the entire cortical column using a detailed M1 model with 15 neuron types that simulated the full-depth of a 300μm diameter column with 10,073 neurons and ~18e6 connections. Self-organized and self-sustained activity from our simulations have power-law values of -1.51 for avalanche size and -1.98 for duration distributions, which are in the range noted in both in vitro and in vivo neural avalanche preparations reported by Beggs and Plentz (2003). We applied a 0.57 nA, 100ms stimulus across 40μm in diameter and full column depth at each of 49 gridded locations (40μm) across the pia surface of our 400μm diameter cylindrical cortical column. Stimuli applied to 4 locations (8.2%) produced no sustained responses. Self-sustained activity was seen in the other 45 locations, which always included activity in IT5B or IT5B and IT6. In 6 locations activity was restricted to IT5B or IT5B/IT6 alone (avalanche size: ~ -2.8). Intermittent spread of activity from IT5B/IT6 across other neuron types and layers was seen in 24 locations (avalanche size: ~ -2.0). In 15 locations, frequent spread of activity to other neuron types and layers was observed (avalanche size: ~ -1.5). Avalanches were defined using binned spiking activity (1ms bins). Each avalanche was composed of adjacent bins filled with one or more action potentials, preceded and followed by at least one empty bin. A prolonged 10-minute M1 simulation with different connectivity produced 15,579 avalanches during sustained activity after the initial 100ms stimulation. Again, IT5B/IT6 activity was constant and punctuated by more widespread activity. Three distinct patterns of activity spontaneously recurred and could be characterized by delta, beta, or gamma frequency dominance. All large-scale avalanches were composed of 1 or a combination of these 3 recognizable patterns. Between the large-scale avalanches we saw three patterns of activity: 1) continuous IT5B and IT6 neuron activity, 2) vigorous layer 5 and IT6 activity, or 3) vigorous layer 5 and IT6 activity that transitioned to continuous IT5B and IT6 activity. Since cortical column activity with just IT5B and IT6 activity showed little correlation (very steep and narrow distributions of avalanche sizes and durations), we hypothesize that the addition of avalanches with layer 5 and IT6 activity (activity patterns 2 and 3 above) result in more correlated activity and power-law values closer to -1.51 and -1.98 for size and duration respectively. In conclusion, the increase in correlated activity among neuronal components parallels the emergence of clearly identifiable activity patterns across time and cortical layers and may generate rhythmic activity.

**Fig. 1:**
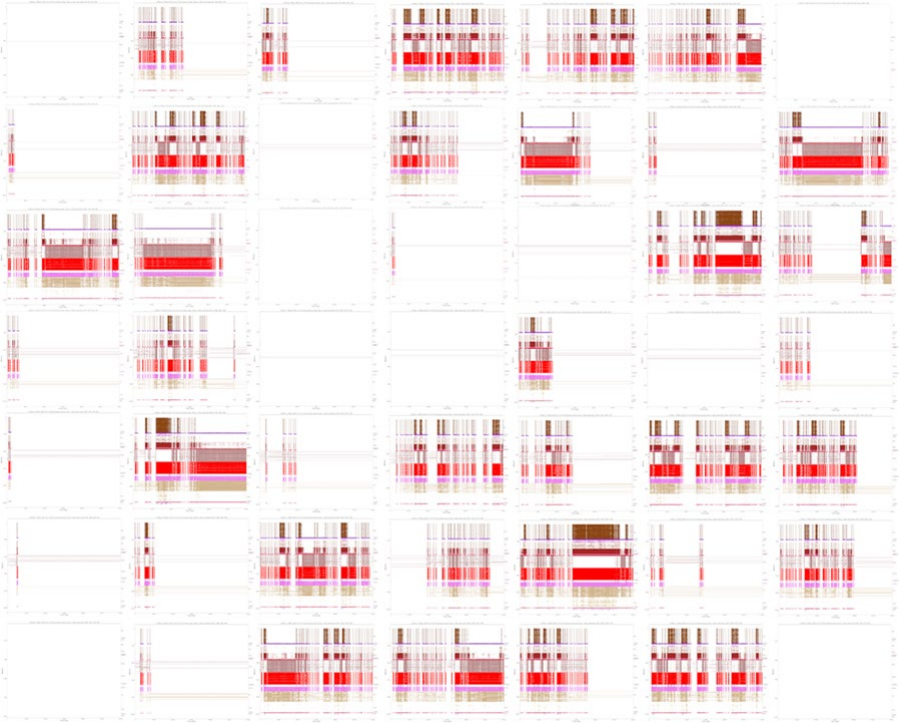
Forty-nine raster plots each showing 60 seconds of activity across all 10,073 neurons after a 100 millisecond 0.57 nano-amperes current was applied to a volume of the cortical column 40 um in diameter and spanning the full 1,350 um depth from pia to white matter

## P122 Adaptive activity-dependent myelination promotes synchronization in large-scale brain networks

### John Griffiths^1^, Jeremie Lefebvre^2^, Rabiya Noori^3^, Daniel Park^4^, Sonya Bells^5^, Paul Frankland^5^, Donald Mabbott^5^

#### ^1^Centre for Addiction and Mental Health, Krembil Centre for Neuroinformatics, Toronto, Canada; ^2^University of Ottawa, Department of Biology, Ottawa, Canada; ^3^Krembil Research Institute, Toronto, Canada; ^4^University of Toronto, Toronto, Canada; ^5^The Hospital for Sick Children, Neurosciences and Mental Health, Toronto, Canada

##### **Correspondence:** Jeremie Lefebvre (jeremie.lefebvre@uottawa.ca)

*BMC Neuroscience* 2020, **21(Suppl 1)**:P122

Communication and oscillatory synchrony between distributed neural populations is believed to play a key role in multiple cognitive and neural functions. These interactions are mediated by long-range myelinated axonal fibre bundles, collectively termed as white matter. While traditionally considered to be static after development, white matter properties have been shown to change in an activity-dependent way through learning and behavior: a phenomenon known as white matter plasticity. In the central nervous system this plasticity stems from oligodendroglia, which form myelin sheaths to regulate the conduction of nerve impulses across the brain, hence critically impacting neural communication. We here shift the focus from neural to glial contribution to brain synchronization and examine the impact of adaptive, activity-dependent change in conduction velocity on the large-scale phase-synchronization of neural oscillators.

We used a network model built of reciprocally coupled Kuramoto phase oscillators whose connections are based on available primate large-scale white matter neuroanatomy data. Our computational and mathematical results show that such adaptive plasticity endows white matter networks with self-regulatory and self-organizing properties, where conduction delay statistics are autonomously adjusted to ensure efficient neural communication. Specifically, our analysis shows that adaptive conduction velocities along axonal connections stabilizes oscillatory neural activity across a wide range of connectivity gain and frequency bands. Resulting conduction delays become statistically similar, promoting phase-locking irrespective of the distances. As a corollary, global phase-locked states are more resilient to diffuse decreases in connectivity, reflecting damage caused by a neurological disease, for instance. Our work suggests that adaptive myelination may be a mechanism that enable brain networks with a means of temporal self-organization, resilience and homeostasis.

## P123 Computational implementation in NEURON of inter-cellular calcium waves in syncytial tissue

### Nilapratim Sengupta, Rohit Manchanda

#### Indian Institute of Technology Bombay, Department of Biosciences and Bioengineering, Mumbai, India

##### **Correspondence:** Nilapratim Sengupta (nilapratim@iitb.ac.in)

*BMC Neuroscience* 2020, **21(Suppl 1)**:P123

Calcium plays highly critical roles in various physiological processes such as muscle contraction, neurotransmission, cell growth and proliferation. Intracellular calcium handling mechanisms have been studied extensively. However, concepts of intracellular calcium dynamics must be complemented with the knowledge of calcium spread in tissues where neighbouring cells are coupled to each other forming a syncytium. Intercellular calcium waves (ICW) are ‘complex spatiotemporal events’ essentially comprising of an elevated level of intracellular calcium that appears to spread from the initiating/stimulated cell to the coupled neighbours [1]. Gap junction mediated diffusion has been identified as a crucial mechanism for ICW in syncytial tissues [2].

The complex structure of syncytial tissue, coupled with different signalling molecules and their varied mechanisms of involvement makes it difficult to study ICW from a quantitative point of view using in vitro or in vivo experiments. Though mathematical models describing ICW propagation in two-dimensions exist, there is no report of a biophysical model to account for three dimensional propagation of ICW in vivo in syncytial tissues. A key objective of our work was to realize, using the NEURON platform, a model for 3-D propagation. Several computational labs (primarily working on neural networks) make use of this platform to build models. However to our knowledge, in none of the existing cellular network models has chemical coupling of cells been incorporated. Successful implementation of our developed technique would produce a biophysically detailed model incorporating structural, electrical as well as chemical aspects that could be used to upgrade all existing models once suitable changes in parameters are made.

Gap junctions, in the NEURON platform, have usually been modelled as low resistance electrical shunts between connected cells [3]. In a novel approach we modelled the gap junction such as to enable it to transfer calcium between connected cells, based on the ion’s electro-chemical gradient. We have equipped the detrusor smooth muscle cell model with intracellular calcium handling mechanisms and then modified the gap junctional connection to incorporate intercellular calcium flow, besides non-specific current. We have successfully simulated calcium spread from the source cell to its adjoining neighbours and beyond. Within the network, as the distance from the source cell increases, extent of calcium flow diminishes as it propagates due to diffusion, buffering and its being pumped out of the cells (Fig. 1).

**Fig. 1:**
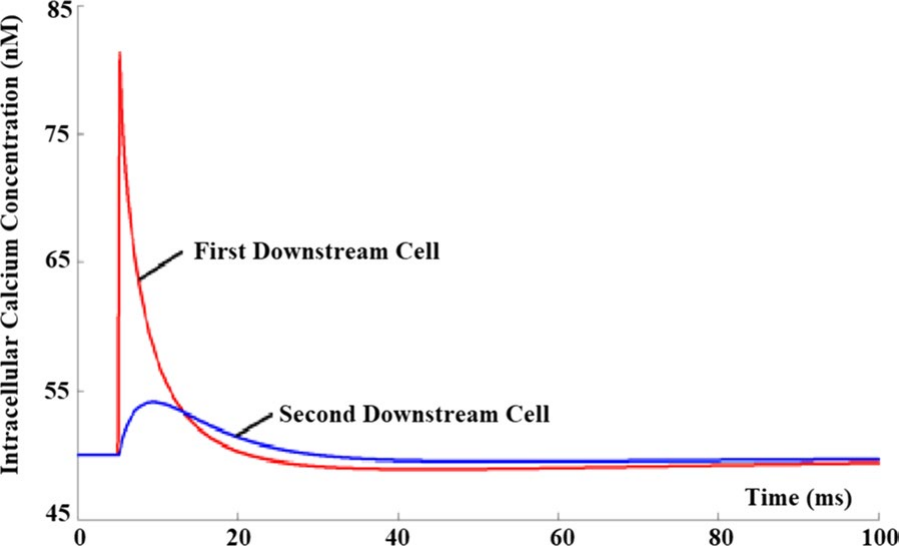
Calcium transients triggered in connected neighbouring cells due to ICW propagation from stimulated source cell

Our model is in a preliminary stage and is yet to be tuned. Literature pertaining to ICW in detrusor is scant. Hence the model would need to be tuned in terms of overall cellular response, with active ion channels integrated. Subsequently, techniques mimicking messenger regeneration would need to be incorporated, besides passive diffusion.

**References**

1. Leybaert L, Sanderson MJ. Intercellular Ca2+ waves: mechanisms and function. Physiological Reviews. 2012; 92(3): 1359-92.
2. Sanderson MJ, Charles AC, Dirksen ER. Mechanical stimulation and intercellular communication increases intracellular Ca2+ in epithelial cells. Cell regulation. 1990; 1(8): 585-96.
3. Appukuttan S, Brain KL, Manchanda R. A computational model of urinary bladder smooth muscle syncytium. Journal of Computational Neuroscience. 2015; 38(1): 167-87.

## P124 Morphologically-detailed reconstruction of cerebellar glial cells

### Laura Keto, Tiina Manninen

#### Tampere University, Faculty of Medicine and Health Technology, Tampere, Finland

##### **Correspondence:** Laura Keto (laura.keto@tuni.fi)

*BMC Neuroscience* 2020, **21(Suppl 1)**:P124

The cerebellar circuitry has been modeled widely, with realistic and accurate models now existing for most of the cerebellar neurons [1]. The glial cell population of the cerebellum, however, has been largely neglected by computational modelers. No realistic whole-cell models have been previously implemented for any of the cerebellar glial cell types; oligodendrocytes, microglia, or astroglia. In this work, we were interested in reconstructing a detailed morphology for the best-known cerebellar astroglial cell type, Bergmann glia. Bergmann glia are radial astrocytes of the cerebellar cortex, with somata located at the Purkinje cell layer, 3-6 long processes extending through the molecular layer, and endfeet. The processes give rise to smaller appendages characterized by microdomains that enwrap neuronal synapses [2]. The reciprocal communication between Bergmann glia and the neighboring neurons is vital for development and plasticity of the cerebellum [3].

Currently no reconstructions of cerebellar glial cells are available in public databases. The reconstruction of Bergmann glia required both an astroglial stem tree as well as a more detailed morphology for reconstructing the astroglial nanoscopic architecture. The stem tree was built with the NEURON CellBuilder tool [4] with values found from literature [2,5]. For the nanoscopic architecture, a 3D reconstruction of a Bergmann glial appendage was recreated based on a video file [2] with AgiSoft Metashape and Blender. As the exact reconstruction of the nanoscopic architecture would be computationally unfeasible, a novel computational tool ASTRO [6] was used to define statistical properties from the reconstructed appendage. The final morphology was assembled with ASTRO and verified functionally by simulating microscopic calcium dynamics with the tool.

**Acknowledgements**: We are very grateful to Prof. Helmut Kettenmann for providing us the video file of Bergmann glia appendage. The work was supported by Academy of Finland (Nos. 326494, 326495).

**References**

1. D’Angelo E, et al. Modeling the cerebellar microcircuit: New strategies for a long-standing issue. Frontiers in Cellular Neuroscience. 2016; 10: 176.
2. Grosche J, et al. Microdomains for neuron-glia interaction: Parallel fiber signaling to Bergmann glial cells. Nature Neuroscience. 1999; 2(2): 139–143.
3. Bellamy TC. Interactions between Purkinje neurones and Bergmann glia. The Cerebellum. 2006; 5(2): 116–126.
4. Carnevale NT, Hines ML. The NEURON book. Cambridge University Press; 2006.
5. Lippman JJ, Lordkipanidze T, Buell ME, Yoon SO, Dunaevsky A. Morphogenesis and regulation of Bergmann glial processes during Purkinje cell dendritic spine ensheathment and synaptogenesis. Glia. 2008; 56(13): 1463–1477.
6. Savtchenko LP, et al. Disentangling astroglial physiology with a realistic cell model in silico. Nature Communications. 2018; 9: 3554.

## P125 A predictive coding model of transitive inference

### Moritz Moeller^1^, Rafal Bogacz^2^, Sanjay Manohar^1^

#### ^1^University of Oxford, Nuffield Department of Clinical Neurosciences, Oxford, United Kingdom; ^2^University of Oxford, MRC Brain Network Dynamics Unit, Oxford, United Kingdom

##### **Correspondence:** Moritz Moeller (moritz.moeller@ndcn.ox.ac.uk)

*BMC Neuroscience* 2020, **21(Suppl 1)**:P125

Transitive inference – deducing that “A is better than C” from the premises “A is better than B” and “B is better than C” – is a basic form of deductive reasoning; both humans and animals are capable of it. However, the mechanism that enables transitive inference is not understood. Partly, this is due to the absence of a concrete, falsifiable formulation of the so-called cognitive explanation of transitive inference (which suggests that subjects combine the facts they observe into a mental model, which they then use for reasoning). In this work, we use the predictive coding method to derive a precise, mathematical implementation of the cognitive explanation of transitive inference (Fig. 1A shows a schematic representation of the model we use). We test our model by simulating a set of typical transitive inference experiments and show that it reproduces several phenomena observed in animal experiments. For example, our model reproduces the gradual acquisition of premise pairs (A > B, B > C) and the simultaneously emerging capability for transitive inference (A>C) (Fig. 1B). We expect this work to lead to novel testable predictions that will inspire future experiments and help to uncover the mechanism behind transitive inference. Further, our work adds support to predictive coding as a universal organising principle of brain function.

**Fig. 1:**
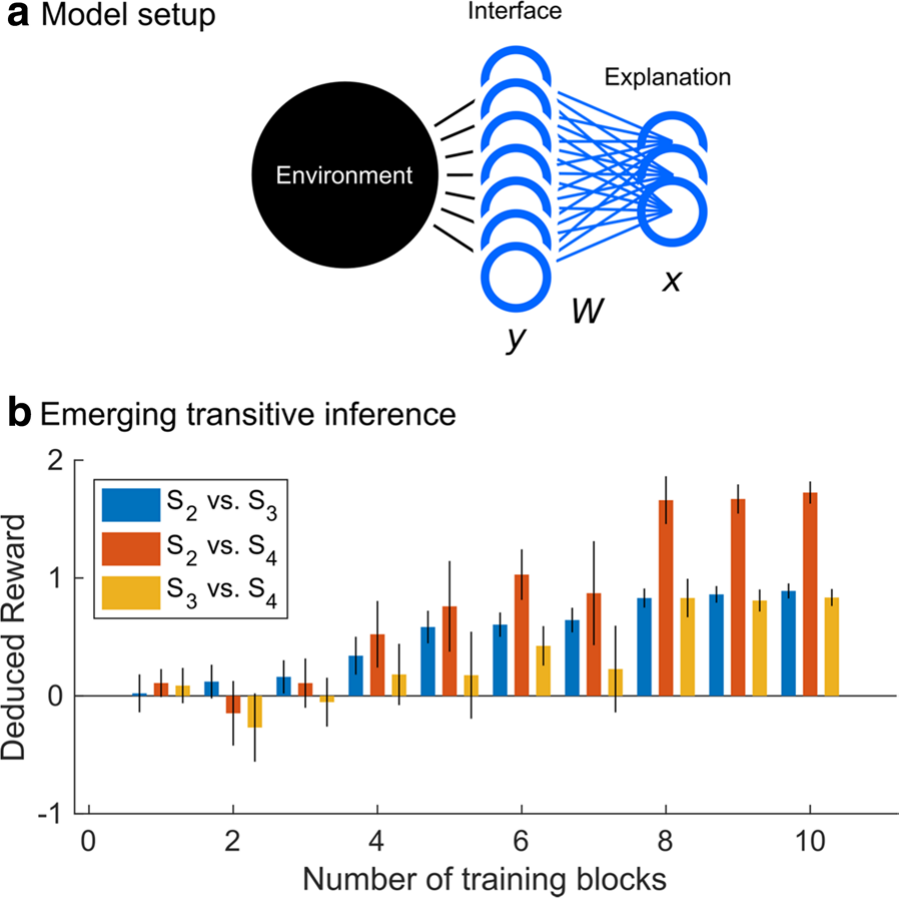
**A** Schematic representation of the predictive coding network used to model transitive inference. **B** Model predictions of pairwise value differences in a hierarchy stimuli A > B > C. As the premises A > B and B > C are learned, the model’s evaluations become consistent with the hierarchy

## P126 Topological Bayesian signal processing with application to EEG

### Christopher Oballe^1^, Alan Czerne^1^, David Boothe^2^, Scott Kerick^2^, Piotr Franaszczuk^2^, Vasileios Maroulas^1^

#### ^1^University of Tennessee, Department of Mathematics, Knoxville, Tennessee, United States of America; ^2^U.S. Army Research Laboratory, Human Research Engineering Directorate, Aberdeen Proving Ground, Maryland, United States of America

##### **Correspondence:** Piotr Franaszczuk (pfranasz@gmail.com)

*BMC Neuroscience* 2020, **21(Suppl 1)**:P126

Electroencephalography is a neuroimaging technique that works by monitoring electrical activity in the brain. Electrodes are placed on the scalp and local changes in voltage are measured over time to produce a collection of time series known as electroencephalograms (EEGs). Traditional signal processing metrics, such as power spectral densities (PSDs), are generally used to analyze EEG since frequency content of EEG is associated with different brain states. Conventionally, PSD estimates are obtained via discrete Fourier transforms. While this method effectively detects low-frequency components because of their high powers, high-frequency activity may go unnoticed because of its relatively weaker power. We employ a topological Bayesian approach that successfully captures even these low-power, high-frequency components of EEG.

Topological data analysis encompasses a broad set of techniques that investigate the shape of data. One of the predominant tools in topological data analysis is persistent homology, which creates topological descriptors called persistence diagrams from datasets. In particular, persistent homology offers a novel technique for time series analysis. To motivate our use of persistent homology to study frequency content of signals, we establish explicit links between features of persistence diagrams, like cardinality and spatial distributions of points, to those of the Fourier series of deterministic signals, specifically the location of peaks and their relative powers. The topological Bayesian approach allows for quantification of these cardinality and spatial distributions by modelling persistence diagrams as marked Poisson point processes.

We test our Bayesian topological method to classify synthetic EEG. We employ three common classifiers: linear regression and support vector machines with linear and radial kernels, respectively. We simulate synthetic EEG with an autoregressive (AR) model, which works by recasting a standard AR model as linearly filtered white noise, enabling straightforward computation of PSDs. The AR model allows us to control the location and width of peaks in PSDs. With this model in hand, we create five classes of signals with peaks in their PSDs at zero to simulate the approximate 1/f behavior of EEG PSDs, four of which also have oscillatory components at 6 Hz (theta), 10 Hz (alpha), 14 Hz (low beta), and 21 Hz (high beta); the fifth class (null) lacks any such component. We repeat this process for two different widths of peaks, narrow (4 Hz) and wide (32 Hz). With data in hand, we extract features using periodograms, persistence diagrams, and our Bayesian topological method, then independently use these features in classification for the wide and narrow width cases. Preliminarily, while both the Bayesian topological method and periodogram features obtain near perfect for the narrow peak case, the Bayesian topological method outperforms the periodogram features over all tested classifiers in the wide peak case.

## P127 A quantification of cross-frequency coupling via topological methods

### Alan Cherne^1^, Christopher Oballe^1^, David Boothe^2^, Melvin Felton^2^, Piotr Franaszczuk^2^, Vasileos Maroulas^1^

#### ^1^University of Tennessee, Department of Mathematics, Knoxville, United States of America; ^2^U.S. Army Combat Capabilities Development Command, United States of America

##### **Correspondence:** David Boothe (david.l.boothe7.civ@mail.mil)

*BMC Neuroscience* 2020, **21(Suppl 1)**:P127

A key feature in electroencephalograms (EEG) is the existence of distinct oscillatory components – theta (4-7 Hz), alpha (8-13Hz), beta (14-30Hz), and gamma (40-100Hz). Cross frequency coupling has been observed between these frequency bands in both the local field potential (LFP) and electroencephalogram (EEG). While the association between activity in distinct oscillatory frequencies and important brain functions is well established, the functional role of cross frequency coupling is poorly characterized, but has been hypothesized to underlie cortical functions like working memory, learning, and computation [1,2].

The most common form of cross frequency coupling observed in brain activity recordings is the modulation of the amplitude of a higher frequency oscillation by the phase of a lower frequency oscillation, a phenomenon known as phase-amplitude coupling (PAC). We present a method for detecting PAC in signals that avoids some pitfalls in existing methods and combines techniques developed in the field of topological data analysis (TDA). When analyzing data using TDA, an object called a persistence diagram, is commonly constructed. In the case of time series the persistence diagram that is generated represents compactly all the peaks and valleys that occur in the signal. We inspect the persistence diagrams to detect the presence of phase-amplitude coupling using the intuition that PAC will impart asymmetry to the upper and lower segments of the diagram. This representation of the data has the advantage that it does not require the choice of Fourier analysis parameters, binning sizes, and phase estimations that are necessary in current methods [3].

We test the performance of our metric on two kinds of synthetic signals, the first is a phenomenological model with varying levels of phase-amplitude coupling [4] as defined by the Kullback-Liebler divergence from the uniform case of signals with no PAC. The second is from simulated single cell neuronal data based on a layer 5 pyramidal cell [5,6]. Finally, we benchmark this method against methods explored previously [4] in EEG data recorded from human subjects.

**References**

1. VanRullen R, Koch C. Is perception discrete or continuous?. Trends in cognitive sciences. 2003; 7(5): 207-13.
2. Canolty RT, Knight RT. The functional role of cross-frequency coupling. Trends in Cognitive Sciences. 2010; 14(11), 507-515.
3. Cohen MX. Assesing transient cross-frequency coupling in EEG data. Journal of Neuroscience Methods. 2008; 168: 494-499.
4. Tort ABL, Komorowski R, Eichenbaum H, Kopell N. Measuring Phase-Amplitude Coupling Between Neural Oscillations of Different Frequencies. Journal of Neurophysiology. 2010; 104: 1195-210.
5. Felton Jr MA, Yu AB, Boothe DL, Oie KS, Franaszczuk PJ. Resonance analysis as a tool for characterizing functional division of layer 5 pyramidal neurons. Frontiers in Computational Neuroscience. 2018; 12:29.
6. Traub RD, et al. Single-column thalamocortical network model exhibiting gamma oscillations, sleep spindles, and epileptogenic bursts. Journal of Neurophysiology. 2005; 93(4): 2194-232.

## P128 An integrate-and-fire model of narrow band modulation in mouse visual cortex

### Nicolò Meneghetti^1,2^, Alberto Mazzoni^1,2^

#### ^1^The Biorobotics Institute, Scuola Superiore Sant’Anna, Pisa, Italy; ^2^Department of Excellence for Robotics and AI, Scuola Superiore Sant’Anna, Pisa, Italy

##### **Correspondence:** Nicolò Meneghetti (nicolo.meneghetti@santannapisa.it)

*BMC Neuroscience* 2020, **21(Suppl 1)**:P128

Gamma band neuronal oscillations are involved with sensory processing ubiquitously in the central nervous system. They emerge from the coordinated interaction of excitation and inhibition and are a biological marker of local active network computations [1]. Visual features as contrast and orientation are known to modulate broad band gamma activity in the primary visual cortex (V1) of primates [2]. In mouse V1, however, a narrow band within gamma oscillation was found to display specific functional sensitivity to visual features [3].

Here we present a network of recurrent excitatory-inhibitory spiking neurons reproducing the gamma narrow band dynamics in mouse V1 observed in [3], building on previous works of our group [4,5]. By combining experimental data analysis and simulations, we show that a proper design of the simulated thalamic input results in the network to exhibit both narrow and broad band gamma activity.

We reproduced the spectral and temporal modulations of V1 local field potentials of awake mice presented with gratings of different contrast levels by approximating the thalamic input rate with two linear functions defined over complementary contrast ranges. We propose a theoretical framework in which the external thalamic drive is responsible for inducing the emergence of broad by triggering cortical resonances and narrow band gamma activity by inducing entrainment to an oscillatory drive. Our results support in particular the hypothesis of a subcortical origin of the narrow gamma band [3].

Our network provides a simple and effective model of contrast-induced gamma activity in rodents V1. The model could be easily extended to reproduce the modulation of V1 gamma activity induced by other visual stimulus features. Moreover, the model could help to investigate network dynamics responsible for pathological dysfunctions of physiological visual information processing in mice.

**References**

1. Buzsáki G, Wang XJ. Mechanisms of gamma oscillations. Annual Review of Neuroscience. 2012; 35: 203-25.
2. Henrie JA, Shapley R. LFP power spectra in V1 cortex: the graded effect of stimulus contrast. Journal of Neurophysiology. 2005; 94(1): 479-90.
3. Saleem AB, et al. Subcortical source and modulation of the narrowband gamma oscillation in mouse visual cortex. Neuron. 2017; 93(2): 315-22.
4. Mazzoni A, Brunel N, Cavallari S, Logothetis NK, Panzeri S. Cortical dynamics during naturalistic sensory stimulations: experiments and models. Journal of Physiology-Paris. 2011; 105(1-3): 2-15.
5. Mazzoni A, Linden H, Cuntz H, Lansner A, Panzeri S, Einevoll GT. Computing the local field potential (LFP) from integrate-and-fire network models. PLoS Computational Biology. 2015; 11(12): e1004584.

## P129 Correlated inputs to the striatum generate beta over-synchronization in in silico cortico-basal ganglia network

### Elena Manferlotti^1^, Matteo Vissani^2^, Arvind Kumar^3^, Alberto Mazzoni^2^

#### ^1^Scuola Superiore Sant’Anna, The Biorobotics Institute and Department of Excellence for Robotics and AI, Pisa, Italy; ^2^Scuola Superiore Sant’Anna Pisa, The Biorobotic institute, Pisa, Italy; ^3^KTH Royal Institute of Technology, Computational Brain Science, Stockholm, Sweden

##### **Correspondence:** Elena Manferlotti (elena.manferlotti@gmail.com)

*BMC Neuroscience* 2020, **21(Suppl 1)**:P129

Parkinson’s Disease (PD) is known to be associated with over-synchronized oscillations in the beta frequency range (13-35Hz) in motor cortex and basal ganglia (BG) [1]. Although the mechanisms underlying the emergence of these oscillations are poorly understood, several excitatory-inhibitory loops have been identified in the cortex-BG networks that might initiate or generate them.

Recent experimental data suggests that striatal spiny projection neurons (SPNs) are phase locked to the beta oscillation cycles [2]. Indeed, transient change in the SPNs firing rate is sufficient to unleash oscillations into the mutually connected globus pallidus externus (GPe) sub-thalamic nucleus (STN) network [3].

Here, we investigate the effect of temporal synchrony of SPNs activity on beta oscillations simulating a biologically plausible BG model with spiking neurons [4]. The likely source of correlations in the SPNs is thalamo-cortical input, since striatal connectivity is too sparse. Therefore, we injected correlated inputs to the SPNs. Our model showed the emergence of beta band aberrant synchronization as the network switches from the uncorrelated to the correlated input. Crucially, inputs displayed a fixed firing rate, that is the over-synchronization emerged only because of the input synchrony. Furthermore, increased input correlation resulted in enhanced Globus Pallidus internus (GPi) firing rate as observed experimentally [5]. Next, we investigated the possible consequences of these results for Deep Brain Stimulation (DBS) simulating high frequency injections into the STN. Our preliminary results showed that even a short window of stimulation was enough to reduce beta oscillations in the firing rate of STN, GPe and GPi nuclei.

Our study provides innovative observations about the origin and propagation of PD-related beta oscillations in the BG and their reduction due to DBS. It paves the way toward in silico testing of DBS parameters that could be used to determine optimal parameters of stimulation offline rather than during surgical implants.

References

1. Oswal A, Brown P, Litvak V. Synchronized neural oscillations and the pathophysiology of Parkinson’s disease. Current Opinion in Neurology. 2013; 26: 662–70.
2. Sharott A, et al. A Population of Indirect Pathway Striatal Projection Neurons Is Selectively Entrained to Parkinsonian Beta Oscillations. Journal of Neuroscience. 2017; 37: 9977–98.
3. Mirzaei A, et al. Sensorimotor processing in the basal ganglia leads to transient beta oscillations during behavior. Journal of Neuroscience. 2017; 37(46): 11220-32.
4. Lindahl M, Hellgren Kotaleski J. Untangling Basal Ganglia Network Dynamics and Function: Role of Dopamine Depletion and Inhibition Investigated in a Spiking Network Model. eNeuro. 2016; 3.
5. Magnin M, Morel A, Jeanmonod D. Single-unit analysis of the pallidum, thalamus and subthalamic nucleus in parkinsonian patients. Neuroscience. 2000; 96: 549–64.

## P130 Unifying information theory and machine learning in a model of cochlear implant electrode discrimination

### Xiao Gao^1^, David Grayden^1^, Mark McDonnell^2^

#### ^1^University of Melbourne, Department of Biomedical Engineering, Melbourne, Australia; ^2^University of South Australia, School of Information Technology and Mathematical Sciences, Adelaide, Australia

##### **Correspondence:** Xiao Gao (xiao.gao@unimelb.edu.au)

*BMC Neuroscience* 2020, **21(Suppl 1)**:P130

Despite the success of cochlear implants (CIs) over more than three decades, wide inter-subject variability in speech perception is reported [1]. The key factors that cause variability between users are unclear. We previously developed an information theoretic modelling framework that enables estimation of the optimal number of electrodes and quantification of electrode discrimination ability [2,3]. However, the optimal number of electrodes was estimated based only on statistical correlations between channel outputs and inputs, and the model did not quantitatively model psychophysical measurements and study inter-subject variability.

Here, we unified information theoretic and machine learning techniques to investigate the key factors that may limit the performance of CIs. The framework used a neural network classifier to predict which electrode was stimulated for a given simulated activation pattern of the auditory nerve, and mutual information was then estimated between the actual stimulated electrode and the predicted one.

Using the framework, electrode discrimination was quantified with a range of parameter choices, as shown in Figure 1. The columns from left to right show how the distance between electrodes and auditory nerve fibres, *r*, the number of surviving fibres, *N*, the maximum current level (modelled as the percentage of surviving fibres, *N*, that generate action potentials for a given stimulated electrode), and the attenuation in electrode current, *A*, affect the model performance, respectively. The parameters were chosen to reflect the key factors that are believed to limit the performance of CIs. The model shows sensitivity to parameter choices, where smaller *r*, larger *N*, and higher attenuation in current lead to higher mutual information and improved classification.

**Fig. 1:**
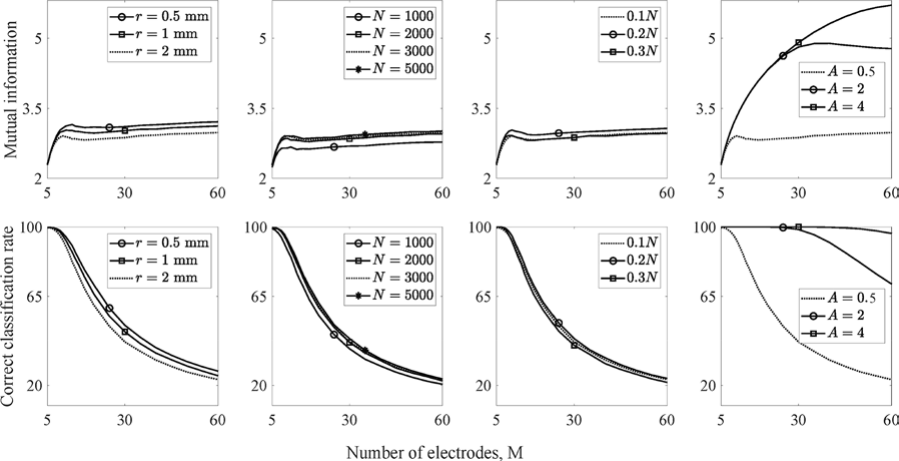
Model performance with a range of parameter choices

This approach provides a flexible framework that may be used to investigate the key factors that limit the performance of cochlear implants. We aim to investigate its application to personalised configurations of CIs.

**Acknowledgments:** This work is supported by a McKenzie Fellowship, The University of Melbourne.

**References**

1. Holden LK, et al. Factors affecting open-set word recognition in adults with cochlear implants. Ear and hearing. 2013; 34(3): 342.
2. Gao X, Grayden DB, McDonnell MD. Stochastic information transfer from cochlear implant electrodes to auditory nerve fibers. Physical Review E. 2014; 90(2): 022722.
3. Gao X, Grayden DB, McDonnell MD. Modeling electrode place discrimination in cochlear implant stimulation. IEEE Transactions on Biomedical Engineering. 2016; 64(9): 2219-29.

## P131 Unsupervised metadata tagging of computational neuroscience literature, towards question answering

### Evan Cudone^1^, Robert McDougal^2^

#### ^1^Yale University, Computational Biology and Bioinformatics, New Haven, Connecticut, United States of America; ^2^Yale University, Department of Neuroscience, New Haven, Connecticut, United States of America

##### **Correspondence:** Evan Cudone (evan.cudone@yale.edu)

*BMC Neuroscience* 2020, **21(Suppl 1)**:P131

Curation and knowledge dissemination of the computational neuroscience field requires many unique considerations as it utilizes language, methods, and ideas from diverse areas including biology, chemistry, physics, mathematics, medicine, and computer science. In order to effectively facilitate curation and knowledge dissemination for the computational neuroscience community we must first develop a robust representation of its existing literature. Using unsupervised topic modeling approaches, a metadata tagging schema was developed for computational neuroscience literature from ModelDB (a repository of computational neuroscience models), and compared to that of a larger neuroscience corpus. This analysis shows key differences in the types of discoveries and knowledge addressed in neuroscience and its computational subdiscipline, and gives insight into how an automated question answering system might differ between the two.

## P132 Preventing retinal ganglion cell axon bundle activation with oriented rectangular electrodes

### Wei Tong^1^, Michael R Ibbotson^2^, Hamish Meffin^3^

#### ^1^National Vision Research Institute, Melbourne, Australia; ^2^Australian College of Optometry, The National Vision Research Institute, Melbourne, Australia; ^3^University of Melbourne, Biomedical Engineering, Melbourne, Australia

##### **Correspondence:** Wei Tong (wei.tong@unimelb.edu.au)

*BMC Neuroscience* 2020, **21(Suppl 1)**:P132

Retinal prostheses can restore visual sensations in people that have lost their photoreceptors by electrically stimulating surviving retinal ganglion cells (RGCs). Currently, there are mainly three types of retinal prostheses under development, based on their implantation locations: epi-retinal, sub-retinal and suprachoroidal [1]. Clinical studies from all three types of devices indicate that, although a sense of vision can be restored, the visual acuity obtained is limited and functional vision, such as navigation and facial recognition remains challenging. One major difficulty is associated with the low spatial resolution obtained from electrical stimulation, i.e. the large spread of activation amongst RGCs leads to blurred or distorted visual percepts. Particularly, with epi-retinal implants, experiments have revealed that the leading cause of widespread activation is the unintended activation of passing RGC axons, which lead to elongated phosphines in patients [2].

This work proposes to use rectangular electrodes oriented parallel to the axon bundles to prevent the activation of passing axon bundles. Here, we first used simulation to investigate the interaction of neural tissue orientation and stimulation electrode configuration on the RGC activation patterns. A four-layer computational model of epiretinal extracellular stimulation that captures the effect of neurite orientation in anisotropic tissue was applied, as previously described [3], using a volume conductor model known as the cellular composite model. As shown in Figure 1a, our model shows that stimulating with rectangular electrode aligned with the nerve fiber layer (i.e. passing axon bundles), can be used to achieve selective activation of axon initial segments, rather than passing fibers.

**Fig. 1:**
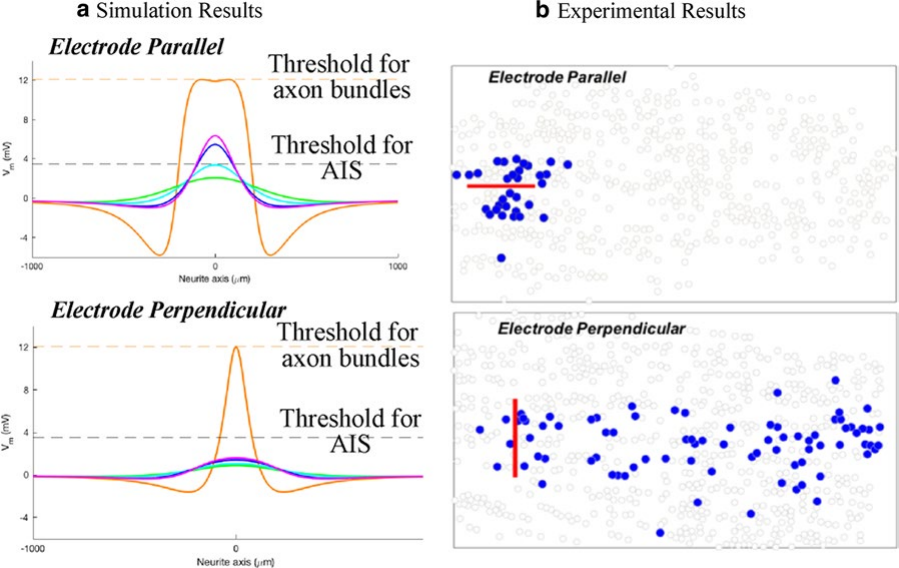
Simulated membrane potentials **a** and experimental calcium imaging **b** results both indicate that a rectangular electrode oriented parallel to the axon bundles can lead to localised RGC activation by avoiding the unintended activation of passing axon bundles

The simulation results were then confirmed with experiments. Here, data were acquired from adult Long Evan rats by recording the response of RGCs from whole-mount retina preparations using calcium imaging. Electrical stimulation was delivered through a diamond coated carbon fiber electrode with a length of 200µm and diameter of 10µm. The electrode was placed either parallel or perpendicular to the RGC axon bundles. Biphasic stimuli with different pulse durations of 33-500µs were tested. Our experimental observations (Fig. 1b) are consistent with the expectations of the simulations, and the use of rectangular electrodes placed parallel to axon bundles can significantly reduce the activation of RGC axon bundles. When using biphasic stimulation as short as 33µs, the activated RGCs were mostly confined to the region below or very close-to the electrode, as observed using confocal microscopy.

To conclude, this work provides a stimulation strategy for reducing the spread of RGC activation for epi-retinal prostheses. Using ultrashort pulses together with rectangular electrodes parallel to the RGC axon bundles, the performance of epi-retinal prostheses will be improved significantly, thus promising to restore a higher quality of vision to the blind.

**References**

1. Weiland JD, Walston ST, Humayun MS. Electrical stimulation of the retina to produce artificial vision. Annual Review of Vision Science. 2016; 2: 273-94.
2. Nanduri D, et al. Frequency and amplitude modulation have different effects on the percepts elicited by retinal stimulation. Investigative ophthalmology & visual science. 2012; 53(1): 205-14.
3. Esler TB, et al. Minimizing activation of overlying axons with epiretinal stimulation: The role of fiber orientation and electrode configuration. PloS one. 2018; 13(3): e0193598.

## P133 Recurrent neural networks trained in multisensory integration tasks reveal a diversity of selectivity and structural properties

### Amparo Gilhuis^1^, Shirin Dora^2^, Cyriel Pennartz^1^, Jorge Mejias^1^

#### ^1^University of Amsterdam, Swammerdam Institute for Life Sciences, Amsterdam, Netherlands; ^2^Ulster University, Intelligent Systems Research Centre, Londonderry, United Kingdom

##### **Correspondence:** Jorge Mejias (jorge.f.mejias@gmail.com)

*BMC Neuroscience* 2020, **21(Suppl 1)**:P133

The brain continuously processes sensory information from multiple modalities, giving rise to internal representations of the outside world. If and how the information from multiple modalities is being integrated has extensively been investigated over the past years, leading to more insight in multisensory integration (MSI) and its underlying mechanisms [1]. However, the different experimental paradigms used to investigate MSI involve different cognitive resources and situational demands. In this study, we investigated how different experimental paradigms of MSI reflect on behavior output and in their corresponding neural activity patterns. We did so by designing a recurrent neural network (RNN) with the biological plausible feature of differentiating between excitatory and inhibitory units [2]. For each of the three multisensory processing tasks considered [3,4], an RNN was optimized to perform the tasks with similar performance as found in animals. Network models trained on different experimental paradigms showed significant distinct selectivity and connectivity patterns. Selectivity for both modality and choice was found in network models that were trained on the paradigm that involved higher cognitive resources. Network models trained on paradigms that involve more bottom-up processes mostly experienced choice selectivity. Increasing the level of network noise in network models that at first did not experience modality selectivity led to an increase in modality selectivity. We propose that a higher range of selectivity arises when a task is more demanding, either due to higher network noise (which makes the task harder for the animal) or a more difficult experimental paradigm. The higher range of selectivity is thought to improve the flexibility of the network model, which could be a necessity for the network models to achieve good performance, and the resulting neural heterogeneity could be used for more general information processing strategies [5,6].

**Acknowledgements:** This work was partially supported by the European Union’s Horizon 2020 Framework Programme for Research and Innovation under the Specific Grant Agreement No. 785907.

**References**

1. Chandrasekaran C. Computational principles and models of multisensory integration. Current Opinion in Neurobiology. 2017; 43: 25-34.
2. Song HF, Yang GR, Wang XJ. Training excitatory-inhibitory recurrent neural networks for cognitive tasks: a simple and flexible framework. PLoS Computational Biology. 2016; 12(2): e1004792.
3. Raposo D, Kaufman MT, Churchland AK. A category-free neural population supports evolving demands during decision-making. Nature Neuroscience. 2014; 17: 1784.
4. Meijer GT, Pie JL, Dolman TL, Pennartz C, Lansink CS. Audiovisual integration enhances stimulus detection performance in mice. Frontiers in Behavioral Neuroscience. 2018; 12: 231.
5. Mejias JF, Longtin A. Optimal heterogeneity for coding in spiking neural networks. Physical Review Letters. 2012; 108(22): 228102.
6. Mejias JF, Longtin A. Differential effects of excitatory and inhibitory heterogeneity on the gain and asynchronous state of sparse cortical networks. Frontiers in Computational Neuroscience. 2014; 8: 107.

## P134 A fluid hierarchy in multisensory integration properties of large-scale cortical networks

### Ronaldo Nunes^1^, Marcelo Reyes^1^, Raphael de Camargo^1^, Jorge Mejias^2^

#### ^1^Universidade Federal do ABC, Center for Mathematics, Computing and Cognition, Santo Andre, Brazil; ^2^University of Amsterdam, Swammerdam Institute for Life Sciences, Amsterdam, Netherlands

##### **Correspondence:** Jorge Mejias (jorge.f.mejias@gmail.com)

*BMC Neuroscience* 2020, **21(Suppl 1)**:P134

A fundamental ingredient for perception is the integration of information from different sensory modalities. This process, known as multisensory integration (MSI), has been studied extensively using animal and computational models [1]. It is not yet clear, however, how different brain areas contribute to MSI, and identifying relevant areas remains challenging. Part of the reason is that simultaneous electrophysiological recordings from different brain areas has developed only recently [2], and the intensity, noise profile and delay responses are diverse for different sensory signals [1]. Furthermore, computational models have traditionally focused only on a few areas, a limitation imposed by the lack of reliable anatomical data on brain networks. We present here a theoretical and computational study of the mechanisms underlying MSI in the mouse brain, by constraining our model with a recently acquired anatomical brain connectivity dataset [3]. Our simulations of the resulting large-scale cortical network reveal the existence of a hierarchy of crossmodal excitability properties, with areas at the top of the hierarchy being the best candidates for integrating information from multiple modalities. Furthermore, our model predicts that the position of a given area in such hierarchy is highly fluid and depends on the strength of the sensory input received by the network. For example, we observe that the particular set of areas integrating visuotactile stimuli changes depending on the level of visual contrast. By simulating a simplified network model and developing its corresponding mean-field approximation, we determine that the origin of such hierarchical dynamics is the structural heterogeneity of the network, which is a salient property of cortical networks [3,4]. Finally, we extend our results to macaque cortical networks [5] to show that the hierarchy of crossmodal excitability is also present in other mammals, and we characterize how frequency-specific interactions are affected by hierarchical dynamics and define functional connectivity [6]. Our work provides a compelling explanation as of why is it not possible to identify unique MSI areas even for a well-defined multisensory task, and suggests that MSI circuits are highly context-dependent.

**Acknowledgements**: This study was financed in part by the University of Amsterdam and the Coordenacao de Aperfeicoamento de Pessoal de Nivel Superior Brasil (Capes) - Finance Code 001.

**References**

1. Chandrasekaran C. Computational principles and models of multisensory integration. Current Opinion in Neurobiology. 2017; 43: 25-34.
2. Hong G, Lieber CM. Novel electrode technologies for neural recordings. Nature Reviews Neuroscience. 2019; 20(6): 330-45.
3. Gamanut R, et al. The mouse cortical connectome, characterized by an ultra-dense cortical graph, maintains specificity by distinct connectivity profiles. Neuron. 2018; 97: 698-715.
4. Mejias JF, Longtin A. Differential effects of excitatory and inhibitory heterogeneity on the gain and asynchronous state of sparse cortical networks. Frontiers in Computational Neuroscience. 2014; 8: 107.
5. Mejias JF, Murray JD, Kennedy H, Wang XJ. Feedforward and feedback frequency-dependent interactions in a large-scale laminar network of the primate cortex. Science Advances. 2016; 2(11): e1601335.
6. Nunes RV, Reyes MB, De Camargo RY. Evaluation of connectivity estimates using spiking neuronal network models. Biological Cybernetics. 2019; 113(3): 309-20.

## P135 A cortical model examining mismatch negativity deficits in schizophrenia

### Gili Karni^1^, Christoph Metzner^2^

#### ^1^Minerva at KGI, San Francisco, California, United States of America; ^2^Technische Universität Berlin, Department of Software Engineering and Theoretical Computer Science, Berlin, Germany

##### **Correspondence:** Christoph Metzner (cmetzner@ni.tu-berlin.de)

*BMC Neuroscience* 2020, **21(Suppl 1)**:P135

Recent advances in computational modeling, genome-wide association studies, neuroimaging, and theoretical neuroscience pose better opportunities to study neuropsychiatric disorders, such as schizophrenia (SZC) [1]. However, despite a repeated examination of its well-characterized phenotypes, our understanding of SZC’s neurophysiological biomarkers or cortical dynamics remain elusive.

This study presents a biophysical spiking neuron model of perceptual inference, based on the predictive coding framework [2]. The model, implemented in NetPyNE [3], incorporates various single-cell models of both excitatory and inhibitory neurons [4,5], mimicking the circuits of the primary auditory cortex. This model allows for the exploration of the effects bio-genetic variants (expressed via ion-channels or synaptic mechanism alterations, see [6]) have on auditory mismatch negativity (MMN) deficits, a common biomarker for SZC [7]. More particularly, the model distinguishes between repetition suppression and prediction error and examines their respective contribution to the MMN. The first part of this report establishes the model’s explanatory power using two well-known paradigms: the oddball paradigm and the cascade paradigm. Both can reproduce the electrophysiological measures of the MMN among healthy subjects. Later, via tuning the parameters of single-neuron equations or the network’s synaptic weights, the model exhibits the expected LFP changes associated with SZC [8].

Therefore, this model enables exploring how biogenetic alterations affect the underlying components of the observed MMN deficits. Novel, yet preliminary, predictions are presented and suggested future steps for validations are listed. This model could support studies exploring genetic effects on the MMN (or other aspects of predictive coding) in the auditory cortex.

**References**

1. Krystal JH, et al. Computational psychiatry and the challenge of schizophrenia. 2017.
2. Bastos AM, et al. Canonical microcircuits for predictive coding. Neuron. 2012; 76(4): 695-711.
3. Garrido MI, Kilner JM, Stephan KE, Friston KJ. The mismatch negativity: a review of underlying mechanisms. Clinical Neurophysiology. 2009; 120(3): 453-63.
4. Beeman, D. Comparison with human layer 2/3 pyramidal cell dendritic morphologies. Poster session presented at the meeting of Society for Neuroscience 2018, San Diego.
5. Vierling-Claassen D, Cardin J, Moore CI, Jones SR. Computational modeling of distinct neocortical oscillations driven by cell-type selective optogenetic drive: separable resonant circuits controlled by low-threshold spiking and fast-spiking interneurons. Frontiers in Human Neuroscience. 2010 Nov; 4: 198.
6. Mäki-Marttunen T, et al. Functional effects of schizophrenia-linked genetic variants on intrinsic single-neuron excitability: a modeling study. Biological Psychiatry: Cognitive Neuroscience and Neuroimaging. 2016; 1(1): 49-59.
7. Dura-Bernal S, et al. NetPyNE, a tool for data-driven multiscale modeling of brain circuits. Elife. 2019; 8: e44494.
8. Michie PT, Malmierca MS, Harms L, Todd J. The neurobiology of MMN and implications for schizophrenia. Biological psychology. 2016; 116: 90-7.

## P136 Effect of independent noise on the synchronization of interacting excitatory-inhibitory networks

### Lucas Rebscher^1^, Christoph Metzner^2^

#### ^1^Technische Universität Berlin, Neural Information Processing, Berlin, Germany; ^2^Technische Universität Berlin, Department of Software Engineering and Theoretical Computer Science, Berlin, Germany

##### **Correspondence:** Lucas Rebscher (lucas.rebscher@campus.tu-berlin.de)

*BMC Neuroscience* 2020, **21(Suppl 1)**:P136

Gamma rhythms play a major role in different processes in the brain, such as attention, working memory and sensory processing. The communication-through-coherence (CTC) hypothesis [1,2] suggests that synchronization in the gamma band is one of the key mechanisms in neuronal communication and counterintuitively noise can have beneficial effects on the communication [3].

Recently, Meng et al. [4] showed that synchronization across interacting networks of inhibitory neurons increases while synchronization within these networks decreases when neurons are subject to independent noise. They focused on inhibitory-inhibitory connections with gamma band activity produced by the interneuronal network gamma mechanism (ING). However, experimental and modeling studies [5] point towards an important role of the pyramidal-interneuronal network gamma (PING) mechanism in the cortex and the established view is that cortico-cortical connections are predominately excitatory [6].

We build up on Meng et al. [4] results and intend to verify if their findings can be observed in interacting gamma rhythms produced by a PING mechanism. In our ongoing research we model interacting excitatory-inhibitory networks and analyze how synchronization changes depending on strength and correlation of noise in different network settings. We expect to see the same effect in our model as (1) the delay of spiking by inhibition is integral to both ING and PING and (2) Meng et al. [4] replicated the effect in different neuron models as well as relaxation oscillators.

Uncovering whether, and if yes, under which conditions, stochastic fluctuations can also have beneficial effects on gamma oscillations produced by a PING mechanism, would further our understanding of inter-regional communication. However, importantly, it might also yield mechanistic explanations for altered neuronal dynamics in psychiatric disorders, since for example, disturbances in neuronal oscillations in the gamma band, especially reduced synchronization, are a key finding in schizophrenia [7].

**References**

1. Fries, P. A mechanism for cognitive dynamics: neuronal communication through neuronal coherence. Trends in Cognitive Sciences. 2005; 9(10): 474-480.
2. Fries P. Rhythms for cognition: communication through coherence. Neuron. 2015, 88(1), 220-235.
3. McDonnell MD, Ward LM. The benefits of noise in neural systems: bridging theory and experiment. Nature Reviews Neuroscience. 2011; 12(7): 415-425.
4. Meng JH, Riecke H. Synchronization by uncorrelated noise: interacting rhythms in interconnected oscillator networks. Scientific Reports. 2018; 8(1): 1-4.
5. Tiesinga P, Sejnowski TJ. Cortical enlightenment: are attentional gamma oscillations driven by ING or PING?. Neuron. 2009; 63(6): 727-732.
6. Lodato S, Arlotta P. Generating neuronal diversity in the mammalian cerebral cortex. Annual Review of Cell and Developmental Biology. 2015; 31: 699-720.
7. Uhlhaas P, Singer W. Oscillations and neuronal dynamics in schizophrenia: the search for basic symptoms and translational opportunities. Biological Psychiatry. 2015; 77(12): 1001-1009.

## P137 General anesthesia reduces complexity and temporal asymmetry of the informational structures derived from neural recordings in Drosophila

### Roberto Munoz^1^, Angus Leung^2^, Aidan Zecevik^1^, Felix Pollock^1^, Dror Cohen^3^, Bruno can Swinderen^4^, Naotsugu Tsuchiya^5^, Kavan Modi^1^

#### ^1^Monash University, School of Physics and Astronomy, Melbourne, Australia; ^2^Monash University, School of Psychological Sciences, Melbourne, Australia; ^3^National Institute of Information and Communications Technology, Osaka, Japan; ^4^The University of Queensland, St Lucia, Australia; ^5^Monash University, School of Psychological Sciences and Turner Institute for Brain and Mental Health, Melbourne, Australia

##### **Correspondence:** Roberto Munoz (roberto.munoz@monash.edu)

*BMC Neuroscience* 2020, **21(Suppl 1)**:P137

We apply techniques from the field of computational mechanics to evaluate the statistical complexity of neural recording data from fruit flies. First, we connect statistical complexity to the flies’ level of conscious arousal, which is manipulated by general anaesthesia (isoflurane). We show that the complexity of even single channel time series data decreases under anaesthesia. The observed difference in complexity between the two states of conscious arousal increases as higher orders of temporal correlations are taken into account. We then go on to show that, in addition to reducing complexity, anaesthesia also modulates the informational structure between the forward and reverse-time neural signals. Specifically, using three distinct notions of temporal asymmetry we show that anaesthesia reduces temporal asymmetry on information-theoretic and information-geometric grounds. In contrast to prior work, our results show that: (1) Complexity differences can emerge at very short time scales and across broad regions of the fly brain, thus heralding the macroscopic state of anaesthesia in a previously unforeseen manner, and (2) that general anaesthesia also modulates the temporal asymmetry of neural signals. Together, our results demonstrate that anaesthetised brains become both less structured and more reversible.

## P138 Processing Capacity of recurrent spiking networks

### Tobias Schulte to Brinke^1^, Fahad Khalid^2^, Renato Duarte^3^, Abigail Morrison^1^

#### ^1^Forschungszentrum Jülich, Jülich, Germany; ^2^Forschungszentrum Jülich, Institute for Advanced Simulation and Jülich Supercomputing Centre, Jülich, Germany; ^3^Jülich Research Center, Institute of Neuroscience and Medicine (INM-6) and Institute for Advanced Simulation (IAS-6), Jülich, Germany

##### **Correspondence:** Tobias Schulte to Brinke (t.schulte.to.brinke@fz-juelich.de)

*BMC Neuroscience* 2020, **21(Suppl 1)**:P138

One of the most prevalent characteristics of neurobiological systems is the abundance of recurrent connectivity. Regardless of the spatial scale considered, recurrence is a fundamental design principle and a core anatomical feature, permeating the micro-, meso- and macroscopic levels. In essence, the brain (and, in particular, the mammalian neocortex) can be seen as a large recurrent network of recurrent networks. Despite the ubiquity of these observations, it remains unclear whether recurrence and the characteristics of its biophysical properties correspond to important functional specializations and if so, to what extent.

Intuitively, from a computational perspective, recurrence allows information to be propagated in time, i.e. past information reverberates so as to influence online processing, endowing the circuits with memory and sensitivity to temporal structure. However, even in its simpler formulations, the functional relevance and computational consequences of recurrence in biophysical models of spiking networks are not clear or unambiguous and its effects vary depending on the type and characteristics of the system under analysis and the nature of the computational task. Therefore, it would be extremely useful, from both an engineering and a neurobiological perspective, to know to what extent is recurrence necessary for neural computation.

In this work, we set out to quantify the extent to which recurrence modulates a circuit’s computational capacity, by systematically measuring its ability to perform arbitrary transformations on an input, following [1]. By varying the strength and density of recurrent connections in balanced networks of spiking neurons, we evaluate the effect of recurrence on the complexity of the transformations the circuit can carry out and on the memory it is able to sustain. Preliminary results demonstrates some constraints on recurrent connectivity that optimize its processing capabilities for mappings that involve both linear memory and varying degrees of nonlinearity.

Additionally, given that the metric we employ is particularly computationally-heavy (evaluating the system’s capacity to represent thousands of target functions), a careful optimization and parallelization strategy is employed, enabling its application to networks of neuroscientific interest. We present a highly scalable and computationally efficient software, which pre-computes the thousands of necessary target polynomial functions for each point in a large combinatorial space, accesses these target functions through an efficient lookup operation, caches functions that need to be called multiple times with the same inputs and optimizes the most compute-intensive hotspots with Cython. In combination with MPI for internode communication this results in a highly scalable and computationally efficient implementation to determine the processing capacity of a dynamical system.

**Acknowledgments:** The authors gratefully acknowledge the computing time granted by the JARA Vergabegremium and provided on the JARA Partition part of the supercomputer JURECA at Forschungszentrum Jülich.

**Reference**

1. Dambre J, Verstraeten D, Schrauwen B, Massar S. Information Processing Capacity of Dynamical Systems. Scientific Reports. 2012; 2: 514.

## P139 Stereotyped population dynamics in the medial entorhinal cortex

### Soledad G Cogno^1^, Flavio Donato^2^, Horst A Obenhaus^1^, Irene R Jacobsen^1^, May-Britt Moser^1^, Edvard I Moser^1^

#### ^1^NTNU, Kavli Institute for Systems Neuroscience and Centre for Neural Computation, Trondheim, Norway; ^2^University of Basel, Biozentrum, Basel, Switzerland

##### **Correspondence:** Soledad G Cogno (soledad.g.cogno@ntnu.no)

*BMC Neuroscience* 2020, **21(Suppl 1)**:P139

The medial entorhinal cortex (MEC) supports the brain’s representation of space with distinct cell types (grid, border, object-vector, head-directions and speed cells). Since no single sensory stimulus can faithfully predict the firing of these cells, attractor network models postulate that spatially-tuned firing emerges from specific connectivity motives. To determine how those motives constrain the self-organized activity in the MEC, we tested mice in a spontaneous locomotion task under sensory-deprived conditions, when activity likely is determined by the intrinsic structure of the network. Using 2-photon calcium imaging, we monitored the activity of large populations of MEC neurons in mice running on a wheel in darkness.

To reveal network dynamics we applied dimensionality reduction techniques to the spike matrix. This way we unveiled the presence of motifs that involve the sequential activation of neurons (“waves”). Waves lasted from tens of seconds to minutes, swept through the entire network of active cells and did not exhibit any anatomical organization. Waves were not found in spike-time-shuffled data. Furthermore, waves did not map the position of the mouse on the wheel and were not restricted to running epochs. Single neurons exhibited a wide range of locking degrees to the waves, indicating that the observed dynamics is a population effect rather than a single cell phenomenon. Overall, our results suggest that a large fraction of MEC-L2 neurons participates in common global dynamics that often takes the form of stereotyped waves. These activity patterns might couple the activity of neurons with distinct tuning characteristics in MEC.

## P140 How hard are NP-complete problems for humans?

### Pablo Franco, Karlo Doroc, Nitin Yadav, Peter Bossaerts, Carsten Murawski

#### University of Melbourne, Department of Finance, Melbourne, Australia

##### **Correspondence:** Pablo Franco (jfranco1@student.unimelb.edu.au)

*BMC Neuroscience* 2020, **21(Suppl 1)**:P140

It is widely accepted that humans have limited cognitive resources and that these finite resources impose restrictions on what the brain can compute. Although endowed with limited computational power, humans are still presented daily with decisions that require solving complex problems. This raises a tension between computational capacity and the computational requirements of solving a problem. In order to understand how hardness of problems affect problem-solving ability we propose a measure to quantify the difficulty of problems for humans. For this we make use of computational complexity theory, a widely studied theory used to quantify the hardness of problems for electronic computers. It has been proposed that computational complexity theory can be applied to humans, but it remains an open empirical question whether this is the case.

We study how difficulty of problems affects decision quality in complex problems by studying a measure of expected difficulty over random instances (i.e. random cases) of a problem. This measure, which we refer to as instance complexity (IC), quantifies the expected hardness of a decision problems; that is, problems that have a yes/no answer. More specifically, this measure captures how constrained the problem is, based on a small number of features of the instance. Overall, IC has three main advantages. Firstly, it is a well-studied measure that has been proven to be applicable to a large range of problems for electronic computers. Secondly, it allows calculation of expected hardness of a problem ex-ante, that is, before solving the problem. And lastly, it captures complexity that is independent of a particular algorithm or model of computation. Thus, it is considered to characterize the inherent computational complexity of random instances, which is independent of the system solving it.

In this study we test whether IC is a generalizable measure, for humans, of the expected hardness of solving a problem. For this purpose, we ran a set of experiments in which human participants solved a set of instances of one of three widely studied NP-Complete problems, namely the Traveling Salesperson, the Knapsack Problem or Boolean Satisfiability. Instances varied in their IC. We show that participants expended more effort on instances with higher IC, but that decision quality was lower in those instances. Together, our results suggest that IC can be used to measure the expected computational requirements of solving random instances of a problem, based on an instance’s features.

The findings of this study speak to the broader question of whether there is a link between the computation model in humans and electronic computers. Specifically, this study gives evidence that the average hardness of random instances can be characterized via the same set of parameters for both computing systems. This provides support that computational complexity theory applies to humans. Moreover, we argue that decision-makers could use IC to estimate the expected costs of performing a task. One reason is that the estimation of IC can be done without having to solve the problem. Furthermore, the results of this study suggest that IC captures the hardness of a random instance. Most importantly, our findings suggest that people modulate their effort according to IC. Altogether, this generates future avenues for research, based on IC, that could shed light into the cognitive resource allocation process in the brain.

## P141 A synthetic likelihood solution to the silent synapse estimation problem

### Michael Lynn^1^, Jean-Claude Beique^1^, Kevin Lee^1^, Cary Soares^1^, Richard Naud^2^

#### ^1^University of Ottawa, Cellular and Molecular Medicine, Ottawa, Canada; ^2^University of Ottawa, Cellular and Molecular Medicine; Department of Physics, Ottawa, Canada

##### **Correspondence:** Michael Lynn (mlynn101@uottawa.ca)

*BMC Neuroscience* 2020, **21(Suppl 1)**:P141

Functional features of populations of synapses are typically inferred from random electrophysiological sampling of small subsets of synapses. Are these samples unbiased? Here, we developed a biophysically constrained statistical framework for addressing this question and applied it to assess the performance of a widely used method based on a failure-rate analysis to quantify the occurrence of silent (AMPAR- lacking) synapses in neural networks. We simulated this method in silico and found that it is characterized by strong and systematic biases, poor reliability and weak statistical power. Key conclusions were validated by whole-cell recordings from hippocampal neurons. To address these shortcomings, we developed a simulator of the experimental protocol and used it to compute a synthetic likelihood. By maximizing the likelihood, we inferred silent synapse fraction with no bias, low variance and superior statistical power over alternatives. Together, this generalizable approach highlights how a simulator of experimental methodologies can substantially improve the estimation of physiological properties.

## P142 Using reinforcement learning to train biophysically detailed models of visual-motor cortex to play Atari games

### Haroon Anwar^1^, Salvador Dura-Bernal^1^, Cliff C Kerr^2^, George L Chadderdon^3^, William W Lytton^4^, Peter Lakatos^1^, Samuel A Neymotin^1^

#### ^1^Nathan Kline Institute for Psychiatric Research, Orangeburg, New York, United States of America; ^2^University of Sydney, School of Physics, Sydney, Australia; ^3^Burnet Institute, Melbourne, Australia; ^4^SUNY Downstate Medical Center, Department of Physiology and Pharmacology, Brooklyn, New York, United States of America

##### **Correspondence:** Haroon Anwar (haroon.anwar@nki.rfmh.org)

*BMC Neuroscience* 2020, **21(Suppl 1)**:P142

Computational neuroscientists build biophysically detailed models of neurons and neural circuits primarily to understand the origin of dynamics observed in experimental data. Much of these efforts are dedicated to match ensemble activity of the neurons in the modeled brain region while often ignoring multimodal information flow across brain regions and associated behaviors. Although these efforts have led us to improved mechanistic understanding of electrophysiological behavior of diverse types of neurons and neural networks, these approaches fall short of linking detailed models with associated behaviors in a closed-loop setting. In this study, we bridged that gap by developing biophysically detailed multimodal models of brain regions involved in processing visual information, generating motor behaviors and making associations between visual and motor neural representations by deploying reward-based learning mechanisms. We build a simple model of visual cortex receiving topological inputs from the interfaced Atari-game ‘pong’ environment (provided by the OpenAI’s Gym). This modeled region processed, integrated and relayed visual information about the game environment across the hierarchy of higher order visual areas (V1/V2 -> V4 -> IT). As we moved from V1 to IT, the number of neurons in each area decreased whereas the synaptic connections increased. This feature was included in the model to reflect the anatomical convergence suggested in the literature and to have a broader tuning for input features in progression up the visual cortical hierarchy. We used compartmental models of both excitatory and inhibitory neurons interconnected via AMPA (for excitation) or GABA (for inhibition) synapses. The strengths of synaptic connections were adjusted so that the information was reliably transmitted across visual areas. In our motor cortex model, neurons associated with a particular motor action were grouped together and received inputs from all visual areas. For the game Pong, we used two populations of motor neurons, for generating “up” and “down” move commands. All the synapses between visual and motor cortex were plastic, so that the connection strengths could be increased or decreased via reinforcement learning. When an action was generated in the model of motor cortex driven by visual representation of the environment in the model of visual cortex, that action generated a move in the game, which in turn updated the environment and triggered a response to the action: reward (+1), punishment (-1) or no-response (0). These signals drove the reinforcement learning at the synapses between visual cortex and motor cortex by strengthening or weakening them so that the model could learn which actions were rewarding in a given environment. Here we present an exploratory analysis as a proof-of-concept for using biophysically detailed modeling of neural circuits to solve problems that have so far only been tackled using artificial neural networks. We aim to use this framework to further simplify to make it more deep-learning-like and also to extend the architecture to make it biologically realistic. Comparing the performance of trained models using different architectures will allow us to dissect the mechanisms underlying production of behavior and will bridge the gap between the electrophysiological dynamics of neural circuits and associated behaviors.

## P143 Biophysically-detailed multiscale model of macaque auditory thalamocortical circuits reproduces physiological oscillations

### Salvador Dura-Bernal^1^, Erica Y Griffith^1^, Annamaria Barczak^2^, Monica N O’Connell^2^, Tammy McGinnis^2^, Haroon Anwar^3^, William W Lytton^1^, Peter Lakatos^3^, Samuel A Neymotin^3^

#### ^1^SUNY Downstate Medical Center, Department of Physiology and Pharmacology, Brooklyn, New York, United States of America; ^2^Nathan Kline Institute for Psychiatric Research, Center for Biomedical Imaging and Neuromodulation, Orangeburg, New York, United States of America; ^3^Nathan Kline Institute for Psychiatric Research, Orangeburg, New York, United States of America

##### **Correspondence:** Salvador Dura-Bernal (salvadordura@gmail.com)

*BMC Neuroscience* 2020, **21(Suppl 1)**:P143

We used the NEURON simulator with NetPyNE to develop a biophysically-detailed model of the macaque auditory thalamocortical system. We simulated a cortical column with a cortical depth of 2000um and 200um diameter, containing over 12k neurons and 30M synapses. Neuron densities, laminar locations, classes, morphology and biophysics, and connectivity at the long-range, local and dendritic scale were derived from published experimental data (Fig. 1). We used the model to investigate the mechanisms and function of neuronal oscillatory patterns observed in the auditory system in electrophysiological data recorded simultaneously from nonhuman primate primary auditory cortex (A1) and the medial geniculate body (MGB), while the awake subjects were presented with different classes of auditory stimuli, including speech.

**Fig. 1:**
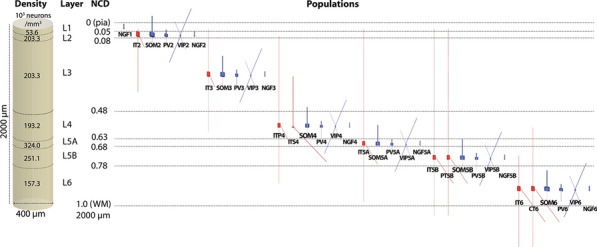
Dimensions of simulated A1 column with overall laminar cell densities, layer boundaries, cell morphologies and distribution of populations

The model A1 includes 6 cortical layers and multiple populations of neurons consisting of 4 excitatory (intratelencephalic (IT), spiny stellate (ITS), pyramidal-tract (PT), and corticothalamic (CT)), and 4 inhibitory types (somatostatin (SOM), parvalbumin (PV), vasoactive intestinal peptide (VIP), and neurogliaform (NGF)). Cells were distributed across layers 2-6, except NGF cells which were also included in L1, as these have been identified as important targets of the thalamic matrix. The A1 model was reciprocally connected to the thalamic model to mimic anatomically verified connectivity. The thalamic model included the medial geniculate body (MGB) and the thalamic reticular nucleus (TRN). MGB includes core and matrix populations of thalamocortical (TC) neurons with distinct projection patterns to different layers of A1, and thalamic interneurons (TI) projecting locally. TRN included thalamic reticular neurons (RE) primarily inhibiting MGB.

Thalamocortical neurons were driven by artificial spike generators simulating background inputs from non-modeled brain regions. Auditory stimulus related inputs were simulated using phenomenological models of the cochlear auditory nerve and the inferior colliculus (IC) that captured the main physiological transformations occurring in these regions. The output of the IC model was then used to drive the thalamocortical populations. This allowed us to provide any arbitrary sound as input to the model, including those used during our macaque in vivo experiments, thus facilitating matching model to data.

We used evolutionary algorithms to tune the network to generate experimentally-constrained firing rates for each of the 42 neural populations. We tuned 12 high-level connectivity parameters, including background input and E->E, E->I, I->E, I->I weight gains, within parameter value ranges constrained biologically. Each simulated second required approximately 1 hour on 96 supercomputer cores. For the evolutionary optimization we ran 100 simultaneous simulations (9,600 cores) every generation. To the best of our knowledge, this is the first time evolutionary optimization has been successfully used for large-scale biophysically-detailed network models.

We will use our model to determine mechanistic origins of spatiotemporal neuronal oscillatory patterns observed in vivo using an iterative modeling data-analysis process. At the end of the process, to confirm model predictions, we will use targeted deep brain electrical microstimulation and pharmacological manipulations.

**Acknowledgements:** Funded by NIH NIDCDR01DC012947, U24EB028998, U01EB017695, DOH01-C32250GG-3450000, Army Research Office W911NF-19-1-0402.

## P144 Closed loop parameter estimation using GPU acceleration with GeNN

### Felix B Kern^1^, Michael Crossley^2^, Rafael Levi^3^, György Kemenes^2^, Thomas Nowotny^4^

#### ^1^University of Tokyo, International Research Center for Neurointelligence, Tokyo, Japan; ^2^University of Sussex, School of Life Sciences, Brighton, United Kingdom; ^3^Universidad Autonoma de Madrid, Escuela Politechnica Superior, Madrid, Spain; ^4^University of Sussex, School of Engineering and Informatics, Brighton, United Kingdom

##### **Correspondence:** Thomas Nowotny (t.nowotny@sussex.ac.uk)

*BMC Neuroscience* 2020, **21(Suppl 1)**:P144

A common approach to understanding neuronal function is to build accurate and predictive models of the excitable membrane. Models are typically based on voltage clamp data where ion channels of different types are pharmacologically isolated and the stationary state and timescale of (in)activation are estimated based on the transmembrane currents observed in response to a set of constant voltage steps. The basic method can be extended with different stepping protocols or input waveforms and by performing parameter fits on the full time series. Further improvements are achieved with parameter estimation on additional current clamp data, an active field of research. Some examples of employed estimation approaches include adaptive coupling to synchronise the model to data, driving neurons with chaotic input signals, and using distributions of parameter values in a path integral method.

Enabled by our GPU enhanced neural networks (GeNN) framework [1], we here present work that makes a different conceptual advance of performing parameter estimation in an online closed loop approach while the neuron is being recorded. In doing so we can select stimulations that are highly informative for the parameter estimation process at any given time. We can also track time dependent parameters by observing how parameter estimates develop over time.

To demonstrate our new method we use the model system of the B1 motor cell in the buccal ganglion of the pond snail *Lymnaea stagnalis*. Neurons are recorded with two sharp electrodes in current clamp mode. We have built a conductance based initial model from a published set of Hodgkin-Huxley conductances [2], using standard parameter estimation methods and data we obtained with a simple set of current steps. To perform closed loop parameter estimation, we use a genetic algorithm (GA) in which a population of 8192 model neurons with candidate parameter values is simulated on a GPU (NVIDIA Tesla K40c) in parallel and in real time. Models are then compared to the response of the recorded neuron and selected for goodness of fit, as is standard for a GA approach. The novel element of our method is the next step, where we evaluate a pool of candidate stimuli against the model population, selecting the stimulus with the most diverse responses for the next epoch. The selected stimulus is then applied to both the recorded neuron and the population of models and the normal GA procedure continues.

Figure 1 shows a representative example of online fitting to a neuron. We first fit a set of 52 parameters to fine-tune model kinetics to the cell under stationary conditions. Then, we restricted fitting to non-kinetic parameters (maximum conductances, equilibrium potentials, and capacitance) and continued to run the algorithm described above, while at the same time manipulating sodium levels in the extracellular bath. The online fitting procedure can detect and track the change in sodium concentration as putative changes in sodium conductance and reversal potential.

**Fig. 1:**
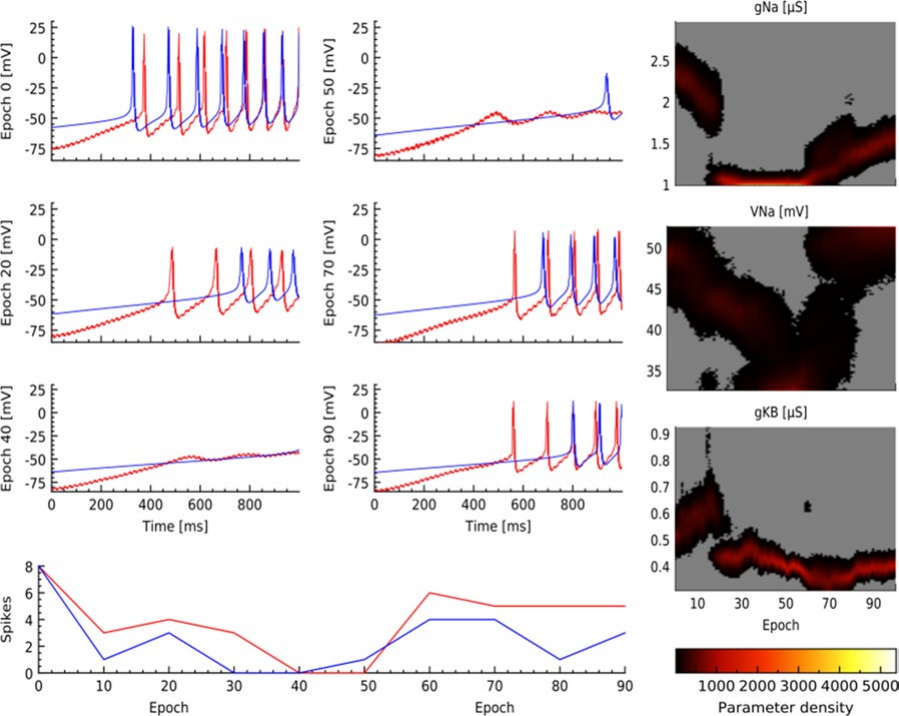
Example fitting run. Sodium-free saline is washed in (epochs 1-50), and gradually removed (51-100). Left: Voltage of the neuron (red) and best model (blue) in response to a ramp stimulus. Bottom: Spike counts with the same stimulus. Right: Value distributions of some of the fitted parameters (y axis) through time (x axis). The model recapitulates the change in sodium (top 2 plots)

**Acknowledgments:** This work was partially supported by the EU under Grant Agreement 785907 (HBP SGA2).

**References**

1. Yavuz E, Turner JP, Nowotny T. GeNN: a code generation framework for accelerated brain simulations. Scientific Reports. 2016; 6: 18854.
2. Vehovszky A, Szabo H, Elliott CJH. Octopamine increases the excitability of neurons in the snail feeding system by modulation of inward sodium current but not outward potassium currents. BMC Neuroscience. 2005; 6: 70.

## P145 Possible roles of non-synaptic interactions between olfactory receptor neurons in insects

### Mario Pannunzi^1^, Thomas Nowotny^2^

#### ^1^University of Sussex, Department of Informatics, Brighton, United Kingdom; ^2^University of Sussex, School of Engineering and Informatics, Brighton, United Kingdom

##### **Correspondence:** Mario Pannunzi (mario.pannunzi@gmail.com)

*BMC Neuroscience* 2020, **21(Suppl 1)**:P145

In insects, olfactory receptor neurons (ORNs) are grouped in hairs (sensilla) in a stereotypical way. For example, in *Drosophila* each sensillum houses 2 or 4 ORNs. ORNs that are co-housed in the same sensillum interact with each other via a non-synaptic mechanism (NSI, see Fig. 1a), a mechanism which is still not fully understood. The mechanism could simply be a spandrel or instead improve the function of the insect olfactory system. A number of hypotheses have been suggested [1] trying to explain the potential role of NSIs.

**Fig. 1:**
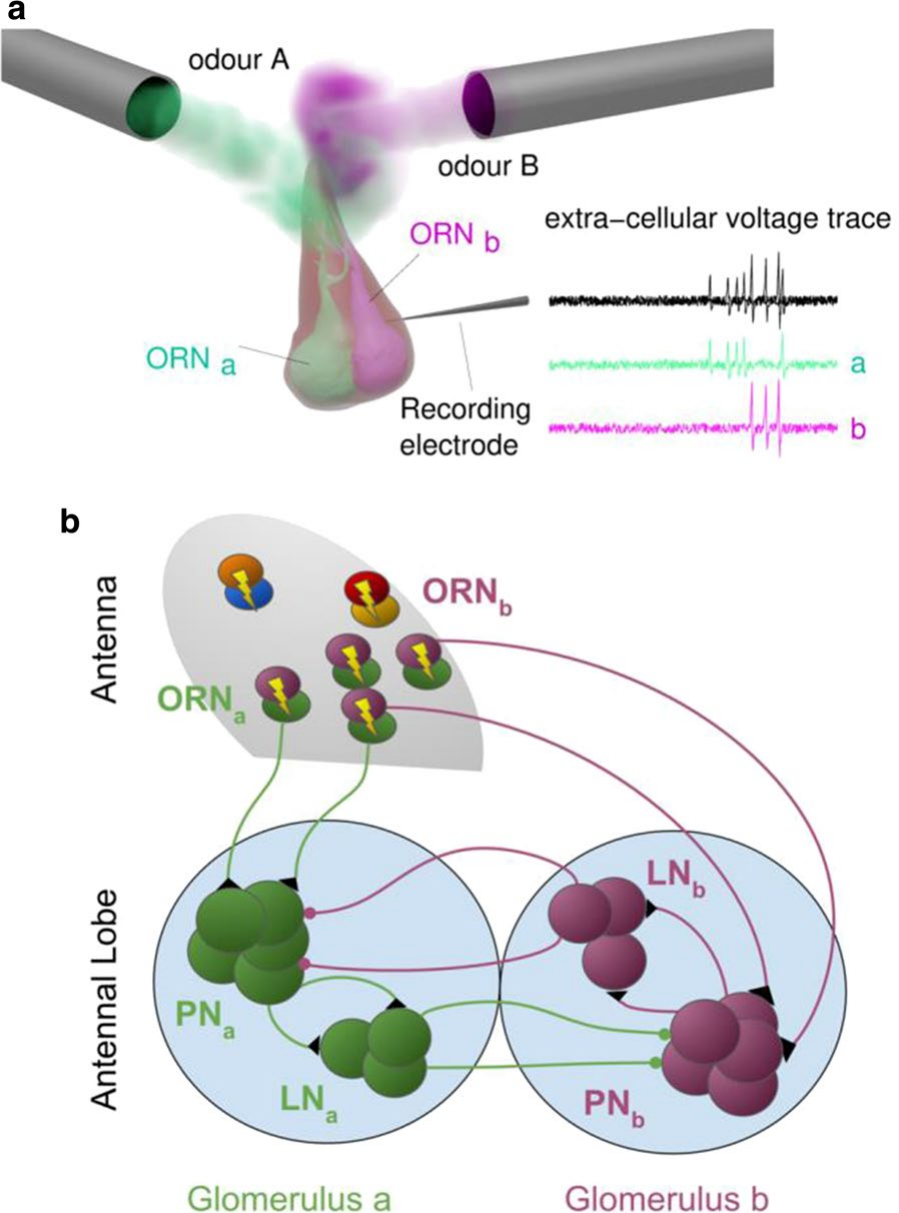
**a** Non-synaptic interaction (NSI) between ORNs is probably mediated by an electrical field interaction between closely apposed ORNs in olfactory sensilla. **b** Our model: two ORN types and their respective PNs and LNs, in the AL. Each ORN type is tuned to a set of odorants and converges onto its corresponding PNs. Each PN excites its respective LNs, and is inhibited from LNs of the other type

We analyzed two hypotheses that suggest that NSIs play a role in odor sensing in mixtures with a computational model of the first two layers of the *Drosophila* olfactory system - the ORNs on the antennae and the glomeruli, with projection neurons (PNs) and local neurons (LNs), in the antennal lobe (AL, see Fig. 1b). The model is the first to consider NSIs between ORNs in the context of the circuits of the first and second layer of processing in the insect olfactory pathway. We constrained the model by reproducing the responses to a set of typical odor stimuli reported in the literature. Then, we tested the feasibility of the hypotheses and compared the advantages of having NSIs against a control network which lacked any interaction between ORNs or PNs, and with a network without NSIs but strong lateral inhibition in the AL, a mechanism proposed to be a valid alternative to NSIs.

The two tested hypotheses were: 1) NSIs could improve the concentration ratio identification of a mixture of odorants by increasing the dynamic range over which it can be perceived without distortion. 2) NSIs could help insects to distinguish mixtures of odorants emanating from a single source against those emanating from two separate sources, by improving the capacity to encode the correlation between olfactory stimuli.

For the first hypothesis, we observed that: 1) When comparing the capacity to encode the ratio of the concentration of short synchronous whiffs via PN responses, both networks, the one with NSIs and the one with AL inhibition, outperform the ‘control network’. Moreover, the NSIs help more than the LN inhibition for this task. This effect is stronger for very short stimuli (<100ms) than for longer stimuli. 2) When a network with LN inhibition (with NSIs) is stimulated with asynchronous whiffs of two odorants, its PN outputs in response to the second whiffs are strongly (mildly) altered by the response to the first whiff.

More complex interpretations are needed when assessing the capacity to encode the correlation between two odorants. We noted that: 1) In terms of average PN activity, the network with LN inhibition is able to encode stimulus correlation but the network with NSI mechanism is not, but 2) In terms of peak PN activity (response higher than a given threshold), the network with NSI mechanism encodes correlations better than the one with LN inhibition. This improvement is bigger for shorter whiff durations (<100 ms).

**Acknowledgments:** This research was funded by the Human Frontiers Science Program, grant RGP0053/2015 (Odor Objects), the European Union under grant agreement 785907 (HBP SGA2) and a Leverhulme Trust Research Project Grant.

**Reference**

1. Todd JL, Baker TC. Function of peripheral olfactory organs. In: Insect olfaction 1999 (pp. 67-96). Springer, Berlin, Heidelberg.

## P146 Larger GPU-accelerated brain simulations with procedural connectivity

### James Knight^1^, Thomas Nowotny^2^

#### ^1^University of Sussex, Department of Informatics, Brighton, United Kingdom; ^2^University of Sussex, School of Engineering and Informatics, Brighton, United Kingdom

##### **Correspondence:** James Knight (j.c.knight@sussex.ac.uk)

*BMC Neuroscience* 2020, **21(Suppl 1)**:P146

Large-scale brain simulations are important tools for investigating brains’ dynamics and function. However, due to insufficient computing power and a lack of detailed connectivity data, brain models built at the cellular level have often been limited to the scale of individual microcircuits [1]. Larger models of multiple areas have typically been built at a higher level of abstraction. However, recent data [2] showed that, in the cortex, there are features of neuronal activity which can only be reproduced by modelling multiple interconnected microcircuits at the cellular level.

Even small mammals have trillions of synapses and as each synapse typically requires at least 32 bits of storage in a simulation, a model of this scale requires terabytes of memory – more than any single desktop machine has available. Therefore, until now, simulating large-scale models has required access to a distributed computer system. Such systems are costly and power-hungry, meaning that they are normally shared resources, accessible only to a limited number of well-funded researchers for limited runtimes. Neuromorphic systems are a potential alternative but few are currently able to simulate the density of connectivity found in the brain and most are still prototypes with limited availability. Alternatively, Graphical Processing Units (GPUs) have proved useful in tasks including training deep learning systems. In our previous work [3] we showed that using GeNN – our GPU accelerated spiking neural network simulator – models with around 100×103 neurons and 1×109 synapses could be simulated on a single GPU at a similar speed to supercomputers and neuromorphic systems. However, individual GPUs do not have enough memory to simulate larger brain models and GPU clusters suffer from the same issues as any other distributed computer systems.

Here, we present extensions to GeNN that enable it to ‘procedurally’ generate connectivity and synaptic weights ‘on the go’ as spikes are triggered instead of retrieving them from memory. This approach is well-suited to GPU architectures because their raw computational power is often under-utilised when simulating spiking neural networks due to memory bandwidth limitations. We demonstrate the power of our approach with a model of the Macaque visual cortex consisting of 4×106 neurons and 24×109 synapses [4]. We find that, with our new method, this model can be simulated correctly on a single GPU and up to 35% faster than supercomputer simulations [5]. We believe that this is a significant step towards making large-scale brain modelling accessible to more researchers.

**Fig. 1:**
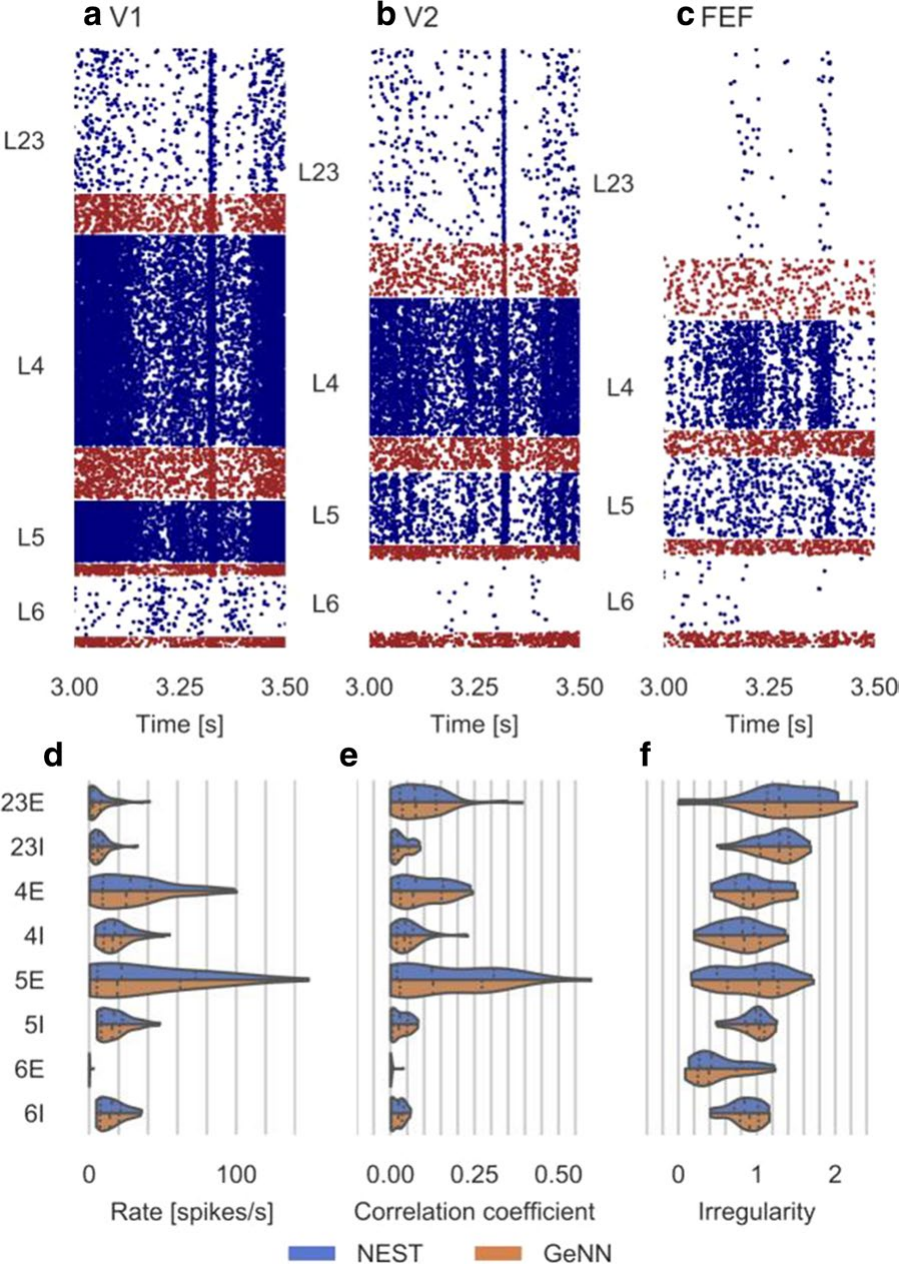
Results of macaque visual cortex model simulation. **A-C** Raster plots of activity of 3% of neurons in 3 areas. Blue: excitatory, red: inhibitory. **D-F** Statistics for each population across all 32 areas simulated using GeNN and NEST (supercomputer). **D** Average firing rates. **E** Average pairwise correlation coefficients. **F** Average irregularity measured by revised local variation

**Acknowledgements:** This work was funded by the EPSRC (Grant number EP/P006094/1).

**References**

1. Potjans T, Diesmann M. The Cell-Type Specific Cortical Microcircuit: Relating Structure and Activity in a Full-Scale Spiking Network Model. Cerebral Cortex. 2014; 24: 785-806.
2. Belitski A, et al. Low-frequency local field potentials and spikes in primary visual cortex convey independent visual information. Journal of Neuroscience. 2008; 28(22): 5696-709.
3. Knight J, Nowotny T. GPUs Outperform Current HPC and Neuromorphic Solutions in Terms of Speed and Energy When Simulating a Highly-Connected Cortical Model. Frontiers in Neuroscience. 2018; 12: 1-19.
4. Schmidt M, et al. A multi-scale layer-resolved spiking network model of resting-state dynamics in macaque visual cortical areas. PLoS Computational Biology. 2018; 14(10): e1006359.
5. Knight J, Nowotny T. Larger GPU-accelerated brain simulations with procedural connectivity. BioRxiv. 2020. 2020.04.27.063693

## P147 Seizure forecasting from long-term EEG and ECG data using critical slowing principle

### Wendy Xiong^1^, Tatiana Kameneva^2^, Elisabeth Lambert^3^, Ewan Nurse^4^

#### ^1^Swinburne University of Technology, Melbourne, Australia; ^2^Swinburne University of Technology, Telecommunication Electrical Robotics and Biomedical Engineering, Melbourne, Australia; ^3^Swinburne University of Technology, School of Health Sciences, Department of Health and Medical Sciences, Melbourne, Australia; ^4^Seer Medical, Melbourne, Australia

##### **Correspondence:** Wendy Xiong (wenjuan.xiong1@gmail.com)

*BMC Neuroscience* 2020, **21(Suppl 1)**:P147

Epilepsy is a neurological disorder characterized by recurrent seizures that are transient symptoms of synchronous neuronal activity in the brain. Epilepsy affects more than 50 million people worldwide [1]. In Australia, over 225,000 people live with epilepsy [2]. Seizure prediction allows patients and caregivers to deliver early interventions and prevent serious injuries. Electroencephalography (EEG) has been used to predict seizure onset, with varying success between participants [3,4]. There is an increasing interest to use electrocardiogram (ECG) to help with seizures detection and prediction. The aim of this study is to use long-term continuous recordings of EEG and ECG data to forecast seizures.

EEG and ECG data from 7 patients was used for analysis. Data was recorded using 21 EEG electrodes and 3 ECG electrodes by Seer with an ambulatory video-EEG-ECG system. The average period of recording was 95 hours (range 51-160 hours). Data was annotated by a clinician to indicate seizure onset and offset. On average, 4 clinical seizures occurred per participant (range 2-10). EEG and ECG data were bandpass filtered using Butterworth filter (1-30 Hz for EEG, 3-45 Hz for ECG).

A characteristic of a system that is nearing a critical transition is critical slowing, which refers to the tendency of the system to take longer to return to equilibrium after perturbations, measured by an increase in signal variance and autocorrelation [5]. The variance and autocorrelation of EEG and ECG signals were calculated for each electrode in 1 s window for each time point. The autocorrelation value was set to the width of half maximum of the autocorrelation function. The instantaneous phases of variance and autocorrelation signals were calculated at each time point using Hilbert transform. To extract long (1 day) and short (20 s in EEG, 10 min in ECG) cycles in the variance and autocorrelation signals, a moving average filter has been applied. The relationship between seizure onset times and phase of variances and autocorrelation were investigated in long and short cycles. The probability distribution for seizure occurrence was determined for each time point. The seizure likelihood was determined at three levels: low, medium and high, based on two thresholds defined as functions of maximum seizure probability. Data analysis was performed in Python 3.

Results show that the variance and autocorrelation of EEG data increased at the time of seizure onset in 66.7% and 68.3% of cases, respectively. The variance and autocorrelation of ECG data increased at the time of seizure onset in 60% and 50% cases, respectively. Long and short cycles of variance and autocorrelation had consistent results. Result indicate that critical slowing may be present in a neural system during seizures and this feature could be used to forecast seizures.

**References**

1. Thijs RD, Surges R, O’Brien TJ, Sander JW. Epilepsy in adults. The Lancet. 2019; 393(10172): 689-701.
2. Epilepsy Action Australia [internet]. Facts and Statistics [cited 2020 July 8]. Available from: www.epilepsy.org.au.
3. Cook MJ, et al. Prediction of seizure likelihood with a long-term, implanted seizure advisory system in patients with drug-resistant epilepsy: a first-in-man study. The Lancet Neurology. 2013; 12(6): 563-71.
4. Karoly PJ, Ung H, Grayden DB, Kuhlmann L, Leyde K, Cook MJ, Freestone DR. The circadian profile of epilepsy improves seizure forecasting. Brain. 2017; 140(8): 2169-82.
5. Scheffer M, et al. Early-warning signals for critical transitions. Nature. 2009; 461(7260): 53-9.

## P148 Biophysically grounded mean-field models of neural populations under electrical stimulation

### Caglar Cakan^1^, Klaus Obermayer^2^

#### ^1^Technische Universität Berlin, Berlin, Germany; ^2^Technische Universität Berlin, Department of Software Engineering and Theoretical Computer Science, Berlin, Germany

##### **Correspondence:** Caglar Cakan (cakan@ni.tu-berlin.de)

*BMC Neuroscience* 2020, **21(Suppl 1)**:P148

Electrical stimulation of neural systems is a key tool for understanding neural dynamics and ultimately for developing clinical treatments. Many applications of electrical stimulation affect large populations of neurons. However, computational models of large networks of spiking neurons are inherently hard to simulate and analyze. We evaluate a reduced mean-field model of excitatory and inhibitory adaptive exponential integrate-and-fire (AdEx) neurons which can be used to efficiently study the effects of electrical stimulation on large neural populations. The rich dynamical properties of this basic cortical model are described in detail and validated using large network simulations. Bifurcation diagrams (Fig. 1) reflecting the network’s state reveal asynchronous up and down-states, bistable regimes, and oscillatory regions corresponding to fast excitation-inhibition and slow excitation-adaptation feedback loops. The biophysical parameters of the AdEx neuron can be coupled to an electric field with realistic field strengths which then can be propagated up to the population description. We show how on the edge of bifurcation, direct electrical inputs cause network state transitions, such as turning on and off oscillations of the population rate. Oscillatory input can frequency-entrain and phase-lock endogenous oscillations. Relatively weak electric field strengths on the order of 1 V/m are able to produce these effects, indicating that field effects are strongly amplified in the network. The effects of time-varying external stimulation are well predicted by the mean-field model, further underpinning the utility of low-dimensional neural mass models.

**Fig. 1:**
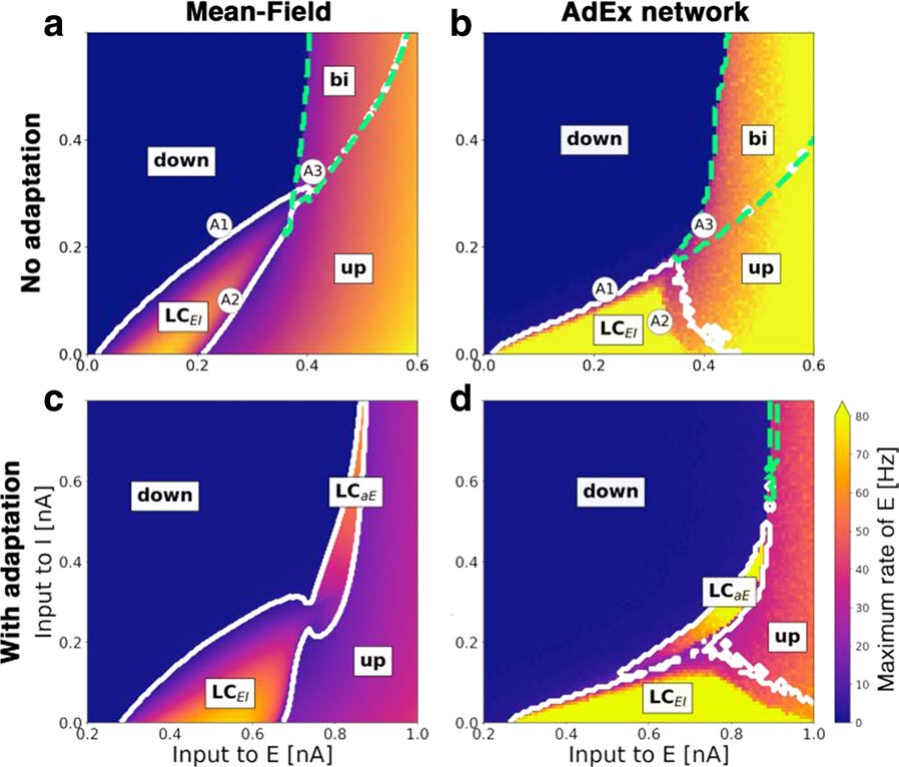
Bifurcation diagrams depict the state space of the E-I system in terms of the mean external inputs. **a-b** Mean-field model and AdEx network without adaptation with up and down-states, a bistable region bi (green dashed contour) and an oscillatory region LC_EI_ (white solid contour). **c-d** With somatic adaptation. The bistable region is replaced by a slow oscillatory region LC_aE_

## P149 Towards large-scale model of sleeping brain: sleep spindles in network mass models

### Nikola Jajcay, Klaus Obermayer

#### Technische Universitat Berlin, Neural Information Processing Group, Berlin, Germany

##### **Correspondence:** Nikola Jajcay (jajcay@ni.tu-berlin.de)

*BMC Neuroscience* 2020, **21(Suppl 1)**:P149

The hierarchical nesting of sleep spindles and cortical slow oscillations is considered a precursor of successful episodic memory consolidation where it, presumably, sets the stage for memory traces migration from short-term hippocampal storage to longer-lasting neocortical sites [1]. Spindles are thought to be generated in the thalamus and projected onto cortical sites, while slow oscillations originate in the cortex and migrate in wave-like patterns to the thalamus, hence the interplay between the two rhythms is orchestrated within the thalamocortical circuitry. The state-of-the-art mass models of the thalamocortical loop, however, only consider relevant motifs and patterns in sleep but discard network effects. Here we take the first steps towards integrating thalamocortical projections into a large-scale brain model and modelling whole-brain cortical slow-wave activity and thalamic spindles as seen in non-REM sleep.

We model the thalamus as a network node containing one excitatory and one inhibitory mass representing thalamocortical relay neurons and thalamic reticular nuclei, respectively. With little deviations, our model follows the thalamic component developed in [2]. In the thalamic submodule, we investigated its spindling behaviour upon changing, firstly, conductances of rectifying and T-type calcium current, by which the thalamus can be parametrised in three oscillatory regimes: fast oscillations, dominated by Ca current; spindle regimes with a balanced interplay of Ca and rectifying currents, and slow delta oscillations for strong hyperpolarisation. Next, by the application of external excitatory firing rate drive, which simulates excitatory source connected to the thalamus, we found dynamically interesting spindle-promoting regimes in interaction with thalamic conductances (see Fig. 1 for estimated number of spindles), and by changing the parameters or external drive, we were also able to control the inter-spindle interval, spindle duration, and shape of spindle envelope.

**Fig. 1:**
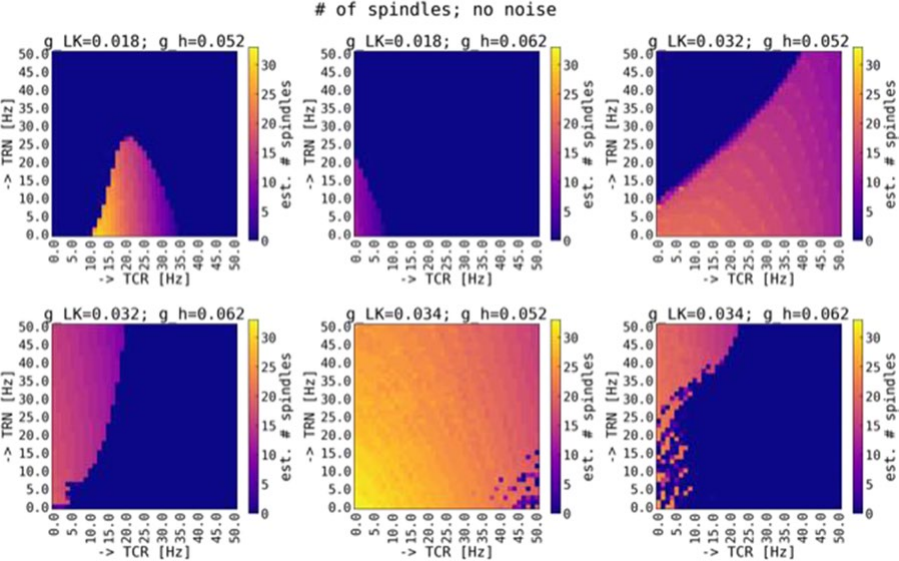
Various oscillatory regimes for isolated thalamus. Each panel shows estimated number of spindles (color-coded) in 60 seconds-long simulation dependent on excitatory rate drive to thalamocortical relay mass (TCR) and thalamic reticular nuclei (TRN). Different panels encode different conductances for potassium leak current (gLK) and rectifying current (gh)

Secondly, we connected the thalamic node to one cortical node, modelled as interconnected excitatory and inhibitory adaptive exponential integrate-and-fire neuronal masses [3]. The excitatory mass contains a spike-triggered adaptation mechanism by which the node is parametrised to sit in the limit cycle generating slow oscillations by the means of excitation-adaptation feedback loop. We investigated the dynamical repertoire of the connected model concerning the connection strength and network delays. Our preliminary results indeed show that, upon connection, the thalamic spindles are imprinted into cortical node activity

where they modulate slow oscillation envelope, and that slow oscillation activity of the cortex, in turn, shapes spindling behaviour of thalamocortical relay mass by affecting spindle duration and inter-spindle interval. Our connected model also conserves the phase-phase and phase-amplitude couplings reported in the literature on observed EEG data or other thalamocortical models.

Our results suggest that thalamic mass model of spindle activity can be connected to various mass or mean-field models of cortical nodes and, after careful treatment of network connections and delays, we believe that our conclusions would carry over to the large-scale network model.

**References**

1. Ji D, Wilson MA. Coordinated memory replay in the visual cortex and hippocampus during sleep. Nature neuroscience. 2007; 10(1): 100-7.
2. Schellenberger Costa M, et al. A thalamocortical neural mass model of the EEG during NREM sleep and its response to auditory stimulation. PLoS Computational Biology. 2016; 12(9): e1005022.
3. Cakan C, Obermayer K. Biophysically grounded mean-field models of neural populations under electrical stimulation. PLoS Computational Biology. 2020; 16(4):ce1007822.

## P150 The cortical ignition is related to local and mesoscale features of structural connectivity in the connectome of non-human organisms

### Samy Castro, Patricio Orio

#### Universidad de Valparaiso, Centro Interdisciplinario de Neurociencia de Valparaíso, Valparaíso, Chile

##### **Correspondence:** Samy Castro (samy.castro@cinv.cl)

*BMC Neuroscience* 2020, **21(Suppl 1)**:P150

One way to study the fluctuations of cortical activity is the ignition, i.e. the fast transition from low to high firing rate on cortical regions. The capability of cortical regions to flexibly sustain an ‘ignited’ state of activity has been related to conscious perception and hierarchical information processing. Also, we theoretically showed that the propensity of cortical regions to be ignited is tightly linked to the core-shell structure of the human connectome, i.e. the shells of strongest within-connected subsets of regions [1]. Moreover, the weight of connections (in particular the *inputs*) has the greatest influence in the propensity of a region to get ignited. Now, using the connectomes of non-human organisms (macaque [2], mouse [3], rat [4] and fruit fly [5]), we assessed whether the relationship between ignition and both local and mesoscale structural organization is maintained in the related organisms. The ignition capabilities of each connectome are obtained from the whole-brain mean-field model, using simulations of the resting-state cortical activity. Then, the structural organization is analyzed using thes-core decomposition for the mesoscale level, and the degree, betweenness centrality and participation index for the local level. The order in which cortical regions are ignited is correlated to both thes-core and the strength of the regions (i.e. hubs) of the different organisms (Fig. 1). Moreover, we found that ignition recruitment is primarily related to weights of the inputs, rather than the outputs, of each region. The local level better explains the region propensity to get ignited in the case of macaque and rat, whereas the mesoscale fits better in the case of the fruit fly and mouse. We suggest that the weighted organization of non-human connectomes, as in the human, operates as a structural principle of ignition rooted in evolution.

**Fig. 1:**
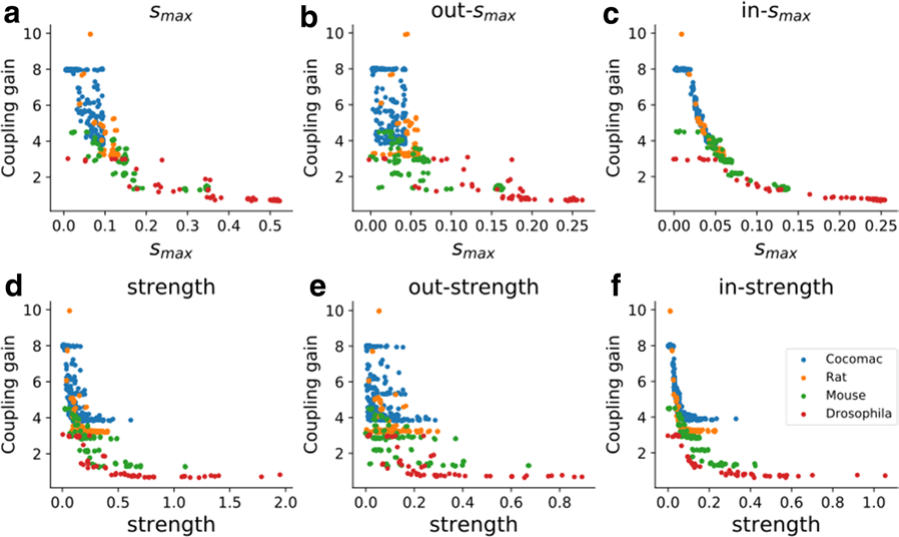
Ignition recruitment is related to input weights in the local and mesoscale level. **A-F** The scatter plots of the coupling gain of ignition (x-axis) and the mesoscale level, **A** smax, **B** out-smax, and **C** in-smax (y-axis), and local level, **D** strength, **E** out-strength, and **F** in-strength of each region of the cocomac (blue), rat (orange), mouse (green) and drosophila (red) dataset

**Acknowledgements:** This work was supported by Fondecyt Grant 1181076 (PO) and the Advanced Center for Electrical and Electronic Engineering (FB0008 CONICYT, Chile)(PO, SC). The Centro Interdisciplinario de Neurociencia de Valparaíso (CINV) is a Millenium Institute supported by the Millennium Scientific Initiative of the Ministerio de Economía (Chile). SC was funded by Beca Doctorado Nacional CONICYT 21140603 and by Programa de Doctorado en Ciencias, mención Neurociencia, Universidad de Valparaíso.

**References**

1. Castro S, El-Deredy W, Battaglia D, Orio P. Cortical ignition dynamics is tightly linked to the core organisation of the human connectome. bioRxiv. 2020.
2. Bakker R, Wachtler T, Diesmann M. CoCoMac 2.0 and the future of tract-tracing databases. Frontiers in Neuroinformatics. 2012 Dec; 6: 30.
3. Rubinov M, Ypma R, Watson C, and Bullmore E. Wiring Cost and Topological Participation of the Mouse Brain Connectome. PNAS. 2015, 112(32): 10032–10037.
4. Bota M, Sporns O, and Swanson L. Architecture of the Cerebral Cortical Association Connectome Underlying Cognition. PNAS. 2015, 112(16): E2093–2101.
5. Shih CT, et al. Connectomics-based analysis of information flow in the Drosophila brain. Current Biology. 2015; 25(10): 1249-58.

## P151 Generalisation of stimulus representation across somatosensory cortex areas in a cellular-resolution photostimulus detection task

### Thijs L van der Plas, James M Rowland, Robert M Lees, Adam M Packer

#### University of Oxford, Department of Physiology, Anatomy, and Genetics, Oxford, United Kingdom

##### **Correspondence:** Thijs L van der Plas (thijs.vanderplas@dtc.ox.ac.uk)

*BMC Neuroscience* 2020, **21(Suppl 1)**:P151

Mice use whiskers to explore their environment. Whisker stimulation elicits a neural response in primary (S1) and secondary (S2) somatosensory cortex, two highly interconnected and hierarchically organised brain regions. Their interaction has been related to stimulus detection [1], although its precise functional role remains unclear [2]. Here, we aim to assign a function to this circuit for a stimulus detection task, by assessing how S1-S2 interactions facilitate stimulus perception.

We have conditioned mice to detect 2-photon optogenetic stimulation of random ensembles of S1 cells. This allows us to control the number of stimulated cells on a trial by trial basis, and to separate the initial stimulus representation from the ensuing network response. Simultaneously, we record the calcium activity of both stimulated and unstimulated cells in S1 and S2, rendering an all-optical approach to study neural dynamics [3]. In short, we are able to directly stimulate S1 neurons, hence defining the initial stimulus in S1, while recording the subsequent S1 and S2 neural response.

Mice were conditioned to report the photostimulus by licking a water spout. The task was divided into Go trials, where a varying number (5 - 150) of cells were stimulated, and Catch trials without stimulation. Behavioural accuracy increased as more cells were stimulated (Fig. 1A), indicating that our task operated in the regime of perception.

We observed strongly elevated, sustained neural population activity in both S1 and S2 on successful Go trials (Hits), compared to both unsuccessful Go trials (Misses) and to licking behaviour in the absence of a stimulus (False Positives). This suggests that S1 and S2 encode information during Hit trials that is different from both passive stimulus-induced activity and neural signals driven by movement and reward.

To confirm whether neurons indeed encoded stimulus information, we performed a stimulus decoding analysis on S1 and S2 neural activity separately. We only consider trials where mice licked (i.e. Hits and False Positives), to avoid a behavioural bias. Here, we observe a significant difference between S1 (where the stimulus occurred) and S2 (Fig. 1B): Stimulus information could only be decoded from S2 after a considerable time post-stimulus, while S1 could be decoded directly post-stimulus. Hence, stimulus information has propagated (directly or indirectly) from S1 to S2.

Furthermore, we find a striking dynamic property of information coding in the S1-S2 circuit. Directly post-stimulus at 1s, decoding accuracy in S1 depends on the stimulus strength, the number of stimulated cells (Fig. 1C). However, after a delay of 3s, we find that accuracy has increased, and has become independent of the original stimulus strength (Fig. 1C). S2 decoding accuracy increased equivalently (not shown), even though S2 decoding performs at chance level directly post-stimulus (Fig. 1B).

The stimulus detection task design requires the animals to elicit the same response, independent of stimulus strength. Our results show that the S1-S2 circuit dynamically performs this computation: by propagating stimulus information between S1 and S2, the neural code becomes independent of the original stimulus strength. Hence, we uncover a putative mechanism of how interregional communication can transform stimulus information to facilitate stimulus detection.

**Fig. 1:**
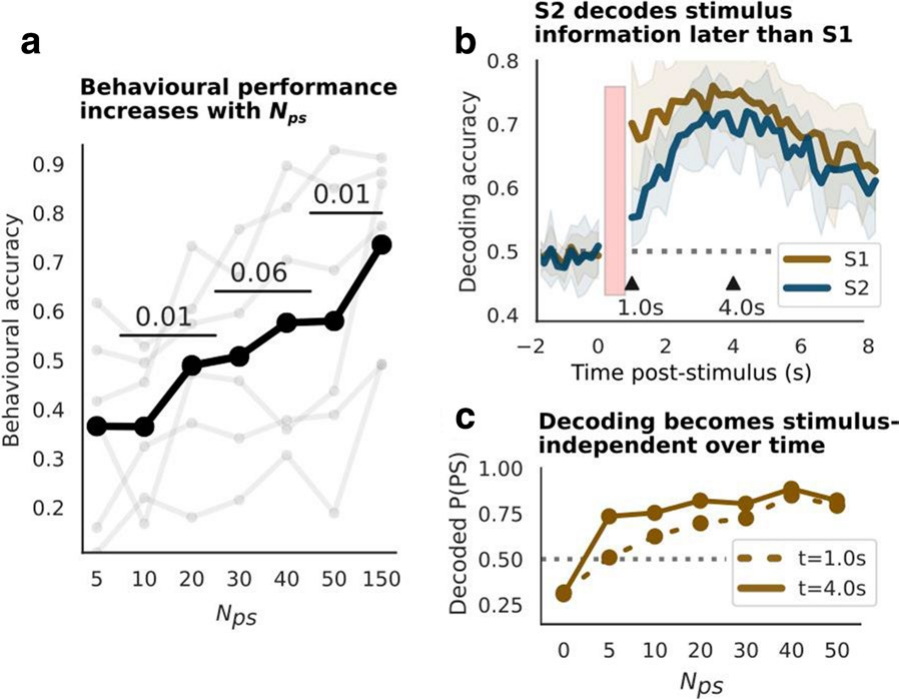
**A** Behavioural accuracy (fraction of correct trials) is plotted against the number of photostimulated (PS) neurons Nps (P-values, Wilcoxon). **B** Decoding accuracy was calculated per time point with logistic regression. Shaded areas indicate std. across 6 mice. Red box indicates the 2-photon stimulus. **C** Decoded probability of PS in S1 for 2 time points (indicated by triangles in B), split by Nps

**References**

1. Kwon SE, Yang H, Minamisawa G, O’Connor DH. Sensory and decision-related activity propagate in a cortical feedback loop during touch perception. Nature neuroscience. 2016; 19(9): 1243-9.
2. Ni J, Chen JL. Long‐range cortical dynamics: a perspective from the mouse sensorimotor whisker system. European Journal of Neuroscience. 2017; 46(8): 2315-24.
3. Packer AM, Russell LE, Dalgleish HW, Häusser M. Simultaneous all-optical manipulation and recording of neural circuit activity with cellular resolution in vivo. Nature methods. 2015; 12(2): 140-6.

## P152 Face-selective neurons arise spontaneously in untrained deep neural networks

### Min Song^1^, Seungdae Baek^1^, Jaeson Jang^1^, Gwangsu Kim^2^, Se-Bum Paik^1^

#### ^1^Korea Advanced Institute of Science and Technology, Department of Bio and Brain Engineering, Daejeon, South Korea; ^2^Korea Advanced Institute of Science and Technology, Department of Physics, Daejeon, South Korea

##### **Correspondence:** Min Song (night@kaist.ac.kr)

*BMC Neuroscience* 2020, **21(Suppl 1)**:P152

In the primate brain, the neurons that selectively respond to faces are observed and considered as the basis of face recognition [1]. Although such face-selective neurons are observed in infant animals [2], the origin of face-selectivity is still under debate, because conflicting findings have raised questions whether this neuronal selectivity can arise spontaneously [3], or requires training from visual experience [2]. Here, we show that face-selective neurons can spontaneously arise in untrained deep neural networks (DNN), together with the previous notion that DNN could be considered as a visual cortex model that can perform human-level visual function and predict neuronal responses. Using biologically-inspired neural networks, AlexNet, we measured responses of the last convolutional layer to the image sets of face and 15 non-face classes. We found that face-selective neurons arise in untrained AlexNet with randomly permuted weights, where the face-selective neuron was defined as a neuron that showed a significantly higher response to face images compared to non-face images. To qualitatively examine the feature-selective response of these face-selective neurons, we reconstructed the preferred feature images of individual neurons using the reverse correlation method. We found face-components, such as eyes, nose, and mouth, in preferred feature images of face-selective neurons whereas no noticeable shape was found in neurons with no selectivity. Next, to test whether the selective response of these neurons could provide sufficient information to classify a face from other objects, we trained a support vector machine (SVM) to classify whether the given image was a face using neural responses of the untrained network. As a result, the SVM trained with only face-selective neurons shows significantly better performance than that trained with neurons with no selectivity. Next, to examine whether the face-selective neurons show view-point invariant characteristics observed in monkeys, we measured the responses of the permuted AlexNet while face images from five different angles were provided to the network. Surprisingly, the face-selective neurons in the network show viewpoint invariant responses and their level of invariance increased along the network hierarchy in the permuted AlexNet, similar to that in monkey IT. Lastly, to examine the origin of face-selectivity in untrained neural networks, we implemented a randomly initialized network where values in each weight kernel were randomly drawn from a weight distribution of the pre-trained AlexNet. We found that the number of face-selective neurons abruptly decreases when the weight variation is reduced to 52% of that in the pre-trained network. These results suggest that statistical variation present in the random feedforward projections could solely drive the emergence of innate face-selective neurons in the visual system. Overall, our findings provide insight into the origin of cognitive functions in both artificial and biological neural networks.

**Fig. 1:**
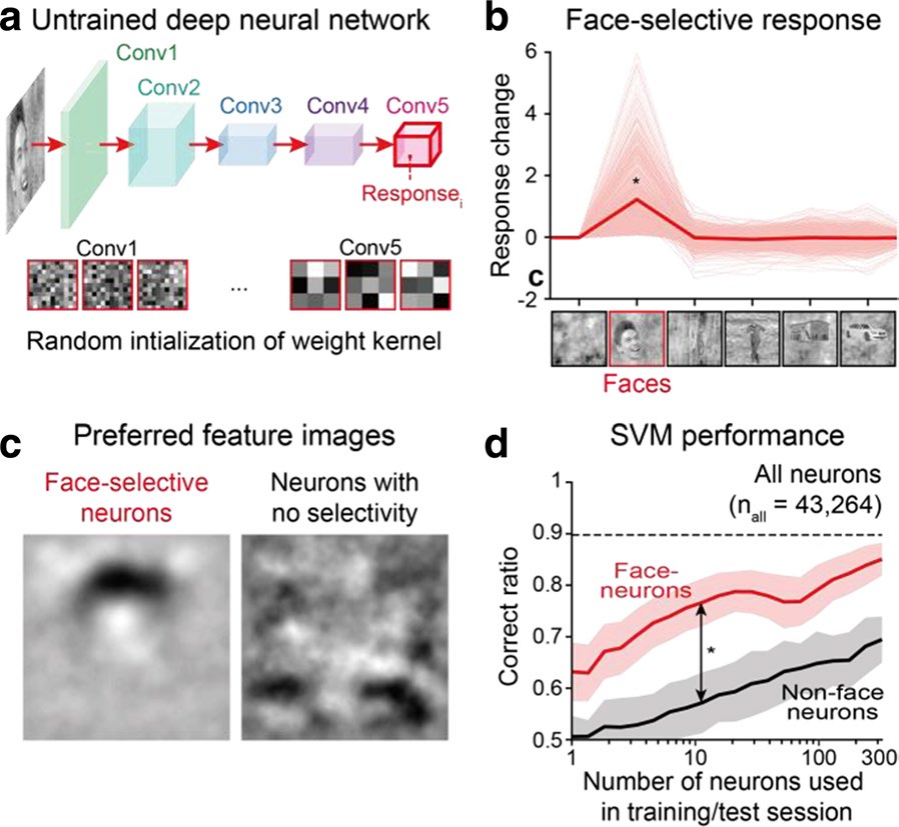
Spontaneous emergence of face selectivity in untrained networks. **a** An untrained AlexNet. **b** Tuning curves for face-selective neurons in untrained network. **c** Preferred feature images in untrained network. **d** Face classification performance of SVM using face-selective neurons in untrained network

**Acknowledgements:** this work was supported by Grant Number: (2019M3E5D2A01058328, 2019R1A2C4069863).

**References**

1. Tsao DY, Freiwald WA, Tootell RB, Livingstone MS. A cortical region consisting entirely of face-selective cells. Science. 2006; 311(5761): 670-4.
2. Livingstone MS, et al. Development of the macaque face-patch system. Nature Communications. 2017; 8(1): 1-2.
3. Deen B, et al. Organization of high-level visual cortex in human infants. Nature communications. 2017; 8(1): 1-0.

## P153 Number-selective units can spontaneously arise in untrained deep neural networks

### Jaeson Jang^1^, Gwangsu Kim^2^, Seungdae Baek^1^, Min Song^1^, Se-Bum Paik^1^

#### ^1^Korea Advanced Institute of Science and Technology, Department of Bio and Brain Engineering, Daejeon, South Korea; ^2^Korea Advanced Institute of Science and Technology, Department of Physics, Daejeon, South Korea

##### **Correspondence:** Jaeson Jang (jaesonjang@kaist.ac.kr)

*BMC Neuroscience* 2020, **21(Suppl 1)**:P153

Number sense is an ability to estimate number of visual items (numerosity) without counting, which is observed in newborn animals of various species. In single-neuron recordings in numerically naïve monkeys, it was observed that individual neurons can respond selectively to the numerosity [1]. This suggests that number-selective neurons spontaneously arise for a foundation of innate number sense, but it remains unclear how these neurons originate in the absence of learning. Here, using a deep neural network (DNN) designed from the structure of a visual pathway (AlexNet), we show that number tuning of network units can spontaneously arise in untrained networks, even in the absence of any learning. To devise an untrained network, we randomly permuted the weights of filters in each convolutional layer of the pre-trained AlexNet and examined the response to images of dot patterns representing numbers from 1 to 30. For stimuli, we used three different sets to ensure invariance of the number tuning for certain geometric factors (stimulus size, density, and area). A network unit was considered to be number-selective if its response significantly changes across the numerosity (p < 0.01, two-way ANOVA) but there is no significant effect for the stimulus set or interaction between two factors (p > 0.01). Importantly, number-selective units were observed in the permuted AlexNet (9.58% of units in the last convolutional layer), even though the network was never trained for any task after being permuted. Observed number-selective units followed the Weber-Fechner law observed in the brain, where the width of the tuning curves increases proportionally in the numerosity. We also showed that these units enable the network to perform a number discrimination task, by training a support vector machine (SVM) to compare numerosities in two different images using the response of number-selective units. Next, to explain how number-selective units emerge in permuted networks, we hypothesized that the number tuning to various numerosities can be initiated from the monotonic unit activities in the earlier layer, the response of which monotonically decreases or increases as the given numerosity increases. To test this idea, we performed a model simulation for the randomly weighed summation of tuning curves of increasing and decreasing activities and confirmed that tuning to all the tested numerosities was successfully generated. Notably, the curve tuned to smaller numbers was generated by the summation of strongly weighted decreasing activities and weakly weighted increasing activities. As expected, in the permuted AlexNet, we observed that number-selective units tuned to smaller numbers receive strong inputs from the decreasing units and vice versa. These results suggest that number-tuned neurons may spontaneously arise from the statistical variation of feedforward projections in the visual pathway during the early development stage. This finding provides new insights into the origin of cognitive functions in biological brains, as well as in artificial neural networks.

**Fig. 1:**
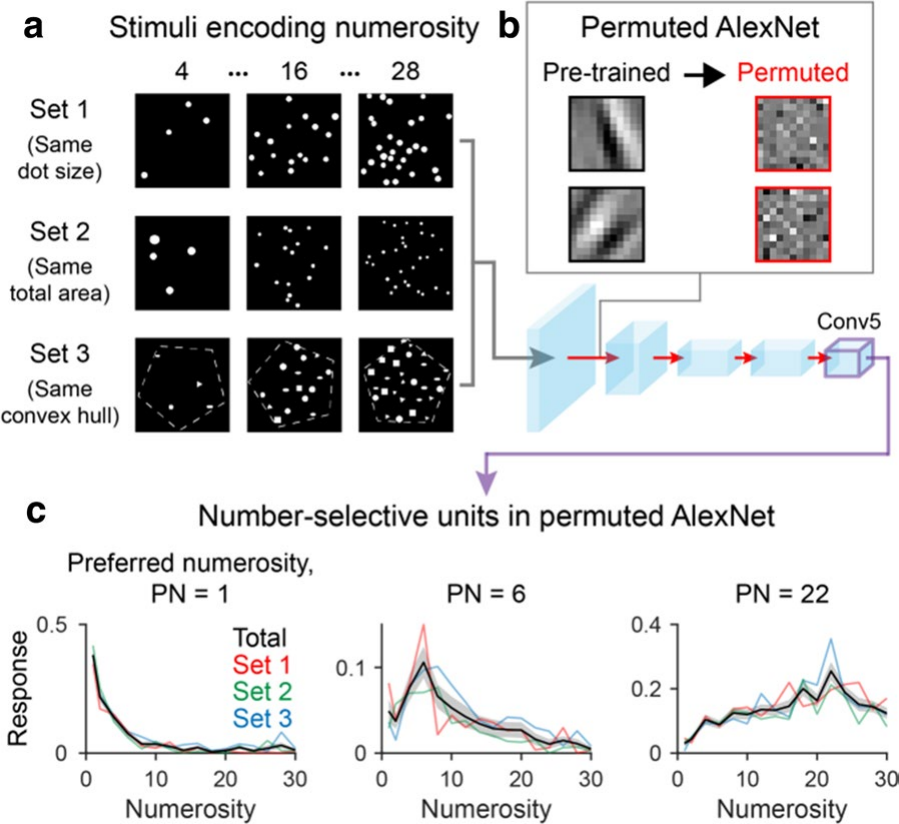
Spontaneous emergence of number-selective units in untrained deep neural networks. **a** Stimuli encoding different numerosities and geometric factors. **b** An untrained AlexNet was devised by randomly permuting the weights of the pre-trained AlexNet. **c** Tuning curves of individual number-selective network units

**Acknowledgments:** We thank the National Research Foundation of Korea (NRF) for supporting Se-Bum Paik with 2019R1A2C4069863 and 2019M3E5D2A01058328.

**Reference**

1. Viswanathan P, Nieder A. Neuronal correlates of a visual “sense of number” in primate parietal and prefrontal cortices. Proceedings of the National Academy of Sciences. 2013; 110(27): 11187-92.

## P154 Rhythmic eye movement predicts active perception of ambiguous visual stimulus

### Woochul Choi^1^, Hyeonsu Lee^2^, Se-Bum Paik^2^

#### ^1^Korea Advanced Institute of Science and Technology, Daejeon, South Korea; ^2^Korea Advanced Institute of Science and Technology, Department of Bio and Brain Engineering, Daejeon, South Korea

##### **Correspondence:** Woochul Choi (choiwc1128@kaist.ac.kr)

*BMC Neuroscience* 2020, **21(Suppl 1)**:P154

When an ambiguous sensory stimulus is given, our brain often actively interprets the given stimulus to dissolve ambiguity. A particular example is the condition of ‘bistable perception’, where a given stimulus can be interpreted as two different states. Under this ambiguity, our perception alternates between two possible interpretations quasi-periodically with switching frequency varying across individuals. This characteristic dynamics of bistable perception is thought to reveal how the brain recognizes incomplete visual signals to lead to a perceptual decision, and a number of studies have been performed to investigate the mechanism of its rhythmic perceptual alternation. However, understanding the dynamics of bistable perception has proved elusive, as it is a complicated process involving interrelated cognitive and motor processes even including top-down intention and eye movements. Recent studies reported that specific eye movement occurs during bistable perception [1], but it is still not known whether eye movements can actively induce perceptual decision, or they are just accompanied after the decision. Here, we show that eye movement may not solely induce perceptual behavior, but the eye movement patterns reflect the perceptual decisions for interpretation of ambiguous stimuli. We performed a human psychophysics experiment with simultaneous eye-tracking, using three bistable stimuli – racetrack, rotating cylinder, and Necker cube. We found that eye gaze slowly oscillates with 5-10s intervals, the period of which was positively correlated to the frequency of perceptual switch. In addition, we found that eye gaze movements were observed in the opposite directions before two different perceptual decisions are made. The preceding eye gaze can thus predict the perceptual decision with ~90% accuracy. We also found that the frequency of the saccadic eye movement during free viewing, which does not require any active interpretation, was correlated with the period of perceptual switch, implying that dynamics of eye movement reflects the characteristic of bistable perception. Next, to isolate the effect of eye movement from intention, we first asked the subjects to have a strong intention to switch (or stay) their perceived state during experiments. With such manipulations, we found that both perceptual decision and eye movements were significantly altered, compared to the case of non-intended trials. We then controlled visual stimuli so that the subject’s eye movement follows the traces of intention-controlled trials, without actual intention to change their behavior. Under this condition, even though subjects’ eye movements mimic those of the intended trial, perceptual decisions were not significantly biased. This suggests that eye movements alone cannot bias perceptual behavior in bistable perception. Taken together, the results suggest that 1) rhythmic eye movement correlates with active visual perception, 2) preceding eye gaze trajectory predicts individual decision but 3) eye movement may not solely induce perceptual decision. These results collectively suggest a relationship between eye movement control, top-down intention, and active perception.

**Fig. 1:**
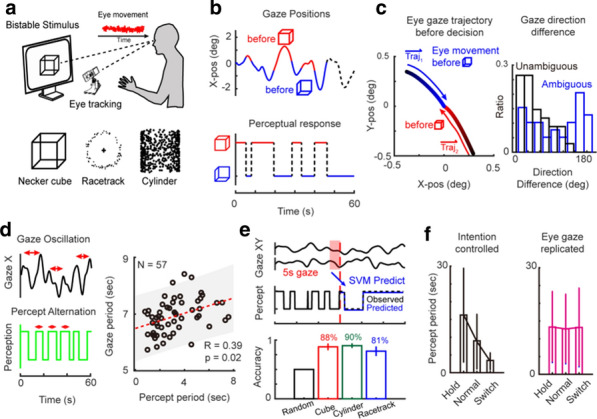
Eye gaze reflecting perceptual decisions **A** Three bistable perception tasks with eye-tracking **B** Gaze and perceptual response **C** Gaze trajectories before decision **D** Correlation between the period of gaze oscillation and perceptual alternation. **E** Gaze position can predict perception **F** Intention alters perceptual behavior but replicated eye gaze without intention does not alter perception

**Acknowledgements:** This research was supported by National Research Foundation of Korea, Grant Number: 2019M3E5D2A01058328, 2019R1A2C4069863.

**Reference**

1. Polgári P, Causin JB, Weiner L, Bertschy G, Giersch A. Novel method to measure temporal windows based on eye movements during viewing of the Necker cube. PLOS One. 2020; 15(1): e0227506.

## P155 A model for unsupervised object categorization in infants

### Sunho Lee^1^, Youngjin Park^2^, Se-Bum Paik^2^

#### ^1^Korea Advanced Institute of Science and Technology, Daejeon, South Korea; ^2^Korea Advanced Institute of Science and Technology, Department of Bio and Brain Engineering, Daejeon, South Korea

##### **Correspondence:** Youngjin Park (yodamaster@kaist.ac.kr)

*BMC Neuroscience* 2020, **21(Suppl 1)**:P155

Both the brain and recent deep neural networks (DNNs) can successfully perform visual object recognition at similar levels. However, to acquire this function, DNNs generally require a large amount of training with a huge number of labeled data, whereas the brain does not appear to need such artificially labeled images to learn. Moreover, human infants, who certainly never experienced any training, are still able to classify unfamiliar object categories [1]. The mechanism by which the immature brain can categorize visual objects without any supervisory feedback remains elusive. Here, we suggest a biologically plausible circuit model that can correctly categorize natural images without any supervision. Instead of supervised signals, which are believed to be essential to train the system, we focused on the temporal continuity of the natural scene. Natural visual stimuli to which infants are exposed repeatedly have temporal continuity [2], unlike the dataset of images used to train artificial DNNs. In this regard, to detect the discontinuity in a natural scene that is potentially equivalent to the border of the image cluster of the same object, we designed a ‘differential unit’ (Fig. 1, DU). The DU estimates the difference between the current input and delayed input before seconds, and thereby can detect the temporal difference of visual input in real-time. In addition to the DU, to memorize the representation of visual objects, we also designed a ‘readout network’ (Fig. 1, k-Winners-Take-All network and readout), which is linked to the filtered pool5 units of randomized AlexNet. The randomized AlexNet corresponds to the early visual pathway of infants and functions as an image abstractor, where its weights are randomly initialized and fixed. The connection weights between the readout and pool5 units can be updated by Hebbian plasticity, but because the DU continuously inhibits the readout, the plasticity was blocked initially. However, when the temporal difference of response becomes below a certain threshold (which means that the same object was consistently detected), the DU stops the inhibition, and connections between the ensemble of pool5 units (highly activated for that object) and the readout are strengthened. During the test session, we can identify the category of the given test images by simply choosing the readout that shows the highest response. To validate the model performance, we made a sequence of images by sorting the CIFAR-10 dataset by categories, which mimics the temporal continuity of the natural scene. The model was trained by the designed image sequence, and tested by a separate validation set. As a result, we achieved 35% classification accuracy, which is significantly higher than the chance level of 10%. Based on the present findings, we suggest a biologically-plausible mechanism of object categorization with no supervision, and we believe that our model can explain how the visual function arises in the early stages of the brain without supervised learning.

**Fig. 1:**
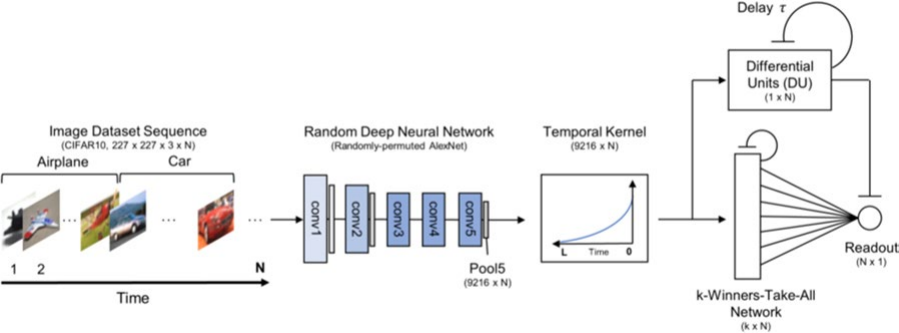
The overall structure of the model. The model consists of the randomly initialized AlexNet (image abstractor), temporal kernel, differential unit (DU), k-Winner-Take-All network and readout

**Acknowledgements:** This work was supported byNational Research Foundation of Korea (No.2019M3E5D2A01058328,2019R1A2C4069863).

**References**

1. Rakison DH, Yermolayeva Y. Infant categorization. Wiley Interdisciplinary Reviews: Cognitive Science. 2010; 1(6): 894-905.
2. Wiskott L, Sejnowski TJ. Slow feature analysis: Unsupervised learning of invariances. Neural computation. 2002; 14(4): 715-70.

## P156 Analytical solution of linearized equations of the Morris-Lecar neuron model at large constant stimulation

### Tatiana Zemskova^1^, Alexander Paraskevov^2^

#### ^1^Ecole Polytechnique, Palaiseau, France; ^2^National Research Centre “Kurchatov Institute”, Institute for Information Transmission Problems, Moscow, Russia

##### **Correspondence:** Tatiana Zemskova (zemskova.ts@phystech.edu)

*BMC Neuroscience* 2020, **21(Suppl 1)**:P156

The Morris-Lecar model (MLM) [1] is a classical biophysical model of spike generation by the neuron, which takes into account dynamics of voltage-dependent ion channels and realistically describes the spike waveform. MLM predict that upon stimulation of the neuron with sufficiently large constant depolarizing current *I*stim, there exists a finite interval of *I*stim values where periodic spike generation occurs [2-4]. Numerical simulations show that the cessation of periodic generation of spikes above the upper boundary of this interval occurs through damping of the spike amplitude, arising with a delay inversely proportional to *I*stim value. In particular, the damped dynamics can be divided into four successive stages: 1) minor primary damping, which reflects a typical transient to stationary state, 2) plateau of nearly undamped periodic oscillations, which determines the aforementioned delay, 3) strong damping, and 4) reaching a constant asymptotic value. As the last two stages resemble the well-known exponentially-damped harmonic oscillations, we tackled to find an analytical description for these stages [5].

First, we have linearized the MLM equations at the vicinity of the stationary asymptotic value of the neuronal potential. The resulting equations have been then reduced to an inhomogeneous Volterra integral equation of the 2nd kind. In turn, the latter has been transformed into an ordinary differential equation of the second order with a time-dependent coefficient at the first-order derivative. As this time dependence was just an exponential decay, we considered its asymptotic value and analytically solved the final equation. In order to verify the analytical solution found, we have compared it with the numerical solution obtained using the standard MATLAB tools for systems of ordinary differential equations.

We have accurately shown that the linearized system of equations of the MLM can be reduced to a standard equation of damped harmonic oscillations for the neuron potential. Since all coefficients of this equation are explicitly expressed through the parameters of the original MLM, one can directly (i.e. without any fitting) compare the numerical and analytical solutions for dynamics of the neuron potential at the stages of strong damping and reaching a constant asymptotic value. The results allow a quantitative study of the applicability boundary of linear stability analysis that implies exponential damping.

**References**

1. Morris C, Lecar H. Voltage oscillations in the barnacle giant muscle fiber. Biophysical Journal 1981.; 35(1): 193-213.
2. Tateno T, Harsch A, Robinson HP. Threshold firing frequency–current relationships of neurons in rat somatosensory cortex: type 1 and type 2 dynamics. Journal of Neurophysiology. 2004; 92(4): 2283-94.
3. Tsumoto K, Kitajima H, Yoshinaga T, Aihara K, Kawakami H. Bifurcations in Morris–Lecar neuron model. Neurocomputing. 2006; 69(4-6): 293-316.
4. Nguyen LH, Hong KS, Park S. Bifurcation control of the Morris-Lecar neuron model via a dynamic state-feedback control. Biological Cybernetics. 2012; 106(10): 587-594.
5. Paraskevov AV, Zemskova TS. Analytical solution of linearized equations of the Morris-Lecar neuron model at large constant stimulation, bioRxiv. 2019.

## P157 Mesoscopic spiking neuronal network model capturing the remote activation of “epilepsy” focus

### Dmitrii Zendrikov^1^, Alexander Paraskevov^2^

#### ^1^University of Zurich, Institute of Neuroinformatics, Zurich, Switzerland; ^2^National Research Centre “Kurchatov Institute”, Institute for Information Transmission Problems, Moscow, Russia

##### **Correspondence:** Dmitrii Zendrikov (dmitrii@ini.uzh.ch)

*BMC Neuroscience* 2020, **21(Suppl 1)**:P157

Spontaneous focal synchronization of collective spiking followed by induced traveling waves can occur in the cortical sheet and in cultured planar neuronal networks. In the first case, it is focal epilepsy leading to a seizure and, in the second, it is synchronization that originates from one of a few steady nucleation sites resulting in a so-called population spike. Assuming functional similarity between the nucleation sites and non-lesional epileptic foci, the major unsolved issue in both cases is that whether activation of the focus occurs inside it (i.e., autonomously relative to the interaction with surrounding neuronal tissue) or from the outside. The “internal” scenario implies that the focus spatially contains some pacemakers. In turn, several experimental findings indicate a complex spatially non-local activation of epileptic focus [1-4]. In modeling studies, we address the issue in order to verify the validity of this conclusion.

We use generative mechanistic model of planar neuronal network exhibiting irregular spontaneous population spikes, which emerge from a few spontaneously-formed stationary nucleation sites. The model consists of leaky integrate-and-fire neurons connected by synapses with short-term plasticity, forming spatially-dependent “small-world” network topology, where synaptic connection probability decreases exponentially with the distance between neurons. Spiking activity in the network occurs due to some fraction of pacemaker neurons. Importantly, the spatial configuration of pacemaker neurons was artificially engineered in order to resolve the above-mentioned problem: all pacemakers were placed within a circular central spot so that their spatial density was equal to the average density of neurons. Leaving the global dynamic regime unaffected, this spatial configuration crucially helps to clarify the activation process, visualizing of which is hindered at spatially-uniform pacemaker distribution.

Extensive simulations [5] have shown that steady and spontaneous nucleation sites of population spikes (i) can emerge in spatial regions, which are far away from the spot with pacemakers and (ii) can be activated even without direct links from pacemakers. The results demonstrate the principle possibility of external, or remote, activation of a focal source of epileptic activity in the brain and favor the interpretation in the above-mentioned experimental findings. The suggested deterministic model provides the means to study this network phenomenon systematically and reproducibly.

**References**

1. Schevon CA, et al. Cortical abnormalities in epilepsy revealed by local EEG synchrony. Neuroimage. 2007; 35(1): 140-8.
2. Zaveri HP, et al. Localization-related epilepsy exhibits significant connectivity away from the seizure-onset area. Neuroreport. 2009; 20(9): 891-5.
3. Bower MR, Stead M, Meyer FB, Marsh WR, Worrell GA. Spatiotemporal neuronal correlates of seizure generation in focal epilepsy. Epilepsia. 2012; 53(5): 807-16.
4. Paz JT, Huguenard JR. Microcircuits and their interactions in epilepsy: is the focus out of focus?. Nature Neuroscience. 2015; 18(3): 351.
5. Paraskevov AV, Zendrikov DK. Capturing remote activation of epilepsy source?. bioRxiv. 2018; 355255.

## P158 Controllability for nonlinear dynamic brain system under biological constrains

### Jiyoung Kang^1^, Hae-Jeong Park^2^

#### ^1^Yonsei University, Center for Systems and Translational Brain Sciences, Seoul, South Korea; ^2^Yonsei University College of Medicine, Department of Nuclear Medicine, Seoul, South Korea

##### **Correspondence:** Jiyoung Kang (jiyoungkang01@gmail.com)

*BMC Neuroscience* 2020, **21(Suppl 1)**:P158

Controllability of the brain system has been studied in the domain of network science. However, most of studies on the controllability of the brain have not considered the nonlinear nature of the brain. In the present study, we suggest a computational framework to control the brain system with a consideration of nonlinear brain dynamics. Our framework is based on a hypothesis that a brain with a disease has specific brain dynamics different from that of a normal brain and can be analyzed by using an energy landscape analysis. For both normal and abnormal brain systems, multistable activation states (attractors) and transition rates were investigated by performing an energy landscape analysis based on a pairwise maximum entropy model. In the current virtual framework, we simulated how dynamics of a disease brain can be changed to that of the normal brain by external treatments under biological constrains. By doing this, we tried to find a strategy for optimal treatments that control the target brain to generate brain state dynamics similar to that of the healthy brain. We assumed that the target brain changes not only at a treated region or treated connectivity, but also it induces changes in the neighbors that the treated region interacts. By allowing changes in the neighborhood in response to the treatment to a target region, we showed an optimal controllability that takes into account of the nonlinear responses of the brain after treatment. We applied the current framework to find a virtually optimal way of perturbing the network system of the schizophrenia to induced healthy state dynamics. We expect that this computational framework for controllability would help treatment planning for the nonlinear brain system, after empirical evaluation and validation.

**Acknowledgments:** This research was supported by Brain Research Program and the Korea Research Fellowship Program through the National Research Foundation of Korea (NRF) funded by the Ministry of Science and ICT (NRF-2017M3C7A1049051 and NRF-2017H1D3A1A01053094).

## P159 Effective connectivity between the forebrain and tectum in the lavar zebra fish for opto-motor behaviors

### Dongmyeong Lee^1^, Junho Son^1^, Jinseok Eo^2^, Jiyoung Kang^3^, Hae-Jeong Park^4^

#### ^1^Yonsei University College of Medicine, Seoul, South Korea; ^2^Yonsei University College of Medicine, Department of Radiology, Department of Psychiatry, Seoul, South Korea; ^3^Yonsei University, Center for Systems and Translational Brain Sciences, Seoul, South Korea; ^4^Yonsei University College of Medicine, Department of Nuclear Medicine, Seoul, South Korea

##### **Correspondence:** Dongmyeong Lee (dmyeong@inu.ac.kr)

*BMC Neuroscience* 2020, **21(Suppl 1)**:P159

The role of the forebrain of the zebra fish has not been clearly understood particularly during the opto-motor behaviors. Most studies on the opto-motor behavior have been researched in terms of interactions between the retina and the tectum. However, the role of the forebrain has not been fully explored. In the current study, we explored how the forebrain works during the zebra’s opto-moto behavior by estimating the context-dependent effective connectivity between the tectum and the forebrain using dynamic causal modelling (DCM) of the calcium signals. We hypothesized that the forebrain plays a role in modulating the tectum as top-down process. For the six lavar zebra fish (elavl3: H2B-GCaMP6f, 5~7dpf) during opto-motor behavior [1], we applied principal component analysis to the calcium imaging time series to identify neural modes that respond synchronously to the optic stimulus. As a result, three neural modes at the forebrain and two (left/right) neural modes at the tectum were identified and used to construct a functional circuitry for the zebra fish. A DCM with a convolution-based dynamic neural state model and a dynamic calcium ion concentration model for calcium signals [2] was applied to fit the time series of the five modes. Onsets of both stimuli were assigned to the bilateral tectum to derive the system. The left and right stimuli were used independently as two modulation inputs to effective connectivity. Using DCM, we inverted effective connectivity among the fully connected nodes. For a group level analysis of the direction-dependent effective connectivity, we used parametric empirical Bayesian analysis of the individual effective connectivity. The neurons related to opto-moto behaviors in the forebrain were not highly lateralized compared to those at the tectum. The left and right optic stimulation modulated the effective connectivity from the forebrain to the tectum in a direction dependent way. When responding to a directional stimulus, the mode in the tectum suppresses the contra-lateral neural mode. Our computational modelling results suggest the active involvement of the forebrain in modulating top-down effective connectivity for opto-motor behaviors.

**References**

1. Chen X, et al. Brain-wide organization of neuronal activity and convergent sensorimotor transformations in larval zebrafish. Neuron. 2018; 100(4): 876-90.
2. Jung K, Kang J, Chung S, Park HJ. Dynamic causal modeling for calcium imaging: Exploration of differential effective connectivity for sensory processing in a barrel cortical column. NeuroImage. 2019; 201: 116008.

## P160 Dynamic causal modeling of single cell level activities observed in the calcium imaging

### Jinseok Eo^1^, Jiyoung Kang^2^, Dongmyeong Lee^3^, Junho Son^3^, Hae-Jeong Park^4^

#### ^1^Yonsei University, Seoul, South Korea; ^2^Yonsei University, Center for Systems and Translational Brain Sciences, Seoul, South Korea; ^3^Yonsei University College of Medicine, Seoul, South Korea; ^4^Yonsei University College of Medicine, Department of Nuclear Medicine, Seoul, South Korea

##### **Correspondence:** Jinseok Eo (jinseok007@naver.com)

*BMC Neuroscience* 2020, **21(Suppl 1)**:P160

Multi-photon calcium imaging (CaI) makes it possible to analyze distributed neural activity in the neuronal level. Most studies using CaI have analyzed the neural system in terms of functional connectivity, i.e., temporal synchrony among nodes, which lacks information on the asymmetric interactions, called effective connectivity. Recent computational modelling techniques have been introduced to infer effective connectivity among neural populations using dynamic causal modelling (DCM) [1,2]. These studies have used a firing-rate based neural state dynamic model, combined with a calcium ion kinetic model in the neural population level, which is not appropriate to model neural interactions among individual neurons. In the single neuron level, a quadratic gaussian integrate-and-fire neural state model (QGIF) in combination with a calcium kinetic equation was proposed to fit CaI at a single neuron [3]. To make it applicable to exploring interactions among multiple neurons, we extended the previous model of [3] to a general circuit with multi-nodes in the DCM framework. We utilized a QGIF model for a single neuronal activity, with conductance based neural connectivity (synaptic conductances are approximated with alpha function), and a CaI state dynamic equation. Bayesian model optimization is applied to find optimal model parameters (e.g., effective connectivity) using a variational expectation maximization scheme implemented in the DCM. We confirmed the reliability of the proposed modeling and model inversion process using simulation experiments. We applied the proposed method to explore effective connectivity among neural cells from the neural activity observed in CaI at the rodent’s barrel cortex during successful and failed whisking. The results suggest the plausibility of the proposed method in the analysis of neural interactions observed in the CaI in the neuron level.

**References**

1. Jung K, Kang J, Chung S, Park HJ. Dynamic causal modeling for calcium imaging: Exploration of differential effective connectivity for sensory processing in a barrel cortical column. NeuroImage. 2019; 201: 116008.
2. Rosch RE, Hunter PR, Baldeweg T, Friston KJ, Meyer MP. Calcium imaging and dynamic causal modelling reveal brain-wide changes in effective connectivity and synaptic dynamics during epileptic seizures. PLoS Computational Biology. 2018; 14(8): e1006375.
3. Rahmati V, Kirmse K, Marković D, Holthoff K, Kiebel SJ. Inferring neuronal dynamics from calcium imaging data using biophysical models and Bayesian inference. PLoS Computational Biology. 2016; 12(2): e1004736.

## P161 Less is more: a new computational approach for analyzing EEG for brain computer interface applications

### Andi Partovi^1^, Mehrshad Hosseini^2^, Milad Soleimani^2^, Sahand Sadeghpoor^2^, Erfan Habibi Panah Fard^2^, Kiana Liaghat^2^, Soroush Ziaee^2^, Farhad Goodarzy^3^

#### ^1^The University of Melbourne, Biomedical Engineering, Melbourne, Australia; ^2^KNT University of Technology, Tehran, Iran; ^3^The University of Melbourne, Medicine, Dentistry and Health Sciences, Melbourne, Australia

##### **Correspondence:** Farhad Goodarzy (goodarzy@unimelb.edu.au)

*BMC Neuroscience* 2020, **21(Suppl 1)**:P161

Brain computer interfaces (BCI) are amongst the exhilarating applications of computational neuroscience and have been increasingly the focus of research around the world. Currently, abundant effort in BCI research is devoted to offline analysis of recorded data to achieve higher accuracies in decoding [1]. Although this has led to the development of new methods and algorithms, the problem of online decoding of subject intentions remains a challenging one [2]. One of the restrictive steps of BCI design is the need for complex preprocessing steps required to extract features from the recorded signals to be used by the classifiers to distinguish intentions of the subject [1]. The other hindering factor is the variability of the recorded signals. EEG recordings use between 20-128 electrodes at sampling rates of 250 up to 1KHz for BCI applications. This data is recorded from the whole brain and in/between subject(s) variability intensifies the problem even further. This problem is currently mitigated through manual and careful feature engineering steps and tweaking of classifier parameters.

We are proposing to reduce the complexity of the architecture by 1) using only raw recorded signals with no preprocessing, 2) reducing the number of channels used for classification and 3) a single convolutional neural network (CNN) to be used for classification amongst all subjects. We have limited our preliminary results to EEG signal analysis of a left/right/rest motor imagery task as this is the most popular signal used in BCI applications. We have previously shown [3] that our proposed CNN can reliably decode intentions utilizing same architecture for multiple subjects. Here, we are extending our method to 4 new subjects and show that drastically reducing channels has insignificant effect on decoding results. We have also expanded our decoding results to 3-class classification and obtained the same decoding accuracies by only using a few channels for classification. Our results are summarized in Table 1.

**Table 1:** Summary of results

	2-Class			3-Class		
# of electrodes	all	10	2	all	10	3
S1 (64)	84	84	72	60	60	58
S2 (64)	89	90	81	68	66	60
S3 (38)	90	88	80	73	74	70
S4 (22)	86	86	78	82	80	75

Our results show that drastically reducing the complexity of data, can still yield comparable performance while using a single decoder for multiple subjects. Since the choice of the channels for decoding is based on the mental task, one can envision the use of these methods to create practical, reliable online BCI solutions. Also using raw data for analysis and the use of a single architecture to classify all subjects allows for a hardware to be designed to even further improves efficiency of the system.

**Acknowledgments:** We thank NHMRC for funding Goodarzy F. with the project grant.

**References**

1. Lotte F, et al. A review of classification algorithms for EEG-based brain–computer interfaces: a 10 year update. Journal of Neural Engineering. 2018; 15(3): 031005.
2. Movahedi F, Coyle JL, Sejdić E. Deep belief networks for electroencephalography: A review of recent contributions and future outlooks. IEEE Journal of Biomedical and Health Informatics. 2017; 22(3): 642-52.
3. Partovi A, et al. A Convolutional Neural Network Model for Decoding EEG signals in a Hand-Squeeze Task. In 8th International Winter Conference on Brain-Computer Interface. 2020; 1-6.

## P162 Approximative models for enabling multi-scale simulations

### Mikko Lehtimäki^1^, Marja-Leena Linne^2^, Lassi Paunonen^3^

#### ^1^Tampere University, Faculty of Medicine and Health Technology, Tampere, Finland; ^2^Tampere University, Medicine and Health Sciences, Tampere, Finland; ^3^Tampere University, Faculty of Information Technology and Communication Sciences, Tampere, Finland

##### **Correspondence:** Mikko Lehtimäki (lehtimaki.mikko@gmail.com)

*BMC Neuroscience* 2020, **21(Suppl 1)**:P162

In computational neuroscience there is a great demand to incorporate more molecular and cellular level detail into mathematical models of neuronal networks. This is deemed necessary in order to recreate phenomena such as learning, memory and behavior in silico. However, numerical simulation of such multi-scale models is resource intensive, if not impossible. This problem has been partially overcome by using simplified synapse, neuron and population models that replace biological variables and mechanisms from the models with phenomenological descriptions. While useful, this approach causes information loss that might diminish the value of such models, as the variables might lack biological meaning.

In this study we present approximation as an alternative to simplification. By using mathematical model order reduction (MOR) methods approximations can be derived algorithmically. Here we compute reduced models with the Discrete Empirical Interpolation Method (DEIM) [1] algorithm along with its advanced variants. The appeal of these methods is that there is no need to linearize the model, make assumptions of the system behavior or discard any variables. A reduced model can be simulated efficiently in a low-dimensional subspace where a smaller number of equations needs to be solved. An approximation of the original high-dimensional model can be reconstructed at any time. The acceleration in simulation time gained this way requires no special hardware and can be readily implemented in any programming language.

We discuss results from approximating three nonlinear systems; chemical reactions in the synapse, a compartmental neuronal network and a multi-dimensional mean-field model [2-4]. We have made the code to approximate the mean-field model open source [https://github.com/Mikkolehtimaki/neuro-mor]. We demonstrate the value of reduced models in computational neuroscience and explain the pros and cons of several different reduction methods with regards to the above models. Especially implementation of mathematical model order reduction algorithms in neuronal simulators and using reduced models in neuromorphic hardware are potential applications of these methods for enabling multi-scale simulations of brain activity.

**Acknowledgements**: M.L. is supported by TUNI Graduate School, M.-L.L by Academy of Finland grant 297893 and L.P. by grants 298182 and 310489. This research has received funding from the European Union’s Horizon 2020 Framework Programme for Research and Innovation under the Specific Grant Agreement No. 785907 (Human Brain Project SGA2).

**References**

1. Chaturantabut S, Sorensen DC. Nonlinear model reduction via discrete empirical interpolation. SIAM Journal on Scientific Computing. 2010; 32(5): 2737-64.
2. Lehtimäki M, Paunonen L, Pohjolainen S, Linne ML. Order reduction for a signaling pathway model of neuronal synaptic plasticity. IFAC-PapersOnLine. 2017; 50(1): 7687-92.
3. Lehtimäki M, Paunonen L, Linne ML. Projection-based order reduction of a nonlinear biophysical neuronal network model. In IEEE 58th Conference on Decision and Control. 2019; 1-6.
4. Lehtimäki M, Seppälä I, Paunonen L, Linne ML. Accelerated Simulation of a Neuronal Population via Mathematical Model Order Reduction. In 2nd IEEE International Conference on Artificial Intelligence Circuits and Systems. 2020; 118-122.

## P163 Biophysics and dynamics shape the cross-correlation properties of monosynaptic connections

### Horacio Rotstein^1^, Rodrigo Pena^2^

#### ^1^New Jersey Institute of Technology, Federated Department of Biological Sciences, NJIT / Rutgers University, Newark, New Jersey, United States of America; ^2^New Jersey Institute of Technology, Federated Department of Biological Sciences, Newark, New Jersey, United States of America

##### **Correspondence:** Horacio Rotstein (horacio@njit.edu)

*BMC Neuroscience* 2020, **21(Suppl 1)**:P163

Finely-timed spike relationships provide knowledge of putative monosynaptic connections in populations of neurons. Recent experiments involving hippocampal *in vivo* recordings were able to demonstrate such a relationship by means of the cross-correlation function (CCF) [1,2]. A sharp peak within a few milliseconds in the CCF indicates the presence of a connection. Yet, neurons that are not monosynaptically connected can emit spikes within some short temporal distance as a result of network co-modulation [3], usually in the form of background noise. In general, there is an agreement that CCFs are shaped by either the connectivity, synaptic properties, or background activity [4]. However, it remains unclear whether and how the postsynaptic intrinsic neuronal properties such as the ionic currents’ nonlinearities and time constants shape the CCFs between pre- and postsynaptic neurons. The presence of presynaptic-dependent postsynaptic signatures may serve to differentiate between correlation and causation.

We address these issues by combining biophysical modeling, numerical simulations and dynamical systems tools. We extend the framework developed in [5] to describe an ultra-precise monosynaptic connection by including ionic currents with representative dynamics. The model consists of two neurons receiving uncorrelated noise where the presynaptic neuron sends a fixed number of synaptic events to the postsynaptic neuron. CCF is computed as an average over a number of trials. We consider a number of scenarios corresponding to different levels of the ionic currents, their nonlinearities and effective time constants.

Our results show the emergence of an additional slower and wider temporal relationship, after the sharp peak in the CCF. This relationship depends on the dynamic properties present in the postsynaptic neuron model (ionic currents) in the subthreshold regime. Upon a synaptic event, if the neuron is not on the verge of a spike, it will increase its voltage following some dynamics, which depends particularly on the effective time constant, and which will be reflected in the CCF. This temporal relationship may not be clearly observed in experiments due to a high signal-to-noise ratio and is not capturing external modulation effects. We explain this effect using a phase-plane description where we capture the spike-initiation nonlinearity in terms of nullclines and connect it to the CCF.

We expect that these results will help the identification of monosynaptic connections between different neuron types, in particular, those connections among neurons from different classes.

**Acknowledgments:** This work was supported by the National Science Foundation grant DMS-1608077 (HGR).

**References**

1. English DF, et al. Pyramidal cell-interneuron circuit architecture and dynamics in hippocampal networks. Neuron. 2017; 96(2): 505-20.
2. Constantinidis C, Goldman-Rakic PS. Correlated discharges among putative pyramidal neurons and interneurons in the primate prefrontal cortex. Journal of Neurophysiology. 2002; 88(6): 3487-97.
3. Yu J, Ferster D. Functional coupling from simple to complex cells in the visually driven cortical circuit. Journal of Neuroscience. 2013; 33(48): 18855-66.
4. Ostojic S, Brunel N, Hakim V. How connectivity, background activity, and synaptic properties shape the cross-correlation between spike trains. Journal of Neuroscience. 2009; 29(33): 10234-53.
5. Platkiewicz J, Saccomano Z, McKenzie S, English D, Amarasingham A. Monosynaptic inference via finely-timed spikes. arXiv preprint arXiv:1909.08553. 2019.

## P164 Using entropy to compute phase transitions of large networks of neurons

### Wei Qin, Andre Peterson

#### The University of Melbourne, Biomedical Engineering, Melbourne, Australia

##### **Correspondence:** Wei Qin (wqin1@student.unimelb.edu.au)

*BMC Neuroscience* 2020, **21(Suppl 1)**:P164

Phase transitions are often used to describe pathological brain state transitions observed in neurological diseases such as epilepsy. Typically, the study of the dynamics of neurons that are nonlinearly coupled and have complex network structures is done via large scale numerical simulations, which are mathematically intractable. Otherwise, analysis is performed where the network structure is averaged over and made spatially homogeneous. For a networked nonlinear dynamical system, phase transitions or bifurcations are computed via changes in the local stability around the fixed points. However, in such a system it is very difficult to compute the fixed points as the dimensionality of the system becomes large due to nested nonlinearities. We know from numerical simulations that the system becomes ‘chaotic’ [1] as the order parameter (variance of the connectivity matrix) is increased and that microscopically this phase transition corresponds to an exponential increase in the number of fixed points [2]. This phase transition has also been computed for heterogeneous network structures such as Dale’s law [3]. However, it is very difficult to numerically verify these results. To quantify the change in network dynamics, we compute the entropy, a quantity which describes the number of states or information in a system. We show in this paper that the Network entropy (NE), a term derived from Shannon Entropy, can be used as a numerical indicator of a change in the number of equilibria. Hence, it is also a numerical method to estimate the change in stability of a network. It is developed via a Symbolic Dynamic approach based on probability distributions of the system state, which provides a measure of the number of states of the system.

In this paper, a first order neural model with a time-constant and instantaneous synapses is networked. The network connectivities are described by a random matrix with mean and variance. Dale’s law can be integrated into the model by changing the connectivity matrix. We estimate the stability of a network via measuring the entropy of the network states using numerical simulations with different realisations of the connectivity matrix. The result demonstrates that the transition points from the analytical results for each case coincided with the measured NEs. It suggests the NEs can be used in numerical simulations to estimate the changes in the number of the fixed points, a.k.a. the phase transitions. This work provides a novel approach to estimate the network states and phase transitions via numerical simulations. Future works are needed to discover the mathematical relationship between the fixed points and entropy. Furthermore, it is interesting to use entropy to predict the dynamical behaviours of a system in an early stage. The discovery can be used to understand the brain state transitions and for the early diagnosis of neurological diseases, such as epilepsy.

**References**

1. Stern M, Sompolinsky H, Abbott LF. Dynamics of random neural networks with bistable units. Physical Review E. 2014; 90(6): 062710.
2. Wainrib G, Touboul J. Topological and dynamical complexity of random neural networks. Physical Review Letters. 2013;110(11): 118101.
3. Ipsen JR, Peterson AD. Consequences of Dale’s law on the stability-complexity relationship of random neural networks. 2019; arXiv: 1907.07293.

## P165 Using dynamical mean field theory to study synchronization in the brain.

### Isabelle Harris^1^, Anthony Burkitt^2^, Hamish Meffin^3^, Andre Peterson^2^

#### ^1^The University of Melbourne, Department of Medicine, Melbourne, Australia; ^2^The University of Melbourne, Department of Biomedical Engineering, Melbourne, Australia; ^3^National Vision Research Institute, Melbourne, Australia

##### **Correspondence:** Isabelle Harris (iharris@student.unimelb.edu.au)

*BMC Neuroscience* 2020, **21(Suppl 1)**:P165

This work focuses on the dynamics of large networks of neurons, and particularly aims to study the effects of brain structures and functions on state transitions, such as those found in epilepsy. We derive a measure of synchronization based on network structures to identify a state change in the system, specifically, the state change between a non-seizure state and a seizure state.

Currently, the modelling framework in theoretical neuroscience focuses on using dynamical systems analysis of neural field models, and numerical simulations to disentangle the influences of structure and function on brain dynamics. However, these models and methods use continuous spatial averages of network connectivity. In this work, we are particularly interested in the spatial structures and functions that induce a state transition from a state of intrinsically fluctuating and complex activity (non-seizure state), to a state of synchronisation and simplistic activity (seizure state). Using a first order neural network model with a discrete spatial field given by a coupling matrix, a set of self-consistent equations that describe the nature of the activity of network structures with populations of neurons, can be derived using dynamical mean field theory [1]. This set of self-consistent equations can be solved semi-analytically, and we use these solutions to derive a measure of synchronisation and hence, excitability: the coefficient of variation (CV). The CV is a common measure of synchrony used in theoretical neuroscience [2], but it has also been recently used in the analysis of animal model data [3]. We use the derived expression of CV to show that under certain network structure and function conditions there exists a transition from a state of intrinsic fluctuating activity to a state of synchronization and hyper-excitation. This state transition is analogous to the transition from a non-seizure to a seizure state. Furthermore, we calculate the CV for numerically simulated time series outputs of the model used, to compare and verify our analytical expression of CV.

This measure of CV defined in this work is dependent on the network structures that are thought to be instrumental in initiating seizure transitions in the brain. As seizure events are typically due to pathological brain structures and function, we have used the CV as a theoretical measure of whether particular brain structures are susceptible to epileptic state transitions in the brain.

**References**

1. Mastrogiuseppe F, Ostojic S. Intrinsically-generated fluctuating activity in excitatory-inhibitory networks. PLoS Computational Biology. 2017; 13(4): e1005498.
2. Meffin H, Burkitt AN, Grayden DB. (2004). An analytical model for the ‘large, fluctuating synaptic conductance state’ typical of neocortical neurons in vivo. Journal of Computational Neuroscience. 2004; 16(2): 159–175.
3. Fontenele AJ, et al. Criticality between cortical states. Physical Review Letters. 2019; 122(20): 208101.

## P166 Compensating method for the lack of connection on topographic neuron network edge

### Cecilia Romaro^1^, Antonio C Roque^1^, Jose R C Piqueira^2^

#### ^1^University of Sao Paulo, Physics Department, Sao Paulo, Brazil; ^2^Escola Politecnica, Department of Telecommunications and Control Engineering, Sao Paulo, Brazil

##### **Correspondence:** Cecilia Romaro (cecilia.romaro@usp.br)

*BMC Neuroscience* 2020, **21(Suppl 1)**:P166

Studying the dynamics of a neuron network has been a challenge to computational Neuroscience [1,2]. Doing so in a neuron network with topographic organization is even more demanding due to the boundary condition, i.e. the interruption of the topographic pattern of connection in network edges, which changes network boundary activity. The neurons on the edge of the network present underside behavior due to a lack (or excess) of connections and a torus solution may introduce undesired oscillations. Facing such strain, this work presents a method based on mean field potential (i.e. first and second-order statistics of neuron network dynamics) to sustain neuron boundary activity – such as neurons on the core of the layer network activity – without introducing an oscillation component.

This method is based on the rescaling presented on CNS previous works and consists of:

Step 1: Calculating the scale factor k_i for any neuron i in network as follows: for a neuron i, k_i is given by the average of total number of connections received divided by the average of total number of connections that would be received IF the network had no boundaries – was a set of infinity neurons;

Step 2: Increasing the synaptic weights by dividing them by the square root of the scale factor;

Step 3: Providing each cell with a DC input current with a value corresponding to the total input lost due to network edge (boundary cut).

In essence, the boundary correction method numerically estimates the normalized density function of connection on the first step, then weights each neuron connection based on this density, and finally balances the threshold to grant the neuron/layer activity. This method was successfully applied on consolidated models such as Brunel [1] and PD [2], among others.

Firstly the models were reimplemented and the results were reproduced. Secondly, a topographic patter of connection was introduced to the models including the consideration that neurons near each other have a higher probability of connection then those further from each other. A different activity rises on both network boundary neurons and sometimes on core neurons. This method was applied and the activities were driven back to the original ones.

The algorithmic of rescaling method can be found in any one of example-application available in GitHub (https://github.com/ceciliaromaro/recoup-the-first-and-second-order-statistics-of-neuron-network-dynamics)

**Acknowledgements:** This work was produced as part of the activities of FAPESP Research, Disseminations and Innovation Center for Neuromathematics (Grant 2013/07699-0, S. Paulo Research Foundation).

**References**

1. Brunel N. Dynamics of sparsely connected networks of excitatory and inhibitory spiking neurons. Journal of Computational Neuroscience. 2000; 8(3): 183–208
2. Potjans TC and Diesmann M. The cell-type specific cortical microcircuit: relating structure and activity in a full-scale spiking network model. Cerebral Cortex. 2014; 24: 785-806.

## P167 Modelling ipRGC-influenced light response on circadian phase, melatonin suppression and subjective sleepiness.

### Tahereh Tekieh^1^, Peter Robinson^2^, Steven Lockley^3^, Stephan McCloskey^2^, M S Zobaer^2^, Svetlana Postnova^2^

#### ^1^The University of Sydney, Sydney, Australia; ^2^The University of Sydney, School of Physics, Sydney, Australia; ^3^Monash University, Turner Institute for Brain and Mental Health, School of Psychological Sciences, Melbourne, Australia

##### **Correspondence:** Tahereh Tekieh (tahereh.tekieh@sydney.edu.au)

*BMC Neuroscience* 2020, **21(Suppl 1)**:P32

A physiologically-based model of arousal dynamics is extended to incorporate the spectral effects of light (as an input to the model) on the circadian rhythms, melatonin dynamics and subjective sleepiness. Doing this, photopic illuminance in the model is replaced with melanopic irradiance which, reflects the role of melanopsin, a photopigment expressed in ipRGCs (intrinsically photosensitive retinal ganglion cells). Melanopsin-expressing ipRGCs are the primary cells in the retina mediating the effect of light to different non-visual related brain regions. Melanopsins are short wavelength sensitive and their main target is the circadian clock located in suprachiasmatic nuclei (SCN), with output signals regulating sleep/wake cycles, alertness, and hormone secretion. The melanopic irradiance is thus used as the light input to the model, which affects the dynamic circadian oscillator, melatonin (hormone produced in pineal gland) profile and sleepiness. The dynamic circadian oscillator is extended according to the melanopic irradiance definition and tested against experimental circadian phase dose- and phase-response data. The function which demonstrates melatonin suppression in presence of light is re-calibrated against melatonin dose-response data for monochromatic and polychromatic light sources. A new light-dependent term is then introduced into the homeostatic weight component of subjective sleepiness to represent the direct effect of light. The new term responds dynamically to light and is calibrated against experimental data with different light spectrums. The model predictions are compared to a total of 14 experimental studies containing 26 data sets for 14 different spectral light profiles. The extended melanopic model shows an average reduction in prediction error relative to the model used prior. Overall, incorporating melanopic irradiance allows simulation of wavelength-dependent responses to light observed in experiments and explains most of the observations. Models demonstrating the effect of light on circadian dynamics, sleep, and sleepiness need to use ipRGC-influenced responses as a non-visual measure of light; e.g., melanopic irradiance, instead of the traditionally used illuminance based on the visual system.

## P168 Spike initiation properties in the axon: simulations in a biophysically detailed model

### Nooshin Abdollahi^1^, Amin Kamaleddin Ezabadi^2^, Stephanie Ratte^2^, Steve Prescott^3^

#### ^1^Univeristy of Toronto, Institute of Biomaterials and Biomedical Engineering, Toronto, Canada; ^2^The Hospital for Sick Children, University of Toronto, Institute of Biomaterials and Biomedical Engineering, Neuroscience and Mental Health, Toronto, Canada; ^3^University of Toronto, Institute of Biomaterials and Biomedical Engineering, Neurosciences and Mental Health & Department of Physiology, Toronto, Canada

##### **Correspondence:** Nooshin Abdollahi (nooshin.abdollahi@mail.utoronto.ca)

*BMC Neuroscience* 2020, **21(Suppl 1)**:P168

Spikes are usually initiated at the axon initial segment (AIS), the most excitable site of a neuron. Yet other regions of the neuron are also excitable; indeed, axonal excitability is critical for spike propagation. While there are many studies on somatic and dendritic excitability, axon excitability has yet to be thoroughly investigated in most neurons because the small size of the axon precludes most experiments. There are some recordings from the cut end of axons (i.e. blebs) suggesting that axons do not spike repetitively during sustained depolarization but, instead, spike only at the onset of abrupt depolarization, consistent with class 3 excitability. However, it remains unclear whether transient spiking accurately reflects axon excitability or is an artifact of axon damage. Using a novel optogenetic approach, recent experiments from our lab have shown that axon does indeed have class 3 excitability. Although the optogenetic method is less invasive than bleb recordings, it still has some limitations that necessitated simulations in order to definitively interpret the experimental results. I have built a multicompartment model of a pyramidal neuron with a detailed myelinated axon that reproduces the observed experimental data collected in our lab. The model has helped us confirm the site of spike initiation based on the shape (kinkiness) of spikes recorded in the soma. Simulations also confirmed that even when targeting the axon for photostimulation, a small degree of stray light can hit the dendrites and evoked spikes in the AIS. The results ultimately confirm that unlike spike initiation in the AIS, which relies on class 1 excitability, spike propagation in the axon occurs on the basis of class 3 excitability (Fig. 1).

**Fig. 1:**
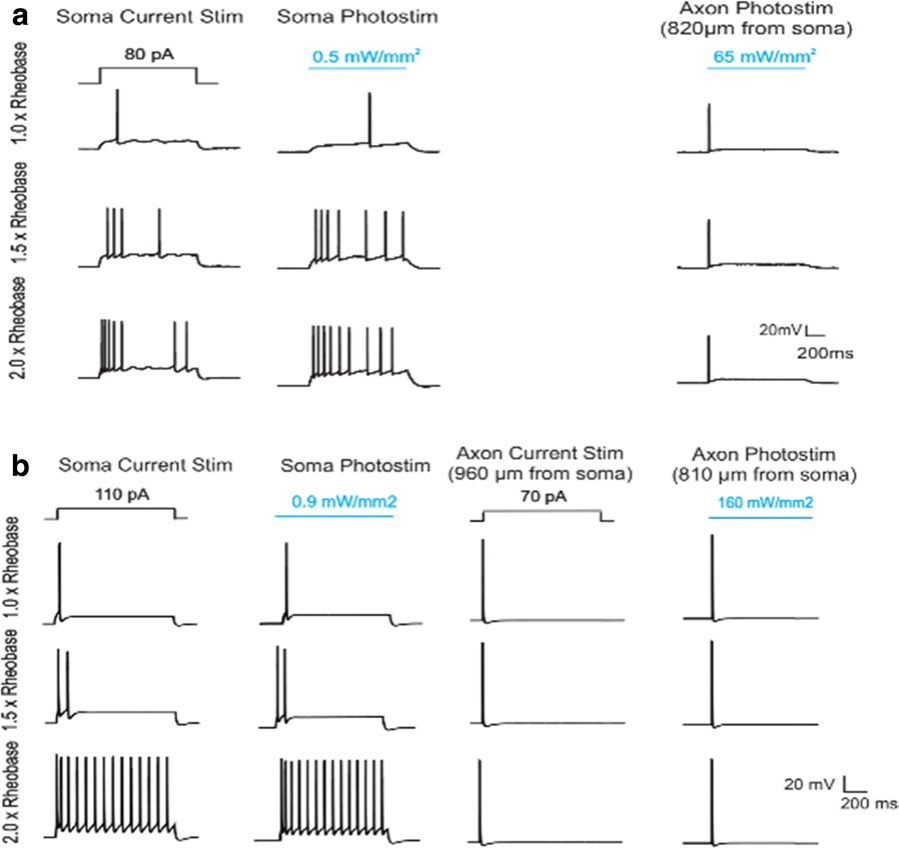
**A** Experimental results. **B** Simulation results. Current injection and photostimulation of the soma evoked repetitive spiking, consistent with class 1 excitability. However, current injection and photostimulation of the axon evoked a single spike at stimulus onset, consistent with class 3 excitability

## P169 Entrainment of competitive threshold-linear networks

### Andrea Bel^1^, Horacio Rotstein^2^, Walter Reartes^1^

#### ^1^Universidad Nacional del Sur, Departamento de Matemática, Bahía Blanca, Argentina; ^2^New Jersey Institute of Technology, Federated Department of Biological Sciences, NJIT / Rutgers University, Newark, New Jersey, United States of America

##### **Correspondence:** Andrea Bel (andrea.bel@uns.edu.ar)

*BMC Neuroscience* 2020, **21(Suppl 1)**:P169

Neuronal oscillations are ubiquitous in the brain and emerge from the combined activity of the participating neurons (or nodes), the connectivity and the network topology. Recent neurotechnological advances have made it possible to interrogate neuronal circuits by perturbing one or more of its nodes. The response to periodic inputs has been used as a tool to identify the oscillatory properties of circuits and the flow of information in networks. However, a general theory that explains the underlying mechanisms and allows to make predictions is lacking beyond the single neuron level.

Threshold-linear network (TLN) models describe the activity of connected nodes where the contribution of the connectivity terms is linear above some threshold value (typically zero), while the network is disconnected below it. In their simplest description, the dynamics of the individual nodes are one-dimensional and linear. When the nodes in the network are neurons or neuronal populations, their activity can be interpreted as the firing rate, and therefore the TLNs represent firing rate models [1].

Competitive threshold-linear networks (CTLNs) are a class of TLNs where the connectivity weights are all negative and there are no self-connections [2,3]. Inhibitory networks arise in many neuronal systems and have been shown to underlie the generation of rhythmic activity in cognition and motor behavior [4,5]. Despite their simplicity, TLNs and CTLNs produce complex behavior including multistability, periodic, quasi-periodic and chaotic solutions [2,3,6].

In this work, we consider CTLNs with three or more nodes and cyclic symmetry in which oscillatory solutions are observed. We first assume that an external oscillatory input is added to one of the nodes and, by defining a Poincaré map, we numerically study the response properties of the CTLN networks. We determine the ranges of input amplitude and frequency in which the CTLN is able to follow the input (1:1 entrainment). For this we define local and global entrainment measures that convey different information. We then study how the entrainment properties of the CTLNs is affected by changes in (i) the time scale of each node, (ii) the number of nodes in the network, and (iii) the strength of the inhibitory connections. Finally, we extend our results to include other entrainment scenarios (e.g., 2:1) and other network topologies.

**Acknowledgments:** This work was supported by the Universidad Nacional del Sur grant PGI 24/L113-2019 (AB, WR) and the National Science Foundation grant DMS-1608077 (HGR).

References

1. Dayan P, Abbott LF. Theoretical neuroscience: computational and mathematical modeling of neural systems. The MIT Press. 2001.
2. Curto C, Morrison K. Pattern completion in symmetric threshold-linear networks. Neural Computation. 2016; 28(12): 2825-52.
3. Morrison K, Degeratu A, Itskov V, Curto C. Diversity of emergent dynamics in competitive threshold-linear networks: A preliminary report. arXiv preprint. 2016.
4. Arriaga M, Han EB. Dedicated hippocampal inhibitory networks for locomotion and immobility. Journal of Neuroscience. 2017; 37(38): 9222-38.
5. Burke DA, Rotstein HG, Alvarez VA. Striatal local circuitry: a new framework for lateral inhibition. Neuron. 2017; 96(2): 267-84.
6. Hahnloser RL. On the piecewise analysis of networks of linear threshold neurons. Neural Networks. 1998; 11(4): 691-7.

## P170 Optically imaged map of orientation preferences in visual cortex of an Australian marsupial, the Tammar Wallaby Macropus eugenii.

### Young Jun Jung^1^, Ali Almasi^2^, Shi Sun^1^, Shaun Cloherty^3^, Hamish Meffin^4^, Michael Ibbotson^5^, Molis Yunzab^2^, Sebastien Bauquier^6^, Marilyn Renfree^7^

#### ^1^The University of Melbourne, Department of Optometry and Vision Sciences, Melbourne, Australia; ^2^National Vision Research Institute, Melbourne, Australia; ^3^Monash University, Department of Physiology, Melbourne, Australia; ^4^University of Melbourne, Biomedical Engineering, Melbourne, Australia; ^5^University of Melbourne, Melbourne, Australia; ^6^The University of Melbourne, Veterinary, Melbourne, Australia; ^7^The University of Melbourne, Biosciences, Melboune, Australia

##### **Correspondence:** Young Jun Jung (95jungy@gmail.com)

*BMC Neuroscience* 2020, **21(Suppl 1)**:P170

Orientation selectivity (OS) is a key feature of neurons in the mammalian primary visual cortex. In rodents and rabbits, these neurons are randomly distributed across V1 while in cats and all primates, cells with similar OS preferences cluster together into cortical columns. Could it be that mammals with smaller primary visual cortices, relatively undifferentiated cortices or poor-resolution vision are restricted to having salt-and-pepper OS maps? This is not true, because in gray squirrel, a highly visual rodent with good spatial resolution and a V1 that is highly differentiated, no clear functional organisation of OS preferences exists in V1. We do not know yet why the maps coding OS preferences are so radically different in rodents/rabbits compared to the clear similarities across other mammalian visual systems.

Several models of cortical OS maps have been created incorporating Hebbian plasticity, intracortical interactions and the properties of growing axons. But these models mainly focus on maps arising from intracortical interactions. Here we focus on two factors contributing to map formation: the topography of retina and phylogeny. One promising method of predicting whether or not a species has pinwheel maps is to look at the central-to-peripheral ratio (CP ratio) of retinal cell density. We have found that animals with high CP ratios (>7) have orientation columns while those with low CP ratios (<4) have random OS maps. We also investigated whether the development of OS maps is influenced by a genetic factor related to phylogeny. A problem with the existing literature is that OS maps have been investigated in only a small subset of mammals. We suggest that the rodents and rabbits might have lost the genetic capacity to develop OS maps, but that the mammalian line may have originally evolved with the genetic capacity to create orientation columns.

We studied a highly visual marsupial, the Tammar wallaby (*Macropus Eugenii*), which represents a phylogenetically distinct branch of mammals for which the orientation map structure is unknown. The topography of RCC’s in wallabies is very similar to cats and primates. They have a high density of RGC in the retinal specialization, indicated by a high CP ratio of 20. If orientation columns are the mammalian norm and if species with high CP ratios have OS maps, we would predict the existence of orientation columns in wallaby cortex. We used intrinsic optical imaging and multi-channel electrophysiology methods to examine the functional organization of the wallaby cortex. We found robust OS in a high proportion of cells in the primary visual cortex and clear orientation columns similar to those found in cats and primates but with bias towards vertical and horizontal preferences, suggesting lifestyle-driven variations. The findings suggest that orientation columns are the norm and it might be that the rodents and rabbits are unusual in terms of mammalian cortical architecture.

## P171 Neural routing: determination of the fastest flows and fastest routes in brain networks

### Paula Sanz-Leon^1^, Pierpaolo Sorrentino^2^, Fabio Baselice^3^, Rosaria Rucco^4^, Leonardo L Gollo^5^, James A Roberts^6^

#### ^1^University of Sydney, QIMR Berghofer, Brisbane, Australia; ^2^Aix-Marseille/ Parthenope University of Naples, Marseille, France; ^3^Parthenope University of Naples, Biomedical Engineering, Naples, Italy; ^4^Parthenope University of Naples, Motor Sciences and Wellness, Naples, Italy; ^5^QIMR Berghofer/ Monash University, Melbourne, Australia; ^6^QIMR Berghofer, Computational Biology, Brisbane, Australia

##### **Correspondence:** Paula Sanz-Leon (pmsl.academic@gmail.com)

*BMC Neuroscience* 2020, **21(Suppl 1)**:P171

**Background:** Large-scale brain networks [1] are characterized by global and local functional and structural metrics [2,3] that have furthered our understanding of brain function [4]. These metrics are based on the idea that information in a network flows along the shortest paths, either topological [5] or geometrical [6]. In this work, we propose two functional network connectivity measures based on the physical concept of flow [7,8], encompassing both geometrical and temporal aspects of neural activity. We term the first measure modal fastest flows, a time-averaged representation of the (fastest) flow lines revealing portions of physical space along which a particle (e.g., wave packet, information, spike) would travel at the maximal speed possible. The second measure, fastest neural routes, refers to a dense matrix where the weights are the average transit time a packet of information would take to travel from region ‘j’ to region ‘i’.

**Method:** (generation of fastest flow lines): We use our neural-flows toolbox [8,9] to derive flow fields from source-reconstructed MEG data. Fastest flow lines are then generated in 3 steps. First, we estimate flow vectors halfway between pairs of regions, transforming flow vectors into an edge property rather than a nodal property. Second, we trace a flow line starting from j, following the fastest flow to one of its nearest neighbours within a small spherical region. This process is done iteratively until reaching region i, and repeated for every possible region-pairwise combination. Flow lines are the sequences of maximal instantaneous speeds. Third, we average the values of each flow line to produce a matrix of fastest flows between pairs of regions.

**Results:** (modal fastest flows and fastest neural routes): We time-averaged the modal fastest flows (MFF), into a single matrix of conduction speeds. A comparison between functional connectivity derived from MEG timeseries and our MFF, indicates high similarity, quantified with the correlation matrix distance (cmd) [10] – 0 if matrices are equal, and 1 if completely different – and in this case cmd = 0.18. Paths highlighted by flow lines are not necessarily the shortest (in physical distance). Thus, we combine MFF with pairwise distance metrics to derive the fastest neural routes of information flow: the euclidean distance between pairs of regions, and the flow line lengths. Distributions of transit times are presented in Figure 1. Our MFF matrix combined with the fibre length of structural connectome, can be used as a first approximation of heterogeneous time delays (s) in brain networks.

**Fig. 1:**
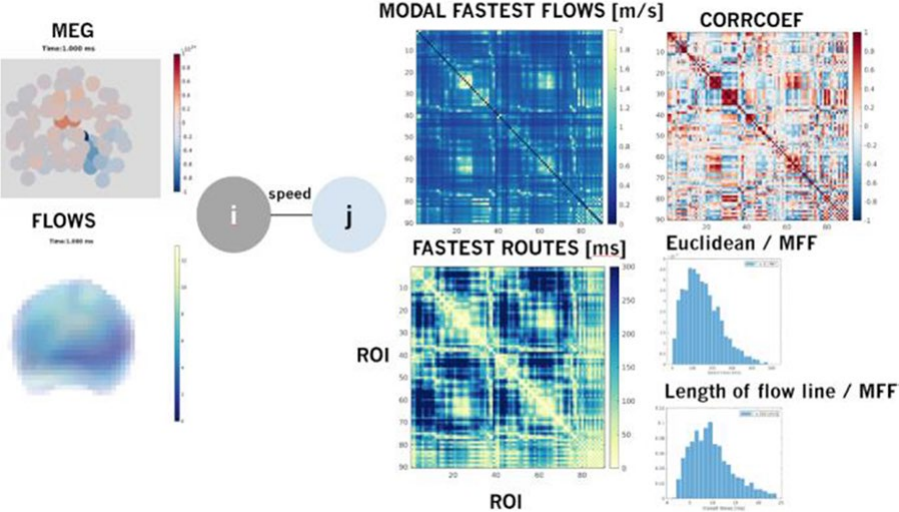
Flows are estimated from source-reconstructed MEG and defined at the sources. We then translate flows into edge propeties. Then, we derive the matrix of fastest flows (m/s), which strongly resembles traditional functional connectivity (Pearson’s correlation coefficients). To estimate the fastest routes (ms), we use different distance metrics

**References**

1. Bullmore E, Sporns O. Complex brain networks: graph theoretical analysis of structural and functional systems. Nature Reviews Neuroscience. 2009; 10(3): 186-98.
2. Rubinov M, Sporns O. Complex network measures of brain connectivity: uses and interpretations. Neuroimage. 2010; 52(3): 1059-69.
3. Zalesky A, Fornito A, Bullmore ET. Network-based statistic: identifying differences in brain networks. Neuroimage. 2010; 53(4): 1197-207.
4. Fornito A, Zalesky A, Breakspear M. The connectomics of brain disorders. Nature Reviews Neuroscience. 2015; 16(3): 159-72.
5. Fornito A, Zalesky A, Breakspear M. Graph analysis of the human connectome: promise, progress, and pitfalls. Neuroimage. 2013; 80: 426-44.
6. Seguin C, Van Den Heuvel MP, Zalesky A. Navigation of brain networks. Proceedings of the National Academy of Sciences. 2018; 115(24): 6297-302.
7. Townsend RG, Gong P. Detection and analysis of spatiotemporal patterns in brain activity. PLoS Computational Biology. 2018; 14(12): e1006643.
8. Sanz-Leon P, et al. Neuroimage toolbox *[in prep].*
9. Roberts JA, Gollo LL, Abeysuriya RG, Roberts G, Mitchell PB, Woolrich MW, Breakspear M. Metastable brain waves. Nature Communications. 2019; 10(1): 1-7.
10. Herdin M, Czink N, Ozcelik H, Bonek E. Correlation matrix distance, a meaningful measure for evaluation of non-stationary MIMO channels. In IEEE 61st Vehicular Technology Conference 2005; 1: 136-140.

## P172 Spatiotemporal brain waves on resting-state MEG data

### James Pang^1^, Paula Sanz-Leon^2^, Jonathan Hadida^3^, Leonardo Gollo^4^, Mark Woolrich^3^, James A Roberts^5^

#### ^1^QIMR Berghofer Medical Research Institute, Genetics & Computational Biology, Brisbane, Australia; ^2^QIMR Berghofer Medical Research Institute, Brain Modeling Group, Brisbane, Australia; ^3^University of Oxford, Oxford Centre for Human Brain Activity, Oxford, United Kingdom; ^4^Monash University, Melbourne, Australia; ^5^QIMR Berghofer, Computational Biology, Brisbane, Australia

##### **Correspondence:** James Pang (james.pang@qimrberghofer.edu.au)

*BMC Neuroscience* 2020, **21(Suppl 1)**:P172

Human brain function relies on the integration and coordination of neuronal activity on multiple scales. Several works have revealed that this is possible through spontaneous or evoked synchronization of activities of neural circuits in the brain, allowing spatially correlated patterns that propagate in time to emerge, known as brain waves [1]. These brain waves have been observed in empirical macroscopic and mesoscopic measurements [2,3] and computational brain network models [4], and have been shown to support various brain functions such as visual perception [5]. However, brain waves are rarely investigated in resting-state experimental settings (i.e., without performing an explicit task).

Here, we investigate large-scale spatiotemporal brain waves in resting-state human magnetoencephalography (MEG), which is becoming a popular imaging modality due to its high spatial and temporal resolution, enabling more accurate analysis of macroscopic brain waves. We use source reconstructed single-subject MEG data projected onto the cortical surface and then decompose the signal into various typical frequency bands from delta to gamma. We find that organized patterns of waves traveling in space and time exist in the resting-state data at the different frequency bands; an example of which is shown in the time snapshots of the alpha-filtered MEG signal in Figure 1A and the corresponding phase maps in Figure 1B. Using the methods in [3] for estimating instantaneous phase speeds, we find that, in general, waves with higher temporal frequencies tend to propagate more rapidly (Fig. 1C). In addition, the speeds match those in the literature using other modalities (e.g., electrocorticography in [2]), suggesting the reliability of our analyses. In summary, our work shows that macroscopic brain waves can be observed in resting-state MEG data even for a single subject, enabling the use of MEG alongside computational models in future investigations on how brain waves affect and relate to large-scale brain networks and the emergence of cognition and behavior.

**Fig. 1:**
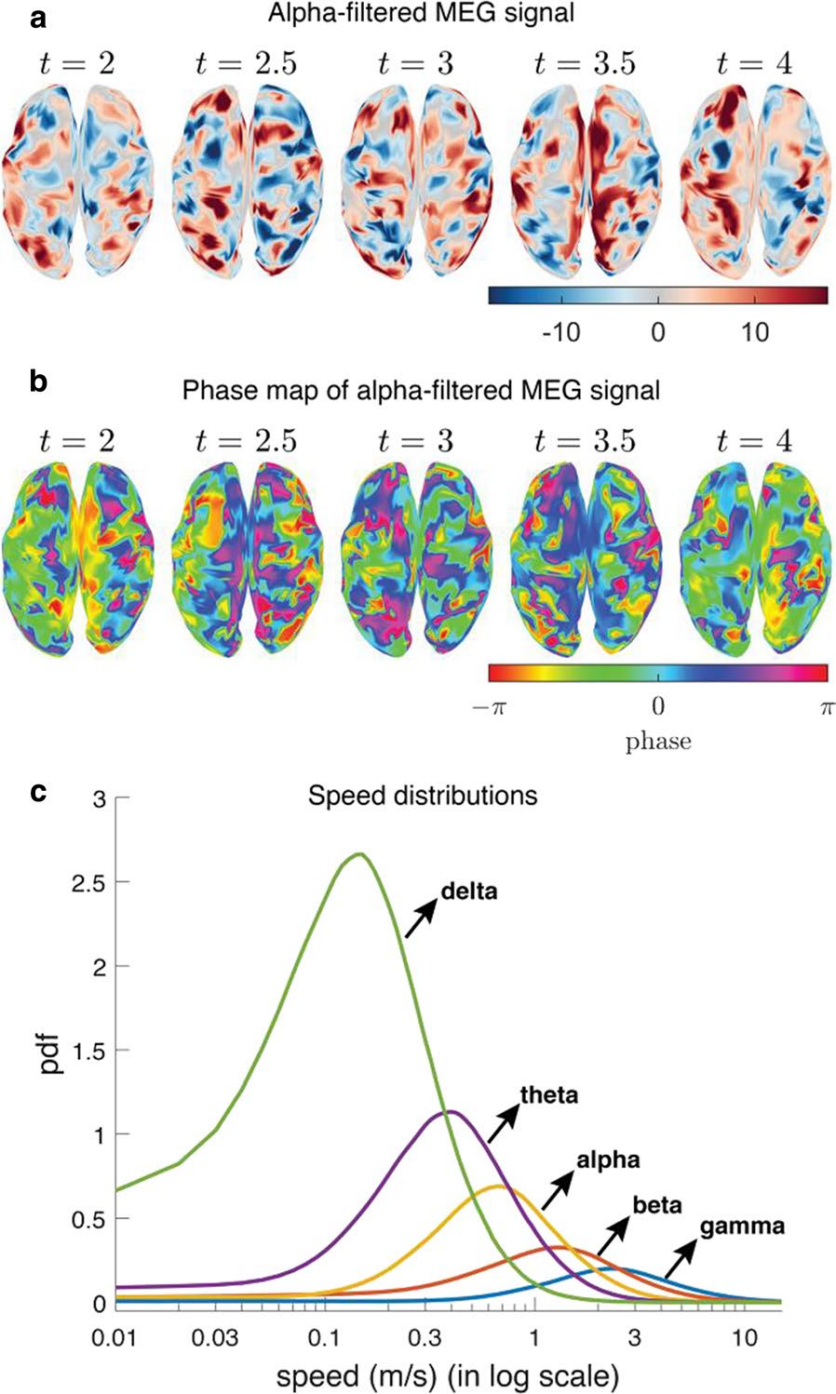
**A** Spatiotemporal variation of MEG signal filtered at the alpha frequency band. **B** Spatiotemporal phase maps of the signal in panel A. **C** Kernel density distribution of wave propagation speeds at different frequency bands

**References**

1. Muller L, Chavane F, Reynolds J, and Sejnowski TJ. Cortical travelling waves: Mechanisms and computational principles. Nature Reviews Neuroscience. 2018; 19(5): 255–268.
2. Zhang H, Watrous AJ, Patel A, and Jacobs J. Theta and alpha oscillations are traveling waves in the human neocortex. Neuron. 2018; 98: 1269–1281.
3. Rubino D, Robbins KA, and Hatsopoulos. Propagating waves mediate information transfer in the motor cortex. Nature Neuroscience. 2006; 9: 1549–1557.
4. Roberts JA, et al. Metastable brain waves. Nature Communications. 2019; 1056.
5. Zanos TP, Mineault PJ, Nasiotis KT, Guitton D, and Pack CC. A sensorimotor role for traveling waves in primate visual cortex. Neuron. 2015; 85(3): 615–627.

## P173 How energy constraints shape brain dynamics during hypoxia and epileptic seizures

### Shrey Dutta^1^, James A Roberts^2^

#### ^1^QIMR Berghofer Medical Research Institute, Faculty of Medicine, Brisbane, Australia; ^2^QIMR Berghofer, Computational Biology, Brisbane, Australia

##### **Correspondence:** Shrey Dutta (shrey.dutta@qimrberghofer.edu.au)

*BMC Neuroscience* 2020, **21(Suppl 1)**:P173

The brain consumes 20% of the body’s energy, 10 times more than predicted by its mass, which makes it highly sensitive to metabolic disturbances [1]. Asphyxia and epileptic seizures disrupt energy and oxygen availability in the brain, leading to pathological activity in the electroencephalogram (EEG) [2-4]. Modelling the bidirectional relationship between brain activity and energy resources is crucial to understand brain disorders where metabolic disturbances are implicated. Most models of brain activity do not explicitly include metabolic variables and so are unable to address dynamical constraints on energy resources. Here, we explore the roles of energy demand and energy supply in Hodgkin-Huxley neurons augmented with the energy resource dynamics of Na+/K+ pumps [4]. Using a small-scale network of excitatory and inhibitory neurons, we show that during high energy demand and low energy supply (extreme hypoxia) the model simulates scale-free burst suppression with asymmetric longer-duration bursts (Fig. 1) – similar to empirical EEG from infants recovering from hypoxia. During normal energy demand and low-to-moderate energy supply the model generates several types of epileptic seizures (Fig. 1). We also show multiple mechanisms for seizure terminations depending on the magnitude of hypoxia. Seizure termination during low energy supply is due to depletion of local energy resources, while during moderate energy supply ion (Na+& K+) imbalances terminate the seizure. This suggests that seizure termination due to lack of energy is a potential mechanism for postictal generalised EEG suppression. Our results unify burst suppression during hypoxia and epileptic seizures, and our modelling provides a general platform to study brain pathologies linked with metabolic disturbances.

**Fig. 1:**
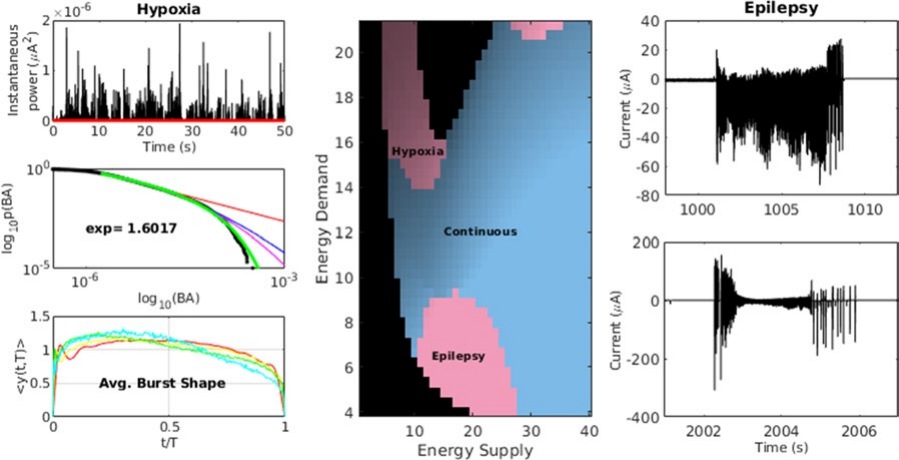
Unification in a single model of pathological patterns seen during baby hypoxia and epileptic seizures. Middle: Phase transitions between the regimes of hypoxia, healthy continuous activity and epilepsy in the energy demand-supply plane. Left: Model generated scale-free burst suppression in hypoxia showing power law regimes with asymmetric burst shape. Right: Model generated epileptic seizures

**References**

1. Raichle ME. The brain’s dark energy. Science-New York Then Washington-. 2006; 314(5803): 1249.
2. Roberts JA, Iyer KK, Finnigan S, Vanhatalo S, Breakspear M. Scale-free bursting in human cortex following hypoxia at birth. Journal of Neuroscience. 2014; 34(19): 6557-72.
3. Jirsa VK, Stacey WC, Quilichini PP, Ivanov AI, Bernard C. On the nature of seizure dynamics. Brain. 2014; 137(8): 2210-30.
4. Wei Y, Ullah G, Ingram J, Schiff SJ. Oxygen and seizure dynamics: II. Computational modeling. Journal of Neurophysiology. 2014; 112(2): 213-23.

## P174 Modal-polar representation of evoked response potentials

### Rawan El-Zghir^1^, Natasha Gabay^2^, Peter Robinson^3^

#### ^1^The University of Sydney, Sydney, Australia; ^2^University of Sydney, School of Physics/Complex Systems, Sydney, Australia; ^3^The University of Sydney, School of Physics, Sydney, Australia

##### **Correspondence:** Rawan El-Zghir (relz2030@uni.sydney.edu.au)

*BMC Neuroscience* 2020, **21(Suppl 1)**:P174

Event related potentials (ERPs) have grabbed the attention of neuroscientists as significant voltage fluctuations of the brain after a visual, auditory, or sensory stimulation to the nervous system. ERPs and correlation functions between signals at different points are key elements for investigating cognitive features and signal processing of the brain. To predict ERPs and correlation functions corresponding to distinct arousal states, we use a corticothalamic neural field theory which contains physiologically based parameters corresponding to different physical quantities. Within this framework, ERPs and correlation functions depend on transcendental equations which are not analytically tractable. We approximate the temporal transfer function in terms of poles or resonances to derive formulas for the ERP and correlation functions which greatly simplify their analytic forms. The dominant resonances of the system correspond to slow frequency, alpha, and beta frequencies. Our calculations are based on contour integration via the Cauchy-residue theorem that allows us to find explicit expressions for the ERP and correlation functions in terms of real and imaginary parts of the residues and poles. For each arousal state, we isolate the different resonances of the system and find that the eyes-closed wake state is distinguished by a more prominent alpha resonance compared to the eyes-open wake state, as expected. We found that 8 poles are sufficient to study the main dynamics of the system in the awake eyes-closed case (with around 3 % accuracy at the alpha peak) and 10 poles for the awake eyes-opened case (with around 2 % accuracy at the alpha peak). Similarly, we found that 8 poles are sufficient to reproduce ERPs corresponding to REM and S1 sleep stages, whereas only 6 poles are sufficient to study the dynamics of deeper sleep stages (slow wave sleep). This framework provides a physiologically-based tool which predicts ERPs and correlations corresponding to a given transfer function.

## P175 Analytic model for feature maps in the primary visual cortex

### Xiaochen Li, Peter Robinson

#### University of Sydney, School of Physics, Sydney, Australia

##### **Correspondence:** Xiaochen Li (morninglxc@hotmail.com)

*BMC Neuroscience* 2020, **21(Suppl 1)**:P175

This study proposes a compact analytic model that describes the orientation preference (OP) and ocular dominance (OD) maps of the primary visual cortex (V1) in hypercolumns, within which OP and OD are arranged as pinwheels and stripes. This model consists of two parts: (i) an OP operator, which uses a linear combination of weighted partial derivatives to incorporate the small-scale local neuron sensitivity to the preferred orientation of the visual inputs; and (ii) a receptive field (RF) operator, which models the spatial RF structure of V1 simple cell, and it is derived from finding the neural activities at arbitrary location with a directional anisotropic modulation of projections from neighboring neurons at scales of a few tenths of a millimetre. The parameters of the proposed OP-OD map model are tuned to maximize the neural response at the desired OP, by matching the width of OP tuning curves with experimental results. Moreover, we find that the weights of the partial derivatives in OP operator do not significantly affect the OP selectivity of the neuron, whereas the overall envelope of the RF operator does. This agrees with Hubel and Wiesel’s prediction [1], that orientation tuning width of V1 simple cell is related to the elongation of its RF.

The simplified OP-OD map is used to provide inputs to neural field theory (NFT) analysis of the approximate periodic OP-OD structure of V1. This is done by decomposing the OP-OD map representation in Fourier domain to generate a sparse set of Fourier coefficients. In addition, only the least number of coefficients, which are enough to preserves the basic spatial arrangement of OP-OD map, are passed to NFT for investigating OP map related neural activities. The decomposition is also applied on more realistic OP maps generated from published models and its properties are discussed.

**Reference**

1. Hubel DH, Wiesel TN. Receptive fields, binocular interaction and functional architecture in the cat’s visual cortex. The Journal of Physiology. 1962; 160(1): 106.

## P176 Using deep convolutional neural networks to visualise the receptive fields of high level visual cortical neurons

### Brett Schmerl^1^, Declan Rowley^2^, Elizabeth Zavitz^2^, Hsin-Hao Yu^2^, Nicholas Price^2^, Marcello Rosa^2^

#### ^1^University of South Australia, School of Information Technology and Mathematical Sciences, Adelaide, Australia; ^2^Monash University, Department of Physiology; Australian Centre of Excellence for Integrative Brain Function, Melbourne, Australia

##### **Correspondence:** Brett Schmerl (brett.schmerl@unisa.edu.au)

*BMC Neuroscience* 2020, **21(Suppl 1)**:P176

Understanding the image features that are encoded by neurons throughout the hierarchy of visual cortical areas, particularly in areas higher in the hierarchy that have more complex response properties than in V1, is a challenging yet fundamental goal in visual neuroscience that is often achieved by visualising their pattern of responses [1]. Visualising image features responsible for driving activity of individual units in a hierarchical system used for visual processing for the purposes of understanding the system’s functioning and information representation is also encountered in the study of deep convolutional neural networks.

In this study we train deep convolutional neural networks on spiking data recorded from individual neurons in a mid-tier visual area (the dorsomedial area, DM) of the anaesthetised marmoset monkey whilst the animal is presented with changing patterns of spatiotemporally white noise [2]. We show that convolutional neural networks are capable of learning statistically significant input-output relationships of these neurons and are thus able to perform classification of the spiking behaviour of the neuron given the stimuli. Furthermore, we applied deconvolutional techniques [3] used to visualise image features encoded by the convolutional model, thus allowing visualisation of input image features that are significant to determining spiking behaviour, by proxy, of the neuron. A comparison between the features recovered using this technique and those recovered by traditional methods of analysis is presented.

**References**

1. Jones JP, Palmer LA. The two-dimensional spatial structure of simple receptive fields in cat striate cortex. Journal of neurophysiology. 1987; 58(6): 1187-211.
2. Lui LL, Bourne JA, Rosa MG. Functional response properties of neurons in the dorsomedial visual area of New World monkeys (Callithrix jacchus). Cerebral Cortex. 2006; 16(2): 162-77.
3. Zeiler MD, Fergus R. Visualizing and understanding convolutional networks. In European conference on computer vision 2014; 818-833. Springer, Cham.

## P177 Modeling theta-band resonance in a neocortical circuit

### Rodrigo Pena^1^, Horacio Rotstein^2^

#### ^1^New Jersey Institute of Technology, Federated Department of Biological Sciences, Newark, New Jersey, United States of America; ^2^New Jersey Institute of Technology, Federated Department of Biological Sciences, NJIT/ Rutgers University, Newark, New Jersey, United States of America

##### **Correspondence:** Rodrigo Pena (pena@njit.edu)

*BMC Neuroscience* 2020, **21(Suppl 1)**:P177

The neocortex is a brain region responsible for many higher-order functions. Sensory signals arriving from different areas are integrated into the neocortex. Oscillations at certain frequency bands are believed to coordinate activity in many areas [1]. Resonance refers to the ability of a system to generate an amplified response if the input oscillation is tuned in a specific frequency band. Recent work showed the role of inhibition on the control of theta (4-11 Hz) oscillations through resonance [2]. By using optogenetic activation interneurons (inhibitory) induced theta-band-limited spiking in pyramidal (excitatory) neurons. On the other side, direct optogenetic activation of pyramidal cells did not generate any resonance pattern. Although it is clear that this phenomenon is neuron-specific, the network architecture responsible for the observed resonance and how this is related to the correct gating of the signals in such a network is currently unknown.

We address these issues by constructing a microcircuit biophysical minimal model of the neocortex using the Hodgkin-Huxley formalism [3]. We consider two pyramidal cells (PYR), one parvalbumin-positive (PV) interneuron, and one somatostatin-expressing (SOM) interneuron. These cells are interconnected with exponential decaying event-driven synapses where short-term depression/facilitation is present when appropriate [4]. Every cell spontaneously fires while receiving a noise input process to simulate *in vivo* synaptic barrage [5]. We apply periodic currents with different frequencies into PV cells and evaluate the PYR firing rate.

By applying oscillatory activation in PV, theta-band resonance was induced in PYRs whereas direct activation of PYRs did not show resonance, as experimentally reported. First, our results highlight the importance of post-inhibitory rebound in order to transfer signals from PV to PYR cells. Secondly, our results show that SOMs, adaptation, depression, and facilitation regulate these resonance effects. We explain these effects in terms of additional frequency filters that are added to the system: adaptation and facilitation act as a high-pass filter while depression acts as a low-pass filter. SOM cells regulate the low frequencies since they connect to other neurons through facilitation. In summary, when a current with a specific frequency is applied to the PV cells, this input signal is processed by a combination of filters, in the form of synapses and ionic currents, until a final output is produced from PYR cells. Our results highlight the importance of the combined activity of different neocortical cells in flexibly selecting inputs.

**Acknowledgments:** This work was supported by the National Science Foundation grant DMS-1608077 (HGR).

**References**

1. Buzsaki, G. Rhythms of the Brain. Oxford University Press, 2006.
2. Stark E, Eichler R, Roux L, Fujisawa S, Rotstein HG, Buzsáki G. Inhibition-induced theta resonance in cortical circuits. Neuron. 2013; 80(5): 1263-76.
3. Pospischil M, et al. Minimal Hodgkin–Huxley type models for different classes of cortical and thalamic neurons. Biological Cybernetics. 2008; 99(4-5): 427-41.
4. Tsodyks M, Pawelzik K, Markram H. Neural networks with dynamic synapses. Neural computation. 1998; 10(4): 821-35.
5. Destexhe A, Rudolph M, Fellous JM, Sejnowski TJ. Fluctuating synaptic conductances recreate in vivo-like activity in neocortical neurons. Neuroscience. 2001; 107(1): 13-24.

## P178 Neuronal resonance may not be apparent, but still present, for realistic input signals using standard impedance measurements

### Rodrigo Pena^1^, Ulises Chialva^2^, Horacio Rotstein^3^

#### ^1^New Jersey Institute of Technology, Federated Department of Biological Sciences, Newark, New Jersey, United States of America; ^2^Universidad Nacional del Sur, Departmento de Matemática and CONICET, Bahía Blanca, Argentina; ^3^New Jersey Institute of Technology, Federated Department of Biological Sciences, NJIT/ Rutgers University, Newark, New Jersey, United States of America

##### **Correspondence:** Rodrigo Pena (pena@njit.edu)

*BMC Neuroscience* 2020, **21(Suppl 1)**:P178

The impedance of a neuron reflects the frequency-dependent input-output relationships and is typically computed using sinusoidal inputs [1]. Oscillatory currents with gradually increasing frequencies or broadband noise have been used so the response is equally tested at all frequencies [2,3]. The ability of a neuron to amplify its response to specific non-zero input frequencies (resonance) has been questioned [4]. The question arises of whether the differences in the type of response (low- vs. band-pass) can be ascribed to the type of input and not necessarily to the details of the frequency content. More specifically, whether and how the use of different types of biophysically plausible periodic inputs (e.g., sinusoidal, synaptic-like) produce qualitatively different frequency-dependent input-output curves. We address these issues by injecting different types of inputs to biophysically plausible neuronal models including currents that are known to produce subthreshold (membrane potential) resonance in response to sinusoidal inputs. All input signals have the same amplitude and frequency content, but the frequencies may come in “different order” (e.g., monotonically increasing, randomly distributed or “shuffled”). The waveforms include sinusoidal, square-waves and synaptic-like functions. The impedance is computed either (i) as the ratio of the Fourier transforms (FT) of the voltage (*V*) and the current (*I*) or (ii) by the difference in the amplitude envelope responses normalized by the input amplitude.

We show that if the inputs involve abrupt changes (e.g., square-waves, synaptic-like), transients contribute to the output signal, which qualitatively modify the impedance profile. This can cause a mismatch between the impedance computed using (i) vs. (ii) given that the FT captures these transients as higher harmonics (Fig. 1). Therefore, a resonance observed in the response pattern may not be captured by the impedance using standard definitions and may require a more careful analysis. Furthermore, when input frequencies are presented in a “shuffled” order, these transient effects produce responses with additional amplification to the higher frequency responses.

**Fig. 1:**
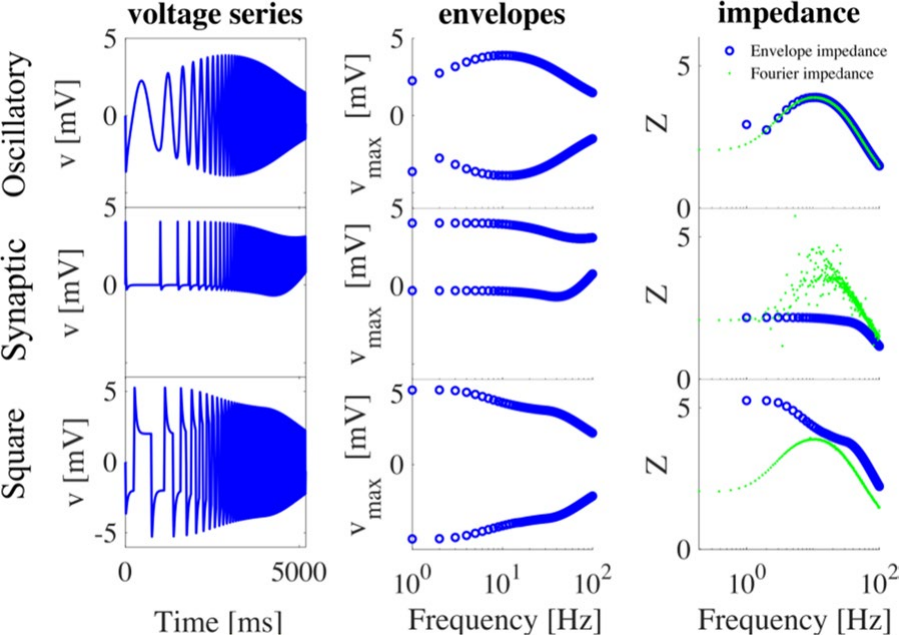
Three different inputs applied to the linear model. From top to bottom: oscillatory input, synaptic input, and pulse input. First column: voltage series. Second column: voltage-envelopes. Third column: Impedance profile computed from the Fourier transform method and from the envelope method. The figure shows how different the impedance profile can be even within the same input frequency content

Our results highlight both the flexibility and limitations of the impedance profile measurements and demonstrate that resonance may be present in neuronal systems, but are not apparent unless one uses the appropriate types of inputs and output metrics. Furthermore, our results question the ability of the standard impedance metric to make predictions for a more general class of inputs.

**Acknowledgments:** This work was supported by the National Science Foundation grant DMS-1608077 (HGR).

**References**

1. Hutcheon B, Yarom Y. Resonance, oscillation and the intrinsic frequency preferences of neurons. Trends in Neurosciences. 2000; 23(5): 216-22.
2. Kispersky TJ, Fernandez FR, Economo MN, White JA. Spike resonance properties in hippocampal O-LM cells are dependent on refractory dynamics. Journal of Neuroscience. 2012; 32(11): 3637-51.
3. Schreiber S, Erchova I, Heinemann U, Herz AV. Subthreshold resonance explains the frequency-dependent integration of periodic as well as random stimuli in the entorhinal cortex. Journal of Neurophysiology. 2004; 92(1): 408-15.
4. Zemankovics R, Káli S, Paulsen O, Freund TF, Hájos N. Differences in subthreshold resonance of hippocampal pyramidal cells and interneurons: the role of h‐current and passive membrane characteristics. The Journal of Physiology. 2010; 588(12): 2109-32.

## P179 Synaptic short-term plasticity and temporal filters: interplay of synaptic and postsynaptic dynamics

### Yugarshi Mondal^1^, Rodrigo Pena^2^, Horacio Rotstein^3^

#### ^1^Stony Brook University, Applied Math, Stony Brook, New York, United States of America; ^2^New Jersey Institute of Technology, Federated Department of Biological Sciences, Newark, New Jersey, United States of America; ^3^New Jersey Institute of Technology/ Rutgers University, Federated Department of Biological Sciences, Newark, New Jersey, United States of America

##### **Correspondence:** Yugarshi Mondal (yugarshi.mondal@stonybrook.edu)

*BMC Neuroscience* 2020, **21(Suppl 1)**:P179

Short-term plasticity (STP) is the process by which a synapse changes its efficacy in a history-dependent manner. It is hypothesized that STP’s information processing capabilities are connected to the way it implements temporal filtering in response to a sequence of presynaptic events [1,2]. Depression (STD) and facilitation (STF) refer to the decrease and increase (e.g., Fig. 1A-B) of the efficacy in the synaptic response to a presynaptic spike as compared to the previous one. Sequences of presynaptic spikes create synaptic *temporal filters* (TFs) (e.g., Fig. 1). STD and STF lead to low-pass and high-pass (Figs. 1A- B) TFs. The presence of both STD and STF may lead to band-pass TFs (Fig. 1C). These synaptic TFs are communicated to the postsynaptic membrane potential. However, it is unknown whether the properties of the postsynaptic TFs (PSTFs) are inherited from the synaptic TFs or there is additional processing involved.

We report on the results of a computational study aimed at identifying the types of TFs and PSTFs in response to periodic presynaptic inputs and their dependence on the biophysical and dynamic properties of the participating components. We implement biophysically plausible (conductance-based) computational models of a synapse with STP [3], which drives a post-synaptic cell. We characterize the TFs that arise in the synapse. We determine the conditions under which synaptic low-, high- and band-pass filters arise in terms of the STP and synaptic time constants and the presynaptic input frequency. We also determine how the long-term time constants of the synaptic envelope responses (Fig. 1, black curves) depend on the STP time constants, which operate at the single event level. While the envelopes for the low- and high-pass TFs have a single time constant that depends on depression or facilitation time constants, accordingly, the envelope for the band-pass TF has three time constants that depends on the time constants for (i) depression only, (ii) facilitation only, and (iii) a combination of both. This is in contrast to the naïve expectation that there would be only two time constants involved (depression and facilitation only). We then extend our study to include the postsynaptic cell. We identify and characterize the different types of PSTFs in terms of the properties of the input TF and the properties of the receiving cell. We show that while under certain conditions, the PSTFs are qualitatively a copy of the synaptic TFs and share many of the TF’s dynamic properties, in other biophysical conditions, the PSTF exhibit a higher degree of complexity, which involve a multiplicity of time scales (e.g., depression/facilitation, synaptic, membrane, ionic currents, summation).

**Fig. 1:**
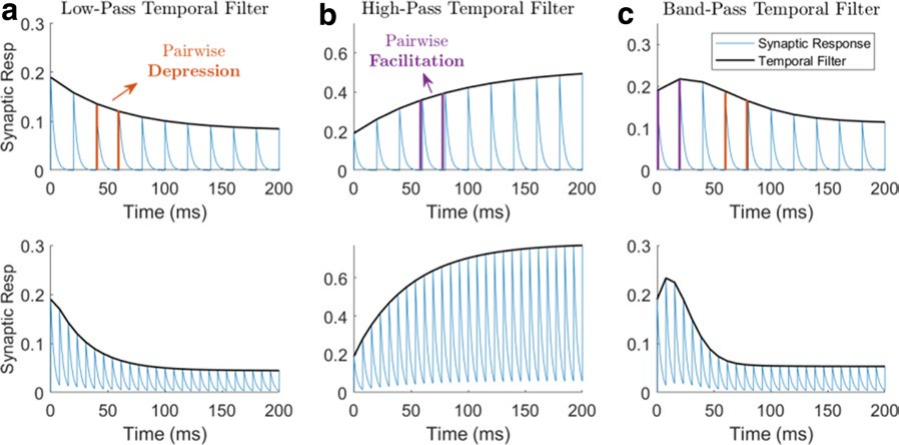
Representative examples of TFs generated by STP. **A** low-pass TF. **B** high-pass TF. **C** band-pass TF. Presynaptic input frequencies are 50 Hz (Top) and 150 Hz (Bottom). Examples of pairwise depression and facilitation are shown in orange and purple. The black envelope curves represent the TFs

Overall, our results highlight the complexity of TFs and PSTFs, which reflects the complexity of the underlying interactions. This has implications for the understanding of network filters and the development of effective synaptic decoding algorithms.

**Acknowledgments:** Funded in part by a grant (DMS-1608077 to HGR) and a Graduate Research Fellowship (to YM) both from the NSF.

**References**

1. Zucker RS, Regehr WG. Short-term synaptic plasticity. Annual review of physiology. 2002; 64(1): 355-405.
2. Fortune ES, Rose GJ. Short-term synaptic plasticity as a temporal filter. Trends in neurosciences. 2001; 24(7): 381-5.
3. Tsodyks M, Pawelzik K, Markram H. Neural networks with dynamic synapses. Neural computation. 1998; 10(4): 821-35.

## P180 Mean field theory inference and learning in networks with stochastic natural exponential family neurons

### Ivan Davidovich^1^, Benjamin Dunn^2^, John Hertz^3^, Yasser Roudi^1^

#### ^1^NTNU, Kavli Institute for Systems Neuroscience, Trondheim, Norway; ^2^NTNU, Department of Mathematical Sciences, Trondheim, Norway; ^3^Nordita and University of Copenhagen, Copenhagen, Denmark

##### **Correspondence:** Ivan Davidovich (ivan.a.davidovich@ntnu.no)

*BMC Neuroscience* 2020, **21(Suppl 1)**:P180

The advent of experimental techniques that allow for the simultaneous recording of an unprecedented number of neurons has created a necessity for analysis tools that can handle very large datasets. Non-equilibrium models from statistical mechanics such as the kinetic Ising model have established themselves as natural tools for studying these types of complex networks and datasets. The applicability of the kinetic Ising model is however limited by the binary nature of its neurons. To alleviate this constraint, we extend previous analyses based on statistical mechanics to dynamical models of networks in which the state of the neurons can take values according to distributions in the mono-parametric natural exponential family. Using a mean field approach, we obtain both dynamical TAP equations for the expected activity of these states when the connectivity of the network is known as well as a naive mean field estimation of the connectivity when the activity of the neurons has been observed. We test the validity and accuracy of our analytical results by applying them to simulations, obtaining good agreement within specific regions of parameter space. For the inference of the network’s connectivity we compare to exact learning via gradient ascent, obtaining excellent agreement for networks of Gaussian neurons and small discrepancies for Poisson networks.

## P181 Long-term turnover dynamics in area CA1 of hippocampus are consistent with plasticity of non-spatial inputs

### Federico Devalle, Alex Roxin

#### Centre de Recerca Matemàtica, Barcelona, Spain

##### **Correspondence:** Federico Devalle (federico.devalle@gmail.com)

*BMC Neuroscience* 2020, **21(Suppl 1)**:P181

Hippocampal representation of space over long time scales is dynamic [1]. Longitudinal calcium imaging of CA1 neurons of mice repeatedly traversing the same environment over weeks exhibits turnover: only a small subset of pyramidal cells are active over the entire course of the experiment, while most of the population drops in and out of the ensemble representation of the environment. Yet, whenever active, place cells typically retain their place field location.

Here, we hypothesize that cells turnover in CA1 is due to the interplay between two types of synaptic inputs to CA1 pyramidal neurons: a stable spatial input from CA3 place cells, and a time-varying non-spatial input. We first test this hypothesis by fitting a statistical model to CA1 calcium imaging data of mice repeatedly visiting the same familiar track over the course of two weeks [2]. In the statistical model, cells are described as threshold units, active when the sum of the spatial and non-spatial inputs they receive is larger than a threshold. Spatial (stable) and non-spatial (time-varying) inputs are modeled as gaussian random variables. The statistical model has three parameters: the relative width of the distribution of spatial and non-spatial inputs, the neuronal threshold, and the auto-correlation of the time-varying non-spatial inputs. By fitting those parameters, the model quantitatively describes all relevant turnover statistics observed in the experimental data: the probability that a cell active on one day will be active on subsequent days, the distribution of the total number of sessions in which cells are active, and cells survival probability.

Based on these results, we then propose a spiking network model of the hippocampus which accounts for turnover dynamics. In the spiking network model, CA1 pyramidal cells integrate spatially-modulated synaptic inputs from CA3 place cells, inhibitory inputs from CA1 interneurons, and non-spatial inputs from a layer of cortical neurons. Integration of a large number of random CA3 spatial inputs generates spatially-modulated subthreshold voltage in CA1 pyramidal cells, and non-spatial inputs modulate the excitability of CA1 cells. While spatial connections are stable, a fraction of the non-spatial connections are rewired over time. Rewiring non-spatial connections shifts cells excitability, and hence determines whether a cell is active and participate to the ensemble representation at a given time or is silent, generating turnover. Importantly, whenever cells are active, their place field is in the same location of the environment, consistent with experimental findings.

By adjusting the relative width of the distributions of spatial and non-spatial inputs (which can be calculated analytically) and the auto-correlation of non-spatial inputs, the spiking network model accurately fits all relevant turnover statistics observed in the experimental data. Finally, introducing (weak) correlations among CA3 spatial inputs, the model is also able to capture the distribution of spatial information observed in CA1 pyramidal cells.

**References**

1. Ziv Y, Burns LD, Cocker ED, Hamel EO, Ghosh KK, Kitch LJ, El Gamal A, Schnitzer MJ. Long-term dynamics of CA1 hippocampal place codes. Nature Neuroscience. 2013; 16(3): 264.
2. Rubin A, Geva N, Sheintuch L, Ziv Y. Hippocampal ensemble dynamics timestamp events in long-term memory. Elife. 2015; 4: e12247.

## P182 Intermittent synchronization in a pyramidal-interneuron gamma (PING) network

### Quynh-Anh Nguyen^1^, Leonid Rubchinsky^2^

#### ^1^Indiana University Purdue University Indianapolis, Mathematical Sciences, Indianapolis, Indiana, United States of America; ^2^Indiana University Purdue University Indianapolis and Indiana University School of Medicine, Department of Mathematical Sciences and Stark Neurosciences Research Institute, Indianapolis, Indiana, United States of America

##### **Correspondence:** Quynh-Anh Nguyen (qpnguyen@iu.edu)

*BMC Neuroscience* 2020, **21(Suppl 1)**:P182

Synchronization in neural system plays an important role in many brain functions such as perception and memory. Abnormal synchronization can be observed in neurological disorders such as Parkinson’s disease, schizophrenia, autism, and addiction. When the coupling strength is moderate, the synchronization is highly intermittent even in a short time scale. That is, a system exhibits intervals of synchronization followed by intervals of desynchronization. Thus, neural circuits dynamics may show different distributions of duration of desynchronization even if the synchronization strength is similar.

In general, some partially synchronized systems can exhibit a few but long desynchronized intervals while other systems can yield many but short desynchronized intervals. Experimental data thus far has shown that neural synchronization follows the latter trend in either healthy or diseased brains [1-3]. It was suggested that there are functional advantages of circuitry with many short durations of desynchronization [4].

In this study, we use a conductance-based PING network to study neural synchronization specifically in the low gamma band. Gamma frequency rhythm is known to play important role in cognitive functions such as percept formation, object representation, learning, and memory. Several experimental studies indicate that while both healthy and diseased brains have many short intervals of desynchronization, there are differences in their distributions of desynchronization durations [2,3,5]. This study explores the cellular and synaptic effects on the temporal patterning of the partially synchronized model gamma rhythms and considers potential functional implications of different temporal patterns.

**Acknowledgements:** This work was supported by NSF grant DMS 1813819.

**References**

1. Ahn S, Rubchinsky LL. Short desynchronization episodes prevail in synchronous dynamics of human brain rhythms. Chaos. 2013; 23(1): 013138.
2. Malaia EA, Ahn S, Rubchinsky LL. Dysregulation of temporal dynamics of synchronous neural activity in adolescents on autism spectrum. Autism Research. 2020; 13(1): 24-31.
3. Ahn S, Rubchinsky LL, Lapish CC. Dynamical reorganization of synchronous activity patterns in prefrontal cortex–hippocampus networks during behavioral sensitization. Cerebral Cortex. 2014; 24(10): 2553-61.
4. Ahn S, Rubchinsky LL. Potential mechanisms and functions of intermittent neural synchronization. Frontiers in Computational Neuroscience. 2017; 11: 44.
5. Ahn S, Zauber SE, Worth RM, Witt T, Rubchinsky LL. Neural synchronization: Average strength vs. temporal patterning. 2018.

## P183 Universal fingerprints of slow-wave activity in in vivo, in vitro and in silico cortical networks

### Alessandra Camassa^1^, Andrea Galluzzi^2^, Miguel Dasilva^1^, Beatriz Rebollo^1^, Maurizio Mattia^2^, Maria V Sanchez-Vives^3^

#### ^1^Institut d’Investigacions Biomèdiques August Pi i Sunyer, Systems Neuroscience, Barcelona, Spain; ^2^Istituto Superiore di Sanitá, National Center for Radiation Protection and Computational Physics, Rome, Italy; ^3^Institut d’Investigacions Biomèdiques August Pi i Sunyer, ICREA, Systems Neuroscience, Barcelona, Spain

##### **Correspondence:** Alessandra Camassa (ale.camassa22@gmail.com)

*BMC Neuroscience* 2020, **21(Suppl 1)**:P183

The cerebral cortex as a structured network is able to spontaneously express different types of dynamics that are continuously changing over time according to the ongoing brain state. Transitions across brain states correlate with changes in network excitability and functional connectivity giving rise to a wide repertoire of spatiotemporal patterns of neuronal activity [1]. The quasi-periodic occurrence of travelling waves - namely slow-wave activity (SWA) - characterizes the cortical networks under unconscious brain states. The spatiotemporal patterns generated under SWA are shaped by the structure and excitability of the underlying network [2,3]. Thus, the emergent wavefronts portray the characteristics of the dynamical regime under which they have been spawned. Here we aimed to develop novel analytical methods to capture wave propagation features and to identify the universal fingerprints of the cortical network activity generated by different preparations all spontaneously expressing SWA, in order to gain a deeper understanding of functional mechanisms underlying the cortical network organization. To do so, we studied the spatiotemporal dynamics of the cortex under SWA in three different frameworks: *in vivo*, performing extracellular recordings of cortical activity in deeply anesthetized mice with a superficial multielectrode array; *in vitro*, recording the electrophysiological signals from cortical slices cut from ferret visual cortex; *in silico*, in a simulated multimodular network of spiking neurons [2,4]. We studied network dynamics by characterizing the spatiotemporal patterns of propagation of the activation wavefronts developing a phase-based method that allow an accurate reconstruction of the waves travelling across the cortex both in experimental and simulated data [5]. We complemented the study of network dynamics with the computation of network synchronization over time, evaluating the variability of ongoing synchrony fluctuations that entail dynamically changing states, in our case Up and Down states of SWA. Finally, we evaluated the dynamical richness of the cortical activity by estimating the dimensionality of the system dynamics over time. We adopted an approach drawn from experimental fluid dynamics in physics [6]. Applying an empirical eigenfunction approach by means of the algorithm of Singular Value Decomposition (SVD) it is possible to quantify the instantaneous energy of the system and its effective dimension, and to study the evolution of the system dimension over time as well as its dependence on the structure and on the dynamical state of the system. In this way, we were able to compare the mechanistic underpinning of SWA when the intact cortex is functionally disconnected (*in vivo* under deep anesthesia) and when it is anatomically disconnected from the rest of the brain (*in vitro* in cortical slices), and finally exploiting the model, to emphasize the universal nature of this slow rhythm highlighting both the differences and similarities between experimental conditions.

**Acknowledgements**: Funded by the EU H2020 Research and Innovation Programme, Grant 720270 (HBP SGA2).

**References**

1. Stitt I, Hollensteiner KJ, Galindo-Leon E, Pieper F, Fiedler E, Stieglitz T, Engler G, Nolte G, Engel AK. Dynamic reconfiguration of cortical functional connectivity across brain states. Scientific Reports. 2017; 7(1): 1-4.
2. Capone C, et al. Slow waves in cortical slices: how spontaneous activity is shaped by laminar structure. Cerebral Cortex. 2019; 29(1): 319-35.
3. Barbero-Castillo A, et al. Proceedings# 31: cortical network complexity under different levels of excitability controlled by electric fields. Brain Stimulation: Basic, Translational, and Clinical Research in Neuromodulation. 2019; 12(2): e97-9.
4. Mattia M, Pani P, Mirabella G, Costa S, Del Giudice P, Ferraina S. Heterogeneous attractor cell assemblies for motor planning in premotor cortex. Journal of Neuroscience. 2013; 33(27): 11155-68.
5. Muller L, Reynaud A, Chavane F, Destexhe A. The stimulus-evoked population response in visual cortex of awake monkey is a propagating wave. Nature communications. 2014; 5(1): 1-4.
6. Schiff SJ, Huang X, Wu JY. Dynamical evolution of spatiotemporal patterns in mammalian middle cortex. Physical Review Letters. 2007; 98(17): 178102.

## P184 Selection of ictogenic zones in epilepsy surgery using computational models

### Petroula Laiou^1^, Eugenio Abela^1^, Mark Richardson^1^, Marc Goodfellow^2^, Eleftherios Avramidis^3^, Marinho Lopes^4^, Michael Mueller^5^, Ozgur Akman^6^, Christian Rummel^7^, Kaspar Schindler^5^

#### ^1^King’s College London, Institute of Psychiatry, Psychology and Neuroscience, London, United Kingdom; ^2^University of Exeter, Living Systems Institute, Exeter, United Kingdom; ^3^University of Cambridge, Research Computing Services, Cambridge, United Kingdom; ^4^University of Cardiff, Brain Research Imaging Centre, Cardiff, United Kingdom; ^5^Bern University Hospital, Department of Neurology, Bern, Switzerland; ^6^University of Exeter, College of Engineering, Mathematics and Physical Sciences, Exeter, United Kingdom; ^7^Bern University Hospital, Support Center for Advanced Neuroimaging, University Institute for Diagnostic and Interventional Neu, Bern, Switzerland

##### **Correspondence:** Petroula Laiou (petroula.laiou@kcl.ac.uk)

*BMC Neuroscience* 2020, **21(Suppl 1)**:P184

Epilepsy surgery is a therapeutic option that can alleviate seizures in people with refractory epilepsy. The aim of surgery is to remove the epileptogenic zone, i.e. the brain tissue that is considered indispensable for seizure generation [1,2]. During the presurgical evaluation clinical teams integrate diverse information and if seizure freedom is achieved after surgery, it is considered that the epileptogenic zone has been resected [1]. Unfortunately, the rates of post-surgical seizure freedom are currently sub-optimal, due to the lack of understanding the seizure generating mechanism and assessing the consequences of surgical resections.

Recent computational studies in large-scale brain networks [3-5] have been developed with the aim to inform epilepsy surgery. Here, we use a modelling framework that allows to investigate how sets of nodes contribute to the seizure generating capability (i.e. ictogenicity) of a network [6]. In particular, we use a measure called Set Ictogencity (SI) in order to quantify the contribution of a set of nodes to ictogenicity. We use artificial networks with various topologies and examine how SI varies across different sets of nodes. In networks with small size we compute SI for all possible sets of nodes and show that the ictogenicity across sets depends on network topology. However, in large networks the computation of SI of all possible sets is a combinatorial problem that becomes intractable. Therefore, we combine computational models with a genetic algorithm to search for sets with minimal size that contribute to the seizure generation.

We demonstrate the potential applicability of these methods by identifying optimal set of nodes to resect in brain networks derived from a cohort of 20 people who underwent epilepsy surgery. In addition, we show that this modelling framework has the potential to assist epilepsy surgery by suggesting alternative resection sites as well as allowing the avoidance of brain regions that should not be resected.

**References**

1. Rosenow F, Lüders H. Presurgical evaluation of epilepsy. Brain. 2001; 124: 1683–700.
2. Nowell M, Miserocchi A, McEvoy AW, Duncan JS. Advances in epilepsy surgery. Journal of Neurology Neurosurgery & Psychiatry. 2014; 85: 1273–9.
3. Goodfellow M, Rummel C, Abela E, Richardson MP, Schindler K, Terry JR. Estimation of brain network ictogenicity predicts outcome from epilepsy surgery. Scientific Reports. 2016; 6: 29215.
4. Steimer A, Müller M, Schindler K. Predictive modeling of EEG time series for evaluating surgery targets in epilepsy patients. Human Brain Mapping. 2017; 38(5): 2509-31.
5. Müller M, et al. Linear and nonlinear interrelations show fundamentally distinct network structure in preictal intracranial EEG of epilepsy patients. Human Brain Mapping. 2020; 41(2): 467-83.
6. Laiou P, et al. Quantification and selection of ictogenic zones in epilepsy surgery. Frontiers in Neurology. 2019; 10: 1045.

## P185 Higher-order interactions induced by strong shared inputs

### Safura Rashid Shomali^1^, Seyyed Nader Rasuli^2,3^, Hideaki Shimazaki^4^

#### ^1^Institute for Research in Fundamental Sciences, School of Cognitive Sciences, Tehran, Iran; ^2^University of Guilan, Department of Physics, Rasht, Iran; ^3^Institute for Research in Fundamental Sciences, School of Physics, Tehran, Iran; ^4^Hokkaido University, Center for Human Nature, Artificial Intelligence and Neuroscience, Hokkaido, Japan

##### **Correspondence:** Safura Rashid Shomali (safura@ipm.ir)

*BMC Neuroscience* 2020, **21(Suppl 1)**:P185

Experimental studies demonstrated that neural populations exhibit correlated spiking activity that goes beyond pairwise correlations and involves higher-order interactions [1-5]. These higher-order interactions are known to encode stimulus information or the internal state of the brain [1-5]. However, the origin of this population activity and types of presynaptic neurons inducing the higher-order interactions remain unclear. Here we investigate how the interactions [6] among groups of 3, 4, and then N neurons emerge, when they receive common inputs on top of independent noisy background inputs, assuming simple connecting motifs. Given Poissonian common inputs, we calculate the neural interactions among clusters of neurons in a small time-window for the limit of the strong common input’s amplitude. When 2 or 3 neurons share excitatory/inhibitory common inputs, their pairwise and triple-wise interactions are well explained as functions of their baseline spontaneous rate, and the common-input’s rate [7].

We analytically solve the interactions for a cluster of more than 3 neurons when all of them share strong excitatory/inhibitory common input. Then, extending our analysis to the arbitrary number of N neurons we show that the N-th order interaction among neurons is still a simple function of the postsynaptic and common input rates. However, in larger populations, the N-th order interaction more strongly depends on the spontaneous rate of postsynaptic neuron rather than input rate. We also observe that larger number of neurons induce stronger magnitude of interactions, regardless of interaction’s sign. Moreover, shared excitatory inputs to all neurons always generate interactions with positive sign, while shared inhibitory inputs induce interactions with oscillatory signs with respect to N. Finally, we obtain the analytic result when excitatory or inhibitory inputs are shared among N-1 out of all N neurons: surprisingly, the N-th order interactions exhibit signs opposite to those found when the common inputs is shared by all N neurons.

In all mentioned cases, when the spontaneous activity of postsynaptic neurons is low, excitatory inputs can generate strong positive/negative higher-order interactions, whereas for high spontaneous activity, inhibitory neurons can induce large absolute values of higher-order interactions. These results are valid for any neuron model and solely based on the assumption of strong common inputs given to neurons. Since cortical, subcortical, and retinal neurons mostly exhibit spontaneous activity less than λ=40 Hz, for small time-window of Δ=5ms, these neurons are in low spontaneous regime i.e. λΔ < 0.2. Therefore we suggest that the significant higher-order interactions observed in retina, hippocampus, and cortices reveal that motifs of strong excitatory rather than inhibitory shared inputs are present and dominant there.

Finally, we draw a table that links the strength of interactions and their signs to motifs, both for low and high spontaneous activity regimes. So based on interactions obtained from experimental data, it is possible to predict the underlying motif behind it. For example, for a specific experiment done in the hippocampal CA3 region [8], the observed negative 3rd-order, positive 4th-order, and negative 5th-order interactions leads us to the architecture of excitatory to pairs, that can generate such interactions simultaneously.

**References**

1. Ohiorhenuan IE, et al. Sparse coding and high-order correlations in fine-scale cortical networks. Nature. 2010; 466: 617–621.
2. Montani F, et al. The impact of high-order interactions on the rate of synchronous discharge and information transmission in somatosensory cortex. Philosophical Transactions of the Royal Society A: Mathematical, Physical and Engineering Sciences. 2009; 367(1901): 3297-310.
3. Yu S, et al. Higher-order interactions characterized in cortical activity. The Journal of Neuroscience. 2011; 31: 17514–17526.
4. Shimazaki H, et al. State-space analysis of time varying higher-order spike correlation for multiple neural spike train data. PLoS Computational Biology. 2012; 8: e1002385.
5. Ganmor E, Segev R, Schneidman E. Sparse low-order interaction network underlies a highly correlated and learnable neural population code. Proceedings of the National Academy of sciences. 2011; 108(23): 9679-84.
6. Nakahara H, Amari S. Information-geometric measure for neural spikes. Neural Computation. 2002; 14: 2269–2316.
7. Shomali SR, Ahmadabadi MN, Rasuli SN, Shimazaki H. Uncovering Network Architecture Using an Exact Statistical Input-Output Relation of a Neuron Model. bioRxiv. 2018: 479956.
8. Shimazaki H, Sadeghi K, Ishikawa T, Ikegaya Y, Toyoizumi T. Simultaneous silence organizes structured higher-order interactions in neural populations. Scientific Reports. 2015; 5(1): 1-3.

## P186 Inhibitory neurons locate at a center of effective cortical networks, and have high ability to control other neurons.

### Motoki Kajiwara^1^, Masanori Shimono^2^

#### ^1^Kyoto University, Kyoto, Japan; ^2^Kyoto University, Graduate School of Medicine and Faculty of Medicine, Kyoto, Japan

##### **Correspondence:** Motoki Kajiwara (mokki.70878@gmail.com)

*BMC Neuroscience* 2020, **21(Suppl 1)**:P186

The brain is a network system in which excitatory and inhibitory neurons keep the activity balanced in the highly non-uniform connectivity pattern of the microconnectome. It is well known that the relative percentage of inhibitory neurons is much smaller than excitatory neurons. So, in general, how the inhibitory neurons can keep the balance with the surrounding excitatory neurons is an important question.

This study simultaneously recorded electric signals from ~1000 neurons from seven acute brain slices of mice with a MEA (multi-electrode array) to analyze the network architectures of cortical neurons. Subsequently, we analyzed the spike data to reconstruct the causal interaction networks between the neurons from their spiking activities. The utilized analysis mainly consists of the following three steps: first, Transfer Entropy was adopted from previous research to reconstruct the neural network. Briefly, Transfer Entropy quantifies the amount of information transferred between neurons and is suitable for the effective connectivity analysis of neural networks. This allowed to elucidate the microconnectome and the comprehensive and quantitative characteristics of interaction networks among neurons. Second, our study distinguishes between excitatory synapses and inhibitory synapses using a newly developed method called sorted local transfer entropy (fig. 1a). Third, we also applied methods from graph theory to evaluate the network architecture. Especially, we observed that the precedence in centrality and controlling ability of inhibitory neurons. The centrality was quantified with K-core centrality, and the controlling ability was quantified with the ratio of nodes included in FVSs (Feedback Vertex Sets). Fourth, we stained acute brain slices and gave layer labels to individual neurons. Further detail will be shown in [1].

**Fig. 1:**
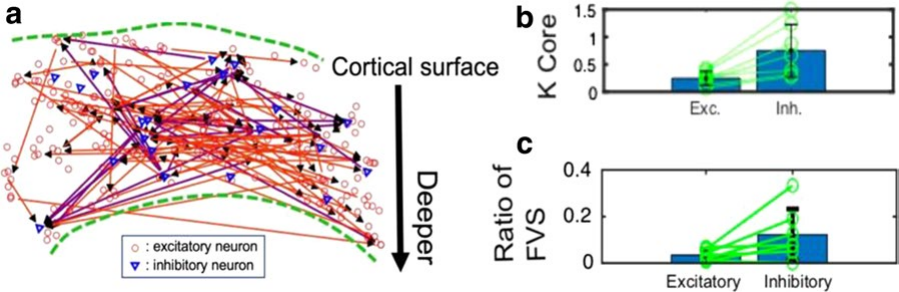
**a** Shows an example of interaction networks among neurons. **b** shows the difference of averaged K core values for all excitatory and inhibitory neurons. **c** shows the difference of ratio of number of FVS within excitatory or inhibitory neuron pools. The E/I difference was consistently and significantly observed among 7 cortical slices (Wilcoxon paired test; p<0.05)

As a result, we found that inhibitory neurons, locating highly central and having strong controlling ability of other neurons (fig. 1b-c), mainly locate in deep cortical layers by comparing with distribution of neurons coloured by NeuN immunostaining data. Preceding the observation, we also found that inhibitory neurons show higher firing rate than excitatory neurons, and that their firing rate also closely obey a log-normal distribution as previously known about excitatory neurons. Additionally, their connectivity strengths also obeyed a log-normal distribution.

**Acknowledgements:** This study was supported by several MEXT fundings (19H05215, 17K19456) and Leading Initiative for Excellent Young Researchers (LEADER) program, and grants from the Uehara Memorial Foundation.

**Reference**

1. Kajiwara M, Nomura R, Goetze F, Akutsu T, Shimono M. Inhibitory neurons are a Central Controlling regulator in the effective cortical microconnectome. bioRxiv. 2020.

## P187 Modulation of the hierarchical gradient of cognitive information processing dynamics during rest and task

### Oliver Cliff^1^, Mike Li^1^, Dennis Hernaus^2^, Lianne Scholtens^3^, Eli Müller^4^, Brandon Munn^4^, Gabriel Wainstein^4^, Ben Fulcher^5^, Joseph Lizier^1^, James Shine^4^

#### ^1^The University of Sydney, Centre for Complex Systems, Sydney, Australia; ^2^University of Maryland, Maryland Psychiatric Research Center, School of Medicine, Baltimore, Maryland, United States of America; ^3^Vrije Universiteit Amsterdam, Amsterdam, Netherlands; ^4^The University of Sydney, Brain and Mind Centre, Sydney, Australia; ^5^The University of Sydney, School of Physics, Sydney, Australia

##### **Correspondence:** Joseph Lizier (joseph.lizier@sydney.edu.au)

*BMC Neuroscience* 2020, **21(Suppl 1)**:P187

Cognition involves the dynamic adaptation of information processing resources as a function of task demands. To date, the neural mechanisms responsible for mediating this process remain poorly understood. In this study, we integrated cognitive neuroscience with information theory, network topology and neuropharmacology to advance our understanding of the fundamental computational processes that give rise to cognitive function in the human brain.

In our first experiment, we consider the contrast between dynamic whole-brain blood oxygen level dependent (BOLD) data from both the resting state and a cognitively-challenging N-back task from the Human Connectome Project (N = 457) [1,2]. We translated the raw BOLD activity levels into time series that represent the dynamics of neural information processing by measuring information flows (pairwise between regions, using transfer entropy) and information storage (self-prediction in individual regions, using active information storage) as a function of time throughout the experiment [3].

Our results show that cognitive task performance alters the whole-brain information-processing landscape in a low-dimensional manner: during rest, information flowed from granular to agranular cortices, whereas this pattern was reversed during the performance of the N-back task. These contrasting gradients of information flow reflect the difference between a stronger “bottom-up” mode during rest (with inputs from sensory cortices sent up for interpretation as the dominant flow) versus a stronger “top-down” mode during task (where task performance is facilitated by higher level control and the increase of associated flows).

To test a hypothesized mechanism for this switch [4], we modulated central noradrenaline levels in a double-blind, cross-over atomoxetine pharmacological fMRI study (N = 19) [5]. We found that potentiating the noradrenergic system altered the information processing dynamics by augmenting information transfer to and from the frontoparietal cortices. Together, our results provide a conceptual bridge between cognitive function, network topology, information theory and the ascending neuromodulatory arousal system.

**References**

1. Barch DM, et al. Function in the human connectome: task-fMRI and individual differences in behavior. Neuroimage. 2013; 80: 169-89.
2. Glasser MF, et al. The minimal preprocessing pipelines for the Human Connectome Project. Neuroimage. 2013; 80: 105-24.
3. Lizier JT. JIDT: An information-theoretic toolkit for studying the dynamics of complex systems. Frontiers in Robotics and AI. 2014; 1: 11.
4. Shine JM, Aburn MJ, Breakspear M, Poldrack RA. The modulation of neural gain facilitates a transition between functional segregation and integration in the brain. Elife. 2018; 7: e31130.
5. Hernaus D, Santa MM, Offermann JS, Van Amelsvoort T. Noradrenaline transporter blockade increases fronto-parietal functional connectivity relevant for working memory. European Neuropsychopharmacology. 2017; 27(4): 399-410.

## P188 Neuro-PC: causal functional connectivity for neural dynamics

### Rahul Biswas^1^, Eli Shlizerman^2^

#### ^1^University of Washington, Statistics, Seattle, Washington, United States of America; ^2^University of Washington, Electrical and Computer Engineering, Seattle, Washington, United States of America

##### **Correspondence:** Rahul Biswas (rbiswas1@uw.edu)

*BMC Neuroscience* 2020, **21(Suppl 1)**:P188

Neuronal interactions lead to behavior and cognition, so that investigating the interactions in the brain network is essential to understanding brain function. However, approaches that characterize the causal relationships between neuronal time series – causal functional connectivity – have not been well investigated. In this work, we develop methodology for inferring the causal functional connectivity between neurons. The methodology at the core relies on adapting the PC algorithm, a state-of-the-art method for statistical causal inference, to the neuronal time series scenario. We validate the performance of the method in synthetic signals generated from continuous time artificial neural networks. We further obtain the causal functional connectivity between neurons in mice brain under different visual stimuli from electro-physiological neural recordings.

**Fig. 1:**
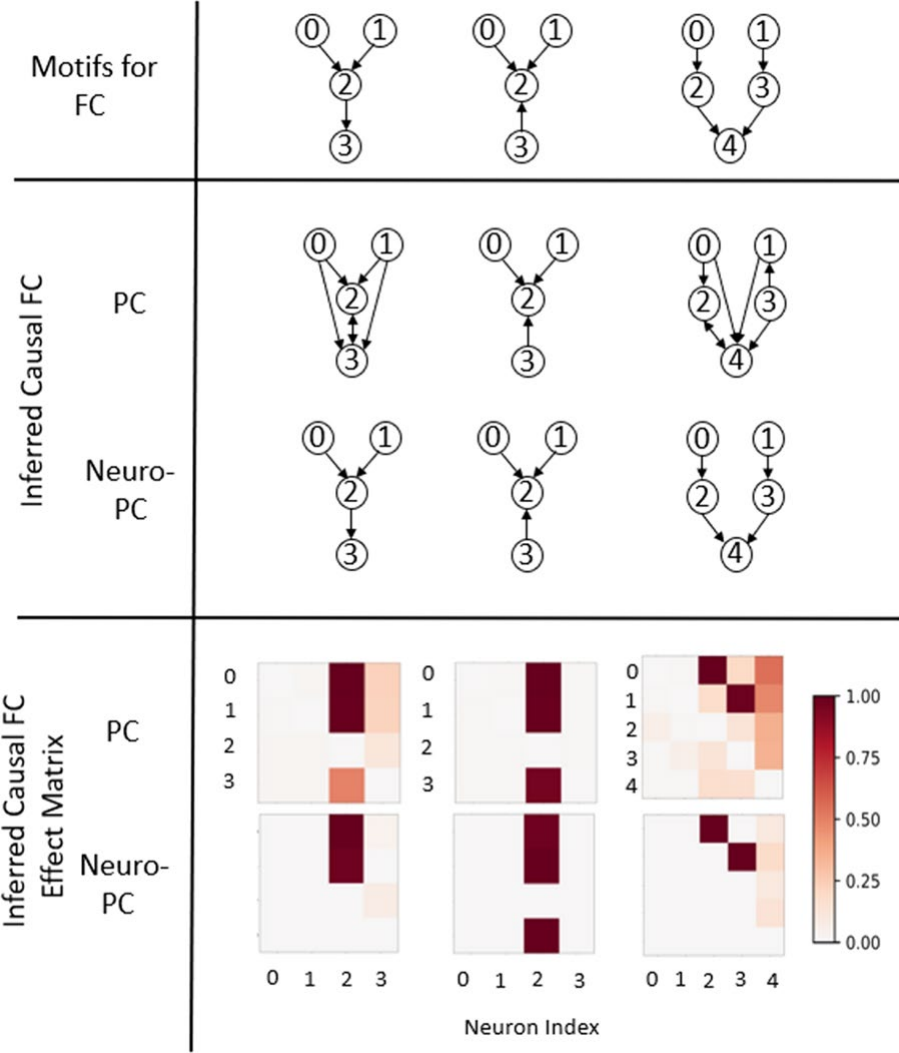
This figure compares the accuracy of the neuro-PC and traditional PC algorithm in inferring the causal functional connectivity from synthetic neural signals. The signals were generated by a continuous-time recurrent neural network with connection weights of 0.5 and connections shown by the motifs in the first row of this figure

## P189 Computationally going where experiments cannot: a dynamical assessment of dendritic currents in the behaving animal

### Alexandre Guet McCreight^1^, Frances Skinner^2^

#### ^1^Centre for Addiction and Mental Health, Krembil Centre for Neuroinformatics, Toronto, Canada; ^2^Krembil Research Institute, Division of Clinical and Computational Neuroscience, Toronto, Canada

##### **Correspondence:** Alexandre Guet McCreight (agmccrei@gmail.com)

*BMC Neuroscience* 2020, **21(Suppl 1)**:P189

Though the electrophysiology techniques that we use to probe neuronal function have made large advancements, neuronal function remains shrouded in mystery. Little is known about the current contributions that govern cell excitability across different neuronal subtypes and their dendritic compartments *in vivo*. The picture that we do have is largely based on somatic recordings performed *in vitro*.

Uncovering dendritic current contributions in neuron subtypes that represent a minority of the neuronal population is not currently a feasible task using purely experimental means. Thus, we employ morphologically-detailed multi-compartment models, and specifically, we use two models of a specific type of inhibitory interneuron, the oriens lacunosum moleculare (OLM) cell. The OLM cell is a well-studied cell type in CA1 hippocampus that is important in gating sensory and contextual information.

We use these models to assess the current contribution profile across the different somatic and dendritic compartments of the models in the presence of levels of synaptic bombardment that would occur *in vivo* and compare them to corresponding *in vitro* scenarios with somatic current injections that generate the same spike rates. Using this approach, we identify changes in dendritic excitability, current contributions, and current co-activation patterns.

We find that during *in vivo*-like scenarios the relative timing between different channel current activation patterns and voltage are preserved. On the other hand, when compared across morphological compartments, current and voltage signals were more decorrelated during *in vivo*-like scenarios, suggesting decreased signal propagation. We also observe that changes do occur during *in vivo*-like scenarios on the level of relative current contribution profiles. More specifically, in addition to shifts in the relative balances of currents that are most active during spikes, we report robust enhancements in dendritic hyperpolarization-activated cyclic nucleotide-gated channel (HCN, or h-current) activation during *in vivo*-like contexts [1]. This suggests that dendritically-located h-channels are functionally important in altering signal propagation in the behaving animal.

**Reference**

1. Guet-McCreight A, Skinner FK. Computationally going where experiments cannot: a dynamical assessment of dendritic ion channel currents during in vivo-like states. F1000Research. 2020; 9(180): 180.

## P190 Neural Field Theory: modelling development of topography via activity based mechanisms.

### Nicholas Gale^1^, Michael Small^2^

#### ^1^University of Cambridge, Applied Mathematics and Theoretical Physics, Cambridge, United Kingdom; ^2^University of Western Australia, Mathematics, Perth, Australia

##### **Correspondence:** Nicholas Gale (nmg41@cam.ac.uk)

*BMC Neuroscience* 2020, **21(Suppl 1)**:P190

Topographic maps are brain structures which connect two regions [1]. These maps are essential features of primary sensory signal processing. A prototypical animal model of such a system is the mouse retinotopic map [2]. Topography is developed using three distinct mechanisms: chemotaxis, competition, and activity based refinement [3]. Chemotaxis establishes a coarse topography with broad dendritic arbors which is followed by three stages of spontaneously generated waves of electrical activity in the retina: first at E16-P0, then from P0-P11, and finally from P11-P14 [4]. These three periods of have distinct spatio-temporal characteristics and likely perform different functions in the development of the retinotopic system. They are concurrent with electrical activity in the SC and the correlations between these signals guide Hebbian plasticity to make the refinement. Unified models of activity and genetics have found success in predicting the effects of chemical perturbations, but not activity-based perturbations [5]. The activity mechanism in these models condenses the activity into a purely spatial and radially symmetric isotropic form.

A good model of electrical activity in brain regions with lateral connectivity and dense homogenous cell types such as those in the SC is neural field theory (NFT) [6]. A theoretical framework of Hebbian-based plasticity that can incorporate time-signatures of activity has been developed for NFT [7]. This framework allows for the incorporation of a more accurate and complete description of spatio-temporally varying waves. In this paper we shall demonstrate that NFT can support the refinement and establishment of precise topography via waves of propagating activity and biologically reasonable Hebbian learning rules and therefore establish it as a useful model to study the development of topographic systems.

We develop an analytical solution to the field equation by first linearizing the sigmoid activation function. We then proceed with computational analysis of three key parameters: the width of the wave stimulus, wave-speed, and the width of the lateral connections. Finally, we discuss the limitations of the model, implications of these results in the context of the β2 knock-out (an activity perturbation), and future directions.

**References**

1. Udin SB, Fawcett JW. Formation of topographic maps. Annual Review of Neuroscience. 1988; 11(1): 289-327.
2. Ackman JB, Burbridge TJ, Crair MC. Retinal waves coordinate patterned activity throughout the developing visual system. Nature. 2012; 490(7419): 219-25.
3. Cang J, Feldheim DA. Developmental mechanisms of topographic map formation and alignment. Annual Review of Neuroscience. 2013; 36: 51-77.
4. Bansal A, et al. Mice lacking specific nicotinic acetylcholine receptor subunits exhibit dramatically altered spontaneous activity patterns and reveal a limited role for retinal waves in forming ON and OFF circuits in the inner retina. Journal of Neuroscience. 2000; 20(20): 7672-81.
5. Hjorth JJ, Sterratt DC, Cutts CS, Willshaw DJ, Eglen SJ. Quantitative assessment of computational models for retinotopic map formation. Developmental Neurobiology. 2015; 75(6): 641-66.
6. Amari SI. Dynamics of pattern formation in lateral-inhibition type neural fields. Biological Cybernetics. 1977; 27(2): 77-87.
7. Robinson PA. Neural field theory of synaptic plasticity. Journal of Theoretical Biology. 2011; 285(1): 156-63.

## P191 Electro-physiology models of cells with spherical geometry

### Jiamu Jiang^1^, Mark van Rossum^2^, Paul Smith^3^

#### ^1^University of Nottingham, Mathematics, Nottingham, United Kingdom; ^2^University of Nottingham, School of Psychology and School of Mathematical Sciences, Nottingham, United Kingdom; ^3^University of Nottingham, School of Life Sciences, Nottingham, United Kingdom

##### **Correspondence:** Jiamu Jiang (jiamu.jiang@nottingham.ac.uk)

*BMC Neuroscience* 2020, **21(Suppl 1)**:P191

Neurons and other cells use electrical signals for intra- and intercellular communication. Accurate mathematical models have been developed to describe the spatial-temporal dynamics of their voltage in response to input current. Best known is the cable equation, which describes voltage propagation along the length of a passive cable in response to current injection [1]. However, this model is not appropriate for cells with geometries other than the cylinder. Here we study the flow of electrical currents in cells with a spherical geometry in which only a thin shell close to the surface conducts. Such geometries arise for instance in white adipocytes (fat cells), which are cells consisting of an insulating lipid droplet core surrounded by a thin conductive cytoplasm shell, and might also be relevant for some types of spherically shaped peripheral neurons.

First, we construct a circuit model based on the nature of the passive membrane, and derive the equivalent of the cable equation for spherical geometries. We derive the steady-state solution analytically and show that the shape of the voltage profile depends on a single parameter that describes its electrotonic compactness. Furthermore, we show that, in contrast to the cable equation, the voltage profile across the cell is sensitive to the electrode geometry.

Next, we numerically explore the time-dependent solution to step input currents. In particular, we find that the charging and discharging are much faster than one would expect from the membrane time constant, which is important when one aims to extract fundamental membrane properties from experimental recordings.

Finally, we consider voltage-clamping experiments, often used to measure input current of cells and examine the distortions arising from imperfect space-clamp.

In conclusion, our study yields an equivalent of the cable equation for spherical geometries, which can facilitate further investigations of the electrical signals on cells with spherical structures.

**Reference**

1. Koch C. Biophysics of computation: information processing in single neurons. Oxford university press; 2004.

## P192 Estimating transfer entropy in continuous time for spike trains

### David Shorten^1^, Joseph Lizier^1^, Richard Spinney^2^

#### ^1^University of Sydney, Complex Systems Research Group, Sydney, Australia; ^2^University of New South Wales, Single Molecule Science, Sydney, Australia

##### **Correspondence:** David Shorten (david.shorten@sydney.edu.au)

*BMC Neuroscience* 2020, **21(Suppl 1)**:P192

Transfer entropy (TE) [1] is a measure of the flow of information between components in a system. It is defined as the mutual information between the past of a source and the present state of a target, conditioned on the past of the target. It has received widespread application in neuroscience [2], both for characterising information flows as well as inferring effective connectivity from data sources such as MEG, EEG, fMRI, calcium imaging and electrode arrays. Previous applications of TE to spike trains have relied on time discretisation, where the spike train is divided into time bins and the TE is estimated from the numbers of spikes occurring in each bin. There are, however, several disadvantages to estimating TE from time-discretised data [3]. Firstly, as time discretisation is a lossy transformation of the data, any estimator based on time discretisation is not consistent (it will not converge to the true value of the TE in the limit of infinite data). Secondly, whilst the loss of resolution of the discretisation will decrease with decreasing bin size, this requires larger dimensionality of the history embeddings to capture correlations over similar time intervals. This results in an exponential increase in the state space size being sampled and therefore the data requirements.

Recently, a continuous-time framework [3] for transfer entropy was developed. This framework has a distinct advantage in that it demonstrates that, for spike trains, the TE can be calculated solely from contributions occurring at spikes. This presentation reports on a newly developed continuous-time estimator for transfer entropy for spike trains which utilises this framework. Importantly, this new estimator is a consistent estimator of the TE. As it does not require time discretisation, it calculates the TE based on the raw interspike interval timings of the source and target neurons. Similar to the popular KSG estimator [4] for mutual information and TE, it performs estimation using the statistics of K-nearest-neighbour searches in the target and source history spaces. Tests on synthetic datasets of coupled and uncoupled point processes have confirmed that the estimator is consistent and has low bias. Similar tests of the time-discretised estimator have found it to not be consistent and have larger bias. The efficacy of the estimator is further demonstrated on the task of inferring the connectivity of biophyiscal models of the pyloric network of the crustacean stomatogastric ganglion. Granger causality (which is equivalent to TE under the assumption of Gaussian variables) has been shown to be incapable of inferring this particular network [5], although it was demonstrated that it could be inferred by a generalised linear model.

**References**

1. Schreiber T. Measuring information transfer. Physical Review Letters. 2000; 85(2): 461.
2. Wibral M, Vicente R, Lizier JT, editors. Directed information measures in neuroscience. Berlin: Springer; 2014.
3. Spinney RE, Prokopenko M, Lizier JT. Transfer entropy in continuous time, with applications to jump and neural spiking processes. Physical Review E. 2017; 95(3): 032319.
4. Kraskov A, Stögbauer H, Grassberger P. Estimating mutual information. Physical review E. 2004; 69(6): 066138.
5. Kispersky T, Gutierrez GJ, Marder E. Functional connectivity in a rhythmic inhibitory circuit using Granger causality. Neural Systems & Circuits. 2011; 1(1): 9.

## P193 Input strength dependence of the beta component of gamma-band auditory steady-state responses in patients with schizophrenia

### Christoph Metzner^1^, Volker Steuber^2^

#### ^1^Technische Universität Berlin, Department of Software Engineering and Theoretical Computer Science, Berlin, Germany; ^2^University of Hertfordshire, Biocomputation Research Group, Hatfield, United Kingdom

##### **Correspondence:** Christoph Metzner (cmetzner@ni.tu-berlin.de)

*BMC Neuroscience* 2020, **21(Suppl 1)**:P193

The mechanisms underlying circuit dysfunctions in schizophrenia (SCZ) remain poorly understood. Auditory steady-state response (ASSRs), especially in the gamma and beta band, have been suggested as a potential biomarker for SCZ. While the reduction of 40Hz power for 40Hz drive has been well established and replicated in SCZ patients, studies are inconclusive when it comes to an increase in 20Hz power during 40Hz drive [1]. There might be several factors explaining the inconsistencies, including differences in the sensitivity of the recording modality (EEG vs MEG), differences in stimuli (click-trains vs amplitude-modulated tones) and also large differences in the amplitude of the stimuli.

Here, we used a computational model of ASSR deficits in SCZ [2-4], in which increased IPSC decay times at GABAergic synapses produce ASSR deficits as seen experimentally. We investigated the effect of input strength on gamma and beta band power during gamma ASSR stimulation. We found that the pronounced increase in beta power during gamma stimulation seen experimentally could only be reproduced in the model for a specific range of input strengths. More specifically, if the input was too weak the network failed to produce a strong oscillatory rhythm. When the input was in the specific range, the rhythmic drive at 40Hz produced a strong 40Hz rhythm in the control network, however, in the ‘SCZ-like’ network, the prolonged inhibition led to a so-called ‘beat-skipping’, where the network would only strongly respond to every other input. This mechanism was responsible for the emergence of the pronounced 20Hz beta peak in the power spectrum. However, if the input exceeded a certain strength value, the 20Hz peak in the power spectrum disappeared again. In this case, prolonged inhibition due to the increased IPSC times was insufficient to suppress the now stronger gamma drive from the input, resulting in an absence of the beat-skipping and single peak at 40Hz in the power spectrum.

Here, we employed an established model of gamma and beta band ASSR deficits in SCZ to explore the dependence of a beta component in response to gamma drive on the strength of the input. Our finding that the beta component only existed for a specific range of input strengths might explain the seemingly inconsistent reporting in experimental studies and suggests that future ASSR studies should explicitly explore different amplitudes of their stimuli.

**References**

1. Thune H, Recasens M, Uhlhaas PJ. The 40-Hz auditory steady-state response in patients with schizophrenia: a meta-analysis. JAMA Psychiatry 2016; 73(11).
2. Vierling-Claassen D, Siekmeier P, Stufflebeam S, Kopell N. Modeling GABA alterations in schizophrenia: a link between impaired inhibition and altered gamma and beta range auditory entrainment. Journal of Neurophysiology. 2008; 99(5).
3. Metzner C. [Re] Modeling GABA alterations in schizophrenia: a link between impaired inhibition and gamma and beta auditory entrainment. ReScience 3(1).
4. Metzner C, Zurowski B, Steuber V. The role of parvalbumin-positive interneurons in auditory steady-state response deficits in schizophrenia. Sci Rep. 2019; 9(1).

## P194 Pattern separation based on rate coding in a biologically detailed cerebellar network model

### Ohki Katakura^1^, Reinoud Maex^2^, Shabnam Kadir^1^, Volker Steuber^2^

#### ^1^University of Hertfordshire, Centre for Computer Science and Informatics Research, Hatfield, United Kingdom; ^2^University of Hertfordshire, Biocomputation Research Group, Hatfield, United Kingdom

##### **Correspondence:** Ohki Katakura (mistypail.1058@gmail.com)

*BMC Neuroscience* 2020, **21(Suppl 1)**:P194

The cerebellum is involved in motor learning, temporal information processing and cognition. Inspired by the well-characterised anatomy of the cerebellum, several network models and theories of cerebellar function have been developed, such as the Marr-Albus-Ito theory of cerebellar learning. However, although morphologically realistic cerebellar neuronal models with realistic ion channel dynamics exist in isolation, a complete cerebellar cortical model comprising such biologically detailed neurons is still missing. Sudhakar et al. have implemented a cerebellar granular layer (GL) model composed of biologically detailed granule and Golgi cells (GrCs and GoCs) [1]. Here, we modified this model and integrated it with a multi-compartmental PC model, which included detailed Hodgkin-Huxley type representations of ion channels [2]. The original GL model had a length of 1.5 mm along the transversal axis. As parallel fibres (PFs), the axons of GrCs, extend for 2.0 mm along this axis, we rescaled the GL network model to 4.0 mm in transversal direction and placed the dendritic tree of the PC model at the centre of the network. Additionally, to reduce the computational requirements, we employed a sparser density of 1.92 million GrCs per mm3 in our GL model. Each spine of the PC model was connected to the nearest PF within the sagittal-vertical plane, which resulted in 143,725 PF inputs to the PC model. Inhibitory input from molecular layer interneurons (MLIs) to the PC was modelled implicitly by providing inhibitory Poisson input from 1,695 spike generators. Most of our simulations were run with 5 Hz MF background excitation and 8 Hz background MLI inhibition, which resulted in PC baseline spike rates between 50 and 60 Hz.

In a first set of simulations, our network was tested in a simple pattern separation task: a patch of excitatory mossy fibre (MF) input to the GL was stimulated; the network learnt the input pattern based on long-term depression (LTD) at PF-PC synapses; and the PC behaviour in response to learnt and novel patterns was compared. The stimulated MF patch had a radius of 100 um. The stimulation resulted in the activation of a cylindrical region of the GL above the patch. Activated GoCs spread out of the patch along the transversal axis. The initial GrC excitation lasted for about 5 ms, after which feedback inhibition from GoCs reduced the GrC spike rate to about 50% of the peak value. The resulting burst of GrC activity activated the PC model with a delay up to 5 ms. In the presence of a sufficient amount of MLI inhibition, the PC firing rate initially increased sharply in response to stimulation of the MF patch. After the MF input had been learnt based on LTD at the PF-PC synapses, the PC spike rate increases in response to learnt MF input disappeared, while equivalent novel MF stimuli still resulted in spike rate increases. These simulation results predict that a biophysically detailed PC model embedded in a realistic cerebellar network model can, under certain circumstances, employ a rate code to distinguish between learnt and novel MF input patterns.

**References**

1. Sudhakar SK, et al. Spatiotemporal network coding of physiological mossy fiber inputs by the cerebellar granular layer. PLoS Computational Biology. 2017; 13(9): e1005754.
2. De Schutter ER, Bower JM. An active membrane model of the cerebellar Purkinje cell. I. Simulation of current clamps in slice. Journal of Neurophysiology. 1994; 71(1): 375-400.

## P195 Associative memory performance in peripherally-lesioned networks repaired by homeostatic structural plasticity

### Ankur Sinha^1^, Christoph Metzner^2^, Rod Adams^3^, Neil Davey^1^, Michael Schmuker^1^, Volker Steuber^1^

#### ^1^University of Hertfordshire, Biocomputation Research Group, Hatfield, United Kingdom; ^2^Technische Universität Berlin, Department of Software Engineering and Theoretical Computer Science, Berlin, Germany; ^3^University of Hertfordshire, Biocomputation Group, Hatfield, United Kingdom

##### **Correspondence:** Ankur Sinha (a.sinha2@herts.ac.uk)

*BMC Neuroscience* 2020, **21(Suppl 1)**:P195

In spite of a plethora of peripheral lesion experiments documenting that structural plasticity causes large scale changes in brain networks [1-3], our understanding of the mechanisms of structural plasticity remains limited. Structural plasticity acts over extended periods of time, albeit at a slow rate, to modify network connectivity by the formation and removal of synapses. Alterations in network connectivity are expected to affect network function, but the resulting functional consequences of structural plasticity have not been studied in detail.

To study the activity dependent growth characteristics of neurites, which underlie network reconfiguration, we previously developed a novel model of peripheral lesioning and subsequent repair in a balanced cortical Asynchronous Irregular (AI) spiking network [4]. The network used in our model, which represents a physiological brain network, was selected since it has been demonstrated to function as an attractor-less associative memory store [5]. Using this new model, we investigated the functional effects of repair mediated by homeostatic structural plasticity on the network. We stored associative memories in the network and recalled them at different stages of the simulation by stimulating a random subset of their neurons: before deafferentation, after deafferentation but before repair, and after deafferentation during repair. At each recall, recall performance was quantified using a Signal to Noise ratio (SNR) metric [6].

Associative memories that include neurons deafferented by the peripheral lesion experience a reduction in their recall performance proportionate to the number of deprived neurons. Our results indicate that while structural plasticity restores activity of deafferented neurons to pre-injury levels, it does not restore the performance of the stored associative memories. This suggests that associative memories stored before a peripheral lesion are not necessarily protected in the repair process. Further research is needed to explore whether the repair process can be modulated to retain the performance of the stored associative memories.

**References**

1. Rasmusson DD. Reorganization of raccoon somatosensory cortex following removal of the fifth digit. Journal of Comparative Neurology. 1982; 205(4): 313-26.
2. Keck T, et al. Massive restructuring of neuronal circuits during functional reorganization of adult visual cortex. Nature Neuroscience. 2008; 11(10): 1162-7.
3. Keck T, et al. Loss of sensory input causes rapid structural changes of inhibitory neurons in adult mouse visual cortex. Neuron. 2011; 71(5): 869-82.
4. Sinha A, et al. Growth Rules for the Repair of Asynchronous Irregular Neuronal Networks after Peripheral Lesions. BioRxiv. 2019; 810846.
5. Vogels TP, Sprekeler H, Zenke F, Clopath C, Gerstner W. Inhibitory plasticity balances excitation and inhibition in sensory pathways and memory networks. Science. 2011; 334(6062): 1569-73.
6. Dayan P, Willshaw DJ. Optimising synaptic learning rules in linear associative memories. Biological Cybernetics. 1991; 65(4): 253-65.

## P196 Robustness of ultrasonic modulation of the subthalamic nucleus to GABAergic perturbation

### Thomas Tarnaud^1^, Wout Joseph^1^, Ruben Schoeters^1^, Luc Martens^1^, Timothy Van Renterghem^2^, Emmeric Tanghe^1^

#### ^1^University of Ghent - IMEC, INTEC WAVES, Ghent, Belgium; ^2^University of Ghent, INTEC WAVES, Ghent, Belgium

##### **Correspondence:** Thomas Tarnaud (thomas.tarnaud@ugent.be)

*BMC Neuroscience* 2020, **21(Suppl 1)**:P196

**Introduction:** Deep brain stimulation (DBS) is a surgical treatment for movement and neuropsychiatric disorders. Here, the subthalamic nucleus (STN) is the most common target for the treatment of advanced Parkinson’s disease (PD). Although DBS has proven effective, the procedure is associated with surgical risks such as infection and haemorrhage. Consequentially, we investigated the possibility of using ultrasound (US) as a non-invasive and reversible alternative of conventional DBS. Here, we expand on our study on the spiking behaviour of a computational STN model [1], insonicated with continuous-wave and pulsed US of different intensities. In particular, the sensitivity of the simulated STN response to hyperpolarizing input (e.g., GABAergic globus pallidus afferents) is investigated.

**Methods:** A computational model for insonication of the STN is created by combining the Otsuka-model of a plateau-potential generating STN neuron [2] with the bilayer sonophore model [3,4]. After careful validation of our model implementation by comparison with theoretical and experimental literature, simulations are performed of the STN-neuron insonicated with different ultrasonic intensities and pulse waveforms. The robustness of the simulated response to GABAergic input is tested by injecting brief hyperpolarizing currents.

**Results:** Our model results predict intensity dependent spiking modes of the STN neurons. For continuous waveforms, three different observed spiking modes in order of increasing ultrasonic intensity are low-frequency spiking, high-frequency (>120 Hz) spiking with significant spike-frequency and spike-amplitude adaptation, and a silenced mode. Simulation results indicate that only the silenced mode is robust to brief hyperpolarizing input. In contrast, the STN response will saturate robustly to the pulse repetition frequency in pulsed US, for sufficiently large intensity and pulse repetition frequency.

**Conclusion:** Model results of the ultrasonically stimulated plateau-potential generating STN predict intensity dependent spiking modes that could be useful for the treatment of PD. High-frequency spiking of the STN might “jam” pathological network activity or result in the creation of an information lesion due to short-term synaptic depression, which are potential mechanisms ascribed to conventional DBS. In contrast, the silenced mode in which the STN transmembrane potential is fixed to a stable plateau might be functionally equivalent to subthalamotomy and to depolarization blockage of STN efferents during DBS. The former and latter STN mode is induced robustly by pulsed and continuous wave US, respectively.

**References**

1. Tarnaud T, Joseph W, Martens L, Tanghe E. Computational modeling of ultrasonic subthalamic nucleus stimulation. IEEE Transactions on Biomedical Engineering. 2018; 66(4): 1155-64.
2. Otsuka T, Abe T, Tsukagawa T, Song WJ. Conductance-based model of the voltage-dependent generation of a plateau potential in subthalamic neurons. Journal of Neurophysiology. 2004; 92(1): 255-64.
3. Plaksin M, Shoham S, Kimmel E. Intramembrane cavitation as a predictive bio-piezoelectric mechanism for ultrasonic brain stimulation. Physical Review X. 2014; 4(1): 011004.
4. Lemaire T, Neufeld E, Kuster N, Micera S. Understanding ultrasound neuromodulation using a computationally efficient and interpretable model of intramembrane cavitation. Journal of Neural Engineering. 2019; 16(4): 046007.

## P197 Computational modelling of the Locus Coeruleus

### Ruben Schoeters^1^, Thomas Tarnaud^1^, Wout Joseph^1^, Luc Martens^1^, Robrecht Raedt^2^, Emmeric Tanghe^1^

#### ^1^University of Ghent - IMEC, INTEC WAVES, Ghent, Belgium; ^2^University of Ghent, Department of head and skin - 4Brain lab, Ghent, Belgium

##### **Correspondence:** Ruben Schoeters (ruben.schoeters@ugent.be)

*BMC Neuroscience* 2020, **21(Suppl 1)**:P197

The locus coeruleus (LC) is one of the most dominant noradrenergic systems in the brain that supplies the central nervous system with norepinephrine through widespread efferent projections. Consequently, it plays an important role in attention, feeding behaviour and sleep-to-wake transition [1]. Moreover, studies have shown that the locus coeruleus is correlated to the anticonvulsive action of vagus nerve stimulation (VNS) [2]. To date, the underlying mechanisms of VNS and the LC are, however, not fully understood. Therefore, we derived a computational model, such that in silico investigations can be performed. Based on the work of Carter et al. [3], we created a single compartment model that matched our in vivo measurements. These were extracted from rat brains at the 4Brain lab. The original model created by Carter et al. was a conductance-based model of the locus coeruleus and hypocretin neurons, used for the investigation of the sleep-to-wake transition. When the hypocretin neurons are omitted, our measured tonic firing rate of 3.35 ± 0.49Hz could not be reached with the original two compartment model by means of continuous current injection. The maximal achievable tonic firing rate was 0.75 Hz for a current of 0.4 A/m^2^, while a bursting behaviour followed by depolarization block was observed for higher inputs. When combined into a single compartment model, the required frequency is reached with a 0.39 A/m^2^ current injection. There were no notable differences in state occupancies that could explain the difference in firing rate. Therefore, we concluded that the lower firing rate observed in the two compartment model is solely due to spatial filtering. Finally, we compared the pinch response. The pinch was modelled as a rectangular current pulse. With an amplitude of 0.0314 A/m^2^ and pulse duration of 0.9 s, an equivalent firing rate (13.64 ± 2.75Hz vs.13.86Hz) and refractory period (1.186 ± 0.234s vs.1.09s, the measurements and model, respectively) are observed.

**References**

1. Purves D, et al. Neuroscience, volume 3. 2004.
2. Raedt R, et al. Increased hippocampal noradrenaline is a biomarker for efficacy of vagus nerve stimulation in a limbic seizure model. Journal of Neurochemistry. 2011; 117(3): 461-9.
3. Carter ME, et al. Mechanism for Hypocretin-mediated sleep-to-wake transitions. Proceedings of the National Academy of Sciences. 2012; 109(39): E2635-44.

## P198 Voltage-dependent synaptic plasticity in magnetic tunnel junctions

### Saeideh R Akbarabadi^1^, Mojtaba M Asl^2^, Peter Tass^3^

#### ^1^University of Guilan, Department of Physics, Rasht, Iran; ^2^Institute for Advanced Studies in Basic Sciences, Department of Physics, Zanjan, Iran; ^3^Stanford University, Neurosurgery, Stanford, California, United States of America

##### **Correspondence:** Saeideh R Akbarabadi (saeidehramezani7@gmail.com)

*BMC Neuroscience* 2020, **21(Suppl 1)**:P198

Spike-timing-dependent plasticity (STDP) is a fundamental learning mechanism that shapes plastic synaptic strengths in brain networks according to pre- and post-synaptic spike times [1]. Later, a model of voltage-based STDP was proposed based on the postsynaptic membrane potential to explain experimentally observed connectivity patterns in cortex [2]. Synaptic plasticity plays a key role in memory retention by modulating functional cortical circuitry in memory networks. The development of solid-state devices in recent years provided a means for computational implementation and experimental realization of neuromorphic structures designed to emulate adaptive behavior of synapses in brain. Particularly, spin-polarized transport through magnetic tunnel junctions (MTJs) is a well-characterized mechanism for the implementation of learning process due to the rapid and high-density information storage capabilities of MTJs as a memory device [3].

Previously, it has been shown that the emergent synaptic structure between a pair of neurons characterized by two reciprocally coupled synapses with STDP (see Fig. 1A) can be theoretically predicted by the effective synaptic strength in the two-neuron motif, i.e. the ratio of relative synaptic strengths to their sum [4]. In this study, we considered a two-terminal single-molecule MTJ that consists of two ferromagnetic (FM) cobalt electrodes separated by a phenyl dithiol (PDT) molecule (see Fig. 1B, top) and investigated transport properties using a non-equilibrium Green’s function (NEGF) formalism. By introducing an effective spin-polarized tunneling conductance, i.e. the ratio of relative conductances in parallel (P) and anti-parallel (AP) configurations to their sum, we show that the change in the two-component conductance crucially depends on the bias voltage applied to the MTJ where its behavior is reminiscent of the classical STDP (Fig 1A-B, bottom).

**Fig. 1:**
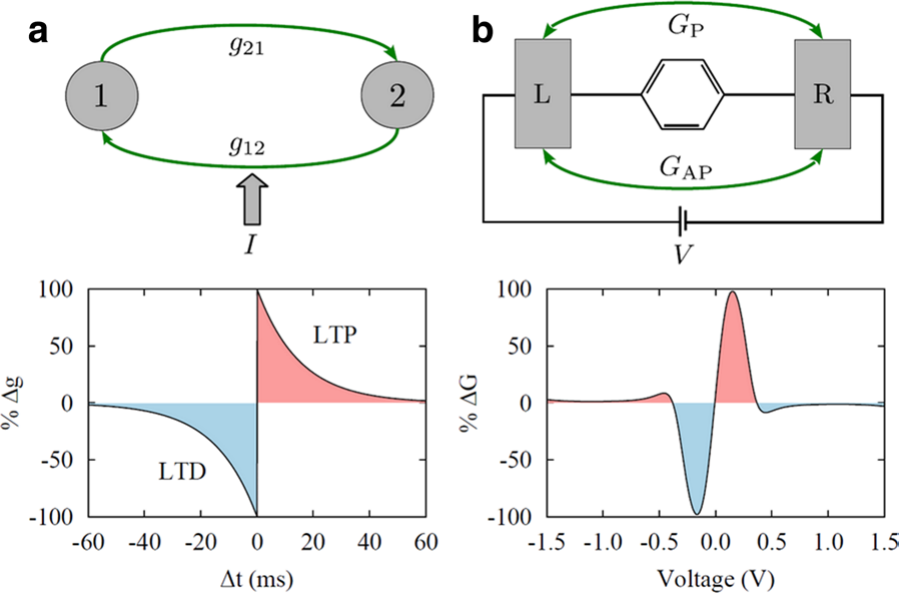
Voltage-dependent synaptic plasticity in the magnetic tunnel junction. **A** Two-neuron motif (top) and the change in the synaptic strengths induced by spike timings (bottom). **B** Two-terminal single-molecule magnetic tunnel junction (top) and the voltage-dependent change in the effective tunneling conductance (bottom)

While the optimized window for tunneling conductance is a property of the central molecule, the asymmetric change in the effective tunneling conductance is determined by the FM electrodes, and hence, can be controlled by chemical engineering of the junction. The spin-polarized current is constant near zero bias voltage, however, when a voltage is applied to the junction, the molecular energy levels are positioned within the bias window and the spin-polarized current is increased (decreased) for positive (negative) bias voltage. This provides a suitable framework to study voltage-dependent long-term potentiation (LTP) and depression (LTD) in MTJs. Ultimately, our results may contribute to the further development of neuromorphic memory devices engineered based on the adaptive properties of synapses in brain networks.

**References**

1. Bi GQ, Poo MM. Synaptic modifications in cultured hippocampal neurons: dependence on spike timing, synaptic strength, and postsynaptic cell type. Journal of Neuroscience. 1998; 18(24): 10464-72.
2. Clopath C, Büsing L, Vasilaki E, Gerstner W. Connectivity reflects coding: a model of voltage-based STDP with homeostasis. Nature Neuroscience. 2010; 13(3): 344-352.
3. Ramezani Akbarabadi S, Golsanamlou Z, Rahimpour Soleimani H. Study of Length-Dependent Tunneling Magnetoresistance in Two Phenyl Based Molecules. Current Physical Chemistry. 2014; 4(3): 285-9.
4. Asl MM, Valizadeh A, Tass PA. Delay-induced multistability and loop formation in neuronal networks with spike-timing-dependent plasticity. Scientific Reports. 2018; 8(1): 1-5.

## P199 Stimulation-induced decoupling by spike-timing-dependent plasticity in neuronal networks with propagation delays

### Mojtaba Madadi Asl^1^, Alireza Valizade^1^, Peter A Tass^2^

#### ^1^Institute for Advanced Studies in Basic Sciences, Department of Physics, Zanjan, Iran; ^2^Stanford University, Department of Neurosurgery, Stanford, California, United States of America

##### **Correspondence:** Mojtaba Madadi Asl (mojtabamadadi7@gmail.com)

*BMC Neuroscience* 2020, **21(Suppl 1)**:P199

Several neurological disorders such as Parkinson’s disease and epilepsy are characterized by pathological neuronal synchronization in cortical and sub-cortical areas. Therapeutic brain stimulation techniques aimed at shifting the pathological dynamics of the diseased brain towards healthy attractor states, are employed to restore physiological patterns of synaptic connectivity by decoupling strongly connected neurons [1]. Dynamics of cortical neuronal populations crucially depends on the synaptic connectivity of the cortex which continually change by spike-timing-dependent plasticity (STDP) [2]. It has recently shown that the effect of the STDP on the structure and the dynamics of the neuronal networks is profoundly dependent on the delay in transmission of the signals between the neurons [3]. In particular, propagation delays lead to multi-stability of the network structure such that strong bidirectional loops, loosely connected pairs of neurons, or asymmetric unidirectional connections can emerge between different pairs of neurons under the influence of STDP [4].

In the present study, by theoretical analysis of a reciprocally coupled two-neuron motif we show that the decoupling of the neurons and neuronal populations by stimulation depends on the imbalance of STDP potentiation/depression rates and time constants and the transmission delays. Then the theoretical predictions were numerically tested in a two-layer model of oscillatory networks composed of excitatory and inhibitory neurons where the individual neurons fire irregularly. The patterned stimulation is delivered simultaneously to all neurons (excitatory and inhibitory) in both layers which are connected to each other by plastic excitatory synapses characterized by interlayer propagation delays and are modified according to the STDP rule. Figure 1A shows that the stimulation pattern can shape the interlayer connections by modulating the slowly evolving synaptic dynamics and accordingly desynchronized the neuronal activity. In this way, the synaptic strengths between the layers can change from a strongly coupled regime (Fig. 1B, grey) to a more physiologically favored weakly connected state (Fig. 1B, colored distribution) due to stimulation. Furthermore, two-neuron loops which were prevalent before the stimulation onset, are entirely eliminated after the stimulation due to the stimulation-induced decoupling effect (Fig. 1C, red curve). Our results may contribute to the further optimization of therapeutic brain stimulation protocols and thus can provide new insights to the treatment of patients with hyper-synchronized neurological brain disorders.

**Fig. 1:**
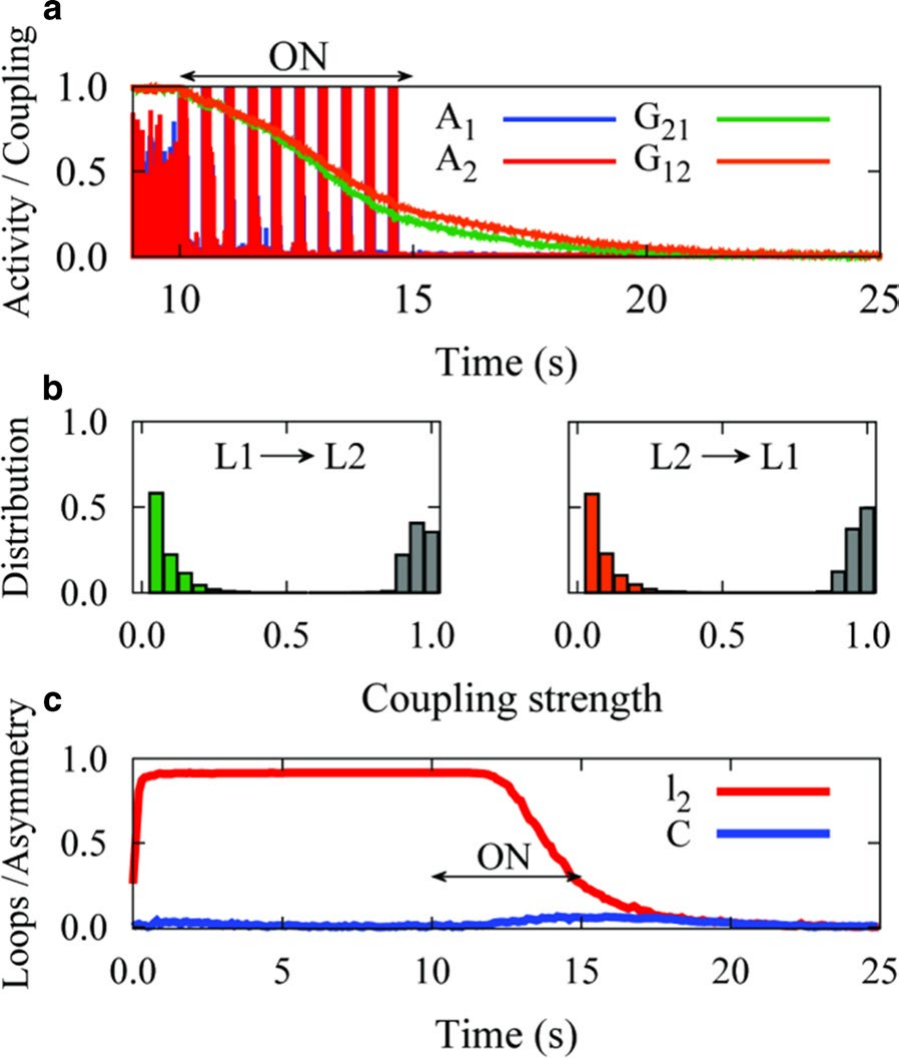
Stimulation-induced decoupling of the synaptic strengths in the two-layer network. **A** Population activity (A1/A2) and interlayer mean coupling (G21/G12). **B** Distribution of the interlayer synaptic strengths before (grey) and after (colored) stimulation cycle. **C** The number of two-neuron loops (l2) and the interlayer connection asymmetry index (C)

**References**

1. Tass PA, Majtanik M. Long-term anti-kindling effects of desynchronizing brain stimulation: a theoretical study. Biological Cybernetics. 2006; 94(1): 58-66.
2. Song S, Abbott LF. Cortical development and remapping through spike timing-dependent plasticity. Neuron. 2001 Oct; 32(2): 339-50.
3. Madadi Asl M, Valizadeh A, Tass PA. Dendritic and axonal propagation delays determine emergent structures of neuronal networks with plastic synapses. Scientific Reports. 2017; 7: 39682.
4. Madadi Asl M, Valizadeh A, Tass PA. Delay-induced multistability and loop formation in neuronal networks with spike-timing-dependent plasticity. Scientific Reports. 2018; 8(1): 1-5.

## P200 Computational modeling of the input/output mapping in the cerebellar cortex

### Akshay Markanday^1^, Sungho Hong^2^, Junya Inoue^1^, Peter Dicke^1^, Erik De Schutter^2^, Peter Thier^1^

#### ^1^Hertie Institute for Clinical Brain Research, Department of Cognitive Neurology, Tübingen, Germany; ^2^Okinawa Institute of Science and Technology, Computational Neuroscience Unit, Onna-son, Japan

##### **Correspondence:** Sungho Hong (shhong@oist.jp)

*BMC Neuroscience* 2020, **21(Suppl 1)**:P200

The cerebellar cortex is a brain region deeply involved in sensorimotor coordination and adaptation. It receives external inputs via axons called the mossy fibers (MF), delivering diverse information, including sensory and motor signals, from other brain regions. Then, the output neurons, Purkinje cells (PC), transmit the result of computation by the network. Many studies have elucidated how different stages of computation in this neural circuit represents sensory and motor information. However, circuit-level information processing has not been well-understood.

Here we investigated this question by characterizing how MF firings transform into PC outputs in recording data from those cells (n=110 and 135, respectively) in rhesus monkeys that were performing a sensorimotor task (M. Mulatta; n=2). We trained the animals for a saccadic eye movement task, where they followed a target jumping back and forth between two horizontal target locations. The fast pace and repetitive nature of the task led to a gradual decline in saccade velocities (fatigue).

We found that the firing rates of MFs linearly encoded eye speed and saccade duration, consistent with previous studies (e.g. [1]). Using the linear rate coding property of MFs and also PCs, we constructed the rate coding models of individual cells from the data and formed the virtual populations of those models for each cell type. This method enabled us to analyze eye speed-dependent variability of the population responses beyond the firing rate across trials.

By using the virtual population of MFs and PCs, we found that the activities of MFs and PCs can be both characterized by low dimensional “manifolds” [2] that resemble the limit cycles. Here, the PC manifold is higher-dimensional as compared to that of MFs and has more complex representations of variability in eye movements. Nonetheless, there exists a linear transformation between the two populations [3], which can accurately predict the average and also velocity-dependent variability in the firing rate of individual neurons.

Based on these results, we suggest that the MFs deliver a compressed, low dimensional copy of sensorimotor information from other brain areas, possibly via convergence [3], and the cerebellar cortical circuit decompresses/transforms it to higher dimensional outputs, carrying the reorganized representation of the behavioral variability.

References

1. Ohtsuka K, Noda H. Burst discharges of mossy fibers in the oculomotor vermis of macaque monkeys during saccadic eye movements. Neuroscience Research. 1992; 15: 102–114.
2. Gallego JA, et al. Neural manifolds for the control of movement. Neuron. 2017; 94: 978–984.
3. Tanaka H, Ishikawa T, Kakei S. Neural evidence of the cerebellum as a state predictor. Cerebellum. 2019; 18: 349–371.

## P201 Frequency-separated principal components analysis of cortical population activity

### Jean-Philippe Thivierge

#### University of Ottawa, School of Psychology, Ottawa, Canada

##### **Correspondence:** Jean-Philippe Thivierge (jean-philippe.thivierge@uottawa.ca)

*BMC Neuroscience* 2020, **21(Suppl 1)**:P201

Neocortical activity is characterized by the presence of low-dimensional fluctuations in firing rate that are coordinated across neurons [1]. Despite a wealth of experiments and models, the role of low-dimensional fluctuations remains unclear, in part due to limited data analysis techniques. While several approaches exist to perform dimensionality reduction [2], there is a lack of methods designed to extract frequency-specific, low-dimensional fluctuations from neural signals. This is true even with methods aimed at finding rotational structure in PCA [3], as these approaches suffer from a lack of frequency‐specific separation of components.

Here, we describe a technique termed frequency-separated principal components analysis (FS-PCA) that addresses this issue. This talk is organized as a tutorial where we first show toy examples that apply FS-PCA to artificial signals. Then, we provide an application of FS-PCA to both spontaneous and evoked cortical activity. Finally, we discuss the interpretation, limitations, and possible extensions of this technique to problems in systems neuroscience.

FS-PCA is based on recent theoretical advances on the eigenspectrum of Hankel matrices [4]. As a first example, we consider a sine wave with added zero-mean Gaussian noise (Fig.1a). We show that this signal can be converted to a Hankel matrix (Fig. 1b) whose eigenspectrum contains 2*f* + 1 largest eigenvalues, where *f* is the number of characteristic frequencies of the original signal. The reconstructed signal obtained from FS-PCA closely matches the amplitude, phase, and frequency of the original signal (Fig. 1c).

**Fig. 1:**
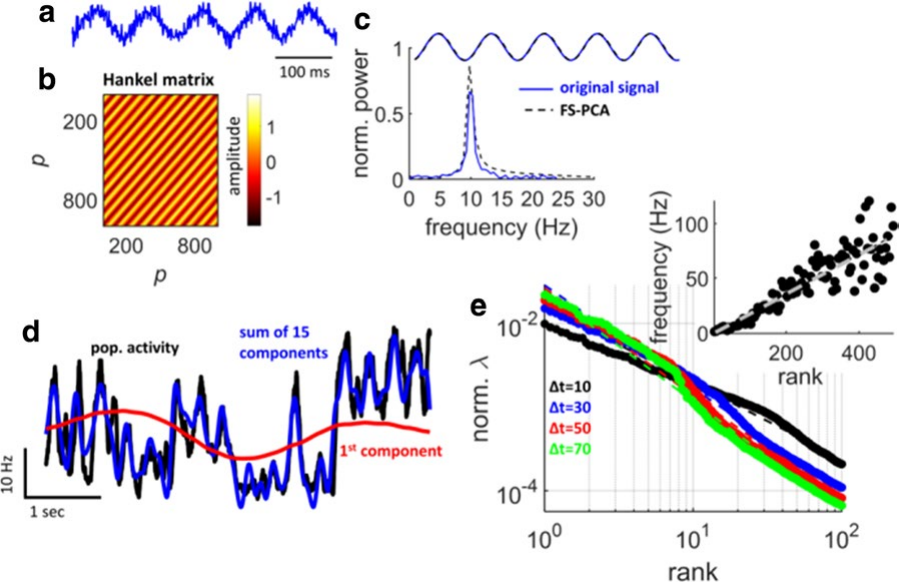
Frequency-separated principal components analysis of artificial and cortical signals. **a** Example of noisy sine wave. **b** Hankel matrix obtained from “a”. **c** Top, original and reconstructed signals. Bottom, power spectra. **d** Reconstruction of V1 mean population activity. **e** Distribution of ranked eigenvalues. Inset, relation between rank and characteristic frequency of each component

Next, we apply FS-PCA to population recordings from macaque V1 cortex. We show that the first dimension of the reconstructed signal captures the slow, low-frequency fluctuations in mean population activity observed over time (Fig. 1d, red line). Adding further dimensions markedly improves the reconstruction of population activity (Fig.1d, blue line). Overall, ranked eigenvalues obtained from FS-PCA followed an approximate power-law where the highest ranked dimensions captured a large proportion of the data (Fig.1e). In turn, highest-ranked dimensions had a lower characteristic frequency than lower dimensions (Fig.1e, inset). In sum, these results suggest that while a broad spectrum of frequencies contributed to population activity, fluctuations in spontaneous activity were dominated by low-frequency components.

**References**

1. Cohen MR, Kohn A. Measuring and interpreting neuronal correlations. Nature Neuroscience. 2011; 14: 811–819.
2. Cunningham JP, Yu BM. Dimensionality reduction for large-scale neural recordings. Nature Neuroscience. 2014; 17: 1500-1509.
3. Churchland MM, et al. Neural population dynamics during reaching. Nature. 2012; 487: 51-56.
4. Li Z, Li W, Zhao X. Feature frequency extraction based on principal component analysis and its application in axis orbit. Shock and Vibration. 2018; 2530248.

## P202 Analysis of Parkinson’s disease tremor with t-SNE

### Dmitrii Todoro

#### Centre de Recerca Matemàtica, Computational Neuroscience Group, Spain

##### **Correspondence:** Dmitrii Todoro (todorovdi@gmail.com)

*BMC Neuroscience* 2020, **21(Suppl 1)**:P202

Many symptoms of Parkinson’s disease, despite a long history of research, are still not completely understood. In particular, there is no clear understanding on how Parkinsonian rest tremor is generated and even how it can be detected and distinguished from voluntary movements in neural recordings. We use multimodal (MEG and STN LFP) brain data, recorded from tremor-dominant PD patients and employ t-Distributed Stochastic Neighbor embedding (t-SNE) on it to evaluate how distinguishable are distinct behavioral states (rest, tremor, voluntary movements). We also describe which data features (both spectral and time-domain ones) contribute most to such classification.

## P203 A neural mechanism of working-memory manipulation in a categorization-task performance

### Yoshiki Kashimori, Hikaru Tokuhara

#### The University of Electro-Communications, Department of Engineering Science, Tokyo, Japan

##### **Correspondence:** Yoshiki Kashimori (kashi@pc.uec.ac.jp)

*BMC Neuroscience* 2020, **21(Suppl 1)**:P203

Working memory has a function by which temporal information is maintained and recognized in the brain, and is ubiquitous in various brain regions. A growing body of working memory research has indicated the neural mechanism underlying the maintenance of working memory. Several studies have demonstrated that working memory is maintained by a persistent activity of neural assemblies. Other studies have proposed that it is maintained by a short-term synaptic plasticity. The mechanism of working memory maintenance is still a matter of debate. Furthermore, it is unclear how working memory is linked to behavior and decision-making.

In this study, to clarify the neural mechanisms underlying the maintenance and manipulation of working memory, we focus on the function of prefrontal cortex in a delayed match-to-categorization task studied by Freedman et al. [1]. In this task, monkeys were presented with a sample and a test stimulus, separated by a delay period, and were trained to judge whether these stimuli were from the same category. Freedman et al. showed that working memory of category information was formed in the PFC. Our previous model demonstrated the neural mechanism of the working memory shaped in the PFC [2]. In this study, we aim to understand a unified mechanism of working memory maintenance and its manipulation for behavior. We develop a network model that performs the maintenance and recognition of temporal information of a sample and a test stimulus. The model consists of the networks of IT and PFC. The PFC model is further constructed with a positive-feedback-loop layer, a recurrent network, and a decision layer. The positive-feedback-loop layer produces a persistent activity of a previously presented stimulus, allowing the layer to maintain information of a sample stimulus as working memory. The recurrent network encodes the temporal information of a sample stimulus and a test stimulus. The learning of temporal information was made by Backpropagation Through Time method. The decision layer has neurons responding to a match and a non-match trial. We also investigate the discrimination ability of our model for more complex tasks that have longer temporal sequences and many category numbers.

We demonstrate that maintenance of working memory and encoding of temporal sequence are sequentially manipulated in different areas of the PFC. We also show that the temporal sequence is encoded by activity pattern of the recurrent circuit, independently of task decision. The sparseness of activity pattern increases with increasing the number of category. The principal component analysis of activity patterns reveals that the activity patterns of non-match trials move far away from the activity patterns of match trials as the learning proceeds. Furthermore, we show that the decision of task trials is adjusted by the learning of the connections between recurrent neurons representing the activity patterns and decision neurons, according to task context.

**References**

1. Freedman DJ, Riesenhuber M, Poggio T, Miller EK. A comparison of primate prefrontal and inferior temporal cortices during visual categorization. Journal of Neuroscience. 2003; 23(12): 5235-46.
2. Abe Y, Fujita K, Kashimori Y. Visual and Category Representations Shaped by the Interaction Between Inferior Temporal and Prefrontal Cortices. Cognitive Computation. 2018; 10(5): 687-702.

## P204 Stabilization of spiking neural networks using stochastic and high-frequency neurostimulation disrupts seizure-like transitions

### Scott Rich^1^, Axel Hutt^2^, Frances Skinner^1^, Jeremie Lefebvre^3^, Taufik Valiante^1^

#### ^1^Krembil Research Institute, Division of Clinical and Computational Neuroscience, Toronto, Canada; ^2^INRIA Nancy Grand Est, MIMESIS, Strasbourg, France; ^3^University of Ottawa, Department of Biology, Ottawa, Canada

##### **Correspondence:** Scott Rich (sbrich@umich.edu)

*BMC Neuroscience* 2020, **21(Suppl 1)**:P204

Epilepsy is one of the most common serious neurological disorders in the world, typified by repeated unprovoked seizures. Such seizures are characterized by an abrupt transition to a hyper-active, and often hyper-synchronous, brain state [1]. These in vivo transitions share noted similarities to mathematical transitions occurring through bifurcations, suggesting that the study of seizure onset is fertile ground for interdisciplinary research [2]. Neuromodulation represents a promising avenue for clinical intervention in patients with epilepsy, although the field is still wanting for principled understandings of how such devices mitigate seizure onset [3]. Such understanding of neuromodulatory mechanisms may allow for better stimulation strategies to reduce the burden of seizures.

Here, we use a network model to probe how sudden transitions into oscillatory dynamics, which share clear parallels with seizure onset, are influenced by both intrinsic and extrinsic inputs. These extrinsic inputs can be viewed as a model of a neuromodulatory intervention. Building on a previous model of cortical gamma activity [4], the model consists of 500 excitatory and 500 inhibitory all-to-all connected Poisson neurons with heterogeneity implemented in their rheobase analogues.

A combination of numerical simulations and mean-field analyses revealed that high variance and/or high frequency stimulation waveforms were most efficient in preventing multi-stability in these networks, where multi-stability serves as a mathematical harbinger of the sudden transition between asynchronous and oscillatory network dynamics. Furthermore, our analysis showed that stabilization of neural activity is via a selective recruitment of inhibitory cells, providing a theoretical undergird for the known key role these cells play in both the healthy and diseased brain. Interestingly, this effect occurred without the need to precisely “target” the inhibitory population, highlighting that neuromodulatory devices utilizing these stimulation paradigms may not need to be excessively “precise” in order to elicit the desired response. While deep brain stimulation systems have long been thought to affect neural circuits via the creation of a “functional” or “informational” lesion [5], potentially through depolarization blockade [6], these findings provide theoretical support for a distinct mechanism of action through selective interneuronal activation. Taken together, these results provide new vistas on the underlying mechanisms through which neuromodulatory approaches stabilize neural microcircuit activity, utilizing a variety of computational tools including numerical simulation, mean-field reduction, and stochastic stability analysis.

**References**

1. Reynolds EH. Introduction: epilepsy in the world. Epilepsia. 2002; 43: 1-3.
2. Wending F, et al. Computational models of epileptiform activity. Journal of Neuroscience Methods. 2016; 260: 233-251.
3. Salanova V. Deep brain stimulation for epilepsy. Epilepsy & Behavior. 2018; 18(6): 514-532.
4. Herrmann C, et al. Shaping neural oscillations with periodic stimulation. Journal of Neuroscience. 2016; 36(19): 5328-5337.
5. Grill WM, et al. Deep brain stimulation creates an informational lesion of the stimulated nucleus. Neuroreport. 2004; 15(7): 1137-1140.
6. McIntyre CC, et al. Uncovering the mechanism(s) of action of deep brain stimulation: activation, inhibition, or both. Clinical Neurophysiology. 2004; 115(6): 1239-1248.

## P205 Development of efficient connectivity for reliable signal transmission through STDP

### Hedyeh Rezaei^1^, Ad Aertsen^2^, Arvind Kumar^3^, Alireza Valizade^1^

#### ^1^Institute for Advanced Studies in Basic Sciences, Physics, Zanjan, Iran; ^2^Bernstein Center Freiburg, University Freiburg, Biology, Freiburg, Germany; ^3^KTH Royal Institute of Technology, Computational Brain Science, Stockholm, Sweden

##### **Correspondence:** Hedyeh Rezaei (h.rezaei@iasbs.ac.ir)

*BMC Neuroscience* 2020, **21(Suppl 1)**:P205

A fundamental requirement for brain function is the efficient communication between different brain regions. This requirement turns out not to be trivial in the presence of various noise sources in the brain. Two possible strategies taken by the nervous system to face the abundance of noise could be to integrate signals either across time (firing rate) or across a population of neurons (synchrony) to retain the signal in spite of the uncorrelated noisy background. However, the reliable transmission of signals requires either strong and sparse or dense and weak connections for reliable transmission of rate codes or synchrony codes, respectively [1].

However, the typical connectivity between brain regions is neither strong nor dense. Recent work has highlighted the importance of feedback connections. Feedback connections can strengthen the signals through reverberations in the bi-directionally coupled modules [2]. This mechanism depends on the matching of the total effective delay along forward and backward intermodule connections and the period of the local oscillations in the modules, determined by the within and between module connections. This raises the question how the networks in the brain might tune their parameters in a range that favours such reliable and economic signal transmission. Here, we tested if the biological synaptic plasticity rules can self-organize an initially disorganized network to such a tuned regime for reliable signal transmission. Inspired by Hebb’s postulate [3], we hypothesized that in the presence of abundant synaptic connections between the modules in a developmental stage of the nervous system, only those with matching parameters for reliable transmission can potentiate. While potentiation of these synapses facilitates the reliable transmission of signals, depression of other ‘unfit’ connections reduces the structural cost and gives rise to an efficient substrate for reliable signal transmission. We found that with STDP, the intermodule connections with delays matching the oscillation period of a single network module were potentiated, whereas other connections were ultimately eliminated (Fig. 1a-b). We also found how this mechanism facilitated reliable signal transmission to downstream areas in case the network consisted of several (up to 10) such modules (Fig. 1c). Our results suggest that STDP can lead to the emergence of networks with tuned parameters for reliable and efficient signal transmission out of an initially inefficient network with an extravagant structural cost.

**Fig. 1:**
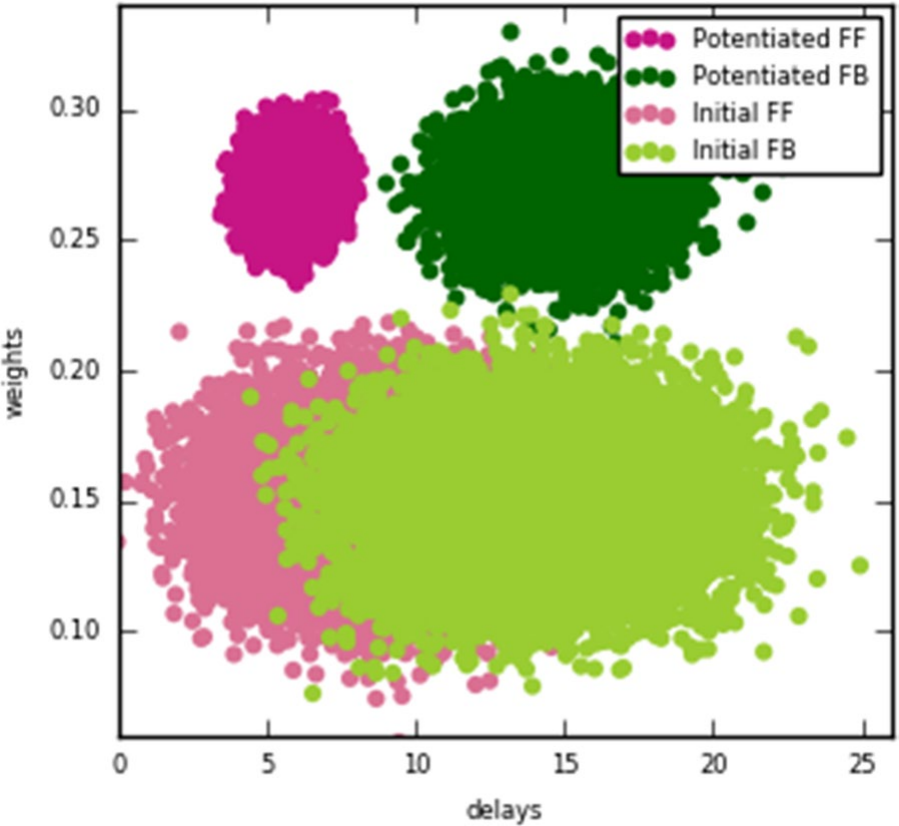
Development of efficient connectivity for reliable signal transmission. Scatter plot shows the initial and final weights and delays of forward and backward synaptic connections. Distribution of the delays in the initial and final states in two directions. Sum of peaks of final distribution of feedforward and feedback delays matched the resonance period (20-25ms) of the network modules

**References**

1. Kumar A, Rotter S, Aertsen A. Spiking activity propagation in neuronal networks: reconciling different perspectives on neural coding. Nature Reviews Neuroscience. 2010; 11(9): 615-27.
2. Rezaei H, Aertsen A, Kumar A, Valizadeh A. Facilitating the propagation of spiking activity in feedforward networks by including feedback. BioRxiv. 2019; 712547.
3. Bi GQ, Poo MM. Synaptic modification by correlated activity: Hebb’s postulate revisited. Annual Review of Neuroscience. 2001 Mar; 24(1): 139-66.

## P206 Parameter exploration in neuron and synapse models driven by stimuli from living neuron recordings

### Manuel Reyes-Sanchez^1^, Irene Elices^1^, Rodrigo Amaducci^1^, Francisco B Rodriguez^2^, Pablo Varona^2^

#### ^1^Universidad Autónoma Madrid, Grupo de Neurocomputación Biológica, Madrid, Spain; ^2^Universidad Autónoma Madrid, Ingeniería Informática, Madrid, Spain

##### **Correspondence:** Ronald Calabrese (manuel.reyes@uam.es)

*BMC Neuroscience* 2020, **21(Suppl 1)**:P206

In this work, we present an approach to automatically explore neuron and synapse model parameter to archive target dynamics or characterize emergent phenomena which rely on the temporal structure of biological recordings that are used as inputs to the models. The associated exploration and mapping allow us to assess the role of different elements in the equations of the neuron and synapse models to build a nontrivial integration of sequential information, which is also reflected in the time course of the corresponding model response.

We illustrate this methodology in the context of dynamical invariants defined as cycle-by-cycle preserved relationships between time intervals that build robust sequences in neural rhythms. We have recently unveiled the existence of such invariants in the pyloric CPG of crustacean, even under the presence of intrinsic or induced large variability in the rhythms [1]. The proposed strategy can be generalized for many types of neural recordings and models.

During such protocol, we input biological data with a characteristic temporal structure to different model neurons. The biological recordings are preprocessed online to adapt the corresponding time and amplitude scales to those of the synapse and neuron models using a set of algorithms developed in our previous works [2,3]. Our methodology can then map the neuron and synapse parameters that yield a predefined dynamic taking into account the temporal structure of the model output. The algorithms allow for a full characterization of the parameter space that contributes to the generation of the predefined dynamics.

To illustrate this protocol that combines experimental recordings and theoretical paradigms, we have applied it to the search for dynamical invariants established between a living CPG cell and a model neuron connected through a graded synapse model. Dynamical invariants are preserved cycle-by-cycle, even during transients. In our validation tests, we have mapped the presence of a linear relationship, i.e. an invariant, between the interval defined by the beginning of the bursting activity of the two neurons (first-to-first spike interval between the living and model neurons) and the instantaneous period of their sequence in such hybrid circuit.

The protocol has been used to assess the role of model and synaptic parameters in the generation of the dynamical invariant, achieving a high efficient mapping in a few minutes. We argue that this approach can also be employed to readily characterize optimal parameters in the construction of hybrid circuits built with living and artificial neurons and connections, and, generally, to validate neuron and synapse models.

**Acknowledgements:** Funded by AEI/FEDER PGC2018-095895-B-I00 and TIN2017-84452-R.

**References**

1. Elices I, Levi R, Arroyo D, Rodriguez FB, Varona P. Robust dynamical invariants in sequential neural activity. Scientific Reports. 2019; 9(1): 1-3.
2. Amaducci R, Reyes-Sanchez M, Elices I, Rodriguez FB, Varona P. RTHybrid: a standardized and open-source real-time software model library for experimental neuroscience. Frontiers in Neuroinformatics. 2019; 13:11.
3. Reyes-Sanchez M, Amaducci R, Elices I, Rodriguez FB, Varona P. Automatic adaptation of model neurons and connections to build hybrid circuits with living networks. Neuroinformatics. 2020; 1-7.

## P207 Hybrid robot driven by a closed-loop interaction with a living central pattern generator with online feedback

### Rodrigo Amaducci^1^, Irene Elices^1^, Manuel Reyes-Sanchez^1^, Alicia Garrido-Peña^1^, Rafael Levi^1^, Francisco B Rodriguez^2^, Pablo Varona^2^

#### ^1^Universidad Autónoma Madrid, Grupo de Neurocomputacion Biologica, Madrid, Spain; ^2^Universidad Autónoma Madrid, Ingeniería Informática, Madrid, Spain

##### **Correspondence:** Rodrigo Amaducci (amaducci.rodrigo@gmail.com)

*BMC Neuroscience* 2020, **21(Suppl 1)**:P207

A hybrid robot or hybrot is a technology that combines living cells and networks with robotics. This technology is largely undeveloped and has been mainly implemented with neuron cultures and multichannel electrode arrays [1,2]. Hybrots have a lot of potential to study neural networks properties involved in the control of locomotion, sensorimotor transformation and behavior.

Central pattern generators (CPG) are neural circuits that produce robust rhythmic sequences involved in motor functions such as breathing or walking. Because of their role in generating and coordinating motor rhythms, bio-inspired CPGs have been widely employed in robotic paradigms [3] including the design of novel mechanisms for autonomous locomotion [4]. However, the intrinsic mechanisms that give rise to the coordination of living CPG dynamics have not been used yet for hybrid robot implementation.

In this work, we present the first hybrot controlled by a living CPG from the crab Carcinus Maenas. The robot and the living neural circuit are connected following a closed-loop protocol that involves a dynamic-clamp setup to communicate both elements through Bluetooth signaling. We show that effective robotic locomotion is achieved when it is controlled and coordinated by the flexible rhythmic sequences produced by the circuit of living motoneurons. The robot is equipped with a light sensor that sends a sensory feedback to the CPG in the form of intracellular current injection. We report the analysis of the presence of dynamical invariants in the intervals that build up the sequential activations of the living circuit [5] and how they are transmitted to the robot resulting in a coordinated locomotion. In turn, the robotic sensory feedback is translated into a variation of the living network activity while keeping the motor sequence, which results in a coherent response to the change in the environmental light.

**Acknowledgements**: We acknowledge support from AEI/FEDER PGC2018-095895-B-I00 and TIN2017-84452-R.

**References**

1. Potter SM. Hybrots: hybrid systems of cultured neurons+ robots, for studying dynamic computation and learning. In Simulation of Adaptive Behavior 7: Workshop on Motor Control in Humans and Robots-On the interplay of real brains and artificial devices, Edinburgh, Scotland 2002.
2. Li Y, Sun R, Wang Y, Li H, Zheng X. A novel robot system integrating biological and mechanical intelligence based on dissociated neural network-controlled closed-loop environment. PloS one. 2016; 11(11): e0165600.
3. Ijspeert AJ. Central pattern generators for locomotion control in animals and robots: a review. Neural Networks. 2008; 21(4): 642-53.
4. Herrero-Carrón F, Rodríguez FB, Varona P. Bio-inspired design strategies for central pattern generator control in modular robotics. Bioinspiration & Biomimetics. 2011; 6(1): 016006.
5. Buzsáki G, Watson BO. Brain rhythms and neural syntax: implications for efficient coding of cognitive content and neuropsychiatric disease. Dialogues in Clinical Neuroscience. 2012; 14(4): 345.

## P208 NEST 3.0, NESTML and NEST Desktop: new user experience and classroom readiness

### Markus Diesmann^1^, Jochen M Eppler^1^, Susanne Kunkel^2^, Charl Linssen^3^, Håkon Mørk^2^, Abigail Morrison^1^, Hans Ekkehard Plesser^2^, Sebastian Spreizer^4^, Stine Brekke Vennemo^2^

#### ^1^Forschungszentrum Jülich GmbH, Jülich, Germany; ^2^Norwegian University of Life Sciences, Faculty of Science and Technology, Ås, Norway; ^3^Forschungszentrum Jülich, Jülich, Germany; ^4^Forschungszentrum Jülich GmbH; University of Freiburg, Freiburg, Germany

##### **Correspondence:** Charl Linssen (c.linssen@fz-juelich.de)

*BMC Neuroscience* 2020, **21(Suppl 1)**:P208

Over the last year, major advances have taken place in NEST Simulator and its associated tooling. This poster describes updates in NEST 3.0, NESTML and NEST Desktop.

NEST 3.0 is the next major version update of NEST. With it, changes are made not only to the user interface but also to the inner workings of NEST. In the PyNEST interface, new concepts are introduced for the compact and efficient description of large populations of neurons and synapses as well as distributions of parameter values. The PyNEST Topology module is integrated into the standard PyNEST package, so that creation and connection of spatial networks can now be performed by calling the standard functions. NEST 3.0 improves the expressiveness of model descriptions and the speed of network creation. A new and improved infrastructure for handling recordings has been implemented, with built-in backends to record to memory, ASCII files and screen.

NESTML is a domain-specific language for neurons and synapses. It serves as a specification and exchange format, where dynamical systems are expressed in continuous time (e.g., using differential equations) and have the additional ability to receive and emit precisely timed events (representing action potentials). Feature highlights include a concise yet expressive syntax inspired by Python, direct entry of dynamical equations, and imperative programming-style specification of event handling and generation.

NESTML comes with a powerful toolchain, written in Python, and is released under the GNU GPL v2.0. It parses a given model and performs code generation (“transpiling”). The generated code targets a particular hardware and software platform (e.g. NEST running on a high-performance computing cluster) with highly optimised and performant code. The toolchain performs detailed analytical and numerical analysis to yield optimal solver recommendations, and precise solutions where possible. Target platforms can be added flexibly using Jinja2 templates. As a result, NEST users can now specify neuron and synapse models in the same way they specify the network structure, using a domain-specific language that is independent of the underlying C++ code.

NEST Desktop is a web-based graphical user interface which enables the rapid construction, parametrization, and instrumentation of neuronal network models typically used in computational neuroscience. The client-server architecture supports installation-free access to NEST. The primary objective is to provide an accessible classroom tool that allows users to rapidly explore neuroscience concepts without the need to learn a simulator control language at the same time. NEST Desktop opens NEST technology for a new user group, namely students in the classroom, and contributes to equal opportunities in education.

These advances, combined with work on the user-level documentation and deployment mechanisms, contribute to the creation and maturation of the NEST ecosystem as a component of a software infrastructure for neuroscience.

**Acknowledgements:** This research has received partial funding from the Helmholtz Association through IVF no. SO-092 (Advanced Computing Architectures, ACA) and the European Union’s Horizon 2020 research and innovation programme under grant agreement No 720270 (HBP SGA1) and No 785907 (HBP SGA2), and the EU Horizon 2020 programme “DEEP-EST” (contract no. ICT-754304). Use of the JURECA supercomputer through VSR grant JINB33.

## P209 A predictive model of serotonergic fiber densities based on reflected Fractional Brownian Motion

### Skirmantas Janusonis^1^, Ralf Metzler^2^, Thomas Vojta^3^

#### ^1^University of California, Department of Psychological and Brain Sciences, Santa Barbara, California, United States of America; ^2^University of Potsdam, Institute of Physics and Astronomy, Potsdam-Golm, Germany; ^3^Missouri University of Science and Technology, Department of Physics, Rolla, Missouri, United States of America

##### **Correspondence:** Skirmantas Janusonis (janusonis@ucsb.edu)

*BMC Neuroscience* 2020, **21(Suppl 1)**:P209

All vertebrate brains contain a dense matrix of thin axons (fibers) that release serotonin (5-hydroxytryptamine), a neurotransmitter that modulates a wide range of neural, glial, and vascular processes. Altered serotonergic fiber densities have been associated with a number of mental disorders and conditions, such as Autism Spectrum Disorder, Major Depressive Disorder, and exposure to 3,4-methylenedioxymethamphetamine (MDMA, “Ecstasy”). Also, serotonergic fibers can regrow in adulthood and therefore can support the functional recovery of the brain after injury. However, the processes that lead to the self-organization and plasticity of this fiber system remain poorly understood.

Our previous research has shown that the trajectories of serotonergic fibers in terminal fields can be modeled as random walks [1,2]. We now introduce a computational model that is based on Fractional Brownian Motion (FBM), a continuous stochastic process that generalizes normal Brownian Motion and allows correlations between non-overlapping increments. The model capitalizes on the recently discovered properties of the reflected FBM (rFBM) in one-dimensional domains [3,4].

FBM is parametrized by the Hurst index (*H*) that allows subdiffusion (*H* < ½) and superdiffusion (*H* > ½). We show that in the superdiffusive regime rFBM-walks recapitulate some key features of regional serotonergic fiber densities, on the whole-brain scale. Specifically, by using supercomputing simulations of fibers as FBM-paths in two-dimensional brain-like domains, we demonstrate that the resultant steady-state distributions approximate the fiber distributions in mouse brain sections immunostained for the serotonin transporter (a marker for serotonergic fibers in the adult brain). These results do not sensitively depend on the *H*-value (for *H* > ½), precise estimates of which are currently difficult to obtain experimentally.

This novel framework can support predictive descriptions and manipulations of the serotonergic matrix and it can be further extended to incorporate the detailed physical properties of the fibers and their environment. We also show that this neuroscience-motivated approach can stimulate theoretical investigations of rFBM in two- and three-dimensional domains, with potential applications in other fields of science.

**Acknowledgements:** This research is funded by the National Science Foundation (grants #1822517 and #1921515 to SJ), the National Institute of Mental Health (grant #MH117488 to SJ), the California NanoSystems Institute (Challenge grants to SJ), the Research Corporation for Science Advancement (a Cottrell SEED Award to TV), and the German Research Foundation (DFG grant #ME 1535/7-1 to RM), and the Foundation of Polish Science (an Alexander von Humboldt Polish Honorary Research Scholarship to RM).

**References**

1. Janušonis S, Detering N. A stochastic approach to serotonergic fibers in mental disorders. Biochimie. 2019; 161: 15-22.
2. Janušonis S, Mays KC, Hingorani MT. Serotonergic Axons as 3D-Walks. 2019.
3. Wada AH, Vojta T. Fractional Brownian motion with a reflecting wall. Physical Review E. 2018; 97(2): 020102.
4. Guggenberger T, Pagnini G, Vojta T, Metzler R. Fractional Brownian motion in a finite interval: correlations effect depletion or accretion zones of particles near boundaries. New Journal of Physics. 2019; 21(2): 022002.

## P210 Seizure pathways change on circadian and slower timescales in individual patients with focal epilepsy

### Yujiang Wang

#### Newcastle University, Newcastle upon Tyne, United Kingdom

##### **Correspondence:** Yujiang Wang (yujiang.wang@newcastle.ac.uk)

*BMC Neuroscience* 2020, **21(Suppl 1)**:P210

Personalised medicine requires that treatments adapt to not only the patient, but changing factors within each individual. Although epilepsy is a dynamic disorder characterised by pathological fluctuations in brain state, surprisingly little is known about whether and how seizures vary in the same patient. We quantitatively compared within-patient seizure network evolutions using intracranial electroencephalographic (iEEG) recordings of over 500 seizures from 31 patients with focal epilepsy (mean 16.5 seizures/patient). In all patients, we found variability in seizure paths through the space of possible network dynamics. Seizures with similar pathways tended to occur closer together in time, and a simple model suggested that seizure pathways change on circadian and/or slower timescales in the majority of patients. These temporal relationships occurred independent of whether the patient underwent antiepileptic medication reduction. Our results suggest that various modulatory processes, operating at different timescales, shape within-patient seizure evolutions, leading to variable seizure pathways that may require tailored treatment approaches [1].

**Reference**

1. Schroeder GM, et al. Seizure pathways change on circadian and slower timescales in individual patients with focal epilepsy. Proceedings of the National Academy of Sciences. 2020; 117(20): 11048-58.

## P211 Neural networks architectures that detect visual motion like biological brains

### Hamish Pratt^1^, Bernard Evans^2^, Thomas Rowntree^3^, Ian Reid^3^, Steven Wiederman^2^

#### ^1^The University of Adelaide, School of Computer Science, Adelaide, Australia; ^2^The University of Adelaide, Adelaide Medical School, Adelaide, Australia; ^3^The University of Adelaide, Australian Institute of Machine Learning, Adelaide, Australia

##### **Correspondence:** Hamish Pratt (hamishc.pratt@gmail.com)

*BMC Neuroscience* 2020, **21(Suppl 1)**:P211

Convolutional neural networks (CNNs) have become the-state-of-the-art for image classification and object detection tasks, as they have the ability to combine appearance features in a scene. CNNs used for detection and classification tasks primarily process single static images to combine the features. In a manner similar to biological brains, some neural networks also utilise motion as complementary information to aid object detection tasks. However, unlike the brain, these networks rarely classify ‘moving objects’ in a scene. Our research analyses a neural network’s ability to detect unique motion cues in scenes without any appearance, to understand the limits for neural networks to process motion information. We generated variant CNN models to understand different architectures that can process motion information and built a recurrent CNN with information skip layers for our experiments. By comparing our network’s detection rates against psychophysical stimuli used in human experiments, we found the neural network and humans both struggled to correctly detect unique motion in similar conditions. When trained for detecting higher orders of motion, stimuli observable by even small insects, the network responded strongly to the order of motion for which it was trained against, and was, for the majority, unresponsive to the other motion orders. To further test the ability of motion detection in neural networks, we trained a neural network against detecting repeating spatio-temporal signals inside a scene of random noise. The results from our experiments show that alongside convolutional neural networks’ success in detecting appearance features for object classification, they are able to detect motion without appearance. With the understanding of similarities to biological brains and limitations in which these neural networks perform fundamental vision tasks like motion detection, we will have a better understanding of a network’s suitability for real-world applications.

## P212 Neuron conduction delay plasticity for unsupervised learning

### Joshua Arnold, Peter Stratton, Janet Wiles

#### University of Queensland, School of ITEE, Brisbane, Australia

##### **Correspondence:** Joshua Arnold (joshua.arnold1@uqconnect.edu.au)

*BMC Neuroscience* 2020, **21(Suppl 1)**:P212

Spiking neurons inherently represent time due to their momentary discrete action potentials; as such, they are well poised to process spatiotemporal data. Despite their temporal nature, most computational learning rules focus on modulating synaptic efficacy (weight), which only indirectly influences a neuron’s temporal dynamics.

Weight-based rules are well suited to solving synchronous spatial learning tasks, as demonstrated by the surge of interest in rate-coded neurons performing frame-based image classification using backpropagation.

For temporal tasks, however, weight based learning rules often implicitly rely on the temporal dynamics of membrane equations or synaptic transfer functions to discriminate between spatially identical, but temporally distinct, inputs.

Allowing spiking neurons to perform some aspect of explicit temporal learning offers significant advantages for learning asynchronous spatiotemporal patterns compared to weight-based rules alone.

With improvements in imaging techniques, there is accumulating evidence for action-potential conduction velocity plasticity over long and short timescales [1,2].

The biological mechanisms implementing Conduction Delay Plasticity (CDP) could include myelination, changes in axon diameter, changes to nodes of Ranvier length, bouton movement, or likely some combination of these mechanisms and others not listed.

While the precise nature and interaction of the biological mechanisms underlying CDP remain elusive, computational models provide a framework in which theories can be tested.

Several CDP learning rules have been suggested with greatly varying levels of biological fidelity and computational efficiency; in particular, we focus on one rule called Synaptic Delay Variance Learning [3].

Here we demonstrate the ability of a Leaky Integrate and Fire spiking model using only CDP (no weight learning) to learn a repeating spatiotemporal pattern in a continuous time input stream with no training signal; that is, the delays self-organise to represent the temporal structure of the input.

A neuron receives 2000 afferents firing with Poisson distributions of 10Hz, while the embedded pattern is presented with a Poisson distribution of 5Hz and consists of 500 afferents firing once within a 50ms period.

The input is normalised such that the patterns cause no change in overall activity during presentations and all afferents involved in the pattern are adjusted to maintain a 10Hz firing rate.

After 250 seconds of training, the neuron is tested for 50 seconds and successfully responds to 99.7% of pattern presentations with 3.1% false positives, averaged over 100 trials.

These results provide a demonstration of CDP as a functional computational learning rule enabling spiking neurons to perform unsupervised learning of spatiotemporal data.

**References**

1. Fields RD. A new mechanism of nervous system plasticity: activity-dependent myelination. Nature Reviews Neuroscience. 2015; 16(12): 756-67.
2. Arancibia-Carcamo IL, Ford MC, Cossell L, Ishida K, Tohyama K, Attwell D. Node of Ranvier length as a potential regulator of myelinated axon conduction speed. Elife. 2017; 6: e23329.
3. Wright PW, Wiles J. Learning transmission delays in spiking neural networks: A novel approach to sequence learning based on spike delay variance. In The 2012 International Joint Conference on Neural Networks. 2012 Jun 10 (pp. 1-8). IEEE.

## P213 Local synaptic connections alter spike responses and signal propagation in models of globus pallidus pars externa

### Erick Olivares, Matthew Higgs, Charles Wilson

#### Universidad de Texas at San Antonio, Department of Biology, San Antonio, Texas, United States of America

##### **Correspondence:** Erick Olivares (erickolivaresb@gmail.com)

*BMC Neuroscience* 2020, **21(Suppl 1)**:P213

Globus pallidus pars externa (GPe) has been seen as a relay nucleus in the indirect pathway of the basal ganglia, which simply inverts the inhibitory signal arriving from striatum. In this view, the information flowing through GPe runs in parallel paths having no interaction with one another. However, GPe neurons are fast autonomous oscillators that project axon collaterals spanning a wide area, creating an active local inhibitory network. How does local connectivity affect GPe steady-state firing and responses to stimuli? To answer that question, we constructed network models of GPe using experimentally measured neuron firing rates and input-output properties and four different structures of local connectivity.

GPe neurons are intrinsic oscillators, so they can be simulated using phase models, which allow us to predict the time of the next spike considering all the synaptic inputs arriving during the inter-spike interval (ISI). We experimentally measured the firing rates, phase resetting curves (PRCs), and inter-spike membrane potential trajectories of a sample of GPe neurons (n=19) in mouse brain slices. Using the dataset of PRCs and firing rates, we generated 1000 artificial neurons with the diversity of the recorded neurons. The local connectivity in GPe is known to be GABAergic, and we can measure the amplitudes and kinetics of the unitary synaptic conductances. In addition, the average probability of connection between any two neurons in GPe can be estimated from anatomical studies. As the connectivity patterns are unknown, we simulated networks with four qualitatively different adjacency matrices (Fig. 1A): “regular”, “random”, “small world” and “hierarchical”. Each network was created using the same set of 1000 neurons and the same total number of connections (10000). At steady state, the four networks produced similar distributions of firing rates and coefficient of variation of ISI.

**Fig. 1:**
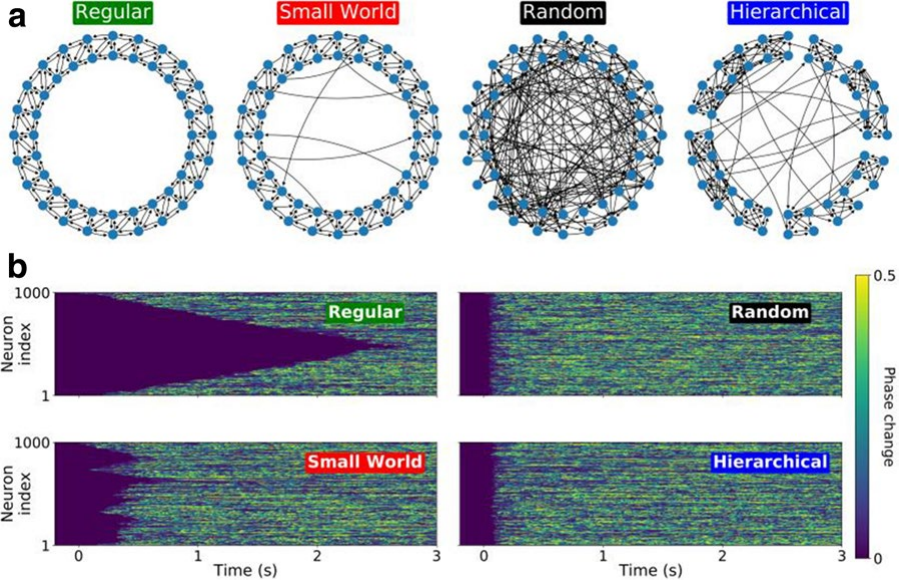
**A** Simplified representation of the four networks used in the study. Each architecture contains the same number of neurons and connections. **B** Perturbation generated by a single IPSP applied to one neuron propagates across the entire network. Figures show the absolute difference in phase between the perturbed and unperturbed simulations

In the phase model, the local inhibitory synaptic barrage alters the stationary density of phase from the uniform distribution of an unperturbed oscillator. In the networks simulated here, the neurons spent more time at late phases, i.e. near spiking. This was more evident in regular and small-world networks compared to hierarchical and random networks. The skewed phase density altered the response to an external excitatory input, as measured by the PSTH. The networks with the greatest skew produced the largest response amplitude and the briefest response time course.

Because of the GPe neurons’ autonomous firing and connectivity, a local perturbation might propagate through the entire network (Fig. 1B). We investigated this by comparing the neurons’ phases in matched simulations with and without perturbation by a single synaptic input. The disturbance propagated more often in the random network (69%) than in the regular network (61%). When the disturbance propagated, it changed the phase of most neurons in the network, but did not alter the overall firing rates, ISI distributions, or phase density. The perturbations propagated faster and with shorter latencies in hierarchical and random networks, and slowest with longest latency in the regular network.

Our results show that in sparsely coupled networks of autonomous oscillator neurons like GPe, the architecture of connections has little effect on the steady-state firing rates, yet can determine the response to external perturbation, and may allow widespread signal propagation within the network.

## P214 Bayesian model for multisensory integration and segregation

### Ma Xiangyu^1^, He Wang^1^, Min Yan^1^, Wenhao Zhang^2^, K Y Michael Wong^1^

#### ^1^Hong Kong University of Science and Technology, Institute for Physics, Hong Kong; ^2^University of Pittsburgh, Institute for Physics, Pittsburgh, Pennsylvania, United States of America

##### **Correspondence:** Ma Xiangyu (xmaal@connect.ust.hk)

*BMC Neuroscience* 2020, **21(Suppl 1)**:P214

The brain processes information from different sensory modalities in our daily routine, and the neural system should have the ability to distinguish whether different signals originate from the same source. Experimental data suggested that the brain can integrate visual and vestibular cues to infer heading-direction according to Bayesian prediction. In the dorsal medial superior temporal (MSTd) area and the ventral intraparietal (VIP) area, there exist two types of neurons, congruent and opposite neurons. By focusing on a prior distribution of stimuli that is fully correlated, a recent work by Zhang et al. [1] suggested that those two distinct types of neurons have complementary roles in multisensory integration and segregation. In the proposed distributed network architecture, cues of different modalities are processed by different modules, but the modules are reciprocally connected. Congruent neurons of given preferred stimuli in one module are connected to the congruent neurons in the other module with similar preferred stimuli. In contrast, opposite neurons of given preferred stimuli in the two modules are connected to their counterparts with opposite preferred stimuli. This facilitates the congruent neurons to yield Bayesian posterior estimates of multisensory integration in a broad range of parameters, and the opposite neurons to provide signals dependent on cue disparity, enabling the segregation of cues in subsequent processing. However, in the previous model, there are parameter ranges that the inference can only be approximately Bayesian. Hence, in this work, we will approach the dynamics analytically and propose improvements for achieving more accurate Bayesian inference.

Furthermore, the Bayes-optimality in the previous work was based on a prior distribution of stimuli that is fully correlated, whereas in practice, there are many other scenarios described by priors with more than one components. For example, studies in causal inference consider prior distributions with a correlated and an independent component. In the second part of our work, we propose a neural circuit with additional modules to tackle these cases. In addition, we further illustrate that the network encodes strong evidence for the correlations between the prior information and the network structure. Finally, we discuss how the Bayes factor reveals the potential of our network model as a decision making neural circuit for causal inference.

**Reference**

1. Zhang WH, et al. Complementary congruent and opposite neurons achieve concurrent multisensory integration and segregation. Elife. 2019; 8: e43753.

## P215 Loss aversion and outcome-value encoding: a negative association between posterior insula activity and loss aversion coefficient

### Ka Chun Wu^1^, Isaac Ip^2^, Fiona Ching^2^, Heytou Chiu^2^, Rosa Chan^3^, Savio Wong^2^

#### ^1^The Chinese University of Hong Kong, Department of Educational Psychology, Hong Kong; ^2^The Chinese University of Hong Kong, Laboratory for Brain and Education; Department of Educational Psychology, Hong Kong; ^3^City University of Hong Kong, Department of Electrical Engineering, Hong Kong

##### **Correspondence:** Ka Chun Wu (kachunwu7-s@link.cuhk.edu.hk)

*BMC Neuroscience* 2020, **21(Suppl 1)**:P215

In prospect theory, loss aversion is one important parameter that modulates one’s decision in involving risk. Previous studies find that amygdala activity is related to the degree of loss aversion during the action-selection processes. In this study, we examine the brain response associated with decision outcome and how that varies across subjects with different degrees of loss aversion. We expect that people with high loss aversion experience stronger emotional impact when receiving a negative outcome after taking risk. We hypothesize a person’s degree of loss aversion could be reflected by the BOLD contrast across decision outcomes.

To test this hypothesis, we recorded and analysed the fMRI data of twenty-one participants (10 males and 11 females; M age = 17.9 ± 0.75) during the Loss Aversion Task (LAT) [1]. The LAT was implemented with a rapid event-related design in which participants were given two options: NoGamble option with a guaranteed outcome and Gamble with 50% chance of getting a better-than-NoGamble outcome and 50% chance of getting a worse-than-NoGamble outcome (Fig. 1A). The utility of the two options varied so that one option has higher or equal utility respect to another. Participants were presented with a feedback indicating the outcome. Loss aversion coefficient (lambda; ƛ= −beta loss / beta gain) is estimated by fitting the behavioural responses to the logistic function. A higher lambda value indicates stronger loss aversion, with ƛ = 1 meaning equal weight for gain and loss.

**Fig. 1:**
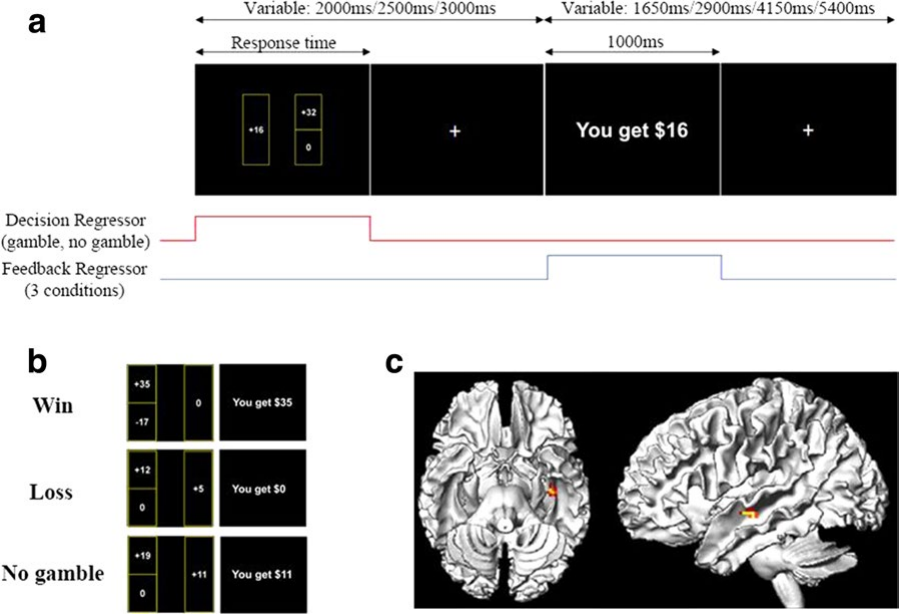
**A** Design of the Loss Aversion Task (LAT). **B** Example of the three feedback conditions: Win, Loss and No gamble. **C** Lambda is negatively correlated with the activity of left posterior insular cortex

We find feedback-related activities at medial prefrontal cortex (mPFC) but find no significant difference among the valence of feedback (gain, loss, no-gain/loss). Condition contrasts reveal that the activity of the left posterior insular cortex during gambling loss relative to guaranteed loss is negatively correlated with participants’ lambda (Fig. 1B). In the other words, gambling loss elicit stronger insula response relative to guaranteed loss in participants with lower lambda, while those with higher lambda do not differentiate between gambling and guaranteed loss. The insular cortex potentially increases the sensitivity of the anticipated loss, or alternatively, reduces the sensitivity to the gamble loss [2]. Both interpretations lead to the likelihood a person choose to take risk in a long run given both gambling loss and guaranteed loss had similar subjective value in the past. In conclusion, the individual difference in loss aversion could be capture by condition contrasts in a LAT and gives insight to the model of outcome-value encoding.

**References**

1. Tom SM, Fox CR, Trepel C, Poldrack RA. The neural basis of loss aversion in decision-making under risk. Science. 2007; 315(5811): 515-518.
2. Canessa N, et al. Neural markers of loss aversion in resting-state brain activity. NeuroImage. 2017;146: 257-265.

## P216 Multiplicity and correlations of unidirectional and reciprocal connections in the nervous system of the Caenorhabditis elegans

### Edgar Wright^1^, Alexander Goltsev^1,2^

#### ^1^University of Aveiro, Department of Physics, Aveiro, Portugal; ^2^A.F. Ioffe Physical-Technical Institute, St. Petersburg, Russia

##### **Correspondence:** Alexander Goltsev (goltsev@ua.pt)

*BMC Neuroscience* 2020, **21(Suppl 1)**:P216

Reciprocally connected pairs (RCPs) of neurons are the simplest structural motif in neuronal networks. More complex structural motifs are composed of three or more neurons. RCPs are formed by reciprocal synapses, and represent local microcircuits that can act as feedback loops. Evidence of the ubiquitous presence of RCPs in the central nervous system of different animals is well-established. Statistical analysis of connections between principal cortical cells has shown that RCPs are overrepresented in the somatosensory cortex, neocortex, and olfactory bulb. RCPs are also overrepresented in the neuronal network of the nematode *Caenorhabditis elegans* (*C. elegans*) [1].

In this work we analysed the statistics of reciprocal and undirectional chemical connections between pairs of neurons in the neuronal connectomes of the male and hermaphrodite *C. elegans*, using data recently published in [2]. First, our analysis shows that even if all unidirectional connections are removed, i.e. if approximately 63% of all connections are removed, approximately 83% of neurons with chemical synapses in the male (87%) in the hermaphrodite) remain in the strongly connected cluster, where they are reachable from each other through sequences of reciprocal connections. This result shows that reciprocal connections provide communication between most neurons with chemical synapses in the *C. elegans*. Second, average multiplicity was found to be larger among reciprocal connections than unidirectional connections, both among afferent and efferent connections. The probability that a connection has large multiplicity (over 10 synapses per connection) is larger among reciprocal connections. Third, it was found that most neurons with an above-average number of presynaptic neighbors have a number of afferent synapses which is on average larger than the average connectome multiplicity. Moreover, the larger the in-degree of a neuron the larger the multiplicity of the afferent connections to this neuron (Fig. 1). The number of efferent connections, however, was found to be largely independent of the number of postsynaptic neurons. Fourth, the number of afferent synapses and the number of presynaptic neurons are strongly correlated, such that neurons with more presynaptic neighbors receive disproportionally more synapses.

Given the known functional roles of some RCPs, it is possible that enhanced multiplicity among RCPs is the result of their function. For example, RCPs have been implicated in memory formation. Since the formation of long-term memory results in an increase in the number of dendritic spines on neurons that are part of a memory engram, it is possible that a similar mechanism plays a role in the enhanced multiplicity of reciprocal connections in the *C. elegans*. The enhanced multiplicity may in part result from Hebbian structural plasticity. As neurons with a larger number of presynaptic neighbors are more likely to be activated, they are also more likely to experience prolonged periods of high activity, which in turn can induce the formation of more synapses. Conversely, the multiplicity of neurons with less presynaptic neighbors should decrease as the result of increased periods of low neuronal activity.

**Fig. 1:**
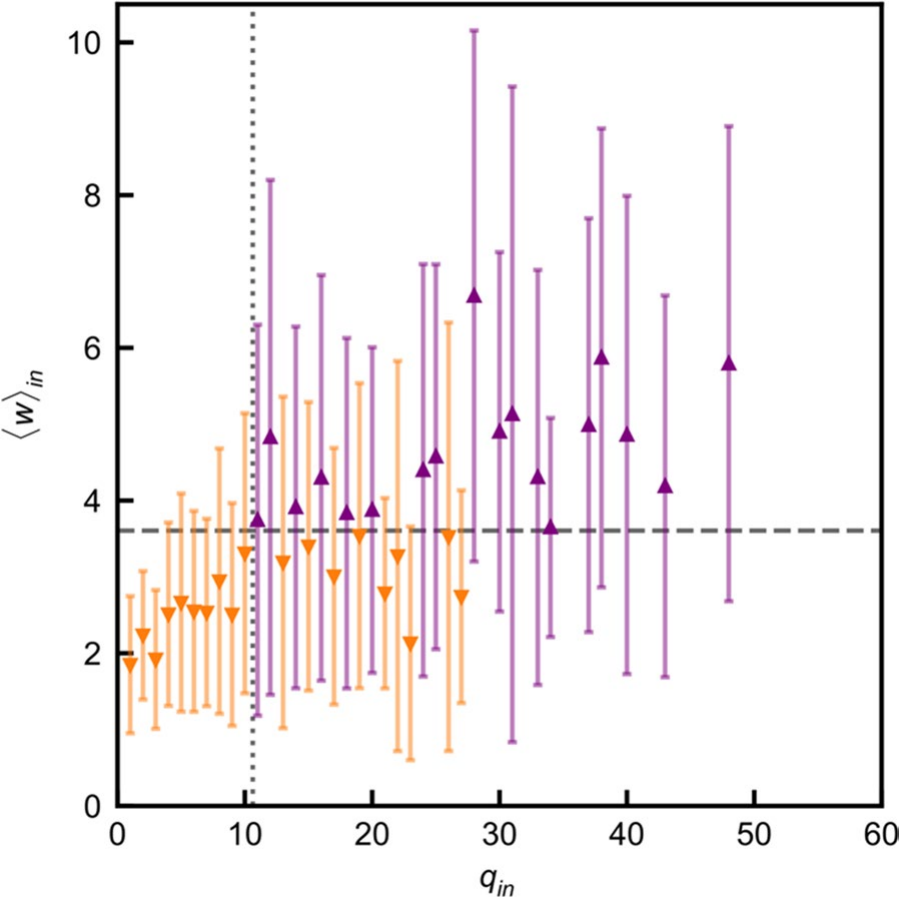
Average synaptic multiplicity *w*_in_ of afferent connections to neurons with *q*_in_ presynaptic neighbors, for the male *C. elegans*. Above-average multiplicity (>3.61) is indicated by upward (purple) triangles and below-average multiplicity is indicated by downward (orange) triangles. Vertical bars measure the standard deviation (spread about the average value). The vertical dotted line indicates the average number of presynaptic neighbors

**References**

1. Varshney LR, Chen BL, Paniagua E, Hall DH, Chklovskii DB. Structural properties of the Caenorhabditis elegans neuronal network. PLoS Computational Biology. 2011; 7(2): e1001066.
2. Cook SJ, et al. Whole-animal connectomes of both Caenorhabditis elegans sexes. Nature. 2019; 571(7763): 63-71.

## P217 Autaptic connections can implement state transitions in spiking neural networks for temporal pattern recognition

### Yaqoob Muhammad^1^, Volker Steuber^2^, Borys Wróbel^3^

#### ^1^University of Hertfordshire, Computer Science, Hatfield, United Kingdom; ^2^University of Hertfordshire, Biocomputation Research Group, Hatfield, United Kingdom; ^3^Adam Mickiewicz University in Poznan, Evolving Systems Laboratory, Poznan, Poland

##### **Correspondence:** Yaqoob Muhammad (m.yaqoob3@herts.ac.uk)

*BMC Neuroscience* 2020, **21(Suppl 1)**:P217

In biological neuronal networks, autaptic connection or autapses are synaptic connections between the axon and dendrites of a single neuron, which can be either excitatory (glutamatergic) or inhibitory (GABAergic). Since their first discovery four decades ago [1], the existence of autapses has now been documented in various brain regions including neocortex, hippocampus and cerebellum [2]. However, the functional role of autapses is still unknown [3]. In this work, we show the importance of autapses for temporal pattern recognition in simple spiking neural networks. The computational task is to recognise a specific signal sequence in a stream of inputs so that a single output neuron spikes for the correct input signal, while remaining silent for other input signals. Having understood the role of autapses and the resulting switching mechanism in networks evolved for recognising signals of length two and three [4], we were able to define rules for constructing the topology of a network handcrafted for recognising a signal sequence of length m with n interneurons. We show that autapses are crucial for switching the network between states and observe that a minimal network recognising a signal of length m requires at least (m-1) autaptic connections. In contrast to solutions obtained by the evolutionary algorithm in [4] we show that the number of interneurons required to recognise a signal is equal to the length of the signal. Finally, we demonstrate that a successful recogniser network (where n is greater than or equal to three) must have three specialised neurons: a “lock”, “switch” and “accept” neuron, in addition to the other state maintaining neurons (N0, N1, … Nn-4), whose number depends on length of the signal. All interneurons in the network require an excitatory autaptic connection, apart from the “accept” neuron.The “lock” neuron is always active (thanks to an excitatory autapse), which prevents the output from spiking except when the network receives the second to last correct input signal and allows the output neuron to spike in response to the correct last input. If the lock is released by the second to last correct input signal, the “accept” neuron (i) produces spike/s in the output neuron when the network receives the last correct input and (ii) sends a signal to the “switch” neuron, which transforms the network back into the start state. The “switch” neuron is responsible for the transition between the network start state and other possible inter-signal network states. In the future, we intend to explore other functional roles of autapses and higher-order loops in larger neuronal networks.

**References**

1. Van Der Loos H, Glaser EM. Autapses in neocortex cerebri: synapses between a pyramidal cell’s axon and its own dendrites. Brain Research. 1972; 48: 355-60.
2. Bacci A, Huguenard JR. Enhancement of spike-timing precision by autaptic transmission in neocortical inhibitory interneurons. Neuron. 2006; 49(1): 119-30.
3. Wiles L, et al. Autaptic connections shift network excitability and bursting. Scientific reports. 2017; 7: 44006.
4. Yaqoob M, Steuber V, Wróbel B. The Importance of Self-excitation in Spiking Neural Networks Evolved to Recognize Temporal Patterns. In International Conference on Artificial Neural Networks 2019 Sep 17 (pp. 758-771). Springer, Cham.

## P218 The geometry of spatio-temporal odorant mixture encoding in the Drosophila mushroom body

### Aurel A Lazar, Tingkai Liu, Chung-Heng Yeh

#### Columbia University, Electrical Engineering, New York, United States of America

##### **Correspondence:** Tingkai Liu (tl2747@columbia.edu)

*BMC Neuroscience* 2020, **21(Suppl 1)**:P218

Biological organisms are constantly challenged with navigating odorant scenes comprised of complex time-varying mixtures of volatile compounds. To characterize odorant mixture encoding and processing, two seemingly contradictory hypotheses have been considered: Elemental and Configural. The Elemental scheme [1,2] encodes mixtures linearly with identifiable components, while the Configural scheme [3] encodes mixtures as a holistic odor object distinct from its components. Here, we advance a feedback normalization model of the Drosophila early olfactory system that reconciles the two encoding schemes, and analyze the geometry of the resulting odorant encoding space.

Our model consists of Projection Neurons (PNs), Kenyon Cells (KCs) and the Anterior Paired Lateral (APL) neuron. To quantify the degree to which a mixture is encoded elementally vs configurally, we employ the Cosine Similarity (CS) between the KC code of the odorant mixture and its pure components. We show that, due to the global feedback gain control exerted by the APL neuron and the KC spiking mechanism, the steady-state KC output is an input-invariant sparse combinatorial code with consistently 5-10% active neurons. This sparse code results in a configural mixture code with low CS scores against all pure components, enabling the association of different valences to an odorant mixture from its components. Preceding the steady-state phase, the circuit makes full use of gradient encoding in the first two layers of the olfactory pathway [4] and the APL temporal dynamics to encode each mixture elementally with about 25% active neurons. This code exhibits a high (∼1) CS score with all pure components in the mixture, indicating high linear decodability. Moreover, we demonstrate that the elemental encoding phase enables cognitive functions for odorant processing. For example, combined with an attention-driven modulation signal, elemental encoding overtakes configural encoding in steady state and promotes odorant tracing for navigation.

Next, we investigate the geometry of the odorant and the KC spaces and show that smooth interpolation between odorant input vectors leads to sharp discontinuities in the KC representation space. Further analysis reveals that the steady-state KC combinatorial codes are almost binary, concentrating around the corners of a high dimensional cube in KC space.

This sparse grid-like structure gives rise to a distinctive clustering of odorant mixture identities in the KC space, with high intra-cluster similarity and inter-cluster dis-similarity. This geometric view of the KC encoding space suggests that sharp transitions in the KC representation is the result of crossing cluster boundaries, which leads to large jumps across vertices of the KC cube, and explains previous observations that small compositional changes of mixtures (mixture ratio or component identities) can incur large differences in perception. Similarly, the transition from elemental to configural phases across time corresponds to a trajectory in the KC space from a subspace spanned by KC codes of odorant components to the vertices of the cube.

**Fig. 1:**
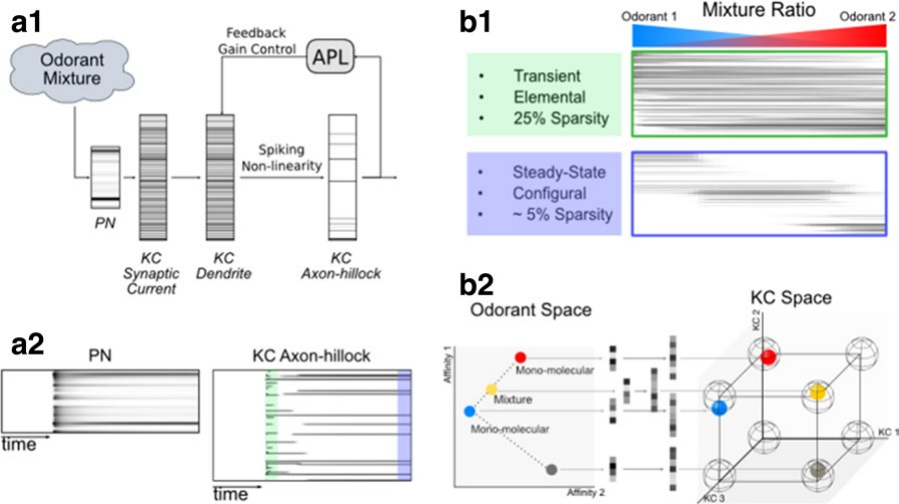
**A1** PN-KC-APL Architecture Overview. **A2** Spatio-Temporal Dynamics of Input/Output of PN-KC-APL Circuit. **B1** Transient and Steady-state KC across binary odorant mixtures shows different transition smoothness properties. **B2** KC Combinatorial code occupy corners of a hypercube. Mixing odorants causes KC response to jump between vertices

**Acknowledgments:** The research reported here was supported, in part, by NSF grant #1544383 and in part by AFOSR grant #FA9550-16-1-0410.

**References**

1. Rokni D, Murthy VN. Analysis and Synthesis in Olfaction. ACS Chemical Neuroscience. 2014 5(10):870–72.
2. Sehdev A, Szyszka P. Segregation of unknown odors from mixtures based on stimulus onset asynchrony in honey bees. Frontiers in Behavioral Neuroscience. 2019; 13: 155.
3. Thomas-Danguin T, et al. The perception of odor objects in everyday life: a review on the processing of odor mixtures. Frontiers in Psychology. 2014; 5: 504.
4. Kim AJ, Lazar AA, Slutskiy YB. Projection neurons in Drosophila antennal lobes signal the acceleration of odor concentrations. Elife. 2015; 4: e06651.

## P219 Interpretable modeling of neurons in cortical area V4 via compressed convolutional neural networks

### Reza Abbasi-Asl^1^, Bin Yu^2^

#### ^1^University of California, Neurology, Bioengineering and Therapeutic Sciences, San Francisco, California, United States of America; ^2^University of California, Statistics, Electrical Engineering and Computer Sciences, San Francisco, California, United States of America

##### **Correspondence:** Reza Abbasi-Asl (reza.abbasiasl@ucsf.edu)

*BMC Neuroscience* 2020, **21(Suppl 1)**:P219

Characterizing the functions of neurons in visual cortex is a central problem in visual sensory processing. Along the ventral visual pathway, functions of the neurons in the cortical area V4 are less understood compared to early visual areas V1 and V2. This is primarily because of V4 neurons’ highly nonlinear response properties. As a consequence, building predictive models for these neurons has been one of the challenging tasks in computational neuroscience. Recently, models based on convolutional neural networks (CNNs) have shown promise in predicting the activity of V4 neurons. More importantly, interpreting CNN-based models has offered tools to understand V4 neurons’ functional properties through visualizing their pattern selectivity. These interpretations, however, are based on models with hundreds of convolutional filters. Therefore, it is challenging to present a sparse set of filter bases to model each V4 neuron. To address this limitation, we propose two algorithms to remove redundant filters in the CNN-based models of V4 neurons. First, CAR compression that prunes filters from the CNN based on the filter’s contribution to the image classification accuracy. CAR is a greedy compression scheme to obtain smaller and more interpretable CNNs, while achieving close to original accuracy. Second, RAR compression that prunes filters based on their contribution to the neural response prediction accuracy. Both CAR and RAR provide a new set of simpler accurate models for V4 neurons. These models achieve almost similar (for CAR) or higher (for RAR) accuracy compared to the original model. Using the compressed models, we are able to find a sparse set of filters that accurately model V4 neurons. We identify and visualize a total of 25 filters in AlexNet that accurately model V4 neurons in non-human primates. The features extracted by these 25 filters can predict the spike rates of 71 V4 neurons with an average correlation coefficient of 51%. By visualizing the patterns selected by these models, we further demonstrate that V4 neurons are modeled via curvature or texture filters, as well as other more complicated filters. Our results present one of the first efforts to bridge between large-scale convolutional models of neurons and interpretable sparse networks.

## P220 Extracellular positive spikes in cat primary visual cortex may correspond from the axons of cells originating from the thalamus

### Shi Hai Sun^1^, Ali Almasi^1^, Hamish Meffin^2^, Michael R Ibbotson^1^, Molis Yunzab^1^

#### ^1^Australian College of Optometry, The National Vision Research Institute, Melbourne, Australia; ^2^University of Melbourne, Biomedical Engineering, Melbourne, Australia

##### **Correspondence:** Shi Hai Sun (sunsh@student.unimelb.edu.au)

*BMC Neuroscience* 2020, **21(Suppl 1)**:P220

Extracellular spike waveforms from recordings in the visual cortex have been classified into either regular spiking (RS) or fast spiking (FS) units, which are commonly associated with excitatory and inhibitory neurons, respectively. While both these types of spike waveforms are negative-dominant, we show that there are also distinct classes with positive-dominant waveforms, which are not regularly reported. The spatial receptive fields (RFs) of these different spike waveform types were estimated and we found that each spike type had distinctly different RF structure.

In this study, we systemically classified 1,225 single units (SUs) in cat visual area 17 (V1) into five categories by the shape of their spike waveforms: RS units (53%, n =645) which are biphasic, have a dominant negative peak, and a slow declining slope at the end of the waveform; FS units (18%, n = 226) which are biphasic, have a dominant negative peak, and a fast declining slope at the end of the waveform; triphasic spiking units (TS, 10%, n = 122) which have a positive first peak that is >10% of the negative peak, followed by a large negative peak and then a smaller positive peak; compound spiking units (CS, 7%, n = 82) which are also triphasic but with a significantly longer waveform; and positive spiking units (PS, 12%, n = 150) which have a positive peak greater than the negative peak.

Of these 1,225 SUs, 341 had their spatial RFs estimated as the spatial filters in a powerful model-based analysis method to objectively determine the RF characteristics of the recorded units, which revealed the existence of non-oriented and blob-like (orientation bandwidth > 110°) and oriented and Gabor-like RFs (orientation bandwidth < 90°). RS and FS units had mostly oriented RFs (94%, and 96%, respectively), TS and CS units have an even mixture of both RF types (47% oriented and 53% non-oriented, and 56% oriented and 44% non-oriented, respectively), while PS units had mostly non-oriented RFs (83% blob-like).

Units with non-oriented RFs have similar spatial structures to the centre-surround RFs reported in the thalamus, suggesting that units with non-oriented RFs could have originated from the sub-cortical area. We calculated several response properties that are statistically distinguishable between cortical and thalamic neural populations: spike-rate, burstiness, and response latency. On average, PS units had significantly higher spike-rate (t-test, p < 0.01), significantly higher proportion of burst spikes (p < 0.001), and significantly shorter response latency (p < 0.001) to RS and FS units. We also recorded from V1 before and after the application of muscimol (a cortical silencer) and found that PS units remained while RS and FS units did not.

Thus, our results suggest that PS units, which have mostly non-oriented RFs, thalamic-like response properties and remain after cortical silencing, are recordings of axons originating from the thalamus. RS and FS units correspond to cortical neurons, which have mostly orientated RFs and do not remain after cortical silencing. Our results suggest that cortically implanted electrodes are able to record activity simultaneously from thalamic axon afferents and from the somas of cortical neurons, thus allowing us to assess connectivity between two brain areas while only recording from one area.

## P221 Visualization of pathways of potential neurostructures in neurorehabilitation period based on MRI data processing

### Margarita Zaleshina^1^, Alexander Zaleshin^2^

#### ^1^Moscow Institute of Physics and Technology, Moscow, Russia; ^2^Institute of Higher Nervous Activity and Neurophysiology, Moscow, Russia

##### **Correspondence:** Margarita Zaleshina (zaleshina@gmail.com)

*BMC Neuroscience* 2020, **21(Suppl 1)**:P221

Growth, formation and movement of biological structures are determined by characteristics of the environment and requirements for obtaining external resources. Likewise, the topological organization of the brain, consisting of a set of neurostructures, has a direct effect on the brain’s ability to perceive or process data. Additionally, localized damage to a small part of the brain will result in specific disturbances of isolated mental facilities, such as perception or movement [1]. Many researchers are currently studying regeneration and formation of a new spatial filling of tissue at the sites of damage. The individual variability of the anatomy and connectivity of the brain affects the formation of its structure [2]. Studies of both tissue features and the distribution and orientation of individual components are widely used to visualize the microstructures of individual brain regions or to determine the locations of biomarkers [3]. At the same time, it can be shown that neurorehabilitation depends not only on the characteristics of the whole brain, but also on the particular features of the distinct area where growth and recovery occur directly.

In this work, we study cases of regeneration of cortical neurostructures, when the damaged area is filled with new elements for a long period of time. The analysis compares the calculated growth directions of neurostructures, the calculated trajectories of their growth, taking into account the existing environment, and the real growth paths identified on the basis of MRI data.

Our study takes into account that the ways of formation of neural structures during neurorehabilitation have two main characteristics that differ in scale and in details. The first characteristic is the average direction of the formation of new neurostructures. Such a direction, as a whole, is caused by an increase in the “favorableness” of the environment in which growth occurs. The second characteristic is a detailed following of external elements in the existing biological environment, that is, on the one hand, rounding obstacles, and on the other hand, the use of convenient “corridors” for growth and advancement (Fig. 1).

Data packages (fMRI) are collected from Human Connectome Project (https://www.humanconnectome.org/data/). These fMRI could be converted to diffusion-weighted images (dMRI), which are used for tractography analysis and for investigate the heterogeneity of microstructural features.

The study uses spatial data analysis, which calculates the main corridors and growth directions, taking into account the available cortical volume filling. Data at the boundaries of tissue are excluded from analysis to minimize the impact of partial volume averaging with surrounding tissues.

**Fig. 1:**
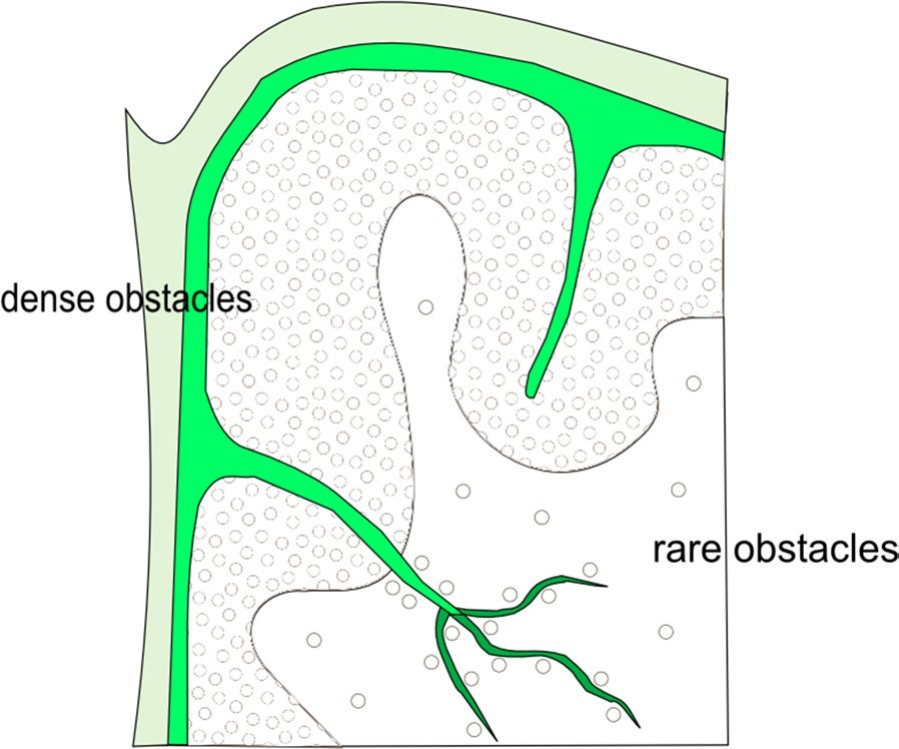
Formation of growth paths depending on the density of obstacles

**References**

1. Eickhoff SB, Constable RT, Yeo BT. Topographic organization of the cerebral cortex and brain cartography. Neuroimage. 2018; 170: 332-47.
2. Maier-Hein L, et al. Why rankings of biomedical image analysis competitions should be interpreted with care. Nature Communications. 2018; 9(1): 1-3.
3. Fick RH, Wassermann D, Deriche R. The dmipy toolbox: Diffusion mri multi-compartment modeling and microstructure recovery made easy. Frontiers in Neuroinformatics. 2019; 13: 64.

## P222 High-resolution connectivity analyses

### Sina M Lakouraj^1^, Vanessa Cropley^2^, Ye Tian^3^, Andrew Zalesky^1,3^

#### ^1^The University of Melbourne, Department of Biomedical Engineering, Melbourne, Australia; ^2^Melbourne Neuropsychiatry Centre, Melbourne, Australia; ^3^The University of Melbourne, Department of Psychiatry, Melbourne, Australia

##### **Correspondence:** Sina M Lakouraj (sina.mansour.lakouraj@gmail.com)

*BMC Neuroscience* 2020, **21(Suppl 1)**:P222

**Introduction:** No two brains are alike. Neuroimaging can be used to elucidate the neural basis of human identity and to map neural correlates of human behavior and cognition. While most neuroimaging features are commonly studied in the highest resolution provided by the scanner, brain connectivity studies are traditionally conducted in a lower resolution of a predefined atlas. The aim of this study was to contrast the identification (fingerprinting) and behavior prediction capabilities of the atlas-based brain networks with a novel ultra-high-resolution model of brain networks. This comparison will explore the potential gains of conducting brain connectivity analyses in a higher resolution.

**Methods:** We analyzed neuroimaging and behavioral data acquired from 1000 individuals participating in the Human Connectome Project (HCP). These individuals formed a “test” group. A repeated MRI scan on a different day from 42 of these individuals formed a “retest” group. Structural connectivity was mapped using probabilistic tractography of Diffusion MRI. Functional connectivity was mapped using resting-state functional MRI. Additionally, surface maps for cortical measures of thickness, curvature, sulcal depth, and myelination were sourced from the HCP. Hence, each scan was associated with 6 different neuroimaging characteristics of structure, morphology, and connectivity, each of which was mapped at the higher resolution of vertices (~32,000 nodes per hemisphere) as well as regions comprising an established atlas (180 regions per hemisphere), yielding 12 total measures.

For each measure, a similarity metric for all scan pairs was computed. This similarity information was used to quantify the extent of identifiable information captured by neuroimaging measures. We computed the effect size difference in intra- and inter-subject similarity distributions as an identifiability metric. Hence, higher effect size differences translated to higher precision in identification. To capture the extent of behavioral associations of every measure, independent component analysis was used to decompose 109 behavioral measures sourced from HCP to five core continuous dimensions, characterizing cognitive performance, illicit substance use, tobacco use, personality-emotion traits, and mental health. Variance component modeling was used to evaluate the extent to which each neuroimaging measure could explain individual variation in each behavioral dimension.

**Results:** Comparing the correlates of neural identity and behavior in atlas-based models with the ultra-high-resolution alternative revealed the extent of information gain achieved by the increase in spatial resolution (Fig. 1). Our findings show behavioral associations of all neuroimaging modalities significantly increased as a result of the increase in spatial resolution. In particular, behavior associations of structural connectivity, functional connectivity, and cortical thickness benefitted the most from ultra-high-resolution analyses. The neural correlates of individual identity were also better detected in higher resolution, especially for structural connectivity and all measures of morphology (cortical curvature, thickness, and sulcal depth). The identification improvements of functional connectivity and myelination were minimal. We proposed a novel model of high-resolution structural brain networks that surpasses the ability of atlas-based alternatives in both identification and behavior explanation of individuals.

**Fig. 1:**
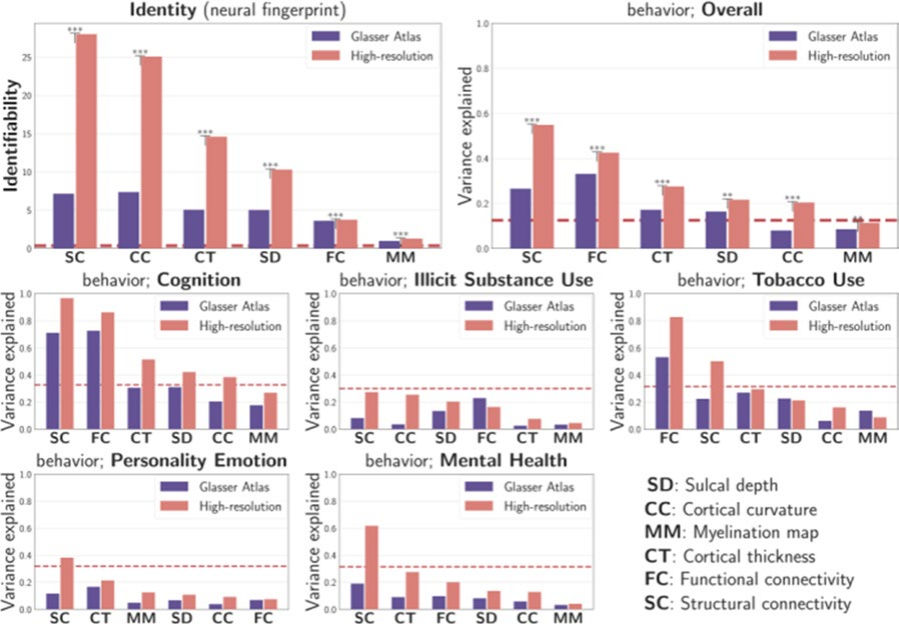
Identification effect size (top left) and overall behavior association (top right) captured at the resolution of a well-established cortical atlas (violet), and a high-resolution alternative (pink). Associations are decomposed to data-driven components of behavior (bottom row). The dashed lines present the 95% confidence interval. Asterisks quantify effect sizes (***: large, **: medium, *: small)

## P223 Less is more: wiring-economical modular networks support self-sustained firing-economical neural avalanches for efficient processing

### Shengjun Wang^1^, Junhao Liang^2^, Changsong Zhou^3^

#### ^1^Shananxi Normal University, Physics, Xi’An, China; ^2^Hong Kong Baptist University, Hong Kong, China; ^3^Hong Kong Baptist University, Physics, Hong Kong, China

##### **Correspondence:** Changsong Zhou (cszhou@hkbu.edu.hk)

*BMC Neuroscience* 2020, **21(Suppl 1)**:P223

Complex neural network in the brain is remarkably cost-efficient while the basic mechanisms underlying its structure-dynamics economy are not clear. Here we study the intricate interplay between wiring and running cost with modular network topology, self-sustained activity and critical avalanche dynamical mode in biologically plausible excitation-inhibition balanced spatial neuronal network. When rewiring the initially wiring-expensive sparse random network gradually to wiring-economical modular network, its self-sustained dynamics changes from asynchronous spiking to critical avalanches state with strongly reduced firing rate and greatly enhanced response sensitivity to transient stimuli. Thus, the system can counter intuitively achieve much more functional values with much less costs in both wiring and firing. The dynamic mechanism is explained as a proximity to Hopf bifurcation in the macroscopic mean-field in separated modules when increasing the connection density. Our work reveals the generic mechanism underlying the cost-economical structural organization and function-efficient critical dynamics of neural systems, providing insights to brain-inspired efficient computational designs.

## P224 Hopf bifurcation in mean field explains critical avalanches in excitation-inhibition balanced neuronal networks: a mechanism for multiscale variability

### Junhao Liang^1^, Tianshou Zhou^2^, Changsong Zhou^3^

#### ^1^Hong Kong Baptist University, Hong Kong, China; ^2^Sun Yat-sen University, School of Mathematics, Guang Zhou, China; ^3^Hong Kong Baptist University, Physics, Hong Kong, China

##### **Correspondence:** Changsong Zhou (cszhou@hkbu.edu.hk)

*BMC Neuroscience* 2020, **21(Suppl 1)**:P224

Cortical neural circuits display highly irregular spiking in individual neurons but variably sized collective firing, oscillations and critical avalanches at the population level, all of which have functional importance for information processing. Theoretically, the balance of excitation and inhibition inputs is thought to account for spiking irregularity and critical avalanches may originate from an underlying phase transition. However, the theoretical reconciliation of these multilevel dynamic aspects remains an open question. Herein, we show that excitation-inhibition (E-I) balanced network with synaptic kinetics can maintain irregular spiking dynamics with different levels of synchrony and critical avalanches emerge near the synchronous transition point. The mechanism is unveiled by a novel mean-field theory that derives the field equations governing the network macroscopic dynamics. It reveals that the E-I balanced state of the network manifesting irregular individual spiking is characterized by a macroscopic stable state, which can be either a fixed point or a periodic motion and the transition is predicted by a Hopf bifurcation in the macroscopic field. Furthermore, these multiscale variable behaviours can be jointly observed in the spontaneous activities of mouse cortical slice *in vitro*, indicating universality of the theoretical prediction. Our theory unveils the mechanism that permits complex neural activities in different spatiotemporal scales to coexist and elucidates a possible origin of the criticality of neural systems. It also provides a theoretical framework for analyzing the macroscopic dynamics of E-I balanced networks and its relationship to the microscopic counterparts, which can be useful for large-scale modeling and computation of cortical dynamics.

## P225 Functional identification of the odorant transduction process of Drosophila olfactory sensory neurons

### Aurel A Lazar, Tingkai Liu, Yiyin Zhou

#### Columbia University, Electrical Engineering, New York, United States of America

##### **Correspondence:** Tingkai Liu (tl2747@columbia.edu)

*BMC Neuroscience* 2020, **21(Suppl 1)**:P225

A recent empirical model of the olfactory sensory neurons (OSNs) described on the molecular level the mechanics of the Odorant Transduction Process (OTP, Fig. 1A, top) [1]. A system of nonlinear differential equations modeling the OTP in cascade with a biological spike generator successfully captured the experimentally observed responses of OSNs.

**Fig. 1:**
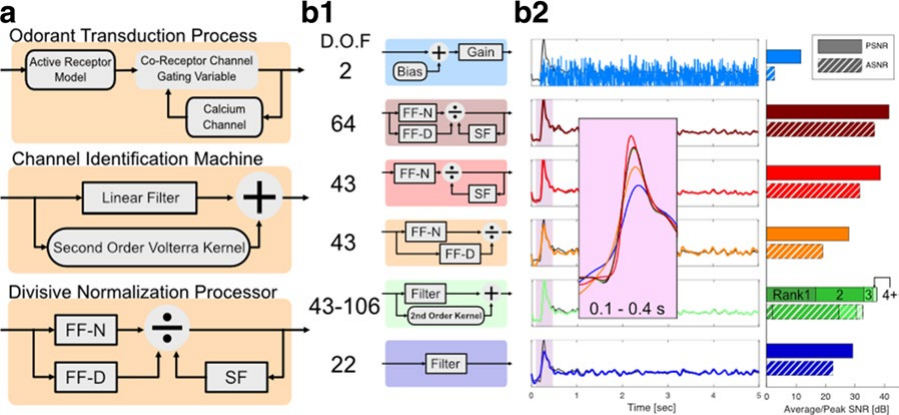
**A** Model architectures of OTP, CIM and DNP. **B1** Instantiations of the identification circuits and their corresponding degree of freedom. **B2** Comparison between output of OTP (in black) and identified models. Peak-SNRs between original and identified outputs are shown to the right. **B2 inset** Zoomed in view of transient responses

Here we functionally identify the OTP model on the algorithmic level with two state-of-the-art system identification methods (i) Channel Identification Machines (CIMs) [2] (Fig. 1A, middle) and (ii) Divisive Normalization Processors (DNPs) [3, 4] (Fig. 1A, bottom). We examined 5 model structures with different degrees of freedom under these two model architectures (Fig. 1B1).

Overall, the full temporal DNP successfully captured the OTP dynamics with the highest average and peak signal-to-noise ratios (ASNR & PSNR) of 36.6 dB and 41.5 dB respectively when predicting the response to a novel stimulus (Fig. 1B2). This is 6 dB higher than the identified CIM model of comparable model complexity (rank 2), and 4 dB higher than the full-rank CIM. While the linear filter alone identified by the CIM has a PSNR of 29.2 dB that is comparable to the prediction given by the DNP with only FF-D processor, a closer examination revealed that it did not predict very well the transient responses at the onset of the stimulus that is critical in the context of olfactory encoding. The highly nonlinear transient response is nonetheless well captured by the full temporal DNP (Fig. 1B2, inset).

Furthermore, we observed that the FF-N and FF-D processors identified in the full temporal DNP consistently resemble each other in their functional forms, with FF-N generally having higher 3 dB Bandwidth than FF-D (data not shown). This prompted us to closely examine the mechanism that gives rise to the OTP’s 2D encoding property where the output of OTP model captures both the odorant concentration and concentration gradient. We instantiated a DNP with FF-N and FF-D processors modeled as linear lowpass filters with different bandwidths. Surprisingly, the simple model is able to capture the essential 2D encoding across all stimuli described in [1] (data not shown), suggesting a general approach that enables simultaneous encoding of input signal’s amplitude and gradient with divisive normalization.

Concluding, by evaluating two functional identification methods, we established a functional description of the empirical OTP model using divisive normalization processors. In addition, the identified DNP provided insights on the form of divisive normalization that leads to the simultaneous encoding of both the concentration and concentration gradient. Divisive processing has previously been used as a key component in describing the functional dynamics of blowfly photoreceptors [5], suggesting that DNPs may be universally employed for the identification of nonlinear processing in the early sensory systems.

**Acknowledgments**: The research reported here was supported by AFOSR under grant #FA9550-16-1-0410 and by DARPA under contract #HR0011-19-9-0035.

**References**

1. Lazar AA, Yeh CH. A molecular odorant transduction model and the complexity of spatio-temporal encoding in the Drosophila antenna. PLoS Computational Biology. 2020; 16(4): e1007751.
2. Lazar AA, Slutskiy YB. Spiking neural circuits with dendritic stimulus processors. Journal of Computational Neuroscience. 2015; 38(1): 1-24.
3. Lazar AA, Ukani NH, Zhou Y. Sparse identification of contrast gain control in the fruit fly photoreceptor and amacrine cell layer. The Journal of Mathematical Neuroscience. 2020; 10(1): 1-35.
4. Brittain C, et al. Severity dependent distribution of impairments in PSP and CBS: Interactive visualizations. Parkinsonism & Related Disorders. 2019; 60: 138-45.
5. Van Hateren JH, Snippe HP. Information theoretical evaluation of parametric models of gain control in blowfly photoreceptor cells. Vision Research. 2001; 41(14): 1851-65.

